# Bioinformatic analysis of meningococcal Msf and Opc to inform vaccine antigen design

**DOI:** 10.1371/journal.pone.0193940

**Published:** 2018-03-16

**Authors:** Clio A. Andreae, Richard B. Sessions, Mumtaz Virji, Darryl. J. Hill

**Affiliations:** 1 School of Cellular and Molecular Medicine, University of Bristol, Bristol, United Kingdom; 2 School of Biochemistry, University of Bristol, Bristol, United Kingdom; Universidad Nacional de la Plata, ARGENTINA

## Abstract

*Neisseria meningitidis* is an antigenically and genetically variable Gram-negative bacterium and a causative agent of meningococcal meningitis and septicaemia. Meningococci encode many outer membrane proteins, including Opa, Opc, Msf, fHbp and NadA, identified as being involved in colonisation of the host and evasion of the immune response. Although vaccines are available for the prevention of some types of meningococcal disease, none currently offer universal protection. We have used sequences within the *Neisseria* PubMLST database to determine the variability of *msf* and *opc* in 6,500 isolates. *In-silico* analysis revealed that although *opc* is highly conserved, it is not present in all isolates, with most isolates in clonal complex ST-11 lacking a functional *opc*. In comparison, *msf* is found in all meningococcal isolates, and displays diversity in the N-terminal domain. We identified 20 distinct Msf sequence variants (Msf SV), associated with differences in number of residues within the putative Vn binding motifs. Moreover, we showed distinct correlations with certain Msf SVs and isolates associated with either hyperinvasive lineages or those clonal complexes associated with a carriage state. We have demonstrated differences in Vn binding between three Msf SVs and generated a cross reactive Msf polyclonal antibody. Our study has highlighted the importance of using large datasets to inform vaccine development and provide further information on the antigenic diversity exhibited by *N*. *meningitidis*.

## Introduction

*Neisseria meningitidis* is an important cause of meningococcal meningitis and septicaemia worldwide. Case numbers vary geographically with incidence rates up to 14 per 100,000 population across Europe, compared to as much as 1,000 per 100,000 population during epidemics in sub-Saharan Africa [1]. There are 13 distinct serogroups of *N*. *meningitidis*, characterised by differences in the capsular polysaccharide, with six (A, B, C, W-135, X and Y) being associated with invasive disease. Protein-polysaccharide conjugate vaccines are currently available for the prevention of meningococcal disease caused by serogroups A, C, W-135 and Y [2, 3]. However, polysaccharide conjugate vaccines have serious limitations as they are not effective against strains possessing the serogroup B capsule for example, due to its similarity to human neuraminic acid [4]. To combat serogroup B disease, a four component vaccine (containing Neisserial adhesin A (NadA), Neisserial Heparin Binding Antigen (NHBA), factor H binding protein (fHbp), and the outer-membrane vesicles from the New Zealand outbreak strain NZ98/254) known as Bexsero, was developed [5] and has been licensed for use in the United Kingdom. Bexsero is estimated to cover 66 to 76% of circulating serogroup B isolates due to antigenic variation of the components present in the vaccine [6, 7]. Thus, whilst a number of meningococcal vaccines are available for the prevention of invasive disease, none currently provide universal protection [2].

*N*. *meningitidis* strains are clustered by multi-locus sequencing typing (MLST) into distinct clonal complexes (cc), comprising of closely related sequence types (ST) [8]. MLST analysis has highlighted the variability exhibited by meningococci and has led to the identification of hyperinvasive lineages (e.g. cc ST-8, ST-269, ST-41/44 and ST-11) which are more commonly associated with invasive meningococcal disease.

Residing in the human nasopharynx, *N*. *meningitidis* can disseminate to cause invasive disease following entry into the blood and central nervous system [9, 10]. Survival in the body depends on the ability of these bacteria to avoid killing by the innate and adaptive immune defences. *N*. *meningitidis* has a range of surface expressed components, aiding in colonisation and evasion of the host immune system [9, 11–13]. Such components include, Opc, fHbp and the trimeric autotransporter adhesins, NadA and Meningococcal surface fibril (Msf; also referred to as Neisseria *hia* homologue A, NhhA) [14–16]. Opc is an integral outer membrane protein, composed of a 10-stranded β-barrel structure with 5 surface exposed loops [17, 18], required for adherence to, and invasion of eukaryotic cells [12, 19]. Opc is also involved in the evasion of the human immune response, through its ability to bind to the negative regulator of complement, vitronectin (Vn) [20, 21]. Loop 2 of Opc is associated with interaction with Vn and monoclonal antibodies binding to this loop results in significant inhibition of meningococcal binding [17, 21]. Opc binds to the activated form of vitronectin, requiring sulphated tyrosine Y (56) and Y (59) of the Vn connecting region [21]. Several studies have identified Opc as a potential vaccine candidate, due to its contribution to bactericidal activity [22–24].

Msf is a trimeric autotransporter adhesin (TAA) exhibiting similarity to both Hia and Hsf from *Haemophilus influenzae* [25–27]. Such proteins are multifunctional surface proteins involved in biofilm formation, cell adhesion and invasion, auto-aggregation, and survival of complement mediated killing. Sharing common structural architecture, TAA’s are often composed of three distinct domains; the N-terminal head domain, stalk and β-barrel required to anchor the protein to the outer membrane [28, 29]. Msf supports adhesion and invasion of epithelial cells [14, 15, 30], as well as modulation of the immune response ensuring maintenance of carriage [31]. In addition to the outer membrane protein Opc [21], Msf facilitates meningococcal escape of complement-mediated killing via binding to Vn [15, 32], and is known to bind to matrix proteins such as heparan sulphate and laminin [30]. Although Msf expression is variable between strains of *N*. *meningitidis* [25], its expression is thought to influence host-pathogen interactions, with its expression being induced in response to epithelial attachment [30], and in the presence of different iron presentations [33].

The Neisseria PubMLST database (https://pubmlst.org/neisseria/) is an online repository [34] containing genome and isolate information from a large number of *N*. *meningitidis* isolates from all over the globe. This database has been used to determine population structuring of meningococcal antigens [35]. In our study, we used the PubMLST database to determine the sequence variability of Msf and Opc in 6,500 meningococcal isolates, and determined their distribution within different serogroups, and clonal complexes. Generation of this database has informed this and other *in vitro* studies and is helping to determine the potential for these antigens to be used as meningococcal vaccine candidates.

## Materials and methods

### Neisseria PubMLST database analysis

The *Neisseria* PubMLST isolate BIGSdb (https://pubmlst.org/neisseria/) database was used to determine the conservation of *msf* and *opc* between meningococcal isolates [36]. BLAST analysis was performed on 6,500 *Neisseria meningitidis* isolates using *N*. *meningitidis* strain MC58 NMB0992 (*msf*) or NMB1053 (*opc*) as our reference genes. Additional information on each isolates serogroup, clonal complex, disease state and country of origin was also obtained from the PubMLST BIGSdb.

### Alignments and protein sequence analysis

Both the *msf* and *opc* nucleotide sequences from each isolate were translated and aligned by MUSCLE using Mega6 software prior to further manual analysis using BioEdit software. Msf sequence variants (SV) were described based on increasing number of amino acids between the conserved motifs previously described to be required for vitronectin binding [32], and were manually assigned for each of the 6,500 meningococcal isolates in our dataset. Those isolates which only displayed around 28–30% identity to NMB1053 (*opc*) from MC58, or those having a ‘truncated’ gene (displaying around 45% identity to MC58 NMB1053), were annotated as Opc negative (–ve). All other isolates showing with *opc* genes showing 97–100% sequence identity to NMB1053 we assigned as Opc positive (+ve).

The daTAA online software (https://toolkit.tuebingen.mpg.de/dataa) was used to predict the representative structure of the trimeric autotransporter adhesins from *N*. *meningitidis* NMB0992 (Msf), and *Haemophilus influenzae* Hia (AAL79953) and Hsf (AAX33325) [37].

### Bacterial strains and media

All *N*. *meningitidis* strains used in this study were grown on brain heart infusion media supplemented with 10% horse blood solidified with 1.5% bacteriological agar (HBHI agar) at 37°C in 5% CO_2_. *Escherichia coli* for the expression of recombinant proteins were grown in Lysogeny broth supplemented with 100 μg/mL ampicillin at 200 rpm (37°C). *N*. *meningitidis* whole cell lysates were created following resuspension of colonies in distilled water containing 1 x protein inhibitor cocktail (PIC). Samples were freeze thawed three times and stored at -80°C until required.

### N-terminal Msf recombinant protein production

Recombinant proteins of the N-terminal domain, amino acid residues 49–189 of Msf-SV types (SV-1, SV-2, and SV-5) and amino acid residues 31–178 of Msf SV-8, were expressed and purified from *E*. *coli* BL21 (DE3) expression cells. Msf recombinants where cloned into pOPINF using the Infusion cloning kit (Clontech) following PCR using primers shown in Table 1. The corresponding plasmid constructs were then transformed into BL21 (DE3) expression cells, induced using 1 mM IPTG overnight at 20°C, and purified following a two-step method involving initial purification through Ni-NTA beads using an imidazole gradient, followed by ion exchange using a Q sepharose HiTrap column against a NaCl gradient (0–500 mM). This generated Msf N-terminal recombinant proteins referred to as rMsfSV-1_49-189_, rMsfSV-2_49-186_, rMsfSV-5_49-189,_ and rMsfSV-8_31-178._

**Table 1:** Primers used to clone the N-terminal domain of different Msf sequence.

Primer	Sequence (5’-3’)
MC58-MsfFWD	AAGTTCTGTTTCAGGGCCCGGCAAGTGCTAACAATGAAGAGC
PMC3-MsfFWD	AAGTTCTGTTTCAGGGCCCGGCGAATGCTACCGATGAAGATC
PMC8-MsfFWD	AAGTTCTGTTTCAGGGCCCGGCGAATGCTACCGATACCGATG
M07 240646-MsfFWD	AAGTTCTGTTTCAGGGCCCGCGAATGCTACCGATGAAGATGA
pOPINF_MsfRV	ATGGTCTAGAAAGCTTTACGTATCGGTCAAAGTCGAACC

Primer sequences to allow for cloning into pOPINF are underlined for each forward and reverse primer.

### Antisera against N-terminal domain of Msf SV-1, SV-2 and SV-5

Antisera was produced by Cambridge Research Biochemicals following a 77 day immunisation protocol with 2 mg of an equal mix of N-terminal recombinant proteins of Msf SV-1, SV-2 and SV-5 using Freunds complete adjuvant.

### Enzyme-linked immunosorbent assay (ELISA)

ELISA plates (96 well, Immulon 2HB) were coated overnight with 100 μL of 3 μM Opc or Msf N-terminal recombinant proteins (rMsfSV-1_49-189_, rMsfSV-2_49-186_, or rMsfSV-5_49-189,_) in 50 mM carbonate buffer (pH 9.6). Control wells were coated with 3% BSA only. Unbound protein was removed by washing with ELISA wash (154 mM NaCl containing 0.05% Tween-20). Wells were blocked with 3% BSA in phosphate buffered saline containing 0.05% Tween-20 (PBST) for 1h at RT. The wells were then incubated with a final concentration of 2 μg/mL Vn for 1h at RT. Unbound Vn was removed by washing with ELISA wash and the bound Vn was detected using anti-vitronectin MsX (8E6 clone, Millipore) and anti-mouse alkaline phosphatase secondary antibody. ELISA plates were developed using SigmaFast p-nitrophenyl phosphate substrate and the absorbance was measured at 405 nm.

### Molecular modelling of the N-terminal domain of Msf

Molecular models of the N-terminal domain of representative sequences of Msf sequence variant SV-5 and SV-8 were constructed to determine what effect additions of repeating NTN motifs had on the structure of the vitronectin binding domain. The 10 amino acid (Msf SV-5) and 14 amino acid (Msf SV-8) loop variants were modelled using our original structural model of Msf [32], with the insertions built into the N-terminal end of the strand between residues 120–123 of the canonical or ‘wild-type’ structure (e.g. the sequence of Msf from MC58).

### Statistical analysis

Statistical analysis of our dataset of 6,500 isolates was performed using the GraphPad Prism 7 software. Wilson/Brown analysis was used to determine the proportion of isolates (expressed as a percentage) being either *opc* negative or positive, or possessing a distinct Msf sequence variant type, with confidence intervals (CI) determined to 95% assuming a binomial distribution.

### Ethics statement

Human serum was used as a source of endogenous complement in supplementary figures. Sera were obtained from the UK NHS Blood and Transplant Service (NHSBT) and derived from blood donated with consent from healthy adults, where it was identified as surplus to clinical requirement or unsuitable for therapeutic use in line with NHSBT processes: http://hospital.blood.co.uk/components/non-clinical-issue/.

No animals were used in this study. However, animal products used were purchased from commercial suppliers and were produced conforming to appropriate national guidelines. No animals were used in this study. However, animal products used were purchased from commercial suppliers and were produced conforming to appropriate national guidelines.

## Results

### Opc, although not present in all meningococcal isolates, is highly conserved

The role of Opc in serum resistance and adherence to and invasion of epithelial and endothelial cells is well established [12, 21]. Our analysis of 6,500 meningococcal isolates revealed Opc to be highly conserved, with each Opc positive isolate displaying 97–100% identity to NMB1053 (Table 1). Out of 6,500 isolates 4,237 were Opc positive, with the remaining 2,263 isolates either lacking or having a truncated form of Opc (Table 2). The majority of *opc* negative isolates, which displayed only 28 to 30% sequence identity to NMB1053 due to gene truncation, belonged to serogroups C and W and were associated with the presence of a premature stop codon. Most of the other serogroups were Opc positive (Table 2). Notably, genome sequence information is not available for all isolates, so it is possible many strains possessing *opc* gene sequences that appeared truncated or lacking from our analysis were in fact Opc positive, Opc prevalence could therefore be underestimated.

**Table 2:** Diversity of Opc in *N*. *meningitidis* isolates.

Serogroup	Opc+ ^a^	Opc- ^b^
**A**	229	2
**B**	2294	555
**C**	302	601
**W**	132	785
**Y**	625	2
**X**	28	3
**NG**	597	309
**E**	18	3
**H**	1	0
**Z**	11	3

^a^ Meningococcal isolates *opc* displaying over 97.7% sequence identity to NMB1053 are classed as Opc positive (+).

^b^ Those isolates with an either lacking or having a truncated *opc* (exhibiting 28.9 to 45.7% identity to NMB1053) were annotated as Opc negative (-) isolates.

Meningococcal isolates associated with invasive disease are largely grouped into hyperinvasive lineages (e.g. clonal complexes (cc) ST-1, ST-4, ST-5, ST-8, ST-11, ST-32, ST-41/44, and ST-269) [38–40]. In comparison, those isolates predominantly associated with carriage tending to be associated with clonal complexes distinct from those associated with invasive disease e.g. ST-23 and ST-35 [38, 41]. Our *in-silico* analysis revealed 67.12% (CI = 65.2–69.03%) of *opc* negative isolates to be in cc ST-11, 12.4% (CI = 11.12–13.84%) to be cc ST-213 and 4.73% (CI = 3.93–5.68%) belonged to cc ST-461. This absence of *opc* within isolates from cc ST-11 is thought to contribute to the inability for these isolates to cause infection of the meninges, with isolates from cc ST-11 primarily associated with severe sepsis [42]. Analysis on isolates from hyperinvasive lineages and clonal complexes positively associated with carriage, revealed isolates from invasive disease cc ST-8 (100%; CI = 88.65–100%) and cc ST-11 (99.92%; CI 99.63–99.99% see S1 Table in Supplementary Information) to lack a functional copy of *opc*. In addition, all isolates from cc ST-213 (281 isolates), ST-8 (30 isolates), ST-334 (24 isolates), ST-53 (52 isolates), and 107/108 isolates from ST-461 are *opc* negative. In comparison 96–100% of isolates from hyperinvasive clonal complexes ST-1, ST-4, ST-5, ST-32 and ST-41/44 and those positively associated with carriage (cc ST-23, cc ST-35 and cc ST-60) were all Opc positive (see supplementary information S1 Table).

### Msf is found in all meningococcal isolates but displays variability within the N-terminal domain

**Fig 1:**
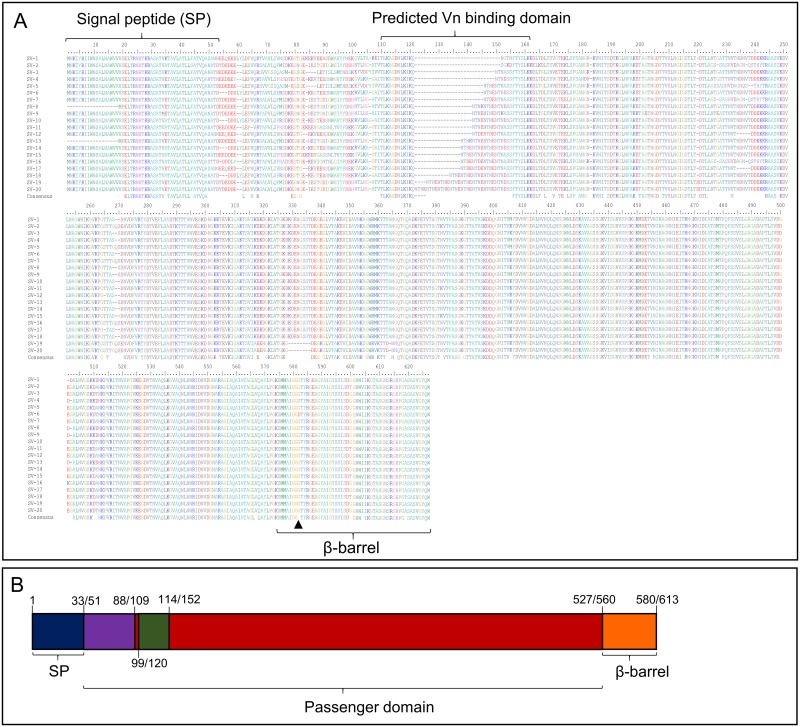
Amino acid sequence alignment of representative *N*. *meningitidis* Msf protein sequences from each defined Msf SV. A) Representative sequences of each of the Msf SV showing the locations of the signal peptide, predicted Vn binding domain and β-barrel. Different amino acid residues are represented with a different colour. Position of the G/D and G/S amino acid replacement within the β-barrel, seen in either Msf SV-6 or Msf SV-4, is denoted by the arrow. B) Diagrammatic representation of variable regions of Msf. Colours denote variable and conserved regions; blue—signal peptide (mostly conserved—exceptions seen with Msf SV-8 and SV-13); purple—N-terminal variable region; green—variable region between conserved motifs associated with Vn binding; and red—passenger domain (conserved). Approximate amino acid residue numbers for positions of signal peptide, variable regions are noted in the diagram. Includes location of amino acid residues from those SVs with ‘shorter’ Msf sequences. Variation in Msf size is largely due to differences within the N-terminal domain of the protein.

As Msf supports colonisation and evasion of the immune response, it has potential for inclusion in a meningococcal vaccine [25, 26, 32]. Bioinformatic analysis on 6,500 isolates revealed Msf to be highly conserved with the most diverse sequences displaying 84% sequence identity to NMB0992 from MC58 overall (Table 2). Much of the diversity observed is located between residues 51–200 of the Msf pre-protein (Fig 1).

The Vn binding domain of Msf is predicted to be located within residues 39–82 [32] which includes the conserved amino acid sequences TLKAGDNLKIKQ and FTYSLKK (Fig 1). These regions are found in all 6,500 meningococcal isolates analysed and are highly conserved (displaying 99–100% sequence identity; with one amino acid (underlined in bold) change seen in a small number of strains; e.g. TLK**S**GDNLKIKQ or TLKAG**G**NLKIKQ). Our analysis revealed there to be 20 distinct sequence variants (Msf SVs), with variations in both the number and sequence of amino acid resides between the conserved regions. Notably, a large proportion of these SVs are due to additions of repeating Asn (N), Thr (T) and Asn (N) residues (Table 3).

**Table 3:** Msf sequence variant distribution among meningococcal isolates.

Sequence between conserved TLKAGDNLKIKQ and FTYSLKK motifs	Msf sequence variant (SV)	No. of isolates	Percent distribution of Msf SV	Percent of Opc +ve isolates
**NGTN** ^a^*	SV-1	1,203	18%	88%
**SGKD** *	SV-2	2,199	34%	27%
**NTNASS**	SV-3	15	0.2%	87%
**NTNDSS** ^c^	SV-4	26	0.4%	100%
NTNENTNASS*	SV-5	684	10%	89%
**NTNENTNDSS** ^b^	SV-6	286	4%	98%
**NTDENTNASS**	SV-7	89	1.4%	54%
**NTNKNTNENTNDSS** ^d^	SV-8	434	6.7%	97%
**NTNENINENTKASS**	SV-9	14	0.2%	100%
**NTNENTNENTNDSS**	SV-10	223	3.4%	83%
**NTNENTNENTNASS**	SV-11	705	11%	97%
**NTDENTDENTNASS**	SV-12	522	8%	48%
**NTNKNTNENTNENTNDSS**^d^	SV-13	3	0.05%	100%
**NTNENTNENTNENTNDSS**	SV-14	30	0.5%	67%
**NTNENTNENTNENTNASS**	SV-15	32	0.5%	34%
**NTDENTDENTNENTNASS**	SV-16	15	0.2%	100%
**NTDENTDENTDENTNASS**	SV-17	7	0.11%	43%
**NTNENTNENTNENTNENTNDSS**	SV-18	5	0.1%	80%
**NTNENTNENTNENTNENTNENTNENTNDSS**	SV-19	4	0.1%	100%
**NTNENTNENTNENTNENTNENTNENTNENTNDSS**	SV-20	2	0.03%	100%

^a^ MC58 NMB0992 was used as query for initial BLAST search on PubMLST;

* Msf-SV used in further *in vitro* analysis

^b^ 260/286 (90.9%) with Msf SV-6 isolates with the **NTNENTNDSS** motif have a G/D replacement in the translocator domain (**SMMAIGGD** rather than **SMMAIGGG**). In addition, 3/30 SV-14 isolates also have this mutation. This naturally occurring single residue mutation, has been shown to affect trimerisation of the protein (monomeric rather than trimeric form), reduced adherence and cause a defect in surface localisation.

^c^ All isolates with Msf SV-4 have a serine replacement at the same location of the aspartate replacement in the β-barrel (arrow in Fig 1).

^d^ 92% (400/434) with SV-8 and all SV-13 *msf* nucleotide sequence contains a 14 bp deletion within the 51-aa signal peptide, taking the protein sequence out of frame. See text for more details.

Number of isolates analysed = 6,500.

Further analysis of our dataset revealed there to be a correlation between Msf SV and presence/absence of Opc (Table 3). This observation demonstrated a correlation with distinct clonal complexes. We found that 99.4% of isolates from the hyperinvasive ST-11 complex encode Msf SV-2 and are *opc* negative, whereas all 281 isolates from the carriage associated ST-213 complex are *opc* negative, with 89% (CI = 85.6–92.7%) of these encoding Msf SV-12. In addition, less than 50% of isolates encoding longer sequence variants Msf SV-12, Msf SV-15 and Msf SV-17 are Opc positive.

### *N*. *meningitidis* Msf SV types display distinct correlations with both serogroup and clonal complexes

**Fig 2:**
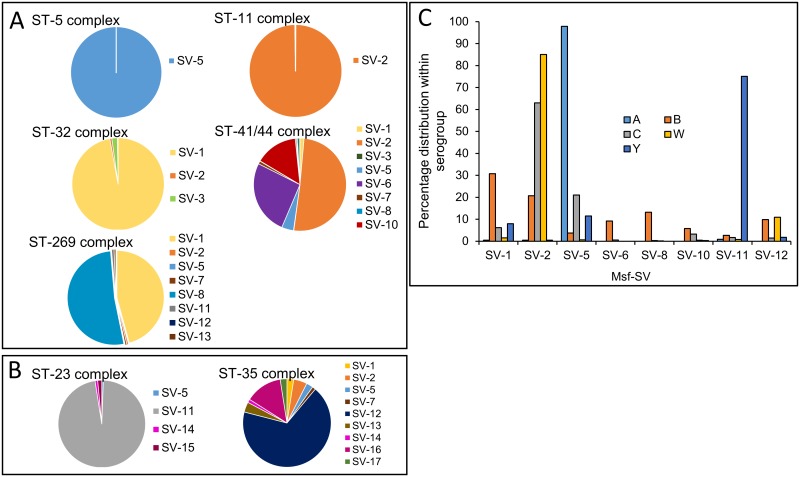
Msf sequence variants display correlations with distinct serogroups and clonal complexes of *N*. *meningitidis*. A) A large proportion of isolates from clonal complexes (cc) associated with invasive meningococcal disease (cc ST-5–144 isolates; ST-11–1520 isolates; ST-32–414 isolates; ST-41/44–984 isolates; and ST-269–670 isolates) show a predominance for Msf SV-1 (yellow), SV-2 (orange) or SV-5 (light blue). See supplementary information S2 Table for confidence intervals. B) In comparison those isolates falling into cc ST-23 (535 isolates) and ST-35 (81 isolates), which positively associate with carriage primarily encode either Msf SV-11 (grey) or Msf SV-12 (dark blue). C) Prevalence of the most common Msf SVs in invasive *N*. *meningitidis* isolates from serogroups A (blue), B (orange), C (green), W (red), and Y (green). Data shows results of a bioinformatic analysis on 6,500 meningococcal isolates, showing only those Msf SV’s that appear in more than 100 different isolates.

Being human restricted, variability in meningococcal surface proteins is thought to have occurred due to immune selective pressure, with several variants exhibiting non-random overlapping associations with distinct clonal complexes. Our analysis has revealed there to be an association of certain Msf sequence variants with isolates from hyperinvasive lineages and isolates from cc previously associated with a carriage state (Fig 2). The prevalence of Msf sequence variants was notably different between isolates from hyperinvasive lineages (Fig 2A), which primarily possess Msf SV-1, SV-2 and SV-5, and those associated with carriage which primarily have Msf SV-11 and SV-12 (Fig 2B). Analysis revealed that 99.5% of isolates from cc ST-11, predominantly serogroup C and W, were *opc* negative and encode Msf SV-2 (CI = 98.9–99.7%, see Fig 2 and S2 Table). In comparison, meningococcal serogroup A isolates, from cc ST-1, ST-4 and ST-5, encoded Msf SV-5 (Fig 2 and S2 Table). In addition, 151/161 isolates from cc ST-865, associated in our database with endemic isolates from South Africa, were Opc positive and encode Msf SV-5. In contrast, Msf SV-1 was largely found in serogroup B isolates and most commonly seen in isolates from cc ST-32 (33.4%; CI = 30.8–36.1%) and cc ST-269 (25.4%; CI = 23–28%; S1 Table). As seen with other surface antigens, serogroup B isolates from cc ST-41/44 complex, displayed a more diverse range of Msf SV’s. Although there does not seem to be a distinct Msf SV associated with ST-41/44 there is a predominance for Msf SV-2, as seen with 50.7% of isolates from this cc (Fig 2 and S2 Table).

In comparison to hyperinvasive lineages, those isolates from clonal complexes associated with a carriage state, such as cc ST-23 and ST-35, were more commonly found to encode Msf SV-11 and SV-12. For example, 95.14% (CI = 93–96.7%) of isolates from ST-23 encoded Msf SV-11, whereas 71.6% (CI = 61–80.3%) those from cc ST-35 encoded Msf SV-12 (S2 Table).

### Sequence analysis predicts certain Msf SVs to be non-functional, due to amino acid substitutions within the β-barrel, whilst some contain deletions within the signal peptide

Some meningococcal isolates have been shown to contain a naturally occurring single amino acid mutation within the β-barrel domain of Msf, specifically a change from a glycine (G) to an aspartate (D) [43] (see position 581 in Fig 1). This substitution was shown to influence the trimerisation of the protein, resulting in a monomeric form of Msf corresponding to reduced surface localisation, adhesion to host cells and reduction in antibody bactericidal killing by Msf specific antisera [43]. Our bioinformatic analysis has shown that this mutation predominantly occurs within those isolates encoding Msf SV-6. Moreover, 90.9% of isolates with Msf SV-6 have this mutation within the β-barrel, with 89.2% (CI = 85–92.3%; see S3 Table) of these isolates being from cc ST-41/44. Note, 23/286 Msf SV-6 isolates have not been classified into any cc. This G/D substitution was also observed in 3/30 isolates expressing Msf SV-14. In addition, we identified that all isolates (with 96% of these isolates being from ST-198) encoding Msf SV-4 to have a glycine to serine mutation at the same position seen for the G/D (Fig 1). The consequence of this G/S replacement seen in the β-barrel domain of Msf SV-4 is not currently known but potentially one could predict this mutation to affect trimerisation, as seen with the G/D replacement [43].

Autotransporter adhesins are transported via the Sec translocation pathway prior to insertion into the outer membrane, a process achieved by the presence of a signal peptide at the N-terminus of the pre-protein. Unlike other N-terminal signal peptides, some autotransporters have unusually long signal peptides, which exhibit a conserved tripartite structure consisting of an *n* (N-terminal extension), *h* (containing hydrophobic residues) and *c* (containing the signal SP cleavage site). These long signal peptide sequences in some cases exceeds 50 amino acid residues, and are known as extended signal peptide sequences (ESPR), which is thought to play a post-translational role, in regulation of protein export [44–46].

**Fig 3:**
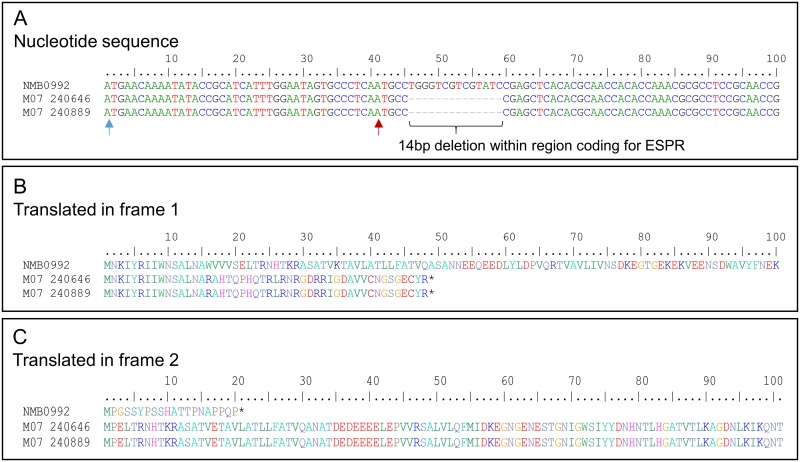
A large proportion of isolates encoding Msf-SV-8 have a 14 bp deletion within the ESPR taking the protein out of frame. A) *msf* nucleotide sequence from *N*. *meningitidis* MC58 (NMB0992 –Msf SV-1) and serogroup B isolates M07 2040646 and M07 240889 (Msf SV-8). Blue arrow—ATG start giving Msf ESPR signal peptide (translated in frame 1 –see B). Red arrow—possible alternate ATG start (translated in frame 2 –see C). Sequence alignment showed the majority of isolates with Msf SV-8 to have a 14 bp deletion within the signal peptide (A), resulting in a premature stop codon/protein reading out of frame (B). C) If the second ATG (red arrow) is used a full length Msf protein could be produced, but with a much shorter signal peptide. Predicted Vn binding domain shown above (in B and C). Asterisk (*) indicates premature stop codon.

Msf, like many other TAA’s possesses an extended signal peptide sequence (ESPR). Our analysis revealed 92% (400/434) of isolates encoding Msf SV-8, and those with Msf SV-13, have a 14 bp deletion within the nucleotide sequence coding for the ESPR (see Fig 3), resulting in a premature stop codon not seen any the other Msf sequence variants. Notably if the sequences were translated in frame 2, utilising an alternate start codon, those same sequences would code for a full length Msf with a ‘shorter’ signal peptide (predicted SP starting MPEL), confirmed using SignalP 4.1 online software.

### Msf SV-5 displays a significant reduction in vitronectin binding when compared to Msf SV-1 and Msf SV-2

**Fig 4:**
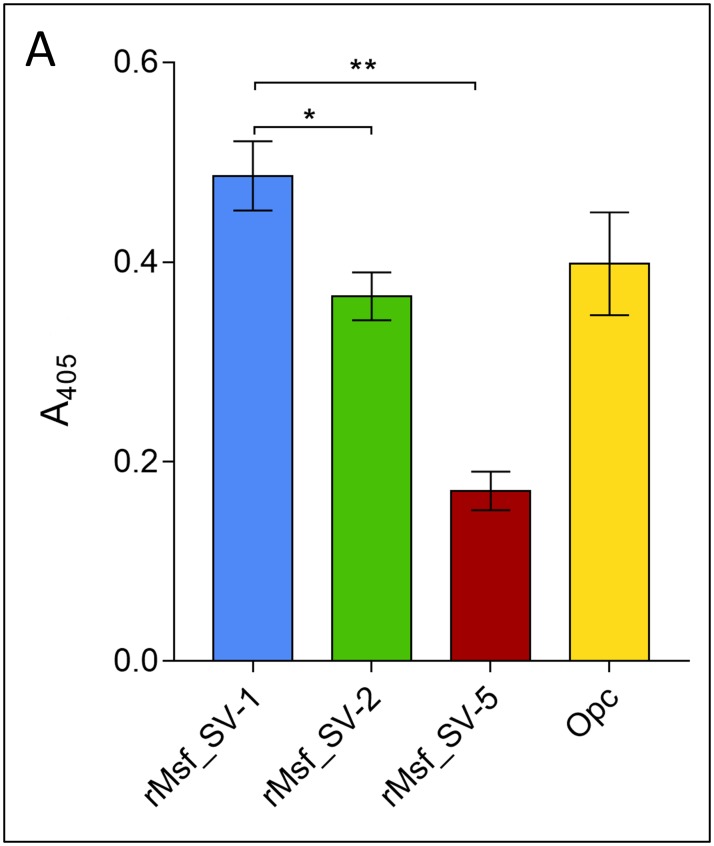
Msf recombinant proteins of the three predominant Msf SVs associated with hyperinvasive isolates exhibit differential binding to vitronectin. ELISA Immulon 2HB plates were coated overnight with 3 μM of either Msf recombinant proteins (rMsf_SV-1_49-189,_ blue; rMsf_SV-2_49-186_, green; or rMsf_SV-5_49-189_, red) or purified Opc (yellow) protein, prior to blocking with 3% BSA. The plates were subsequently overlaid with 0.2 μg vitronectin and vitronectin binding was detected using anti-Vn monoclonal antibody 8E6 and anti-mouse alkaline phosphatase IgG (secondary antibody). SIGMA*FAST*^*™*^ p-Nitrophenyl phosphate tablets were used to detect alkaline phosphatase activity in the ELISA and the absorbance was measured at 405 nm. Results are the average of three independent experiments; p-values (≤ 0.01) indicated with * or **. Error bars show standard error between replicates.

The predicted Vn binding domain of Msf contains several positively charged residues (e.g. lysine) thought to exhibit charge complementarity with the negatively charged vitronectin peptide. Given that Msf SV-3 to SV-20 have an increased number of non-charged polar residues (NTN, Table 2), we hypothesised that the Msf SVs would display differences in Vn binding due to a change in charge. To test this hypothesis, recombinant proteins (rMsfSV-1_49-189_, rMsfSV5_49-189,_ and rMsfSV2_49-186_) were assayed for Vn binding by ELISA. As predicted rMsfSV5_49-189_ (which has the motif NTNENTNASS between the conserved TLKAGDNLKIKQ and FTYSLKK motifs) showed a significant reduction in Vn binding when compared with rMsfSV-1_49-189_, rMsfSV2_49-186_ and Opc (Fig 4).

**Fig 5:**
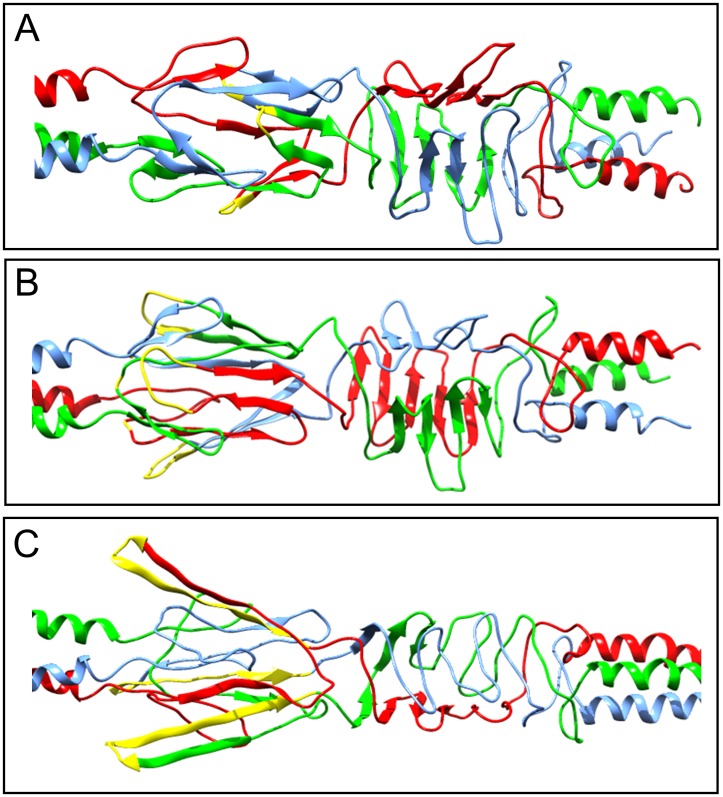
Addition of repeating NTN motifs between the conserved predicted Vn binding motifs causes a looping out of the structure. Ribbon structure and molecular model of the N-terminal Vn binding domain of MC58 Msf (SV-1; NGTN motif) (A), 10 amino acid loop variant (e.g. found in Msf SV-5, SV-6 and SV-7) (B), and a 14 amino acid loop variant (e.g. found in Msf SV-8, SV-9, SV-10, and SV-12) (C). The MC58 Msf molecular (wild-type) model was used to predict structural differences of the addition of a repeating NTN motif between the conserved TLKAGDNLKIKQ and FTYSLKK motifs (B and C), seen for the 10 and 14 residue loop variants. Each chain of the Msf trimer is differently coloured (blue, red or green) with the location of the NGTN and loop variant motifs shown in yellow.

The difference in Vn binding affinity displayed by our Msf recombinant proteins may be due to structural differences in the previously characterised Vn binding domain located within the N-terminus of Msf (Table 3, and [32]). To further demonstrate this, we have modelled both a 10 amino acid loop (for Msf SV-5 to SV-7) and 14 amino acid loop variant (for Msf SV 8 to 12) into our current N-terminal domain model of Msf [32] (Fig 5). Our models indicated that increasing the number of NTN motifs between the conserved motifs (TLKAGDNLKIKQ and FTYSLKK) can only be accommodated in the structure by ‘looping out’ within the predicted Vn binding domain (Fig 5B and 5C). Further functional and structural analyses are required to validate our molecular models and provide further clues as to the driving force for the evolution of such diversity in Msf N-terminal domain.

### Antisera generated against recombinant proteins of three Msf sequence variants capable of recognising at least 11 out of 20 Msf SVs

Antisera against full length Msf (TA38), or residues 1–203 (anti-MsfA) from MC58 (15, 32), revealed a lack of cross reactivity with other Msf variants. Because of the lack of cross reactivity with rMsfSV-5_49-189,_ rMsfSV-2_49-186_, immunisations were performed using all three recombinants, generating polyclonal antisera referred to as anti-MsfSV1/2/5. Our anti-MsfSV1/2/5 antibody was able to detected four N-terminal recombinant proteins (rMsfSV-1_49-189_, rMsfSV-2_49-186_, rMsfSV-5_49-189,_ and rMsfSV-8_31-178_), and 11/20 Msf SV expressed by meningococcal clinical isolates (Table 4 and S2 Fig).

**Table 4:** Antiserum generated against Msf SV-1, SV-2, and SV-5 is cross reactive.

Isolates	Serogroup	Msf-SV ^a^	Western blot result ^d^	% of isolates with Msf SV ^e^
*N-terminal recombinant proteins*				
rMsfSV-1_49-189_ ^b^	-	SV-1	++	18.4%
rMsfSV-2_49-189_ ^b^	-	SV-2	++	33.8%
rMsfSV-5_49-186_ ^b^	-	SV-5	++	10.4%
rMsfSV-8_31-178_ ^b^	-	SV-8	++	6.6%
*N*. *meningitidis clinical isolates*				
MC58 ^c^	B	SV-1	++	18.4%
PMC3 ^c^	A	SV-5	+	10.4%
PMC8 ^c^	C	SV-2	+	33.8%
PMC9	C	SV-11	+++	10.8%
PMC14	C	SV-14	+++	0.5%
M07 240646 ^c^	B	SV-8	-	6.6%
M07 240789	B	SV-9	+	0.21%
M07 240680	B	SV-10	+	3.4%
M07 240909	B	SV-12	++	8%
M07 240669	B	SV-6	++	4.5%
M07 240949	B	SV-3	+	0.2%

^a^ Isolate Msf SV determined by PCR and sequencing, later confirmed for some of the meningococcal isolates by genome sequencing

^b^ N-terminal Msf recombinant proteins, rMsfSV-1_49-189_, rMsfSV-2_49-186_, rMsfSV-5_49−189_, and rMsfSV-831-178, were expressed in *E*. *coli* and purified as previously described (see Methods).

^c^ Strains used for cloning and expression of N-terminal domain of different Msf-SVs as recombinant proteins. Lack of *msf* expression seen for M07 240646 is likely due to the presence of a 14 bp deletion within the coding region for the signal peptide, resulting in a premature stop codon.

^d^ Western blot analysis indicating relative level of expression (- = no detectable expression; + = low levels of expression detected; ++ = good expression level; +++ = high levels of expression). See S2 Fig for raw data.

^e^ Prevalence of meningococcal isolates with Msf-SV from Neisseria PubMLST analysis on 6,500 meningococcal isolates.

### The putative Msf vitronectin binding motif is found in more than one location within *Haemophilus influenzae* Hsf and Hia

**Fig 6:**
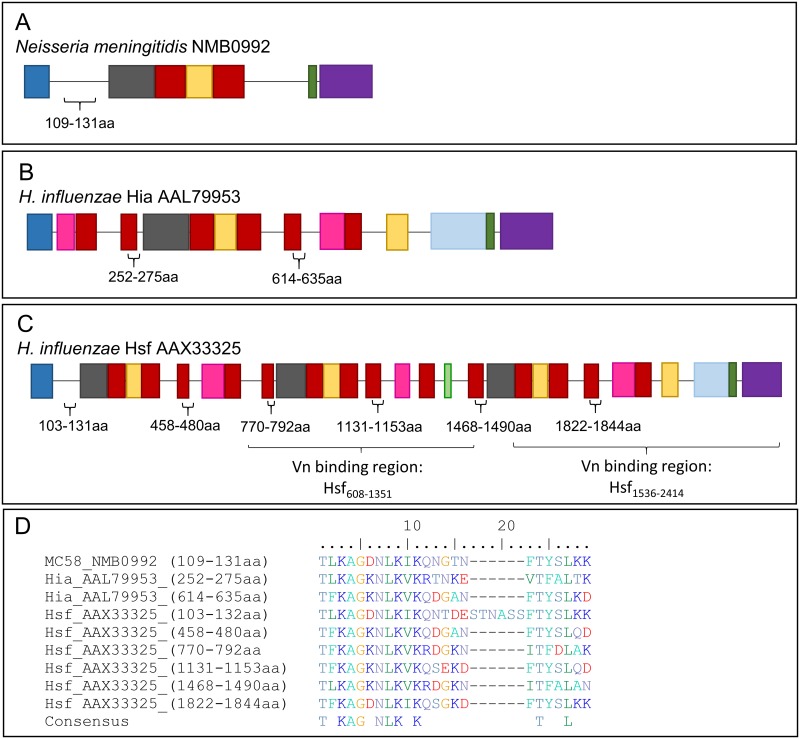
Domain annotation and location of repeat regions of homology seen between *N*. *meningitidis* Msf and *H*. *influenzae* Hia and Hsf. The architecture of the trimeric autotransporter adhesin *N*. *meningitidis* Msf (A), and Hia AAL79953 (B) and Hsf AAX33325 (C) from *H*. *influenzae* was predicted using dTAA online software. dTAA domain annotations; blue–signal peptide; red—Trp ring; grey—GANG; yellow—KG; green—neck; pink—connector (ISneck); light blue—TTT; light green—YadA like head. Approximate positions of sequences aligning to the Msf vitronectin binding domain (VnBD) (see sequences in D) indicated in brackets. Previously identified VnBD for Msf (amino acid resides 90–133, (32)) and Hsf (amino acid residues 608–1351 and 1536–2414; [48]) shown under each respective diagram. Diagram is not drawn to scale. N and C-terminus of each protein sequence are indicated. D) Sequence alignment of ‘repeat’ regions seen in Hia and Hsf, with two repeat regions seen for Hia, and six for Hsf. Consensus sequence shows residues conserved between all sequences within the alignment.

*N*. *meningitidis* Msf displays homology to Hsf and Hia from *Haemophilus influenzae*, which play roles in vitronectin binding, serum resistance and adhesion [27, 47, 48]. Due to the sequence conservation seen between all three TAAs and the repetitive nature of Hia and Hsf, we decided to examine whether the Msf vitronectin binding motif was present in one or more locations within both *H*. *influenzae* proteins. *N*. *meningitidis* Msf (NMB0992) amino acid residues 1–203 was used for BLAST analysis against Hsf (AAX33325) and Hia (AAL79953), with a 60% or 56% identity seen for each respective protein. Interestingly, both Hia and Hsf showed more than one or more homologous regions (two for Hia; six for Hsf) to the predicted Msf vitronectin binding domain (see Fig 6B–6D for amino acid residue locations), with several conserved residues seen in between each ‘repeat region’ of Hia, Hsf and Msf. In addition, three of the ‘repeat’ motifs seen in Hsf (Fig 6D) are located within the vitronectin binding domain of Hsf; two in Hsf_608-1351_ and one in Hsf_1536-2414_ (Fig 6C and 6D) [48].

## Discussion

*N*. *meningitidis* is a human restricted pathogen that exhibits a high degree of variation, a necessity believed to have evolved to ensure its maintenance of a carriage state, evasion of the host immune response. However, this variation is can contribute to invasion of host cells and survival in the blood leading to sepsis and infection of the meninges. The diversiy observed in *N*. *meningitidis* outer membrane proteins poses a significant problem when designing an effective universal meningococcal vaccine. Hence, although a number of vaccines have been developed, none provide protection against all meningococcal isolates [2, 3], with Bexsero predicted to cover 66 to 76% of circulating serogroup B isolates [6, 7]. Universal meningococcal vaccine coverage is further complicated by the global distribution of different clonal complexes and by the occurrence of capsule switching [7, 49].

*N*. *meningitidis* outer-membrane proteins are known to exhibit variable expression levels, with a 15-fold difference in expression seen for fHbp [50]. Although the expression of Msf is known to vary between strains of *N*. *meningitidis* [25], Msf has been shown to be upregulated in response to epithelial attachment [30] and changes in iron presentation [33].

Diversity of meningococcal outer-membrane proteins is known to result in the lack of generation of cross reactive antibodies, making production of a universal vaccine difficult. To highlight this difficulty, antibodies raised against NadA variant 4 do not recognise any other NadA variants, and although bactericidal activity is seen against NadA4, only very low levels of bactericidal killing seen against meningococci expressing alternate variants [51].

As *msf* is present in all meningococcal isolates sequenced to date, is expressed *in vivo*, elicits bactericidal antibodies and provides serum resistance, it is regarded, along with *opc*, as a potential candidate for inclusion in a universal vaccine [15, 21, 25, 26, 32]. In addition to our previous studies, we have shown that antibodies generated against the Vn binding domains of Msf SV-1, SV-2 and SV-5 (anti-MsfSV1/2/5) and a synthetic peptide of Opc loop 2 (anti-Opcloop2-KLH), display serum bactericidal and function blocking activity (see Supplementary information and S1 Fig). In addition, we have shown our anti-MsfSV1/2/5 antibody to be cross reactive, recognising 11/20 Msf sequence variants. Both Msf and Opc exhibit variable expression levels, with differences in *opc* expression being due to phase variation [12, 30, 52]. Previously, simultaneous expression of Msf and Opc has been shown to impart an increase in serum resistance and vitronectin binding, when compared to expression of either Msf or Opc alone [15]. Our analysis has revealed that the absence of *opc* for the most part is correlated with the presence of Msf SV-2, with the majority of isolates with this phenotype being from cc ST-11.

Although *N*. *meningitidis* outer membrane proteins, such fHbp and NadA, are known to display a high degree of antigenic variation, distinct associations with clonal complexes and sequence variants have been observed [53–55]. FHbp, found in all meningococcal isolates, is required for modulation of the immune response by binding to human factor H, a negative regulator of the alternate complement pathway. PorA, FetA, NadA and fHbp sequence variants, exhibit non-random, non-overlapping association with distinct clonal complexes [35, 40, 53–57]. This highly structured diversity of antigenic variants is thought to have evolved through strong immune selection leading to stable dominant selection of alleles at different antigenic loci [35, 40]. Interestingly, our analysis revealed there to be a similar association with distinct Msf SVs and certain serogroups and clonal complexes (Fig 2). For example, isolates from cc ST-11, previously associated with fHbp peptide 22 and NadA variant 29 [54, 55], predominantly have Msf SV-2 but lack *opc*. In comparison isolates from ST-1, ST-4 and ST-5, responsible for meningococcal epidemics/pandemics in sub-Saharan Africa [40, 58], are associated with fHbp variant 1 [55], and predominantly have Msf SV-5 and are Opc positive (S2 Table).

This association between outer membrane protein variant type and clonal complex is thought to be due to the high degree of genetic stability exhibited by meningococci [53]. For example, immunogenic antigens in serogroup A isolates associated with cc ST-5 have been shown to be highly conserved, suggesting evasion of the immune response is not linked to emergence of epidemic waves in sub-Saharan Africa meningitis belt [40]. However, while genes encoding immunogenic antigens (including fHbp, NadA and Opc) are largely conserved, allelic differences in genes required for metabolic functions and environmental and genetic information processing have been seen between different pandemic waves of cc ST-5 [40]. Indeed, associations between clonal complexes and protein variant types of Msf (this study), as well as fHbp and NadA have been observed [57, 59]. For example, ST-32 isolates tend to have NadA1 and fHbp sub-variant 1.1 and are commonly associated with Msf SV-1, whereas NadA2 and NadA3 being more commonly found with cc ST-11 (which predominantly has Msf SV-2; S2 Table) and ST-8 (predominantly Msf SV-10; S2 Table) [59]. Our analysis has shown a clear distinction in Msf SV type with isolates associated with either invasive disease or carriage. This is illustrated in the case of Msf SV-1, SV-2 and SV-5 which are largely associated with invasive isolates (e.g. cc ST-8, ST-11, ST-269 and ST-41/44) and Msf SV-11 and SV-12 being associated with cc ST-23, ST-35, ST-60 and ST-22 which are commonly associated with carriage (Fig 2, S2 and S3 Tables). Whilst isolates used in this study represented 58 different countries, it should be noted that the majority of isolates from the PubMLST BIGSdb are from the UK hence may not truly represent the global distribution of Msf and Opc. Further information on the isolates used in this study is provided in S4 Table to facilitate further analyses.

Our analysis has indicated that three variant types (Msf SV-6, Msf SV-8 and Msf SV-13) found in ~11% of meningococcal isolates in our database, may not be efficiently expressed/exported to the outer-membrane, due to either a mutation within the β-barrel or a 14 bp deletion within the sequence encoding the ESPR (Table 3, Figs 2 and 3). Considering all other Msf SV have the full length 51 aa signal peptide, ESPR are required for proper translocation of TAA’s to the outer-membrane. No detectable *in vitro* expression of clinical isolates encoding Msf SV-8 was observed and, as anti-MsfSV1/2/5 is able to detect rMsfSV-8_31−178_, one can predict there to have been an immune selective pressure for this 14 bp deletion (S2 Fig). It is important to note that out of the 434 isolates that encode either a predicted Msf SV-8 or SV-13 only 11 lack Opc, and the clear majority of isolates are from clonal complex ST-269 (79.95%; CI = 75.932–83.45%—S3 Table), suggesting a selection advantage in expression of at least one vitronectin binding protein. In addition, those isolates encoding Msf SV-6, largely associated with cc ST-41/44, are also Opc positive. The predicted lack of expression of this Msf sequence variant may be compensated for by Opc, which performs similar functions as Msf e.g. in terms of vitronectin binding and adherence to host cells.

Our analysis has shown a difference in Msf vitronectin binding capabilities exhibited by Msf SV-1, SV-2 and SV-5, with the presence of repeating NTN motifs causing a predicted structural alternation resulting in a reduction in Vn binding, as seen with rMsfSV-5_49-186_ (Figs 4 and 5). We propose that the Msf sequence variants (described in Table 2) have evolved as consequence of immune selection pressure, balanced with selection to maintain function, in this case binding to vitronectin, and hence is linked to the evolution of clonal complexes. Our results indicate that an increased number of repeating NTN motifs result in the reduction/loss of vitronectin binding capability and ‘looping out’ of the structure, compensated for by the presence of Opc which performs similar functions. This ‘looping out’ of the N-terminal domain has precedent, and has been observed in N-terminal structure of the trimeric autotransporter adhesin NadA [56]. There are, however, exceptions to this hypothesis as isolates found in cc ST-213 and cc ST-8 lack *opc* and encode Msf sequence variants predicted not to bind to Vn as well as Msf SV-1 and SV-2. For example, 89% of isolates from cc ST-213 encode Msf SV-12, and 70% of cc ST-8 isolates encode Msf SV-10, which, due to the presence of multiple NTN motifs within the Vn binding domain and predicted structural changes, may display a significant reduction in vitronectin binding affinity (Table 2, Figs 4 and 5). It is also plausible that ST-8 and 213 complex isolates may have further adaptations allowing immune evasion. Further functional and structural work into the Msf sequence is required to validate these predictions. It may be possible that the NTN repeat motifs provide an advantage to meningococci. For example, NTN repeats and the NTNENTNENTNASS sequence (seen in Msf SV-11) are also found within asparagine-rich antigen (locus SBT70246) from *Plasmodium malariae*. *Plasmodium* asparagine-rich proteins have been shown to regulate sporozoite gene expression and liver stage development, and are required for induction of a protective immune response [60].

*N*. *meningitidis* Msf displays homology to Hsf and Hia from *H*. *influenzae*. In comparison to Msf, Hia and Hsf are much larger and are known to have repetitive architecture (protein monomers; Msf ~62 kDa, Hia ~114 kDa and Hsf ~250 kDa). Hia has been shown to display repetitive architecture, containing two binding pockets which display varying affinities for epithelial cell attachment [61, 62]. Hsf is conserved among all typeable *H*. *influenzae* isolates and, like *N*. *meningitidis* Msf, binds Vn and is required for survival within human serum [48]. *H*. *influenzae* Hsf forms a unique twisted hair-pin like structure, with the N-terminal domain located near the C-terminus at the base of the trimeric fibril structure [63], having two distinct vitronectin binding domains located within Hsf_608-1351_ and Hsf_1536-2414_ [48] (Fig 6C). Although two vitronectin binding domains were identified [48], an alternate study identified Hsf binding domain 2 (BD2), located at the N-terminus, to serve as the major vitronectin binding domain of Hsf (64). Hsf AAX33325 Vn BD1 and BD2 (Fig 6C and 6D) BD2 [64], contains the three motifs showing homology to the putative Msf Vn binding domain. Comparison of the putative Msf vitronectin binding domain with Hia and Hsf revealed the conservation of a T(n)KAG(n)K(n)K motif (consensus sequence in Fig 6D) in the putative vitronectin binding domain both Msf and Hsf, with Hsf having these motifs at both the N and C-terminus. Indeed, lysine resides K66 and K68 of the mature Msf protein have been implicated in vitronectin binding, with a KIK66-68-AIA mutation causing a reduced Vn binding affinity [32]. These two lysines form part of the consensus sequence seen in Fig 6D, indicating that these residues could also be required for Vn binding in both *N*. *meningitidis* Msf and *H*. *influenzae* Hsf.

## Conclusions

This study has highlighted the importance of using large scale bioinformatic datasets to gain a greater understanding of the variability exhibited by meningococcal outer-membrane adhesins. Analysis of over 6,000 meningococcal isolates has revealed there to be 20 distinct Msf sequence variants, defined by our group as having increasing number of amino acid residues between two highly conserved motifs previously associated with Vn binding (32). We have shown the Msf sequence variants to display correlations with distinct serogroups, clonal complexes and presence/absence of *opc*, shown three recombinant Msf proteins to display differences in vitronectin binding affinity, and have generated a cross reactive antibody capable of recognising at least 11/20 Msf sequence variants, which displays both serum bactericidal and function blocking activity.

We envisage that use of Msf and Opc as vaccine antigens could potentially provide much broader vaccine coverage. Indeed, our bioinformatic analysis has indicated that if both Msf and/or Opc are expressed *in vivo*, and antibodies generated against both the vitronectin binding domains of Msf SV-1, SV-2 and SV-5 and Opc are bactericidal, a vaccine containing these proteins could provide protection against 98% of meningococcal isolates. Further work however is required to validate this prediction. We believe using large scale *in silico* analysis to determine the variability exhibited by putative meningococcal vaccine candidates is a key step in the creation of an effective and universal vaccine to prevent meningococcal disease.

## Supporting information

S1 Table Prevalence of Opc within in those clonal complexes positively associated with invasive disease or carriage. (PDF)

S2 Table Prevalence of each Msf sequence variant in those clonal complexes positively associated with invasive disease or carriage. (PDF)

S3 Table Prevalence of the most common Msf sequence variants within all meningococcal clonal complexes within our database. (PDF)

S4 Table Details of meningococcal isolates used in this study. (PDF)

S1 Fig Antibodies against the functional domains of Msf or Opc are serum bactericidal and function blocking. (PDF)

S2 Fig Msf exhibits varying expression levels in different meningococcal clinical isolates. (PDF)
