# Supplementary material for: Bioinformatic analysis of meningococcal Msf and Opc to inform vaccine antigen design
Source: PLoS One. 2018 Mar 16;13(3):e0193940. doi: 10.1371/journal.pone.0193940 (PMC5856348; doi:10.1371/journal.pone.0193940)
Supplement: S1 Table — (PDF) [file pone.0193940.s001.pdf]

Table S1 - Prevalence of Opc within in those clonal complexes positively associated with invasive disease or carriage

Clonal complex	Opc presence/absence	
	Positive[a]	Negative[b]
**Hyperinvasive lineages**		
ST-1	98.44 (91.67 - 99.92)	4.45 (0.08 - 8.33)
ST-4	100 (85.13 - 100)	0 (0 - 14.87)
ST-5	100 (97.4 - 100)	0 (0 - 2.59)
ST-8	0 (0 - 11.35)	**100 (88.65 - 100)**
ST-11	0.657 (0.003 - 0.37)	**99.92 (99.63 - 99.99)**
ST-32	100 (99.08 - 100)	0 (0 - 0.919)
ST-41/44	96.54 (95.21 - 97.52)	3.46 (2.48 - 4.79)
ST-269	100 (99.4 - 100)	0 (0 - 0.57)
**Carriage**		
ST-23	100 (99.28 - 100)	0 (0 - 0.72)
ST-35	100 (95.47 - 100)	0 (0 - 4.52)
ST-60	100 (96.65 - 100)	0 (0 - 3.34)

[a] Meningococcal isolates opc displaying over 97.7% sequence identity to NMB1053 are classed as Opc positive. [b] Those isolates with an either lacking or having a truncated opc (exhibiting 28.85 to 45.7% identity to NMB1053) were annotated as Opc negative isolates.

Porportion of isolates from each clonal complex Opc postive/negative expressed as a mean percentage, displaying lower and upper confidence intervals (CI). Analysis performed using Wilson/Brown method, CI to 95%.
