# Supplementary material for: Bioinformatic analysis of meningococcal Msf and Opc to inform vaccine antigen design
Source: PLoS One. 2018 Mar 16;13(3):e0193940. doi: 10.1371/journal.pone.0193940 (PMC5856348; doi:10.1371/journal.pone.0193940)
Supplement: S2 Table — (PDF) [file pone.0193940.s002.pdf]

Table S2 - Prevalence of each Msf sequence variant in those clonal complexes positively associated with invasive disease or carriage

	Msf sequence variant																			
Clonal complex	SV-1	SV-2	SV-3	SV-4	SV-5	SV-6	SV-7	SV-8	SV-9	SV-10	SV-11	SV-12	SV-13	SV-14	SV-15	SV-16	SV-17	SV-18	SV-19	SV-20
**Hyperinvasive lineages**																				
ST-1	*[a]	1.563 (0.08 - 8.33)	*[a]	*[a]	**98.44 (91.67 - 99.91)**	*[a]	*[a]	*[a]	*[a]	*[a]	*[a]	*[a]	*[a]	*[a]	*[a]	*[a]	*[a]	*[a]	*[a]	*[a]
ST-4	*[b]	*[b]	*[b]	*[b]	**100 (85.135 - 100)**	*[b]	*[b]	*[b]	*[b]	*[b]	*[b]	*[b]	*[b]	*[b]	*[b]	*[b]	*[b]	*[b]	*[b]	*[b]
ST-5	*[c]	*[c]	*[c]	*[c]	**100 (97.4 - 100)**	*[c]	*[c]	*[c]	*[c]	*[c]	*[c]	*[c]	*[c]	*[c]	*[c]	*[c]	*[c]	*[c]	*[c]	*[c]
ST-8	*[d]	*[d]	*[d]	*[d]	*[d]	*[d]	*[d]	*[d]	*[d]	**70 (52.124 - 83.34)**	*[d]	*[d]	*[d]	**30 (16.66 - 47.876)**	*[d]	*[d]	*[d]	*[d]	*[d]	*[d]
ST-11	0.066 (0.003 - 0.372)	**99.47 (98.96 - 99.7)**	*[e]	*[e]	0.066 (0.003 - 0.372)	*[e]	*[e]	0.066 (0.003 - 0.372)	*[e]	0.132 (0.023 - 0.478)	*[e]	0.197 (0.053 - 0.579)	*[e]	*[e]	*[e]	*[e]	*[e]	*[e]	*[e]	*[e]
ST-32	**97.1 (95 - 98.33)**	0.725 (0.197 - 2.11)	1.93 (0.982 - 3.76)	*[f]	0.24 (0.012 - 1.355)	*[f]	*[f]	*[f]	*[f]	*[f]	*[f]	*[f]	*[f]	*[f]	*[f]	*[f]	*[f]	*[f]	*[f]	*[f]
ST-41/44	1.423 (0.849 - 2.373)	**50.71 (47.59 - 53.83)**	0.102 (0.005 - 0.573)	*[g]	4.268 (3.17 - 5.72)	**25.91 (23.27 - 28.74)**	1.02 (0.55 - 1.86)	0.102 (0.005 - 0.573)	*[g]	**14.84 (12.75 - 17.195)**	0.41 (0.158 - 1.04)	0.305 (0.083 - 0.892)	*[g]	0.813 (0.413 - 1.596)	0.102 (0.005 - 0.573)	*[g]	*[g]	*[g]	*[g]	*[g]
ST-269	**45.67 (41.94 - 49.46)**	0.45 (0.122 - 1.308)	*[h]	*[h]	0.149 (0.007 - 0.84)	*[h]	0.45 (0.122 - 1.308)	**51.79 (48.01 - 55.55)**	*[h]	*[h]	0.75 (0.319 - 1.734)	0.15 (0.007 - 0.84)	0.45 (0.122 - 1.308)	*[h]	*[h]	*[h]	0.15 (0.007 - 0.84)	*[h]	*[h]	*[h]
**Carriage**																				
ST-23	0.374 (0.066 - 1.353)	0.187 (0.009 -1.05)	*[i]	*[i]	0.561 (0.153 - 1.64)	*[i]	0.374 (0.066 - 1.353)	0.187 (0.009 - 1.05)	*[i]	0.374 (0.066 - 1.353)	**95.14 (92.98 - 96.66)**	0.374 (0.066 - 1.353)	0.187 (0.009 - 1.05)	0.935 (0.399 - 2.169)	1.495 (0.759 - 2.923)	*[i]	*[i]	*[i]	*[i]	*[i]
ST-35	2.47 (0.44 - 8.56)	4.94 (1.936 - 12.02)	*[j]	*[j]	2.47 (0.44 - 8.56)	*[j]	1.235 (0.063 - 6.667)	*[j]	*[j]	*[j]	*[j]	**71.6 (60.98 - 80.27)**	3.7 (1.01 - 10.332)	1.235 (0.063 - 6.667)	*[j]	**13.58 (7.756 - 22.703)**	2.47 (0.44 - 8.56)	*[j]	*[j]	*[j]
ST-60	0.901 (0.46 - 4.93)	2.7 (0.736 - 7.646)	*[k]	*[k]	4.5 (1.939 - 10.113)	0.901 (0.46 - 4.93)	*[k]	0.901 (0.46 - 4.93)	*[k]	0.901 (0.46 - 4.93)	**85.59 (77.86 - 90.927)**	2.7 (0.736 - 7.646)	*[k]	0.901 (0.46 - 4.93)	*[k]	*[k]	*[k]	*[k]	*[k]	*[k]

Table shows prevalence of each Msf SV within each clonal complex. Expressed as a percentage, showing the mean and the lower and upper confidence intervals (95% CI) in brackets. The most common SV within each clonal complex is highlighted in bold. Analysis performed using Wilson/Brown method to a 95% CI.

*a = 0 (0 - 5.66);*b = 0 (0 - 14.865); *c = 0 (0 - 2.59); *d = 0 (0 - 11.35); *e = 0 (0 - 0.252); *f = 0 (0 - 0.919); *g = 0 (0 - 0.388); *h = 0 (0 - 0.57); *i = 0 (0 - 0.712); *j = 0 (0 - 4.53); *k = 0 (0 - 3.345)
