# Supplementary material for: Bioinformatic analysis of meningococcal Msf and Opc to inform vaccine antigen design
Source: PLoS One. 2018 Mar 16;13(3):e0193940. doi: 10.1371/journal.pone.0193940 (PMC5856348; doi:10.1371/journal.pone.0193940)
Supplement: S3 Table — (PDF) [file pone.0193940.s003.pdf]

Table S3 - Prevalence of the most common Msf sequence variants within all meningococcal clonal complexes within our database.

Clonal Complexes

Msf sequence variants	ST-1 complex	ST-103 complex	ST-106 complex	ST-11 complex	ST-1117 complex	ST-1136 complex	ST-1157 complex	ST-116 complex	ST-162 complex	ST-167 complex	ST-174 complex
SV-1	*a	0.083 (0.004 - 0.469)	0.748 (0.394 - 0.748)	0.083 (0.004 - 0.469)	*a	*a	5.4 (4.26 - 6.82)	*a	7.15 (5.82 - 8.75)	*a	4.07 (3.09 - 5.344)
SV-2	0.045 (0.002 - 0.257)	*b	*b	**68.76 (66.79 - 70.66)**	*b	1.36 (0.957 - 1.94)	0.045 (0.002 - 0.257)	*b	*b	*b	*b
SV-5	9.21 (7.26 - 11.61)	5.85 (4.32 - 7.86)	*c	0.146 (0.007 - 0.823)	*c	*c	*c	*c	*c	8.92 (7 - 11.289)	*c
SV-6	*d	*d	*d	*d	*d	*d	*d	*d	0.699 (0.124 - 2.513)	*d	*d
SV-8	*e	*e	*e	0.23 (0.012 - 1.29)	*e	*e	*e	*e	*e	*e	*e
SV-10	*f	*f	*f	0.897 (0.159 - 3.21)	1.79 (0.699 - 4.52)	*f	*f	*f	*f	*f	*f
SV-11	*g	0.28 (0.05 - 1.029)	*g	*g	*g	*g	0.142 (0.007 - 0.8)	*g	*g	*g	*g
SV-12	*h	*h	0.388 (0.069 - 1.404)	0.58 (0.158 - 1.698)	*h	*h	*h	1.359 (0.659 - 2.78)	*h	*h	*h

Msf sequence variants	ST-175 complex	ST-178 complex	ST-18 complex	ST-181 complex	ST-198 complex	ST-212 complex	ST-213 complex	ST-22 complex	ST-226 complex	ST-23 complex	ST-231 complex
SV-1	*a	0.333 (0.129 - 0.852)	0.083 (0.004 - 0.469)	*a	*a	*a	0.582 (0.282 - 1.196)	0.083 (0.004 - 0.469)	*a	0.166 (0.029 - 0.604)	*a
SV-2	0.045 (0.002 - 0.257)	*b	*b	*b	*b	*b	0.364 (0.184 - 0.716)	0.045 (0.002 - 0.257)	*b	0.045 (0.002 - 0.257)	*b
SV-5	1.169 (0.59 - 2.29)	*c	0.73 (0.312 - 1.699)	2.632 (1.67 - 4.12)	*c	*c	0.146 (0.007 - 0.823)	*c	*c	0.4389 (0.12 -1.28)	*c
SV-6	*d	*d	*d	*d	*d	*d	1.049 (0.285 - 3.038)	*d	*d	*d	*d
SV-8	*e	*e	*e	*e	*e	*e	*e	*e	*e	0.23 (0.012 - 1.29)	*e
SV-10	*f	*f	*f	*f	*f	*f	0.448 (0.02 - 1.652)	*f	0.448 (0.02 - 1.652)	0.897 (0.159 - 3.21)	0.897 (0.159 - 3.21)
SV-11	*g	*g	*g	*g	*g	1.278 (0.674 - 2.412)	0.71 (0.303 - 1.652)	0.43 (0.116 - 1.245)	*g	**72.19 (68.78 - 75.37)**	*g
SV-12	*h	*h	*h	*h	0.194 (0.009 - 1.09)	*h	**48.35 (44.06 - 52.66)**	**24.9 (21.2 - 28.79)**	*h	0.388 (0.069 - 1.405)	*h

Msf sequence variants	ST-254 complex	ST-269 complex	ST-282 complex	ST-292 complex	ST-32 complex	ST-334 complex	ST-35 complex	ST-364 complex	ST-37 complex	ST-4 complex	ST-41/44 complex
SV-1	1.247 (0.757 - 2.05)	**25.44 (23.05 - 27.97)**	*a	*a	**33.42 (30.81 - 36.13)**	0.166 (0.029 - 0.604)	0.166 (0.029 - 0.604)	0.333 (0.129 - 0.852)	*a	*a	1.16 (0.69 - 1.943)
SV-2	*b	0.14 (0.04 - 0.4)	*b	*b	0.14 (0.04 - 0.4)	*b	0.182 (0.07 - 0.467)	*b	*b	*b	**22.69 (20.99 - 24.489)**
SV-5	*c	0.146 (0.007 - 0.823)	*c	*c	0.146 (0.007 - 0.823)	*c	0.29 (0.05 - 1.06)	*c	*c	3.22 (2.133 - 4.821)	6.14 (4.57 - 8.196))
SV-6	*d	*d	*d	*d	*d	*d	*d	*d	*d	*d	**89.16 (85.025 - 92.258)**
SV-8	*e	**79.82 (75.796 - 83.32)**	*e	*e	*e	*e	*e	*e	*e	*e	0.23 (0.012 - 1.29)
SV-10	*f	*f	*f	*f	*f	*f	*f	*f	*f	*f	**65.47 (59.016 - 71.4)**
SV-11	*g	0.71 (0.303 - 1.652)	*g	*g	*g	0.284 (0.05 - 1.029)	*g	*g	*g	*g	0.568 (0.22 - 1.45)
SV-12	*h	0.194 (0.009 - 1.09)	*h	0.194 (0.009 - 1.09)	*h	*h	**11.1 (8.69 - 14.1)**	*h	*h	*h	0.58 (0.158 - 1.698)

Msf sequence variants	ST-4240/6688 complex	ST-461 complex	ST-4821 complex	ST-5 complex	ST-53 complex	ST-549 complex	ST-60 complex	ST-750 complex	ST-8 complex	ST-865 complex	ST-92 complex
SV-1	*a	8.15 (6.73 - 9.83)	*a	*a	*a	*a	0.083 (0.004 - 0.469)	0.166 (0.029 - 0.604)	*a	0.083 (0.004 - 0.469)	1.413 (0.88 - 2.25)
SV-2	*b	0.045 (0.002 - 0.257)	0.045 (0.002 - 0.257)	*b	2.23 (1.689 - 2.93)	*b	0.136 (0.037 - 0.4)	*b	*b	0.27 (.125 - 0.594)	*b
SV-5	0.146 (0.007 - 0.823)	1.17 (0.594 - 2.29)	*c	**21.05 (18.16 - 24.265)**	*c	0.146 (0.007 - 0.823)	0.73 (0.31 - 1.69)	0.146 (0.007 - 0.823)	*c	**22.08 (19.13 - 25.34)**	*c
SV-6	*d	0.349 (0.179 - 1.953)	*d	*d	*d	*d	0.349 (0.179 - 1.953)	*d	*d	0.349 (0.179 - 1.953)	*d
SV-8	*e	*e	*e	*e	*e	*e	0.23 (0.012 - 1.29)	*e	*e	*e	*e
SV-10	*f	*f	*f	*f	*f	*f	0.448 (0.02 - 1.652)	*f	9.42 (6.24 - 13.97)	0.448 (0.02 - 1.652)	*f
SV-11	*g	*g	*g	*g	0.284 (0.05 - 1.029)	0.142 (0.007 - 0.8)	13.47 (11.152 - 16.195)	*g	*g	*g	*g
SV-12	*h	*h	*h	*h	0.194 (0.01 - 1.09)	*h	0.58 (0.158 - 1.698)	*h	*h	*h	*h

Table shows what proportion of Msf SV occurs within all clonal complex types of *N. meningitidis* found in our dataset, most common highlighted in bold. Expressed as a percentage, showing the mean and the lower and upper confidence intervals (95% CI) in brackets. Analysis performed using Wilson/Brown method to a 95% CI. Data only for those Msf SV found in over 100 isolates. Analysis excludes any isolates not defined into a clonal complex.

*a = 0 (0 - 0.32); *b = 0 (0 - 0.17); *c = 0 (0 - 0.558); *d = 0 (0 - 1.33); *e = 0 (0 - 0.877); *f = 0 (0 -1.69); *g = 0 (0 - 0.54); *h = 0 (0 -0.74)
