# Supplementary material for: Bioinformatic analysis of meningococcal Msf and Opc to inform vaccine antigen design
Source: PLoS One. 2018 Mar 16;13(3):e0193940. doi: 10.1371/journal.pone.0193940 (PMC5856348; doi:10.1371/journal.pone.0193940)
Supplement: S4 Table — (PDF) [file pone.0193940.s004.pdf]

| Isolate id | Isolate | Species | Serogroup | Clonal complex | Epidemiology | Country | Msf sequence variant | Opc +/- |
|---|---|---|---|---|---|---|---|---|
| 1 | A4/M1027 | Neisseria meningitidis | A | ST-4 complex | Epidemic | USA | SV-5 | Opc+ |
| 2 | 120M | Neisseria meningitidis | A | ST-1 complex | Endemic | Pakistan | SV-5 | Opc+ |
| 7 | 7891 | Neisseria meningitidis | A | ST-5 complex | Epidemic | Finland | SV-5 | Opc+ |
| 10 | 6748 | Neisseria meningitidis | A | ST-1 complex | Epidemic | Canada | SV-5 | Opc+ |
| 11 | 129 | Neisseria meningitidis | A | ST-1 complex | Epidemic | Germany | SV-5 | Opc+ |
| 13 | 139M | Neisseria meningitidis | A | ST-1 complex | Endemic | Philippines | SV-5 | Opc+ |
| 19 | S3131 | Neisseria meningitidis | A | ST-4 complex | Endemic | Ghana | SV-5 | Opc+ |
| 24 | S4355 | Neisseria meningitidis | A | ST-5 complex | Endemic | Denmark | SV-5 | Opc+ |
| 30 | 14 | Neisseria meningitidis | NG | ST-53 complex | Endemic | Germany | SV-2 | Opc- |
| 31 | 10 | Neisseria meningitidis | A | ST-4 complex | Endemic | Burkina Faso | SV-5 | Opc+ |
| 34 | 20 | Neisseria meningitidis | A | ST-1 complex | No value | Niger | SV-5 | Opc+ |
| 35 | 26 | Neisseria meningitidis | A | ST-4 complex | Epidemic | Niger | SV-5 | Opc+ |
| 46 | 255 | Neisseria meningitidis | A | ST-4 complex | Endemic | Burkina Faso | SV-5 | Opc+ |
| 52 | 243 | Neisseria meningitidis | A | ST-4 complex | Endemic | Cameroon | SV-5 | Opc+ |
| 61 | 393 | Neisseria meningitidis | A | ST-1 complex | Epidemic | Greece | SV-5 | Opc+ |
| 64 | 254 | Neisseria meningitidis | A | ST-1 complex | Endemic | Djibouti | SV-5 | Opc+ |
| 67 | S5611 | Neisseria meningitidis | A | ST-1 complex | Endemic | Australia | SV-5 | Opc+ |
| 82 | 11-004 | Neisseria meningitidis | A | ST-5 complex | Epidemic | China | SV-5 | Opc+ |
| 84 | IAL2229 | Neisseria meningitidis | A | ST-5 complex | Pandemic | Brazil | SV-5 | Opc+ |
| 90 | CN100 | Neisseria meningitidis | A | No value | Epidemic | UK | SV-3 | Opc+ |
| 120 | F4698 | Neisseria meningitidis | A | ST-5 complex | No value | Saudi Arabia | SV-5 | Opc+ |
| 128 | F6124 | Neisseria meningitidis | A | ST-5 complex | No value | Chad | SV-5 | Opc+ |
| 160 | 1014 | Neisseria meningitidis | A | ST-4 complex | Endemic | Sudan | SV-5 | Opc+ |
| 210 | H1964 | Neisseria meningitidis | A | ST-5 complex | Epidemic | UK | SV-5 | Opc+ |
| 237 | H44/76 | Neisseria meningitidis | B | ST-32 complex | Epidemic | Norway | SV-1 | Opc+ |
| 238 | 153 | Neisseria meningitidis | A | ST-5 complex | Pandemic | China | SV-5 | Opc+ |
| 239 | 154 | Neisseria meningitidis | A | ST-5 complex | Pandemic | China | SV-5 | Opc+ |
| 240 | MC58 | Neisseria meningitidis | B | ST-32 complex | No value | UK | SV-1 | Opc+ |
| 299 | 80049 | Neisseria meningitidis | A | ST-5 complex | Endemic | China | SV-5 | Opc+ |
| 314 | D1 | Neisseria meningitidis | C | ST-11 complex | Endemic | Mali | SV-2 | Opc- |
| 316 | D8 | Neisseria meningitidis | A | ST-4 complex | Endemic | Mali | SV-5 | Opc+ |
| 340 | 196/87 | Neisseria meningitidis | C | ST-32 complex | No value | Norway | SV-1 | Opc+ |
| 343 | 500 | Neisseria meningitidis | C | ST-11 complex | Endemic | Italy | SV-2 | Opc- |
| 344 | F1576 | Neisseria meningitidis | C | ST-11 complex | Endemic | Ghana | SV-2 | Opc- |
| 349 | 38VI | Neisseria meningitidis | B | ST-11 complex | No value | USA | SV-2 | Opc- |
| 369 | M597 | Neisseria meningitidis | C | ST-11 complex | No value | Israel | SV-2 | Opc- |
| 387 | 2059001 | Neisseria meningitidis | A | ST-4 complex | Endemic | Mali | SV-5 | Opc+ |
| 391 | 90/18311 | Neisseria meningitidis | C | ST-11 complex | No value | UK | SV-2 | Opc- |
| 398 | BZ 10 | Neisseria meningitidis | B | ST-8 complex | No value | The Netherlands | SV-10 | Opc- |
| 400 | BZ 83 | Neisseria meningitidis | B | ST-32 complex | No value | The Netherlands | SV-1 | Opc+ |
| 401 | BZ 133 | Neisseria meningitidis | B | ST-1 complex | No value | The Netherlands | SV-5 | Opc+ |
| 403 | BZ 147 | Neisseria meningitidis | B | ST-41/44 complex | No value | The Netherlands | SV-1 | Opc+ |
| 407 | BZ 163 | Neisseria meningitidis | B | ST-8 complex | No value | The Netherlands | SV-10 | Opc- |
| 408 | BZ 169 | Neisseria meningitidis | B | ST-32 complex | No value | The Netherlands | SV-1 | Opc+ |
| 409 | BZ 198 | Neisseria meningitidis | B | ST-41/44 complex | Endemic | The Netherlands | SV-6 | Opc+ |
| 410 | BZ 232 | Neisseria meningitidis | B | ST-37 complex | No value | The Netherlands | SV-7 | Opc- |
| 411 | DK 24 | Neisseria meningitidis | B | ST-4240/6688 complex | No value | Denmark | SV-5 | Opc- |
| 412 | DK 353 | Neisseria meningitidis | B | ST-37 complex | No value | Denmark | SV-7 | Opc- |
| 413 | EG 328 | Neisseria meningitidis | B | ST-18 complex | No value | Germany | SV-7 | Opc- |
| 414 | EG 327 | Neisseria meningitidis | B | ST-18 complex | No value | Germany | SV-5 | Opc- |
| 415 | EG 329 | Neisseria meningitidis | B | ST-32 complex | No value | Germany | SV-1 | Opc+ |
| 416 | EG 011 | Neisseria meningitidis | B | No value | No value | Germany | SV-8 | Opc- |
| 417 | NG 3/88 | Neisseria meningitidis | B | No value | No value | Norway | SV-7 | Opc+ |
| 418 | NG 4/88 | Neisseria meningitidis | B | No value | No value | Norway | SV-12 | Opc+ |
| 419 | NG 6/88 | Neisseria meningitidis | B | ST-269 complex | No value | Norway | SV-8 | Opc+ |
| 420 | NG F26 | Neisseria meningitidis | B | ST-269 complex | No value | Norway | SV-13 | Opc+ |
| 421 | NG H15 | Neisseria meningitidis | B | ST-41/44 complex | Carrier | Norway | SV-10 | Opc+ |
| 422 | NG H41 | Neisseria meningitidis | B | No value | No value | Norway | SV-5 | Opc+ |
| 423 | NG H38 | Neisseria meningitidis | B | No value | No value | Norway | SV-8 | Opc- |
| 424 | NG E31 | Neisseria meningitidis | B | ST-364 complex | No value | Norway | SV-7 | Opc- |
| 425 | NG G40 | Neisseria meningitidis | B | No value | Carrier | Norway | SV-1 | Opc+ |
| 426 | NG E28 | Neisseria meningitidis | B | No value | Carrier | Norway | SV-1 | Opc- |
| 427 | NG E30 | Neisseria meningitidis | B | ST-41/44 complex | Carrier | Norway | SV-10 | Opc+ |
| 428 | NG H36 | Neisseria meningitidis | B | ST-41/44 complex | Carrier | Norway | SV-10 | Opc+ |
| 430 | NG 080 | Neisseria meningitidis | B | ST-32 complex | No value | Norway | SV-1 | Opc+ |
| 431 | NG144/82 | Neisseria meningitidis | B | ST-32 complex | No value | Norway | SV-1 | Opc+ |
| 434 | NG PB24 | Neisseria meningitidis | B | ST-32 complex | Epidemic | Norway | SV-1 | Opc+ |
| 436 | NG P20 | Neisseria meningitidis | B | ST-11 complex | No value | Norway | SV-2 | Opc- |
| 441 | 8680 | Neisseria meningitidis | B | ST-32 complex | No value | Chile | SV-1 | Opc+ |
| 442 | 297-0 | Neisseria meningitidis | B | ST-254 complex | Carrier | Chile | SV-1 | Opc+ |
| 443 | 3906 | Neisseria meningitidis | B | No value | No value | China | SV-6 | Opc+ |
| 444 | SWZ107 | Neisseria meningitidis | B | ST-35 complex | No value | Switzerland | SV-12 | Opc+ |
| 445 | 528 | Neisseria meningitidis | B | ST-18 complex | Endemic | Russia | SV-7 | Opc- |

| | | | | | | | | |
|---|---|---|---|---|---|---|---|---|
| 446 | 1000 | Neisseria meningitidis | B | ST-18 complex | Endemic | Russia | SV-7 | Opc+ |
| 451 | 14/1455 | Neisseria meningitidis | A | ST-5 complex | Pandemic | Russia | SV-5 | Opc+ |
| 466 | 371 | Neisseria meningitidis | A | ST-1 complex | No value | India | SV-5 | Opc+ |
| 467 | 690 | Neisseria meningitidis | A | ST-4 complex | No value | India | SV-5 | Opc+ |
| 468 | BRAZ10 | Neisseria meningitidis | C | ST-11 complex | No value | Brazil | SV-2 | Opc- |
| 488 | 106 | Neisseria meningitidis | A | ST-1 complex | Epidemic | Morocco | SV-5 | Opc+ |
| 492 | 79128 | Neisseria meningitidis | A | ST-1 complex | Epidemic | China | SV-5 | Opc+ |
| 493 | 322/85 | Neisseria meningitidis | A | ST-1 complex | Endemic | Germany | SV-5 | Opc+ |
| 494 | 79126 | Neisseria meningitidis | A | ST-1 complex | Epidemic | China | SV-5 | Opc+ |
| 507 | MA-5756 | Neisseria meningitidis | C | ST-11 complex | No value | Spain | SV-2 | Opc- |
| 597 | 92001 | Neisseria meningitidis | A | ST-5 complex | Epidemic | China | SV-5 | Opc+ |
| 613 | Z2491 | Neisseria meningitidis | A | ST-4 complex | Epidemic | The Gambia | SV-5 | Opc+ |
| 638 | G2136 | Neisseria meningitidis | B | ST-8 complex | No value | UK | SV-10 | Opc- |
| 639 | B6116/77 | Neisseria meningitidis | B | ST-8 complex | No value | Iceland | SV-10 | Opc- |
| 640 | SB25 | Neisseria meningitidis | C | ST-8 complex | No value | South Africa | SV-10 | Opc- |
| 641 | 94/155 | Neisseria meningitidis | C | ST-8 complex | No value | New Zealand | SV-10 | Opc- |
| 642 | 312 901 | Neisseria meningitidis | C | ST-8 complex | No value | UK | SV-10 | Opc- |
| 643 | AK22 | Neisseria meningitidis | B | ST-8 complex | No value | Greece | SV-14 | Opc- |
| 644 | L93/4286 | Neisseria meningitidis | C | ST-11 complex | No value | UK | SV-2 | Opc- |
| 645 | 204/92 | Neisseria meningitidis | B | ST-32 complex | No value | Cuba | SV-1 | Opc+ |
| 646 | 400 | Neisseria meningitidis | B | ST-41/44 complex | No value | Austria | SV-6 | Opc+ |
| 647 | AK50 | Neisseria meningitidis | B | ST-41/44 complex | No value | Greece | SV-1 | Opc+ |
| 648 | M-101/93 | Neisseria meningitidis | B | ST-41/44 complex | No value | Iceland | SV-1 | Opc+ |
| 649 | 50/94 | Neisseria meningitidis | B | ST-41/44 complex | No value | Norway | SV-1 | Opc+ |
| 650 | M40/94 | Neisseria meningitidis | B | ST-41/44 complex | No value | Chile | SV-6 | Opc+ |
| 651 | 931905 | Neisseria meningitidis | B | ST-41/44 complex | No value | The Netherlands | SV-6 | Opc+ |
| 652 | N45/96 | Neisseria meningitidis | B | ST-41/44 complex | No value | Norway | SV-6 | Opc+ |
| 653 | 91/40 | Neisseria meningitidis | B | ST-41/44 complex | Epidemic | New Zealand | SV-6 | Opc+ |
| 654 | 88/03415 | Neisseria meningitidis | B | ST-41/44 complex | No value | UK | SV-1 | Opc+ |
| 655 | E32 | Neisseria meningitidis | Z | ST-334 complex | No value | Norway | SV-11 | Opc- |
| 656 | E26 | Neisseria meningitidis | X | ST-198 complex | No value | Norway | SV-7 | Opc+ |
| 657 | 860060 | Neisseria meningitidis | X | ST-750 complex | No value | The Netherlands | SV-1 | Opc+ |
| 658 | 890326 | Neisseria meningitidis | Z | ST-103 complex | No value | The Netherlands | SV-5 | Opc- |
| 659 | A22 | Neisseria meningitidis | W | ST-22 complex | No value | Norway | SV-12 | Opc+ |
| 660 | 71/94 | Neisseria meningitidis | Y | ST-23 complex | No value | Norway | SV-11 | Opc+ |
| 661 | 860800 | Neisseria meningitidis | Y | ST-167 complex | No value | The Netherlands | SV-5 | Opc+ |
| 662 | 2837 | Neisseria meningitidis | C | ST-11 complex | Endemic | UK | SV-2 | Opc- |
| 663 | 2839 | Neisseria meningitidis | C | ST-11 complex | Endemic | UK | SV-2 | Opc- |
| 664 | 2838 | Neisseria meningitidis | C | ST-11 complex | Endemic | UK | SV-2 | Opc- |
| 665 | 2845 | Neisseria meningitidis | C | ST-11 complex | Endemic | UK | SV-2 | Opc- |
| 666 | 2843 | Neisseria meningitidis | C | ST-11 complex | Endemic | UK | SV-2 | Opc- |
| 667 | 2842 | Neisseria meningitidis | C | ST-11 complex | Endemic | UK | SV-2 | Opc- |
| 669 | 2846 | Neisseria meningitidis | C | ST-11 complex | Endemic | UK | SV-2 | Opc- |
| 670 | 2840 | Neisseria meningitidis | C | ST-11 complex | Endemic | UK | SV-2 | Opc- |
| 671 | 2844 | Neisseria meningitidis | C | ST-11 complex | Endemic | UK | SV-2 | Opc- |
| 672 | 2847 | Neisseria meningitidis | C | ST-11 complex | Endemic | UK | SV-2 | Opc- |
| 698 | FAM18 | Neisseria meningitidis | C | ST-11 complex | Endemic | USA | SV-2 | Opc- |
| 890 | OX9930407 | Neisseria meningitidis | NG | No value | No value | No value | SV-12 | Opc- |
| 930 | 0013/93 | Neisseria meningitidis | NG | ST-116 complex | Carrier | Czech Republic | SV-12 | Opc+ |
| 931 | 0014/93 | Neisseria meningitidis | NG | ST-116 complex | Carrier | Czech Republic | SV-12 | Opc+ |
| 932 | 0015/93 | Neisseria meningitidis | C | No value | Carrier | Czech Republic | SV-8 | Opc- |
| 933 | 0016/93 | Neisseria meningitidis | NG | ST-18 complex | Carrier | Czech Republic | SV-5 | Opc- |
| 934 | 0017/93 | Neisseria meningitidis | NG | No value | Carrier | Czech Republic | SV-12 | Opc+ |
| 935 | 0018/93 | Neisseria meningitidis | E | ST-106 complex | Carrier | Czech Republic | SV-1 | Opc+ |
| 936 | 0019/93 | Neisseria meningitidis | E | ST-106 complex | Carrier | Czech Republic | SV-1 | Opc+ |
| 939 | 0022/93 | Neisseria meningitidis | C | ST-11 complex | Carrier | Czech Republic | SV-2 | Opc- |
| 940 | 0023/93 | Neisseria meningitidis | C | ST-11 complex | Carrier | Czech Republic | SV-2 | Opc- |
| 942 | 0025/93 | Neisseria meningitidis | C | ST-11 complex | Carrier | Czech Republic | SV-2 | Opc- |
| 943 | 0029/93 | Neisseria meningitidis | NG | No value | Carrier | Czech Republic | SV-2 | Opc- |
| 944 | 0030/93 | Neisseria meningitidis | NG | ST-92 complex | Carrier | Czech Republic | SV-1 | Opc+ |
| 945 | 0031/93 | Neisseria meningitidis | B | No value | Carrier | Czech Republic | SV-5 | Opc- |
| 946 | 0033/93 | Neisseria meningitidis | C | ST-11 complex | Carrier | Czech Republic | SV-2 | Opc- |
| 947 | 0034/93 | Neisseria meningitidis | NG | No value | Carrier | Czech Republic | SV-2 | Opc- |
| 948 | 0035/93 | Neisseria meningitidis | C | ST-11 complex | Carrier | Czech Republic | SV-2 | Opc- |
| 949 | 0036/93 | Neisseria meningitidis | C | ST-11 complex | Carrier | Czech Republic | SV-2 | Opc- |
| 950 | 0037/93 | Neisseria meningitidis | C | ST-11 complex | Carrier | Czech Republic | SV-2 | Opc- |
| 951 | 0038/93 | Neisseria meningitidis | X | No value | Carrier | Czech Republic | SV-5 | Opc+ |
| 952 | 0039/93 | Neisseria meningitidis | C | ST-11 complex | Carrier | Czech Republic | SV-2 | Opc- |
| 953 | 0040/93 | Neisseria meningitidis | X | No value | Carrier | Czech Republic | SV-5 | Opc+ |
| 954 | 0042/93 | Neisseria meningitidis | C | ST-11 complex | Carrier | Czech Republic | SV-2 | Opc- |
| 955 | 0043/93 | Neisseria meningitidis | C | ST-11 complex | Carrier | Czech Republic | SV-2 | Opc- |
| 956 | 0044/93 | Neisseria meningitidis | NG | ST-92 complex | Carrier | Czech Republic | SV-1 | Opc+ |
| 957 | 0045/93 | Neisseria meningitidis | C | ST-11 complex | Carrier | Czech Republic | SV-2 | Opc- |
| 958 | 0046/93 | Neisseria meningitidis | C | ST-11 complex | Carrier | Czech Republic | SV-2 | Opc- |

| | | | | | | | | |
|---|---|---|---|---|---|---|---|---|
| 960 | 0048/93 | Neisseria meningitidis | NG | ST-103 complex | Carrier | Czech Republic | SV-5 | Opc+ |
| 961 | 0054/93 | Neisseria meningitidis | NG | No value | Carrier | Czech Republic | SV-11 | Opc+ |
| 964 | 0057/93 | Neisseria meningitidis | B | No value | Carrier | Czech Republic | SV-12 | Opc- |
| 965 | 0058/93 | Neisseria meningitidis | B | No value | Carrier | Czech Republic | SV-12 | Opc- |
| 966 | 0059/93 | Neisseria meningitidis | Y | ST-92 complex | Carrier | Czech Republic | SV-1 | Opc+ |
| 967 | 0060/93 | Neisseria meningitidis | NG | ST-35 complex | Carrier | Czech Republic | SV-12 | Opc+ |
| 968 | 0061/93 | Neisseria meningitidis | NG | ST-22 complex | Carrier | Czech Republic | SV-12 | Opc+ |
| 969 | 0062/93 | Neisseria meningitidis | B | ST-32 complex | Carrier | Czech Republic | SV-1 | Opc+ |
| 970 | 0063/93 | Neisseria meningitidis | NG | No value | Carrier | Czech Republic | SV-2 | Opc- |
| 971 | 0065/93 | Neisseria meningitidis | NG | ST-92 complex | Carrier | Czech Republic | SV-1 | Opc+ |
| 972 | 0066/93 | Neisseria meningitidis | NG | ST-53 complex | Carrier | Czech Republic | SV-2 | Opc- |
| 973 | 0067/93 | Neisseria meningitidis | C | ST-41/44 complex | Carrier | Czech Republic | SV-10 | Opc+ |
| 974 | 0068/93 | Neisseria meningitidis | Z | ST-92 complex | Carrier | Czech Republic | SV-1 | Opc+ |
| 975 | 0069/93 | Neisseria meningitidis | NG | ST-41/44 complex | Carrier | Czech Republic | SV-10 | Opc+ |
| 976 | 0070/93 | Neisseria meningitidis | B | ST-106 complex | Carrier | Czech Republic | SV-1 | Opc+ |
| 977 | 0071/93 | Neisseria meningitidis | C | ST-41/44 complex | Carrier | Czech Republic | SV-10 | Opc+ |
| 978 | 0073/93 | Neisseria meningitidis | C | ST-11 complex | Carrier | Czech Republic | SV-2 | Opc- |
| 979 | 0074/93 | Neisseria meningitidis | C | ST-11 complex | Carrier | Czech Republic | SV-2 | Opc- |
| 980 | 0077/93 | Neisseria meningitidis | NG | No value | Carrier | Czech Republic | SV-5 | Opc+ |
| 981 | 0078/93 | Neisseria meningitidis | C | ST-116 complex | Carrier | Czech Republic | SV-12 | Opc+ |
| 982 | 0079/93 | Neisseria meningitidis | C | ST-11 complex | Carrier | Czech Republic | SV-2 | Opc- |
| 983 | 0081/93 | Neisseria meningitidis | NG | ST-106 complex | Carrier | Czech Republic | SV-12 | Opc+ |
| 984 | 0083/93 | Neisseria meningitidis | NG | ST-53 complex | Carrier | Czech Republic | SV-2 | Opc- |
| 985 | 0084/93 | Neisseria meningitidis | Z | ST-103 complex | Carrier | Czech Republic | SV-5 | Opc+ |
| 986 | 0085/93 | Neisseria meningitidis | NG | ST-92 complex | Carrier | Czech Republic | SV-1 | Opc+ |
| 987 | 0086/93 | Neisseria meningitidis | NG | ST-92 complex | Carrier | Czech Republic | SV-1 | Opc+ |
| 988 | 0087/93 | Neisseria meningitidis | NG | ST-106 complex | Carrier | Czech Republic | SV-1 | Opc+ |
| 989 | 0088/93 | Neisseria meningitidis | Z | No value | Carrier | Czech Republic | SV-5 | Opc+ |
| 991 | 0091/93 | Neisseria meningitidis | B | ST-41/44 complex | Carrier | Czech Republic | SV-10 | Opc+ |
| 992 | 0093/93 | Neisseria meningitidis | C | ST-11 complex | Carrier | Czech Republic | SV-2 | Opc- |
| 993 | 0095/93 | Neisseria meningitidis | C | ST-11 complex | Carrier | Czech Republic | SV-2 | Opc- |
| 994 | 0096/93 | Neisseria meningitidis | C | ST-11 complex | Carrier | Czech Republic | SV-2 | Opc- |
| 1017 | BM33a | Neisseria meningitidis | B | ST-35 complex | Carrier | Greece | SV-12 | Opc+ |
| 1018 | BM33e | Neisseria meningitidis | B | ST-35 complex | Carrier | Greece | SV-12 | Opc+ |
| 1019 | BM35 | Neisseria meningitidis | B | ST-162 complex | Sporadic case | Greece | SV-1 | Opc+ |
| 1020 | BM55 | Neisseria meningitidis | B | No value | Sporadic case | Greece | SV-10 | Opc+ |
| 1021 | BM65 | Neisseria meningitidis | B | No value | Sporadic case | Greece | SV-10 | Opc+ |
| 1038 | 8013 | Neisseria meningitidis | C | ST-18 complex | Sporadic case | France | SV-5 | Opc- |
| 1057 | BM95 | Neisseria meningitidis | B | No value | Sporadic case | Greece | SV-12 | Opc+ |
| 1058 | BM95a | Neisseria meningitidis | B | No value | Carrier | Greece | SV-12 | Opc+ |
| 1059 | BM95b | Neisseria meningitidis | B | No value | Carrier | Greece | SV-12 | Opc+ |
| 1099 | BM68b | Neisseria meningitidis | NG | ST-231 complex | Carrier | Greece | SV-10 | Opc- |
| 1168 | BM33 | Neisseria meningitidis | B | ST-32 complex | Sporadic case | Greece | SV-1 | Opc+ |
| 1169 | BM45 | Neisseria meningitidis | C | ST-11 complex | Sporadic case | Greece | SV-2 | Opc- |
| 1170 | BM45a | Neisseria meningitidis | C | ST-11 complex | Carrier | Greece | SV-2 | Opc- |
| 1171 | BM48 | Neisseria meningitidis | C | ST-11 complex | Sporadic case | Greece | SV-2 | Opc- |
| 1172 | BM48a | Neisseria meningitidis | C | ST-11 complex | Carrier | Greece | SV-2 | Opc- |
| 1173 | BM59 | Neisseria meningitidis | B | ST-41/44 complex | Sporadic case | Greece | SV-6 | Opc+ |
| 1174 | BM59a | Neisseria meningitidis | B | ST-41/44 complex | Carrier | Greece | SV-6 | Opc+ |
| 1175 | BM59b | Neisseria meningitidis | B | ST-41/44 complex | Carrier | Greece | SV-6 | Opc+ |
| 1176 | BM59c | Neisseria meningitidis | B | ST-41/44 complex | Carrier | Greece | SV-6 | Opc+ |
| 1177 | BM80 | Neisseria meningitidis | B | ST-8 complex | Sporadic case | Greece | SV-10 | Opc- |
| 1178 | W-72 | Neisseria meningitidis | C | ST-11 complex | Sporadic case | Greece | SV-2 | Opc- |
| 1179 | W-72a | Neisseria meningitidis | C | ST-11 complex | Carrier | Greece | SV-2 | Opc- |
| 1180 | W-72b | Neisseria meningitidis | C | ST-11 complex | Carrier | Greece | SV-2 | Opc- |
| 1181 | W-138 | Neisseria meningitidis | B | ST-11 complex | Sporadic case | Greece | SV-2 | Opc- |
| 1182 | W-138a | Neisseria meningitidis | A | ST-103 complex | Carrier | Greece | SV-5 | Opc+ |
| 1191 | BM33b | Neisseria meningitidis | B | ST-35 complex | Carrier | Greece | SV-12 | Opc+ |
| 1192 | BM33c | Neisseria meningitidis | B | ST-35 complex | Carrier | Greece | SV-12 | Opc+ |
| 1193 | BM33d | Neisseria meningitidis | B | ST-35 complex | Carrier | Greece | SV-12 | Opc+ |
| 1194 | BM34 | Neisseria meningitidis | B | ST-103 complex | Sporadic case | Greece | SV-11 | Opc+ |
| 1195 | BM49a | Neisseria meningitidis | B | ST-35 complex | Carrier | Greece | SV-12 | Opc+ |
| 1196 | BM49b | Neisseria meningitidis | B | ST-35 complex | Carrier | Greece | SV-12 | Opc+ |
| 1197 | BM34a | Neisseria meningitidis | B | ST-103 complex | Carrier | Greece | SV-11 | Opc+ |
| 1198 | BM68 | Neisseria meningitidis | B | ST-162 complex | Sporadic case | Greece | SV-1 | Opc+ |
| 1199 | BM80a | Neisseria meningitidis | B | ST-162 complex | Carrier | Greece | SV-1 | Opc+ |
| 1200 | W-134 | Neisseria meningitidis | B | ST-162 complex | Sporadic case | Greece | SV-1 | Opc+ |
| 1201 | W-134a | Neisseria meningitidis | B | ST-162 complex | Carrier | Greece | SV-1 | Opc+ |
| 1202 | W-134b | Neisseria meningitidis | B | ST-162 complex | Carrier | Greece | SV-1 | Opc+ |
| 1203 | BM55a | Neisseria meningitidis | B | No value | Carrier | Greece | SV-10 | Opc+ |
| 1204 | BM65a | Neisseria meningitidis | B | No value | Carrier | Greece | SV-10 | Opc+ |
| 1205 | BM65b | Neisseria meningitidis | B | No value | Carrier | Greece | SV-14 | Opc+ |
| 1206 | BM65c | Neisseria meningitidis | B | No value | Carrier | Greece | SV-10 | Opc+ |
| 1207 | M99 240593 | Neisseria meningitidis | C | ST-212 complex | Epidemic | UK | SV-14 | Opc+ |

| | | | | | | | | |
|---|---|---|---|---|---|---|---|---|
| 1570 | 0101/93 | Neisseria meningitidis | NG | ST-11 complex | Carrier | Czech Republic | SV-2 | Opc- |
| 1571 | 0102/93 | Neisseria meningitidis | C | ST-11 complex | Carrier | Czech Republic | SV-2 | Opc- |
| 1572 | 0103/93 | Neisseria meningitidis | C | ST-11 complex | Carrier | Czech Republic | SV-2 | Opc- |
| 1573 | 0104/93 | Neisseria meningitidis | C | ST-11 complex | Carrier | Czech Republic | SV-2 | Opc- |
| 1574 | 0105/93 | Neisseria meningitidis | C | No value | Carrier | Czech Republic | SV-5 | Opc+ |
| 1576 | 0107/93 | Neisseria meningitidis | E | ST-106 complex | Carrier | Czech Republic | SV-12 | Opc+ |
| 1578 | 0109/93 | Neisseria meningitidis | C | ST-11 complex | Carrier | Czech Republic | SV-2 | Opc- |
| 1579 | 0110/93 | Neisseria meningitidis | NG | ST-92 complex | Carrier | Czech Republic | SV-1 | Opc+ |
| 1580 | 0111/93 | Neisseria meningitidis | Y | No value | Carrier | Czech Republic | SV-12 | Opc+ |
| 1581 | 0112/93 | Neisseria meningitidis | NG | No value | Carrier | Czech Republic | SV-12 | Opc+ |
| 1582 | 0113/93 | Neisseria meningitidis | C | ST-11 complex | Carrier | Czech Republic | SV-2 | Opc- |
| 1583 | 0115/93 | Neisseria meningitidis | C | ST-11 complex | Carrier | Czech Republic | SV-2 | Opc- |
| 1585 | 0117/93 | Neisseria meningitidis | B | ST-41/44 complex | Carrier | Czech Republic | SV-1 | Opc+ |
| 1586 | 0118/93 | Neisseria meningitidis | NG | ST-18 complex | Carrier | Czech Republic | SV-7 | Opc- |
| 1587 | 0119/93 | Neisseria meningitidis | C | ST-11 complex | Carrier | Czech Republic | SV-2 | Opc- |
| 1588 | 0120/93 | Neisseria meningitidis | B | ST-41/44 complex | Carrier | Czech Republic | SV-10 | Opc+ |
| 1589 | 0198/93 | Neisseria meningitidis | NG | ST-92 complex | Carrier | Czech Republic | SV-1 | Opc+ |
| 1590 | 0199/93 | Neisseria meningitidis | B | No value | Carrier | Czech Republic | SV-10 | Opc+ |
| 1591 | 0200/93 | Neisseria meningitidis | B | No value | Carrier | Czech Republic | SV-10 | Opc+ |
| 1593 | 0202/93 | Neisseria meningitidis | B | No value | Carrier | Czech Republic | SV-10 | Opc+ |
| 1594 | 0203/93 | Neisseria meningitidis | Z | ST-116 complex | Carrier | Czech Republic | SV-12 | Opc+ |
| 1595 | 0204/93 | Neisseria meningitidis | E | ST-174 complex | Carrier | Czech Republic | SV-7 | Opc+ |
| 1596 | 0205/93 | Neisseria meningitidis | NG | No value | Carrier | Czech Republic | SV-1 | Opc- |
| 1597 | 0206/93 | Neisseria meningitidis | NG | ST-53 complex | Carrier | Czech Republic | SV-2 | Opc- |
| 1598 | 0208/93 | Neisseria meningitidis | NG | ST-106 complex | Carrier | Czech Republic | SV-1 | Opc+ |
| 1599 | 0209/93 | Neisseria meningitidis | X | No value | Carrier | Czech Republic | SV-1 | Opc- |
| 1600 | 0210/93 | Neisseria meningitidis | E | No value | Carrier | Czech Republic | SV-2 | Opc- |
| 1601 | 0211/93 | Neisseria meningitidis | NG | ST-269 complex | Carrier | Czech Republic | SV-8 | Opc+ |
| 1602 | 0212/93 | Neisseria meningitidis | B | No value | Carrier | Czech Republic | SV-2 | Opc- |
| 1603 | 0213/93 | Neisseria meningitidis | B | ST-41/44 complex | Carrier | Czech Republic | SV-10 | Opc+ |
| 1604 | 0214/93 | Neisseria meningitidis | NG | ST-41/44 complex | Carrier | Czech Republic | SV-10 | Opc+ |
| 1605 | 0215/93 | Neisseria meningitidis | NG | ST-41/44 complex | Carrier | Czech Republic | SV-10 | Opc+ |
| 1606 | 0216/93 | Neisseria meningitidis | B | ST-41/44 complex | Carrier | Czech Republic | SV-10 | Opc+ |
| 1607 | 0217/93 | Neisseria meningitidis | B | ST-41/44 complex | Carrier | Czech Republic | SV-10 | Opc+ |
| 1608 | 0218/93 | Neisseria meningitidis | Y | ST-92 complex | Carrier | Czech Republic | SV-1 | Opc+ |
| 1609 | 0219/93 | Neisseria meningitidis | NG | ST-116 complex | Carrier | Czech Republic | SV-12 | Opc+ |
| 1610 | 0220/93 | Neisseria meningitidis | B | ST-92 complex | Carrier | Czech Republic | SV-1 | Opc+ |
| 1611 | 0221/93 | Neisseria meningitidis | E | ST-106 complex | Carrier | Czech Republic | SV-1 | Opc+ |
| 1612 | 0222/93 | Neisseria meningitidis | E | ST-106 complex | Carrier | Czech Republic | SV-1 | Opc+ |
| 1613 | 0223/93 | Neisseria meningitidis | NG | ST-116 complex | Carrier | Czech Republic | SV-12 | Opc+ |
| 1614 | 0224/93 | Neisseria meningitidis | Y | ST-92 complex | Carrier | Czech Republic | SV-1 | Opc+ |
| 1615 | 0225/93 | Neisseria meningitidis | B | ST-41/44 complex | Carrier | Czech Republic | SV-10 | Opc+ |
| 1616 | 0226/93 | Neisseria meningitidis | B | ST-41/44 complex | Carrier | Czech Republic | SV-10 | Opc+ |
| 1617 | 0227/93 | Neisseria meningitidis | B | ST-41/44 complex | Carrier | Czech Republic | SV-10 | Opc+ |
| 1618 | 0228/93 | Neisseria meningitidis | B | ST-41/44 complex | Carrier | Czech Republic | SV-10 | Opc+ |
| 1619 | 0229/93 | Neisseria meningitidis | NG | No value | Carrier | Czech Republic | SV-5 | Opc+ |
| 1620 | 0230/93 | Neisseria meningitidis | NG | ST-231 complex | Carrier | Czech Republic | SV-7 | Opc- |
| 1621 | 0231/93 | Neisseria meningitidis | NG | No value | Carrier | Czech Republic | SV-11 | Opc- |
| 1622 | 0232/93 | Neisseria meningitidis | C | No value | Carrier | Czech Republic | SV-5 | Opc+ |
| 1623 | 0233/93 | Neisseria meningitidis | B | No value | Carrier | Czech Republic | SV-10 | Opc+ |
| 1624 | 0234/93 | Neisseria meningitidis | NG | ST-106 complex | Carrier | Czech Republic | SV-1 | Opc+ |
| 1625 | 0236/93 | Neisseria meningitidis | X | No value | Carrier | Czech Republic | SV-11 | Opc+ |
| 1626 | 0237/93 | Neisseria meningitidis | B | No value | Carrier | Czech Republic | SV-10 | Opc- |
| 1627 | 0238/93 | Neisseria meningitidis | NG | No value | Carrier | Czech Republic | SV-1 | Opc- |
| 1628 | 0239/93 | Neisseria meningitidis | B | No value | Carrier | Czech Republic | SV-1 | Opc- |
| 1629 | 0240/93 | Neisseria meningitidis | NG | ST-116 complex | Carrier | Czech Republic | SV-12 | Opc+ |
| 1630 | 0241/93 | Neisseria meningitidis | NG | ST-549 complex | Carrier | Czech Republic | SV-11 | Opc+ |
| 1631 | 0242/93 | Neisseria meningitidis | B | No value | Carrier | Czech Republic | SV-10 | Opc+ |
| 1633 | 0244/93 | Neisseria meningitidis | B | ST-41/44 complex | Carrier | Czech Republic | SV-10 | Opc+ |
| 1636 | 0248/93 | Neisseria meningitidis | C | No value | Carrier | Czech Republic | SV-5 | Opc+ |
| 1637 | 0250/93 | Neisseria meningitidis | NG | ST-106 complex | Carrier | Czech Republic | SV-1 | Opc+ |
| 1638 | 0251/93 | Neisseria meningitidis | B | No value | Carrier | Czech Republic | SV-10 | Opc+ |
| 1639 | 0252/93 | Neisseria meningitidis | NG | ST-53 complex | Carrier | Czech Republic | SV-2 | Opc- |
| 1640 | 0253/93 | Neisseria meningitidis | B | ST-41/44 complex | Carrier | Czech Republic | SV-10 | Opc+ |
| 1641 | 0254/93 | Neisseria meningitidis | C | No value | Carrier | Czech Republic | SV-5 | Opc- |
| 1642 | 0255/93 | Neisseria meningitidis | B | ST-41/44 complex | Carrier | Czech Republic | SV-10 | Opc+ |
| 1643 | 0256/93 | Neisseria meningitidis | B | No value | Carrier | Czech Republic | SV-12 | Opc+ |
| 1644 | 0257/93 | Neisseria meningitidis | X | No value | Carrier | Czech Republic | SV-2 | Opc- |
| 1645 | 0258/93 | Neisseria meningitidis | B | ST-41/44 complex | Carrier | Czech Republic | SV-10 | Opc+ |
| 1646 | 0265/93 | Neisseria meningitidis | X | No value | Carrier | Czech Republic | SV-1 | Opc- |
| 1647 | 0267/93 | Neisseria meningitidis | X | No value | Carrier | Czech Republic | SV-5 | Opc+ |
| 1648 | 0268/93 | Neisseria meningitidis | NG | ST-92 complex | Carrier | Czech Republic | SV-1 | Opc+ |
| 1649 | 0269/93 | Neisseria meningitidis | NG | ST-53 complex | Carrier | Czech Republic | SV-2 | Opc- |
| 1650 | 0270/93 | Neisseria meningitidis | C | ST-11 complex | Carrier | Czech Republic | SV-2 | Opc- |

| | | | | | | | | |
|---|---|---|---|---|---|---|---|---|
| 1651 | 0271/93 | Neisseria meningitidis | NG | No value | Carrier | Czech Republic | SV-10 | Opc+ |
| 1652 | 0272/93 | Neisseria meningitidis | C | ST-11 complex | Carrier | Czech Republic | SV-2 | Opc- |
| 1653 | 0276/93 | Neisseria meningitidis | NG | ST-92 complex | Carrier | Czech Republic | SV-1 | Opc+ |
| 1654 | 0277/93 | Neisseria meningitidis | NG | ST-53 complex | Carrier | Czech Republic | SV-2 | Opc- |
| 1655 | 0279/93 | Neisseria meningitidis | B | ST-231 complex | Carrier | Czech Republic | SV-10 | Opc- |
| 1656 | 0280/93 | Neisseria meningitidis | B | ST-41/44 complex | Carrier | Czech Republic | SV-10 | Opc+ |
| 1658 | 0295/93 | Neisseria meningitidis | NG | ST-92 complex | Carrier | Czech Republic | SV-1 | Opc+ |
| 1659 | 0297/93 | Neisseria meningitidis | C | No value | Carrier | Czech Republic | SV-5 | Opc+ |
| 1884 | 0184/93 | Neisseria meningitidis | B | No value | No value | Czech Republic | SV-10 | Opc+ |
| 1885 | 0298/93 | Neisseria meningitidis | B | No value | Carrier | Czech Republic | SV-10 | Opc+ |
| 1893 | 0003/93 | Neisseria meningitidis | C | No value | No value | Czech Republic | SV-5 | Opc+ |
| 1905 | 0300/93 | Neisseria meningitidis | NG | No value | Carrier | Czech Republic | SV-8 | Opc- |
| 1919 | 0517/93 | Neisseria meningitidis | B | No value | No value | Czech Republic | SV-10 | Opc- |
| 1924 | 0301/93 | Neisseria meningitidis | NG | ST-549 complex | Carrier | Czech Republic | SV-5 | Opc+ |
| 1933 | 0007/93 | Neisseria meningitidis | B | No value | No value | Czech Republic | SV-5 | Opc- |
| 1935 | 0350/93 | Neisseria meningitidis | B | ST-18 complex | No value | Czech Republic | SV-7 | Opc- |
| 1936 | 0302/93 | Neisseria meningitidis | NG | ST-254 complex | Carrier | Czech Republic | SV-1 | Opc+ |
| 1941 | 0304/93 | Neisseria meningitidis | C | No value | Carrier | Czech Republic | SV-5 | Opc+ |
| 1947 | 0050/93 | Neisseria meningitidis | B | No value | No value | Czech Republic | SV-7 | Opc+ |
| 1948 | 0305/93 | Neisseria meningitidis | B | No value | Carrier | Czech Republic | SV-10 | Opc- |
| 1949 | 0306/93 | Neisseria meningitidis | B | ST-22 complex | Carrier | Czech Republic | SV-12 | Opc+ |
| 1955 | 0011/93 | Neisseria meningitidis | B | ST-41/44 complex | No value | Czech Republic | SV-10 | Opc+ |
| 1957 | 0497/93 | Neisseria meningitidis | B | No value | No value | Czech Republic | SV-10 | Opc- |
| 1958 | 0307/93 | Neisseria meningitidis | NG | ST-41/44 complex | Carrier | Czech Republic | SV-10 | Opc+ |
| 2077 | 153 | Neisseria meningitidis | E | ST-60 complex | Endemic | Germany | SV-5 | Opc+ |
| 2212 | 0502/93 | Neisseria meningitidis | B | ST-292 complex | No value | Czech Republic | SV-12 | Opc+ |
| 2214 | 0192/93 | Neisseria meningitidis | B | No value | No value | Czech Republic | SV-11 | Opc- |
| 2237 | 0005/93 | Neisseria meningitidis | B | No value | No value | Czech Republic | SV-5 | Opc- |
| 5171 | 0085/00 | Neisseria meningitidis | B | ST-11 complex | No value | Czech Republic | SV-2 | Opc- |
| 8139 | 0320/93 | Neisseria meningitidis | B | ST-41/44 complex | Carrier | Czech Republic | SV-10 | Opc+ |
| 8144 | 0325/93 | Neisseria meningitidis | B | ST-41/44 complex | Carrier | Czech Republic | SV-5 | Opc- |
| 8150 | 0336/93 | Neisseria meningitidis | C | No value | Carrier | Czech Republic | SV-5 | Opc+ |
| 8156 | 0342/93 | Neisseria meningitidis | B | ST-41/44 complex | Carrier | Czech Republic | SV-5 | Opc- |
| 8164 | 0404/93 | Neisseria meningitidis | B | ST-41/44 complex | Carrier | Czech Republic | SV-5 | Opc- |
| 8168 | 0409/93 | Neisseria meningitidis | NG | ST-41/44 complex | Carrier | Czech Republic | SV-10 | Opc+ |
| 8171 | 0412/93 | Neisseria meningitidis | NG | ST-41/44 complex | Carrier | Czech Republic | SV-5 | Opc- |
| 8173 | 0414/93 | Neisseria meningitidis | B | ST-41/44 complex | Carrier | Czech Republic | SV-5 | Opc- |
| 8176 | 0421/93 | Neisseria meningitidis | NG | ST-41/44 complex | Carrier | Czech Republic | SV-5 | Opc- |
| 9756 | 275 | Neisseria meningitidis | W | ST-22 complex | Endemic | Germany | SV-12 | Opc+ |
| 12672 | 53442 | Neisseria meningitidis | C | ST-4821 complex | No value | China | SV-2 | Opc+ |
| 13066 | 2789 | Neisseria meningitidis | NG | ST-35 complex | Carrier | Finland | SV-7 | Opc+ |
| 14480 | M5178 | Neisseria meningitidis | B | ST-32 complex | No value | USA | SV-1 | Opc+ |
| 14627 | M20918 | Neisseria meningitidis | A | ST-5 complex | No value | USA | SV-5 | Opc+ |
| 15024 | 0181/93 | Neisseria meningitidis | C | No value | No value | Czech Republic | SV-5 | Opc+ |
| 15255 | 0390/93 | Neisseria meningitidis | C | No value | No value | Czech Republic | SV-5 | Opc+ |
| 15259 | 0397/93 | Neisseria meningitidis | NG | No value | Carrier | Czech Republic | SV-10 | Opc+ |
| 15308 | 0443/93 | Neisseria meningitidis | B | No value | No value | Czech Republic | SV-10 | Opc+ |
| 17881 | Z5463BC | Neisseria meningitidis | A | ST-4 complex | No value | France | SV-5 | Opc+ |
| 17882 | Z5463 | Neisseria meningitidis | A | ST-4 complex | No value | The Gambia | SV-5 | Opc+ |
| 17883 | Z5463PI | Neisseria meningitidis | A | ST-4 complex | No value | The Gambia | SV-5 | Opc+ |
| 18968 | M10 240474 | Neisseria meningitidis | B | ST-269 complex | No value | UK | SV-1 | Opc+ |
| 18969 | M10 240478 | Neisseria meningitidis | B | ST-41/44 complex | No value | UK | SV-2 | Opc+ |
| 19023 | M10 240473 | Neisseria meningitidis | B | ST-269 complex | No value | UK | SV-8 | Opc+ |
| 19024 | M10 240480 | Neisseria meningitidis | B | ST-41/44 complex | No value | UK | SV-2 | Opc+ |
| 19025 | M10 240481 | Neisseria meningitidis | B | No value | No value | UK | SV-8 | Opc+ |
| 19026 | M10 240482 | Neisseria meningitidis | B | ST-41/44 complex | No value | UK | SV-2 | Opc+ |
| 19027 | M10 240484 | Neisseria meningitidis | B | ST-41/44 complex | No value | UK | SV-10 | Opc+ |
| 19028 | M10 240485 | Neisseria meningitidis | B | ST-269 complex | No value | UK | SV-8 | Opc+ |
| 19029 | M10 240487 | Neisseria meningitidis | B | ST-269 complex | No value | UK | SV-1 | Opc+ |
| 19030 | M10 240489 | Neisseria meningitidis | B | ST-461 complex | No value | UK | SV-1 | Opc- |
| 19031 | M10 240490 | Neisseria meningitidis | B | ST-269 complex | No value | UK | SV-8 | Opc+ |
| 19032 | M10 240498 | Neisseria meningitidis | B | ST-41/44 complex | No value | UK | SV-2 | Opc+ |
| 19260 | WUE 2594 | Neisseria meningitidis | A | ST-5 complex | No value | Germany | SV-5 | Opc+ |
| 19261 | alpha710 | Neisseria meningitidis | NG | ST-41/44 complex | No value | Germany | SV-5 | Opc- |
| 19262 | ATCC 13091 | Neisseria meningitidis | B | No value | No value | USA | SV-2 | Opc- |
| 19263 | NZ-05/33 | Neisseria meningitidis | NG | ST-41/44 complex | No value | New Zealand | SV-6 | Opc+ |
| 19264 | M04-240196 | Neisseria meningitidis | NG | ST-269 complex | No value | UK | SV-1 | Opc+ |
| 19265 | M01-240355 | Neisseria meningitidis | NG | ST-213 complex | No value | UK | SV-12 | Opc- |
| 19266 | M01-240149 | Neisseria meningitidis | NG | ST-41/44 complex | No value | UK | SV-2 | Opc+ |
| 19357 | N1568 | Neisseria meningitidis | NG | ST-181 complex | No value | Unknown | SV-5 | Opc+ |
| 19358 | M6190 | Neisseria meningitidis | NG | ST-11 complex | No value | Unknown | SV-2 | Opc- |
| 19359 | M13399 | Neisseria meningitidis | NG | ST-269 complex | No value | Unknown | SV-1 | Opc+ |
| 19360 | M0579 | Neisseria meningitidis | NG | ST-41/44 complex | No value | USA | SV-5 | Opc+ |
| 19361 | ES14902 | Neisseria meningitidis | NG | ST-11 complex | No value | Unknown | SV-5 | Opc- |

| | | | | | | | | |
|---|---|---|---|---|---|---|---|---|
| 19362 | CU385 | Neisseria meningitidis | NG | ST-32 complex | No value | Unknown | SV-2 | Opc+ |
| 19363 | 961-5945 | Neisseria meningitidis | NG | ST-8 complex | No value | Unknown | SV-10 | Opc- |
| 19364 | M01-240013 | Neisseria meningitidis | NG | No value | No value | Unknown | SV-1 | Opc+ |
| 19365 | K1207 | Neisseria meningitidis | C | ST-11 complex | Epidemic | Italy | SV-12 | Opc- |
| 19366 | M10699 | Neisseria meningitidis | B | ST-32 complex | No value | USA | SV-2 | Opc+ |
| 19367 | M11791 | Neisseria meningitidis | Y | ST-23 complex | No value | USA | SV-1 | Opc+ |
| 19368 | M13220 | Neisseria meningitidis | A | ST-5 complex | No value | Philippines | SV-5 | Opc+ |
| 19369 | M13519 | Neisseria meningitidis | W | ST-11 complex | No value | USA | SV-2 | Opc- |
| 19370 | M14900 | Neisseria meningitidis | Y | ST-23 complex | No value | USA | SV-11 | Opc+ |
| 19371 | M15141 | Neisseria meningitidis | C | ST-11 complex | No value | USA | SV-2 | Opc- |
| 19372 | M15293 | Neisseria meningitidis | NG | ST-32 complex | No value | USA | SV-1 | Opc+ |
| 19373 | M16207 | Neisseria meningitidis | B | ST-162 complex | No value | USA | SV-1 | Opc+ |
| 19374 | M16917 | Neisseria meningitidis | NG | ST-11 complex | No value | USA | SV-2 | Opc- |
| 19377 | M17661 | Neisseria meningitidis | W | ST-11 complex | No value | USA | SV-2 | Opc- |
| 19378 | M18575 | Neisseria meningitidis | A | ST-5 complex | No value | Burkina Faso | SV-5 | Opc+ |
| 19379 | M18774 | Neisseria meningitidis | W | ST-11 complex | No value | USA | SV-2 | Opc- |
| 19380 | M20899 | Neisseria meningitidis | Y | ST-167 complex | No value | USA | SV-5 | Opc+ |
| 19381 | NM9261 | Neisseria meningitidis | W | ST-11 complex | No value | Burkina Faso | SV-2 | Opc- |
| 19505 | S0108 | Neisseria meningitidis | C | ST-11 complex | Sporadic case | Italy | SV-2 | Opc- |
| 19957 | M00 241317 | Neisseria meningitidis | W | ST-11 complex | No value | UK | SV-2 | Opc- |
| 19958 | M10 240476 | Neisseria meningitidis | B | ST-213 complex | No value | UK | SV-12 | Opc- |
| 19959 | M10 240499 | Neisseria meningitidis | B | ST-269 complex | No value | UK | SV-8 | Opc+ |
| 19960 | M10 240500 | Neisseria meningitidis | B | ST-41/44 complex | No value | UK | SV-6 | Opc+ |
| 19961 | M10 240502 | Neisseria meningitidis | B | ST-41/44 complex | No value | UK | SV-6 | Opc+ |
| 19962 | M10 240503 | Neisseria meningitidis | Y | ST-23 complex | No value | UK | SV-11 | Opc+ |
| 19963 | M10 240505 | Neisseria meningitidis | Y | ST-23 complex | No value | UK | SV-11 | Opc+ |
| 19964 | M10 240507 | Neisseria meningitidis | Y | ST-23 complex | No value | UK | SV-11 | Opc+ |
| 19965 | M10 240508 | Neisseria meningitidis | B | No value | No value | UK | SV-12 | Opc+ |
| 19966 | M10 240511 | Neisseria meningitidis | B | No value | No value | UK | SV-8 | Opc+ |
| 19967 | M10 240512 | Neisseria meningitidis | B | ST-41/44 complex | No value | UK | SV-2 | Opc+ |
| 19968 | M10 240514 | Neisseria meningitidis | W | ST-11 complex | No value | UK | SV-2 | Opc- |
| 19969 | M10 240515 | Neisseria meningitidis | B | ST-269 complex | No value | UK | SV-1 | Opc+ |
| 19970 | M10 240520 | Neisseria meningitidis | Y | ST-23 complex | No value | UK | SV-11 | Opc+ |
| 19971 | M10 240521 | Neisseria meningitidis | B | ST-41/44 complex | No value | UK | SV-6 | Opc+ |
| 19972 | M10 240522 | Neisseria meningitidis | B | ST-41/44 complex | No value | UK | SV-2 | Opc+ |
| 19973 | M10 240525 | Neisseria meningitidis | B | ST-32 complex | No value | UK | SV-1 | Opc+ |
| 19974 | M10 240527 | Neisseria meningitidis | B | ST-269 complex | No value | UK | SV-1 | Opc+ |
| 19975 | M10 240528 | Neisseria meningitidis | B | ST-269 complex | No value | UK | SV-8 | Opc+ |
| 19976 | M10 240530 | Neisseria meningitidis | Y | ST-23 complex | No value | UK | SV-11 | Opc+ |
| 19977 | M10 240531 | Neisseria meningitidis | Y | ST-23 complex | No value | UK | SV-11 | Opc+ |
| 19978 | M10 240532 | Neisseria meningitidis | B | ST-41/44 complex | No value | UK | SV-2 | Opc+ |
| 19979 | M10 240534 | Neisseria meningitidis | Y | ST-23 complex | No value | UK | SV-11 | Opc+ |
| 19980 | M10 240536 | Neisseria meningitidis | Y | ST-23 complex | No value | UK | SV-11 | Opc+ |
| 19981 | M10 240540 | Neisseria meningitidis | Y | ST-23 complex | No value | UK | SV-11 | Opc+ |
| 19982 | M10 240546 | Neisseria meningitidis | B | ST-213 complex | No value | UK | SV-12 | Opc- |
| 19983 | M10 240547 | Neisseria meningitidis | B | ST-32 complex | No value | UK | SV-1 | Opc+ |
| 19984 | M10 240548 | Neisseria meningitidis | B | ST-41/44 complex | No value | UK | SV-6 | Opc+ |
| 19985 | M10 240549 | Neisseria meningitidis | B | ST-41/44 complex | No value | UK | SV-2 | Opc+ |
| 19986 | M10 240550 | Neisseria meningitidis | B | ST-269 complex | No value | UK | SV-8 | Opc+ |
| 19987 | M10 240553 | Neisseria meningitidis | B | ST-269 complex | No value | UK | SV-8 | Opc+ |
| 19988 | M10 240556 | Neisseria meningitidis | B | ST-41/44 complex | No value | UK | SV-6 | Opc+ |
| 19989 | M10 240558 | Neisseria meningitidis | B | ST-41/44 complex | No value | UK | SV-6 | Opc+ |
| 19990 | M10 240559 | Neisseria meningitidis | B | ST-162 complex | No value | UK | SV-1 | Opc+ |
| 19991 | M10 240566 | Neisseria meningitidis | B | ST-213 complex | No value | UK | SV-12 | Opc- |
| 19992 | M10 240568 | Neisseria meningitidis | B | ST-213 complex | No value | UK | SV-12 | Opc- |
| 19993 | M10 240569 | Neisseria meningitidis | B | ST-18 complex | No value | UK | SV-7 | Opc- |
| 19994 | M10 240570 | Neisseria meningitidis | B | ST-41/44 complex | No value | UK | SV-5 | Opc+ |
| 19995 | M10 240572 | Neisseria meningitidis | B | ST-269 complex | No value | UK | SV-8 | Opc+ |
| 19996 | M10 240578 | Neisseria meningitidis | B | ST-32 complex | No value | UK | SV-1 | Opc+ |
| 19997 | M10 240579 | Neisseria meningitidis | B | No value | No value | UK | SV-10 | Opc+ |
| 19998 | M10 240580 | Neisseria meningitidis | Y | ST-23 complex | No value | UK | SV-10 | Opc+ |
| 19999 | M10 240581 | Neisseria meningitidis | NG | ST-1136 complex | No value | UK | SV-2 | Opc+ |
| 20000 | M10 240582 | Neisseria meningitidis | B | ST-32 complex | No value | UK | SV-1 | Opc+ |
| 20001 | M10 240583 | Neisseria meningitidis | B | No value | No value | UK | SV-10 | Opc+ |
| 20002 | M10 240585 | Neisseria meningitidis | B | ST-41/44 complex | No value | UK | SV-2 | Opc+ |
| 20003 | M10 240586 | Neisseria meningitidis | B | ST-41/44 complex | No value | UK | SV-2 | Opc+ |
| 20004 | M10 240587 | Neisseria meningitidis | B | ST-41/44 complex | No value | UK | SV-5 | Opc- |
| 20005 | M10 240589 | Neisseria meningitidis | B | ST-41/44 complex | No value | UK | SV-6 | Opc+ |
| 20006 | M10 240590 | Neisseria meningitidis | Y | ST-23 complex | No value | UK | SV-11 | Opc+ |
| 20007 | M10 240591 | Neisseria meningitidis | B | ST-461 complex | No value | UK | SV-1 | Opc- |
| 20008 | M10 240592 | Neisseria meningitidis | B | ST-41/44 complex | No value | UK | SV-6 | Opc+ |
| 20009 | M10 240595 | Neisseria meningitidis | B | ST-213 complex | No value | UK | SV-12 | Opc- |
| 20010 | M10 240597 | Neisseria meningitidis | B | ST-41/44 complex | No value | UK | SV-2 | Opc+ |
| 20011 | M10 240598 | Neisseria meningitidis | B | ST-41/44 complex | No value | UK | SV-6 | Opc+ |

| | | | | | | | | |
|---|---|---|---|---|---|---|---|---|
| 20012 | M10 240602 | Neisseria meningitidis | B | ST-269 complex | No value | UK | SV-8 | Opc+ |
| 20013 | M10 240604 | Neisseria meningitidis | B | ST-41/44 complex | No value | UK | SV-2 | Opc+ |
| 20014 | M10 240605 | Neisseria meningitidis | B | ST-41/44 complex | No value | UK | SV-8 | Opc+ |
| 20015 | M10 240606 | Neisseria meningitidis | B | ST-213 complex | No value | UK | SV-12 | Opc- |
| 20016 | M10 240607 | Neisseria meningitidis | C | ST-103 complex | No value | UK | SV-5 | Opc+ |
| 20017 | M10 240611 | Neisseria meningitidis | B | ST-32 complex | No value | UK | SV-1 | Opc+ |
| 20018 | M10 240612 | Neisseria meningitidis | B | ST-269 complex | No value | UK | SV-1 | Opc+ |
| 20019 | M10 240613 | Neisseria meningitidis | B | ST-269 complex | No value | UK | SV-8 | Opc+ |
| 20020 | M10 240614 | Neisseria meningitidis | B | No value | No value | UK | SV-7 | Opc- |
| 20021 | M10 240616 | Neisseria meningitidis | W | ST-22 complex | No value | UK | SV-12 | Opc+ |
| 20022 | M10 240617 | Neisseria meningitidis | B | ST-213 complex | No value | UK | SV-12 | Opc- |
| 20023 | M10 240618 | Neisseria meningitidis | E | ST-60 complex | No value | UK | SV-11 | Opc+ |
| 20024 | M10 240619 | Neisseria meningitidis | B | ST-213 complex | No value | UK | SV-12 | Opc- |
| 20025 | M10 240621 | Neisseria meningitidis | B | ST-213 complex | No value | UK | SV-12 | Opc- |
| 20026 | M10 240622 | Neisseria meningitidis | B | No value | No value | UK | SV-8 | Opc+ |
| 20027 | M10 240623 | Neisseria meningitidis | B | ST-269 complex | No value | UK | SV-8 | Opc+ |
| 20028 | M10 240624 | Neisseria meningitidis | B | ST-269 complex | No value | UK | SV-8 | Opc+ |
| 20029 | M10 240626 | Neisseria meningitidis | B | ST-269 complex | No value | UK | SV-8 | Opc+ |
| 20030 | M10 240627 | Neisseria meningitidis | B | ST-32 complex | No value | UK | SV-1 | Opc+ |
| 20031 | M10 240631 | Neisseria meningitidis | C | ST-269 complex | No value | UK | SV-1 | Opc+ |
| 20032 | M10 240632 | Neisseria meningitidis | Y | ST-23 complex | No value | UK | SV-14 | Opc+ |
| 20033 | M10 240633 | Neisseria meningitidis | Y | ST-23 complex | No value | UK | SV-14 | Opc+ |
| 20034 | M10 240634 | Neisseria meningitidis | B | ST-41/44 complex | No value | UK | SV-6 | Opc+ |
| 20035 | M10 240636 | Neisseria meningitidis | B | ST-1157 complex | No value | UK | SV-1 | Opc+ |
| 20036 | M10 240638 | Neisseria meningitidis | B | ST-269 complex | No value | UK | SV-8 | Opc+ |
| 20037 | M10 240639 | Neisseria meningitidis | B | No value | No value | UK | SV-10 | Opc+ |
| 20038 | M10 240640 | Neisseria meningitidis | B | No value | No value | UK | SV-8 | Opc+ |
| 20039 | M10 240641 | Neisseria meningitidis | W | ST-22 complex | No value | UK | SV-12 | Opc+ |
| 20040 | M10 240642 | Neisseria meningitidis | B | No value | No value | UK | SV-8 | Opc+ |
| 20041 | M10 240643 | Neisseria meningitidis | Y | ST-23 complex | No value | UK | SV-11 | Opc+ |
| 20042 | M10 240644 | Neisseria meningitidis | Y | ST-22 complex | No value | UK | SV-11 | Opc+ |
| 20043 | M10 240645 | Neisseria meningitidis | B | ST-269 complex | No value | UK | SV-1 | Opc+ |
| 20044 | M10 240649 | Neisseria meningitidis | B | ST-269 complex | No value | UK | SV-8 | Opc+ |
| 20045 | M10 240650 | Neisseria meningitidis | B | No value | No value | UK | SV-5 | Opc- |
| 20046 | M10 240651 | Neisseria meningitidis | B | ST-213 complex | No value | UK | SV-12 | Opc- |
| 20047 | M10 240652 | Neisseria meningitidis | B | ST-269 complex | No value | UK | SV-8 | Opc+ |
| 20048 | M10 240657 | Neisseria meningitidis | B | ST-35 complex | No value | UK | SV-12 | Opc+ |
| 20049 | M10 240659 | Neisseria meningitidis | B | ST-41/44 complex | No value | UK | SV-6 | Opc+ |
| 20050 | M10 240661 | Neisseria meningitidis | B | ST-269 complex | No value | UK | SV-8 | Opc+ |
| 20051 | M10 240662 | Neisseria meningitidis | B | ST-41/44 complex | No value | UK | SV-2 | Opc+ |
| 20052 | M10 240665 | Neisseria meningitidis | B | ST-213 complex | No value | UK | SV-12 | Opc- |
| 20053 | M10 240666 | Neisseria meningitidis | W | ST-174 complex | No value | UK | SV-1 | Opc+ |
| 20054 | M10 240668 | Neisseria meningitidis | X | No value | No value | UK | SV-2 | Opc+ |
| 20055 | M10 240669 | Neisseria meningitidis | B | ST-41/44 complex | No value | UK | SV-6 | Opc+ |
| 20056 | M10 240670 | Neisseria meningitidis | B | ST-269 complex | No value | UK | SV-1 | Opc+ |
| 20057 | M10 240671 | Neisseria meningitidis | W | ST-11 complex | No value | UK | SV-2 | Opc- |
| 20058 | M10 240674 | Neisseria meningitidis | B | ST-41/44 complex | No value | UK | SV-2 | Opc+ |
| 20059 | M10 240675 | Neisseria meningitidis | Y | ST-167 complex | No value | UK | SV-5 | Opc+ |
| 20060 | M10 240676 | Neisseria meningitidis | B | No value | No value | UK | SV-7 | Opc+ |
| 20061 | M10 240677 | Neisseria meningitidis | B | No value | No value | UK | SV-12 | Opc+ |
| 20062 | M10 240678 | Neisseria meningitidis | B | ST-41/44 complex | No value | UK | SV-6 | Opc+ |
| 20063 | M10 240681 | Neisseria meningitidis | B | ST-41/44 complex | No value | UK | SV-6 | Opc+ |
| 20064 | M10 240682 | Neisseria meningitidis | B | ST-269 complex | No value | UK | SV-1 | Opc+ |
| 20065 | M10 240683 | Neisseria meningitidis | B | ST-18 complex | No value | UK | SV-7 | Opc- |
| 20066 | M10 240684 | Neisseria meningitidis | B | ST-11 complex | No value | UK | SV-2 | Opc- |
| 20067 | M10 240685 | Neisseria meningitidis | Y | ST-23 complex | No value | UK | SV-11 | Opc+ |
| 20068 | M10 240687 | Neisseria meningitidis | B | ST-60 complex | No value | UK | SV-11 | Opc+ |
| 20069 | M10 240689 | Neisseria meningitidis | B | ST-41/44 complex | No value | UK | SV-2 | Opc+ |
| 20070 | M10 240690 | Neisseria meningitidis | B | ST-41/44 complex | No value | UK | SV-2 | Opc+ |
| 20071 | M10 240693 | Neisseria meningitidis | B | ST-60 complex | No value | UK | SV-11 | Opc+ |
| 20072 | M10 240694 | Neisseria meningitidis | Y | ST-174 complex | No value | UK | SV-1 | Opc+ |
| 20073 | M10 240698 | Neisseria meningitidis | B | ST-282 complex | No value | UK | SV-9 | Opc+ |
| 20074 | M10 240700 | Neisseria meningitidis | Y | ST-23 complex | No value | UK | SV-11 | Opc+ |
| 20075 | M10 240701 | Neisseria meningitidis | B | ST-213 complex | No value | UK | SV-12 | Opc- |
| 20076 | M10 240702 | Neisseria meningitidis | B | ST-41/44 complex | No value | UK | SV-2 | Opc+ |
| 20077 | M10 240703 | Neisseria meningitidis | B | ST-213 complex | No value | UK | SV-12 | Opc- |
| 20078 | M10 240704 | Neisseria meningitidis | B | ST-269 complex | No value | UK | SV-1 | Opc+ |
| 20079 | M10 240705 | Neisseria meningitidis | C | ST-103 complex | No value | UK | SV-5 | Opc+ |
| 20080 | M10 240707 | Neisseria meningitidis | B | ST-41/44 complex | No value | UK | SV-2 | Opc+ |
| 20081 | M10 240708 | Neisseria meningitidis | B | ST-269 complex | No value | UK | SV-1 | Opc+ |
| 20082 | M10 240709 | Neisseria meningitidis | B | ST-269 complex | No value | UK | SV-1 | Opc+ |
| 20083 | M10 240711 | Neisseria meningitidis | B | ST-269 complex | No value | UK | SV-8 | Opc+ |
| 20084 | M10 240712 | Neisseria meningitidis | B | ST-213 complex | No value | UK | SV-12 | Opc- |
| 20085 | M10 240713 | Neisseria meningitidis | B | ST-269 complex | No value | UK | SV-8 | Opc+ |

| | | | | | | | | |
|---|---|---|---|---|---|---|---|---|
| 20086 | M10 240716 | Neisseria meningitidis | B | ST-461 complex | No value | UK | SV-1 | Opc- |
| 20087 | M10 240717 | Neisseria meningitidis | B | ST-213 complex | No value | UK | SV-12 | Opc- |
| 20088 | M10 240718 | Neisseria meningitidis | B | ST-60 complex | No value | UK | SV-14 | Opc+ |
| 20089 | M10 240719 | Neisseria meningitidis | B | ST-32 complex | No value | UK | SV-1 | Opc+ |
| 20090 | M10 240720 | Neisseria meningitidis | B | ST-269 complex | No value | UK | SV-1 | Opc+ |
| 20091 | M10 240721 | Neisseria meningitidis | B | ST-41/44 complex | No value | UK | SV-6 | Opc+ |
| 20092 | M10 240722 | Neisseria meningitidis | B | ST-41/44 complex | No value | UK | SV-6 | Opc+ |
| 20093 | M10 240723 | Neisseria meningitidis | B | ST-41/44 complex | No value | UK | SV-5 | Opc+ |
| 20094 | M10 240724 | Neisseria meningitidis | B | ST-41/44 complex | No value | UK | SV-2 | Opc+ |
| 20095 | M10 240728 | Neisseria meningitidis | B | ST-269 complex | No value | UK | SV-1 | Opc+ |
| 20096 | M10 240729 | Neisseria meningitidis | B | ST-269 complex | No value | UK | SV-1 | Opc+ |
| 20097 | M10 240730 | Neisseria meningitidis | B | ST-41/44 complex | No value | UK | SV-12 | Opc+ |
| 20098 | M10 240731 | Neisseria meningitidis | B | No value | No value | UK | SV-8 | Opc+ |
| 20099 | M10 240732 | Neisseria meningitidis | Y | ST-23 complex | No value | UK | SV-14 | Opc+ |
| 20100 | M10 240733 | Neisseria meningitidis | B | No value | No value | UK | SV-8 | Opc+ |
| 20101 | M10 240737 | Neisseria meningitidis | W | ST-22 complex | No value | UK | SV-12 | Opc+ |
| 20102 | M10 240742 | Neisseria meningitidis | B | ST-213 complex | No value | UK | SV-1 | Opc- |
| 20103 | M10 240743 | Neisseria meningitidis | B | ST-269 complex | No value | UK | SV-1 | Opc+ |
| 20104 | M10 240745 | Neisseria meningitidis | Y | ST-23 complex | No value | UK | SV-11 | Opc+ |
| 20105 | M10 240746 | Neisseria meningitidis | B | ST-41/44 complex | No value | UK | SV-2 | Opc+ |
| 20106 | M10 240747 | Neisseria meningitidis | B | ST-41/44 complex | No value | UK | SV-10 | Opc+ |
| 20107 | M10 240748 | Neisseria meningitidis | B | ST-41/44 complex | No value | UK | SV-2 | Opc+ |
| 20108 | M10 240749 | Neisseria meningitidis | B | ST-41/44 complex | No value | UK | SV-2 | Opc+ |
| 20109 | M10 240750 | Neisseria meningitidis | B | ST-41/44 complex | No value | UK | SV-7 | Opc+ |
| 20110 | M10 240751 | Neisseria meningitidis | B | ST-213 complex | No value | UK | SV-12 | Opc- |
| 20111 | M10 240752 | Neisseria meningitidis | B | ST-269 complex | No value | UK | SV-8 | Opc+ |
| 20112 | M10 240753 | Neisseria meningitidis | B | ST-32 complex | No value | UK | SV-1 | Opc+ |
| 20113 | M10 240754 | Neisseria meningitidis | B | ST-41/44 complex | No value | UK | SV-2 | Opc+ |
| 20114 | M10 240756 | Neisseria meningitidis | W | ST-22 complex | No value | UK | SV-12 | Opc+ |
| 20115 | M10 240759 | Neisseria meningitidis | Y | ST-174 complex | No value | UK | SV-1 | Opc+ |
| 20116 | M10 240761 | Neisseria meningitidis | B | ST-41/44 complex | No value | UK | SV-2 | Opc+ |
| 20117 | M10 240762 | Neisseria meningitidis | B | ST-41/44 complex | No value | UK | SV-2 | Opc+ |
| 20118 | M10 240763 | Neisseria meningitidis | B | No value | No value | UK | SV-5 | Opc+ |
| 20119 | M10 240766 | Neisseria meningitidis | B | ST-269 complex | No value | UK | SV-1 | Opc+ |
| 20120 | M10 240767 | Neisseria meningitidis | B | ST-32 complex | No value | UK | SV-1 | Opc+ |
| 20121 | M10 240768 | Neisseria meningitidis | B | ST-269 complex | No value | UK | SV-1 | Opc+ |
| 20122 | M10 240770 | Neisseria meningitidis | B | ST-269 complex | No value | UK | SV-1 | Opc+ |
| 20123 | M10 240771 | Neisseria meningitidis | B | ST-282 complex | No value | UK | SV-9 | Opc+ |
| 20124 | M10 240772 | Neisseria meningitidis | B | ST-269 complex | No value | UK | SV-8 | Opc+ |
| 20125 | M10 240773 | Neisseria meningitidis | B | ST-41/44 complex | No value | UK | SV-2 | Opc+ |
| 20126 | M10 240774 | Neisseria meningitidis | B | ST-269 complex | No value | UK | SV-8 | Opc+ |
| 20127 | M10 240775 | Neisseria meningitidis | B | ST-41/44 complex | No value | UK | SV-2 | Opc+ |
| 20128 | M10 240776 | Neisseria meningitidis | Y | ST-23 complex | No value | UK | SV-11 | Opc+ |
| 20129 | M10 240777 | Neisseria meningitidis | Y | ST-23 complex | No value | UK | SV-5 | Opc+ |
| 20130 | M10 240778 | Neisseria meningitidis | B | ST-213 complex | No value | UK | SV-12 | Opc- |
| 20131 | M10 240779 | Neisseria meningitidis | B | ST-269 complex | No value | UK | SV-1 | Opc+ |
| 20132 | M10 240781 | Neisseria meningitidis | Y | ST-22 complex | No value | UK | SV-12 | Opc+ |
| 20133 | M10 240783 | Neisseria meningitidis | B | ST-269 complex | No value | UK | SV-12 | Opc+ |
| 20134 | M10 240785 | Neisseria meningitidis | B | ST-41/44 complex | No value | UK | SV-6 | Opc+ |
| 20135 | M10 240786 | Neisseria meningitidis | Y | ST-23 complex | No value | UK | SV-14 | Opc+ |
| 20136 | M10 240787 | Neisseria meningitidis | Y | ST-23 complex | No value | UK | SV-14 | Opc+ |
| 20137 | M10 240788 | Neisseria meningitidis | B | ST-41/44 complex | No value | UK | SV-2 | Opc+ |
| 20138 | M10 240789 | Neisseria meningitidis | B | ST-269 complex | No value | UK | SV-1 | Opc+ |
| 20139 | M10 240790 | Neisseria meningitidis | B | ST-32 complex | No value | UK | SV-1 | Opc+ |
| 20140 | M10 240793 | Neisseria meningitidis | B | ST-41/44 complex | No value | UK | SV-6 | Opc+ |
| 20141 | M10 240794 | Neisseria meningitidis | B | ST-32 complex | No value | UK | SV-5 | Opc+ |
| 20142 | M10 240796 | Neisseria meningitidis | B | ST-41/44 complex | No value | UK | SV-14 | Opc- |
| 20143 | M10 240798 | Neisseria meningitidis | Y | ST-23 complex | No value | UK | SV-12 | Opc+ |
| 20144 | M10 240799 | Neisseria meningitidis | B | ST-213 complex | No value | UK | SV-2 | Opc- |
| 20145 | M10 240803 | Neisseria meningitidis | B | ST-41/44 complex | No value | UK | SV-2 | Opc+ |
| 20146 | M10 240804 | Neisseria meningitidis | Y | ST-23 complex | No value | UK | SV-11 | Opc+ |
| 20147 | M10 240805 | Neisseria meningitidis | B | No value | No value | UK | SV-8 | Opc+ |
| 20148 | M10 240807 | Neisseria meningitidis | B | ST-41/44 complex | No value | UK | SV-2 | Opc+ |
| 20149 | M10 240808 | Neisseria meningitidis | B | ST-269 complex | No value | UK | SV-1 | Opc+ |
| 20150 | M10 240809 | Neisseria meningitidis | B | ST-41/44 complex | No value | UK | SV-2 | Opc+ |
| 20151 | M10 240810 | Neisseria meningitidis | B | ST-162 complex | No value | UK | SV-1 | Opc+ |
| 20152 | M10 240811 | Neisseria meningitidis | B | ST-1157 complex | No value | UK | SV-1 | Opc+ |
| 20153 | M10 240814 | Neisseria meningitidis | B | ST-269 complex | No value | UK | SV-8 | Opc+ |
| 20154 | M10 240817 | Neisseria meningitidis | W | ST-11 complex | No value | UK | SV-2 | Opc- |
| 20155 | M10 240818 | Neisseria meningitidis | C | ST-11 complex | No value | UK | SV-2 | Opc- |
| 20156 | M10 240819 | Neisseria meningitidis | B | ST-269 complex | No value | UK | SV-8 | Opc+ |
| 20157 | M10 240820 | Neisseria meningitidis | B | ST-41/44 complex | No value | UK | SV-2 | Opc+ |
| 20158 | M10 240821 | Neisseria meningitidis | W | ST-11 complex | No value | UK | SV-2 | Opc- |
| 20159 | M10 240822 | Neisseria meningitidis | W | ST-22 complex | No value | UK | SV-12 | Opc+ |

| | | | | | | | | |
|---|---|---|---|---|---|---|---|---|
| 20160 | M10 240823 | Neisseria meningitidis | B | ST-41/44 complex | No value | UK | SV-10 | Opc+ |
| 20161 | M10 240824 | Neisseria meningitidis | B | ST-32 complex | No value | UK | SV-1 | Opc+ |
| 20162 | M10 240825 | Neisseria meningitidis | B | ST-60 complex | No value | UK | SV-11 | Opc+ |
| 20163 | M10 240826 | Neisseria meningitidis | B | ST-269 complex | No value | UK | SV-8 | Opc+ |
| 20164 | M11 240000 | Neisseria meningitidis | Y | ST-23 complex | No value | UK | SV-11 | Opc+ |
| 20165 | M11 240001 | Neisseria meningitidis | B | ST-461 complex | No value | UK | SV-1 | Opc- |
| 20166 | M11 240002 | Neisseria meningitidis | B | No value | No value | UK | SV-1 | Opc- |
| 20167 | M11 240003 | Neisseria meningitidis | B | ST-41/44 complex | No value | UK | SV-10 | Opc+ |
| 20168 | M11 240004 | Neisseria meningitidis | B | ST-269 complex | No value | UK | SV-8 | Opc+ |
| 20169 | M11 240005 | Neisseria meningitidis | Y | ST-23 complex | No value | UK | SV-10 | Opc+ |
| 20170 | M11 240006 | Neisseria meningitidis | B | ST-32 complex | No value | UK | SV-3 | Opc+ |
| 20171 | M11 240007 | Neisseria meningitidis | Y | ST-23 complex | No value | UK | SV-11 | Opc+ |
| 20172 | M11 240008 | Neisseria meningitidis | B | ST-41/44 complex | No value | UK | SV-1 | Opc+ |
| 20173 | M11 240010 | Neisseria meningitidis | B | ST-269 complex | No value | UK | SV-1 | Opc+ |
| 20174 | M11 240011 | Neisseria meningitidis | B | ST-41/44 complex | No value | UK | SV-6 | Opc+ |
| 20175 | M11 240012 | Neisseria meningitidis | B | ST-41/44 complex | No value | UK | SV-2 | Opc+ |
| 20176 | M11 240013 | Neisseria meningitidis | B | ST-32 complex | No value | UK | SV-1 | Opc+ |
| 20177 | M11 240014 | Neisseria meningitidis | B | ST-461 complex | No value | UK | SV-2 | Opc- |
| 20178 | M11 240015 | Neisseria meningitidis | B | No value | No value | UK | SV-1 | Opc+ |
| 20179 | M11 240016 | Neisseria meningitidis | B | ST-32 complex | No value | UK | SV-1 | Opc+ |
| 20180 | M11 240017 | Neisseria meningitidis | B | ST-41/44 complex | No value | UK | SV-6 | Opc+ |
| 20181 | M11 240018 | Neisseria meningitidis | B | ST-41/44 complex | No value | UK | SV-2 | Opc+ |
| 20182 | M11 240019 | Neisseria meningitidis | B | ST-35 complex | No value | UK | SV-1 | Opc+ |
| 20183 | M11 240021 | Neisseria meningitidis | B | ST-32 complex | No value | UK | SV-1 | Opc+ |
| 20184 | M11 240022 | Neisseria meningitidis | B | ST-269 complex | No value | UK | SV-8 | Opc+ |
| 20185 | M11 240023 | Neisseria meningitidis | B | ST-41/44 complex | No value | UK | SV-2 | Opc+ |
| 20186 | M11 240024 | Neisseria meningitidis | B | ST-41/44 complex | No value | UK | SV-6 | Opc+ |
| 20187 | M11 240025 | Neisseria meningitidis | B | ST-269 complex | No value | UK | SV-1 | Opc+ |
| 20188 | M11 240026 | Neisseria meningitidis | B | ST-269 complex | No value | UK | SV-1 | Opc+ |
| 20189 | M11 240027 | Neisseria meningitidis | B | ST-60 complex | No value | UK | SV-2 | Opc+ |
| 20190 | M11 240028 | Neisseria meningitidis | B | ST-60 complex | No value | UK | SV-11 | Opc+ |
| 20191 | M11 240029 | Neisseria meningitidis | W | ST-22 complex | No value | UK | SV-12 | Opc+ |
| 20192 | M11 240030 | Neisseria meningitidis | B | ST-269 complex | No value | UK | SV-1 | Opc+ |
| 20193 | M11 240031 | Neisseria meningitidis | Y | ST-23 complex | No value | UK | SV-11 | Opc+ |
| 20194 | M11 240032 | Neisseria meningitidis | B | No value | No value | UK | SV-8 | Opc+ |
| 20195 | M11 240034 | Neisseria meningitidis | B | ST-41/44 complex | No value | UK | SV-6 | Opc+ |
| 20196 | M11 240035 | Neisseria meningitidis | W | ST-11 complex | No value | UK | SV-2 | Opc- |
| 20197 | M11 240036 | Neisseria meningitidis | Y | ST-23 complex | No value | UK | SV-11 | Opc+ |
| 20198 | M11 240037 | Neisseria meningitidis | B | ST-32 complex | No value | UK | SV-1 | Opc+ |
| 20199 | M11 240038 | Neisseria meningitidis | Y | ST-167 complex | No value | UK | SV-5 | Opc+ |
| 20200 | M11 240039 | Neisseria meningitidis | B | ST-282 complex | No value | UK | SV-9 | Opc+ |
| 20201 | M11 240040 | Neisseria meningitidis | B | ST-41/44 complex | No value | UK | SV-2 | Opc+ |
| 20202 | M11 240041 | Neisseria meningitidis | B | ST-41/44 complex | No value | UK | SV-2 | Opc+ |
| 20203 | M11 240042 | Neisseria meningitidis | B | ST-461 complex | No value | UK | SV-1 | Opc- |
| 20204 | M11 240043 | Neisseria meningitidis | Y | ST-23 complex | No value | UK | SV-11 | Opc+ |
| 20205 | M11 240044 | Neisseria meningitidis | B | ST-269 complex | No value | UK | SV-1 | Opc+ |
| 20206 | M11 240045 | Neisseria meningitidis | B | ST-18 complex | No value | UK | SV-7 | Opc- |
| 20207 | M11 240046 | Neisseria meningitidis | B | ST-213 complex | No value | UK | SV-12 | Opc- |
| 20208 | M11 240047 | Neisseria meningitidis | B | ST-41/44 complex | No value | UK | SV-2 | Opc+ |
| 20209 | M11 240048 | Neisseria meningitidis | B | ST-60 complex | No value | UK | SV-11 | Opc+ |
| 20210 | M11 240050 | Neisseria meningitidis | B | ST-213 complex | No value | UK | SV-12 | Opc- |
| 20211 | M11 240052 | Neisseria meningitidis | B | ST-461 complex | No value | UK | SV-1 | Opc- |
| 20212 | M11 240053 | Neisseria meningitidis | B | ST-41/44 complex | No value | UK | SV-6 | Opc+ |
| 20213 | M11 240054 | Neisseria meningitidis | B | ST-41/44 complex | No value | UK | SV-2 | Opc+ |
| 20214 | M11 240055 | Neisseria meningitidis | B | ST-41/44 complex | No value | UK | SV-2 | Opc+ |
| 20215 | M11 240056 | Neisseria meningitidis | B | ST-11 complex | No value | UK | SV-2 | Opc- |
| 20216 | M11 240057 | Neisseria meningitidis | W | ST-11 complex | No value | UK | SV-2 | Opc- |
| 20217 | M11 240058 | Neisseria meningitidis | B | ST-269 complex | No value | UK | SV-8 | Opc+ |
| 20218 | M11 240059 | Neisseria meningitidis | B | ST-41/44 complex | No value | UK | SV-2 | Opc+ |
| 20219 | M11 240060 | Neisseria meningitidis | B | ST-35 complex | No value | UK | SV-12 | Opc+ |
| 20220 | M11 240061 | Neisseria meningitidis | B | ST-213 complex | No value | UK | SV-12 | Opc- |
| 20221 | M11 240062 | Neisseria meningitidis | B | ST-269 complex | No value | UK | SV-8 | Opc+ |
| 20222 | M11 240063 | Neisseria meningitidis | B | ST-41/44 complex | No value | UK | SV-2 | Opc+ |
| 20223 | M11 240064 | Neisseria meningitidis | Y | No value | No value | UK | SV-5 | Opc+ |
| 20224 | M11 240065 | Neisseria meningitidis | Y | ST-23 complex | No value | UK | SV-11 | Opc+ |
| 20225 | M11 240066 | Neisseria meningitidis | B | ST-269 complex | No value | UK | SV-8 | Opc+ |
| 20226 | M11 240067 | Neisseria meningitidis | W | ST-11 complex | No value | UK | SV-2 | Opc- |
| 20227 | M11 240068 | Neisseria meningitidis | NG | No value | No value | UK | SV-12 | Opc+ |
| 20228 | M11 240069 | Neisseria meningitidis | B | ST-35 complex | No value | UK | SV-12 | Opc+ |
| 20229 | M11 240070 | Neisseria meningitidis | B | ST-269 complex | No value | UK | SV-1 | Opc+ |
| 20230 | M11 240071 | Neisseria meningitidis | Y | ST-167 complex | No value | UK | SV-5 | Opc+ |
| 20231 | M11 240072 | Neisseria meningitidis | B | ST-269 complex | No value | UK | SV-8 | Opc+ |
| 20232 | M11 240073 | Neisseria meningitidis | Y | ST-174 complex | No value | UK | SV-1 | Opc+ |
| 20233 | M11 240074 | Neisseria meningitidis | B | ST-213 complex | No value | UK | SV-12 | Opc- |

| | | | | | | | | |
|---|---|---|---|---|---|---|---|---|
| 20234 | M11 240076 | Neisseria meningitidis | C | ST-11 complex | No value | UK | SV-2 | Opc- |
| 20235 | M11 240077 | Neisseria meningitidis | B | ST-269 complex | No value | UK | SV-1 | Opc+ |
| 20236 | M11 240079 | Neisseria meningitidis | B | ST-41/44 complex | No value | UK | SV-2 | Opc+ |
| 20237 | M11 240081 | Neisseria meningitidis | B | ST-41/44 complex | No value | UK | SV-2 | Opc+ |
| 20238 | M11 240082 | Neisseria meningitidis | B | ST-32 complex | No value | UK | SV-1 | Opc+ |
| 20239 | M11 240083 | Neisseria meningitidis | B | ST-461 complex | No value | UK | SV-1 | Opc- |
| 20240 | M11 240084 | Neisseria meningitidis | B | ST-18 complex | No value | UK | SV-7 | Opc- |
| 20241 | M11 240085 | Neisseria meningitidis | B | ST-41/44 complex | No value | UK | SV-6 | Opc+ |
| 20242 | M11 240086 | Neisseria meningitidis | B | ST-269 complex | No value | UK | SV-1 | Opc+ |
| 20243 | M11 240088 | Neisseria meningitidis | B | ST-41/44 complex | No value | UK | SV-2 | Opc+ |
| 20244 | M11 240094 | Neisseria meningitidis | C | ST-103 complex | No value | UK | SV-5 | Opc+ |
| 20245 | M11 240096 | Neisseria meningitidis | Y | ST-23 complex | No value | UK | SV-11 | Opc+ |
| 20246 | M11 240097 | Neisseria meningitidis | B | ST-41/44 complex | No value | UK | SV-2 | Opc+ |
| 20247 | M11 240099 | Neisseria meningitidis | W | ST-11 complex | No value | UK | SV-2 | Opc- |
| 20248 | M11 240100 | Neisseria meningitidis | B | ST-103 complex | No value | UK | SV-5 | Opc+ |
| 20249 | M11 240108 | Neisseria meningitidis | B | ST-41/44 complex | No value | UK | SV-6 | Opc+ |
| 20250 | M11 240109 | Neisseria meningitidis | B | ST-32 complex | No value | UK | SV-1 | Opc+ |
| 20251 | M11 240110 | Neisseria meningitidis | B | ST-41/44 complex | No value | UK | SV-2 | Opc+ |
| 20252 | M11 240111 | Neisseria meningitidis | B | ST-213 complex | No value | UK | SV-12 | Opc- |
| 20253 | M11 240112 | Neisseria meningitidis | B | ST-41/44 complex | No value | UK | SV-6 | Opc+ |
| 20254 | M11 240113 | Neisseria meningitidis | B | ST-213 complex | No value | UK | SV-12 | Opc- |
| 20255 | M11 240115 | Neisseria meningitidis | B | ST-41/44 complex | No value | UK | SV-2 | Opc+ |
| 20256 | M11 240116 | Neisseria meningitidis | Y | ST-23 complex | No value | UK | SV-11 | Opc+ |
| 20257 | M11 240117 | Neisseria meningitidis | B | ST-60 complex | No value | UK | SV-11 | Opc+ |
| 20258 | M11 240119 | Neisseria meningitidis | B | ST-41/44 complex | No value | UK | SV-10 | Opc+ |
| 20259 | M11 240121 | Neisseria meningitidis | B | ST-213 complex | No value | UK | SV-12 | Opc- |
| 20260 | M11 240122 | Neisseria meningitidis | B | No value | No value | UK | SV-8 | Opc+ |
| 20261 | M11 240123 | Neisseria meningitidis | B | ST-11 complex | No value | UK | SV-2 | Opc- |
| 20263 | M11 240125 | Neisseria meningitidis | B | ST-41/44 complex | No value | UK | SV-2 | Opc+ |
| 20264 | M11 240126 | Neisseria meningitidis | Y | ST-23 complex | No value | UK | SV-11 | Opc+ |
| 20265 | M11 240128 | Neisseria meningitidis | B | No value | No value | UK | SV-8 | Opc+ |
| 20266 | M11 240129 | Neisseria meningitidis | B | ST-282 complex | No value | UK | SV-9 | Opc+ |
| 20267 | M11 240131 | Neisseria meningitidis | B | ST-162 complex | No value | UK | SV-1 | Opc+ |
| 20268 | M11 240132 | Neisseria meningitidis | B | ST-269 complex | No value | UK | SV-1 | Opc+ |
| 20269 | M11 240133 | Neisseria meningitidis | B | ST-18 complex | No value | UK | SV-7 | Opc- |
| 20270 | M11 240134 | Neisseria meningitidis | B | ST-213 complex | No value | UK | SV-12 | Opc- |
| 20271 | M11 240137 | Neisseria meningitidis | B | ST-213 complex | No value | UK | SV-12 | Opc- |
| 20272 | M11 240139 | Neisseria meningitidis | B | ST-269 complex | No value | UK | SV-8 | Opc+ |
| 20273 | M11 240145 | Neisseria meningitidis | B | ST-41/44 complex | No value | UK | SV-2 | Opc+ |
| 20274 | M11 240146 | Neisseria meningitidis | B | ST-269 complex | No value | UK | SV-8 | Opc+ |
| 20275 | M11 240147 | Neisseria meningitidis | B | ST-213 complex | No value | UK | SV-12 | Opc- |
| 20276 | M11 240148 | Neisseria meningitidis | B | ST-41/44 complex | No value | UK | SV-2 | Opc+ |
| 20277 | M11 240149 | Neisseria meningitidis | B | ST-41/44 complex | No value | UK | SV-2 | Opc+ |
| 20278 | M11 240150 | Neisseria meningitidis | B | ST-269 complex | No value | UK | SV-1 | Opc+ |
| 20279 | M11 240151 | Neisseria meningitidis | B | ST-269 complex | No value | UK | SV-1 | Opc+ |
| 20280 | M11 240152 | Neisseria meningitidis | B | ST-41/44 complex | No value | UK | SV-6 | Opc+ |
| 20281 | M11 240157 | Neisseria meningitidis | Y | ST-23 complex | No value | UK | SV-11 | Opc+ |
| 20282 | M11 240161 | Neisseria meningitidis | Y | ST-174 complex | No value | UK | SV-1 | Opc+ |
| 20283 | M11 240163 | Neisseria meningitidis | B | ST-41/44 complex | No value | UK | SV-2 | Opc+ |
| 20284 | M11 240164 | Neisseria meningitidis | B | ST-41/44 complex | No value | UK | SV-6 | Opc+ |
| 20285 | M11 240165 | Neisseria meningitidis | Y | ST-174 complex | No value | UK | SV-1 | Opc+ |
| 20286 | M11 240166 | Neisseria meningitidis | Y | ST-23 complex | No value | UK | SV-11 | Opc+ |
| 20287 | M11 240167 | Neisseria meningitidis | B | ST-60 complex | No value | UK | SV-11 | Opc+ |
| 20288 | M11 240168 | Neisseria meningitidis | W | ST-11 complex | No value | UK | SV-2 | Opc- |
| 20289 | M11 240170 | Neisseria meningitidis | B | ST-41/44 complex | No value | UK | SV-6 | Opc+ |
| 20290 | M11 240173 | Neisseria meningitidis | B | ST-41/44 complex | No value | UK | SV-2 | Opc+ |
| 20291 | M11 240174 | Neisseria meningitidis | B | ST-35 complex | No value | UK | SV-12 | Opc+ |
| 20292 | M11 240175 | Neisseria meningitidis | B | ST-32 complex | No value | UK | SV-1 | Opc+ |
| 20293 | M11 240176 | Neisseria meningitidis | B | No value | No value | UK | SV-11 | Opc+ |
| 20294 | M11 240180 | Neisseria meningitidis | B | ST-269 complex | No value | UK | SV-1 | Opc+ |
| 20295 | M11 240181 | Neisseria meningitidis | B | ST-213 complex | No value | UK | SV-12 | Opc- |
| 20296 | M11 240182 | Neisseria meningitidis | B | No value | No value | UK | SV-1 | Opc+ |
| 20297 | M11 240183 | Neisseria meningitidis | B | ST-32 complex | No value | UK | SV-1 | Opc+ |
| 20298 | M11 240185 | Neisseria meningitidis | B | ST-269 complex | No value | UK | SV-1 | Opc+ |
| 20299 | M11 240188 | Neisseria meningitidis | B | ST-41/44 complex | No value | UK | SV-2 | Opc+ |
| 20300 | M11 240189 | Neisseria meningitidis | B | ST-282 complex | No value | UK | SV-9 | Opc+ |
| 20301 | M11 240191 | Neisseria meningitidis | B | ST-41/44 complex | No value | UK | SV-2 | Opc+ |
| 20302 | M11 240192 | Neisseria meningitidis | Y | ST-23 complex | No value | UK | SV-11 | Opc+ |
| 20303 | M11 240193 | Neisseria meningitidis | B | ST-41/44 complex | No value | UK | SV-10 | Opc+ |
| 20304 | M11 240195 | Neisseria meningitidis | B | ST-41/44 complex | No value | UK | SV-6 | Opc+ |
| 20305 | M11 240203 | Neisseria meningitidis | B | ST-41/44 complex | No value | UK | SV-6 | Opc+ |
| 20306 | M11 240206 | Neisseria meningitidis | B | ST-461 complex | No value | UK | SV-1 | Opc- |
| 20307 | M11 240207 | Neisseria meningitidis | C | ST-41/44 complex | No value | UK | SV-10 | Opc+ |
| 20308 | M11 240209 | Neisseria meningitidis | Y | ST-174 complex | No value | UK | SV-1 | Opc+ |

| | | | | | | | | |
|---|---|---|---|---|---|---|---|---|
| 20309 | M11 240210 | Neisseria meningitidis | Y | ST-23 complex | No value | UK | SV-11 | Opc+ |
| 20310 | M11 240211 | Neisseria meningitidis | Y | ST-174 complex | No value | UK | SV-1 | Opc+ |
| 20311 | M11 240212 | Neisseria meningitidis | B | ST-269 complex | No value | UK | SV-8 | Opc+ |
| 20312 | M11 240213 | Neisseria meningitidis | B | ST-41/44 complex | No value | UK | SV-2 | Opc+ |
| 20313 | M11 240214 | Neisseria meningitidis | B | ST-461 complex | No value | UK | SV-1 | Opc- |
| 20314 | M11 240215 | Neisseria meningitidis | Y | ST-23 complex | No value | UK | SV-11 | Opc+ |
| 20315 | M11 240216 | Neisseria meningitidis | Y | ST-23 complex | No value | UK | SV-11 | Opc+ |
| 20316 | M11 240226 | Neisseria meningitidis | B | ST-269 complex | No value | UK | SV-1 | Opc+ |
| 20317 | M11 240227 | Neisseria meningitidis | Y | ST-23 complex | No value | UK | SV-11 | Opc+ |
| 20318 | M11 240231 | Neisseria meningitidis | W | ST-22 complex | No value | UK | SV-12 | Opc+ |
| 20319 | M11 240232 | Neisseria meningitidis | B | ST-35 complex | No value | UK | SV-12 | Opc+ |
| 20320 | M11 240233 | Neisseria meningitidis | NG | ST-226 complex | No value | UK | SV-10 | Opc+ |
| 20321 | M11 240234 | Neisseria meningitidis | C | No value | No value | UK | SV-7 | Opc+ |
| 20322 | M11 240236 | Neisseria meningitidis | B | ST-269 complex | No value | UK | SV-8 | Opc+ |
| 20323 | M11 240237 | Neisseria meningitidis | B | No value | No value | UK | SV-10 | Opc+ |
| 20324 | M11 240241 | Neisseria meningitidis | B | ST-41/44 complex | No value | UK | SV-2 | Opc+ |
| 20325 | M11 240242 | Neisseria meningitidis | B | ST-41/44 complex | No value | UK | SV-2 | Opc+ |
| 20326 | M11 240243 | Neisseria meningitidis | B | No value | No value | UK | SV-8 | Opc+ |
| 20327 | M11 240244 | Neisseria meningitidis | B | ST-41/44 complex | No value | UK | SV-6 | Opc+ |
| 20328 | M11 240246 | Neisseria meningitidis | NG | ST-60 complex | No value | UK | SV-11 | Opc+ |
| 20329 | M11 240247 | Neisseria meningitidis | C | ST-11 complex | No value | UK | SV-2 | Opc- |
| 20330 | M11 240248 | Neisseria meningitidis | B | ST-41/44 complex | No value | UK | SV-2 | Opc+ |
| 20331 | M11 240249 | Neisseria meningitidis | B | No value | No value | UK | SV-8 | Opc- |
| 20332 | M11 240251 | Neisseria meningitidis | B | ST-269 complex | No value | UK | SV-1 | Opc+ |
| 20333 | M11 240255 | Neisseria meningitidis | B | ST-41/44 complex | No value | UK | SV-2 | Opc+ |
| 20334 | M11 240256 | Neisseria meningitidis | B | ST-41/44 complex | No value | UK | SV-2 | Opc+ |
| 20335 | M11 240258 | Neisseria meningitidis | Y | ST-23 complex | No value | UK | SV-11 | Opc+ |
| 20336 | M11 240259 | Neisseria meningitidis | B | ST-41/44 complex | No value | UK | SV-2 | Opc+ |
| 20337 | M11 240261 | Neisseria meningitidis | W | ST-22 complex | No value | UK | SV-12 | Opc+ |
| 20338 | M11 240262 | Neisseria meningitidis | A | ST-5 complex | No value | UK | SV-5 | Opc+ |
| 20339 | M11 240263 | Neisseria meningitidis | Y | ST-23 complex | No value | UK | SV-11 | Opc+ |
| 20340 | M11 240265 | Neisseria meningitidis | B | ST-269 complex | No value | UK | SV-1 | Opc+ |
| 20341 | M11 240266 | Neisseria meningitidis | B | ST-213 complex | No value | UK | SV-12 | Opc- |
| 20342 | M11 240268 | Neisseria meningitidis | B | ST-213 complex | No value | UK | SV-2 | Opc- |
| 20343 | M11 240269 | Neisseria meningitidis | B | ST-41/44 complex | No value | UK | SV-1 | Opc+ |
| 20344 | M11 240275 | Neisseria meningitidis | B | ST-269 complex | No value | UK | SV-2 | Opc+ |
| 20345 | M11 240276 | Neisseria meningitidis | B | ST-41/44 complex | No value | UK | SV-11 | Opc+ |
| 20346 | M11 240277 | Neisseria meningitidis | Y | ST-23 complex | No value | UK | SV-11 | Opc+ |
| 20347 | M11 240278 | Neisseria meningitidis | Y | ST-23 complex | No value | UK | SV-11 | Opc+ |
| 20348 | M11 240280 | Neisseria meningitidis | B | ST-35 complex | No value | UK | SV-16 | Opc+ |
| 20349 | M11 240281 | Neisseria meningitidis | B | ST-269 complex | No value | UK | SV-8 | Opc+ |
| 20350 | M11 240282 | Neisseria meningitidis | B | ST-213 complex | No value | UK | SV-12 | Opc- |
| 20351 | M11 240283 | Neisseria meningitidis | Y | ST-23 complex | No value | UK | SV-11 | Opc+ |
| 20352 | M11 240284 | Neisseria meningitidis | B | ST-269 complex | No value | UK | SV-8 | Opc+ |
| 20353 | M11 240285 | Neisseria meningitidis | B | ST-32 complex | No value | UK | SV-1 | Opc+ |
| 20354 | M11 240286 | Neisseria meningitidis | B | No value | No value | UK | SV-5 | Opc+ |
| 20356 | M11 240289 | Neisseria meningitidis | B | ST-41/44 complex | No value | UK | SV-6 | Opc+ |
| 20357 | M11 240290 | Neisseria meningitidis | B | ST-213 complex | No value | UK | SV-12 | Opc- |
| 20358 | M11 240291 | Neisseria meningitidis | B | ST-41/44 complex | No value | UK | SV-2 | Opc+ |
| 20359 | M11 240294 | Neisseria meningitidis | C | ST-11 complex | No value | UK | SV-2 | Opc- |
| 20360 | M11 240295 | Neisseria meningitidis | B | ST-41/44 complex | No value | UK | SV-2 | Opc+ |
| 20361 | M11 240296 | Neisseria meningitidis | B | ST-32 complex | No value | UK | SV-1 | Opc+ |
| 20362 | M11 240297 | Neisseria meningitidis | B | ST-213 complex | No value | UK | SV-12 | Opc- |
| 20363 | M11 240298 | Neisseria meningitidis | Y | ST-23 complex | No value | UK | SV-11 | Opc+ |
| 20364 | M11 240299 | Neisseria meningitidis | B | ST-41/44 complex | No value | UK | SV-2 | Opc+ |
| 20365 | M11 240302 | Neisseria meningitidis | W | ST-22 complex | No value | UK | SV-12 | Opc+ |
| 20366 | M11 240303 | Neisseria meningitidis | B | ST-269 complex | No value | UK | SV-8 | Opc+ |
| 20367 | M11 240304 | Neisseria meningitidis | B | No value | No value | UK | SV-1 | Opc+ |
| 20368 | M11 240305 | Neisseria meningitidis | W | ST-11 complex | No value | UK | SV-2 | Opc- |
| 20369 | M11 240306 | Neisseria meningitidis | B | ST-41/44 complex | No value | UK | SV-2 | Opc+ |
| 20370 | M11 240309 | Neisseria meningitidis | B | No value | No value | UK | SV-8 | Opc+ |
| 20371 | M11 240311 | Neisseria meningitidis | B | ST-41/44 complex | No value | UK | SV-2 | Opc+ |
| 20372 | M11 240312 | Neisseria meningitidis | Y | ST-23 complex | No value | UK | SV-11 | Opc+ |
| 20373 | M11 240313 | Neisseria meningitidis | Y | ST-23 complex | No value | UK | SV-11 | Opc+ |
| 20374 | M11 240314 | Neisseria meningitidis | B | ST-269 complex | No value | UK | SV-8 | Opc+ |
| 20375 | M11 240315 | Neisseria meningitidis | B | ST-269 complex | No value | UK | SV-8 | Opc+ |
| 20376 | M11 240316 | Neisseria meningitidis | B | ST-269 complex | No value | UK | SV-1 | Opc+ |
| 20377 | M11 240317 | Neisseria meningitidis | B | ST-269 complex | No value | UK | SV-1 | Opc+ |
| 20378 | M11 240318 | Neisseria meningitidis | B | ST-269 complex | No value | UK | SV-8 | Opc+ |
| 20379 | M11 240319 | Neisseria meningitidis | Y | ST-23 complex | No value | UK | SV-11 | Opc+ |
| 20380 | M11 240322 | Neisseria meningitidis | Y | ST-23 complex | No value | UK | SV-11 | Opc+ |
| 20381 | M11 240323 | Neisseria meningitidis | B | No value | No value | UK | SV-11 | Opc+ |
| 20382 | M11 240324 | Neisseria meningitidis | C | ST-32 complex | No value | UK | SV-1 | Opc+ |
| 20383 | M11 240326 | Neisseria meningitidis | Y | ST-23 complex | No value | UK | SV-11 | Opc+ |

| | | | | | | | | |
|---|---|---|---|---|---|---|---|---|
| 20384 | M11 240328 | Neisseria meningitidis | B | ST-41/44 complex | No value | UK | SV-2 | Opc+ |
| 20385 | M11 240330 | Neisseria meningitidis | B | ST-269 complex | No value | UK | SV-8 | Opc+ |
| 20386 | M11 240333 | Neisseria meningitidis | B | ST-41/44 complex | No value | UK | SV-10 | Opc+ |
| 20387 | M11 240334 | Neisseria meningitidis | B | ST-269 complex | No value | UK | SV-8 | Opc+ |
| 20388 | M11 240335 | Neisseria meningitidis | B | ST-162 complex | No value | UK | SV-1 | Opc+ |
| 20389 | M11 240337 | Neisseria meningitidis | B | ST-213 complex | No value | UK | SV-12 | Opc- |
| 20390 | M11 240338 | Neisseria meningitidis | B | ST-1157 complex | No value | UK | SV-1 | Opc+ |
| 20391 | M11 240339 | Neisseria meningitidis | B | ST-60 complex | No value | UK | SV-5 | Opc+ |
| 20392 | M11 240341 | Neisseria meningitidis | B | ST-41/44 complex | No value | UK | SV-2 | Opc+ |
| 20393 | M11 240342 | Neisseria meningitidis | B | ST-32 complex | No value | UK | SV-1 | Opc+ |
| 20394 | M11 240343 | Neisseria meningitidis | B | ST-41/44 complex | No value | UK | SV-2 | Opc+ |
| 20395 | M11 240344 | Neisseria meningitidis | B | ST-213 complex | No value | UK | SV-17 | Opc- |
| 20396 | M11 240346 | Neisseria meningitidis | Y | ST-167 complex | No value | UK | SV-5 | Opc+ |
| 20397 | M11 240347 | Neisseria meningitidis | C | ST-103 complex | No value | UK | SV-5 | Opc+ |
| 20398 | M11 240348 | Neisseria meningitidis | B | ST-213 complex | No value | UK | SV-12 | Opc- |
| 20399 | M11 240349 | Neisseria meningitidis | W | ST-22 complex | No value | UK | SV-12 | Opc+ |
| 20400 | M11 240350 | Neisseria meningitidis | B | ST-41/44 complex | No value | UK | SV-6 | Opc+ |
| 20401 | M11 240352 | Neisseria meningitidis | B | ST-269 complex | No value | UK | SV-8 | Opc+ |
| 20402 | M11 240353 | Neisseria meningitidis | B | ST-269 complex | No value | UK | SV-8 | Opc+ |
| 20403 | M11 240356 | Neisseria meningitidis | B | ST-32 complex | No value | UK | SV-1 | Opc+ |
| 20404 | M11 240358 | Neisseria meningitidis | W | ST-23 complex | No value | UK | SV-11 | Opc+ |
| 20405 | M11 240360 | Neisseria meningitidis | B | ST-41/44 complex | No value | UK | SV-6 | Opc+ |
| 20406 | M11 240363 | Neisseria meningitidis | C | No value | No value | UK | SV-5 | Opc+ |
| 20407 | M11 240364 | Neisseria meningitidis | B | ST-41/44 complex | No value | UK | SV-2 | Opc+ |
| 20408 | M11 240365 | Neisseria meningitidis | B | ST-41/44 complex | No value | UK | SV-2 | Opc+ |
| 20409 | M11 240366 | Neisseria meningitidis | B | ST-32 complex | No value | UK | SV-1 | Opc+ |
| 20410 | M11 240367 | Neisseria meningitidis | B | ST-41/44 complex | No value | UK | SV-2 | Opc+ |
| 20411 | M11 240368 | Neisseria meningitidis | B | ST-269 complex | No value | UK | SV-1 | Opc+ |
| 20412 | M11 240369 | Neisseria meningitidis | B | ST-41/44 complex | No value | UK | SV-2 | Opc+ |
| 20413 | M11 240371 | Neisseria meningitidis | B | ST-162 complex | No value | UK | SV-1 | Opc+ |
| 20414 | M11 240372 | Neisseria meningitidis | NG | ST-23 complex | No value | UK | SV-11 | Opc+ |
| 20415 | M11 240373 | Neisseria meningitidis | B | ST-41/44 complex | No value | UK | SV-2 | Opc+ |
| 20416 | M11 240375 | Neisseria meningitidis | B | ST-269 complex | No value | UK | SV-1 | Opc+ |
| 20417 | M11 240376 | Neisseria meningitidis | B | ST-269 complex | No value | UK | SV-1 | Opc+ |
| 20418 | M11 240381 | Neisseria meningitidis | B | ST-162 complex | No value | UK | SV-1 | Opc+ |
| 20419 | M11 240382 | Neisseria meningitidis | B | ST-41/44 complex | No value | UK | SV-2 | Opc+ |
| 20420 | M11 240383 | Neisseria meningitidis | B | ST-35 complex | No value | UK | SV-16 | Opc+ |
| 20421 | M11 240386 | Neisseria meningitidis | B | ST-41/44 complex | No value | UK | SV-1 | Opc+ |
| 20422 | M11 240387 | Neisseria meningitidis | W | ST-22 complex | No value | UK | SV-12 | Opc+ |
| 20423 | M11 240388 | Neisseria meningitidis | B | ST-269 complex | No value | UK | SV-1 | Opc+ |
| 20424 | M11 240389 | Neisseria meningitidis | W | ST-11 complex | No value | UK | SV-2 | Opc- |
| 20425 | M11 240390 | Neisseria meningitidis | B | No value | No value | UK | SV-1 | Opc+ |
| 20426 | M11 240391 | Neisseria meningitidis | B | ST-41/44 complex | No value | UK | SV-2 | Opc+ |
| 20427 | M11 240392 | Neisseria meningitidis | B | ST-32 complex | No value | UK | SV-1 | Opc+ |
| 20428 | M11 240394 | Neisseria meningitidis | B | ST-60 complex | No value | UK | SV-11 | Opc+ |
| 20429 | M11 240395 | Neisseria meningitidis | B | ST-269 complex | No value | UK | SV-8 | Opc+ |
| 20430 | M11 240396 | Neisseria meningitidis | Y | ST-23 complex | No value | UK | SV-11 | Opc+ |
| 20431 | M11 240397 | Neisseria meningitidis | B | ST-269 complex | No value | UK | SV-8 | Opc+ |
| 20432 | M11 240398 | Neisseria meningitidis | B | ST-41/44 complex | No value | UK | SV-2 | Opc+ |
| 20433 | M11 240399 | Neisseria meningitidis | Y | ST-23 complex | No value | UK | SV-11 | Opc+ |
| 20434 | M11 240401 | Neisseria meningitidis | B | ST-269 complex | No value | UK | SV-8 | Opc+ |
| 20435 | M11 240402 | Neisseria meningitidis | B | No value | No value | UK | SV-8 | Opc- |
| 20436 | M11 240403 | Neisseria meningitidis | W | ST-11 complex | No value | UK | SV-2 | Opc- |
| 20437 | M11 240405 | Neisseria meningitidis | B | ST-269 complex | No value | UK | SV-8 | Opc+ |
| 20438 | M11 240406 | Neisseria meningitidis | B | ST-32 complex | No value | UK | SV-1 | Opc+ |
| 20439 | M11 240409 | Neisseria meningitidis | B | ST-269 complex | No value | UK | SV-1 | Opc+ |
| 20440 | M11 240411 | Neisseria meningitidis | Y | ST-23 complex | No value | UK | SV-11 | Opc+ |
| 20441 | M11 240412 | Neisseria meningitidis | B | ST-60 complex | No value | UK | SV-11 | Opc+ |
| 20442 | M11 240413 | Neisseria meningitidis | B | ST-269 complex | No value | UK | SV-8 | Opc+ |
| 20443 | M11 240414 | Neisseria meningitidis | B | ST-41/44 complex | No value | UK | SV-6 | Opc+ |
| 20444 | M11 240417 | Neisseria meningitidis | W | ST-11 complex | No value | UK | SV-2 | Opc- |
| 20445 | M11 240420 | Neisseria meningitidis | B | ST-41/44 complex | No value | UK | SV-2 | Opc+ |
| 20446 | M11 240422 | Neisseria meningitidis | B | ST-269 complex | No value | UK | SV-1 | Opc+ |
| 20447 | M11 240424 | Neisseria meningitidis | B | ST-41/44 complex | No value | UK | SV-5 | Opc- |
| 20448 | M11 240425 | Neisseria meningitidis | B | ST-41/44 complex | No value | UK | SV-2 | Opc+ |
| 20449 | M11 240427 | Neisseria meningitidis | W | ST-11 complex | No value | UK | SV-2 | Opc- |
| 20450 | M11 240428 | Neisseria meningitidis | B | ST-269 complex | No value | UK | SV-8 | Opc+ |
| 20451 | M11 240430 | Neisseria meningitidis | Y | ST-23 complex | No value | UK | SV-11 | Opc+ |
| 20452 | M11 240431 | Neisseria meningitidis | B | No value | No value | UK | SV-8 | Opc+ |
| 20453 | M11 240434 | Neisseria meningitidis | B | ST-41/44 complex | No value | UK | SV-2 | Opc+ |
| 20454 | M11 240435 | Neisseria meningitidis | Y | ST-23 complex | No value | UK | SV-11 | Opc+ |
| 20455 | M11 240436 | Neisseria meningitidis | B | ST-269 complex | No value | UK | SV-8 | Opc+ |
| 20456 | M11 240437 | Neisseria meningitidis | Y | ST-92 complex | No value | UK | SV-1 | Opc+ |
| 20457 | M11 240440 | Neisseria meningitidis | B | ST-60 complex | No value | UK | SV-11 | Opc+ |

| | | | | | | | | |
|---|---|---|---|---|---|---|---|---|
| 20458 | M11 240441 | Neisseria meningitidis | W | ST-174 complex | No value | UK | SV-1 | Opc+ |
| 20459 | M11 240442 | Neisseria meningitidis | Y | ST-23 complex | No value | UK | SV-11 | Opc+ |
| 20460 | M12 240156 | Neisseria meningitidis | W | ST-11 complex | No value | UK | SV-2 | Opc- |
| 20461 | M12 240160 | Neisseria meningitidis | W | ST-11 complex | No value | UK | SV-2 | Opc- |
| 20462 | M12 240196 | Neisseria meningitidis | W | ST-11 complex | No value | UK | SV-2 | Opc- |
| 20470 | M11 240118 | Neisseria meningitidis | B | ST-41/44 complex | No value | UK | SV-2 | Opc+ |
| 20477 | H44/76 | Neisseria meningitidis | B | ST-32 complex | Epidemic | Norway | SV-1 | Opc+ |
| 20562 | Nm8187 | Neisseria meningitidis | Y | ST-167 complex | No value | Canada | SV-5 | Opc+ |
| 20563 | Nm3127 | Neisseria meningitidis | Y | ST-167 complex | No value | Canada | SV-5 | Opc+ |
| 20564 | Nm6938 | Neisseria meningitidis | W | ST-22 complex | No value | Canada | SV-12 | Opc+ |
| 20565 | Nm2732 | Neisseria meningitidis | W | ST-22 complex | No value | Canada | SV-12 | Opc+ |
| 20566 | Nm8663 | Neisseria meningitidis | Y | ST-23 complex | No value | Canada | SV-11 | Opc+ |
| 20567 | Nm6756 | Neisseria meningitidis | Y | ST-23 complex | No value | Canada | SV-11 | Opc+ |
| 20568 | Nm1140 | Neisseria meningitidis | NG | ST-1136 complex | No value | Canada | SV-2 | Opc+ |
| 20736 | M09 240001 | Neisseria meningitidis | NG | ST-269 complex | No value | UK | SV-1 | Opc+ |
| 20737 | M09 240034 | Neisseria meningitidis | NG | ST-269 complex | No value | UK | SV-8 | Opc+ |
| 20738 | M09 240039 | Neisseria meningitidis | NG | ST-41/44 complex | No value | UK | SV-2 | Opc+ |
| 20739 | M09 240042 | Neisseria meningitidis | NG | ST-35 complex | No value | UK | SV-12 | Opc+ |
| 20740 | M09 240044 | Neisseria meningitidis | NG | ST-32 complex | No value | UK | SV-1 | Opc+ |
| 20741 | M09 240064 | Neisseria meningitidis | NG | ST-60 complex | No value | UK | SV-11 | Opc+ |
| 20742 | M09 240078 | Neisseria meningitidis | NG | ST-41/44 complex | No value | UK | SV-2 | Opc+ |
| 20743 | M09 240092 | Neisseria meningitidis | NG | ST-269 complex | No value | UK | SV-8 | Opc+ |
| 20744 | M09 240102 | Neisseria meningitidis | NG | ST-269 complex | No value | UK | SV-1 | Opc+ |
| 20745 | M09 240111 | Neisseria meningitidis | NG | ST-269 complex | No value | UK | SV-8 | Opc+ |
| 20746 | M09 240119 | Neisseria meningitidis | NG | No value | No value | UK | SV-8 | Opc- |
| 20747 | M09 240135 | Neisseria meningitidis | NG | ST-32 complex | No value | UK | SV-1 | Opc+ |
| 20748 | M09 240139 | Neisseria meningitidis | NG | ST-41/44 complex | No value | UK | SV-2 | Opc+ |
| 20749 | M09 240147 | Neisseria meningitidis | NG | ST-269 complex | No value | UK | SV-1 | Opc+ |
| 20750 | M09 240162 | Neisseria meningitidis | NG | ST-269 complex | No value | UK | SV-8 | Opc+ |
| 20751 | M09 240184 | Neisseria meningitidis | NG | ST-269 complex | No value | UK | SV-8 | Opc+ |
| 20752 | M09 240237 | Neisseria meningitidis | NG | ST-269 complex | No value | UK | SV-1 | Opc+ |
| 20753 | M09 240242 | Neisseria meningitidis | NG | ST-461 complex | No value | UK | SV-1 | Opc- |
| 20754 | M09 240243 | Neisseria meningitidis | NG | ST-269 complex | No value | UK | SV-1 | Opc+ |
| 20755 | M09 240256 | Neisseria meningitidis | NG | ST-41/44 complex | No value | UK | SV-2 | Opc+ |
| 20756 | M09 240264 | Neisseria meningitidis | NG | ST-162 complex | No value | UK | SV-1 | Opc+ |
| 20757 | M09 240281 | Neisseria meningitidis | NG | ST-41/44 complex | No value | UK | SV-2 | Opc+ |
| 20758 | M09 240283 | Neisseria meningitidis | NG | ST-41/44 complex | No value | UK | SV-6 | Opc+ |
| 20759 | M09 240296 | Neisseria meningitidis | NG | ST-213 complex | No value | UK | SV-12 | Opc- |
| 20760 | M09 240330 | Neisseria meningitidis | NG | ST-41/44 complex | No value | UK | SV-2 | Opc+ |
| 20761 | M09 240355 | Neisseria meningitidis | NG | ST-269 complex | No value | UK | SV-1 | Opc+ |
| 20762 | M09 240439 | Neisseria meningitidis | NG | ST-41/44 complex | No value | UK | SV-2 | Opc+ |
| 20763 | M09 240440 | Neisseria meningitidis | NG | ST-213 complex | No value | UK | SV-12 | Opc- |
| 20764 | M09 240463 | Neisseria meningitidis | NG | ST-41/44 complex | No value | UK | SV-6 | Opc+ |
| 20765 | M09 240483 | Neisseria meningitidis | NG | ST-18 complex | No value | UK | SV-7 | Opc- |
| 20766 | M09 240506 | Neisseria meningitidis | NG | ST-41/44 complex | No value | UK | SV-10 | Opc+ |
| 20767 | M09 240527 | Neisseria meningitidis | NG | No value | No value | UK | SV-5 | Opc+ |
| 20768 | M09 240585 | Neisseria meningitidis | NG | ST-162 complex | No value | UK | SV-1 | Opc+ |
| 20769 | M09 240659 | Neisseria meningitidis | NG | ST-213 complex | No value | UK | SV-12 | Opc- |
| 20770 | M09 240669 | Neisseria meningitidis | NG | ST-41/44 complex | No value | UK | SV-2 | Opc+ |
| 20771 | M09 240681 | Neisseria meningitidis | NG | ST-213 complex | No value | UK | SV-12 | Opc- |
| 20772 | M09 240793 | Neisseria meningitidis | NG | ST-32 complex | No value | UK | SV-1 | Opc+ |
| 20773 | M09 240831 | Neisseria meningitidis | NG | ST-41/44 complex | No value | UK | SV-2 | Opc+ |
| 20774 | M09 240834 | Neisseria meningitidis | NG | ST-269 complex | No value | UK | SV-1 | Opc+ |
| 20775 | M09 240849 | Neisseria meningitidis | NG | ST-269 complex | No value | UK | SV-7 | Opc+ |
| 20776 | M09 240859 | Neisseria meningitidis | NG | No value | No value | UK | SV-1 | Opc+ |
| 20777 | M09 240872 | Neisseria meningitidis | NG | ST-1157 complex | No value | UK | SV-1 | Opc+ |
| 20778 | M09 240878 | Neisseria meningitidis | NG | ST-269 complex | No value | UK | SV-1 | Opc+ |
| 20779 | M10 240025 | Neisseria meningitidis | NG | ST-269 complex | No value | UK | SV-7 | Opc+ |
| 20780 | M10 240039 | Neisseria meningitidis | NG | ST-1157 complex | No value | UK | SV-1 | Opc+ |
| 20781 | M10 240050 | Neisseria meningitidis | NG | ST-41/44 complex | No value | UK | SV-2 | Opc+ |
| 20782 | M10 240052 | Neisseria meningitidis | NG | ST-41/44 complex | No value | UK | SV-2 | Opc+ |
| 20783 | M10 240064 | Neisseria meningitidis | NG | ST-269 complex | No value | UK | SV-8 | Opc+ |
| 20784 | M10 240074 | Neisseria meningitidis | NG | ST-269 complex | No value | UK | SV-8 | Opc+ |
| 20785 | M10 240080 | Neisseria meningitidis | NG | ST-41/44 complex | No value | UK | SV-2 | Opc+ |
| 20786 | M10 240091 | Neisseria meningitidis | NG | ST-162 complex | No value | UK | SV-1 | Opc+ |
| 20787 | M10 240109 | Neisseria meningitidis | NG | ST-269 complex | No value | UK | SV-8 | Opc+ |
| 20788 | M10 240115 | Neisseria meningitidis | NG | ST-41/44 complex | No value | UK | SV-6 | Opc+ |
| 20789 | M10 240118 | Neisseria meningitidis | NG | ST-32 complex | No value | UK | SV-1 | Opc+ |
| 20790 | M10 240127 | Neisseria meningitidis | NG | ST-60 complex | No value | UK | SV-11 | Opc+ |
| 20791 | M10 240128 | Neisseria meningitidis | NG | No value | No value | UK | SV-1 | Opc+ |
| 20792 | M10 240137 | Neisseria meningitidis | NG | ST-41/44 complex | No value | UK | SV-2 | Opc+ |
| 20793 | M10 240140 | Neisseria meningitidis | NG | ST-461 complex | No value | UK | SV-1 | Opc- |
| 20794 | M10 240146 | Neisseria meningitidis | NG | ST-162 complex | No value | UK | SV-1 | Opc+ |
| 20795 | M10 240168 | Neisseria meningitidis | NG | ST-41/44 complex | No value | UK | SV-6 | Opc+ |

| | | | | | | | | |
|---|---|---|---|---|---|---|---|---|
| 20796 | M10 240173 | Neisseria meningitidis | NG | ST-269 complex | No value | UK | SV-8 | Opc+ |
| 20797 | M10 240180 | Neisseria meningitidis | NG | No value | No value | UK | SV-12 | Opc- |
| 20798 | M10 240184 | Neisseria meningitidis | NG | ST-41/44 complex | No value | UK | SV-2 | Opc+ |
| 20799 | M10 240199 | Neisseria meningitidis | NG | No value | No value | UK | SV-12 | Opc+ |
| 20800 | M10 240219 | Neisseria meningitidis | NG | ST-41/44 complex | No value | UK | SV-6 | Opc+ |
| 20801 | M10 240238 | Neisseria meningitidis | NG | No value | No value | UK | SV-8 | Opc+ |
| 20802 | M10 240300 | Neisseria meningitidis | NG | ST-41/44 complex | No value | UK | SV-7 | Opc+ |
| 20803 | M12 240642 | Neisseria meningitidis | NG | ST-865 complex | No value | UK | SV-5 | Opc+ |
| 20804 | M09 240583 | Neisseria meningitidis | NG | ST-162 complex | No value | UK | SV-1 | Opc+ |
| 20805 | M09 240174 | Neisseria meningitidis | NG | ST-41/44 complex | No value | UK | SV-6 | Opc+ |
| 20806 | M09 240332 | Neisseria meningitidis | NG | ST-269 complex | No value | UK | SV-1 | Opc+ |
| 20807 | M09 240542 | Neisseria meningitidis | NG | ST-269 complex | No value | UK | SV-1 | Opc+ |
| 20808 | M09 240735 | Neisseria meningitidis | NG | ST-269 complex | No value | UK | SV-1 | Opc+ |
| 20809 | M10 240015 | Neisseria meningitidis | NG | ST-41/44 complex | No value | UK | SV-2 | Opc+ |
| 20810 | M10 240645 | Neisseria meningitidis | NG | ST-269 complex | No value | UK | SV-1 | Opc+ |
| 20881 | M00 240227 | Neisseria meningitidis | NG | ST-32 complex | No value | UK | SV-1 | Opc+ |
| 20882 | M00 240303 | Neisseria meningitidis | NG | ST-32 complex | No value | UK | SV-1 | Opc+ |
| 20883 | M00 240304 | Neisseria meningitidis | NG | ST-32 complex | No value | UK | SV-1 | Opc+ |
| 20884 | M00 240613 | Neisseria meningitidis | B | ST-41/44 complex | No value | UK | SV-2 | Opc+ |
| 20885 | M00 240614 | Neisseria meningitidis | NG | ST-41/44 complex | No value | UK | SV-2 | Opc+ |
| 20886 | M01 240245 | Neisseria meningitidis | NG | ST-41/44 complex | No value | UK | SV-6 | Opc+ |
| 20887 | M01 240258 | Neisseria meningitidis | B | ST-41/44 complex | No value | UK | SV-6 | Opc+ |
| 20888 | M01 240259 | Neisseria meningitidis | NG | ST-11 complex | No value | UK | SV-2 | Opc- |
| 20889 | M01 240260 | Neisseria meningitidis | NG | ST-11 complex | No value | UK | SV-2 | Opc- |
| 20890 | M01 240798 | Neisseria meningitidis | NG | ST-269 complex | No value | UK | SV-8 | Opc+ |
| 20891 | M01 240799 | Neisseria meningitidis | NG | ST-269 complex | No value | UK | SV-8 | Opc+ |
| 20892 | M02 240150 | Neisseria meningitidis | NG | ST-269 complex | No value | UK | SV-8 | Opc+ |
| 20893 | M02 240167 | Neisseria meningitidis | NG | ST-269 complex | No value | UK | SV-8 | Opc+ |
| 20894 | M02 240169 | Neisseria meningitidis | NG | ST-269 complex | No value | UK | SV-8 | Opc+ |
| 20895 | M03 241783 | Neisseria meningitidis | NG | ST-32 complex | No value | UK | SV-1 | Opc+ |
| 20896 | M03 241784 | Neisseria meningitidis | NG | ST-32 complex | No value | UK | SV-1 | Opc+ |
| 20899 | M05 240362 | Neisseria meningitidis | NG | ST-60 complex | No value | UK | SV-11 | Opc+ |
| 20900 | M05 240370 | Neisseria meningitidis | NG | ST-60 complex | No value | UK | SV-11 | Opc+ |
| 20901 | M05 241063 | Neisseria meningitidis | NG | ST-35 complex | No value | UK | SV-2 | Opc+ |
| 20902 | M05 241064 | Neisseria meningitidis | NG | ST-35 complex | No value | UK | SV-2 | Opc+ |
| 20903 | M05 241347 | Neisseria meningitidis | NG | ST-22 complex | No value | UK | SV-12 | Opc+ |
| 20904 | M05 241348 | Neisseria meningitidis | NG | ST-22 complex | No value | UK | SV-12 | Opc+ |
| 20905 | M99 240945 | Neisseria meningitidis | B | ST-41/44 complex | No value | UK | SV-2 | Opc+ |
| 20906 | M99 240975 | Neisseria meningitidis | NG | ST-41/44 complex | No value | UK | SV-2 | Opc+ |
| 20907 | M99 241794 | Neisseria meningitidis | B | ST-41/44 complex | No value | Ireland | SV-2 | Opc+ |
| 20908 | M99 241795 | Neisseria meningitidis | NG | ST-41/44 complex | No value | Ireland | SV-2 | Opc+ |
| 20909 | M03 241538 | Neisseria meningitidis | NG | ST-269 complex | No value | UK | SV-1 | Opc+ |
| 20910 | M04 241352 | Neisseria meningitidis | NG | No value | No value | UK | SV-18 | Opc+ |
| 20911 | M05 240240 | Neisseria meningitidis | B | ST-41/44 complex | No value | UK | SV-14 | Opc+ |
| 20912 | M05 241259 | Neisseria meningitidis | NG | ST-213 complex | No value | UK | SV-12 | Opc- |
| 20913 | M01 240285 | Neisseria meningitidis | NG | ST-35 complex | No value | UK | SV-12 | Opc+ |
| 20914 | M01 240286 | Neisseria meningitidis | NG | ST-35 complex | No value | UK | SV-12 | Opc+ |
| 20915 | M01 241920 | Neisseria meningitidis | B | ST-41/44 complex | No value | UK | SV-6 | Opc+ |
| 20916 | M01 241930 | Neisseria meningitidis | B | ST-41/44 complex | No value | UK | SV-6 | Opc+ |
| 20917 | M03 240455 | Neisseria meningitidis | NG | ST-269 complex | No value | UK | SV-8 | Opc+ |
| 20918 | M03 240456 | Neisseria meningitidis | NG | ST-269 complex | No value | UK | SV-8 | Opc+ |
| 20919 | M03 240553 | Neisseria meningitidis | NG | ST-269 complex | No value | UK | SV-1 | Opc+ |
| 20920 | M03 240554 | Neisseria meningitidis | NG | ST-269 complex | No value | UK | SV-1 | Opc+ |
| 20921 | M03 240607 | Neisseria meningitidis | NG | ST-213 complex | No value | UK | SV-12 | Opc- |
| 20922 | M03 240609 | Neisseria meningitidis | NG | ST-213 complex | No value | UK | SV-12 | Opc- |
| 20923 | M03 240829 | Neisseria meningitidis | NG | ST-213 complex | No value | UK | SV-12 | Opc- |
| 20924 | M03 240830 | Neisseria meningitidis | NG | ST-213 complex | No value | UK | SV-12 | Opc- |
| 20925 | M03 241030 | Neisseria meningitidis | NG | ST-213 complex | No value | Ireland | SV-12 | Opc- |
| 20926 | M03 241054 | Neisseria meningitidis | NG | ST-213 complex | No value | Ireland | SV-12 | Opc- |
| 20927 | M03 241144 | Neisseria meningitidis | NG | ST-282 complex | No value | UK | SV-9 | Opc+ |
| 20928 | M03 241160 | Neisseria meningitidis | NG | ST-282 complex | No value | UK | SV-9 | Opc+ |
| 20929 | M03 241484 | Neisseria meningitidis | NG | ST-269 complex | No value | UK | SV-1 | Opc+ |
| 20930 | M04 240008 | Neisseria meningitidis | NG | ST-32 complex | No value | UK | SV-1 | Opc+ |
| 20931 | M04 240009 | Neisseria meningitidis | NG | ST-32 complex | No value | UK | SV-1 | Opc+ |
| 20932 | M04 240882 | Neisseria meningitidis | NG | ST-213 complex | No value | UK | SV-12 | Opc- |
| 20933 | M04 240883 | Neisseria meningitidis | NG | ST-213 complex | No value | UK | SV-12 | Opc- |
| 20934 | M04 241226 | Neisseria meningitidis | NG | ST-269 complex | No value | UK | SV-1 | Opc+ |
| 20935 | M04 241227 | Neisseria meningitidis | NG | ST-269 complex | No value | UK | SV-1 | Opc+ |
| 20936 | M04 241340 | Neisseria meningitidis | NG | No value | No value | UK | SV-18 | Opc+ |
| 20937 | M04 241703 | Neisseria meningitidis | NG | ST-269 complex | No value | UK | SV-1 | Opc+ |
| 20938 | M04 241707 | Neisseria meningitidis | NG | ST-269 complex | No value | UK | SV-1 | Opc+ |
| 20939 | M05 240140 | Neisseria meningitidis | NG | ST-269 complex | No value | UK | SV-1 | Opc+ |
| 20940 | M05 240156 | Neisseria meningitidis | NG | ST-269 complex | No value | UK | SV-1 | Opc+ |
| 20941 | M05 240218 | Neisseria meningitidis | B | ST-41/44 complex | No value | UK | SV-14 | Opc+ |

| | | | | | | | | |
|---|---|---|---|---|---|---|---|---|
| 20942 | M05 240625 | Neisseria meningitidis | NG | No value | No value | UK | SV-8 | Opc+ |
| 20943 | M05 240626 | Neisseria meningitidis | NG | ST-269 complex | No value | UK | SV-8 | Opc+ |
| 20944 | M05 240890 | Neisseria meningitidis | B | ST-41/44 complex | No value | UK | SV-2 | Opc+ |
| 20945 | M05 240897 | Neisseria meningitidis | B | ST-41/44 complex | No value | UK | SV-2 | Opc+ |
| 20946 | M05 240928 | Neisseria meningitidis | NG | No value | No value | UK | SV-1 | Opc- |
| 20947 | M05 240929 | Neisseria meningitidis | NG | No value | No value | UK | SV-1 | Opc- |
| 20948 | M05 240953 | Neisseria meningitidis | NG | ST-213 complex | No value | UK | SV-12 | Opc- |
| 20949 | M05 240954 | Neisseria meningitidis | NG | ST-213 complex | No value | UK | SV-12 | Opc- |
| 20950 | M05 241004 | Neisseria meningitidis | NG | ST-269 complex | No value | UK | SV-1 | Opc+ |
| 20951 | M05 241005 | Neisseria meningitidis | NG | ST-269 complex | No value | UK | SV-1 | Opc+ |
| 20952 | M05 241251 | Neisseria meningitidis | NG | ST-213 complex | No value | UK | SV-12 | Opc- |
| 20953 | M06 240027 | Neisseria meningitidis | NG | ST-269 complex | No value | UK | SV-1 | Opc+ |
| 20954 | M06 240028 | Neisseria meningitidis | NG | ST-269 complex | No value | UK | SV-1 | Opc+ |
| 20955 | M06 240791 | Neisseria meningitidis | NG | ST-269 complex | No value | UK | SV-1 | Opc+ |
| 20956 | M06 240792 | Neisseria meningitidis | NG | ST-269 complex | No value | UK | SV-1 | Opc+ |
| 20957 | M06 241129 | Neisseria meningitidis | NG | ST-269 complex | No value | UK | SV-8 | Opc+ |
| 20958 | M06 241130 | Neisseria meningitidis | NG | ST-269 complex | No value | UK | SV-8 | Opc+ |
| 20959 | M06 241251 | Neisseria meningitidis | NG | ST-334 complex | No value | Malta | SV-1 | Opc- |
| 20960 | M06 241252 | Neisseria meningitidis | NG | ST-334 complex | No value | Malta | SV-1 | Opc- |
| 20961 | M07 240052 | Neisseria meningitidis | NG | ST-269 complex | No value | UK | SV-1 | Opc+ |
| 20962 | M07 240053 | Neisseria meningitidis | NG | No value | No value | UK | SV-1 | Opc+ |
| 20963 | M07 240711 | Neisseria meningitidis | B | ST-41/44 complex | No value | UK | SV-2 | Opc+ |
| 20964 | M07 240712 | Neisseria meningitidis | B | ST-41/44 complex | No value | UK | SV-2 | Opc+ |
| 20965 | M07 241111 | Neisseria meningitidis | B | ST-41/44 complex | No value | UK | SV-2 | Opc+ |
| 20966 | M07 241112 | Neisseria meningitidis | B | ST-41/44 complex | No value | UK | SV-2 | Opc+ |
| 20967 | M08 240052 | Neisseria meningitidis | NG | ST-269 complex | No value | UK | SV-8 | Opc+ |
| 20968 | M08 240053 | Neisseria meningitidis | NG | ST-269 complex | No value | UK | SV-8 | Opc+ |
| 20969 | M08 240215 | Neisseria meningitidis | NG | ST-269 complex | No value | UK | SV-1 | Opc+ |
| 20970 | M08 240216 | Neisseria meningitidis | NG | ST-269 complex | No value | UK | SV-1 | Opc+ |
| 20971 | M08 240422 | Neisseria meningitidis | NG | ST-60 complex | No value | Isle of Man | SV-11 | Opc+ |
| 20972 | M08 240423 | Neisseria meningitidis | NG | ST-60 complex | No value | Isle of Man | SV-11 | Opc+ |
| 20973 | M08 240474 | Neisseria meningitidis | NG | ST-269 complex | No value | UK | SV-8 | Opc+ |
| 20974 | M08 240475 | Neisseria meningitidis | NG | ST-269 complex | No value | UK | SV-8 | Opc+ |
| 20975 | M97 253181 | Neisseria meningitidis | NG | ST-269 complex | No value | UK | SV-8 | Opc+ |
| 20976 | M97 253182 | Neisseria meningitidis | NG | ST-269 complex | No value | UK | SV-8 | Opc+ |
| 20977 | M97 253394 | Neisseria meningitidis | NG | ST-269 complex | No value | UK | SV-1 | Opc+ |
| 20978 | M97 253441 | Neisseria meningitidis | NG | ST-269 complex | No value | UK | SV-1 | Opc+ |
| 20979 | M98 250319 | Neisseria meningitidis | NG | ST-8 complex | No value | UK | SV-14 | Opc- |
| 20980 | M98 250320 | Neisseria meningitidis | NG | ST-8 complex | No value | UK | SV-14 | Opc- |
| 20981 | M98 251152 | Neisseria meningitidis | NG | ST-213 complex | No value | UK | SV-12 | Opc- |
| 20982 | M98 251154 | Neisseria meningitidis | NG | ST-213 complex | No value | UK | SV-12 | Opc- |
| 21092 | M11 240443 | Neisseria meningitidis | B | ST-461 complex | No value | UK | SV-1 | Opc- |
| 21093 | M11 240445 | Neisseria meningitidis | B | ST-269 complex | No value | UK | SV-8 | Opc+ |
| 21094 | M11 240446 | Neisseria meningitidis | Y | ST-23 complex | No value | UK | SV-11 | Opc+ |
| 21095 | M11 240447 | Neisseria meningitidis | B | ST-41/44 complex | No value | UK | SV-6 | Opc+ |
| 21096 | M11 240448 | Neisseria meningitidis | B | ST-32 complex | No value | UK | SV-1 | Opc+ |
| 21097 | M11 240450 | Neisseria meningitidis | B | ST-269 complex | No value | UK | SV-8 | Opc+ |
| 21098 | M11 240451 | Neisseria meningitidis | B | ST-41/44 complex | No value | UK | SV-2 | Opc+ |
| 21099 | M11 240452 | Neisseria meningitidis | B | ST-213 complex | No value | UK | SV-12 | Opc- |
| 21100 | M11 240453 | Neisseria meningitidis | B | ST-269 complex | No value | UK | SV-8 | Opc+ |
| 21101 | M11 240456 | Neisseria meningitidis | B | ST-213 complex | No value | UK | SV-12 | Opc- |
| 21102 | M11 240457 | Neisseria meningitidis | B | ST-41/44 complex | No value | UK | SV-10 | Opc+ |
| 21103 | M11 240458 | Neisseria meningitidis | B | ST-269 complex | No value | UK | SV-8 | Opc+ |
| 21104 | M11 240459 | Neisseria meningitidis | B | ST-269 complex | No value | UK | SV-8 | Opc+ |
| 21105 | M11 240461 | Neisseria meningitidis | Y | ST-23 complex | No value | UK | SV-11 | Opc+ |
| 21106 | M11 240463 | Neisseria meningitidis | B | ST-269 complex | No value | UK | SV-8 | Opc+ |
| 21107 | M11 240465 | Neisseria meningitidis | Y | ST-23 complex | No value | UK | SV-11 | Opc+ |
| 21108 | M11 240466 | Neisseria meningitidis | Y | ST-23 complex | No value | UK | SV-11 | Opc+ |
| 21109 | M11 240467 | Neisseria meningitidis | B | ST-162 complex | No value | UK | SV-1 | Opc+ |
| 21110 | M11 240469 | Neisseria meningitidis | B | ST-11 complex | No value | UK | SV-2 | Opc- |
| 21111 | M11 240470 | Neisseria meningitidis | B | ST-213 complex | No value | UK | SV-12 | Opc- |
| 21112 | M11 240471 | Neisseria meningitidis | B | ST-60 complex | No value | UK | SV-11 | Opc+ |
| 21113 | M11 240472 | Neisseria meningitidis | B | ST-41/44 complex | No value | UK | SV-10 | Opc- |
| 21114 | M11 240473 | Neisseria meningitidis | B | ST-41/44 complex | No value | UK | SV-2 | Opc+ |
| 21115 | M11 240474 | Neisseria meningitidis | C | No value | No value | UK | SV-6 | Opc+ |
| 21116 | M11 240475 | Neisseria meningitidis | B | No value | No value | UK | SV-5 | Opc+ |
| 21117 | M11 240476 | Neisseria meningitidis | B | ST-41/44 complex | No value | UK | SV-10 | Opc+ |
| 21118 | M11 240477 | Neisseria meningitidis | B | ST-269 complex | No value | UK | SV-1 | Opc+ |
| 21119 | M11 240479 | Neisseria meningitidis | B | ST-162 complex | No value | UK | SV-1 | Opc+ |
| 21120 | M11 240480 | Neisseria meningitidis | B | ST-269 complex | No value | UK | SV-1 | Opc+ |
| 21121 | M11 240484 | Neisseria meningitidis | B | ST-269 complex | No value | UK | SV-8 | Opc+ |
| 21122 | M11 240485 | Neisseria meningitidis | B | ST-41/44 complex | No value | UK | SV-2 | Opc+ |
| 21123 | M11 240486 | Neisseria meningitidis | W | ST-11 complex | No value | UK | SV-2 | Opc- |
| 21124 | M11 240487 | Neisseria meningitidis | B | No value | No value | UK | SV-12 | Opc- |

| | | | | | | | | |
|---|---|---|---|---|---|---|---|---|
| 21125 | M11 240488 | Neisseria meningitidis | B | ST-865 complex | No value | UK | SV-6 | Opc+ |
| 21126 | M11 240489 | Neisseria meningitidis | B | ST-213 complex | No value | UK | SV-12 | Opc- |
| 21127 | M11 240491 | Neisseria meningitidis | B | ST-41/44 complex | No value | UK | SV-2 | Opc+ |
| 21128 | M11 240492 | Neisseria meningitidis | B | ST-41/44 complex | No value | UK | SV-2 | Opc+ |
| 21129 | M11 240493 | Neisseria meningitidis | B | ST-41/44 complex | No value | UK | SV-2 | Opc+ |
| 21130 | M11 240494 | Neisseria meningitidis | Y | ST-23 complex | No value | UK | SV-11 | Opc+ |
| 21131 | M11 240495 | Neisseria meningitidis | B | ST-32 complex | No value | UK | SV-1 | Opc+ |
| 21132 | M11 240497 | Neisseria meningitidis | W | ST-22 complex | No value | UK | SV-11 | Opc+ |
| 21133 | M11 240501 | Neisseria meningitidis | B | No value | No value | UK | SV-11 | Opc+ |
| 21134 | M11 240502 | Neisseria meningitidis | C | ST-11 complex | No value | UK | SV-2 | Opc- |
| 21135 | M11 240504 | Neisseria meningitidis | Y | ST-23 complex | No value | Malta | SV-11 | Opc+ |
| 21136 | M11 240505 | Neisseria meningitidis | Y | ST-23 complex | No value | Malta | SV-11 | Opc+ |
| 21137 | M11 240506 | Neisseria meningitidis | B | No value | No value | UK | SV-6 | Opc+ |
| 21138 | M11 240507 | Neisseria meningitidis | Y | ST-23 complex | No value | UK | SV-11 | Opc+ |
| 21139 | M11 240508 | Neisseria meningitidis | W | ST-22 complex | No value | UK | SV-12 | Opc+ |
| 21140 | M11 240509 | Neisseria meningitidis | B | ST-269 complex | No value | UK | SV-8 | Opc+ |
| 21141 | M11 240510 | Neisseria meningitidis | B | ST-213 complex | No value | UK | SV-12 | Opc- |
| 21142 | M11 240592 | Neisseria meningitidis | B | ST-41/44 complex | No value | UK | SV-2 | Opc+ |
| 21143 | M11 240593 | Neisseria meningitidis | B | ST-269 complex | No value | UK | SV-1 | Opc+ |
| 21144 | M11 240594 | Neisseria meningitidis | B | ST-41/44 complex | No value | UK | SV-2 | Opc+ |
| 21145 | M11 240595 | Neisseria meningitidis | B | ST-213 complex | No value | UK | SV-12 | Opc- |
| 21146 | M11 240597 | Neisseria meningitidis | B | ST-269 complex | No value | UK | SV-8 | Opc+ |
| 21147 | M11 240598 | Neisseria meningitidis | B | ST-213 complex | No value | UK | SV-1 | Opc- |
| 21148 | M11 240599 | Neisseria meningitidis | Y | ST-23 complex | No value | UK | SV-11 | Opc+ |
| 21149 | M11 240600 | Neisseria meningitidis | B | ST-41/44 complex | No value | Malta | SV-10 | Opc+ |
| 21150 | M11 240601 | Neisseria meningitidis | NG | ST-103 complex | No value | Malta | SV-5 | Opc+ |
| 21151 | M11 240657 | Neisseria meningitidis | Y | ST-23 complex | No value | UK | SV-11 | Opc+ |
| 21152 | M11 240707 | Neisseria meningitidis | B | ST-41/44 complex | No value | UK | SV-10 | Opc+ |
| 21153 | M11 240710 | Neisseria meningitidis | Y | ST-23 complex | No value | UK | SV-11 | Opc+ |
| 21154 | M11 240712 | Neisseria meningitidis | B | ST-41/44 complex | No value | UK | SV-10 | Opc+ |
| 21155 | M11 240713 | Neisseria meningitidis | B | No value | No value | UK | SV-8 | Opc+ |
| 21156 | M11 240716 | Neisseria meningitidis | W | ST-22 complex | No value | UK | SV-12 | Opc+ |
| 21157 | M11 240717 | Neisseria meningitidis | Y | ST-23 complex | No value | UK | SV-11 | Opc+ |
| 21158 | M11 240719 | Neisseria meningitidis | Y | ST-23 complex | No value | UK | SV-11 | Opc+ |
| 21159 | M11 240721 | Neisseria meningitidis | B | ST-41/44 complex | No value | UK | SV-2 | Opc+ |
| 21160 | M11 240723 | Neisseria meningitidis | B | No value | No value | UK | SV-11 | Opc- |
| 21161 | M11 240724 | Neisseria meningitidis | B | ST-41/44 complex | No value | UK | SV-5 | Opc+ |
| 21162 | M11 240725 | Neisseria meningitidis | B | ST-269 complex | No value | UK | SV-8 | Opc+ |
| 21163 | M11 240726 | Neisseria meningitidis | W | ST-11 complex | No value | UK | SV-2 | Opc- |
| 21164 | M11 240727 | Neisseria meningitidis | B | ST-213 complex | No value | UK | SV-12 | Opc- |
| 21165 | M11 240728 | Neisseria meningitidis | B | ST-41/44 complex | No value | UK | SV-10 | Opc+ |
| 21166 | M11 240730 | Neisseria meningitidis | Y | ST-23 complex | No value | Malta | SV-11 | Opc+ |
| 21167 | M11 240731 | Neisseria meningitidis | Y | ST-23 complex | No value | UK | SV-11 | Opc+ |
| 21168 | M11 240733 | Neisseria meningitidis | B | ST-269 complex | No value | UK | SV-8 | Opc+ |
| 21169 | M11 240734 | Neisseria meningitidis | Y | ST-23 complex | No value | UK | SV-11 | Opc+ |
| 21170 | M11 240735 | Neisseria meningitidis | B | ST-269 complex | No value | UK | SV-8 | Opc+ |
| 21171 | M11 240736 | Neisseria meningitidis | B | ST-41/44 complex | No value | UK | SV-6 | Opc+ |
| 21172 | M11 240737 | Neisseria meningitidis | Y | ST-23 complex | No value | UK | SV-11 | Opc+ |
| 21173 | M11 240738 | Neisseria meningitidis | B | ST-213 complex | No value | UK | SV-12 | Opc- |
| 21174 | M11 240740 | Neisseria meningitidis | B | No value | No value | UK | SV-1 | Opc+ |
| 21175 | M11 240741 | Neisseria meningitidis | W | ST-22 complex | No value | UK | SV-12 | Opc+ |
| 21176 | M11 240742 | Neisseria meningitidis | B | ST-35 complex | No value | UK | SV-12 | Opc+ |
| 21177 | M11 240743 | Neisseria meningitidis | B | ST-162 complex | No value | UK | SV-1 | Opc+ |
| 21178 | M11 240745 | Neisseria meningitidis | Y | ST-23 complex | No value | UK | SV-11 | Opc+ |
| 21179 | M11 240749 | Neisseria meningitidis | B | ST-32 complex | No value | UK | SV-1 | Opc+ |
| 21180 | M11 240750 | Neisseria meningitidis | B | ST-269 complex | No value | UK | SV-1 | Opc+ |
| 21181 | M11 240762 | Neisseria meningitidis | B | ST-269 complex | No value | UK | SV-8 | Opc+ |
| 21182 | M11 240763 | Neisseria meningitidis | B | No value | No value | UK | SV-2 | Opc+ |
| 21183 | M11 240765 | Neisseria meningitidis | Y | ST-23 complex | No value | UK | SV-11 | Opc+ |
| 21184 | M11 240766 | Neisseria meningitidis | B | ST-41/44 complex | No value | UK | SV-6 | Opc+ |
| 21185 | M11 240768 | Neisseria meningitidis | C | ST-11 complex | No value | Malta | SV-2 | Opc- |
| 21186 | M11 240771 | Neisseria meningitidis | B | ST-18 complex | No value | UK | SV-7 | Opc- |
| 21187 | M11 240772 | Neisseria meningitidis | Y | ST-23 complex | No value | UK | SV-11 | Opc+ |
| 21188 | M11 240773 | Neisseria meningitidis | B | ST-41/44 complex | No value | UK | SV-6 | Opc+ |
| 21189 | M11 240774 | Neisseria meningitidis | B | ST-269 complex | No value | UK | SV-1 | Opc+ |
| 21190 | M11 240775 | Neisseria meningitidis | B | ST-269 complex | No value | UK | SV-1 | Opc+ |
| 21191 | M11 240776 | Neisseria meningitidis | B | ST-269 complex | No value | UK | SV-8 | Opc+ |
| 21192 | M11 240779 | Neisseria meningitidis | Y | ST-23 complex | No value | UK | SV-11 | Opc+ |
| 21193 | M11 240780 | Neisseria meningitidis | W | ST-22 complex | No value | UK | SV-12 | Opc+ |
| 21194 | M11 240781 | Neisseria meningitidis | B | ST-269 complex | No value | UK | SV-1 | Opc+ |
| 21195 | M11 240783 | Neisseria meningitidis | B | ST-41/44 complex | No value | UK | SV-2 | Opc+ |
| 21196 | M11 240785 | Neisseria meningitidis | C | ST-11 complex | No value | UK | SV-2 | Opc- |
| 21197 | M11 240787 | Neisseria meningitidis | B | No value | No value | UK | SV-11 | Opc- |
| 21198 | M11 240788 | Neisseria meningitidis | Y | ST-23 complex | No value | UK | SV-11 | Opc+ |

| | | | | | | | | |
|---|---|---|---|---|---|---|---|---|
| 21199 | M11 240789 | Neisseria meningitidis | B | ST-103 complex | No value | UK | SV-5 | Opc+ |
| 21200 | M11 240790 | Neisseria meningitidis | B | ST-41/44 complex | No value | UK | SV-5 | Opc- |
| 21201 | M11 240793 | Neisseria meningitidis | Y | ST-23 complex | No value | UK | SV-11 | Opc+ |
| 21202 | M11 240796 | Neisseria meningitidis | B | ST-269 complex | No value | UK | SV-1 | Opc+ |
| 21203 | M11 240798 | Neisseria meningitidis | W | ST-11 complex | No value | UK | SV-2 | Opc- |
| 21204 | M11 240799 | Neisseria meningitidis | B | ST-32 complex | No value | UK | SV-1 | Opc+ |
| 21205 | M11 240801 | Neisseria meningitidis | Y | ST-23 complex | No value | UK | SV-11 | Opc+ |
| 21206 | M11 240802 | Neisseria meningitidis | W | ST-11 complex | No value | UK | SV-2 | Opc- |
| 21207 | M11 240803 | Neisseria meningitidis | B | ST-269 complex | No value | UK | SV-8 | Opc+ |
| 21208 | M11 240941 | Neisseria meningitidis | C | ST-11 complex | No value | UK | SV-2 | Opc- |
| 21209 | M11 240942 | Neisseria meningitidis | B | ST-269 complex | No value | UK | SV-8 | Opc+ |
| 21210 | M11 240945 | Neisseria meningitidis | B | ST-41/44 complex | No value | UK | SV-6 | Opc+ |
| 21211 | M11 240946 | Neisseria meningitidis | B | ST-269 complex | No value | UK | SV-8 | Opc+ |
| 21212 | M11 240948 | Neisseria meningitidis | B | ST-213 complex | No value | UK | SV-12 | Opc- |
| 21213 | M11 240949 | Neisseria meningitidis | B | ST-41/44 complex | No value | UK | SV-2 | Opc+ |
| 21214 | M11 240953 | Neisseria meningitidis | W | ST-11 complex | No value | UK | SV-2 | Opc- |
| 21215 | M11 240954 | Neisseria meningitidis | B | ST-269 complex | No value | UK | SV-8 | Opc+ |
| 21216 | M11 240975 | Neisseria meningitidis | W | ST-11 complex | No value | UK | SV-2 | Opc- |
| 21217 | M11 240976 | Neisseria meningitidis | B | ST-213 complex | No value | UK | SV-12 | Opc- |
| 21218 | M11 240977 | Neisseria meningitidis | B | ST-213 complex | No value | UK | SV-12 | Opc- |
| 21219 | M11 240978 | Neisseria meningitidis | B | ST-269 complex | No value | UK | SV-8 | Opc+ |
| 21220 | M11 240979 | Neisseria meningitidis | B | ST-269 complex | No value | UK | SV-8 | Opc+ |
| 21221 | M11 240980 | Neisseria meningitidis | B | ST-213 complex | No value | UK | SV-12 | Opc- |
| 21222 | M11 240981 | Neisseria meningitidis | B | ST-41/44 complex | No value | UK | SV-2 | Opc+ |
| 21223 | M11 240982 | Neisseria meningitidis | Y | ST-23 complex | No value | UK | SV-11 | Opc+ |
| 21224 | M11 240983 | Neisseria meningitidis | B | No value | No value | UK | SV-1 | Opc+ |
| 21225 | M11 240984 | Neisseria meningitidis | B | ST-32 complex | No value | UK | SV-1 | Opc+ |
| 21226 | M11 240986 | Neisseria meningitidis | B | ST-269 complex | No value | UK | SV-8 | Opc+ |
| 21227 | M11 240987 | Neisseria meningitidis | Y | ST-23 complex | No value | UK | SV-11 | Opc+ |
| 21228 | M11 240988 | Neisseria meningitidis | B | ST-461 complex | No value | UK | SV-1 | Opc- |
| 21229 | M11 240991 | Neisseria meningitidis | Y | ST-23 complex | No value | UK | SV-15 | Opc+ |
| 21230 | M11 240992 | Neisseria meningitidis | B | ST-41/44 complex | No value | UK | SV-2 | Opc+ |
| 21231 | M11 240993 | Neisseria meningitidis | B | ST-269 complex | No value | UK | SV-1 | Opc+ |
| 21232 | M11 240994 | Neisseria meningitidis | C | ST-11 complex | No value | UK | SV-2 | Opc- |
| 21233 | M11 240995 | Neisseria meningitidis | B | ST-269 complex | No value | UK | SV-8 | Opc+ |
| 21234 | M11 241013 | Neisseria meningitidis | B | ST-41/44 complex | No value | UK | SV-6 | Opc+ |
| 21235 | M11 241014 | Neisseria meningitidis | B | ST-41/44 complex | No value | UK | SV-2 | Opc+ |
| 21236 | M11 241015 | Neisseria meningitidis | B | ST-41/44 complex | No value | UK | SV-2 | Opc+ |
| 21237 | M11 241016 | Neisseria meningitidis | B | ST-11 complex | No value | UK | SV-2 | Opc- |
| 21238 | M11 241018 | Neisseria meningitidis | B | ST-41/44 complex | No value | UK | SV-2 | Opc+ |
| 21239 | M11 241019 | Neisseria meningitidis | B | ST-32 complex | No value | UK | SV-1 | Opc+ |
| 21240 | M11 241023 | Neisseria meningitidis | B | ST-18 complex | No value | UK | SV-7 | Opc- |
| 21241 | M11 241024 | Neisseria meningitidis | B | ST-269 complex | No value | UK | SV-1 | Opc+ |
| 21242 | M11 241025 | Neisseria meningitidis | B | ST-35 complex | No value | UK | SV-12 | Opc+ |
| 21243 | M11 241026 | Neisseria meningitidis | B | ST-1157 complex | No value | UK | SV-1 | Opc+ |
| 21244 | M11 241027 | Neisseria meningitidis | B | ST-269 complex | No value | UK | SV-8 | Opc+ |
| 21245 | M11 241028 | Neisseria meningitidis | B | ST-41/44 complex | No value | UK | SV-2 | Opc+ |
| 21246 | M11 241031 | Neisseria meningitidis | B | ST-461 complex | No value | UK | SV-1 | Opc- |
| 21247 | M11 241032 | Neisseria meningitidis | B | ST-41/44 complex | No value | UK | SV-2 | Opc+ |
| 21248 | M11 241033 | Neisseria meningitidis | B | ST-269 complex | No value | UK | SV-1 | Opc+ |
| 21249 | M11 241034 | Neisseria meningitidis | B | ST-282 complex | No value | UK | SV-9 | Opc+ |
| 21250 | M11 241035 | Neisseria meningitidis | Y | ST-23 complex | No value | UK | SV-15 | Opc+ |
| 21251 | M11 241036 | Neisseria meningitidis | B | ST-269 complex | No value | UK | SV-1 | Opc+ |
| 21252 | M11 241037 | Neisseria meningitidis | B | ST-1157 complex | No value | UK | SV-1 | Opc+ |
| 21253 | M11 241039 | Neisseria meningitidis | C | ST-11 complex | No value | UK | SV-2 | Opc- |
| 21254 | M11 241040 | Neisseria meningitidis | B | ST-41/44 complex | No value | UK | SV-2 | Opc+ |
| 21255 | M11 241042 | Neisseria meningitidis | Y | ST-23 complex | No value | UK | SV-15 | Opc+ |
| 21256 | M11 241043 | Neisseria meningitidis | C | ST-11 complex | No value | UK | SV-2 | Opc- |
| 21257 | M11 241044 | Neisseria meningitidis | B | ST-41/44 complex | No value | UK | SV-2 | Opc+ |
| 21258 | M11 241046 | Neisseria meningitidis | B | ST-41/44 complex | No value | UK | SV-2 | Opc+ |
| 21259 | M11 241047 | Neisseria meningitidis | Y | ST-23 complex | No value | UK | SV-11 | Opc+ |
| 21260 | M11 241048 | Neisseria meningitidis | B | ST-269 complex | No value | UK | SV-8 | Opc+ |
| 21261 | M11 241050 | Neisseria meningitidis | B | ST-41/44 complex | No value | UK | SV-2 | Opc+ |
| 21262 | M11 241051 | Neisseria meningitidis | Y | ST-23 complex | No value | UK | SV-11 | Opc+ |
| 21263 | M11 241054 | Neisseria meningitidis | B | ST-41/44 complex | No value | UK | SV-2 | Opc+ |
| 21264 | M11 241055 | Neisseria meningitidis | B | ST-41/44 complex | No value | UK | SV-2 | Opc+ |
| 21265 | M11 241057 | Neisseria meningitidis | B | ST-32 complex | No value | UK | SV-1 | Opc+ |
| 21266 | M11 241058 | Neisseria meningitidis | B | ST-162 complex | No value | UK | SV-1 | Opc+ |
| 21267 | M11 241059 | Neisseria meningitidis | B | ST-1157 complex | No value | UK | SV-1 | Opc+ |
| 21268 | M11 241060 | Neisseria meningitidis | B | ST-213 complex | No value | UK | SV-12 | Opc- |
| 21269 | M11 241061 | Neisseria meningitidis | B | ST-269 complex | No value | UK | SV-1 | Opc+ |
| 21270 | M11 241063 | Neisseria meningitidis | B | ST-41/44 complex | No value | UK | SV-2 | Opc+ |
| 21271 | M11 241064 | Neisseria meningitidis | Y | ST-22 complex | No value | UK | SV-12 | Opc+ |
| 21272 | M11 241065 | Neisseria meningitidis | Y | ST-23 complex | No value | UK | SV-11 | Opc+ |

| | | | | | | | | |
|---|---|---|---|---|---|---|---|---|
| 21273 | M11 241066 | Neisseria meningitidis | B | ST-41/44 complex | No value | UK | SV-2 | Opc+ |
| 21274 | M11 241067 | Neisseria meningitidis | Y | ST-23 complex | No value | UK | SV-11 | Opc+ |
| 21275 | M11 241068 | Neisseria meningitidis | B | ST-213 complex | No value | UK | SV-12 | Opc- |
| 21276 | M11 241069 | Neisseria meningitidis | Y | ST-23 complex | No value | UK | SV-15 | Opc+ |
| 21277 | M11 241072 | Neisseria meningitidis | B | No value | No value | UK | SV-6 | Opc+ |
| 21278 | M11 241073 | Neisseria meningitidis | B | ST-41/44 complex | No value | UK | SV-2 | Opc+ |
| 21279 | M11 241074 | Neisseria meningitidis | B | ST-269 complex | No value | UK | SV-8 | Opc+ |
| 21280 | M11 241075 | Neisseria meningitidis | B | ST-60 complex | No value | UK | SV-11 | Opc+ |
| 21281 | M11 241076 | Neisseria meningitidis | B | ST-213 complex | No value | UK | SV-12 | Opc- |
| 21282 | M11 241077 | Neisseria meningitidis | B | ST-269 complex | No value | UK | SV-8 | Opc+ |
| 21283 | M11 241078 | Neisseria meningitidis | Y | ST-23 complex | No value | UK | SV-11 | Opc+ |
| 21284 | M12 240000 | Neisseria meningitidis | B | ST-213 complex | No value | UK | SV-12 | Opc- |
| 21285 | M12 240001 | Neisseria meningitidis | B | ST-269 complex | No value | UK | SV-8 | Opc+ |
| 21286 | M12 240002 | Neisseria meningitidis | B | ST-41/44 complex | No value | UK | SV-6 | Opc+ |
| 21287 | M12 240003 | Neisseria meningitidis | B | ST-269 complex | No value | UK | SV-8 | Opc+ |
| 21288 | M12 240004 | Neisseria meningitidis | W | ST-11 complex | No value | UK | SV-2 | Opc- |
| 21289 | M12 240006 | Neisseria meningitidis | B | ST-269 complex | No value | UK | SV-8 | Opc+ |
| 21290 | M12 240008 | Neisseria meningitidis | W | ST-22 complex | No value | UK | SV-12 | Opc+ |
| 21291 | M12 240009 | Neisseria meningitidis | B | ST-41/44 complex | No value | UK | SV-6 | Opc+ |
| 21292 | M12 240010 | Neisseria meningitidis | B | ST-41/44 complex | No value | UK | SV-2 | Opc+ |
| 21293 | M12 240011 | Neisseria meningitidis | Y | ST-23 complex | No value | UK | SV-11 | Opc+ |
| 21294 | M12 240012 | Neisseria meningitidis | Y | ST-23 complex | No value | UK | SV-11 | Opc+ |
| 21295 | M12 240013 | Neisseria meningitidis | Y | ST-23 complex | No value | UK | SV-11 | Opc+ |
| 21296 | M12 240014 | Neisseria meningitidis | C | ST-41/44 complex | No value | UK | SV-10 | Opc+ |
| 21297 | M12 240015 | Neisseria meningitidis | NG | ST-269 complex | No value | UK | SV-1 | Opc+ |
| 21298 | M12 240016 | Neisseria meningitidis | W | ST-11 complex | No value | UK | SV-2 | Opc- |
| 21299 | M12 240018 | Neisseria meningitidis | B | ST-41/44 complex | No value | UK | SV-2 | Opc+ |
| 21300 | M12 240019 | Neisseria meningitidis | B | ST-461 complex | No value | UK | SV-1 | Opc- |
| 21301 | M12 240020 | Neisseria meningitidis | B | ST-41/44 complex | No value | UK | SV-2 | Opc+ |
| 21302 | M12 240021 | Neisseria meningitidis | W | ST-11 complex | No value | UK | SV-2 | Opc- |
| 21303 | M12 240022 | Neisseria meningitidis | B | ST-213 complex | No value | UK | SV-17 | Opc- |
| 21304 | M12 240024 | Neisseria meningitidis | Y | ST-23 complex | No value | UK | SV-15 | Opc+ |
| 21305 | M12 240027 | Neisseria meningitidis | W | ST-11 complex | No value | UK | SV-8 | Opc- |
| 21306 | M12 240028 | Neisseria meningitidis | B | ST-213 complex | No value | UK | SV-12 | Opc- |
| 21307 | M12 240029 | Neisseria meningitidis | B | ST-269 complex | No value | UK | SV-8 | Opc+ |
| 21308 | M12 240030 | Neisseria meningitidis | B | ST-269 complex | No value | UK | SV-8 | Opc+ |
| 21309 | M12 240031 | Neisseria meningitidis | B | ST-162 complex | No value | UK | SV-1 | Opc+ |
| 21310 | M12 240032 | Neisseria meningitidis | B | ST-41/44 complex | No value | UK | SV-2 | Opc+ |
| 21311 | M12 240033 | Neisseria meningitidis | B | ST-11 complex | No value | UK | SV-2 | Opc- |
| 21312 | M12 240034 | Neisseria meningitidis | B | ST-269 complex | No value | UK | SV-1 | Opc+ |
| 21313 | M12 240035 | Neisseria meningitidis | B | ST-41/44 complex | No value | UK | SV-2 | Opc+ |
| 21314 | M12 240036 | Neisseria meningitidis | B | ST-41/44 complex | No value | UK | SV-5 | Opc+ |
| 21315 | M12 240038 | Neisseria meningitidis | B | ST-162 complex | No value | UK | SV-1 | Opc+ |
| 21316 | M12 240039 | Neisseria meningitidis | B | ST-41/44 complex | No value | UK | SV-2 | Opc+ |
| 21317 | M12 240040 | Neisseria meningitidis | B | ST-269 complex | No value | UK | SV-1 | Opc+ |
| 21318 | M12 240041 | Neisseria meningitidis | B | ST-41/44 complex | No value | UK | SV-2 | Opc+ |
| 21319 | M12 240042 | Neisseria meningitidis | B | ST-41/44 complex | No value | UK | SV-2 | Opc+ |
| 21320 | M12 240044 | Neisseria meningitidis | B | No value | No value | UK | SV-7 | Opc+ |
| 21321 | M12 240045 | Neisseria meningitidis | B | ST-41/44 complex | No value | UK | SV-6 | Opc+ |
| 21322 | M12 240046 | Neisseria meningitidis | B | ST-269 complex | No value | UK | SV-8 | Opc+ |
| 21323 | M12 240047 | Neisseria meningitidis | B | ST-213 complex | No value | UK | SV-12 | Opc- |
| 21324 | M12 240048 | Neisseria meningitidis | B | No value | No value | UK | SV-1 | Opc+ |
| 21325 | M12 240051 | Neisseria meningitidis | B | ST-162 complex | No value | UK | SV-1 | Opc+ |
| 21326 | M12 240054 | Neisseria meningitidis | B | ST-32 complex | No value | UK | SV-1 | Opc+ |
| 21327 | M12 240057 | Neisseria meningitidis | B | ST-269 complex | No value | UK | SV-1 | Opc+ |
| 21328 | M12 240058 | Neisseria meningitidis | B | ST-32 complex | No value | UK | SV-1 | Opc+ |
| 21329 | M12 240059 | Neisseria meningitidis | B | ST-269 complex | No value | UK | SV-8 | Opc+ |
| 21330 | M12 240061 | Neisseria meningitidis | B | ST-11 complex | No value | UK | SV-2 | Opc- |
| 21331 | M12 240062 | Neisseria meningitidis | B | ST-18 complex | No value | UK | SV-7 | Opc- |
| 21332 | M12 240063 | Neisseria meningitidis | B | ST-213 complex | No value | UK | SV-12 | Opc- |
| 21333 | M12 240066 | Neisseria meningitidis | B | ST-269 complex | No value | UK | SV-1 | Opc+ |
| 21334 | M12 240067 | Neisseria meningitidis | W | ST-11 complex | No value | UK | SV-2 | Opc- |
| 21335 | M12 240069 | Neisseria meningitidis | B | ST-11 complex | No value | UK | SV-2 | Opc- |
| 21336 | M12 240070 | Neisseria meningitidis | B | No value | No value | UK | SV-8 | Opc- |
| 21337 | M12 240071 | Neisseria meningitidis | B | ST-41/44 complex | No value | UK | SV-6 | Opc+ |
| 21338 | M12 240072 | Neisseria meningitidis | Y | ST-23 complex | No value | UK | SV-11 | Opc+ |
| 21339 | M12 240076 | Neisseria meningitidis | Y | ST-174 complex | No value | UK | SV-1 | Opc+ |
| 21340 | M12 240077 | Neisseria meningitidis | B | ST-41/44 complex | No value | UK | SV-6 | Opc+ |
| 21341 | M12 240078 | Neisseria meningitidis | B | ST-41/44 complex | No value | UK | SV-2 | Opc+ |
| 21342 | M12 240079 | Neisseria meningitidis | B | ST-269 complex | No value | UK | SV-8 | Opc+ |
| 21343 | M12 240080 | Neisseria meningitidis | Y | ST-23 complex | No value | UK | SV-11 | Opc+ |
| 21344 | M12 240081 | Neisseria meningitidis | B | ST-41/44 complex | No value | UK | SV-6 | Opc+ |
| 21345 | M12 240083 | Neisseria meningitidis | B | ST-213 complex | No value | UK | SV-12 | Opc- |
| 21346 | M12 240084 | Neisseria meningitidis | Y | ST-23 complex | No value | UK | SV-11 | Opc+ |

| | | | | | | | | |
|---|---|---|---|---|---|---|---|---|
| 21347 | M12 240085 | Neisseria meningitidis | B | ST-41/44 complex | No value | UK | SV-2 | Opc+ |
| 21348 | M12 240086 | Neisseria meningitidis | B | ST-269 complex | No value | UK | SV-1 | Opc+ |
| 21349 | M12 240088 | Neisseria meningitidis | W | ST-22 complex | No value | UK | SV-12 | Opc+ |
| 21350 | M12 240090 | Neisseria meningitidis | B | ST-269 complex | No value | UK | SV-8 | Opc+ |
| 21351 | M12 240092 | Neisseria meningitidis | B | ST-213 complex | No value | UK | SV-12 | Opc- |
| 21352 | M12 240093 | Neisseria meningitidis | E | ST-60 complex | No value | UK | SV-11 | Opc+ |
| 21353 | M12 240094 | Neisseria meningitidis | B | ST-41/44 complex | No value | UK | SV-2 | Opc+ |
| 21354 | M12 240095 | Neisseria meningitidis | W | ST-11 complex | No value | UK | SV-2 | Opc- |
| 21355 | M12 240097 | Neisseria meningitidis | B | ST-41/44 complex | No value | UK | SV-6 | Opc+ |
| 21356 | M12 240098 | Neisseria meningitidis | Y | ST-23 complex | No value | UK | SV-11 | Opc+ |
| 21357 | M12 240099 | Neisseria meningitidis | B | ST-269 complex | No value | UK | SV-8 | Opc+ |
| 21358 | M12 240100 | Neisseria meningitidis | B | ST-269 complex | No value | UK | SV-1 | Opc+ |
| 21359 | M12 240101 | Neisseria meningitidis | C | ST-11 complex | No value | UK | SV-2 | Opc- |
| 21360 | M12 240103 | Neisseria meningitidis | B | ST-41/44 complex | No value | UK | SV-10 | Opc+ |
| 21361 | M12 240104 | Neisseria meningitidis | NG | ST-11 complex | No value | UK | SV-2 | Opc- |
| 21362 | M12 240105 | Neisseria meningitidis | B | ST-269 complex | No value | UK | SV-8 | Opc+ |
| 21363 | M12 240107 | Neisseria meningitidis | B | ST-213 complex | No value | UK | SV-12 | Opc- |
| 21364 | M12 240111 | Neisseria meningitidis | C | ST-11 complex | No value | UK | SV-2 | Opc- |
| 21365 | M12 240114 | Neisseria meningitidis | B | ST-213 complex | No value | UK | SV-12 | Opc- |
| 21366 | M12 240115 | Neisseria meningitidis | Y | ST-23 complex | No value | UK | SV-11 | Opc+ |
| 21367 | M12 240116 | Neisseria meningitidis | B | ST-269 complex | No value | UK | SV-1 | Opc+ |
| 21368 | M12 240117 | Neisseria meningitidis | B | ST-41/44 complex | No value | UK | SV-6 | Opc+ |
| 21369 | M12 240118 | Neisseria meningitidis | B | ST-41/44 complex | No value | UK | SV-2 | Opc+ |
| 21370 | M12 240120 | Neisseria meningitidis | B | No value | No value | UK | SV-8 | Opc+ |
| 21371 | M12 240121 | Neisseria meningitidis | W | ST-22 complex | No value | UK | SV-12 | Opc+ |
| 21372 | M12 240122 | Neisseria meningitidis | Y | ST-23 complex | No value | UK | SV-11 | Opc+ |
| 21373 | M12 240123 | Neisseria meningitidis | Y | ST-23 complex | No value | UK | SV-11 | Opc+ |
| 21374 | M12 240124 | Neisseria meningitidis | B | ST-41/44 complex | No value | UK | SV-2 | Opc+ |
| 21375 | M12 240125 | Neisseria meningitidis | W | ST-11 complex | No value | UK | SV-2 | Opc- |
| 21376 | M12 240126 | Neisseria meningitidis | B | ST-41/44 complex | No value | UK | SV-2 | Opc+ |
| 21377 | M12 240127 | Neisseria meningitidis | W | ST-11 complex | No value | UK | SV-2 | Opc- |
| 21378 | M12 240128 | Neisseria meningitidis | B | ST-41/44 complex | No value | UK | SV-2 | Opc+ |
| 21379 | M12 240131 | Neisseria meningitidis | NG | ST-41/44 complex | No value | UK | SV-2 | Opc+ |
| 21380 | M12 240132 | Neisseria meningitidis | W | ST-22 complex | No value | UK | SV-12 | Opc+ |
| 21381 | M12 240133 | Neisseria meningitidis | W | ST-11 complex | No value | UK | SV-2 | Opc- |
| 21382 | M12 240134 | Neisseria meningitidis | B | ST-41/44 complex | No value | UK | SV-2 | Opc+ |
| 21383 | M12 240135 | Neisseria meningitidis | B | ST-1157 complex | No value | UK | SV-1 | Opc+ |
| 21384 | M12 240138 | Neisseria meningitidis | B | ST-41/44 complex | No value | UK | SV-10 | Opc+ |
| 21385 | M12 240142 | Neisseria meningitidis | B | ST-213 complex | No value | UK | SV-12 | Opc- |
| 21386 | M12 240144 | Neisseria meningitidis | W | ST-11 complex | No value | UK | SV-2 | Opc- |
| 21387 | M12 240145 | Neisseria meningitidis | B | ST-213 complex | No value | UK | SV-12 | Opc- |
| 21388 | M12 240146 | Neisseria meningitidis | B | ST-32 complex | No value | UK | SV-1 | Opc+ |
| 21389 | M12 240147 | Neisseria meningitidis | NG | ST-198 complex | No value | Austria | SV-4 | Opc+ |
| 21390 | M12 240149 | Neisseria meningitidis | B | ST-32 complex | No value | UK | SV-1 | Opc+ |
| 21391 | M12 240150 | Neisseria meningitidis | B | No value | No value | UK | SV-8 | Opc+ |
| 21392 | M12 240151 | Neisseria meningitidis | B | ST-213 complex | No value | UK | SV-12 | Opc- |
| 21393 | M12 240153 | Neisseria meningitidis | B | ST-269 complex | No value | UK | SV-1 | Opc+ |
| 21394 | M12 240155 | Neisseria meningitidis | B | ST-41/44 complex | No value | UK | SV-2 | Opc+ |
| 21396 | M12 240158 | Neisseria meningitidis | B | ST-60 complex | No value | UK | SV-5 | Opc+ |
| 21398 | M12 240161 | Neisseria meningitidis | B | ST-41/44 complex | No value | UK | SV-2 | Opc+ |
| 21399 | M12 240166 | Neisseria meningitidis | Y | ST-23 complex | No value | UK | SV-11 | Opc+ |
| 21400 | M12 240167 | Neisseria meningitidis | B | ST-41/44 complex | No value | UK | SV-2 | Opc+ |
| 21401 | M12 240168 | Neisseria meningitidis | B | ST-41/44 complex | No value | UK | SV-14 | Opc+ |
| 21402 | M12 240169 | Neisseria meningitidis | NG | ST-41/44 complex | No value | UK | SV-6 | Opc+ |
| 21403 | M12 240171 | Neisseria meningitidis | Y | ST-23 complex | No value | UK | SV-11 | Opc+ |
| 21404 | M12 240174 | Neisseria meningitidis | Y | ST-23 complex | No value | UK | SV-11 | Opc+ |
| 21405 | M12 240175 | Neisseria meningitidis | B | ST-269 complex | No value | UK | SV-8 | Opc+ |
| 21406 | M12 240176 | Neisseria meningitidis | B | ST-269 complex | No value | UK | SV-8 | Opc+ |
| 21407 | M12 240177 | Neisseria meningitidis | C | ST-11 complex | No value | UK | SV-2 | Opc- |
| 21408 | M12 240180 | Neisseria meningitidis | Y | ST-23 complex | No value | UK | SV-11 | Opc+ |
| 21409 | M12 240181 | Neisseria meningitidis | Y | ST-167 complex | No value | UK | SV-5 | Opc+ |
| 21410 | M12 240184 | Neisseria meningitidis | Y | ST-23 complex | No value | UK | SV-11 | Opc+ |
| 21411 | M12 240186 | Neisseria meningitidis | B | ST-41/44 complex | No value | UK | SV-6 | Opc+ |
| 21412 | M12 240187 | Neisseria meningitidis | B | ST-41/44 complex | No value | UK | SV-2 | Opc+ |
| 21413 | M12 240188 | Neisseria meningitidis | B | ST-18 complex | No value | UK | SV-5 | Opc- |
| 21414 | M12 240191 | Neisseria meningitidis | B | ST-41/44 complex | No value | UK | SV-10 | Opc+ |
| 21415 | M12 240194 | Neisseria meningitidis | B | No value | No value | UK | SV-1 | Opc+ |
| 21416 | M12 240197 | Neisseria meningitidis | B | ST-162 complex | No value | UK | SV-1 | Opc+ |
| 21417 | M12 240200 | Neisseria meningitidis | C | ST-103 complex | No value | UK | SV-5 | Opc+ |
| 21418 | M12 240201 | Neisseria meningitidis | B | ST-269 complex | No value | UK | SV-1 | Opc+ |
| 21419 | M12 240202 | Neisseria meningitidis | C | ST-11 complex | No value | UK | SV-2 | Opc- |
| 21420 | M12 240203 | Neisseria meningitidis | B | ST-162 complex | No value | UK | SV-1 | Opc+ |
| 21421 | M12 240205 | Neisseria meningitidis | Y | ST-174 complex | No value | UK | SV-1 | Opc+ |
| 21422 | M12 240206 | Neisseria meningitidis | B | ST-269 complex | No value | UK | SV-8 | Opc+ |

| | | | | | | | | |
|---|---|---|---|---|---|---|---|---|
| 21423 | M12 240207 | Neisseria meningitidis | B | ST-41/44 complex | No value | UK | SV-2 | Opc+ |
| 21424 | M12 240208 | Neisseria meningitidis | B | ST-213 complex | No value | UK | SV-12 | Opc- |
| 21425 | M12 240211 | Neisseria meningitidis | B | ST-269 complex | No value | UK | SV-1 | Opc+ |
| 21426 | M12 240213 | Neisseria meningitidis | B | ST-41/44 complex | No value | UK | SV-2 | Opc+ |
| 21427 | M12 240214 | Neisseria meningitidis | B | No value | No value | UK | SV-13 | Opc+ |
| 21428 | M12 240215 | Neisseria meningitidis | B | ST-213 complex | No value | UK | SV-12 | Opc- |
| 21429 | M12 240216 | Neisseria meningitidis | B | No value | No value | UK | SV-8 | Opc+ |
| 21430 | M12 240219 | Neisseria meningitidis | C | ST-269 complex | No value | UK | SV-1 | Opc+ |
| 21431 | M12 240220 | Neisseria meningitidis | B | ST-41/44 complex | No value | UK | SV-2 | Opc+ |
| 21432 | M12 240222 | Neisseria meningitidis | B | ST-60 complex | No value | UK | SV-11 | Opc+ |
| 21433 | M12 240223 | Neisseria meningitidis | B | ST-269 complex | No value | UK | SV-8 | Opc+ |
| 21434 | M12 240224 | Neisseria meningitidis | B | ST-41/44 complex | No value | UK | SV-2 | Opc+ |
| 21435 | M12 240225 | Neisseria meningitidis | B | ST-213 complex | No value | UK | SV-6 | Opc- |
| 21436 | M12 240226 | Neisseria meningitidis | B | ST-213 complex | No value | UK | SV-12 | Opc- |
| 21437 | M12 240227 | Neisseria meningitidis | B | ST-41/44 complex | No value | UK | SV-2 | Opc+ |
| 21438 | M12 240228 | Neisseria meningitidis | B | ST-213 complex | No value | UK | SV-12 | Opc- |
| 21439 | M12 240229 | Neisseria meningitidis | B | ST-269 complex | No value | UK | SV-8 | Opc+ |
| 21440 | M12 240230 | Neisseria meningitidis | Y | ST-23 complex | No value | UK | SV-11 | Opc+ |
| 21441 | M12 240231 | Neisseria meningitidis | B | ST-32 complex | No value | UK | SV-1 | Opc+ |
| 21442 | M12 240232 | Neisseria meningitidis | B | No value | No value | UK | SV-8 | Opc+ |
| 21443 | M12 240237 | Neisseria meningitidis | Y | ST-23 complex | No value | UK | SV-11 | Opc+ |
| 21444 | M12 240238 | Neisseria meningitidis | B | No value | No value | UK | SV-15 | Opc+ |
| 21445 | M12 240239 | Neisseria meningitidis | B | ST-269 complex | No value | UK | SV-1 | Opc+ |
| 21446 | M12 240240 | Neisseria meningitidis | W | ST-11 complex | No value | UK | SV-2 | Opc- |
| 21447 | M12 240241 | Neisseria meningitidis | Y | ST-23 complex | No value | UK | SV-11 | Opc+ |
| 21448 | M12 240242 | Neisseria meningitidis | Y | ST-23 complex | No value | UK | SV-11 | Opc+ |
| 21449 | M12 240243 | Neisseria meningitidis | B | ST-269 complex | No value | UK | SV-1 | Opc+ |
| 21450 | M12 240245 | Neisseria meningitidis | B | ST-41/44 complex | No value | UK | SV-6 | Opc+ |
| 21451 | M12 240248 | Neisseria meningitidis | Y | ST-23 complex | No value | UK | SV-11 | Opc+ |
| 21452 | M12 240249 | Neisseria meningitidis | B | ST-41/44 complex | No value | UK | SV-2 | Opc+ |
| 21453 | M12 240250 | Neisseria meningitidis | B | No value | No value | UK | SV-18 | Opc- |
| 21454 | M12 240251 | Neisseria meningitidis | W | ST-22 complex | No value | UK | SV-12 | Opc+ |
| 21455 | M12 240252 | Neisseria meningitidis | B | ST-41/44 complex | No value | UK | SV-6 | Opc+ |
| 21456 | M12 240253 | Neisseria meningitidis | B | ST-213 complex | No value | UK | SV-12 | Opc- |
| 21457 | M12 240254 | Neisseria meningitidis | B | ST-213 complex | No value | UK | SV-12 | Opc- |
| 21458 | M12 240255 | Neisseria meningitidis | C | ST-11 complex | No value | UK | SV-2 | Opc- |
| 21460 | M12 240257 | Neisseria meningitidis | B | ST-41/44 complex | No value | UK | SV-10 | Opc+ |
| 21461 | M12 240259 | Neisseria meningitidis | B | No value | No value | UK | SV-8 | Opc- |
| 21462 | M12 240261 | Neisseria meningitidis | B | ST-32 complex | No value | UK | SV-1 | Opc+ |
| 21463 | M12 240263 | Neisseria meningitidis | B | ST-41/44 complex | No value | UK | SV-2 | Opc+ |
| 21464 | M12 240264 | Neisseria meningitidis | B | ST-41/44 complex | No value | UK | SV-2 | Opc+ |
| 21465 | M12 240267 | Neisseria meningitidis | B | ST-269 complex | No value | UK | SV-1 | Opc+ |
| 21466 | M12 240272 | Neisseria meningitidis | Y | ST-23 complex | No value | UK | SV-11 | Opc+ |
| 21467 | M12 240273 | Neisseria meningitidis | B | ST-213 complex | No value | UK | SV-12 | Opc- |
| 21468 | M12 240274 | Neisseria meningitidis | B | ST-41/44 complex | No value | UK | SV-2 | Opc+ |
| 21469 | M12 240277 | Neisseria meningitidis | B | ST-32 complex | No value | UK | SV-1 | Opc+ |
| 21470 | M12 240284 | Neisseria meningitidis | B | ST-41/44 complex | No value | UK | SV-2 | Opc+ |
| 21471 | M12 240287 | Neisseria meningitidis | B | ST-269 complex | No value | UK | SV-8 | Opc+ |
| 21472 | M12 240288 | Neisseria meningitidis | Y | ST-23 complex | No value | UK | SV-11 | Opc+ |
| 21473 | M12 240289 | Neisseria meningitidis | Y | ST-23 complex | No value | UK | SV-11 | Opc+ |
| 21474 | M12 240290 | Neisseria meningitidis | B | ST-41/44 complex | No value | UK | SV-6 | Opc+ |
| 21475 | M12 240291 | Neisseria meningitidis | Y | ST-23 complex | No value | UK | SV-11 | Opc+ |
| 21476 | M12 240293 | Neisseria meningitidis | Y | ST-23 complex | No value | UK | SV-11 | Opc+ |
| 21477 | M12 240294 | Neisseria meningitidis | B | ST-269 complex | No value | UK | SV-1 | Opc+ |
| 21478 | M12 240296 | Neisseria meningitidis | B | ST-41/44 complex | No value | UK | SV-1 | Opc+ |
| 21479 | M12 240299 | Neisseria meningitidis | B | ST-41/44 complex | No value | UK | SV-2 | Opc+ |
| 21480 | M12 240300 | Neisseria meningitidis | Y | ST-23 complex | No value | UK | SV-11 | Opc+ |
| 21481 | M12 240301 | Neisseria meningitidis | B | ST-41/44 complex | No value | UK | SV-2 | Opc+ |
| 21482 | M12 240302 | Neisseria meningitidis | B | No value | No value | UK | SV-10 | Opc+ |
| 21483 | M12 240303 | Neisseria meningitidis | B | No value | No value | UK | SV-8 | Opc+ |
| 21484 | M12 240305 | Neisseria meningitidis | B | ST-41/44 complex | No value | UK | SV-2 | Opc+ |
| 21485 | M12 240306 | Neisseria meningitidis | B | No value | No value | UK | SV-8 | Opc+ |
| 21486 | M12 240307 | Neisseria meningitidis | B | ST-269 complex | No value | UK | SV-1 | Opc+ |
| 21487 | M12 240308 | Neisseria meningitidis | B | ST-41/44 complex | No value | UK | SV-2 | Opc+ |
| 21488 | M12 240309 | Neisseria meningitidis | B | ST-41/44 complex | No value | UK | SV-5 | Opc+ |
| 21489 | M12 240310 | Neisseria meningitidis | B | ST-269 complex | No value | UK | SV-1 | Opc+ |
| 21490 | M12 240314 | Neisseria meningitidis | B | ST-41/44 complex | No value | UK | SV-6 | Opc+ |
| 21491 | M12 240315 | Neisseria meningitidis | B | ST-41/44 complex | No value | UK | SV-6 | Opc+ |
| 21492 | M12 240317 | Neisseria meningitidis | W | ST-11 complex | No value | UK | SV-2 | Opc- |
| 21493 | M12 240318 | Neisseria meningitidis | Y | ST-23 complex | No value | UK | SV-11 | Opc+ |
| 21494 | M12 240319 | Neisseria meningitidis | B | ST-41/44 complex | No value | UK | SV-2 | Opc+ |
| 21495 | M12 240320 | Neisseria meningitidis | B | ST-32 complex | No value | UK | SV-1 | Opc+ |
| 21496 | M12 240321 | Neisseria meningitidis | B | ST-41/44 complex | No value | UK | SV-6 | Opc+ |
| 21497 | M12 240322 | Neisseria meningitidis | Y | ST-23 complex | No value | UK | SV-11 | Opc+ |

| | | | | | | | | |
|---|---|---|---|---|---|---|---|---|
| 21498 | M12 240323 | Neisseria meningitidis | C | ST-269 complex | No value | UK | SV-1 | Opc+ |
| 21499 | M12 240324 | Neisseria meningitidis | W | ST-11 complex | No value | UK | SV-2 | Opc- |
| 21500 | M12 240325 | Neisseria meningitidis | B | ST-41/44 complex | No value | UK | SV-2 | Opc+ |
| 21501 | M12 240326 | Neisseria meningitidis | B | ST-213 complex | No value | UK | SV-12 | Opc- |
| 21502 | M12 240328 | Neisseria meningitidis | Y | ST-23 complex | No value | UK | SV-11 | Opc+ |
| 21503 | M12 240329 | Neisseria meningitidis | B | ST-213 complex | No value | UK | SV-12 | Opc- |
| 21504 | M12 240330 | Neisseria meningitidis | Y | ST-167 complex | No value | UK | SV-5 | Opc+ |
| 21505 | M12 240332 | Neisseria meningitidis | Y | ST-103 complex | No value | UK | SV-5 | Opc+ |
| 21506 | M12 240333 | Neisseria meningitidis | B | ST-41/44 complex | No value | UK | SV-2 | Opc+ |
| 21507 | M12 240334 | Neisseria meningitidis | B | ST-41/44 complex | No value | UK | SV-6 | Opc+ |
| 21508 | M12 240335 | Neisseria meningitidis | B | ST-269 complex | No value | UK | SV-8 | Opc+ |
| 21509 | M12 240641 | Neisseria meningitidis | NG | ST-1157 complex | No value | UK | SV-1 | Opc+ |
| 21573 | SA_serogroup W_NM1 | Neisseria meningitidis | W | ST-11 complex | No value | South Africa | SV-2 | Opc- |
| 21574 | SA_serogroup W_NM2 | Neisseria meningitidis | W | ST-865 complex | No value | South Africa | SV-5 | Opc+ |
| 21575 | SA_serogroup A_NM3 | Neisseria meningitidis | A | No value | No value | South Africa | SV-1 | Opc- |
| 21576 | SA_serogroup W_NM4 | Neisseria meningitidis | W | ST-22 complex | No value | South Africa | SV-12 | Opc+ |
| 21577 | SA_serogroup A_NM5 | Neisseria meningitidis | A | ST-1 complex | No value | South Africa | SV-5 | Opc+ |
| 21578 | SA_serogroup W_NM6 | Neisseria meningitidis | W | ST-11 complex | No value | South Africa | SV-2 | Opc- |
| 21579 | SA_serogroup A_NM7 | Neisseria meningitidis | A | ST-1 complex | No value | South Africa | SV-5 | Opc+ |
| 21580 | SA_serogroup A_NM8 | Neisseria meningitidis | A | ST-1 complex | No value | South Africa | SV-5 | Opc+ |
| 21581 | SA_serogroup W_NM9 | Neisseria meningitidis | W | ST-11 complex | No value | South Africa | SV-2 | Opc- |
| 21582 | SA_serogroup W_NM10 | Neisseria meningitidis | W | ST-11 complex | No value | South Africa | SV-2 | Opc- |
| 21583 | SA_serogroup W_NM11 | Neisseria meningitidis | W | ST-11 complex | No value | South Africa | SV-2 | Opc- |
| 21584 | SA_serogroup W_NM12 | Neisseria meningitidis | W | ST-11 complex | No value | South Africa | SV-2 | Opc- |
| 21585 | SA_serogroup A_NM13 | Neisseria meningitidis | A | ST-1 complex | No value | South Africa | SV-5 | Opc+ |
| 21586 | SA_serogroup A_NM14 | Neisseria meningitidis | A | ST-1 complex | No value | South Africa | SV-5 | Opc+ |
| 21587 | SA_serogroup W_NM15 | Neisseria meningitidis | W | ST-11 complex | No value | South Africa | SV-2 | Opc- |
| 21588 | SA_serogroup W_NM16 | Neisseria meningitidis | W | ST-11 complex | No value | South Africa | SV-2 | Opc- |
| 26024 | 58 | Neisseria meningitidis | B | ST-32 complex | No value | Japan | SV-1 | Opc+ |
| 26025 | 86 | Neisseria meningitidis | B | ST-32 complex | No value | Japan | SV-3 | Opc+ |
| 26026 | 277 | Neisseria meningitidis | B | ST-32 complex | No value | China | SV-3 | Opc+ |
| 26027 | 350 | Neisseria meningitidis | B | ST-32 complex | No value | South Africa | SV-1 | Opc+ |
| 26028 | 2645 | Neisseria meningitidis | B | ST-32 complex | No value | China | SV-3 | Opc+ |
| 26029 | 8733 | Neisseria meningitidis | B | ST-32 complex | No value | Chile | SV-1 | Opc+ |
| 26030 | 79694 | Neisseria meningitidis | B | ST-32 complex | No value | Canada | SV-3 | Opc+ |
| 26031 | 82238 | Neisseria meningitidis | B | ST-32 complex | No value | Canada | SV-1 | Opc+ |
| 26032 | 96038 | Neisseria meningitidis | B | ST-32 complex | No value | Canada | SV-1 | Opc+ |
| 26033 | 25/76 | Neisseria meningitidis | B | ST-32 complex | No value | Denmark | SV-1 | Opc+ |
| 26034 | 27/86 | Neisseria meningitidis | B | ST-32 complex | No value | Brazil | SV-1 | Opc+ |
| 26035 | 270/94 | Neisseria meningitidis | B | ST-32 complex | No value | Argentina | SV-1 | Opc+ |
| 26036 | 70/92 | Neisseria meningitidis | B | ST-32 complex | No value | Cuba | SV-1 | Opc+ |
| 26037 | 71/87 | Neisseria meningitidis | B | ST-32 complex | No value | Brazil | SV-1 | Opc+ |
| 26038 | 84/89 | Neisseria meningitidis | B | ST-32 complex | No value | Brazil | SV-1 | Opc+ |
| 26039 | 92/30 | Neisseria meningitidis | B | ST-32 complex | No value | New Zealand | SV-1 | Opc+ |
| 26040 | 93-N213 | Neisseria meningitidis | NG | ST-32 complex | No value | Australia | SV-3 | Opc+ |
| 26041 | AO5 | Neisseria meningitidis | B | ST-32 complex | No value | South Africa | SV-1 | Opc+ |
| 26042 | H1100/87 | Neisseria meningitidis | B | ST-32 complex | No value | UK | SV-1 | Opc+ |
| 26043 | H172 | Neisseria meningitidis | B | ST-32 complex | No value | USA | SV-1 | Opc+ |
| 26044 | H355 | Neisseria meningitidis | B | ST-32 complex | No value | Norway | SV-1 | Opc+ |
| 26045 | M1037 | Neisseria meningitidis | B | ST-32 complex | No value | USA | SV-1 | Opc+ |
| 26046 | M2528 | Neisseria meningitidis | B | ST-32 complex | No value | USA | SV-1 | Opc+ |
| 26047 | M50 | Neisseria meningitidis | B | ST-32 complex | No value | Morocco | SV-1 | Opc+ |
| 26048 | M64 | Neisseria meningitidis | B | ST-32 complex | No value | Morocco | SV-1 | Opc+ |
| 26049 | MA5587 | Neisseria meningitidis | B | ST-32 complex | No value | Spain | SV-1 | Opc+ |
| 26050 | MA5873 | Neisseria meningitidis | B | ST-32 complex | No value | Spain | SV-1 | Opc+ |
| 26051 | MK521/99 | Neisseria meningitidis | B | ST-32 complex | No value | Ivory Coast | SV-1 | Opc+ |
| 26052 | N20/08 | Neisseria meningitidis | B | ST-32 complex | No value | Norway | SV-1 | Opc+ |
| 26053 | N24/99 | Neisseria meningitidis | C | ST-32 complex | No value | Norway | SV-1 | Opc+ |
| 26054 | N71/00 | Neisseria meningitidis | B | ST-32 complex | No value | Norway | SV-1 | Opc+ |
| 26055 | Nimitpol | Neisseria meningitidis | B | ST-32 complex | No value | Thailand | SV-1 | Opc+ |
| 26056 | P15 | Neisseria meningitidis | B | ST-32 complex | No value | Norway | SV-1 | Opc+ |
| 26057 | P28 | Neisseria meningitidis | B | ST-32 complex | No value | Norway | SV-1 | Opc+ |
| 26252 | BB276 | Neisseria meningitidis | NG | ST-213 complex | No value | UK | SV-12 | Opc- |
| 26253 | T97 | Neisseria meningitidis | NG | ST-53 complex | No value | UK | SV-2 | Opc- |
| 26254 | BB123 | Neisseria meningitidis | NG | ST-41/44 complex | No value | UK | SV-2 | Opc+ |
| 26255 | X59 | Neisseria meningitidis | NG | ST-1157 complex | No value | UK | SV-1 | Opc+ |
| 26256 | T45 | Neisseria meningitidis | NG | ST-35 complex | No value | UK | SV-12 | Opc+ |
| 26257 | BB150 | Neisseria meningitidis | NG | No value | No value | UK | SV-5 | Opc- |
| 26258 | X128 | Neisseria meningitidis | NG | ST-865 complex | No value | UK | SV-5 | Opc+ |
| 26259 | T288 | Neisseria meningitidis | NG | ST-23 complex | No value | UK | SV-11 | Opc+ |
| 26260 | T9 | Neisseria meningitidis | NG | ST-269 complex | No value | UK | SV-1 | Opc+ |
| 26261 | T232 | Neisseria meningitidis | NG | ST-254 complex | No value | UK | SV-1 | Opc+ |
| 26262 | R274 | Neisseria meningitidis | NG | ST-254 complex | No value | UK | SV-1 | Opc+ |
| 26263 | V259 | Neisseria meningitidis | NG | ST-53 complex | No value | UK | SV-2 | Opc- |

| | | | | | | | | |
|---|---|---|---|---|---|---|---|---|
| 26264 | V302 | Neisseria meningitidis | NG | ST-60 complex | No value | UK | SV-11 | Opc+ |
| 26265 | T250 | Neisseria meningitidis | NG | No value | No value | UK | SV-7 | Opc+ |
| 26266 | T141 | Neisseria meningitidis | NG | ST-23 complex | No value | UK | SV-11 | Opc+ |
| 26267 | T98 | Neisseria meningitidis | NG | ST-53 complex | No value | UK | SV-2 | Opc- |
| 26268 | BB77 | Neisseria meningitidis | NG | ST-41/44 complex | No value | UK | SV-10 | Opc+ |
| 26269 | BB98 | Neisseria meningitidis | NG | ST-23 complex | No value | UK | SV-11 | Opc+ |
| 26270 | V145 | Neisseria meningitidis | NG | ST-213 complex | No value | UK | SV-12 | Opc- |
| 26271 | BB66 | Neisseria meningitidis | NG | ST-53 complex | No value | UK | SV-2 | Opc- |
| 26272 | T276 | Neisseria meningitidis | NG | ST-213 complex | No value | UK | SV-12 | Opc- |
| 26273 | BB58 | Neisseria meningitidis | NG | ST-22 complex | No value | UK | SV-12 | Opc+ |
| 26274 | v191 | Neisseria meningitidis | NG | ST-53 complex | No value | UK | SV-2 | Opc- |
| 26275 | BB242 | Neisseria meningitidis | NG | ST-22 complex | No value | UK | SV-12 | Opc+ |
| 26276 | V51 | Neisseria meningitidis | NG | No value | No value | UK | SV-11 | Opc+ |
| 26277 | T219 | Neisseria meningitidis | NG | ST-23 complex | No value | UK | SV-11 | Opc+ |
| 26278 | BB122 | Neisseria meningitidis | NG | ST-60 complex | No value | UK | SV-11 | Opc+ |
| 26279 | BB55 | Neisseria meningitidis | NG | ST-1157 complex | No value | UK | SV-1 | Opc+ |
| 26280 | BB43 | Neisseria meningitidis | NG | ST-23 complex | No value | UK | SV-11 | Opc+ |
| 26281 | BB306 | Neisseria meningitidis | NG | ST-269 complex | No value | UK | SV-8 | Opc+ |
| 26282 | BB215 | Neisseria meningitidis | NG | ST-1157 complex | No value | UK | SV-1 | Opc+ |
| 26283 | R121 | Neisseria meningitidis | NG | ST-53 complex | No value | UK | SV-2 | Opc- |
| 26284 | T248 | Neisseria meningitidis | NG | ST-35 complex | No value | UK | SV-12 | Opc+ |
| 26285 | T150 | Neisseria meningitidis | NG | No value | No value | UK | SV-5 | Opc- |
| 26286 | H18-H18 WT | Neisseria meningitidis | NG | ST-32 complex | No value | UK | SV-1 | Opc+ |
| 26287 | V129 | Neisseria meningitidis | NG | ST-22 complex | No value | UK | SV-12 | Opc+ |
| 26288 | BB89 | Neisseria meningitidis | NG | ST-41/44 complex | No value | UK | SV-5 | Opc- |
| 26289 | R150 | Neisseria meningitidis | NG | No value | No value | UK | SV-5 | Opc- |
| 26290 | T200 | Neisseria meningitidis | NG | ST-167 complex | No value | UK | SV-5 | Opc+ |
| 26291 | BB14 | Neisseria meningitidis | NG | ST-23 complex | No value | UK | SV-11 | Opc+ |
| 26292 | BB60 | Neisseria meningitidis | NG | ST-174 complex | No value | UK | SV-1 | Opc+ |
| 26293 | BB238 | Neisseria meningitidis | NG | ST-41/44 complex | No value | UK | SV-6 | Opc+ |
| 26294 | T236 | Neisseria meningitidis | NG | No value | No value | UK | SV-3 | Opc- |
| 26295 | R234 | Neisseria meningitidis | NG | ST-213 complex | No value | UK | SV-12 | Opc- |
| 26296 | BB126 | Neisseria meningitidis | NG | ST-32 complex | No value | UK | SV-3 | Opc+ |
| 26297 | T36 | Neisseria meningitidis | NG | ST-60 complex | No value | UK | SV-11 | Opc+ |
| 26298 | z77 | Neisseria meningitidis | NG | ST-41/44 complex | No value | UK | SV-10 | Opc+ |
| 26299 | x54 | Neisseria meningitidis | NG | ST-213 complex | No value | UK | SV-12 | Opc- |
| 26300 | V268 | Neisseria meningitidis | NG | ST-35 complex | No value | UK | SV-17 | Opc+ |
| 26301 | T193 | Neisseria meningitidis | NG | ST-32 complex | No value | UK | SV-1 | Opc+ |
| 26302 | R62 | Neisseria meningitidis | NG | ST-269 complex | No value | UK | SV-8 | Opc+ |
| 26303 | T287 | Neisseria meningitidis | NG | ST-22 complex | No value | UK | SV-12 | Opc+ |
| 26304 | x301 | Neisseria meningitidis | NG | ST-23 complex | No value | UK | SV-11 | Opc+ |
| 26305 | T62 | Neisseria meningitidis | NG | ST-269 complex | No value | UK | SV-8 | Opc+ |
| 26306 | R279 | Neisseria meningitidis | NG | ST-60 complex | No value | UK | SV-11 | Opc+ |
| 26307 | BB46 | Neisseria meningitidis | NG | ST-23 complex | No value | UK | SV-11 | Opc+ |
| 26308 | x268 | Neisseria meningitidis | NG | ST-60 complex | No value | UK | SV-11 | Opc+ |
| 26309 | BB128 | Neisseria meningitidis | NG | ST-865 complex | No value | UK | SV-5 | Opc+ |
| 26310 | V234 | Neisseria meningitidis | NG | ST-213 complex | No value | UK | SV-12 | Opc- |
| 26311 | V57 | Neisseria meningitidis | NG | ST-41/44 complex | No value | UK | SV-2 | Opc+ |
| 26312 | R46 | Neisseria meningitidis | NG | ST-23 complex | No value | UK | SV-11 | Opc+ |
| 26313 | R283 | Neisseria meningitidis | NG | ST-1157 complex | No value | UK | SV-1 | Opc+ |
| 26314 | z54 | Neisseria meningitidis | NG | ST-60 complex | No value | UK | SV-11 | Opc+ |
| 26315 | X241 | Neisseria meningitidis | NG | ST-11 complex | No value | UK | SV-2 | Opc- |
| 26316 | R242 | Neisseria meningitidis | NG | No value | No value | UK | SV-1 | Opc+ |
| 26317 | R34 | Neisseria meningitidis | NG | ST-213 complex | No value | UK | SV-12 | Opc- |
| 26318 | BB301 | Neisseria meningitidis | NG | ST-23 complex | No value | UK | SV-11 | Opc+ |
| 26319 | v59 | Neisseria meningitidis | NG | ST-1157 complex | No value | UK | SV-1 | Opc+ |
| 26320 | R136 | Neisseria meningitidis | NG | ST-53 complex | No value | UK | SV-2 | Opc- |
| 26321 | z43 | Neisseria meningitidis | NG | ST-23 complex | No value | UK | SV-11 | Opc+ |
| 26322 | R49 | Neisseria meningitidis | NG | ST-103 complex | No value | UK | SV-5 | Opc+ |
| 26323 | T59 | Neisseria meningitidis | NG | ST-1157 complex | No value | UK | SV-1 | Opc+ |
| 26324 | R22 | Neisseria meningitidis | NG | ST-1157 complex | No value | UK | SV-1 | Opc+ |
| 26325 | x83 | Neisseria meningitidis | NG | ST-53 complex | No value | UK | SV-2 | Opc- |
| 26326 | T304 | Neisseria meningitidis | NG | ST-53 complex | No value | UK | SV-2 | Opc- |
| 26327 | R122 | Neisseria meningitidis | NG | ST-60 complex | No value | UK | SV-11 | Opc+ |
| 26328 | BB156 | Neisseria meningitidis | NG | ST-198 complex | No value | UK | SV-4 | Opc+ |
| 26329 | BB130 | Neisseria meningitidis | NG | ST-60 complex | No value | UK | SV-11 | Opc+ |
| 26331 | BB44 | Neisseria meningitidis | NG | ST-41/44 complex | No value | UK | SV-10 | Opc+ |
| 26332 | BB111 | Neisseria meningitidis | NG | ST-461 complex | No value | UK | SV-1 | Opc- |
| 26333 | R176 | Neisseria meningitidis | NG | ST-41/44 complex | No value | UK | SV-2 | Opc+ |
| 26334 | V196 | Neisseria meningitidis | NG | ST-269 complex | No value | UK | SV-8 | Opc+ |
| 26335 | R310 | Neisseria meningitidis | NG | ST-23 complex | No value | UK | SV-11 | Opc+ |
| 26336 | v216 | Neisseria meningitidis | NG | ST-53 complex | No value | UK | SV-2 | Opc- |
| 26337 | BB62 | Neisseria meningitidis | NG | ST-269 complex | No value | UK | SV-8 | Opc+ |
| 26338 | T142 | Neisseria meningitidis | NG | ST-213 complex | No value | UK | SV-12 | Opc- |

| | | | | | | | | |
|---|---|---|---|---|---|---|---|---|
| 26339 | BB241 | Neisseria meningitidis | NG | ST-11 complex | No value | UK | SV-2 | Opc- |
| 26340 | V128 | Neisseria meningitidis | NG | ST-865 complex | No value | UK | SV-5 | Opc+ |
| 26341 | BB185 | Neisseria meningitidis | NG | ST-1117 complex | No value | UK | SV-10 | Opc- |
| 26342 | V34 | Neisseria meningitidis | NG | ST-23 complex | No value | UK | SV-11 | Opc+ |
| 26343 | BB85 | Neisseria meningitidis | NG | ST-23 complex | No value | UK | SV-11 | Opc+ |
| 26344 | R243 | Neisseria meningitidis | NG | ST-53 complex | No value | UK | SV-2 | Opc- |
| 26345 | T66 | Neisseria meningitidis | NG | ST-23 complex | No value | UK | SV-11 | Opc+ |
| 26346 | BB148 | Neisseria meningitidis | NG | ST-23 complex | No value | UK | SV-11 | Opc+ |
| 26348 | R92 | Neisseria meningitidis | NG | ST-269 complex | No value | UK | SV-8 | Opc+ |
| 26349 | BB279 | Neisseria meningitidis | NG | ST-60 complex | No value | UK | SV-11 | Opc+ |
| 26350 | T3 | Neisseria meningitidis | NG | ST-53 complex | No value | UK | SV-2 | Opc- |
| 26351 | BB107 | Neisseria meningitidis | NG | ST-1136 complex | No value | UK | SV-2 | Opc+ |
| 26352 | BB36 | Neisseria meningitidis | NG | ST-60 complex | No value | UK | SV-11 | Opc+ |
| 26353 | BB64 | Neisseria meningitidis | NG | ST-41/44 complex | No value | UK | SV-10 | Opc+ |
| 26354 | T117 | Neisseria meningitidis | NG | ST-23 complex | No value | UK | SV-11 | Opc+ |
| 26355 | R263 | Neisseria meningitidis | NG | ST-53 complex | No value | UK | SV-2 | Opc- |
| 26356 | T234 | Neisseria meningitidis | NG | ST-213 complex | No value | UK | SV-12 | Opc- |
| 26357 | T233 | Neisseria meningitidis | NG | No value | No value | UK | SV-4 | Opc+ |
| 26358 | T242 | Neisseria meningitidis | NG | No value | No value | UK | SV-1 | Opc+ |
| 26359 | R146 | Neisseria meningitidis | NG | ST-23 complex | No value | UK | SV-8 | Opc+ |
| 26360 | R221 | Neisseria meningitidis | NG | ST-60 complex | No value | UK | SV-2 | Opc+ |
| 26361 | BB45 | Neisseria meningitidis | NG | ST-35 complex | No value | UK | SV-5 | Opc+ |
| 26362 | R156 | Neisseria meningitidis | NG | ST-198 complex | No value | UK | SV-12 | Opc+ |
| 26363 | T79 | Neisseria meningitidis | NG | ST-269 complex | No value | UK | SV-1 | Opc+ |
| 26364 | BB196 | Neisseria meningitidis | NG | ST-269 complex | No value | UK | SV-1 | Opc+ |
| 26365 | z191 | Neisseria meningitidis | NG | ST-53 complex | No value | UK | SV-12 | Opc- |
| 26366 | R128 | Neisseria meningitidis | NG | ST-865 complex | No value | UK | SV-5 | Opc+ |
| 26367 | BB51 | Neisseria meningitidis | NG | ST-213 complex | No value | UK | SV-12 | Opc- |
| 26368 | BB298 | Neisseria meningitidis | NG | ST-1157 complex | No value | UK | SV-1 | Opc+ |
| 26369 | T274 | Neisseria meningitidis | NG | ST-254 complex | No value | UK | SV-1 | Opc+ |
| 26370 | R129 | Neisseria meningitidis | NG | ST-22 complex | No value | UK | SV-12 | Opc+ |
| 26371 | R10 | Neisseria meningitidis | NG | ST-23 complex | No value | UK | SV-11 | Opc+ |
| 26372 | V304 | Neisseria meningitidis | NG | ST-53 complex | No value | UK | SV-2 | Opc- |
| 26373 | R145 | Neisseria meningitidis | NG | ST-213 complex | No value | UK | SV-12 | Opc- |
| 26374 | Z126 | Neisseria meningitidis | NG | ST-32 complex | No value | UK | SV-1 | Opc+ |
| 26375 | R236 | Neisseria meningitidis | NG | No value | No value | UK | SV-3 | Opc- |
| 26376 | R44 | Neisseria meningitidis | NG | ST-198 complex | No value | UK | SV-7 | Opc+ |
| 26377 | BB255 | Neisseria meningitidis | NG | ST-213 complex | No value | UK | SV-12 | Opc- |
| 26378 | BB291 | Neisseria meningitidis | NG | ST-41/44 complex | No value | UK | SV-2 | Opc+ |
| 26379 | R193 | Neisseria meningitidis | NG | ST-32 complex | No value | UK | SV-2 | Opc+ |
| 26381 | R210 | Neisseria meningitidis | NG | ST-22 complex | No value | UK | SV-11 | Opc+ |
| 26382 | X136 | Neisseria meningitidis | NG | ST-53 complex | No value | UK | SV-2 | Opc- |
| 26383 | R216 | Neisseria meningitidis | NG | ST-53 complex | No value | UK | SV-11 | Opc- |
| 26384 | BB262 | Neisseria meningitidis | NG | ST-23 complex | No value | UK | SV-2 | Opc+ |
| 26385 | BB176 | Neisseria meningitidis | NG | ST-1157 complex | No value | UK | SV-2 | Opc+ |
| 26386 | X309 | Neisseria meningitidis | NG | ST-23 complex | No value | UK | SV-11 | Opc+ |
| 26387 | Z242 | Neisseria meningitidis | NG | No value | No value | UK | SV-1 | Opc+ |
| 26388 | V242 | Neisseria meningitidis | NG | No value | No value | UK | SV-1 | Opc+ |
| 26389 | X43 | Neisseria meningitidis | NG | ST-23 complex | No value | UK | SV-11 | Opc+ |
| 26390 | BB210 | Neisseria meningitidis | NG | ST-22 complex | No value | UK | SV-12 | Opc+ |
| 26391 | R186 | Neisseria meningitidis | NG | ST-213 complex | No value | UK | SV-12 | Opc- |
| 26392 | T56 | Neisseria meningitidis | NG | No value | No value | UK | SV-11 | Opc+ |
| 26393 | v18 | Neisseria meningitidis | NG | ST-23 complex | No value | UK | SV-11 | Opc+ |
| 26394 | T121 | Neisseria meningitidis | NG | ST-53 complex | No value | UK | SV-2 | Opc- |
| 26395 | R196 | Neisseria meningitidis | NG | ST-269 complex | No value | UK | SV-8 | Opc+ |
| 26396 | T300 | Neisseria meningitidis | NG | ST-23 complex | No value | UK | SV-11 | Opc+ |
| 26397 | T5 | Neisseria meningitidis | NG | ST-1157 complex | No value | UK | SV-1 | Opc+ |
| 26398 | R104 | Neisseria meningitidis | NG | ST-23 complex | No value | UK | SV-11 | Opc+ |
| 26399 | R155 | Neisseria meningitidis | NG | ST-53 complex | No value | UK | SV-2 | Opc- |
| 26400 | R39 | Neisseria meningitidis | NG | ST-23 complex | No value | UK | SV-11 | Opc+ |
| 26401 | V54 | Neisseria meningitidis | NG | ST-213 complex | No value | UK | SV-12 | Opc- |
| 26402 | R83 | Neisseria meningitidis | NG | ST-53 complex | No value | UK | SV-2 | Opc- |
| 26403 | z10 | Neisseria meningitidis | NG | ST-23 complex | No value | UK | SV-11 | Opc+ |
| 26404 | BB18 | Neisseria meningitidis | NG | ST-178 complex | No value | UK | SV-1 | Opc- |
| 26405 | BB232 | Neisseria meningitidis | NG | ST-254 complex | No value | UK | SV-1 | Opc+ |
| 26407 | BB49 | Neisseria meningitidis | NG | ST-213 complex | No value | UK | SV-11 | Opc- |
| 26408 | BB287 | Neisseria meningitidis | NG | ST-22 complex | No value | UK | SV-12 | Opc+ |
| 26409 | R222 | Neisseria meningitidis | NG | ST-60 complex | No value | UK | SV-12 | Opc+ |
| 26410 | R306 | Neisseria meningitidis | NG | ST-269 complex | No value | UK | SV-11 | Opc+ |
| 26411 | R36 | Neisseria meningitidis | NG | ST-60 complex | No value | UK | SV-8 | Opc+ |
| 26412 | BB219 | Neisseria meningitidis | NG | ST-23 complex | No value | UK | SV-11 | Opc+ |
| 26413 | V79 | Neisseria meningitidis | NG | ST-269 complex | No value | UK | SV-11 | Opc+ |
| 26414 | R4 | Neisseria meningitidis | NG | ST-60 complex | No value | UK | SV-1 | Opc+ |
| 26415 | T191 | Neisseria meningitidis | NG | ST-53 complex | No value | UK | SV-11 | Opc- |

| | | | | | | | | |
|---|---|---|---|---|---|---|---|---|
| 26416 | T306 | Neisseria meningitidis | NG | ST-269 complex | No value | UK | SV-7 | Opc+ |
| 26417 | R105 | Neisseria meningitidis | NG | ST-269 complex | No value | UK | SV-1 | Opc+ |
| 26418 | x222 | Neisseria meningitidis | NG | ST-60 complex | No value | UK | SV-11 | Opc+ |
| 26419 | T119 | Neisseria meningitidis | NG | ST-53 complex | No value | UK | SV-2 | Opc- |
| 26420 | z49 | Neisseria meningitidis | NG | ST-213 complex | No value | UK | SV-12 | Opc- |
| 26421 | X22 | Neisseria meningitidis | NG | ST-213 complex | No value | UK | SV-12 | Opc- |
| 26422 | T107 | Neisseria meningitidis | NG | ST-1136 complex | No value | UK | SV-2 | Opc+ |
| 26423 | R141 | Neisseria meningitidis | NG | ST-23 complex | No value | UK | SV-11 | Opc+ |
| 26424 | T310 | Neisseria meningitidis | NG | ST-23 complex | No value | UK | SV-11 | Opc+ |
| 26425 | R256 | Neisseria meningitidis | NG | ST-60 complex | No value | UK | SV-11 | Opc+ |
| 26426 | T130 | Neisseria meningitidis | NG | ST-167 complex | No value | UK | SV-5 | Opc+ |
| 26427 | T298 | Neisseria meningitidis | NG | ST-1157 complex | No value | UK | SV-1 | Opc+ |
| 26428 | T22 | Neisseria meningitidis | NG | ST-1157 complex | No value | UK | SV-1 | Opc+ |
| 26429 | Z301 | Neisseria meningitidis | NG | ST-23 complex | No value | UK | SV-11 | Opc+ |
| 26430 | T39 | Neisseria meningitidis | NG | ST-23 complex | No value | UK | SV-11 | Opc+ |
| 26431 | R300 | Neisseria meningitidis | NG | ST-23 complex | No value | UK | SV-11 | Opc+ |
| 26432 | T210 | Neisseria meningitidis | NG | ST-22 complex | No value | UK | SV-12 | Opc+ |
| 26433 | T57 | Neisseria meningitidis | NG | ST-41/44 complex | No value | UK | SV-2 | Opc+ |
| 26434 | T302 | Neisseria meningitidis | NG | ST-60 complex | No value | UK | SV-11 | Opc+ |
| 26435 | R200 | Neisseria meningitidis | NG | ST-167 complex | No value | UK | SV-5 | Opc+ |
| 26436 | BB56 | Neisseria meningitidis | NG | No value | No value | UK | SV-11 | Opc+ |
| 26437 | BB204 | Neisseria meningitidis | NG | ST-23 complex | No value | UK | SV-11 | Opc+ |
| 26438 | BB24 | Neisseria meningitidis | NG | No value | No value | UK | SV-7 | Opc+ |
| 26439 | BB5 | Neisseria meningitidis | NG | ST-1157 complex | No value | UK | SV-1 | Opc+ |
| 26440 | X18 | Neisseria meningitidis | NG | ST-35 complex | No value | UK | SV-12 | Opc+ |
| 26441 | BB59 | Neisseria meningitidis | NG | ST-1157 complex | No value | UK | SV-1 | Opc+ |
| 26442 | Z270 | Neisseria meningitidis | NG | ST-23 complex | No value | UK | SV-11 | Opc+ |
| 26443 | R142 | Neisseria meningitidis | NG | ST-213 complex | No value | UK | SV-12 | Opc- |
| 26444 | R97 | Neisseria meningitidis | NG | ST-53 complex | No value | UK | SV-2 | Opc- |
| 26445 | Z119 | Neisseria meningitidis | NG | ST-53 complex | No value | UK | SV-2 | Opc- |
| 26446 | T270 | Neisseria meningitidis | NG | ST-23 complex | No value | UK | SV-11 | Opc+ |
| 26447 | R5 | Neisseria meningitidis | NG | ST-1157 complex | No value | UK | SV-1 | Opc+ |
| 26448 | R148 | Neisseria meningitidis | NG | ST-23 complex | No value | UK | SV-11 | Opc+ |
| 26449 | Z55 | Neisseria meningitidis | NG | ST-1157 complex | No value | UK | SV-1 | Opc+ |
| 26450 | BB159 | Neisseria meningitidis | NG | ST-60 complex | No value | UK | SV-11 | Opc+ |
| 26451 | r119 | Neisseria meningitidis | NG | ST-53 complex | No value | UK | SV-2 | Opc- |
| 26452 | H18-H18-01 | Neisseria meningitidis | NG | ST-32 complex | No value | UK | SV-1 | Opc+ |
| 26453 | z146 | Neisseria meningitidis | NG | ST-23 complex | No value | UK | SV-11 | Opc+ |
| 26454 | R233 | Neisseria meningitidis | NG | ST-198 complex | No value | UK | SV-4 | Opc+ |
| 26455 | C2 | Neisseria meningitidis | NG | ST-32 complex | No value | UK | SV-1 | Opc+ |
| 26456 | BB22 | Neisseria meningitidis | NG | ST-213 complex | No value | UK | SV-12 | Opc- |
| 26457 | R191 | Neisseria meningitidis | NG | ST-53 complex | No value | UK | SV-2 | Opc- |
| 26458 | BB233 | Neisseria meningitidis | NG | ST-198 complex | No value | UK | SV-4 | Opc+ |
| 26459 | T128 | Neisseria meningitidis | NG | ST-865 complex | No value | UK | SV-5 | Opc+ |
| 26460 | T196 | Neisseria meningitidis | NG | ST-269 complex | No value | UK | SV-8 | Opc+ |
| 26461 | Z300 | Neisseria meningitidis | NG | ST-23 complex | No value | UK | SV-11 | Opc+ |
| 26462 | BB141 | Neisseria meningitidis | NG | ST-23 complex | No value | UK | SV-11 | Opc+ |
| 26463 | R291 | Neisseria meningitidis | NG | ST-41/44 complex | No value | UK | SV-6 | Opc+ |
| 26464 | Z221 | Neisseria meningitidis | NG | ST-60 complex | No value | UK | SV-11 | Opc+ |
| 26465 | R248 | Neisseria meningitidis | NG | ST-213 complex | No value | UK | SV-12 | Opc- |
| 26466 | X119 | Neisseria meningitidis | NG | ST-53 complex | No value | UK | SV-2 | Opc- |
| 26467 | BB202 | Neisseria meningitidis | NG | ST-213 complex | No value | UK | SV-1 | Opc- |
| 26468 | Z234 | Neisseria meningitidis | NG | ST-213 complex | No value | UK | SV-12 | Opc- |
| 26469 | BB52 | Neisseria meningitidis | NG | ST-41/44 complex | No value | UK | SV-6 | Opc+ |
| 26470 | z196 | Neisseria meningitidis | NG | ST-269 complex | No value | UK | SV-8 | Opc+ |
| 26471 | T179 | Neisseria meningitidis | NG | ST-23 complex | No value | UK | SV-11 | Opc+ |
| 26472 | BB9 | Neisseria meningitidis | NG | ST-269 complex | No value | UK | SV-1 | Opc+ |
| 26473 | R45 | Neisseria meningitidis | NG | ST-35 complex | No value | UK | SV-12 | Opc+ |
| 26474 | T216 | Neisseria meningitidis | NG | ST-53 complex | No value | UK | SV-2 | Opc- |
| 26475 | R59 | Neisseria meningitidis | NG | ST-1157 complex | No value | UK | SV-1 | Opc+ |
| 26476 | R58 | Neisseria meningitidis | NG | ST-22 complex | No value | UK | SV-12 | Opc+ |
| 26477 | X234 | Neisseria meningitidis | NG | ST-213 complex | No value | UK | SV-12 | Opc- |
| 26480 | z59 | Neisseria meningitidis | NG | ST-1157 complex | No value | UK | SV-1 | Opc+ |
| 26481 | T44 | Neisseria meningitidis | NG | ST-198 complex | No value | UK | SV-7 | Opc+ |
| 26482 | BB90 | Neisseria meningitidis | NG | ST-865 complex | No value | UK | SV-5 | Opc+ |
| 26483 | BB136 | Neisseria meningitidis | NG | ST-198 complex | No value | UK | SV-4 | Opc+ |
| 26484 | R89 | Neisseria meningitidis | NG | ST-41/44 complex | No value | UK | SV-5 | Opc- |
| 26485 | R304 | Neisseria meningitidis | NG | ST-41/44 complex | No value | UK | SV-6 | Opc+ |
| 26486 | BB274 | Neisseria meningitidis | NG | ST-254 complex | No value | UK | SV-1 | Opc+ |
| 26487 | X270 | Neisseria meningitidis | NG | ST-23 complex | No value | UK | SV-11 | Opc+ |
| 26488 | R276 | Neisseria meningitidis | NG | ST-213 complex | No value | UK | SV-12 | Opc- |
| 26489 | R167 | Neisseria meningitidis | NG | ST-23 complex | No value | UK | SV-11 | Opc+ |
| 26490 | z64 | Neisseria meningitidis | NG | ST-41/44 complex | No value | UK | SV-10 | Opc+ |
| 26491 | v306 | Neisseria meningitidis | NG | ST-269 complex | No value | UK | SV-8 | Opc+ |

| | | | | | | | | |
|---|---|---|---|---|---|---|---|---|
| 26492 | T291 | Neisseria meningitidis | NG | ST-41/44 complex | No value | UK | SV-6 | Opc+ |
| 26493 | z34 | Neisseria meningitidis | NG | ST-213 complex | No value | UK | SV-12 | Opc- |
| 26494 | R255 | Neisseria meningitidis | NG | No value | No value | UK | SV-12 | Opc- |
| 26495 | BB218 | Neisseria meningitidis | NG | ST-198 complex | No value | UK | SV-4 | Opc+ |
| 26496 | R262 | Neisseria meningitidis | NG | ST-23 complex | No value | UK | SV-11 | Opc+ |
| 26497 | z139 | Neisseria meningitidis | NG | ST-254 complex | No value | UK | SV-1 | Opc+ |
| 26498 | T279 | Neisseria meningitidis | NG | ST-60 complex | No value | UK | SV-11 | Opc+ |
| 26499 | V22 | Neisseria meningitidis | NG | ST-213 complex | No value | UK | SV-12 | Opc- |
| 26500 | BB83 | Neisseria meningitidis | NG | ST-53 complex | No value | UK | SV-2 | Opc- |
| 26501 | BB186 | Neisseria meningitidis | NG | ST-23 complex | No value | UK | SV-11 | Opc+ |
| 26502 | BB3 | Neisseria meningitidis | NG | ST-11 complex | No value | UK | SV-2 | Opc- |
| 26503 | R54 | Neisseria meningitidis | NG | ST-213 complex | No value | UK | SV-12 | Opc- |
| 26504 | V125 | Neisseria meningitidis | NG | ST-35 complex | No value | UK | SV-16 | Opc+ |
| 26505 | R123 | Neisseria meningitidis | NG | ST-23 complex | No value | UK | SV-11 | Opc+ |
| 26506 | BB34 | Neisseria meningitidis | NG | ST-213 complex | No value | UK | SV-12 | Opc- |
| 26507 | Z46 | Neisseria meningitidis | NG | ST-23 complex | No value | UK | SV-11 | Opc+ |
| 26508 | R66 | Neisseria meningitidis | NG | ST-23 complex | No value | UK | SV-11 | Opc+ |
| 26509 | Z255 | Neisseria meningitidis | NG | ST-213 complex | No value | UK | SV-12 | Opc- |
| 26510 | R117 | Neisseria meningitidis | NG | ST-23 complex | No value | UK | SV-11 | Opc+ |
| 26511 | x210 | Neisseria meningitidis | NG | ST-23 complex | No value | UK | SV-11 | Opc+ |
| 26512 | V241 | Neisseria meningitidis | NG | ST-11 complex | No value | UK | SV-2 | Opc- |
| 26513 | BB283 | Neisseria meningitidis | NG | ST-1157 complex | No value | UK | SV-1 | Opc+ |
| 26514 | BB195 | Neisseria meningitidis | NG | No value | No value | UK | SV-1 | Opc+ |
| 26515 | BB54 | Neisseria meningitidis | NG | ST-60 complex | No value | UK | SV-11 | Opc+ |
| 26516 | R79 | Neisseria meningitidis | NG | ST-269 complex | No value | UK | SV-1 | Opc+ |
| 26517 | X300 | Neisseria meningitidis | NG | ST-23 complex | No value | UK | SV-11 | Opc+ |
| 26518 | v119 | Neisseria meningitidis | NG | ST-53 complex | No value | UK | SV-2 | Opc- |
| 26519 | T53 | Neisseria meningitidis | NG | ST-60 complex | No value | UK | SV-11 | Opc+ |
| 26520 | T4 | Neisseria meningitidis | NG | ST-60 complex | No value | UK | SV-11 | Opc+ |
| 26521 | BB234 | Neisseria meningitidis | NG | ST-213 complex | No value | UK | SV-12 | Opc- |
| 26522 | x121 | Neisseria meningitidis | NG | ST-53 complex | No value | UK | SV-2 | Opc- |
| 26523 | T156 | Neisseria meningitidis | NG | ST-198 complex | No value | UK | SV-4 | Opc+ |
| 26524 | BB181 | Neisseria meningitidis | NG | ST-178 complex | No value | UK | SV-1 | Opc- |
| 26525 | BB110 | Neisseria meningitidis | NG | ST-23 complex | No value | UK | SV-11 | Opc+ |
| 26526 | z18 | Neisseria meningitidis | NG | ST-178 complex | No value | UK | SV-1 | Opc- |
| 26527 | BB39 | Neisseria meningitidis | NG | ST-23 complex | No value | UK | SV-11 | Opc+ |
| 26528 | R270 | Neisseria meningitidis | NG | ST-23 complex | No value | UK | SV-12 | Opc+ |
| 26529 | BB228 | Neisseria meningitidis | NG | ST-53 complex | No value | UK | SV-2 | Opc- |
| 26541 | 2851 | Neisseria meningitidis | NG | ST-32 complex | No value | Norway | SV-1 | Opc+ |
| 26542 | 3043 | Neisseria meningitidis | NG | ST-32 complex | No value | Norway | SV-1 | Opc+ |
| 26543 | 3207 | Neisseria meningitidis | NG | ST-32 complex | No value | Norway | SV-1 | Opc+ |
| 26544 | 3311 | Neisseria meningitidis | NG | ST-32 complex | No value | Norway | SV-1 | Opc+ |
| 26712 | NM233 | Neisseria meningitidis | Y | ST-23 complex | No value | USA | SV-11 | Opc+ |
| 26713 | NM220 | Neisseria meningitidis | Y | ST-23 complex | No value | USA | SV-11 | Opc+ |
| 26731 | M13 240559 | Neisseria meningitidis | C | ST-11 complex | No value | UK | SV-2 | Opc- |
| 26732 | M13 240559 | Neisseria meningitidis | C | ST-11 complex | No value | UK | SV-2 | Opc- |
| 26733 | LNP27256 | Neisseria meningitidis | C | ST-11 complex | Epidemic | France | SV-2 | Opc- |
| 26821 | LNP27257 | Neisseria meningitidis | C | ST-11 complex | Epidemic | France | SV-2 | Opc- |
| 26822 | LNP26948 | Neisseria meningitidis | C | ST-11 complex | Sporadic case | France | SV-2 | Opc- |
| 26823 | 12024_2010 | Neisseria meningitidis | B | ST-41/44 complex | No value | Ireland | SV-2 | Opc+ |
| 26824 | 12025_2010 | Neisseria meningitidis | C | ST-11 complex | No value | Ireland | SV-2 | Opc- |
| 26825 | 12026_2010 | Neisseria meningitidis | C | No value | No value | Ireland | SV-2 | Opc+ |
| 26826 | 12027_2010 | Neisseria meningitidis | B | ST-213 complex | No value | Ireland | SV-12 | Opc- |
| 26828 | 12029_2010 | Neisseria meningitidis | B | ST-32 complex | No value | Ireland | SV-1 | Opc+ |
| 26829 | 12030_2010 | Neisseria meningitidis | B | ST-60 complex | No value | Ireland | SV-11 | Opc+ |
| 26830 | 12031_2010 | Neisseria meningitidis | B | ST-60 complex | No value | Ireland | SV-11 | Opc+ |
| 26831 | 12032_2010 | Neisseria meningitidis | B | ST-41/44 complex | No value | Ireland | SV-2 | Opc+ |
| 26832 | 12033_2010 | Neisseria meningitidis | B | ST-41/44 complex | No value | Ireland | SV-2 | Opc+ |
| 26833 | 12034_2010 | Neisseria meningitidis | B | ST-41/44 complex | No value | Ireland | SV-2 | Opc+ |
| 26834 | 12035_2010 | Neisseria meningitidis | B | ST-41/44 complex | No value | Ireland | SV-2 | Opc+ |
| 26835 | 12001_2011 | Neisseria meningitidis | B | ST-269 complex | No value | Ireland | SV-1 | Opc+ |
| 26836 | 12003_2011 | Neisseria meningitidis | B | ST-41/44 complex | No value | Ireland | SV-6 | Opc+ |
| 26837 | 12002_2011 | Neisseria meningitidis | B | ST-41/44 complex | No value | Ireland | SV-2 | Opc+ |
| 26838 | 12004_2011 | Neisseria meningitidis | B | ST-41/44 complex | No value | Ireland | SV-2 | Opc+ |
| 26839 | 12005_2011 | Neisseria meningitidis | B | ST-461 complex | No value | Ireland | SV-1 | Opc- |
| 26840 | 12006_2011 | Neisseria meningitidis | B | ST-41/44 complex | No value | Ireland | SV-6 | Opc+ |
| 26841 | 12007_2011 | Neisseria meningitidis | E | ST-1157 complex | No value | Ireland | SV-1 | Opc+ |
| 26842 | 12008_2011 | Neisseria meningitidis | B | ST-41/44 complex | No value | Ireland | SV-2 | Opc+ |
| 26843 | 12009_2011 | Neisseria meningitidis | B | ST-41/44 complex | No value | Ireland | SV-2 | Opc+ |
| 26844 | 12010_2011 | Neisseria meningitidis | B | ST-269 complex | No value | Ireland | SV-1 | Opc+ |
| 26845 | 12016_2011 | Neisseria meningitidis | B | ST-269 complex | No value | Ireland | SV-8 | Opc+ |
| 26846 | 12017_2011 | Neisseria meningitidis | B | ST-41/44 complex | No value | Ireland | SV-2 | Opc+ |
| 26847 | 12018_2011 | Neisseria meningitidis | B | ST-461 complex | No value | Ireland | SV-6 | Opc- |
| 26848 | 12019_2011 | Neisseria meningitidis | B | ST-41/44 complex | No value | Ireland | SV-6 | Opc+ |

| | | | | | | | | |
|---|---|---|---|---|---|---|---|---|
| 26849 | 12020_2011 | Neisseria meningitidis | B | ST-41/44 complex | No value | Ireland | SV-2 | Opc+ |
| 26850 | 12021_2011 | Neisseria meningitidis | B | ST-32 complex | No value | Ireland | SV-1 | Opc+ |
| 26851 | 12022_2011 | Neisseria meningitidis | B | ST-269 complex | No value | Ireland | SV-8 | Opc+ |
| 26852 | 12011_2011 | Neisseria meningitidis | B | No value | No value | Ireland | SV-8 | Opc+ |
| 26853 | 12023_2011 | Neisseria meningitidis | C | ST-269 complex | No value | Ireland | SV-8 | Opc+ |
| 26854 | 12024_2011 | Neisseria meningitidis | B | No value | No value | Ireland | SV-8 | Opc+ |
| 26855 | 12025_2011 | Neisseria meningitidis | B | ST-18 complex | No value | Ireland | SV-7 | Opc- |
| 26856 | 12026_2011 | Neisseria meningitidis | B | ST-32 complex | No value | Ireland | SV-3 | Opc+ |
| 26857 | 12027_2011 | Neisseria meningitidis | B | ST-35 complex | No value | Ireland | SV-12 | Opc+ |
| 26858 | 12028_2011 | Neisseria meningitidis | B | No value | No value | Ireland | SV-16 | Opc+ |
| 26859 | 12029_2011 | Neisseria meningitidis | C | ST-11 complex | No value | Ireland | SV-2 | Opc- |
| 26860 | 12030_2011 | Neisseria meningitidis | B | No value | No value | Ireland | SV-1 | Opc+ |
| 26861 | 12031_2011 | Neisseria meningitidis | B | ST-41/44 complex | No value | Ireland | SV-2 | Opc+ |
| 26862 | 12032_2011 | Neisseria meningitidis | B | ST-41/44 complex | No value | Ireland | SV-2 | Opc+ |
| 26863 | 12033_2011 | Neisseria meningitidis | B | ST-162 complex | No value | Ireland | SV-1 | Opc+ |
| 26864 | 12001_2012 | Neisseria meningitidis | B | ST-41/44 complex | No value | Ireland | SV-2 | Opc+ |
| 26865 | 12002_2012 | Neisseria meningitidis | B | ST-41/44 complex | No value | Ireland | SV-6 | Opc+ |
| 26866 | 12003_2012 | Neisseria meningitidis | B | ST-41/44 complex | No value | Ireland | SV-6 | Opc+ |
| 26867 | 12004_2012 | Neisseria meningitidis | B | ST-41/44 complex | No value | Ireland | SV-2 | Opc+ |
| 26868 | 12005_2012 | Neisseria meningitidis | B | ST-269 complex | No value | Ireland | SV-1 | Opc+ |
| 26869 | 12006_2012 | Neisseria meningitidis | B | ST-269 complex | No value | Ireland | SV-1 | Opc+ |
| 26872 | 12009_2012 | Neisseria meningitidis | Y | ST-174 complex | No value | Ireland | SV-1 | Opc+ |
| 26874 | 12011_2012 | Neisseria meningitidis | B | ST-32 complex | No value | Ireland | SV-1 | Opc+ |
| 26875 | 12012_2012 | Neisseria meningitidis | B | ST-41/44 complex | No value | Ireland | SV-10 | Opc+ |
| 26877 | 12014_2012 | Neisseria meningitidis | B | ST-162 complex | No value | Ireland | SV-1 | Opc+ |
| 26878 | 12018_2012 | Neisseria meningitidis | B | No value | No value | Ireland | SV-8 | Opc+ |
| 26879 | 12019_2012 | Neisseria meningitidis | B | ST-269 complex | No value | Ireland | SV-1 | Opc+ |
| 26880 | 12020_2012 | Neisseria meningitidis | B | ST-213 complex | No value | Ireland | SV-12 | Opc- |
| 26881 | 12021_2012 | Neisseria meningitidis | B | ST-8 complex | No value | Ireland | SV-10 | Opc- |
| 26882 | 12022_2012 | Neisseria meningitidis | B | ST-8 complex | No value | Ireland | SV-10 | Opc- |
| 26883 | 12023_2012 | Neisseria meningitidis | Y | ST-23 complex | No value | Ireland | SV-11 | Opc+ |
| 26884 | 12024_2012 | Neisseria meningitidis | B | ST-41/44 complex | No value | Ireland | SV-6 | Opc+ |
| 26885 | 12025_2012 | Neisseria meningitidis | B | ST-269 complex | No value | Ireland | SV-8 | Opc+ |
| 26886 | 12026_2012 | Neisseria meningitidis | B | ST-41/44 complex | No value | Ireland | SV-6 | Opc+ |
| 26887 | 12028_2012 | Neisseria meningitidis | B | ST-103 complex | No value | Ireland | SV-5 | Opc+ |
| 26888 | 12029_2012 | Neisseria meningitidis | B | ST-22 complex | No value | Ireland | SV-12 | Opc+ |
| 26889 | 12030_2012 | Neisseria meningitidis | B | No value | No value | Ireland | SV-8 | Opc+ |
| 26890 | 12031_2012 | Neisseria meningitidis | B | ST-41/44 complex | No value | Ireland | SV-6 | Opc+ |
| 26891 | 12032_2012 | Neisseria meningitidis | B | ST-35 complex | No value | Ireland | SV-16 | Opc+ |
| 26892 | 12033_2012 | Neisseria meningitidis | Y | ST-23 complex | No value | Ireland | SV-11 | Opc+ |
| 26893 | 12036_2012 | Neisseria meningitidis | B | ST-41/44 complex | No value | Ireland | SV-6 | Opc+ |
| 26894 | 12037_2012 | Neisseria meningitidis | B | ST-1157 complex | No value | Ireland | SV-1 | Opc+ |
| 26895 | 12040_2012 | Neisseria meningitidis | B | ST-269 complex | No value | Ireland | SV-1 | Opc+ |
| 26896 | 12041_2012 | Neisseria meningitidis | B | ST-213 complex | No value | Ireland | SV-12 | Opc- |
| 26897 | 12001_2013 | Neisseria meningitidis | B | ST-41/44 complex | No value | Ireland | SV-2 | Opc+ |
| 26898 | 12002_2013 | Neisseria meningitidis | W | ST-11 complex | No value | Ireland | SV-2 | Opc- |
| 26899 | 12003_2013 | Neisseria meningitidis | W | ST-11 complex | No value | Ireland | SV-2 | Opc- |
| 26900 | 12004_2013 | Neisseria meningitidis | W | ST-22 complex | No value | Ireland | SV-12 | Opc+ |
| 26901 | 12005_2013 | Neisseria meningitidis | B | ST-41/44 complex | No value | Ireland | SV-2 | Opc+ |
| 26902 | 12006_2013 | Neisseria meningitidis | Y | ST-174 complex | No value | Ireland | SV-1 | Opc+ |
| 26903 | 12007_2013 | Neisseria meningitidis | B | No value | No value | Ireland | SV-12 | Opc+ |
| 26904 | 12008_2013 | Neisseria meningitidis | B | ST-41/44 complex | No value | Ireland | SV-2 | Opc+ |
| 26905 | 12009_2013 | Neisseria meningitidis | B | ST-269 complex | No value | Ireland | SV-1 | Opc+ |
| 26906 | 12010_2013 | Neisseria meningitidis | B | ST-35 complex | No value | Ireland | SV-12 | Opc+ |
| 26907 | 12011_2013 | Neisseria meningitidis | B | ST-213 complex | No value | Ireland | SV-12 | Opc- |
| 26908 | 12012_2013 | Neisseria meningitidis | X | ST-1157 complex | No value | Ireland | SV-1 | Opc+ |
| 26909 | 12013_2013 | Neisseria meningitidis | B | ST-41/44 complex | No value | Ireland | SV-2 | Opc+ |
| 26910 | 12014_2013 | Neisseria meningitidis | E | ST-1157 complex | No value | Ireland | SV-1 | Opc+ |
| 26911 | 12015_2013 | Neisseria meningitidis | B | ST-269 complex | No value | Ireland | SV-8 | Opc+ |
| 26912 | 12016_2013 | Neisseria meningitidis | B | ST-269 complex | No value | Ireland | SV-1 | Opc+ |
| 26913 | 12017_2013 | Neisseria meningitidis | B | ST-213 complex | No value | Ireland | SV-11 | Opc- |
| 26914 | 12018_2013 | Neisseria meningitidis | W | ST-11 complex | No value | Ireland | SV-2 | Opc- |
| 26915 | 12019_2013 | Neisseria meningitidis | B | ST-41/44 complex | No value | Ireland | SV-2 | Opc+ |
| 26916 | 12055_2009 | Neisseria meningitidis | B | ST-35 complex | No value | Ireland | SV-12 | Opc+ |
| 26917 | 12049_2008 | Neisseria meningitidis | B | ST-269 complex | No value | Ireland | SV-1 | Opc+ |
| 26918 | 10007v1 | Neisseria meningitidis | NG | ST-11 complex | No value | UK | SV-2 | Opc- |
| 26919 | 10117v2 | Neisseria meningitidis | NG | ST-11 complex | No value | UK | SV-2 | Opc- |
| 26920 | 10117v3 | Neisseria meningitidis | NG | ST-11 complex | No value | UK | SV-2 | Opc- |
| 26921 | 10117v4 | Neisseria meningitidis | NG | ST-11 complex | No value | UK | SV-2 | Opc- |
| 26922 | 10215v3 | Neisseria meningitidis | NG | ST-22 complex | No value | UK | SV-12 | Opc+ |
| 26923 | 10215v4 | Neisseria meningitidis | NG | ST-22 complex | No value | UK | SV-12 | Opc+ |
| 26924 | 10215v5 | Neisseria meningitidis | NG | No value | No value | UK | SV-12 | Opc+ |
| 26925 | 10507v4 | Neisseria meningitidis | NG | ST-11 complex | No value | UK | SV-2 | Opc- |
| 26926 | 10507v5 | Neisseria meningitidis | NG | ST-11 complex | No value | UK | SV-2 | Opc- |

| | | | | | | | | |
|---|---|---|---|---|---|---|---|---|
| 26927 | 10551v1 | Neisseria meningitidis | NG | No value | No value | UK | SV-12 | Opc+ |
| 26928 | 10551v2 | Neisseria meningitidis | NG | No value | No value | UK | SV-12 | Opc+ |
| 26929 | 10551v3 | Neisseria meningitidis | NG | No value | No value | UK | SV-12 | Opc+ |
| 26930 | 10569v5 | Neisseria meningitidis | NG | ST-11 complex | No value | UK | SV-2 | Opc- |
| 26931 | 40118v3 | Neisseria meningitidis | NG | ST-11 complex | No value | UK | SV-2 | Opc- |
| 26932 | 40118v4 | Neisseria meningitidis | NG | ST-11 complex | No value | UK | SV-2 | Opc- |
| 26933 | 40161v1 | Neisseria meningitidis | NG | ST-11 complex | No value | UK | SV-2 | Opc- |
| 26934 | 40161v2 | Neisseria meningitidis | NG | ST-11 complex | No value | UK | SV-2 | Opc- |
| 26935 | 40161v3 | Neisseria meningitidis | NG | ST-11 complex | No value | UK | SV-2 | Opc- |
| 26936 | 40161v4 | Neisseria meningitidis | NG | ST-11 complex | No value | UK | SV-2 | Opc- |
| 26937 | 40161v5 | Neisseria meningitidis | NG | ST-11 complex | No value | UK | SV-2 | Opc- |
| 26938 | 60153v1 | Neisseria meningitidis | NG | No value | No value | UK | SV-12 | Opc+ |
| 26939 | 60153v2b | Neisseria meningitidis | NG | No value | No value | UK | SV-12 | Opc+ |
| 26940 | 60153v3 | Neisseria meningitidis | NG | No value | No value | UK | SV-12 | Opc+ |
| 26941 | 60153v4 | Neisseria meningitidis | NG | No value | No value | UK | SV-12 | Opc+ |
| 26942 | 60153v5b | Neisseria meningitidis | NG | No value | No value | UK | SV-12 | Opc+ |
| 26943 | 60153v6 | Neisseria meningitidis | NG | No value | No value | UK | SV-12 | Opc+ |
| 26944 | 70045v4b | Neisseria meningitidis | NG | ST-11 complex | No value | UK | SV-2 | Opc- |
| 26945 | 70306v6 | Neisseria meningitidis | NG | ST-11 complex | No value | UK | SV-2 | Opc- |
| 26946 | 70322v3 | Neisseria meningitidis | NG | ST-11 complex | No value | UK | SV-2 | Opc- |
| 26947 | 70322v4 | Neisseria meningitidis | NG | ST-11 complex | No value | UK | SV-2 | Opc- |
| 26948 | 70322v5 | Neisseria meningitidis | NG | ST-11 complex | No value | UK | SV-2 | Opc- |
| 26949 | 80046v4b | Neisseria meningitidis | NG | ST-11 complex | No value | UK | SV-2 | Opc- |
| 26950 | 80046v5 | Neisseria meningitidis | NG | ST-11 complex | No value | UK | SV-2 | Opc- |
| 26951 | 80046v6 | Neisseria meningitidis | NG | ST-11 complex | No value | UK | SV-2 | Opc- |
| 26952 | 80164v1 | Neisseria meningitidis | NG | ST-11 complex | No value | UK | SV-2 | Opc- |
| 26953 | 80164v2 | Neisseria meningitidis | NG | ST-11 complex | No value | UK | SV-2 | Opc- |
| 26954 | 80241v5 | Neisseria meningitidis | NG | ST-22 complex | No value | UK | SV-12 | Opc+ |
| 26955 | 80305v4 | Neisseria meningitidis | NG | ST-22 complex | No value | UK | SV-12 | Opc+ |
| 26956 | 80305v5 | Neisseria meningitidis | NG | ST-22 complex | No value | UK | SV-12 | Opc+ |
| 26957 | 90021v3 | Neisseria meningitidis | NG | ST-11 complex | No value | UK | SV-2 | Opc- |
| 26958 | 90021v4 | Neisseria meningitidis | NG | ST-11 complex | No value | UK | SV-2 | Opc- |
| 26959 | 90022v2 | Neisseria meningitidis | NG | ST-11 complex | No value | UK | SV-2 | Opc- |
| 26960 | 90022v3 | Neisseria meningitidis | NG | ST-11 complex | No value | UK | SV-2 | Opc- |
| 26961 | 90022v4 | Neisseria meningitidis | NG | ST-11 complex | No value | UK | SV-2 | Opc- |
| 26962 | 90028v1 | Neisseria meningitidis | NG | ST-11 complex | No value | UK | SV-2 | Opc- |
| 26963 | 90034v4 | Neisseria meningitidis | NG | ST-11 complex | No value | UK | SV-2 | Opc- |
| 26964 | 90034v6 | Neisseria meningitidis | NG | ST-11 complex | No value | UK | SV-2 | Opc- |
| 26965 | 90058v2 | Neisseria meningitidis | NG | ST-11 complex | No value | UK | SV-2 | Opc- |
| 26966 | 90058v3 | Neisseria meningitidis | NG | ST-11 complex | No value | UK | SV-2 | Opc- |
| 26967 | 90058v4 | Neisseria meningitidis | NG | ST-11 complex | No value | UK | SV-2 | Opc- |
| 26968 | 90066v1 | Neisseria meningitidis | NG | ST-11 complex | No value | UK | SV-2 | Opc- |
| 26969 | 90075v3 | Neisseria meningitidis | NG | ST-11 complex | No value | UK | SV-2 | Opc- |
| 26970 | 90092v1 | Neisseria meningitidis | NG | ST-11 complex | No value | UK | SV-2 | Opc- |
| 26971 | 90092v2 | Neisseria meningitidis | NG | ST-11 complex | No value | UK | SV-2 | Opc- |
| 26972 | 90092v5 | Neisseria meningitidis | NG | ST-11 complex | No value | UK | SV-2 | Opc- |
| 26973 | 90092v6 | Neisseria meningitidis | NG | ST-11 complex | No value | UK | SV-2 | Opc- |
| 26974 | 90102v3 | Neisseria meningitidis | NG | ST-11 complex | No value | UK | SV-2 | Opc- |
| 26975 | 90102v4 | Neisseria meningitidis | NG | ST-11 complex | No value | UK | SV-2 | Opc- |
| 26976 | 90104v5 | Neisseria meningitidis | NG | ST-22 complex | No value | UK | SV-12 | Opc+ |
| 26978 | 90111v4 | Neisseria meningitidis | NG | ST-11 complex | No value | UK | SV-2 | Opc- |
| 26979 | 90111v5 | Neisseria meningitidis | NG | ST-11 complex | No value | UK | SV-2 | Opc- |
| 26980 | 90119v2 | Neisseria meningitidis | NG | ST-11 complex | No value | UK | SV-2 | Opc- |
| 26981 | 90119v3 | Neisseria meningitidis | NG | ST-11 complex | No value | UK | SV-2 | Opc- |
| 26982 | 90119v4 | Neisseria meningitidis | NG | ST-11 complex | No value | UK | SV-2 | Opc- |
| 26983 | 90119v5 | Neisseria meningitidis | NG | ST-11 complex | No value | UK | SV-2 | Opc- |
| 26984 | 90124v4 | Neisseria meningitidis | NG | ST-11 complex | No value | UK | SV-2 | Opc- |
| 26985 | 90130v1 | Neisseria meningitidis | NG | ST-22 complex | No value | UK | SV-12 | Opc+ |
| 26986 | 90130v2 | Neisseria meningitidis | NG | ST-22 complex | No value | UK | SV-12 | Opc+ |
| 26987 | 90130v5b | Neisseria meningitidis | NG | ST-22 complex | No value | UK | SV-12 | Opc+ |
| 26988 | 90130v6 | Neisseria meningitidis | NG | ST-22 complex | No value | UK | SV-12 | Opc+ |
| 26989 | 90136v3 | Neisseria meningitidis | NG | ST-11 complex | No value | UK | SV-2 | Opc- |
| 26990 | 90136v4 | Neisseria meningitidis | NG | ST-11 complex | No value | UK | SV-2 | Opc- |
| 26991 | 90130v3 | Neisseria meningitidis | NG | ST-11 complex | No value | UK | SV-2 | Opc- |
| 26992 | 90142v1 | Neisseria meningitidis | NG | ST-11 complex | No value | UK | SV-2 | Opc- |
| 26993 | 90149v1 | Neisseria meningitidis | NG | ST-11 complex | No value | UK | SV-2 | Opc- |
| 26994 | 90174v1 | Neisseria meningitidis | NG | ST-11 complex | No value | UK | SV-2 | Opc- |
| 26995 | 90182v1 | Neisseria meningitidis | NG | ST-11 complex | No value | UK | SV-2 | Opc- |
| 26996 | 70306v1 | Neisseria meningitidis | NG | ST-167 complex | No value | UK | SV-5 | Opc+ |
| 26997 | 90075v2 | Neisseria meningitidis | NG | ST-11 complex | No value | UK | SV-2 | Opc- |
| 26998 | 90142v2 | Neisseria meningitidis | NG | ST-1117 complex | No value | UK | SV-10 | Opc- |
| 26999 | 90142v3 | Neisseria meningitidis | NG | ST-1117 complex | No value | UK | SV-10 | Opc- |
| 27087 | LNP19995 | Neisseria meningitidis | W | ST-11 complex | Epidemic | Burkina Faso | SV-2 | Opc- |
| 27088 | LNP21362 | Neisseria meningitidis | B | ST-32 complex | Epidemic | France | SV-1 | Opc+ |

| | | | | | | | | |
|---|---|---|---|---|---|---|---|---|
| 27089 | LNP24198 | Neisseria meningitidis | C | ST-11 complex | Sporadic case | France | SV-2 | Opc- |
| 27778 | M12 240336 | Neisseria meningitidis | B | ST-41/44 complex | No value | UK | SV-6 | Opc+ |
| 27779 | M12 240338 | Neisseria meningitidis | B | ST-35 complex | No value | UK | SV-12 | Opc+ |
| 27780 | M12 240340 | Neisseria meningitidis | B | ST-213 complex | No value | UK | SV-12 | Opc- |
| 27781 | M12 240341 | Neisseria meningitidis | B | ST-269 complex | No value | UK | SV-8 | Opc+ |
| 27782 | M12 240347 | Neisseria meningitidis | B | ST-461 complex | No value | UK | SV-1 | Opc- |
| 27783 | M12 240349 | Neisseria meningitidis | B | ST-41/44 complex | No value | UK | SV-6 | Opc+ |
| 27784 | M12 240644 | Neisseria meningitidis | B | No value | No value | UK | SV-5 | Opc+ |
| 27785 | M12 240647 | Neisseria meningitidis | B | ST-32 complex | No value | UK | SV-1 | Opc+ |
| 27786 | M12 240650 | Neisseria meningitidis | B | ST-41/44 complex | No value | UK | SV-2 | Opc+ |
| 27787 | M12 240651 | Neisseria meningitidis | B | ST-41/44 complex | No value | UK | SV-2 | Opc+ |
| 27788 | M12 240652 | Neisseria meningitidis | B | ST-41/44 complex | No value | UK | SV-2 | Opc+ |
| 27789 | M12 240653 | Neisseria meningitidis | B | ST-269 complex | No value | UK | SV-8 | Opc+ |
| 27790 | M12 240654 | Neisseria meningitidis | B | ST-41/44 complex | No value | UK | SV-2 | Opc+ |
| 27791 | M12 240656 | Neisseria meningitidis | B | ST-41/44 complex | No value | UK | SV-6 | Opc+ |
| 27792 | M12 240658 | Neisseria meningitidis | B | ST-41/44 complex | No value | UK | SV-6 | Opc+ |
| 27793 | M12 240659 | Neisseria meningitidis | B | ST-22 complex | No value | UK | SV-12 | Opc+ |
| 27794 | M12 240664 | Neisseria meningitidis | B | ST-41/44 complex | No value | UK | SV-2 | Opc+ |
| 27795 | M12 240667 | Neisseria meningitidis | B | ST-41/44 complex | No value | UK | SV-2 | Opc+ |
| 27796 | M12 240668 | Neisseria meningitidis | B | ST-41/44 complex | No value | UK | SV-2 | Opc+ |
| 27797 | M12 240669 | Neisseria meningitidis | B | No value | No value | UK | SV-8 | Opc+ |
| 27798 | M12 240672 | Neisseria meningitidis | B | ST-41/44 complex | No value | UK | SV-10 | Opc+ |
| 27799 | M12 240674 | Neisseria meningitidis | B | ST-269 complex | No value | UK | SV-8 | Opc+ |
| 27800 | M12 240675 | Neisseria meningitidis | B | ST-269 complex | No value | UK | SV-1 | Opc+ |
| 27801 | M12 240678 | Neisseria meningitidis | B | ST-269 complex | No value | UK | SV-1 | Opc+ |
| 27803 | M12 240682 | Neisseria meningitidis | B | ST-11 complex | No value | UK | SV-2 | Opc- |
| 27804 | M12 240683 | Neisseria meningitidis | B | ST-269 complex | No value | UK | SV-8 | Opc+ |
| 27805 | M12 240684 | Neisseria meningitidis | B | No value | No value | UK | SV-8 | Opc+ |
| 27806 | M12 240685 | Neisseria meningitidis | B | No value | No value | UK | SV-5 | Opc- |
| 27807 | M12 240688 | Neisseria meningitidis | B | ST-269 complex | No value | UK | SV-8 | Opc+ |
| 27808 | M12 240691 | Neisseria meningitidis | B | ST-269 complex | No value | UK | SV-8 | Opc+ |
| 27809 | M12 240693 | Neisseria meningitidis | B | ST-32 complex | No value | UK | SV-1 | Opc+ |
| 27810 | M12 240694 | Neisseria meningitidis | B | ST-269 complex | No value | UK | SV-1 | Opc+ |
| 27811 | M12 240697 | Neisseria meningitidis | B | ST-41/44 complex | No value | UK | SV-2 | Opc+ |
| 27812 | M12 240699 | Neisseria meningitidis | B | ST-41/44 complex | No value | UK | SV-6 | Opc+ |
| 27813 | M12 240703 | Neisseria meningitidis | B | ST-213 complex | No value | UK | SV-12 | Opc- |
| 27814 | M12 240704 | Neisseria meningitidis | B | ST-461 complex | No value | UK | SV-1 | Opc- |
| 27815 | M12 240705 | Neisseria meningitidis | B | ST-213 complex | No value | UK | SV-5 | Opc- |
| 27816 | M12 240706 | Neisseria meningitidis | B | ST-41/44 complex | No value | UK | SV-2 | Opc+ |
| 27817 | M12 240707 | Neisseria meningitidis | B | ST-269 complex | No value | UK | SV-1 | Opc+ |
| 27818 | M12 240712 | Neisseria meningitidis | B | ST-41/44 complex | No value | UK | SV-2 | Opc+ |
| 27819 | M12 240714 | Neisseria meningitidis | B | ST-32 complex | No value | UK | SV-1 | Opc+ |
| 27820 | M12 240718 | Neisseria meningitidis | B | ST-269 complex | No value | UK | SV-1 | Opc+ |
| 27821 | M12 240719 | Neisseria meningitidis | B | ST-269 complex | No value | UK | SV-8 | Opc+ |
| 27822 | M12 240720 | Neisseria meningitidis | B | ST-41/44 complex | No value | UK | SV-2 | Opc+ |
| 27823 | M12 240724 | Neisseria meningitidis | B | ST-213 complex | No value | UK | SV-12 | Opc- |
| 27824 | M12 240725 | Neisseria meningitidis | B | ST-35 complex | No value | UK | SV-12 | Opc+ |
| 27825 | M12 240729 | Neisseria meningitidis | B | ST-269 complex | No value | UK | SV-8 | Opc+ |
| 27826 | M12 240730 | Neisseria meningitidis | B | ST-32 complex | No value | UK | SV-1 | Opc+ |
| 27827 | M12 240732 | Neisseria meningitidis | B | ST-32 complex | No value | UK | SV-1 | Opc+ |
| 27828 | M12 240733 | Neisseria meningitidis | B | ST-213 complex | No value | UK | SV-12 | Opc- |
| 27829 | M12 240735 | Neisseria meningitidis | B | ST-32 complex | No value | UK | SV-1 | Opc+ |
| 27830 | M12 240736 | Neisseria meningitidis | B | ST-162 complex | No value | UK | SV-1 | Opc+ |
| 27831 | M12 240737 | Neisseria meningitidis | B | ST-269 complex | No value | UK | SV-1 | Opc+ |
| 27832 | M12 240739 | Neisseria meningitidis | B | ST-162 complex | No value | UK | SV-1 | Opc+ |
| 27833 | M12 240741 | Neisseria meningitidis | B | ST-41/44 complex | No value | UK | SV-2 | Opc+ |
| 27834 | M12 240745 | Neisseria meningitidis | B | ST-269 complex | No value | UK | SV-1 | Opc+ |
| 27835 | M12 240749 | Neisseria meningitidis | B | No value | No value | UK | SV-8 | Opc+ |
| 27836 | M12 240750 | Neisseria meningitidis | B | ST-41/44 complex | No value | UK | SV-2 | Opc+ |
| 27837 | M12 240752 | Neisseria meningitidis | B | ST-41/44 complex | No value | UK | SV-2 | Opc+ |
| 27838 | M12 240758 | Neisseria meningitidis | B | ST-32 complex | No value | UK | SV-1 | Opc+ |
| 27839 | M12 240763 | Neisseria meningitidis | B | ST-461 complex | No value | UK | SV-1 | Opc- |
| 27840 | M12 240765 | Neisseria meningitidis | B | ST-269 complex | No value | UK | SV-11 | Opc+ |
| 27841 | M12 240769 | Neisseria meningitidis | B | No value | No value | UK | SV-1 | Opc+ |
| 27842 | M12 240773 | Neisseria meningitidis | B | ST-269 complex | No value | UK | SV-1 | Opc+ |
| 27843 | M12 240778 | Neisseria meningitidis | B | ST-461 complex | No value | UK | SV-1 | Opc- |
| 27844 | M12 240781 | Neisseria meningitidis | B | ST-269 complex | No value | UK | SV-8 | Opc+ |
| 27845 | M12 240782 | Neisseria meningitidis | B | ST-213 complex | No value | UK | SV-12 | Opc- |
| 27846 | M12 240783 | Neisseria meningitidis | B | ST-41/44 complex | No value | UK | SV-2 | Opc+ |
| 27847 | M12 240785 | Neisseria meningitidis | B | ST-41/44 complex | No value | UK | SV-2 | Opc+ |
| 27848 | M12 240786 | Neisseria meningitidis | B | No value | No value | UK | SV-6 | Opc- |
| 27849 | M12 240787 | Neisseria meningitidis | B | ST-461 complex | No value | UK | SV-1 | Opc- |
| 27850 | M12 240790 | Neisseria meningitidis | B | ST-32 complex | No value | UK | SV-1 | Opc+ |
| 27851 | M12 240793 | Neisseria meningitidis | B | ST-269 complex | No value | UK | SV-8 | Opc+ |

| | | | | | | | | |
|---|---|---|---|---|---|---|---|---|
| 27852 | M12 240794 | Neisseria meningitidis | B | ST-35 complex | No value | UK | SV-16 | Opc+ |
| 27853 | M12 240803 | Neisseria meningitidis | B | ST-32 complex | No value | UK | SV-1 | Opc+ |
| 27854 | M12 240805 | Neisseria meningitidis | B | No value | No value | UK | SV-1 | Opc+ |
| 27855 | M12 240806 | Neisseria meningitidis | B | ST-18 complex | No value | UK | SV-7 | Opc- |
| 27856 | M12 240807 | Neisseria meningitidis | B | No value | No value | UK | SV-6 | Opc+ |
| 27857 | M12 240808 | Neisseria meningitidis | B | ST-282 complex | No value | UK | SV-9 | Opc+ |
| 27858 | M12 240809 | Neisseria meningitidis | B | No value | No value | UK | SV-8 | Opc- |
| 27859 | M12 240810 | Neisseria meningitidis | B | ST-269 complex | No value | UK | SV-1 | Opc+ |
| 27860 | M12 240812 | Neisseria meningitidis | B | ST-269 complex | No value | UK | SV-1 | Opc+ |
| 27861 | M12 240814 | Neisseria meningitidis | B | ST-269 complex | No value | UK | SV-8 | Opc+ |
| 27862 | M12 240817 | Neisseria meningitidis | B | ST-32 complex | No value | UK | SV-1 | Opc+ |
| 27863 | M12 240818 | Neisseria meningitidis | B | ST-32 complex | No value | UK | SV-1 | Opc+ |
| 27864 | M12 240819 | Neisseria meningitidis | B | ST-461 complex | No value | UK | SV-1 | Opc- |
| 27865 | M12 240825 | Neisseria meningitidis | B | ST-41/44 complex | No value | UK | SV-2 | Opc+ |
| 27866 | M12 240827 | Neisseria meningitidis | B | ST-269 complex | No value | UK | SV-8 | Opc+ |
| 27867 | M12 240831 | Neisseria meningitidis | B | ST-269 complex | No value | UK | SV-1 | Opc+ |
| 27868 | M12 240834 | Neisseria meningitidis | B | ST-269 complex | No value | UK | SV-1 | Opc+ |
| 27869 | M12 240842 | Neisseria meningitidis | B | ST-269 complex | No value | UK | SV-8 | Opc+ |
| 27870 | M12 240843 | Neisseria meningitidis | B | No value | No value | UK | SV-1 | Opc- |
| 27871 | M12 240844 | Neisseria meningitidis | B | ST-461 complex | No value | UK | SV-5 | Opc- |
| 27872 | M12 240846 | Neisseria meningitidis | B | ST-41/44 complex | No value | UK | SV-2 | Opc+ |
| 27873 | M12 240847 | Neisseria meningitidis | B | ST-41/44 complex | No value | UK | SV-2 | Opc+ |
| 27874 | M12 240848 | Neisseria meningitidis | B | ST-213 complex | No value | UK | SV-12 | Opc- |
| 27875 | M12 240849 | Neisseria meningitidis | B | ST-41/44 complex | No value | UK | SV-2 | Opc+ |
| 27876 | M12 240850 | Neisseria meningitidis | B | ST-41/44 complex | No value | UK | SV-2 | Opc+ |
| 27877 | M12 240851 | Neisseria meningitidis | B | ST-269 complex | No value | UK | SV-8 | Opc+ |
| 27878 | M12 240852 | Neisseria meningitidis | B | ST-269 complex | No value | UK | SV-1 | Opc+ |
| 27879 | M12 240853 | Neisseria meningitidis | B | ST-269 complex | No value | UK | SV-1 | Opc+ |
| 27880 | M12 240858 | Neisseria meningitidis | B | ST-269 complex | No value | UK | SV-1 | Opc+ |
| 27881 | M12 240861 | Neisseria meningitidis | B | No value | No value | UK | SV-5 | Opc- |
| 27882 | M12 240865 | Neisseria meningitidis | B | ST-18 complex | No value | UK | SV-5 | Opc- |
| 27883 | M12 240866 | Neisseria meningitidis | B | ST-213 complex | No value | UK | SV-12 | Opc- |
| 27884 | M12 240867 | Neisseria meningitidis | B | ST-269 complex | No value | UK | SV-1 | Opc+ |
| 27885 | M12 240868 | Neisseria meningitidis | B | ST-41/44 complex | No value | UK | SV-2 | Opc+ |
| 27886 | M12 240872 | Neisseria meningitidis | B | No value | No value | UK | SV-10 | Opc+ |
| 27887 | M12 240873 | Neisseria meningitidis | B | ST-461 complex | No value | UK | SV-1 | Opc- |
| 27888 | M12 240874 | Neisseria meningitidis | B | ST-32 complex | No value | UK | SV-1 | Opc+ |
| 27889 | M12 240876 | Neisseria meningitidis | B | ST-41/44 complex | No value | UK | SV-10 | Opc+ |
| 27890 | M12 240877 | Neisseria meningitidis | B | ST-269 complex | No value | UK | SV-8 | Opc+ |
| 27891 | M12 240878 | Neisseria meningitidis | B | ST-162 complex | No value | UK | SV-1 | Opc+ |
| 27892 | M12 240879 | Neisseria meningitidis | B | ST-269 complex | No value | UK | SV-8 | Opc+ |
| 27893 | M12 240880 | Neisseria meningitidis | B | ST-269 complex | No value | UK | SV-1 | Opc+ |
| 27894 | M12 240884 | Neisseria meningitidis | B | ST-32 complex | No value | UK | SV-1 | Opc+ |
| 27895 | M12 240887 | Neisseria meningitidis | B | ST-41/44 complex | No value | UK | SV-2 | Opc+ |
| 27896 | M12 240893 | Neisseria meningitidis | B | ST-269 complex | No value | UK | SV-1 | Opc+ |
| 27897 | M12 240896 | Neisseria meningitidis | B | ST-32 complex | No value | UK | SV-1 | Opc+ |
| 27898 | M12 240899 | Neisseria meningitidis | B | ST-213 complex | No value | UK | SV-12 | Opc- |
| 27899 | M12 240900 | Neisseria meningitidis | B | ST-269 complex | No value | UK | SV-1 | Opc+ |
| 27900 | M12 240901 | Neisseria meningitidis | B | ST-41/44 complex | No value | UK | SV-14 | Opc+ |
| 27901 | M12 240902 | Neisseria meningitidis | B | ST-41/44 complex | No value | UK | SV-2 | Opc+ |
| 27902 | M13 240000 | Neisseria meningitidis | B | No value | No value | UK | SV-8 | Opc+ |
| 27903 | M13 240001 | Neisseria meningitidis | B | ST-41/44 complex | No value | UK | SV-2 | Opc+ |
| 27904 | M13 240003 | Neisseria meningitidis | B | ST-269 complex | No value | UK | SV-1 | Opc+ |
| 27905 | M13 240004 | Neisseria meningitidis | B | ST-269 complex | No value | UK | SV-1 | Opc+ |
| 27906 | M13 240005 | Neisseria meningitidis | B | ST-269 complex | No value | UK | SV-1 | Opc+ |
| 27907 | M13 240006 | Neisseria meningitidis | B | ST-461 complex | No value | UK | SV-1 | Opc- |
| 27908 | M13 240007 | Neisseria meningitidis | B | ST-41/44 complex | No value | UK | SV-2 | Opc+ |
| 27909 | M13 240009 | Neisseria meningitidis | B | ST-41/44 complex | No value | UK | SV-6 | Opc+ |
| 27910 | M13 240012 | Neisseria meningitidis | B | ST-41/44 complex | No value | UK | SV-2 | Opc+ |
| 27911 | M13 240014 | Neisseria meningitidis | B | ST-269 complex | No value | UK | SV-8 | Opc+ |
| 27912 | M13 240017 | Neisseria meningitidis | B | ST-32 complex | No value | UK | SV-1 | Opc+ |
| 27913 | M13 240019 | Neisseria meningitidis | B | ST-41/44 complex | No value | UK | SV-6 | Opc+ |
| 27914 | M13 240021 | Neisseria meningitidis | B | ST-461 complex | No value | UK | SV-1 | Opc- |
| 27915 | M13 240023 | Neisseria meningitidis | B | ST-35 complex | No value | UK | SV-12 | Opc+ |
| 27916 | M13 240029 | Neisseria meningitidis | B | ST-213 complex | No value | UK | SV-12 | Opc- |
| 27917 | M13 240030 | Neisseria meningitidis | B | ST-269 complex | No value | UK | SV-8 | Opc+ |
| 27918 | M13 240031 | Neisseria meningitidis | B | ST-41/44 complex | No value | UK | SV-10 | Opc+ |
| 27919 | M13 240032 | Neisseria meningitidis | B | ST-35 complex | No value | UK | SV-12 | Opc+ |
| 27920 | M13 240033 | Neisseria meningitidis | B | ST-269 complex | No value | UK | SV-8 | Opc+ |
| 27921 | M13 240036 | Neisseria meningitidis | B | No value | No value | UK | SV-8 | Opc+ |
| 27922 | M13 240039 | Neisseria meningitidis | B | ST-269 complex | No value | UK | SV-1 | Opc+ |
| 27923 | M13 240040 | Neisseria meningitidis | B | ST-269 complex | No value | UK | SV-1 | Opc+ |
| 27924 | M13 240041 | Neisseria meningitidis | B | ST-41/44 complex | No value | UK | SV-6 | Opc+ |
| 27925 | M13 240042 | Neisseria meningitidis | B | No value | No value | UK | SV-10 | Opc- |

| | | | | | | | | |
|---|---|---|---|---|---|---|---|---|
| 27926 | M13 240043 | Neisseria meningitidis | B | ST-269 complex | No value | UK | SV-1 | Opc+ |
| 27927 | M13 240045 | Neisseria meningitidis | B | ST-461 complex | No value | UK | SV-1 | Opc- |
| 27928 | M13 240046 | Neisseria meningitidis | B | ST-269 complex | No value | UK | SV-8 | Opc+ |
| 27929 | M13 240047 | Neisseria meningitidis | B | ST-41/44 complex | No value | UK | SV-6 | Opc+ |
| 27930 | M13 240048 | Neisseria meningitidis | B | ST-269 complex | No value | UK | SV-8 | Opc+ |
| 27931 | M13 240051 | Neisseria meningitidis | B | ST-269 complex | No value | UK | SV-1 | Opc+ |
| 27932 | M13 240054 | Neisseria meningitidis | B | ST-269 complex | No value | UK | SV-1 | Opc+ |
| 27933 | M13 240057 | Neisseria meningitidis | B | ST-461 complex | No value | UK | SV-5 | Opc- |
| 27934 | M13 240063 | Neisseria meningitidis | B | ST-461 complex | No value | UK | SV-1 | Opc- |
| 27935 | M13 240067 | Neisseria meningitidis | B | ST-269 complex | No value | UK | SV-8 | Opc+ |
| 27936 | M13 240069 | Neisseria meningitidis | B | ST-269 complex | No value | UK | SV-8 | Opc+ |
| 27937 | M13 240072 | Neisseria meningitidis | B | ST-269 complex | No value | UK | SV-1 | Opc+ |
| 27938 | M13 240074 | Neisseria meningitidis | B | ST-461 complex | No value | UK | SV-1 | Opc- |
| 27939 | M13 240075 | Neisseria meningitidis | B | No value | No value | UK | SV-8 | Opc+ |
| 27940 | M13 240076 | Neisseria meningitidis | B | No value | No value | UK | SV-19 | Opc+ |
| 27941 | M13 240078 | Neisseria meningitidis | B | ST-461 complex | No value | UK | SV-1 | Opc- |
| 27942 | M13 240081 | Neisseria meningitidis | B | ST-213 complex | No value | UK | SV-12 | Opc- |
| 27943 | M13 240084 | Neisseria meningitidis | B | ST-41/44 complex | No value | UK | SV-6 | Opc+ |
| 27944 | M13 240085 | Neisseria meningitidis | B | ST-41/44 complex | No value | UK | SV-6 | Opc+ |
| 27945 | M13 240086 | Neisseria meningitidis | B | No value | No value | UK | SV-2 | Opc+ |
| 27946 | M13 240088 | Neisseria meningitidis | B | ST-41/44 complex | No value | UK | SV-2 | Opc+ |
| 27947 | M13 240090 | Neisseria meningitidis | B | ST-41/44 complex | No value | UK | SV-6 | Opc+ |
| 27948 | M13 240091 | Neisseria meningitidis | B | ST-162 complex | No value | UK | SV-1 | Opc+ |
| 27949 | M13 240095 | Neisseria meningitidis | B | ST-269 complex | No value | UK | SV-1 | Opc+ |
| 27950 | M13 240097 | Neisseria meningitidis | B | ST-41/44 complex | No value | UK | SV-6 | Opc+ |
| 27951 | M13 240098 | Neisseria meningitidis | B | ST-41/44 complex | No value | UK | SV-10 | Opc+ |
| 27952 | M13 240105 | Neisseria meningitidis | B | No value | No value | UK | SV-1 | Opc+ |
| 27953 | M13 240106 | Neisseria meningitidis | B | ST-32 complex | No value | UK | SV-1 | Opc+ |
| 27954 | M13 240111 | Neisseria meningitidis | B | ST-41/44 complex | No value | UK | SV-2 | Opc+ |
| 27955 | M13 240112 | Neisseria meningitidis | B | ST-269 complex | No value | UK | SV-1 | Opc+ |
| 27956 | M13 240115 | Neisseria meningitidis | B | ST-41/44 complex | No value | UK | SV-2 | Opc+ |
| 27957 | M13 240118 | Neisseria meningitidis | B | ST-32 complex | No value | UK | SV-1 | Opc+ |
| 27958 | M13 240120 | Neisseria meningitidis | B | ST-41/44 complex | No value | UK | SV-2 | Opc+ |
| 27959 | M13 240121 | Neisseria meningitidis | B | ST-269 complex | No value | UK | SV-8 | Opc+ |
| 27960 | M13 240123 | Neisseria meningitidis | B | ST-41/44 complex | No value | UK | SV-2 | Opc+ |
| 27961 | M13 240124 | Neisseria meningitidis | B | ST-269 complex | No value | UK | SV-8 | Opc+ |
| 27962 | M13 240125 | Neisseria meningitidis | B | ST-282 complex | No value | UK | SV-9 | Opc+ |
| 27963 | M13 240127 | Neisseria meningitidis | B | ST-213 complex | No value | UK | SV-12 | Opc- |
| 27964 | M13 240130 | Neisseria meningitidis | B | ST-32 complex | No value | UK | SV-1 | Opc+ |
| 27965 | M13 240132 | Neisseria meningitidis | B | ST-213 complex | No value | UK | SV-12 | Opc- |
| 27966 | M13 240135 | Neisseria meningitidis | B | ST-41/44 complex | No value | UK | SV-2 | Opc+ |
| 27967 | M13 240142 | Neisseria meningitidis | B | ST-41/44 complex | No value | UK | SV-5 | Opc- |
| 27968 | M13 240143 | Neisseria meningitidis | B | ST-162 complex | No value | UK | SV-1 | Opc+ |
| 27969 | M13 240145 | Neisseria meningitidis | B | ST-282 complex | No value | UK | SV-9 | Opc+ |
| 27970 | M13 240147 | Neisseria meningitidis | B | ST-269 complex | No value | UK | SV-1 | Opc+ |
| 27971 | M13 240148 | Neisseria meningitidis | B | No value | No value | UK | SV-11 | Opc- |
| 27972 | M13 240150 | Neisseria meningitidis | B | No value | No value | UK | SV-8 | Opc+ |
| 27973 | M13 240153 | Neisseria meningitidis | B | ST-60 complex | No value | UK | SV-11 | Opc+ |
| 27974 | M13 240157 | Neisseria meningitidis | B | ST-41/44 complex | No value | UK | SV-2 | Opc+ |
| 27975 | M13 240160 | Neisseria meningitidis | B | ST-269 complex | No value | UK | SV-1 | Opc+ |
| 27976 | M13 240161 | Neisseria meningitidis | B | ST-35 complex | No value | UK | SV-12 | Opc+ |
| 27977 | M13 240164 | Neisseria meningitidis | B | ST-269 complex | No value | UK | SV-1 | Opc+ |
| 27978 | M13 240166 | Neisseria meningitidis | B | ST-41/44 complex | No value | UK | SV-2 | Opc+ |
| 27979 | M13 240167 | Neisseria meningitidis | B | ST-269 complex | No value | UK | SV-1 | Opc+ |
| 27980 | M13 240170 | Neisseria meningitidis | B | ST-213 complex | No value | UK | SV-12 | Opc- |
| 27981 | M13 240171 | Neisseria meningitidis | B | ST-41/44 complex | No value | UK | SV-5 | Opc+ |
| 27982 | M13 240172 | Neisseria meningitidis | B | ST-269 complex | No value | UK | SV-8 | Opc+ |
| 27983 | M13 240173 | Neisseria meningitidis | B | ST-41/44 complex | No value | UK | SV-6 | Opc+ |
| 27984 | M13 240174 | Neisseria meningitidis | B | ST-41/44 complex | No value | UK | SV-6 | Opc+ |
| 27985 | M13 240175 | Neisseria meningitidis | B | ST-41/44 complex | No value | UK | SV-6 | Opc+ |
| 27986 | M13 240181 | Neisseria meningitidis | B | ST-41/44 complex | No value | UK | SV-2 | Opc+ |
| 27987 | M13 240183 | Neisseria meningitidis | B | No value | No value | UK | SV-1 | Opc+ |
| 27988 | M13 240187 | Neisseria meningitidis | B | ST-213 complex | No value | UK | SV-12 | Opc- |
| 27989 | M13 240191 | Neisseria meningitidis | B | ST-269 complex | No value | UK | SV-8 | Opc+ |
| 27990 | M13 240192 | Neisseria meningitidis | B | ST-269 complex | No value | UK | SV-8 | Opc+ |
| 27991 | M13 240197 | Neisseria meningitidis | B | ST-269 complex | No value | UK | SV-8 | Opc+ |
| 27992 | M13 240199 | Neisseria meningitidis | B | ST-11 complex | No value | UK | SV-2 | Opc- |
| 27993 | M13 240202 | Neisseria meningitidis | B | No value | No value | UK | SV-8 | Opc+ |
| 27994 | M13 240204 | Neisseria meningitidis | B | ST-41/44 complex | No value | UK | SV-2 | Opc+ |
| 27995 | M13 240205 | Neisseria meningitidis | B | ST-41/44 complex | No value | UK | SV-2 | Opc+ |
| 27996 | M13 240206 | Neisseria meningitidis | B | ST-41/44 complex | No value | UK | SV-10 | Opc+ |
| 27997 | M13 240207 | Neisseria meningitidis | B | ST-269 complex | No value | UK | SV-8 | Opc+ |
| 27998 | M13 240210 | Neisseria meningitidis | B | ST-41/44 complex | No value | UK | SV-2 | Opc+ |
| 27999 | M13 240211 | Neisseria meningitidis | B | No value | No value | UK | SV-8 | Opc+ |

| | | | | | | | | |
|---|---|---|---|---|---|---|---|---|
| 28000 | M13 240212 | Neisseria meningitidis | B | ST-41/44 complex | No value | UK | SV-5 | Opc- |
| 28001 | M13 240213 | Neisseria meningitidis | B | ST-269 complex | No value | UK | SV-8 | Opc+ |
| 28002 | M13 240216 | Neisseria meningitidis | B | ST-32 complex | No value | UK | SV-1 | Opc+ |
| 28003 | M13 240217 | Neisseria meningitidis | B | ST-41/44 complex | No value | UK | SV-2 | Opc+ |
| 28004 | M13 240224 | Neisseria meningitidis | B | No value | No value | UK | SV-2 | Opc+ |
| 28005 | M13 240227 | Neisseria meningitidis | B | ST-269 complex | No value | UK | SV-8 | Opc+ |
| 28006 | M13 240228 | Neisseria meningitidis | B | ST-41/44 complex | No value | UK | SV-2 | Opc+ |
| 28007 | M13 240229 | Neisseria meningitidis | B | ST-32 complex | No value | UK | SV-1 | Opc+ |
| 28008 | M13 240230 | Neisseria meningitidis | B | ST-32 complex | No value | UK | SV-1 | Opc+ |
| 28009 | M13 240231 | Neisseria meningitidis | B | ST-41/44 complex | No value | UK | SV-2 | Opc+ |
| 28010 | M13 240232 | Neisseria meningitidis | B | ST-213 complex | No value | UK | SV-12 | Opc- |
| 28011 | M13 240233 | Neisseria meningitidis | B | ST-269 complex | No value | UK | SV-8 | Opc+ |
| 28012 | M13 240234 | Neisseria meningitidis | B | ST-41/44 complex | No value | UK | SV-2 | Opc+ |
| 28013 | M13 240235 | Neisseria meningitidis | B | ST-41/44 complex | No value | UK | SV-2 | Opc+ |
| 28014 | M13 240237 | Neisseria meningitidis | B | ST-41/44 complex | No value | UK | SV-2 | Opc+ |
| 28015 | M13 240239 | Neisseria meningitidis | B | ST-41/44 complex | No value | UK | SV-2 | Opc+ |
| 28016 | M13 240245 | Neisseria meningitidis | B | ST-269 complex | No value | UK | SV-1 | Opc+ |
| 28017 | M13 240248 | Neisseria meningitidis | B | ST-461 complex | No value | UK | SV-1 | Opc- |
| 28018 | M13 240249 | Neisseria meningitidis | B | ST-213 complex | No value | UK | SV-12 | Opc- |
| 28019 | M13 240250 | Neisseria meningitidis | B | ST-167 complex | No value | UK | SV-5 | Opc+ |
| 28020 | M13 240255 | Neisseria meningitidis | B | ST-269 complex | No value | UK | SV-8 | Opc+ |
| 28021 | M13 240256 | Neisseria meningitidis | B | ST-269 complex | No value | UK | SV-8 | Opc+ |
| 28022 | M13 240257 | Neisseria meningitidis | B | ST-269 complex | No value | UK | SV-8 | Opc+ |
| 28023 | M13 240258 | Neisseria meningitidis | B | ST-213 complex | No value | UK | SV-11 | Opc- |
| 28024 | M13 240261 | Neisseria meningitidis | B | ST-41/44 complex | No value | UK | SV-2 | Opc+ |
| 28025 | M13 240262 | Neisseria meningitidis | B | ST-32 complex | No value | UK | SV-1 | Opc+ |
| 28026 | M13 240263 | Neisseria meningitidis | B | ST-41/44 complex | No value | UK | SV-2 | Opc+ |
| 28027 | M13 240264 | Neisseria meningitidis | B | ST-1157 complex | No value | UK | SV-1 | Opc+ |
| 28028 | M13 240271 | Neisseria meningitidis | B | ST-41/44 complex | No value | UK | SV-6 | Opc+ |
| 28029 | M13 240272 | Neisseria meningitidis | B | ST-162 complex | No value | UK | SV-1 | Opc+ |
| 28030 | M13 240273 | Neisseria meningitidis | B | ST-41/44 complex | No value | UK | SV-2 | Opc+ |
| 28031 | M13 240274 | Neisseria meningitidis | B | ST-269 complex | No value | UK | SV-1 | Opc+ |
| 28032 | M13 240275 | Neisseria meningitidis | B | ST-41/44 complex | No value | UK | SV-2 | Opc+ |
| 28033 | M13 240277 | Neisseria meningitidis | B | ST-41/44 complex | No value | UK | SV-6 | Opc+ |
| 28034 | M13 240281 | Neisseria meningitidis | B | ST-213 complex | No value | UK | SV-12 | Opc- |
| 28035 | M13 240289 | Neisseria meningitidis | B | ST-269 complex | No value | UK | SV-1 | Opc+ |
| 28036 | M13 240292 | Neisseria meningitidis | B | No value | No value | UK | SV-6 | Opc+ |
| 28037 | M13 240294 | Neisseria meningitidis | B | ST-269 complex | No value | UK | SV-8 | Opc+ |
| 28038 | M13 240384 | Neisseria meningitidis | B | ST-32 complex | No value | UK | SV-1 | Opc+ |
| 28039 | M13 240385 | Neisseria meningitidis | B | ST-41/44 complex | No value | UK | SV-2 | Opc+ |
| 28040 | M13 240386 | Neisseria meningitidis | B | No value | No value | UK | SV-1 | Opc+ |
| 28041 | M13 240397 | Neisseria meningitidis | B | No value | No value | UK | SV-16 | Opc+ |
| 28042 | M13 240400 | Neisseria meningitidis | B | ST-269 complex | No value | UK | SV-1 | Opc+ |
| 28043 | M13 240406 | Neisseria meningitidis | B | ST-461 complex | No value | UK | SV-1 | Opc- |
| 28044 | M13 240409 | Neisseria meningitidis | B | ST-41/44 complex | No value | UK | SV-2 | Opc+ |
| 28045 | M13 240411 | Neisseria meningitidis | B | ST-269 complex | No value | UK | SV-1 | Opc+ |
| 28046 | M13 240413 | Neisseria meningitidis | B | ST-41/44 complex | No value | UK | SV-2 | Opc+ |
| 28047 | M13 240414 | Neisseria meningitidis | B | ST-162 complex | No value | UK | SV-1 | Opc+ |
| 28048 | M13 240417 | Neisseria meningitidis | B | ST-60 complex | No value | UK | SV-11 | Opc+ |
| 28049 | M13 240418 | Neisseria meningitidis | B | ST-41/44 complex | No value | UK | SV-2 | Opc+ |
| 28050 | M13 240421 | Neisseria meningitidis | B | ST-269 complex | No value | UK | SV-1 | Opc+ |
| 28051 | M13 240422 | Neisseria meningitidis | B | ST-269 complex | No value | UK | SV-8 | Opc+ |
| 28052 | M13 240424 | Neisseria meningitidis | B | No value | No value | UK | SV-7 | Opc- |
| 28053 | M13 240426 | Neisseria meningitidis | B | No value | No value | UK | SV-8 | Opc+ |
| 28054 | M13 240427 | Neisseria meningitidis | B | ST-213 complex | No value | UK | SV-12 | Opc- |
| 28055 | M13 240428 | Neisseria meningitidis | B | ST-269 complex | No value | UK | SV-8 | Opc+ |
| 28056 | M13 240429 | Neisseria meningitidis | B | ST-269 complex | No value | UK | SV-1 | Opc+ |
| 28057 | M13 240434 | Neisseria meningitidis | B | ST-254 complex | No value | UK | SV-1 | Opc+ |
| 28058 | M13 240437 | Neisseria meningitidis | B | ST-213 complex | No value | UK | SV-12 | Opc- |
| 28059 | M13 240438 | Neisseria meningitidis | B | ST-41/44 complex | No value | UK | SV-15 | Opc+ |
| 28060 | M13 240448 | Neisseria meningitidis | B | ST-103 complex | No value | UK | SV-5 | Opc+ |
| 28061 | M13 240449 | Neisseria meningitidis | B | ST-41/44 complex | No value | UK | SV-2 | Opc+ |
| 28062 | M13 240450 | Neisseria meningitidis | B | ST-269 complex | No value | UK | SV-8 | Opc+ |
| 28063 | M13 240451 | Neisseria meningitidis | B | ST-213 complex | No value | UK | SV-12 | Opc- |
| 28064 | M13 240452 | Neisseria meningitidis | B | ST-41/44 complex | No value | UK | SV-2 | Opc+ |
| 28065 | M13 240453 | Neisseria meningitidis | B | ST-269 complex | No value | UK | SV-1 | Opc+ |
| 28066 | M13 240455 | Neisseria meningitidis | B | ST-269 complex | No value | UK | SV-8 | Opc+ |
| 28067 | M13 240458 | Neisseria meningitidis | B | ST-103 complex | No value | UK | SV-5 | Opc+ |
| 28068 | M13 240461 | Neisseria meningitidis | B | No value | No value | UK | SV-1 | Opc+ |
| 28069 | M13 240463 | Neisseria meningitidis | B | ST-269 complex | No value | UK | SV-8 | Opc+ |
| 28070 | M13 240466 | Neisseria meningitidis | B | ST-269 complex | No value | UK | SV-5 | Opc+ |
| 28071 | M13 240468 | Neisseria meningitidis | B | ST-162 complex | No value | UK | SV-1 | Opc+ |
| 28072 | M13 240472 | Neisseria meningitidis | B | ST-41/44 complex | No value | UK | SV-2 | Opc+ |
| 28073 | M13 240475 | Neisseria meningitidis | B | ST-41/44 complex | No value | UK | SV-2 | Opc+ |

| | | | | | | | | |
|---|---|---|---|---|---|---|---|---|
| 28074 | M13 240476 | Neisseria meningitidis | B | ST-269 complex | No value | UK | SV-8 | Opc+ |
| 28075 | M13 240478 | Neisseria meningitidis | B | ST-269 complex | No value | UK | SV-8 | Opc+ |
| 28076 | M13 240479 | Neisseria meningitidis | B | ST-41/44 complex | No value | UK | SV-2 | Opc+ |
| 28077 | M13 240481 | Neisseria meningitidis | B | ST-213 complex | No value | UK | SV-10 | Opc- |
| 28078 | M13 240483 | Neisseria meningitidis | B | ST-269 complex | No value | UK | SV-8 | Opc+ |
| 28079 | M12 240345 | Neisseria meningitidis | C | ST-103 complex | No value | UK | SV-5 | Opc+ |
| 28080 | M12 240639 | Neisseria meningitidis | C | ST-11 complex | No value | UK | SV-2 | Opc- |
| 28081 | M12 240655 | Neisseria meningitidis | C | ST-269 complex | No value | UK | SV-1 | Opc+ |
| 28082 | M12 240679 | Neisseria meningitidis | C | ST-11 complex | No value | UK | SV-2 | Opc- |
| 28083 | M12 240698 | Neisseria meningitidis | C | ST-11 complex | No value | UK | SV-2 | Opc- |
| 28084 | M12 240716 | Neisseria meningitidis | C | ST-11 complex | No value | UK | SV-2 | Opc- |
| 28085 | M12 240776 | Neisseria meningitidis | C | ST-11 complex | No value | UK | SV-2 | Opc- |
| 28086 | M12 240854 | Neisseria meningitidis | C | ST-174 complex | No value | UK | SV-1 | Opc+ |
| 28087 | M12 240855 | Neisseria meningitidis | C | ST-174 complex | No value | UK | SV-1 | Opc+ |
| 28088 | M13 240002 | Neisseria meningitidis | C | No value | No value | UK | SV-6 | Opc+ |
| 28089 | M13 240008 | Neisseria meningitidis | C | ST-11 complex | No value | UK | SV-2 | Opc- |
| 28090 | M13 240022 | Neisseria meningitidis | C | ST-11 complex | No value | UK | SV-2 | Opc- |
| 28091 | M13 240026 | Neisseria meningitidis | C | ST-269 complex | No value | UK | SV-1 | Opc+ |
| 28092 | M13 240068 | Neisseria meningitidis | C | ST-11 complex | No value | UK | SV-2 | Opc- |
| 28093 | M13 240155 | Neisseria meningitidis | C | ST-11 complex | No value | UK | SV-2 | Opc- |
| 28094 | M13 240162 | Neisseria meningitidis | C | ST-11 complex | No value | UK | SV-2 | Opc- |
| 28095 | M13 240189 | Neisseria meningitidis | C | ST-11 complex | No value | UK | SV-2 | Opc- |
| 28096 | M13 240201 | Neisseria meningitidis | C | No value | No value | UK | SV-5 | Opc+ |
| 28097 | M13 240254 | Neisseria meningitidis | C | ST-11 complex | No value | UK | SV-2 | Opc- |
| 28098 | M13 240280 | Neisseria meningitidis | C | ST-11 complex | No value | UK | SV-2 | Opc- |
| 28099 | M13 240282 | Neisseria meningitidis | C | ST-11 complex | No value | UK | SV-2 | Opc- |
| 28100 | M13 240295 | Neisseria meningitidis | C | ST-11 complex | No value | UK | SV-2 | Opc- |
| 28101 | M13 240398 | Neisseria meningitidis | C | ST-103 complex | No value | UK | SV-5 | Opc+ |
| 28102 | M13 240401 | Neisseria meningitidis | C | ST-11 complex | No value | UK | SV-2 | Opc- |
| 28103 | M13 240402 | Neisseria meningitidis | C | ST-11 complex | No value | UK | SV-2 | Opc- |
| 28104 | M13 240405 | Neisseria meningitidis | C | ST-11 complex | No value | UK | SV-2 | Opc- |
| 28105 | M13 240440 | Neisseria meningitidis | C | ST-11 complex | No value | UK | SV-2 | Opc- |
| 28106 | M13 240460 | Neisseria meningitidis | C | ST-11 complex | No value | UK | SV-2 | Opc- |
| 28107 | M12 240641 | Neisseria meningitidis | NG | ST-1157 complex | No value | UK | SV-1 | Opc+ |
| 28108 | M12 240756 | Neisseria meningitidis | NG | ST-269 complex | No value | UK | SV-8 | Opc+ |
| 28109 | M13 240134 | Neisseria meningitidis | NG | ST-198 complex | No value | UK | SV-4 | Opc+ |
| 28110 | M13 240198 | Neisseria meningitidis | NG | ST-41/44 complex | No value | UK | SV-2 | Opc+ |
| 28111 | M13 240240 | Neisseria meningitidis | NG | ST-213 complex | No value | UK | SV-12 | Opc- |
| 28112 | M13 240399 | Neisseria meningitidis | NG | ST-41/44 complex | No value | UK | SV-2 | Opc+ |
| 28113 | M13 240410 | Neisseria meningitidis | NG | ST-1157 complex | No value | UK | SV-1 | Opc+ |
| 28114 | M12 240337 | Neisseria meningitidis | W | ST-11 complex | No value | UK | SV-2 | Opc- |
| 28115 | M12 240640 | Neisseria meningitidis | W | ST-11 complex | No value | UK | SV-2 | Opc- |
| 28116 | M12 240657 | Neisseria meningitidis | W | ST-11 complex | No value | UK | SV-2 | Opc- |
| 28117 | M12 240663 | Neisseria meningitidis | W | ST-11 complex | No value | UK | SV-2 | Opc- |
| 28118 | M12 240671 | Neisseria meningitidis | W | ST-22 complex | No value | UK | SV-12 | Opc+ |
| 28119 | M12 240702 | Neisseria meningitidis | W | ST-11 complex | No value | UK | SV-2 | Opc- |
| 28120 | M12 240734 | Neisseria meningitidis | W | ST-22 complex | No value | UK | SV-12 | Opc+ |
| 28121 | M12 240751 | Neisseria meningitidis | W | ST-11 complex | No value | UK | SV-2 | Opc- |
| 28122 | M12 240754 | Neisseria meningitidis | W | ST-11 complex | No value | UK | SV-2 | Opc- |
| 28123 | M12 240762 | Neisseria meningitidis | W | ST-22 complex | No value | UK | SV-12 | Opc+ |
| 28124 | M12 240772 | Neisseria meningitidis | W | ST-22 complex | No value | UK | SV-12 | Opc+ |
| 28125 | M12 240774 | Neisseria meningitidis | W | ST-11 complex | No value | UK | SV-2 | Opc- |
| 28126 | M12 240789 | Neisseria meningitidis | W | ST-22 complex | No value | UK | SV-12 | Opc+ |
| 28127 | M12 240823 | Neisseria meningitidis | W | ST-22 complex | No value | UK | SV-12 | Opc+ |
| 28128 | M12 240826 | Neisseria meningitidis | W | ST-11 complex | No value | UK | SV-2 | Opc- |
| 28129 | M12 240862 | Neisseria meningitidis | W | ST-22 complex | No value | UK | SV-12 | Opc+ |
| 28130 | M12 240881 | Neisseria meningitidis | W | ST-23 complex | No value | UK | SV-11 | Opc+ |
| 28131 | M12 240895 | Neisseria meningitidis | W | ST-11 complex | No value | UK | SV-2 | Opc- |
| 28132 | M12 240898 | Neisseria meningitidis | W | ST-11 complex | No value | UK | SV-2 | Opc- |
| 28133 | M13 240013 | Neisseria meningitidis | W | ST-22 complex | No value | UK | SV-12 | Opc+ |
| 28134 | M13 240015 | Neisseria meningitidis | W | ST-11 complex | No value | UK | SV-2 | Opc- |
| 28135 | M13 240025 | Neisseria meningitidis | W | ST-11 complex | No value | UK | SV-2 | Opc- |
| 28136 | M13 240028 | Neisseria meningitidis | W | ST-11 complex | No value | UK | SV-2 | Opc- |
| 28137 | M13 240056 | Neisseria meningitidis | W | ST-11 complex | No value | UK | SV-2 | Opc- |
| 28138 | M13 240066 | Neisseria meningitidis | W | ST-11 complex | No value | UK | SV-2 | Opc- |
| 28139 | M13 240077 | Neisseria meningitidis | W | ST-11 complex | No value | UK | SV-2 | Opc- |
| 28140 | M13 240089 | Neisseria meningitidis | W | ST-22 complex | No value | UK | SV-12 | Opc+ |
| 28141 | M13 240109 | Neisseria meningitidis | W | ST-11 complex | No value | UK | SV-2 | Opc- |
| 28142 | M13 240114 | Neisseria meningitidis | W | ST-11 complex | No value | UK | SV-2 | Opc- |
| 28143 | M13 240158 | Neisseria meningitidis | W | ST-11 complex | No value | UK | SV-2 | Opc- |
| 28144 | M13 240168 | Neisseria meningitidis | W | ST-11 complex | No value | UK | SV-2 | Opc- |
| 28145 | M13 240176 | Neisseria meningitidis | W | ST-22 complex | No value | UK | SV-12 | Opc+ |
| 28146 | M13 240185 | Neisseria meningitidis | W | ST-11 complex | No value | UK | SV-2 | Opc- |
| 28147 | M13 240223 | Neisseria meningitidis | W | ST-11 complex | No value | UK | SV-2 | Opc- |

| | | | | | | | | |
|---|---|---|---|---|---|---|---|---|
| 28148 | M13 240225 | Neisseria meningitidis | W | ST-11 complex | No value | UK | SV-2 | Opc- |
| 28149 | M13 240238 | Neisseria meningitidis | W | ST-11 complex | No value | UK | SV-2 | Opc- |
| 28150 | M13 240246 | Neisseria meningitidis | W | ST-11 complex | No value | UK | SV-2 | Opc- |
| 28151 | M13 240247 | Neisseria meningitidis | W | ST-11 complex | No value | UK | SV-2 | Opc- |
| 28152 | M13 240251 | Neisseria meningitidis | W | ST-11 complex | No value | UK | SV-2 | Opc- |
| 28153 | M13 240269 | Neisseria meningitidis | W | ST-11 complex | No value | UK | SV-2 | Opc- |
| 28154 | M13 240283 | Neisseria meningitidis | W | ST-11 complex | No value | UK | SV-2 | Opc- |
| 28155 | M13 240431 | Neisseria meningitidis | W | ST-22 complex | No value | UK | SV-12 | Opc+ |
| 28156 | M13 240436 | Neisseria meningitidis | W | ST-11 complex | No value | UK | SV-2 | Opc- |
| 28157 | M13 240446 | Neisseria meningitidis | W | ST-11 complex | No value | UK | SV-2 | Opc- |
| 28158 | M13 240457 | Neisseria meningitidis | W | ST-11 complex | No value | UK | SV-2 | Opc- |
| 28159 | M13 240464 | Neisseria meningitidis | W | ST-11 complex | No value | UK | SV-2 | Opc- |
| 28160 | M13 240465 | Neisseria meningitidis | W | ST-22 complex | No value | UK | SV-12 | Opc+ |
| 28161 | M13 240467 | Neisseria meningitidis | W | ST-11 complex | No value | UK | SV-2 | Opc- |
| 28162 | M13 240469 | Neisseria meningitidis | W | ST-11 complex | No value | UK | SV-2 | Opc- |
| 28163 | M13 240473 | Neisseria meningitidis | W | ST-11 complex | No value | UK | SV-2 | Opc- |
| 28164 | M13 240482 | Neisseria meningitidis | W | ST-11 complex | No value | UK | SV-2 | Opc- |
| 28165 | M12 240342 | Neisseria meningitidis | Y | ST-23 complex | No value | UK | SV-11 | Opc+ |
| 28166 | M12 240343 | Neisseria meningitidis | Y | ST-23 complex | No value | UK | SV-11 | Opc+ |
| 28167 | M12 240643 | Neisseria meningitidis | Y | ST-23 complex | No value | UK | SV-11 | Opc+ |
| 28168 | M12 240646 | Neisseria meningitidis | Y | ST-23 complex | No value | UK | SV-11 | Opc+ |
| 28169 | M12 240661 | Neisseria meningitidis | Y | ST-23 complex | No value | UK | SV-11 | Opc+ |
| 28170 | M12 240701 | Neisseria meningitidis | Y | ST-23 complex | No value | UK | SV-11 | Opc+ |
| 28171 | M12 240713 | Neisseria meningitidis | Y | ST-23 complex | No value | UK | SV-11 | Opc+ |
| 28172 | M12 240715 | Neisseria meningitidis | Y | ST-23 complex | No value | UK | SV-11 | Opc+ |
| 28173 | M12 240717 | Neisseria meningitidis | Y | ST-174 complex | No value | UK | SV-1 | Opc+ |
| 28174 | M12 240728 | Neisseria meningitidis | Y | ST-23 complex | No value | UK | SV-11 | Opc+ |
| 28175 | M12 240746 | Neisseria meningitidis | Y | ST-23 complex | No value | UK | SV-11 | Opc+ |
| 28176 | M12 240748 | Neisseria meningitidis | Y | ST-103 complex | No value | UK | SV-5 | Opc+ |
| 28177 | M12 240760 | Neisseria meningitidis | Y | ST-23 complex | No value | UK | SV-11 | Opc+ |
| 28178 | M12 240767 | Neisseria meningitidis | Y | ST-23 complex | No value | UK | SV-11 | Opc+ |
| 28179 | M12 240779 | Neisseria meningitidis | Y | ST-23 complex | No value | UK | SV-11 | Opc+ |
| 28180 | M12 240784 | Neisseria meningitidis | Y | ST-174 complex | No value | UK | SV-1 | Opc+ |
| 28181 | M12 240788 | Neisseria meningitidis | Y | ST-23 complex | No value | UK | SV-11 | Opc+ |
| 28182 | M12 240791 | Neisseria meningitidis | Y | ST-23 complex | No value | UK | SV-11 | Opc+ |
| 28183 | M12 240796 | Neisseria meningitidis | Y | ST-167 complex | No value | UK | SV-5 | Opc+ |
| 28184 | M12 240797 | Neisseria meningitidis | Y | ST-167 complex | No value | UK | SV-5 | Opc+ |
| 28185 | M12 240799 | Neisseria meningitidis | Y | ST-23 complex | No value | UK | SV-11 | Opc+ |
| 28186 | M12 240801 | Neisseria meningitidis | Y | ST-23 complex | No value | UK | SV-11 | Opc+ |
| 28187 | M12 240802 | Neisseria meningitidis | Y | ST-22 complex | No value | UK | SV-12 | Opc+ |
| 28188 | M12 240816 | Neisseria meningitidis | Y | ST-23 complex | No value | UK | SV-11 | Opc+ |
| 28189 | M12 240821 | Neisseria meningitidis | Y | ST-167 complex | No value | UK | SV-5 | Opc+ |
| 28191 | M12 240840 | Neisseria meningitidis | Y | ST-23 complex | No value | UK | SV-15 | Opc+ |
| 28192 | M12 240857 | Neisseria meningitidis | Y | ST-23 complex | No value | UK | SV-11 | Opc+ |
| 28193 | M12 240885 | Neisseria meningitidis | Y | ST-23 complex | No value | UK | SV-11 | Opc+ |
| 28194 | M12 240886 | Neisseria meningitidis | Y | ST-23 complex | No value | UK | SV-11 | Opc+ |
| 28195 | M12 240888 | Neisseria meningitidis | Y | ST-23 complex | No value | UK | SV-11 | Opc+ |
| 28196 | M12 240894 | Neisseria meningitidis | Y | ST-23 complex | No value | UK | SV-11 | Opc+ |
| 28197 | M12 240897 | Neisseria meningitidis | Y | ST-23 complex | No value | UK | SV-11 | Opc+ |
| 28198 | M13 240011 | Neisseria meningitidis | Y | ST-23 complex | No value | UK | SV-11 | Opc+ |
| 28199 | M13 240020 | Neisseria meningitidis | Y | ST-22 complex | No value | UK | SV-12 | Opc+ |
| 28200 | M13 240035 | Neisseria meningitidis | Y | ST-23 complex | No value | UK | SV-11 | Opc+ |
| 28201 | M13 240044 | Neisseria meningitidis | Y | ST-23 complex | No value | UK | SV-11 | Opc+ |
| 28202 | M13 240053 | Neisseria meningitidis | Y | ST-167 complex | No value | UK | SV-5 | Opc+ |
| 28203 | M13 240055 | Neisseria meningitidis | Y | ST-22 complex | No value | UK | SV-12 | Opc+ |
| 28204 | M13 240059 | Neisseria meningitidis | Y | ST-167 complex | No value | UK | SV-5 | Opc+ |
| 28205 | M13 240064 | Neisseria meningitidis | Y | ST-23 complex | No value | UK | SV-11 | Opc+ |
| 28206 | M13 240073 | Neisseria meningitidis | Y | ST-23 complex | No value | UK | SV-11 | Opc+ |
| 28207 | M13 240087 | Neisseria meningitidis | Y | ST-23 complex | No value | UK | SV-11 | Opc+ |
| 28208 | M13 240099 | Neisseria meningitidis | Y | ST-23 complex | No value | UK | SV-11 | Opc+ |
| 28209 | M13 240102 | Neisseria meningitidis | Y | ST-23 complex | No value | UK | SV-11 | Opc+ |
| 28210 | M13 240116 | Neisseria meningitidis | Y | ST-23 complex | No value | UK | SV-11 | Opc+ |
| 28211 | M13 240126 | Neisseria meningitidis | Y | ST-167 complex | No value | UK | SV-5 | Opc+ |
| 28212 | M13 240131 | Neisseria meningitidis | Y | ST-23 complex | No value | UK | SV-11 | Opc+ |
| 28213 | M13 240136 | Neisseria meningitidis | Y | ST-167 complex | No value | UK | SV-5 | Opc+ |
| 28214 | M13 240144 | Neisseria meningitidis | Y | ST-23 complex | No value | UK | SV-11 | Opc+ |
| 28215 | M13 240146 | Neisseria meningitidis | Y | ST-23 complex | No value | UK | SV-11 | Opc+ |
| 28216 | M13 240154 | Neisseria meningitidis | Y | ST-23 complex | No value | UK | SV-11 | Opc+ |
| 28217 | M13 240165 | Neisseria meningitidis | Y | ST-23 complex | No value | UK | SV-11 | Opc+ |
| 28218 | M13 240184 | Neisseria meningitidis | Y | ST-23 complex | No value | UK | SV-11 | Opc+ |
| 28219 | M13 240188 | Neisseria meningitidis | Y | ST-23 complex | No value | UK | SV-11 | Opc+ |
| 28220 | M13 240194 | Neisseria meningitidis | Y | ST-23 complex | No value | UK | SV-11 | Opc+ |
| 28221 | M13 240200 | Neisseria meningitidis | Y | ST-23 complex | No value | UK | SV-11 | Opc+ |
| 28222 | M13 240203 | Neisseria meningitidis | Y | ST-23 complex | No value | UK | SV-11 | Opc+ |

| | | | | | | | | |
|---|---|---|---|---|---|---|---|---|
| 28223 | M13 240218 | Neisseria meningitidis | Y | ST-23 complex | No value | UK | SV-11 | Opc+ |
| 28224 | M13 240220 | Neisseria meningitidis | Y | ST-23 complex | No value | UK | SV-11 | Opc+ |
| 28225 | M13 240226 | Neisseria meningitidis | Y | No value | No value | UK | SV-5 | Opc+ |
| 28226 | M13 240242 | Neisseria meningitidis | Y | ST-23 complex | No value | UK | SV-11 | Opc+ |
| 28227 | M13 240244 | Neisseria meningitidis | Y | ST-23 complex | No value | UK | SV-11 | Opc+ |
| 28228 | M13 240265 | Neisseria meningitidis | Y | ST-23 complex | No value | UK | SV-11 | Opc+ |
| 28229 | M13 240267 | Neisseria meningitidis | Y | ST-23 complex | No value | UK | SV-11 | Opc+ |
| 28230 | M13 240268 | Neisseria meningitidis | Y | ST-23 complex | No value | UK | SV-11 | Opc+ |
| 28231 | M13 240381 | Neisseria meningitidis | Y | ST-23 complex | No value | UK | SV-11 | Opc+ |
| 28232 | M13 240383 | Neisseria meningitidis | Y | ST-865 complex | No value | UK | SV-7 | Opc+ |
| 28233 | M13 240396 | Neisseria meningitidis | Y | ST-23 complex | No value | UK | SV-11 | Opc+ |
| 28234 | M13 240407 | Neisseria meningitidis | Y | ST-23 complex | No value | UK | SV-11 | Opc+ |
| 28235 | M13 240439 | Neisseria meningitidis | Y | ST-23 complex | No value | UK | SV-11 | Opc+ |
| 28247 | N241.1 | Neisseria meningitidis | Y | ST-174 complex | Carrier | UK | SV-1 | Opc+ |
| 28248 | N51.1 | Neisseria meningitidis | Y | ST-174 complex | Carrier | UK | SV-1 | Opc+ |
| 28249 | N52.1 | Neisseria meningitidis | Y | ST-174 complex | Carrier | UK | SV-1 | Opc+ |
| 28250 | N54.1 | Neisseria meningitidis | Y | ST-174 complex | Carrier | UK | SV-1 | Opc+ |
| 28251 | N58.1 | Neisseria meningitidis | Y | ST-174 complex | Carrier | UK | SV-1 | Opc+ |
| 28252 | N59.1 | Neisseria meningitidis | Y | ST-174 complex | Carrier | UK | SV-1 | Opc+ |
| 28253 | N88.1 | Neisseria meningitidis | Y | ST-174 complex | Carrier | UK | SV-1 | Opc+ |
| 28254 | N138.1 | Neisseria meningitidis | Y | ST-174 complex | Carrier | UK | SV-1 | Opc+ |
| 28255 | N64.1 | Neisseria meningitidis | Y | ST-167 complex | Carrier | UK | SV-5 | Opc+ |
| 28256 | N117.1 | Neisseria meningitidis | Y | ST-167 complex | Carrier | UK | SV-5 | Opc+ |
| 28257 | N124.1 | Neisseria meningitidis | Y | ST-167 complex | Carrier | UK | SV-5 | Opc+ |
| 28258 | N128.1 | Neisseria meningitidis | NG | ST-167 complex | Carrier | UK | SV-5 | Opc+ |
| 28259 | N258.1 | Neisseria meningitidis | Y | ST-23 complex | Carrier | UK | SV-11 | Opc+ |
| 28260 | N264.1 | Neisseria meningitidis | Y | ST-23 complex | Carrier | UK | SV-11 | Opc+ |
| 28261 | N259.1 | Neisseria meningitidis | Y | ST-23 complex | Carrier | UK | SV-11 | Opc+ |
| 28262 | N222.1 | Neisseria meningitidis | Y | ST-23 complex | Carrier | UK | SV-11 | Opc+ |
| 28263 | N114.1 | Neisseria meningitidis | E | ST-60 complex | Carrier | UK | SV-11 | Opc+ |
| 28264 | N134.1 | Neisseria meningitidis | NG | ST-60 complex | Carrier | UK | SV-11 | Opc+ |
| 28265 | N185.1 | Neisseria meningitidis | NG | ST-60 complex | Carrier | UK | SV-11 | Opc+ |
| 28266 | N185.2 | Neisseria meningitidis | NG | ST-60 complex | Carrier | UK | SV-11 | Opc+ |
| 28267 | N262.1 | Neisseria meningitidis | NG | ST-1157 complex | Carrier | UK | SV-1 | Opc+ |
| 28268 | N73.1 | Neisseria meningitidis | NG | ST-1157 complex | Carrier | UK | SV-1 | Opc+ |
| 28269 | N188.1 | Neisseria meningitidis | Y | ST-23 complex | Carrier | UK | SV-11 | Opc+ |
| 28270 | N199.1 | Neisseria meningitidis | NG | ST-269 complex | Carrier | UK | SV-1 | Opc+ |
| 28271 | N176.1 | Neisseria meningitidis | NG | ST-32 complex | Carrier | UK | SV-1 | Opc+ |
| 28272 | N86.1 | Neisseria meningitidis | NG | No value | Carrier | UK | SV-2 | Opc+ |
| 28273 | N349.1 | Neisseria meningitidis | Y | ST-174 complex | Carrier | UK | SV-1 | Opc+ |
| 28274 | N424.1 | Neisseria meningitidis | Y | ST-174 complex | Carrier | UK | SV-1 | Opc+ |
| 28275 | N342.1 | Neisseria meningitidis | Y | ST-174 complex | Carrier | UK | SV-1 | Opc+ |
| 28276 | N343.1 | Neisseria meningitidis | Y | ST-174 complex | Carrier | UK | SV-1 | Opc+ |
| 28277 | N429.1 | Neisseria meningitidis | Y | ST-174 complex | Carrier | UK | SV-1 | Opc+ |
| 28278 | N438.1 | Neisseria meningitidis | Y | ST-174 complex | Carrier | UK | SV-1 | Opc+ |
| 28279 | N449.1 | Neisseria meningitidis | Y | ST-174 complex | Carrier | UK | SV-1 | Opc+ |
| 28280 | N331.1 | Neisseria meningitidis | Y | ST-174 complex | Carrier | UK | SV-1 | Opc+ |
| 28281 | N348.1 | Neisseria meningitidis | Y | ST-167 complex | Carrier | UK | SV-5 | Opc+ |
| 28282 | N417.1 | Neisseria meningitidis | NG | ST-167 complex | Carrier | UK | SV-5 | Opc+ |
| 28283 | N336.1 | Neisseria meningitidis | Y | ST-167 complex | Carrier | UK | SV-5 | Opc+ |
| 28284 | N420.1 | Neisseria meningitidis | NG | ST-167 complex | Carrier | UK | SV-5 | Opc+ |
| 28285 | N431.1 | Neisseria meningitidis | Y | ST-23 complex | Carrier | UK | SV-11 | Opc+ |
| 28286 | N359.1 | Neisseria meningitidis | NG | ST-23 complex | Carrier | UK | SV-11 | Opc+ |
| 28287 | N445.1 | Neisseria meningitidis | Y | ST-23 complex | Carrier | UK | SV-11 | Opc+ |
| 28288 | N459.1 | Neisseria meningitidis | Y | ST-23 complex | Carrier | UK | SV-11 | Opc+ |
| 28289 | N330.1 | Neisseria meningitidis | NG | ST-60 complex | Carrier | UK | SV-11 | Opc+ |
| 28290 | N333.1 | Neisseria meningitidis | NG | ST-60 complex | Carrier | UK | SV-11 | Opc+ |
| 28291 | N456.1 | Neisseria meningitidis | NG | ST-60 complex | Carrier | UK | SV-11 | Opc+ |
| 28292 | N456.2 | Neisseria meningitidis | NG | ST-60 complex | Carrier | UK | SV-11 | Opc+ |
| 28293 | N446.1 | Neisseria meningitidis | NG | ST-1157 complex | Carrier | UK | SV-11 | Opc+ |
| 28294 | N450.1 | Neisseria meningitidis | NG | ST-1157 complex | Carrier | UK | SV-1 | Opc+ |
| 28295 | N462.1 | Neisseria meningitidis | Y | ST-23 complex | Carrier | UK | SV-1 | Opc+ |
| 28296 | N378.1 | Neisseria meningitidis | NG | ST-269 complex | Carrier | UK | SV-11 | Opc+ |
| 28297 | N408.1 | Neisseria meningitidis | NG | ST-32 complex | Carrier | UK | SV-1 | Opc+ |
| 28298 | N447.1 | Neisseria meningitidis | NG | No value | Carrier | UK | SV-1 | Opc+ |
| 28800 | LNP27366 | Neisseria meningitidis | C | ST-11 complex | Sporadic case | Poland | SV-2 | Opc+ |
| 29276 | 11258 | Neisseria meningitidis | A | ST-1 complex | Endemic | South Africa | SV-2 | Opc- |
| 29277 | 8942 | Neisseria meningitidis | A | ST-1 complex | Endemic | South Africa | SV-5 | Opc+ |
| 29278 | 13779 | Neisseria meningitidis | A | ST-1 complex | Endemic | South Africa | SV-5 | Opc+ |
| 29280 | 13361 | Neisseria meningitidis | A | ST-1 complex | Endemic | South Africa | SV-5 | Opc+ |
| 29281 | 38277 | Neisseria meningitidis | C | ST-865 complex | Endemic | South Africa | SV-5 | Opc+ |
| 29282 | 13611 | Neisseria meningitidis | A | ST-1 complex | Endemic | South Africa | SV-5 | Opc+ |
| 29283 | 14563 | Neisseria meningitidis | A | ST-1 complex | Endemic | South Africa | SV-5 | Opc+ |
| 29284 | 12633 | Neisseria meningitidis | A | ST-1 complex | Endemic | South Africa | SV-5 | Opc+ |

| | | | | | | | | |
|---|---|---|---|---|---|---|---|---|
| 29285 | 15280 | Neisseria meningitidis | A | ST-1 complex | Endemic | South Africa | SV-5 | Opc+ |
| 29287 | 2666 | Neisseria meningitidis | C | ST-11 complex | Endemic | South Africa | SV-2 | Opc- |
| 29288 | 11100 | Neisseria meningitidis | A | ST-1 complex | Endemic | South Africa | SV-5 | Opc+ |
| 29290 | 9267 | Neisseria meningitidis | A | ST-1 complex | Endemic | South Africa | SV-5 | Opc+ |
| 29291 | 15003 | Neisseria meningitidis | A | ST-5 complex | Endemic | South Africa | SV-5 | Opc+ |
| 29292 | 17342 | Neisseria meningitidis | A | ST-1 complex | Endemic | South Africa | SV-5 | Opc+ |
| 29293 | 10890 | Neisseria meningitidis | A | ST-1 complex | Endemic | South Africa | SV-5 | Opc+ |
| 29295 | 14745 | Neisseria meningitidis | A | ST-1 complex | Endemic | South Africa | SV-5 | Opc+ |
| 29296 | 10352 | Neisseria meningitidis | A | ST-1 complex | Endemic | South Africa | SV-5 | Opc+ |
| 29297 | 10914 | Neisseria meningitidis | A | ST-1 complex | Endemic | South Africa | SV-5 | Opc+ |
| 29298 | 17718 | Neisseria meningitidis | A | ST-1 complex | Endemic | South Africa | SV-5 | Opc+ |
| 29299 | 16440 | Neisseria meningitidis | A | ST-1 complex | Endemic | South Africa | SV-5 | Opc+ |
| 29300 | 30407 | Neisseria meningitidis | A | ST-865 complex | Endemic | South Africa | SV-5 | Opc+ |
| 29301 | 16158 | Neisseria meningitidis | C | ST-1 complex | Endemic | South Africa | SV-5 | Opc+ |
| 29302 | 8828 | Neisseria meningitidis | A | ST-1 complex | Endemic | South Africa | SV-5 | Opc+ |
| 29303 | 9246 | Neisseria meningitidis | A | ST-1 complex | Endemic | South Africa | SV-5 | Opc+ |
| 29304 | 27937 | Neisseria meningitidis | A | ST-5 complex | Endemic | South Africa | SV-5 | Opc+ |
| 29305 | 37724 | Neisseria meningitidis | C | ST-11 complex | Endemic | South Africa | SV-2 | Opc- |
| 29306 | 27150 | Neisseria meningitidis | NG | No value | Endemic | South Africa | SV-1 | Opc- |
| 29307 | 16618_2 | Neisseria meningitidis | NG | ST-1 complex | No value | South Africa | SV-5 | Opc+ |
| 29308 | 36718 | Neisseria meningitidis | NG | No value | No value | South Africa | SV-11 | Opc- |
| 29309 | 37066 | Neisseria meningitidis | NG | ST-60 complex | Endemic | South Africa | SV-11 | Opc+ |
| 29310 | 38465 | Neisseria meningitidis | NG | ST-41/44 complex | Endemic | South Africa | SV-6 | Opc+ |
| 29311 | 38699 | Neisseria meningitidis | NG | ST-254 complex | Endemic | South Africa | SV-1 | Opc+ |
| 29312 | 5957 | Neisseria meningitidis | NG | ST-53 complex | Endemic | South Africa | SV-2 | Opc- |
| 29313 | 39319 | Neisseria meningitidis | W | ST-11 complex | Endemic | South Africa | SV-2 | Opc- |
| 29314 | 39619 | Neisseria meningitidis | W | ST-11 complex | Endemic | South Africa | SV-2 | Opc- |
| 29315 | 16661 | Neisseria meningitidis | W | ST-11 complex | Endemic | South Africa | SV-2 | Opc- |
| 29316 | 13283 | Neisseria meningitidis | W | ST-11 complex | Endemic | South Africa | SV-2 | Opc- |
| 29317 | 20033 | Neisseria meningitidis | W | ST-11 complex | Endemic | South Africa | SV-2 | Opc- |
| 29318 | 40328 | Neisseria meningitidis | W | ST-11 complex | Endemic | South Africa | SV-2 | Opc- |
| 29319 | 35477 | Neisseria meningitidis | W | ST-11 complex | Endemic | South Africa | SV-2 | Opc- |
| 29320 | 5453 | Neisseria meningitidis | W | ST-11 complex | Endemic | South Africa | SV-2 | Opc- |
| 29321 | 14493 | Neisseria meningitidis | W | ST-11 complex | Endemic | South Africa | SV-2 | Opc- |
| 29322 | 12198 | Neisseria meningitidis | W | ST-11 complex | Endemic | South Africa | SV-2 | Opc- |
| 29323 | 37252 | Neisseria meningitidis | W | ST-11 complex | Endemic | South Africa | SV-2 | Opc- |
| 29324 | 11361 | Neisseria meningitidis | W | ST-11 complex | Endemic | South Africa | SV-2 | Opc- |
| 29325 | 11665 | Neisseria meningitidis | W | ST-11 complex | Endemic | South Africa | SV-2 | Opc- |
| 29326 | 2266 | Neisseria meningitidis | W | ST-11 complex | Endemic | South Africa | SV-2 | Opc- |
| 29327 | 14983 | Neisseria meningitidis | W | ST-11 complex | Endemic | South Africa | SV-2 | Opc- |
| 29328 | 30301 | Neisseria meningitidis | W | ST-11 complex | Endemic | South Africa | SV-2 | Opc- |
| 29329 | 17690 | Neisseria meningitidis | W | ST-11 complex | Endemic | South Africa | SV-2 | Opc- |
| 29330 | 15984 | Neisseria meningitidis | W | ST-11 complex | Endemic | South Africa | SV-2 | Opc- |
| 29331 | 4846 | Neisseria meningitidis | W | ST-11 complex | Endemic | South Africa | SV-2 | Opc- |
| 29332 | 30147 | Neisseria meningitidis | W | ST-11 complex | Endemic | South Africa | SV-2 | Opc- |
| 29333 | 11924 | Neisseria meningitidis | W | ST-11 complex | Endemic | South Africa | SV-2 | Opc- |
| 29334 | 11009 | Neisseria meningitidis | W | ST-11 complex | Endemic | South Africa | SV-2 | Opc- |
| 29335 | 10384 | Neisseria meningitidis | W | ST-1 complex | Endemic | South Africa | SV-5 | Opc+ |
| 29336 | 29572 | Neisseria meningitidis | W | ST-11 complex | Endemic | South Africa | SV-2 | Opc- |
| 29337 | 1356 | Neisseria meningitidis | W | ST-11 complex | Endemic | South Africa | SV-2 | Opc- |
| 29338 | 8881 | Neisseria meningitidis | W | ST-11 complex | Endemic | South Africa | SV-2 | Opc- |
| 29339 | 21313 | Neisseria meningitidis | W | ST-11 complex | Endemic | South Africa | SV-2 | Opc- |
| 29340 | 17548 | Neisseria meningitidis | W | ST-11 complex | Endemic | South Africa | SV-2 | Opc- |
| 29341 | 10012 | Neisseria meningitidis | W | ST-11 complex | Endemic | South Africa | SV-2 | Opc- |
| 29342 | 15736 | Neisseria meningitidis | W | ST-11 complex | Endemic | South Africa | SV-2 | Opc- |
| 29343 | 32375 | Neisseria meningitidis | W | ST-23 complex | Endemic | South Africa | SV-11 | Opc+ |
| 29344 | 38107 | Neisseria meningitidis | W | ST-22 complex | Endemic | South Africa | SV-12 | Opc+ |
| 29345 | 28535 | Neisseria meningitidis | W | ST-167 complex | Endemic | South Africa | SV-5 | Opc+ |
| 29346 | 35813 | Neisseria meningitidis | W | ST-11 complex | Endemic | South Africa | SV-2 | Opc- |
| 29347 | 24836 | Neisseria meningitidis | W | ST-11 complex | Endemic | South Africa | SV-2 | Opc- |
| 29348 | 25348 | Neisseria meningitidis | W | ST-11 complex | Endemic | South Africa | SV-2 | Opc- |
| 29349 | 28444 | Neisseria meningitidis | W | ST-11 complex | Endemic | South Africa | SV-2 | Opc- |
| 29350 | 40923 | Neisseria meningitidis | W | ST-22 complex | Endemic | South Africa | SV-12 | Opc+ |
| 29351 | 5855 | Neisseria meningitidis | W | ST-11 complex | Endemic | South Africa | SV-2 | Opc- |
| 29352 | 40932 | Neisseria meningitidis | W | ST-22 complex | Endemic | South Africa | SV-12 | Opc+ |
| 29353 | 19309 | Neisseria meningitidis | W | ST-32 complex | Endemic | South Africa | SV-1 | Opc+ |
| 29355 | 25472 | Neisseria meningitidis | W | ST-11 complex | Endemic | South Africa | SV-2 | Opc- |
| 29356 | 21974 | Neisseria meningitidis | W | ST-11 complex | Endemic | South Africa | SV-2 | Opc- |
| 29357 | 15279 | Neisseria meningitidis | W | ST-11 complex | Endemic | South Africa | SV-2 | Opc- |
| 29358 | 15512 | Neisseria meningitidis | W | ST-11 complex | Endemic | South Africa | SV-2 | Opc- |
| 29359 | 18470 | Neisseria meningitidis | W | ST-11 complex | Endemic | South Africa | SV-2 | Opc- |
| 29360 | 35487 | Neisseria meningitidis | W | ST-11 complex | Endemic | South Africa | SV-2 | Opc- |
| 29361 | 33281 | Neisseria meningitidis | W | ST-11 complex | Endemic | South Africa | SV-2 | Opc- |
| 29362 | 10114 | Neisseria meningitidis | W | ST-11 complex | Endemic | South Africa | SV-2 | Opc- |

| | | | | | | | | |
|---|---|---|---|---|---|---|---|---|
| 29363 | 34709 | Neisseria meningitidis | W | ST-11 complex | Endemic | South Africa | SV-2 | Opc- |
| 29364 | 33509 | Neisseria meningitidis | W | ST-11 complex | Endemic | South Africa | SV-2 | Opc- |
| 29366 | 10992 | Neisseria meningitidis | W | ST-11 complex | Endemic | South Africa | SV-2 | Opc- |
| 29367 | 24204 | Neisseria meningitidis | W | ST-11 complex | Endemic | South Africa | SV-2 | Opc- |
| 29368 | 31847 | Neisseria meningitidis | W | ST-11 complex | Endemic | South Africa | SV-2 | Opc- |
| 29369 | 34873 | Neisseria meningitidis | W | ST-11 complex | Endemic | South Africa | SV-2 | Opc- |
| 29370 | 5032 | Neisseria meningitidis | W | ST-11 complex | Endemic | South Africa | SV-2 | Opc- |
| 29371 | 2725 | Neisseria meningitidis | W | ST-11 complex | Endemic | South Africa | SV-2 | Opc- |
| 29372 | 38108 | Neisseria meningitidis | W | ST-11 complex | Endemic | South Africa | SV-2 | Opc- |
| 29373 | 36146 | Neisseria meningitidis | W | ST-11 complex | Endemic | South Africa | SV-2 | Opc- |
| 29374 | 7764 | Neisseria meningitidis | W | ST-11 complex | Endemic | South Africa | SV-2 | Opc- |
| 29375 | 15766 | Neisseria meningitidis | W | ST-11 complex | Endemic | South Africa | SV-2 | Opc- |
| 29376 | 24581 | Neisseria meningitidis | W | ST-11 complex | Endemic | South Africa | SV-2 | Opc- |
| 29377 | 14395 | Neisseria meningitidis | W | ST-11 complex | Endemic | South Africa | SV-2 | Opc- |
| 29378 | 15781 | Neisseria meningitidis | W | ST-22 complex | Endemic | South Africa | SV-12 | Opc+ |
| 29379 | 24845 | Neisseria meningitidis | W | ST-11 complex | Endemic | South Africa | SV-2 | Opc- |
| 29380 | 9262 | Neisseria meningitidis | W | ST-11 complex | Endemic | South Africa | SV-2 | Opc- |
| 29381 | 16187 | Neisseria meningitidis | W | ST-11 complex | Endemic | South Africa | SV-2 | Opc- |
| 29382 | 3390 | Neisseria meningitidis | W | ST-11 complex | Endemic | South Africa | SV-2 | Opc- |
| 29383 | 16486 | Neisseria meningitidis | W | ST-11 complex | Endemic | South Africa | SV-2 | Opc- |
| 29384 | 4844 | Neisseria meningitidis | W | ST-11 complex | Endemic | South Africa | SV-2 | Opc- |
| 29385 | 21296 | Neisseria meningitidis | W | ST-11 complex | Endemic | South Africa | SV-2 | Opc- |
| 29386 | 28517 | Neisseria meningitidis | W | ST-11 complex | Endemic | South Africa | SV-2 | Opc- |
| 29387 | 41003 | Neisseria meningitidis | W | ST-11 complex | Endemic | South Africa | SV-2 | Opc- |
| 29388 | 30494 | Neisseria meningitidis | W | ST-11 complex | Endemic | South Africa | SV-2 | Opc- |
| 29389 | 35371 | Neisseria meningitidis | W | ST-11 complex | Endemic | South Africa | SV-2 | Opc- |
| 29390 | 37450 | Neisseria meningitidis | W | ST-11 complex | Endemic | South Africa | SV-2 | Opc- |
| 29391 | 23212 | Neisseria meningitidis | W | ST-32 complex | Endemic | South Africa | SV-1 | Opc+ |
| 29393 | 19208 | Neisseria meningitidis | W | ST-11 complex | Endemic | South Africa | SV-2 | Opc- |
| 29394 | 14673 | Neisseria meningitidis | W | ST-11 complex | Endemic | South Africa | SV-2 | Opc- |
| 29395 | 17195 | Neisseria meningitidis | W | ST-11 complex | Endemic | South Africa | SV-2 | Opc- |
| 29396 | 40559 | Neisseria meningitidis | W | ST-11 complex | Endemic | South Africa | SV-2 | Opc- |
| 29397 | 7313 | Neisseria meningitidis | W | ST-11 complex | Endemic | South Africa | SV-2 | Opc- |
| 29398 | 10093 | Neisseria meningitidis | W | ST-11 complex | Endemic | South Africa | SV-2 | Opc- |
| 29399 | 24344 | Neisseria meningitidis | W | ST-11 complex | Endemic | South Africa | SV-2 | Opc- |
| 29400 | 34047 | Neisseria meningitidis | W | ST-11 complex | Endemic | South Africa | SV-2 | Opc- |
| 29401 | 3308 | Neisseria meningitidis | W | ST-11 complex | Endemic | South Africa | SV-2 | Opc- |
| 29402 | 40498 | Neisseria meningitidis | W | ST-11 complex | Endemic | South Africa | SV-2 | Opc- |
| 29403 | 15642 | Neisseria meningitidis | W | ST-22 complex | Endemic | South Africa | SV-12 | Opc+ |
| 29404 | 16059 | Neisseria meningitidis | W | ST-11 complex | Endemic | South Africa | SV-2 | Opc- |
| 29405 | 36527 | Neisseria meningitidis | W | ST-11 complex | Endemic | South Africa | SV-2 | Opc- |
| 29406 | 15823 | Neisseria meningitidis | W | ST-11 complex | Endemic | South Africa | SV-2 | Opc- |
| 29407 | 33822 | Neisseria meningitidis | W | ST-11 complex | Endemic | South Africa | SV-2 | Opc- |
| 29408 | 21822 | Neisseria meningitidis | W | ST-11 complex | Endemic | South Africa | SV-2 | Opc- |
| 29409 | 12782 | Neisseria meningitidis | W | ST-11 complex | Endemic | South Africa | SV-2 | Opc- |
| 29410 | 15783 | Neisseria meningitidis | W | ST-11 complex | Endemic | South Africa | SV-2 | Opc- |
| 29411 | 16421 | Neisseria meningitidis | W | ST-11 complex | Endemic | South Africa | SV-2 | Opc- |
| 29412 | 28319 | Neisseria meningitidis | W | ST-11 complex | Endemic | South Africa | SV-2 | Opc- |
| 29413 | 5523 | Neisseria meningitidis | W | ST-11 complex | Endemic | South Africa | SV-2 | Opc- |
| 29414 | 28189 | Neisseria meningitidis | W | ST-11 complex | Endemic | South Africa | SV-2 | Opc- |
| 29415 | 17561 | Neisseria meningitidis | W | ST-11 complex | Endemic | South Africa | SV-2 | Opc- |
| 29416 | 19923 | Neisseria meningitidis | W | ST-11 complex | Endemic | South Africa | SV-2 | Opc- |
| 29417 | 29246 | Neisseria meningitidis | W | ST-11 complex | Endemic | South Africa | SV-2 | Opc- |
| 29418 | 34455 | Neisseria meningitidis | W | ST-11 complex | Endemic | South Africa | SV-2 | Opc- |
| 29419 | 13719 | Neisseria meningitidis | W | ST-11 complex | Endemic | South Africa | SV-2 | Opc- |
| 29420 | 31810 | Neisseria meningitidis | W | ST-11 complex | Endemic | South Africa | SV-2 | Opc- |
| 29421 | 8373 | Neisseria meningitidis | W | ST-11 complex | Endemic | South Africa | SV-2 | Opc- |
| 29422 | 3025 | Neisseria meningitidis | W | ST-11 complex | Endemic | South Africa | SV-2 | Opc- |
| 29423 | 41043 | Neisseria meningitidis | W | ST-11 complex | Endemic | South Africa | SV-2 | Opc- |
| 29424 | 15663 | Neisseria meningitidis | W | ST-11 complex | Endemic | South Africa | SV-2 | Opc- |
| 29425 | 26790 | Neisseria meningitidis | W | ST-11 complex | Endemic | South Africa | SV-2 | Opc- |
| 29426 | 7720 | Neisseria meningitidis | W | ST-11 complex | Endemic | South Africa | SV-2 | Opc- |
| 29427 | 7789 | Neisseria meningitidis | W | ST-11 complex | Endemic | South Africa | SV-2 | Opc- |
| 29428 | 33702 | Neisseria meningitidis | W | ST-11 complex | Endemic | South Africa | SV-2 | Opc- |
| 29429 | 1237 | Neisseria meningitidis | A | ST-1 complex | Endemic | South Africa | SV-5 | Opc+ |
| 29431 | 16619 | Neisseria meningitidis | A | ST-1 complex | Endemic | South Africa | SV-5 | Opc+ |
| 29432 | 11178 | Neisseria meningitidis | A | ST-1 complex | Endemic | South Africa | SV-5 | Opc+ |
| 29433 | 15883 | Neisseria meningitidis | A | ST-1 complex | Endemic | South Africa | SV-5 | Opc+ |
| 29434 | 139 | Neisseria meningitidis | A | ST-5 complex | Endemic | South Africa | SV-5 | Opc+ |
| 29435 | 22774 | Neisseria meningitidis | W | ST-11 complex | Endemic | South Africa | SV-2 | Opc- |
| 29436 | 16539 | Neisseria meningitidis | A | No value | Endemic | South Africa | SV-5 | Opc+ |
| 29437 | 6993 | Neisseria meningitidis | W | ST-11 complex | Endemic | South Africa | SV-2 | Opc- |
| 29438 | 14301 | Neisseria meningitidis | W | ST-11 complex | Endemic | South Africa | SV-2 | Opc- |
| 29439 | 10803 | Neisseria meningitidis | W | ST-11 complex | Endemic | South Africa | SV-2 | Opc- |

| | | | | | | | | |
|---|---|---|---|---|---|---|---|---|
| 29440 | 10378 | Neisseria meningitidis | W | ST-175 complex | Endemic | South Africa | SV-5 | Opc+ |
| 29441 | 19733 | Neisseria meningitidis | W | ST-11 complex | Endemic | South Africa | SV-2 | Opc- |
| 29442 | 1178 | Neisseria meningitidis | A | ST-1 complex | Endemic | South Africa | SV-5 | Opc+ |
| 29453 | S4 | Neisseria meningitidis | NG | ST-11 complex | No value | Spain | SV-2 | Opc- |
| 29454 | S4B1 | Neisseria meningitidis | NG | ST-11 complex | No value | Spain | SV-2 | Opc- |
| 29455 | S4B2 | Neisseria meningitidis | NG | ST-11 complex | No value | Spain | SV-2 | Opc- |
| 29456 | S4B3 | Neisseria meningitidis | NG | ST-11 complex | No value | Spain | SV-2 | Opc- |
| 29457 | S4Sm1 | Neisseria meningitidis | NG | ST-11 complex | No value | Spain | SV-2 | Opc- |
| 29458 | S4Sm2 | Neisseria meningitidis | NG | ST-11 complex | No value | Spain | SV-2 | Opc- |
| 29459 | S4Sm3 | Neisseria meningitidis | NG | ST-11 complex | No value | Spain | SV-2 | Opc- |
| 29460 | GRW135_strC | Neisseria meningitidis | NG | ST-11 complex | No value | Greece | SV-2 | Opc- |
| 29571 | M04 240932 | Neisseria meningitidis | C | ST-11 complex | No value | UK | SV-2 | Opc- |
| 29572 | M04 240003 | Neisseria meningitidis | C | ST-11 complex | No value | UK | SV-2 | Opc- |
| 29573 | M04 240019 | Neisseria meningitidis | C | ST-11 complex | No value | UK | SV-2 | Opc- |
| 29574 | M04 240020 | Neisseria meningitidis | C | ST-11 complex | No value | UK | SV-2 | Opc- |
| 29575 | M04 240029 | Neisseria meningitidis | C | ST-11 complex | No value | UK | SV-2 | Opc- |
| 29576 | M04 240065 | Neisseria meningitidis | C | ST-11 complex | No value | UK | SV-2 | Opc- |
| 29577 | M04 240078 | Neisseria meningitidis | C | ST-11 complex | No value | UK | SV-2 | Opc- |
| 29578 | M04 240129 | Neisseria meningitidis | C | ST-11 complex | No value | UK | SV-2 | Opc- |
| 29579 | M04 240207 | Neisseria meningitidis | C | No value | No value | Ireland | SV-5 | Opc- |
| 29580 | M04 240235 | Neisseria meningitidis | C | ST-11 complex | No value | UK | SV-2 | Opc- |
| 29581 | M04 240259 | Neisseria meningitidis | C | ST-11 complex | No value | UK | SV-2 | Opc- |
| 29582 | M04 240323 | Neisseria meningitidis | C | ST-11 complex | No value | UK | SV-2 | Opc- |
| 29583 | M04 240335 | Neisseria meningitidis | C | ST-11 complex | No value | UK | SV-2 | Opc- |
| 29584 | M04 240348 | Neisseria meningitidis | C | ST-11 complex | No value | UK | SV-2 | Opc- |
| 29585 | M04 240594 | Neisseria meningitidis | C | ST-11 complex | No value | UK | SV-2 | Opc- |
| 29586 | M04 240752 | Neisseria meningitidis | C | ST-11 complex | No value | UK | SV-2 | Opc- |
| 29587 | M04 240931 | Neisseria meningitidis | C | ST-11 complex | No value | UK | SV-2 | Opc- |
| 29590 | M04 241209 | Neisseria meningitidis | C | ST-11 complex | No value | UK | SV-2 | Opc- |
| 29591 | M04 241276 | Neisseria meningitidis | C | No value | No value | UK | SV-2 | Opc- |
| 29592 | M04 241501 | Neisseria meningitidis | C | ST-11 complex | No value | UK | SV-2 | Opc- |
| 29593 | M04 241542 | Neisseria meningitidis | C | ST-11 complex | No value | UK | SV-2 | Opc- |
| 29595 | M04 241722 | Neisseria meningitidis | C | ST-11 complex | No value | UK | SV-2 | Opc- |
| 29596 | M05 240080 | Neisseria meningitidis | C | ST-11 complex | No value | UK | SV-2 | Opc- |
| 29598 | M05 240258 | Neisseria meningitidis | C | ST-11 complex | No value | Ireland | SV-2 | Opc- |
| 29600 | M05 240316 | Neisseria meningitidis | C | ST-11 complex | No value | UK | SV-2 | Opc- |
| 29601 | M05 240490 | Neisseria meningitidis | C | ST-11 complex | No value | UK | SV-2 | Opc- |
| 29605 | M05 240920 | Neisseria meningitidis | C | ST-11 complex | No value | Ireland | SV-2 | Opc- |
| 29606 | M05 240972 | Neisseria meningitidis | C | ST-11 complex | No value | UK | SV-2 | Opc- |
| 29607 | M05 240990 | Neisseria meningitidis | C | ST-11 complex | No value | Ireland | SV-2 | Opc- |
| 29608 | M05 241164 | Neisseria meningitidis | C | ST-11 complex | No value | UK | SV-2 | Opc- |
| 29609 | M05 241241 | Neisseria meningitidis | C | ST-11 complex | No value | Ireland | SV-2 | Opc- |
| 29610 | M05 241361 | Neisseria meningitidis | C | ST-11 complex | No value | UK | SV-2 | Opc- |
| 29611 | M06 240019 | Neisseria meningitidis | C | ST-11 complex | No value | UK | SV-2 | Opc- |
| 29612 | M06 240067 | Neisseria meningitidis | C | ST-11 complex | No value | UK | SV-2 | Opc- |
| 29613 | M06 240167 | Neisseria meningitidis | C | ST-11 complex | No value | UK | SV-2 | Opc- |
| 29615 | M06 240183 | Neisseria meningitidis | C | ST-11 complex | No value | UK | SV-2 | Opc- |
| 29616 | M06 240340 | Neisseria meningitidis | C | ST-11 complex | No value | Malta | SV-2 | Opc- |
| 29617 | M06 240375 | Neisseria meningitidis | C | ST-11 complex | No value | UK | SV-2 | Opc- |
| 29618 | M06 240493 | Neisseria meningitidis | C | ST-11 complex | No value | Ireland | SV-2 | Opc- |
| 29619 | M06 240536 | Neisseria meningitidis | C | ST-11 complex | No value | UK | SV-2 | Opc- |
| 29620 | M06 240537 | Neisseria meningitidis | C | ST-11 complex | No value | UK | SV-2 | Opc- |
| 29621 | M06 240625 | Neisseria meningitidis | C | ST-11 complex | No value | UK | SV-2 | Opc- |
| 29624 | M06 240168 | Neisseria meningitidis | C | ST-11 complex | No value | UK | SV-2 | Opc- |
| 29625 | M07 240006 | Neisseria meningitidis | C | ST-11 complex | No value | UK | SV-2 | Opc- |
| 29626 | M07 240686 | Neisseria meningitidis | C | ST-11 complex | No value | UK | SV-2 | Opc- |
| 29627 | M07 240723 | Neisseria meningitidis | C | ST-11 complex | No value | UK | SV-2 | Opc- |
| 29628 | M07 240924 | Neisseria meningitidis | C | ST-11 complex | No value | UK | SV-2 | Opc- |
| 29629 | M07 240954 | Neisseria meningitidis | C | ST-11 complex | No value | UK | SV-2 | Opc- |
| 29631 | M07 241093 | Neisseria meningitidis | C | ST-11 complex | No value | UK | SV-2 | Opc- |
| 29633 | M07 240210 | Neisseria meningitidis | C | ST-11 complex | No value | UK | SV-2 | Opc- |
| 29635 | M07 240351 | Neisseria meningitidis | C | ST-11 complex | No value | UK | SV-2 | Opc- |
| 29636 | M07 240541 | Neisseria meningitidis | C | ST-11 complex | No value | UK | SV-2 | Opc- |
| 29637 | M07 240593 | Neisseria meningitidis | C | ST-11 complex | No value | UK | SV-2 | Opc- |
| 29638 | M08 240026 | Neisseria meningitidis | C | ST-11 complex | No value | UK | SV-2 | Opc- |
| 29639 | M08 240185 | Neisseria meningitidis | C | ST-11 complex | No value | UK | SV-2 | Opc- |
| 29640 | M08 240201 | Neisseria meningitidis | C | ST-11 complex | No value | UK | SV-2 | Opc- |
| 29641 | M08 240231 | Neisseria meningitidis | C | ST-11 complex | No value | UK | SV-2 | Opc- |
| 29642 | M08 240270 | Neisseria meningitidis | C | No value | No value | UK | SV-2 | Opc- |
| 29643 | M97 253597 | Neisseria meningitidis | C | ST-11 complex | No value | UK | SV-2 | Opc- |
| 29644 | M98 250473 | Neisseria meningitidis | C | ST-11 complex | No value | UK | SV-2 | Opc- |
| 29645 | M98 251974 | Neisseria meningitidis | C | ST-8 complex | No value | UK | SV-10 | Opc- |
| 29646 | M98 253000 | Neisseria meningitidis | C | ST-11 complex | No value | UK | SV-2 | Opc- |
| 29647 | M98 252341 | Neisseria meningitidis | NG | ST-11 complex | No value | UK | SV-2 | Opc- |

| | | | | | | | | |
|---|---|---|---|---|---|---|---|---|
| 29648 | M98 252422 | Neisseria meningitidis | W | ST-11 complex | No value | UK | SV-2 | Opc- |
| 29649 | M98 252412 | Neisseria meningitidis | W | ST-11 complex | No value | UK | SV-2 | Opc- |
| 29650 | M98 252490 | Neisseria meningitidis | W | ST-11 complex | No value | UK | SV-12 | Opc- |
| 29651 | M98 252407 | Neisseria meningitidis | W | ST-11 complex | No value | UK | SV-2 | Opc- |
| 29652 | M98 252491 | Neisseria meningitidis | W | ST-11 complex | No value | UK | SV-12 | Opc- |
| 29653 | M98 252431 | Neisseria meningitidis | W | ST-11 complex | No value | UK | SV-2 | Opc- |
| 29654 | M99 240321 | Neisseria meningitidis | C | ST-11 complex | No value | UK | SV-2 | Opc- |
| 29655 | M99 241273 | Neisseria meningitidis | C | ST-11 complex | No value | UK | SV-2 | Opc- |
| 29656 | M99 241594 | Neisseria meningitidis | C | ST-11 complex | No value | UK | SV-2 | Opc- |
| 29657 | M99 241396 | Neisseria meningitidis | NG | ST-60 complex | No value | UK | SV-12 | Opc+ |
| 29658 | M99 241667 | Neisseria meningitidis | C | ST-11 complex | No value | UK | SV-2 | Opc- |
| 29661 | M99 243927 | Neisseria meningitidis | NG | ST-11 complex | No value | UK | SV-2 | Opc- |
| 29662 | M00 240085 | Neisseria meningitidis | C | ST-8 complex | No value | UK | SV-10 | Opc- |
| 29663 | M00 240609 | Neisseria meningitidis | C | ST-11 complex | No value | UK | SV-2 | Opc- |
| 29664 | M00 240972 | Neisseria meningitidis | C | ST-11 complex | No value | UK | SV-2 | Opc- |
| 29665 | M00 241315 | Neisseria meningitidis | C | ST-11 complex | No value | UK | SV-2 | Opc- |
| 29666 | M00 241348 | Neisseria meningitidis | C | ST-11 complex | No value | UK | SV-2 | Opc- |
| 29667 | M00 241657 | Neisseria meningitidis | C | ST-11 complex | No value | UK | SV-2 | Opc- |
| 29668 | M00 241980 | Neisseria meningitidis | C | ST-11 complex | No value | UK | SV-2 | Opc- |
| 29669 | M00 242028 | Neisseria meningitidis | NG | ST-11 complex | No value | UK | SV-2 | Opc- |
| 29670 | M00 243009 | Neisseria meningitidis | B | ST-11 complex | No value | UK | SV-2 | Opc- |
| 29671 | M00 243130 | Neisseria meningitidis | B | ST-11 complex | No value | UK | SV-2 | Opc- |
| 29672 | M01 240219 | Neisseria meningitidis | C | ST-11 complex | No value | UK | SV-2 | Opc- |
| 29673 | M01 241306 | Neisseria meningitidis | C | ST-11 complex | No value | UK | SV-2 | Opc- |
| 29674 | M01 241431 | Neisseria meningitidis | C | ST-11 complex | No value | UK | SV-2 | Opc- |
| 29676 | M01 241826 | Neisseria meningitidis | NG | ST-11 complex | No value | UK | SV-2 | Opc- |
| 29677 | M01 242608 | Neisseria meningitidis | W | ST-11 complex | No value | UK | SV-2 | Opc- |
| 29678 | M01 242544 | Neisseria meningitidis | NG | ST-11 complex | No value | UK | SV-2 | Opc- |
| 29679 | M01 242679 | Neisseria meningitidis | W | ST-11 complex | No value | UK | SV-2 | Opc- |
| 29680 | M01 242591 | Neisseria meningitidis | W | ST-11 complex | No value | UK | SV-2 | Opc- |
| 29681 | M01 242700 | Neisseria meningitidis | W | ST-11 complex | No value | UK | SV-2 | Opc- |
| 29682 | M02 240026 | Neisseria meningitidis | W | ST-22 complex | No value | UK | SV-12 | Opc+ |
| 29683 | M02 240124 | Neisseria meningitidis | W | ST-11 complex | No value | UK | SV-2 | Opc- |
| 29684 | M01 242717 | Neisseria meningitidis | W | ST-11 complex | No value | UK | SV-2 | Opc- |
| 29686 | M02 240227 | Neisseria meningitidis | W | ST-11 complex | No value | UK | SV-2 | Opc- |
| 29687 | M02 240235 | Neisseria meningitidis | W | ST-22 complex | No value | UK | SV-12 | Opc+ |
| 29688 | M02 240422 | Neisseria meningitidis | W | ST-11 complex | No value | UK | SV-2 | Opc- |
| 29689 | M02 240440 | Neisseria meningitidis | W | ST-11 complex | No value | UK | SV-2 | Opc- |
| 29690 | M02 240609 | Neisseria meningitidis | W | ST-11 complex | No value | UK | SV-2 | Opc- |
| 29691 | M02 240704 | Neisseria meningitidis | NG | No value | No value | UK | SV-12 | Opc+ |
| 29692 | M02 240839 | Neisseria meningitidis | W | ST-11 complex | No value | UK | SV-2 | Opc- |
| 29693 | M02 241029 | Neisseria meningitidis | C | ST-11 complex | No value | UK | SV-2 | Opc- |
| 29694 | M02 241321 | Neisseria meningitidis | W | ST-11 complex | No value | UK | SV-2 | Opc- |
| 29695 | M02 241603 | Neisseria meningitidis | W | ST-11 complex | No value | UK | SV-2 | Opc- |
| 29696 | M02 241666 | Neisseria meningitidis | W | ST-11 complex | No value | UK | SV-2 | Opc- |
| 29697 | M02 241746 | Neisseria meningitidis | W | ST-11 complex | No value | UK | SV-2 | Opc- |
| 29698 | M02 242123 | Neisseria meningitidis | W | ST-22 complex | No value | UK | SV-12 | Opc+ |
| 29699 | M03 240692 | Neisseria meningitidis | W | ST-11 complex | No value | UK | SV-2 | Opc- |
| 29700 | M03 240696 | Neisseria meningitidis | W | ST-11 complex | No value | UK | SV-2 | Opc- |
| 29702 | M04 240429 | Neisseria meningitidis | W | ST-11 complex | No value | UK | SV-2 | Opc- |
| 29703 | M04 240435 | Neisseria meningitidis | W | ST-11 complex | No value | UK | SV-2 | Opc- |
| 29704 | M05 241067 | Neisseria meningitidis | W | ST-11 complex | No value | UK | SV-2 | Opc- |
| 29705 | M06 240459 | Neisseria meningitidis | W | ST-11 complex | No value | UK | SV-2 | Opc- |
| 29706 | M07 240001 | Neisseria meningitidis | W | No value | No value | UK | SV-12 | Opc+ |
| 29707 | M07 240118 | Neisseria meningitidis | W | ST-11 complex | No value | UK | SV-2 | Opc- |
| 29709 | M07 240774 | Neisseria meningitidis | W | ST-11 complex | No value | UK | SV-2 | Opc- |
| 29710 | M07 240922 | Neisseria meningitidis | W | ST-11 complex | No value | UK | SV-2 | Opc- |
| 29711 | M07 240489 | Neisseria meningitidis | W | ST-22 complex | No value | UK | SV-12 | Opc+ |
| 29712 | M09 240368 | Neisseria meningitidis | W | ST-22 complex | No value | UK | SV-12 | Opc+ |
| 29713 | M09 240316 | Neisseria meningitidis | W | No value | No value | UK | SV-12 | Opc+ |
| 29714 | M10 240005 | Neisseria meningitidis | W | ST-11 complex | No value | UK | SV-2 | Opc- |
| 29715 | M09 240862 | Neisseria meningitidis | W | ST-11 complex | No value | UK | SV-2 | Opc- |
| 29716 | M09 240900 | Neisseria meningitidis | W | ST-11 complex | No value | UK | SV-2 | Opc- |
| 29717 | M10 240134 | Neisseria meningitidis | NG | ST-60 complex | No value | UK | SV-11 | Opc+ |
| 29718 | M10 240389 | Neisseria meningitidis | W | ST-11 complex | No value | UK | SV-2 | Opc- |
| 29719 | M10 240452 | Neisseria meningitidis | W | ST-11 complex | No value | UK | SV-2 | Opc- |
| 29720 | M10 240696 | Neisseria meningitidis | W | ST-11 complex | No value | UK | SV-2 | Opc- |
| 29721 | M13 240491 | Neisseria meningitidis | W | ST-11 complex | No value | UK | SV-2 | Opc- |
| 29722 | M13 240530 | Neisseria meningitidis | W | ST-11 complex | No value | UK | SV-2 | Opc- |
| 29723 | M13 240555 | Neisseria meningitidis | W | ST-11 complex | No value | UK | SV-2 | Opc- |
| 29724 | M13 240591 | Neisseria meningitidis | W | ST-11 complex | No value | UK | SV-2 | Opc- |
| 29725 | M13 240603 | Neisseria meningitidis | W | ST-11 complex | No value | UK | SV-2 | Opc- |
| 29726 | M13 240604 | Neisseria meningitidis | W | ST-11 complex | No value | UK | SV-2 | Opc- |
| 29727 | M13 240545 | Neisseria meningitidis | W | ST-11 complex | No value | UK | SV-2 | Opc- |

| | | | | | | | | |
|---|---|---|---|---|---|---|---|---|
| 29728 | M13 240558 | Neisseria meningitidis | W | ST-11 complex | No value | UK | SV-2 | Opc- |
| 29729 | M13 240594 | Neisseria meningitidis | W | ST-11 complex | No value | UK | SV-2 | Opc- |
| 29730 | M13 240583 | Neisseria meningitidis | W | ST-11 complex | No value | UK | SV-2 | Opc- |
| 29731 | M13 240510 | Neisseria meningitidis | W | ST-11 complex | No value | UK | SV-2 | Opc- |
| 29732 | M13 240633 | Neisseria meningitidis | W | ST-11 complex | No value | UK | SV-2 | Opc- |
| 29733 | M13 240634 | Neisseria meningitidis | W | ST-11 complex | No value | UK | SV-2 | Opc- |
| 29734 | M13 240538 | Neisseria meningitidis | W | ST-11 complex | No value | UK | SV-2 | Opc- |
| 29735 | M13 240515 | Neisseria meningitidis | W | ST-11 complex | No value | UK | SV-2 | Opc- |
| 29736 | M13 240600 | Neisseria meningitidis | W | ST-11 complex | No value | UK | SV-2 | Opc- |
| 29737 | M13 240553 | Neisseria meningitidis | W | ST-11 complex | No value | UK | SV-2 | Opc- |
| 29738 | M97 252092 | Neisseria meningitidis | C | ST-11 complex | No value | UK | SV-2 | Opc- |
| 29739 | M97 252093 | Neisseria meningitidis | C | ST-11 complex | No value | UK | SV-2 | Opc- |
| 29740 | M97 252094 | Neisseria meningitidis | C | ST-11 complex | No value | UK | SV-2 | Opc- |
| 29741 | M97 252240 | Neisseria meningitidis | B | ST-41/44 complex | No value | UK | SV-2 | Opc+ |
| 29742 | M97 252241 | Neisseria meningitidis | B | ST-41/44 complex | No value | UK | SV-2 | Opc+ |
| 29743 | M97 252242 | Neisseria meningitidis | B | ST-41/44 complex | No value | UK | SV-2 | Opc+ |
| 29744 | M98 250082 | Neisseria meningitidis | B | ST-41/44 complex | No value | UK | SV-10 | Opc+ |
| 29745 | M98 250087 | Neisseria meningitidis | B | ST-41/44 complex | No value | UK | SV-10 | Opc+ |
| 29746 | M98 250088 | Neisseria meningitidis | B | ST-41/44 complex | No value | UK | SV-10 | Opc+ |
| 29747 | M98 250375 | Neisseria meningitidis | B | ST-32 complex | No value | UK | SV-1 | Opc+ |
| 29748 | M98 250376 | Neisseria meningitidis | B | ST-32 complex | No value | UK | SV-1 | Opc+ |
| 29749 | M98 250377 | Neisseria meningitidis | B | ST-32 complex | No value | UK | SV-1 | Opc+ |
| 29750 | M98 250786 | Neisseria meningitidis | C | ST-11 complex | No value | UK | SV-2 | Opc- |
| 29751 | M98 250787 | Neisseria meningitidis | C | ST-11 complex | No value | UK | SV-2 | Opc- |
| 29752 | M98 250788 | Neisseria meningitidis | C | ST-11 complex | No value | UK | SV-2 | Opc- |
| 29753 | M98 250838 | Neisseria meningitidis | B | ST-60 complex | No value | UK | SV-11 | Opc+ |
| 29754 | M98 250839 | Neisseria meningitidis | B | ST-60 complex | No value | UK | SV-11 | Opc+ |
| 29755 | M98 250840 | Neisseria meningitidis | B | ST-60 complex | No value | UK | SV-11 | Opc+ |
| 29756 | M98 251153 | Neisseria meningitidis | B | ST-213 complex | No value | UK | SV-12 | Opc- |
| 29757 | M98 252086 | Neisseria meningitidis | B | ST-41/44 complex | No value | UK | SV-5 | Opc- |
| 29758 | M98 252087 | Neisseria meningitidis | B | ST-41/44 complex | No value | UK | SV-5 | Opc- |
| 29759 | M98 252088 | Neisseria meningitidis | B | ST-41/44 complex | No value | UK | SV-5 | Opc- |
| 29760 | M98 252222 | Neisseria meningitidis | C | ST-11 complex | No value | UK | SV-2 | Opc- |
| 29761 | M98 252223 | Neisseria meningitidis | C | ST-11 complex | No value | UK | SV-2 | Opc- |
| 29762 | M98 252224 | Neisseria meningitidis | C | ST-11 complex | No value | UK | SV-2 | Opc- |
| 29763 | M00 240046 | Neisseria meningitidis | B | ST-41/44 complex | No value | UK | SV-2 | Opc+ |
| 29764 | M00 240163 | Neisseria meningitidis | B | ST-41/44 complex | No value | UK | SV-2 | Opc+ |
| 29765 | M00 240207 | Neisseria meningitidis | NG | ST-41/44 complex | No value | UK | SV-2 | Opc+ |
| 29767 | M00 240858 | Neisseria meningitidis | B | ST-41/44 complex | No value | UK | SV-6 | Opc+ |
| 29768 | M00 240859 | Neisseria meningitidis | B | ST-41/44 complex | No value | UK | SV-6 | Opc+ |
| 29769 | M00 240860 | Neisseria meningitidis | B | ST-41/44 complex | No value | UK | SV-6 | Opc+ |
| 29770 | M00 240863 | Neisseria meningitidis | B | ST-41/44 complex | No value | UK | SV-6 | Opc+ |
| 29771 | M00 240877 | Neisseria meningitidis | B | ST-41/44 complex | No value | UK | SV-6 | Opc+ |
| 29772 | M00 240878 | Neisseria meningitidis | B | ST-41/44 complex | No value | UK | SV-6 | Opc+ |
| 29773 | M00 240993 | Neisseria meningitidis | B | ST-41/44 complex | No value | UK | SV-2 | Opc+ |
| 29774 | M00 240995 | Neisseria meningitidis | B | ST-41/44 complex | No value | UK | SV-2 | Opc+ |
| 29775 | M00 241395 | Neisseria meningitidis | W | ST-11 complex | No value | UK | SV-2 | Opc- |
| 29776 | M00 241416 | Neisseria meningitidis | W | ST-11 complex | No value | UK | SV-2 | Opc- |
| 29777 | M00 241428 | Neisseria meningitidis | B | ST-269 complex | No value | Ireland | SV-1 | Opc+ |
| 29778 | M00 241456 | Neisseria meningitidis | W | ST-11 complex | No value | UK | SV-2 | Opc- |
| 29779 | M00 241466 | Neisseria meningitidis | B | ST-269 complex | No value | Ireland | SV-1 | Opc+ |
| 29780 | M00 241467 | Neisseria meningitidis | B | ST-269 complex | No value | Ireland | SV-1 | Opc+ |
| 29784 | M00 242669 | Neisseria meningitidis | C | No value | No value | UK | SV-2 | Opc- |
| 29785 | M00 242710 | Neisseria meningitidis | C | No value | No value | UK | SV-2 | Opc- |
| 29787 | M00 242964 | Neisseria meningitidis | W | ST-22 complex | No value | UK | SV-12 | Opc+ |
| 29788 | M00 242965 | Neisseria meningitidis | W | ST-22 complex | No value | UK | SV-12 | Opc+ |
| 29789 | M01 241286 | Neisseria meningitidis | C | ST-11 complex | No value | UK | SV-2 | Opc- |
| 29790 | M01 241302 | Neisseria meningitidis | C | ST-11 complex | No value | UK | SV-2 | Opc- |
| 29792 | M01 241662 | Neisseria meningitidis | B | ST-269 complex | No value | UK | SV-1 | Opc+ |
| 29793 | M01 241663 | Neisseria meningitidis | B | ST-269 complex | No value | UK | SV-1 | Opc+ |
| 29794 | M01 241677 | Neisseria meningitidis | B | ST-269 complex | No value | UK | SV-1 | Opc+ |
| 29795 | M02 240168 | Neisseria meningitidis | B | ST-269 complex | No value | UK | SV-8 | Opc+ |
| 29796 | M03 240335 | Neisseria meningitidis | B | ST-18 complex | No value | UK | SV-7 | Opc- |
| 29797 | M03 240336 | Neisseria meningitidis | B | ST-18 complex | No value | UK | SV-7 | Opc- |
| 29798 | M03 240337 | Neisseria meningitidis | B | ST-18 complex | No value | UK | SV-7 | Opc- |
| 29800 | M03 241002 | Neisseria meningitidis | B | ST-41/44 complex | No value | UK | SV-2 | Opc+ |
| 29801 | M03 241003 | Neisseria meningitidis | B | ST-41/44 complex | No value | UK | SV-2 | Opc+ |
| 29802 | M03 241017 | Neisseria meningitidis | B | ST-41/44 complex | No value | UK | SV-2 | Opc+ |
| 29803 | M03 241456 | Neisseria meningitidis | Y | ST-23 complex | No value | UK | SV-11 | Opc+ |
| 29804 | M03 241474 | Neisseria meningitidis | Y | ST-23 complex | No value | UK | SV-11 | Opc+ |
| 29805 | M03 241475 | Neisseria meningitidis | Y | ST-23 complex | No value | UK | SV-11 | Opc+ |
| 29806 | M04 241708 | Neisseria meningitidis | B | No value | No value | UK | SV-1 | Opc+ |
| 29807 | M05 240177 | Neisseria meningitidis | B | ST-32 complex | No value | UK | SV-1 | Opc+ |
| 29808 | M05 240208 | Neisseria meningitidis | B | ST-32 complex | No value | UK | SV-1 | Opc+ |

| | | | | | | | | |
|---|---|---|---|---|---|---|---|---|
| 29809 | M05 240209 | Neisseria meningitidis | B | ST-32 complex | No value | UK | SV-1 | Opc+ |
| 29810 | M09 240012 | Neisseria meningitidis | B | ST-32 complex | No value | UK | SV-1 | Opc+ |
| 29811 | M09 240013 | Neisseria meningitidis | B | ST-32 complex | No value | UK | SV-1 | Opc+ |
| 29812 | M09 240022 | Neisseria meningitidis | B | ST-32 complex | No value | UK | SV-1 | Opc+ |
| 29813 | M11 240418 | Neisseria meningitidis | W | ST-11 complex | No value | UK | SV-2 | Opc- |
| 29814 | M11 240419 | Neisseria meningitidis | W | ST-11 complex | No value | UK | SV-2 | Opc- |
| 29816 | M12 240183 | Neisseria meningitidis | Y | ST-167 complex | No value | UK | SV-5 | Opc+ |
| 29817 | M98 252289 | Neisseria meningitidis | B | ST-32 complex | No value | UK | SV-1 | Opc+ |
| 29818 | M98 252328 | Neisseria meningitidis | B | ST-32 complex | No value | UK | SV-1 | Opc+ |
| 29819 | M98 252329 | Neisseria meningitidis | B | ST-32 complex | No value | UK | SV-1 | Opc+ |
| 29820 | M98 252927 | Neisseria meningitidis | C | ST-11 complex | No value | UK | SV-2 | Opc- |
| 29821 | M98 252928 | Neisseria meningitidis | C | ST-11 complex | No value | UK | SV-2 | Opc- |
| 29822 | M98 252947 | Neisseria meningitidis | C | ST-11 complex | No value | UK | SV-2 | Opc- |
| 29823 | M98 253067 | Neisseria meningitidis | B | ST-41/44 complex | No value | UK | SV-2 | Opc+ |
| 29824 | M98 253068 | Neisseria meningitidis | B | ST-41/44 complex | No value | UK | SV-2 | Opc+ |
| 29825 | M98 253069 | Neisseria meningitidis | B | ST-41/44 complex | No value | UK | SV-2 | Opc+ |
| 29826 | M98 253702 | Neisseria meningitidis | B | No value | No value | UK | SV-5 | Opc- |
| 29827 | M98 253703 | Neisseria meningitidis | B | No value | No value | UK | SV-5 | Opc- |
| 29828 | M98 253704 | Neisseria meningitidis | B | No value | No value | UK | SV-5 | Opc- |
| 29829 | M98 253766 | Neisseria meningitidis | B | ST-8 complex | No value | UK | SV-14 | Opc- |
| 29830 | M99 240021 | Neisseria meningitidis | B | ST-8 complex | No value | UK | SV-14 | Opc- |
| 29831 | M98 252173 | Neisseria meningitidis | B | ST-11 complex | No value | UK | SV-2 | Opc- |
| 29832 | M00 240186 | Neisseria meningitidis | C | ST-11 complex | No value | UK | SV-2 | Opc- |
| 29833 | M00 240320 | Neisseria meningitidis | C | ST-11 complex | No value | UK | SV-2 | Opc- |
| 29834 | M00 240543 | Neisseria meningitidis | C | ST-11 complex | No value | UK | SV-2 | Opc- |
| 29835 | M00 240680 | Neisseria meningitidis | C | ST-11 complex | No value | UK | SV-2 | Opc- |
| 29836 | M00 240845 | Neisseria meningitidis | C | ST-11 complex | No value | UK | SV-2 | Opc- |
| 29838 | M00 241306 | Neisseria meningitidis | C | ST-11 complex | No value | UK | SV-2 | Opc- |
| 29840 | M00 241761 | Neisseria meningitidis | C | ST-11 complex | No value | UK | SV-2 | Opc- |
| 29843 | M00 243016 | Neisseria meningitidis | C | ST-11 complex | No value | UK | SV-2 | Opc- |
| 29844 | M00 243289 | Neisseria meningitidis | C | ST-11 complex | No value | UK | SV-2 | Opc- |
| 29846 | M01 240426 | Neisseria meningitidis | C | ST-11 complex | No value | UK | SV-2 | Opc- |
| 29847 | M01 240526 | Neisseria meningitidis | C | ST-11 complex | No value | UK | SV-2 | Opc- |
| 29849 | M01 240493 | Neisseria meningitidis | C | ST-11 complex | No value | Ireland | SV-2 | Opc- |
| 29850 | M01 240514 | Neisseria meningitidis | C | ST-11 complex | No value | UK | SV-2 | Opc- |
| 29851 | M01 240850 | Neisseria meningitidis | C | ST-11 complex | No value | UK | SV-2 | Opc- |
| 29853 | M01 241276 | Neisseria meningitidis | C | ST-11 complex | No value | UK | SV-2 | Opc- |
| 29858 | M01 242751 | Neisseria meningitidis | C | ST-11 complex | No value | Ireland | SV-2 | Opc- |
| 29859 | M01 242752 | Neisseria meningitidis | C | ST-11 complex | No value | UK | SV-2 | Opc- |
| 29860 | M02 240005 | Neisseria meningitidis | C | ST-11 complex | No value | UK | SV-2 | Opc- |
| 29863 | M02 240385 | Neisseria meningitidis | C | ST-11 complex | No value | Ireland | SV-2 | Opc- |
| 29865 | M02 240206 | Neisseria meningitidis | C | ST-11 complex | No value | UK | SV-2 | Opc- |
| 29868 | M02 240945 | Neisseria meningitidis | C | ST-11 complex | No value | UK | SV-2 | Opc- |
| 29870 | M02 241124 | Neisseria meningitidis | C | ST-11 complex | No value | UK | SV-2 | Opc- |
| 29875 | M03 240453 | Neisseria meningitidis | C | ST-11 complex | No value | UK | SV-2 | Opc- |
| 29877 | M03 241704 | Neisseria meningitidis | C | ST-11 complex | No value | Ireland | SV-2 | Opc- |
| 29879 | M08 240142 | Neisseria meningitidis | C | ST-11 complex | No value | UK | SV-2 | Opc- |
| 29888 | M09 240249 | Neisseria meningitidis | C | ST-11 complex | No value | UK | SV-2 | Opc- |
| 29889 | M09 240241 | Neisseria meningitidis | C | ST-11 complex | No value | Malta | SV-2 | Opc- |
| 29890 | M10 240001 | Neisseria meningitidis | C | ST-11 complex | No value | UK | SV-2 | Opc- |
| 29891 | M10 240229 | Neisseria meningitidis | C | ST-11 complex | No value | UK | SV-2 | Opc- |
| 29895 | M13 240496 | Neisseria meningitidis | C | ST-11 complex | No value | UK | SV-2 | Opc- |
| 29896 | M13 240382 | Neisseria meningitidis | C | ST-11 complex | No value | Malta | SV-2 | Opc- |
| 29898 | M13 240514 | Neisseria meningitidis | C | ST-11 complex | No value | UK | SV-2 | Opc- |
| 29899 | M98 250423 | Neisseria meningitidis | C | ST-11 complex | No value | UK | SV-2 | Opc- |
| 29901 | M98 250863 | Neisseria meningitidis | C | ST-11 complex | No value | UK | SV-2 | Opc- |
| 29902 | M98 251032 | Neisseria meningitidis | C | ST-11 complex | No value | UK | SV-2 | Opc- |
| 29904 | M98 251593 | Neisseria meningitidis | C | ST-11 complex | No value | Ireland | SV-2 | Opc- |
| 29907 | M98 252898 | Neisseria meningitidis | C | ST-11 complex | No value | UK | SV-2 | Opc- |
| 29908 | M98 252918 | Neisseria meningitidis | C | ST-11 complex | No value | UK | SV-2 | Opc- |
| 29910 | M98 253743 | Neisseria meningitidis | C | ST-11 complex | No value | UK | SV-2 | Opc- |
| 29912 | M98 253765 | Neisseria meningitidis | C | ST-11 complex | No value | UK | SV-2 | Opc- |
| 29914 | M99 240413 | Neisseria meningitidis | C | ST-11 complex | No value | UK | SV-2 | Opc- |
| 29918 | M99 241412 | Neisseria meningitidis | C | ST-11 complex | No value | Ireland | SV-2 | Opc- |
| 29920 | M99 242207 | Neisseria meningitidis | C | ST-11 complex | No value | UK | SV-2 | Opc- |
| 29922 | M99 242522 | Neisseria meningitidis | C | ST-11 complex | No value | UK | SV-2 | Opc- |
| 29924 | M99 243272 | Neisseria meningitidis | C | ST-11 complex | No value | Ireland | SV-2 | Opc- |
| 29926 | M99 243956 | Neisseria meningitidis | C | ST-8 complex | No value | UK | SV-10 | Opc- |
| 29928 | M00 241341 | Neisseria meningitidis | W | ST-11 complex | No value | UK | SV-2 | Opc- |
| 29929 | M00 241352 | Neisseria meningitidis | W | ST-11 complex | No value | UK | SV-2 | Opc- |
| 29930 | M00 241357 | Neisseria meningitidis | W | ST-11 complex | No value | UK | SV-2 | Opc- |
| 29931 | M00 241362 | Neisseria meningitidis | W | ST-11 complex | No value | UK | SV-2 | Opc- |
| 29932 | M00 241380 | Neisseria meningitidis | W | ST-11 complex | No value | UK | SV-2 | Opc- |
| 29933 | M00 241396 | Neisseria meningitidis | W | ST-11 complex | No value | UK | SV-2 | Opc- |

| | | | | | | | | |
|---|---|---|---|---|---|---|---|---|
| 29934 | M00 241401 | Neisseria meningitidis | W | ST-11 complex | No value | UK | SV-2 | Opc- |
| 29935 | M00 241408 | Neisseria meningitidis | W | ST-11 complex | No value | UK | SV-2 | Opc- |
| 29936 | M00 241420 | Neisseria meningitidis | W | ST-11 complex | No value | UK | SV-2 | Opc- |
| 29937 | M00 241430 | Neisseria meningitidis | W | ST-11 complex | No value | UK | SV-2 | Opc- |
| 29938 | M00 241431 | Neisseria meningitidis | W | ST-11 complex | No value | UK | SV-2 | Opc- |
| 29939 | M00 241432 | Neisseria meningitidis | W | ST-11 complex | No value | UK | SV-2 | Opc- |
| 29940 | M00 241433 | Neisseria meningitidis | W | ST-11 complex | No value | UK | SV-2 | Opc- |
| 29941 | M00 241435 | Neisseria meningitidis | W | ST-11 complex | No value | UK | SV-2 | Opc- |
| 29942 | M00 241445 | Neisseria meningitidis | W | ST-11 complex | No value | UK | SV-2 | Opc- |
| 29943 | M00 241447 | Neisseria meningitidis | W | ST-11 complex | No value | UK | SV-2 | Opc- |
| 29944 | M00 241491 | Neisseria meningitidis | W | ST-11 complex | No value | UK | SV-2 | Opc- |
| 29945 | M00 241510 | Neisseria meningitidis | W | ST-11 complex | No value | UK | SV-2 | Opc- |
| 29946 | M00 241511 | Neisseria meningitidis | W | ST-11 complex | No value | UK | SV-2 | Opc- |
| 29947 | M00 241557 | Neisseria meningitidis | W | ST-11 complex | No value | UK | SV-2 | Opc- |
| 29948 | M00 241578 | Neisseria meningitidis | W | ST-11 complex | No value | UK | SV-2 | Opc- |
| 29949 | M00 241620 | Neisseria meningitidis | W | ST-11 complex | No value | UK | SV-2 | Opc- |
| 29950 | M00 241645 | Neisseria meningitidis | W | ST-11 complex | No value | UK | SV-2 | Opc- |
| 29951 | M00 241655 | Neisseria meningitidis | W | ST-11 complex | No value | UK | SV-2 | Opc- |
| 29952 | M00 241673 | Neisseria meningitidis | W | ST-11 complex | No value | UK | SV-2 | Opc- |
| 29953 | M00 241674 | Neisseria meningitidis | W | ST-11 complex | No value | UK | SV-2 | Opc- |
| 29954 | M00 241675 | Neisseria meningitidis | W | ST-11 complex | No value | UK | SV-2 | Opc- |
| 29955 | M00 241704 | Neisseria meningitidis | W | ST-11 complex | No value | UK | SV-2 | Opc- |
| 29956 | M00 241759 | Neisseria meningitidis | W | ST-11 complex | No value | UK | SV-2 | Opc- |
| 29957 | M00 241760 | Neisseria meningitidis | W | ST-11 complex | No value | UK | SV-2 | Opc- |
| 29958 | M00 241800 | Neisseria meningitidis | W | ST-11 complex | No value | UK | SV-2 | Opc- |
| 29959 | M00 241833 | Neisseria meningitidis | W | ST-11 complex | No value | UK | SV-2 | Opc- |
| 29960 | M00 241894 | Neisseria meningitidis | W | ST-11 complex | No value | UK | SV-2 | Opc- |
| 29961 | M00 241957 | Neisseria meningitidis | W | ST-11 complex | No value | UK | SV-2 | Opc- |
| 29962 | M00 241978 | Neisseria meningitidis | W | ST-11 complex | No value | UK | SV-2 | Opc- |
| 29963 | M00 241986 | Neisseria meningitidis | W | ST-11 complex | No value | UK | SV-2 | Opc- |
| 29964 | M00 242029 | Neisseria meningitidis | W | ST-11 complex | No value | UK | SV-2 | Opc- |
| 29965 | M00 242295 | Neisseria meningitidis | W | ST-11 complex | No value | UK | SV-2 | Opc- |
| 29966 | M00 242531 | Neisseria meningitidis | W | ST-11 complex | No value | UK | SV-2 | Opc- |
| 29967 | M00 242667 | Neisseria meningitidis | W | ST-11 complex | No value | UK | SV-2 | Opc- |
| 29968 | M00 242770 | Neisseria meningitidis | W | ST-11 complex | No value | UK | SV-2 | Opc- |
| 29969 | M00 242916 | Neisseria meningitidis | W | ST-11 complex | No value | UK | SV-2 | Opc- |
| 29970 | M00 242974 | Neisseria meningitidis | W | ST-11 complex | No value | UK | SV-2 | Opc- |
| 29975 | M03 240862 | Neisseria meningitidis | C | No value | No value | UK | SV-2 | Opc- |
| 29976 | M03 241118 | Neisseria meningitidis | C | ST-11 complex | No value | UK | SV-2 | Opc- |
| 29977 | M03 241273 | Neisseria meningitidis | C | ST-11 complex | No value | UK | SV-2 | Opc- |
| 29978 | M03 241465 | Neisseria meningitidis | C | ST-11 complex | No value | UK | SV-2 | Opc- |
| 29979 | M03 241792 | Neisseria meningitidis | C | ST-11 complex | No value | UK | SV-2 | Opc- |
| 29982 | M98 251316 | Neisseria meningitidis | B | ST-11 complex | No value | UK | SV-2 | Opc- |
| 29984 | M99 240124 | Neisseria meningitidis | B | ST-11 complex | No value | UK | SV-2 | Opc- |
| 29987 | M99 243153 | Neisseria meningitidis | B | ST-213 complex | No value | UK | SV-17 | Opc- |
| 29988 | M00 240440 | Neisseria meningitidis | B | ST-11 complex | No value | UK | SV-2 | Opc- |
| 29990 | M00 240815 | Neisseria meningitidis | B | ST-11 complex | No value | UK | SV-2 | Opc- |
| 29991 | M00 243175 | Neisseria meningitidis | W | ST-11 complex | No value | UK | SV-2 | Opc- |
| 29992 | M00 243297 | Neisseria meningitidis | W | ST-11 complex | No value | UK | SV-2 | Opc- |
| 29993 | M01 240025 | Neisseria meningitidis | B | ST-11 complex | No value | UK | SV-2 | Opc- |
| 29994 | M01 240028 | Neisseria meningitidis | W | ST-11 complex | No value | UK | SV-2 | Opc- |
| 29995 | M01 240168 | Neisseria meningitidis | W | ST-11 complex | No value | UK | SV-2 | Opc- |
| 29996 | M01 240240 | Neisseria meningitidis | W | ST-11 complex | No value | UK | SV-2 | Opc- |
| 29997 | M01 240241 | Neisseria meningitidis | W | ST-11 complex | No value | UK | SV-2 | Opc- |
| 29998 | M01 240244 | Neisseria meningitidis | W | ST-11 complex | No value | UK | SV-2 | Opc- |
| 29999 | M01 240354 | Neisseria meningitidis | W | ST-11 complex | No value | UK | SV-2 | Opc- |
| 30000 | M01 240622 | Neisseria meningitidis | B | ST-11 complex | No value | UK | SV-2 | Opc- |
| 30001 | M01 240634 | Neisseria meningitidis | W | ST-11 complex | No value | UK | SV-2 | Opc- |
| 30002 | M01 240659 | Neisseria meningitidis | W | ST-11 complex | No value | UK | SV-2 | Opc- |
| 30003 | M01 240663 | Neisseria meningitidis | B | ST-11 complex | No value | UK | SV-2 | Opc- |
| 30004 | M01 240759 | Neisseria meningitidis | W | ST-11 complex | No value | UK | SV-2 | Opc- |
| 30005 | M01 240760 | Neisseria meningitidis | W | ST-11 complex | No value | UK | SV-2 | Opc- |
| 30006 | M01 240801 | Neisseria meningitidis | W | ST-11 complex | No value | UK | SV-2 | Opc- |
| 30007 | M01 240807 | Neisseria meningitidis | W | ST-11 complex | No value | UK | SV-2 | Opc- |
| 30008 | M01 240808 | Neisseria meningitidis | W | ST-11 complex | No value | UK | SV-2 | Opc- |
| 30009 | M01 240839 | Neisseria meningitidis | W | ST-11 complex | No value | UK | SV-2 | Opc- |
| 30010 | M01 240840 | Neisseria meningitidis | W | ST-11 complex | No value | UK | SV-2 | Opc- |
| 30011 | M01 240859 | Neisseria meningitidis | W | ST-11 complex | No value | UK | SV-2 | Opc- |
| 30012 | M01 240887 | Neisseria meningitidis | W | ST-11 complex | No value | UK | SV-2 | Opc- |
| 30013 | M01 240900 | Neisseria meningitidis | W | ST-11 complex | No value | UK | SV-2 | Opc- |
| 30014 | M01 240902 | Neisseria meningitidis | W | ST-11 complex | No value | UK | SV-2 | Opc- |
| 30015 | M01 240913 | Neisseria meningitidis | W | ST-11 complex | No value | UK | SV-2 | Opc- |
| 30016 | M01 240915 | Neisseria meningitidis | W | ST-11 complex | No value | UK | SV-2 | Opc- |
| 30017 | M01 240916 | Neisseria meningitidis | W | ST-11 complex | No value | UK | SV-2 | Opc- |

| | | | | | | | | |
|---|---|---|---|---|---|---|---|---|
| 30018 | M01 240921 | Neisseria meningitidis | W | ST-11 complex | No value | UK | SV-2 | Opc- |
| 30019 | M01 240949 | Neisseria meningitidis | W | ST-11 complex | No value | UK | SV-2 | Opc- |
| 30020 | M01 240953 | Neisseria meningitidis | W | ST-11 complex | No value | UK | SV-2 | Opc- |
| 30021 | M01 240956 | Neisseria meningitidis | W | ST-11 complex | No value | UK | SV-2 | Opc- |
| 30022 | M01 240959 | Neisseria meningitidis | W | ST-11 complex | No value | UK | SV-2 | Opc- |
| 30023 | M01 240967 | Neisseria meningitidis | W | ST-11 complex | No value | UK | SV-2 | Opc- |
| 30024 | M01 240978 | Neisseria meningitidis | W | ST-11 complex | No value | UK | SV-2 | Opc- |
| 30025 | M01 240979 | Neisseria meningitidis | W | ST-11 complex | No value | UK | SV-2 | Opc- |
| 30026 | M01 240989 | Neisseria meningitidis | W | ST-11 complex | No value | UK | SV-2 | Opc- |
| 30027 | M01 240990 | Neisseria meningitidis | W | ST-11 complex | No value | UK | SV-2 | Opc- |
| 30028 | M01 241031 | Neisseria meningitidis | W | ST-11 complex | No value | UK | SV-2 | Opc- |
| 30029 | M01 241052 | Neisseria meningitidis | W | ST-11 complex | No value | UK | SV-2 | Opc- |
| 30030 | M01 241064 | Neisseria meningitidis | W | ST-11 complex | No value | UK | SV-2 | Opc- |
| 30031 | M01 241093 | Neisseria meningitidis | W | ST-11 complex | No value | UK | SV-2 | Opc- |
| 30032 | M01 241097 | Neisseria meningitidis | W | ST-11 complex | No value | UK | SV-2 | Opc- |
| 30033 | M01 241098 | Neisseria meningitidis | W | ST-11 complex | No value | UK | SV-2 | Opc- |
| 30034 | M01 241133 | Neisseria meningitidis | W | ST-11 complex | No value | UK | SV-2 | Opc- |
| 30035 | M01 241213 | Neisseria meningitidis | W | ST-11 complex | No value | UK | SV-2 | Opc- |
| 30036 | M01 241214 | Neisseria meningitidis | W | ST-11 complex | No value | UK | SV-2 | Opc- |
| 30037 | M01 241368 | Neisseria meningitidis | W | ST-11 complex | No value | UK | SV-2 | Opc- |
| 30038 | M01 241396 | Neisseria meningitidis | W | ST-11 complex | No value | UK | SV-2 | Opc- |
| 30039 | M01 241454 | Neisseria meningitidis | W | ST-11 complex | No value | UK | SV-2 | Opc- |
| 30040 | M01 241494 | Neisseria meningitidis | W | ST-11 complex | No value | UK | SV-2 | Opc- |
| 30041 | M01 241950 | Neisseria meningitidis | W | ST-11 complex | No value | UK | SV-2 | Opc- |
| 30043 | M01 242084 | Neisseria meningitidis | W | ST-11 complex | No value | UK | SV-2 | Opc- |
| 30045 | M01 242572 | Neisseria meningitidis | B | ST-41/44 complex | No value | UK | SV-2 | Opc+ |
| 30046 | M02 240038 | Neisseria meningitidis | B | ST-11 complex | No value | UK | SV-2 | Opc- |
| 30047 | M02 240690 | Neisseria meningitidis | B | ST-11 complex | No value | UK | SV-2 | Opc- |
| 30048 | M02 241095 | Neisseria meningitidis | B | ST-11 complex | No value | UK | SV-2 | Opc- |
| 30051 | M03 241710 | Neisseria meningitidis | B | ST-11 complex | No value | UK | SV-2 | Opc- |
| 30057 | M08 240033 | Neisseria meningitidis | B | No value | No value | UK | SV-5 | Opc- |
| 30058 | M08 240227 | Neisseria meningitidis | B | ST-11 complex | No value | UK | SV-2 | Opc- |
| 30059 | M08 240819 | Neisseria meningitidis | B | ST-11 complex | No value | UK | SV-2 | Opc- |
| 30060 | M09 240026 | Neisseria meningitidis | B | ST-11 complex | No value | UK | SV-2 | Opc- |
| 30063 | M14 240065 | Neisseria meningitidis | W | ST-41/44 complex | No value | Turkey | SV-10 | Opc+ |
| 30064 | M14 240066 | Neisseria meningitidis | W | No value | No value | Turkey | SV-12 | Opc+ |
| 30065 | M14 240067 | Neisseria meningitidis | W | ST-11 complex | No value | Turkey | SV-2 | Opc- |
| 30066 | M14 240068 | Neisseria meningitidis | W | ST-11 complex | No value | Turkey | SV-2 | Opc- |
| 30067 | M14 240069 | Neisseria meningitidis | W | ST-11 complex | No value | Turkey | SV-2 | Opc- |
| 30068 | M14 240070 | Neisseria meningitidis | W | No value | No value | Turkey | SV-1 | Opc+ |
| 30069 | M12 240156 b | Neisseria meningitidis | W | ST-11 complex | No value | UK | SV-2 | Opc- |
| 30070 | M12 240160 b | Neisseria meningitidis | W | ST-11 complex | No value | UK | SV-2 | Opc- |
| 30071 | M12 240196 b | Neisseria meningitidis | W | ST-11 complex | No value | UK | SV-2 | Opc- |
| 30072 | M11 240099 b | Neisseria meningitidis | W | ST-11 complex | No value | UK | SV-2 | Opc- |
| 30073 | 96057 | Neisseria meningitidis | W | ST-11 complex | No value | Chad | SV-2 | Opc- |
| 30074 | 2001001 | Neisseria meningitidis | W | ST-11 complex | No value | Algeria | SV-2 | Opc- |
| 30075 | 2000081 | Neisseria meningitidis | W | ST-11 complex | No value | Senegal | SV-2 | Opc- |
| 30076 | 2000175 | Neisseria meningitidis | W | ST-11 complex | No value | Cameroon | SV-2 | Opc- |
| 30077 | 2000176 | Neisseria meningitidis | W | ST-11 complex | No value | Cameroon | SV-2 | Opc- |
| 30078 | 2002011 | Neisseria meningitidis | W | ST-11 complex | No value | Cameroon | SV-2 | Opc- |
| 30079 | 2000058 | Neisseria meningitidis | W | ST-11 complex | No value | Senegal | SV-2 | Opc- |
| 30080 | 2001076 | Neisseria meningitidis | W | ST-11 complex | No value | Algeria | SV-2 | Opc- |
| 30081 | 2002015 | Neisseria meningitidis | W | ST-11 complex | No value | Cameroon | SV-2 | Opc- |
| 30082 | 2002016 | Neisseria meningitidis | W | ST-11 complex | No value | Cameroon | SV-2 | Opc- |
| 30083 | 2002021 | Neisseria meningitidis | W | ST-11 complex | No value | Cameroon | SV-2 | Opc- |
| 30085 | 2002029 | Neisseria meningitidis | W | ST-11 complex | No value | Cameroon | SV-2 | Opc- |
| 30086 | 2001068 | Neisseria meningitidis | W | ST-11 complex | No value | Chad | SV-2 | Opc- |
| 30087 | 2002018 | Neisseria meningitidis | W | ST-11 complex | No value | Niger | SV-2 | Opc- |
| 30088 | 2002019 | Neisseria meningitidis | W | ST-11 complex | No value | Niger | SV-2 | Opc- |
| 30089 | 2002039 | Neisseria meningitidis | W | ST-11 complex | No value | Niger | SV-2 | Opc- |
| 30090 | 2002040 | Neisseria meningitidis | W | ST-11 complex | No value | Niger | SV-2 | Opc- |
| 30091 | 2002058 | Neisseria meningitidis | W | ST-11 complex | No value | Niger | SV-2 | Opc- |
| 30092 | 2002059 | Neisseria meningitidis | W | ST-11 complex | No value | Niger | SV-2 | Opc- |
| 30093 | 2003019 | Neisseria meningitidis | W | ST-11 complex | No value | Niger | SV-2 | Opc- |
| 30094 | 2003034 | Neisseria meningitidis | W | ST-11 complex | No value | Niger | SV-2 | Opc- |
| 30095 | 2003035 | Neisseria meningitidis | W | ST-11 complex | No value | Niger | SV-2 | Opc- |
| 30096 | 2003054 | Neisseria meningitidis | W | ST-11 complex | No value | Niger | SV-2 | Opc- |
| 30097 | 2003055 | Neisseria meningitidis | W | ST-11 complex | No value | Niger | SV-2 | Opc- |
| 30098 | 2004264 | Neisseria meningitidis | W | ST-11 complex | No value | Burkina Faso | SV-2 | Opc- |
| 30099 | 2004265 | Neisseria meningitidis | W | ST-11 complex | No value | Burkina Faso | SV-2 | Opc- |
| 30100 | 2004267 | Neisseria meningitidis | W | ST-11 complex | No value | Burkina Faso | SV-2 | Opc- |
| 30101 | 2004268 | Neisseria meningitidis | W | ST-11 complex | No value | Burkina Faso | SV-2 | Opc- |
| 30103 | 2001004 | Neisseria meningitidis | W | ST-22 complex | No value | Algeria | SV-2 | Opc+ |
| 30104 | 2001213 | Neisseria meningitidis | W | ST-11 complex | No value | Burkina Faso | SV-2 | Opc- |

| | | | | | | | | |
|---|---|---|---|---|---|---|---|---|
| 30105 | 2001214 | Neisseria meningitidis | W | ST-11 complex | No value | Burkina Faso | SV-2 | Opc- |
| 30106 | 2001215 | Neisseria meningitidis | W | ST-11 complex | No value | Burkina Faso | SV-2 | Opc- |
| 30107 | 2001069 | Neisseria meningitidis | W | ST-11 complex | No value | Senegal | SV-2 | Opc- |
| 30108 | M99 240089 | Neisseria meningitidis | B | ST-8 complex | No value | UK | SV-14 | Opc- |
| 30109 | M99 240400 | Neisseria meningitidis | B | No value | No value | UK | SV-11 | Opc- |
| 30110 | M99 240401 | Neisseria meningitidis | B | No value | No value | UK | SV-11 | Opc- |
| 30111 | M99 240487 | Neisseria meningitidis | B | No value | No value | UK | SV-11 | Opc- |
| 30112 | M99 240981 | Neisseria meningitidis | B | ST-41/44 complex | No value | UK | SV-2 | Opc+ |
| 30113 | M99 240982 | Neisseria meningitidis | B | ST-41/44 complex | No value | UK | SV-2 | Opc+ |
| 30114 | M99 240983 | Neisseria meningitidis | B | No value | No value | UK | SV-2 | Opc+ |
| 30115 | M99 242419 | Neisseria meningitidis | B | ST-41/44 complex | No value | UK | SV-6 | Opc+ |
| 30116 | M99 242434 | Neisseria meningitidis | B | ST-41/44 complex | No value | UK | SV-6 | Opc+ |
| 30117 | M99 242435 | Neisseria meningitidis | B | ST-41/44 complex | No value | UK | SV-6 | Opc+ |
| 30118 | M99 243362 | Neisseria meningitidis | B | ST-364 complex | No value | UK | SV-1 | Opc- |
| 30119 | M99 243841 | Neisseria meningitidis | B | ST-364 complex | No value | UK | SV-1 | Opc- |
| 30120 | M99 243964 | Neisseria meningitidis | B | ST-41/44 complex | No value | UK | SV-2 | Opc+ |
| 30121 | M99 243965 | Neisseria meningitidis | B | ST-41/44 complex | No value | UK | SV-2 | Opc+ |
| 30122 | M00 241434 | Neisseria meningitidis | W | ST-11 complex | No value | UK | SV-2 | Opc- |
| 30123 | M00 241979 | Neisseria meningitidis | W | ST-11 complex | No value | UK | SV-2 | Opc- |
| 30124 | M97 252162 | Neisseria meningitidis | W | ST-22 complex | No value | UK | SV-12 | Opc+ |
| 30125 | M97 252114 | Neisseria meningitidis | W | No value | No value | UK | SV-12 | Opc+ |
| 30126 | M05 240853 | Neisseria meningitidis | C | ST-11 complex | No value | Spain | SV-2 | Opc- |
| 30130 | M05 240857 | Neisseria meningitidis | C | ST-11 complex | No value | Spain | SV-2 | Opc- |
| 30131 | M05 240858 | Neisseria meningitidis | C | ST-11 complex | No value | Spain | SV-2 | Opc- |
| 30132 | M05 240859 | Neisseria meningitidis | C | ST-11 complex | No value | Spain | SV-2 | Opc- |
| 30134 | M05 240861 | Neisseria meningitidis | C | ST-11 complex | No value | Spain | SV-2 | Opc- |
| 30135 | M05 240121 | Neisseria meningitidis | C | No value | No value | Unknown | SV-7 | Opc- |
| 30136 | M13 240635 | Neisseria meningitidis | W | ST-11 complex | No value | UK | SV-2 | Opc- |
| 30137 | M13 240637 | Neisseria meningitidis | W | ST-11 complex | No value | UK | SV-2 | Opc- |
| 30138 | M13 240649 | Neisseria meningitidis | W | ST-11 complex | No value | UK | SV-2 | Opc- |
| 30139 | M13 240655 | Neisseria meningitidis | W | ST-11 complex | No value | UK | SV-2 | Opc- |
| 30140 | M13 240657 | Neisseria meningitidis | W | ST-11 complex | No value | UK | SV-2 | Opc- |
| 30141 | M13 240664 | Neisseria meningitidis | W | ST-11 complex | No value | UK | SV-2 | Opc- |
| 30142 | M13 240666 | Neisseria meningitidis | W | ST-11 complex | No value | UK | SV-2 | Opc- |
| 30143 | M13 240667 | Neisseria meningitidis | W | ST-11 complex | No value | UK | SV-2 | Opc- |
| 30144 | M13 240668 | Neisseria meningitidis | W | ST-11 complex | No value | UK | SV-2 | Opc- |
| 30145 | M13 240672 | Neisseria meningitidis | W | ST-11 complex | No value | UK | SV-2 | Opc- |
| 30146 | M13 240678 | Neisseria meningitidis | W | ST-11 complex | No value | UK | SV-2 | Opc- |
| 30147 | M13 240680 | Neisseria meningitidis | W | ST-11 complex | No value | UK | SV-2 | Opc- |
| 30148 | M13 240681 | Neisseria meningitidis | W | ST-11 complex | No value | UK | SV-2 | Opc- |
| 30150 | M13 240724 | Neisseria meningitidis | W | ST-11 complex | No value | UK | SV-2 | Opc- |
| 30151 | M13 240726 | Neisseria meningitidis | W | ST-11 complex | No value | UK | SV-2 | Opc- |
| 30152 | M13 240730 | Neisseria meningitidis | W | ST-11 complex | No value | UK | SV-2 | Opc- |
| 30153 | M13 240732 | Neisseria meningitidis | W | ST-11 complex | No value | UK | SV-2 | Opc- |
| 30154 | M14 240001 | Neisseria meningitidis | W | ST-11 complex | No value | UK | SV-2 | Opc- |
| 30155 | M14 240002 | Neisseria meningitidis | W | ST-11 complex | No value | UK | SV-2 | Opc- |
| 30156 | M14 240007 | Neisseria meningitidis | W | ST-11 complex | No value | UK | SV-2 | Opc- |
| 30157 | M14 240013 | Neisseria meningitidis | W | ST-11 complex | No value | UK | SV-2 | Opc- |
| 30158 | M14 240019 | Neisseria meningitidis | W | ST-11 complex | No value | UK | SV-2 | Opc- |
| 30159 | M14 240022 | Neisseria meningitidis | W | ST-11 complex | No value | UK | SV-2 | Opc- |
| 30160 | M14 240026 | Neisseria meningitidis | W | ST-11 complex | No value | UK | SV-2 | Opc- |
| 30161 | M14 240029 | Neisseria meningitidis | W | ST-11 complex | No value | UK | SV-2 | Opc- |
| 30162 | M14 240031 | Neisseria meningitidis | W | ST-11 complex | No value | UK | SV-2 | Opc- |
| 30163 | M14 240042 | Neisseria meningitidis | W | ST-11 complex | No value | UK | SV-2 | Opc- |
| 30164 | M14 240043 | Neisseria meningitidis | W | ST-11 complex | No value | UK | SV-2 | Opc- |
| 30165 | M14 240052 | Neisseria meningitidis | W | ST-11 complex | No value | UK | SV-2 | Opc- |
| 30166 | M14 240053 | Neisseria meningitidis | W | ST-11 complex | No value | UK | SV-2 | Opc- |
| 30167 | M14 240054 | Neisseria meningitidis | W | ST-11 complex | No value | UK | SV-2 | Opc- |
| 30168 | M14 240072 | Neisseria meningitidis | W | ST-11 complex | No value | UK | SV-2 | Opc- |
| 30169 | M14 240082 | Neisseria meningitidis | W | ST-11 complex | No value | UK | SV-2 | Opc- |
| 30170 | M14 240086 | Neisseria meningitidis | W | ST-11 complex | No value | UK | SV-2 | Opc- |
| 30171 | M98 252111 | Neisseria meningitidis | C | ST-11 complex | No value | UK | SV-2 | Opc- |
| 30172 | M02 240630 | Neisseria meningitidis | B | No value | No value | UK | SV-8 | Opc- |
| 30173 | NZ98/254 | Neisseria meningitidis | B | ST-41/44 complex | No value | New Zealand | SV-6 | Opc+ |
| 30174 | M97 250455 | Neisseria meningitidis | W | ST-11 complex | No value | UK | SV-2 | Opc- |
| 30175 | M97 250650 | Neisseria meningitidis | W | No value | No value | UK | SV-2 | Opc- |
| 30176 | M97 251635 | Neisseria meningitidis | W | ST-11 complex | No value | UK | SV-2 | Opc- |
| 30177 | M97 252137 | Neisseria meningitidis | W | No value | No value | UK | SV-1 | Opc+ |
| 30178 | M98 250031 | Neisseria meningitidis | W | ST-11 complex | No value | UK | SV-2 | Opc- |
| 30179 | M98 251039 | Neisseria meningitidis | W | ST-11 complex | No value | UK | SV-2 | Opc- |
| 30180 | M98 251534 | Neisseria meningitidis | W | ST-11 complex | No value | UK | SV-2 | Opc- |
| 30181 | M98 253527 | Neisseria meningitidis | W | ST-11 complex | No value | UK | SV-2 | Opc- |
| 30182 | M99 240896 | Neisseria meningitidis | W | ST-22 complex | No value | UK | SV-12 | Opc+ |
| 30183 | M99 242612 | Neisseria meningitidis | W | ST-11 complex | No value | Malta | SV-2 | Opc- |

| | | | | | | | | |
|---|---|---|---|---|---|---|---|---|
| 30184 | M99 243856 | Neisseria meningitidis | W | ST-11 complex | No value | Malta | SV-2 | Opc- |
| 30185 | M96 255770 | Neisseria meningitidis | C | ST-11 complex | No value | UK | SV-2 | Opc- |
| 30188 | M96 255774 | Neisseria meningitidis | C | ST-11 complex | No value | UK | SV-2 | Opc- |
| 30190 | M96 255863 | Neisseria meningitidis | C | ST-11 complex | No value | UK | SV-2 | Opc- |
| 30191 | M96 255864 | Neisseria meningitidis | C | ST-11 complex | No value | UK | SV-2 | Opc- |
| 30192 | M96 255871 | Neisseria meningitidis | C | ST-11 complex | No value | UK | SV-2 | Opc- |
| 30193 | M96 255976 | Neisseria meningitidis | C | ST-11 complex | No value | UK | SV-2 | Opc- |
| 30196 | M99 240595 | Neisseria meningitidis | C | No value | No value | UK | SV-12 | Opc+ |
| 30202 | M99 240747 | Neisseria meningitidis | C | ST-11 complex | No value | UK | SV-2 | Opc- |
| 30206 | M99 240772 | Neisseria meningitidis | C | ST-11 complex | No value | UK | SV-2 | Opc- |
| 30210 | M13 240691 | Neisseria meningitidis | C | ST-11 complex | No value | UK | SV-2 | Opc- |
| 30211 | M13 240735 | Neisseria meningitidis | C | ST-23 complex | No value | UK | SV-11 | Opc+ |
| 30214 | M14 240100 | Neisseria meningitidis | C | ST-11 complex | No value | UK | SV-2 | Opc- |
| 30215 | M14 240107 | Neisseria meningitidis | C | ST-11 complex | No value | UK | SV-2 | Opc- |
| 30216 | 76067 | Neisseria meningitidis | W | ST-174 complex | No value | France | SV-1 | Opc+ |
| 30218 | 78693 | Neisseria meningitidis | W | ST-8 complex | No value | France | SV-10 | Opc- |
| 30219 | 78786 | Neisseria meningitidis | W | ST-8 complex | No value | France | SV-10 | Opc- |
| 30222 | 86276 | Neisseria meningitidis | W | ST-22 complex | No value | France | SV-12 | Opc+ |
| 30223 | C M-137 | Neisseria meningitidis | C | No value | No value | Unknown | SV-7 | Opc- |
| 30224 | M03 240641 | Neisseria meningitidis | C | No value | No value | UK | SV-2 | Opc- |
| 30225 | M03 240792 | Neisseria meningitidis | C | ST-269 complex | No value | UK | SV-1 | Opc+ |
| 30226 | M03 241190 | Neisseria meningitidis | C | ST-11 complex | No value | UK | SV-2 | Opc- |
| 30228 | M03 241594 | Neisseria meningitidis | C | ST-269 complex | No value | UK | SV-1 | Opc+ |
| 30231 | M04 241279 | Neisseria meningitidis | C | ST-269 complex | No value | UK | SV-1 | Opc+ |
| 30232 | M96 252550 | Neisseria meningitidis | C | ST-11 complex | No value | UK | SV-2 | Opc- |
| 30234 | M96 252316 | Neisseria meningitidis | W | ST-11 complex | No value | UK | SV-2 | Opc- |
| 30235 | M96 253521 | Neisseria meningitidis | W | ST-11 complex | No value | UK | SV-2 | Opc- |
| 30236 | M96 255203 | Neisseria meningitidis | W | ST-11 complex | No value | UK | SV-2 | Opc- |
| 30237 | M96 255688 | Neisseria meningitidis | W | ST-11 complex | No value | UK | SV-2 | Opc- |
| 30238 | M96 256246 | Neisseria meningitidis | W | ST-11 complex | No value | UK | SV-2 | Opc- |
| 30239 | F85 0000001 | Neisseria meningitidis | C | ST-11 complex | No value | UK | SV-2 | Opc- |
| 30240 | F85 0000045 | Neisseria meningitidis | C | ST-11 complex | No value | UK | SV-2 | Opc- |
| 30241 | F85 0000076 | Neisseria meningitidis | C | ST-11 complex | No value | UK | SV-2 | Opc- |
| 30242 | F85 0000171 | Neisseria meningitidis | C | ST-11 complex | No value | UK | SV-2 | Opc- |
| 30243 | F85 0000671 | Neisseria meningitidis | C | ST-11 complex | No value | UK | SV-2 | Opc- |
| 30244 | F85 0000704 | Neisseria meningitidis | C | ST-11 complex | No value | UK | SV-2 | Opc- |
| 30245 | F85 0000746 | Neisseria meningitidis | C | ST-11 complex | No value | UK | SV-2 | Opc- |
| 30247 | E84 000967 | Neisseria meningitidis | W | ST-22 complex | No value | UK | SV-12 | Opc+ |
| 30248 | F85 000311 | Neisseria meningitidis | W | No value | No value | UK | SV-1 | Opc+ |
| 30249 | F85 001122 | Neisseria meningitidis | W | ST-174 complex | No value | UK | SV-1 | Opc+ |
| 30250 | G86 000031 | Neisseria meningitidis | W | ST-22 complex | No value | UK | SV-12 | Opc+ |
| 30251 | H87 000098 | Neisseria meningitidis | W | ST-11 complex | No value | UK | SV-2 | Opc- |
| 30252 | H87 002642 | Neisseria meningitidis | W | ST-22 complex | No value | UK | SV-12 | Opc+ |
| 30253 | K89 000575 | Neisseria meningitidis | W | ST-11 complex | No value | UK | SV-2 | Opc- |
| 30255 | L90 002191 | Neisseria meningitidis | W | ST-11 complex | No value | UK | SV-2 | Opc- |
| 30256 | L91 000268 | Neisseria meningitidis | W | ST-22 complex | No value | UK | SV-12 | Opc+ |
| 30257 | M02 242016 | Neisseria meningitidis | B | ST-11 complex | No value | UK | SV-2 | Opc- |
| 30259 | M02 242030 | Neisseria meningitidis | C | ST-11 complex | No value | UK | SV-2 | Opc- |
| 30260 | M02 242007 | Neisseria meningitidis | C | ST-11 complex | No value | UK | SV-2 | Opc- |
| 30261 | M02 242014 | Neisseria meningitidis | B | ST-11 complex | No value | UK | SV-2 | Opc- |
| 30263 | M02 242035 | Neisseria meningitidis | C | ST-11 complex | No value | UK | SV-2 | Opc- |
| 30264 | M02 242062 | Neisseria meningitidis | C | ST-11 complex | No value | UK | SV-2 | Opc- |
| 30265 | M01 242103 | Neisseria meningitidis | W | ST-11 complex | No value | UK | SV-2 | Opc- |
| 30266 | M01 242502 | Neisseria meningitidis | W | ST-11 complex | No value | UK | SV-2 | Opc- |
| 30267 | M01 242503 | Neisseria meningitidis | W | ST-11 complex | No value | UK | SV-2 | Opc- |
| 30268 | M05 240852 | Neisseria meningitidis | C | No value | No value | Germany | SV-2 | Opc+ |
| 30269 | M99 243594 | Neisseria meningitidis | B | ST-5 complex | No value | UK | SV-5 | Opc+ |
| 30270 | M01 240070 | Neisseria meningitidis | W | ST-22 complex | No value | UK | SV-12 | Opc+ |
| 30272 | M09 240651 | Neisseria meningitidis | A | ST-1 complex | No value | Unknown | SV-5 | Opc+ |
| 30273 | M01 240101 | Neisseria meningitidis | B | ST-269 complex | No value | UK | SV-1 | Opc+ |
| 30276 | M01 240364 | Neisseria meningitidis | B | ST-11 complex | No value | UK | SV-2 | Opc- |
| 30277 | M00 242922 | Neisseria meningitidis | B | ST-41/44 complex | No value | UK | SV-2 | Opc+ |
| 30279 | 2012 107 | Neisseria meningitidis | C | ST-41/44 complex | No value | Portugal | SV-10 | Opc+ |
| 30282 | 2012 253 | Neisseria meningitidis | C | No value | No value | Portugal | SV-12 | Opc- |
| 30283 | 2012 493 | Neisseria meningitidis | W | ST-22 complex | No value | Portugal | SV-12 | Opc+ |
| 30284 | M14 240181 | Neisseria meningitidis | C | ST-11 complex | No value | Canada | SV-2 | Opc- |
| 30286 | M14 240183 | Neisseria meningitidis | C | ST-11 complex | No value | Canada | SV-2 | Opc- |
| 30287 | M14 240184 | Neisseria meningitidis | C | ST-11 complex | No value | Canada | SV-2 | Opc- |
| 30288 | M14 240185 | Neisseria meningitidis | C | ST-11 complex | No value | Canada | SV-2 | Opc- |
| 30290 | M14 240187 | Neisseria meningitidis | C | ST-11 complex | No value | Canada | SV-2 | Opc- |
| 30291 | M14 240188 | Neisseria meningitidis | C | ST-11 complex | No value | Canada | SV-2 | Opc- |
| 30294 | M14 240191 | Neisseria meningitidis | C | ST-11 complex | No value | Canada | SV-2 | Opc- |
| 30295 | M14 240192 | Neisseria meningitidis | C | ST-11 complex | No value | Canada | SV-2 | Opc- |
| 30296 | M14 240193 | Neisseria meningitidis | C | ST-11 complex | No value | Canada | SV-2 | Opc- |

| | | | | | | | | |
|---|---|---|---|---|---|---|---|---|
| 30297 | M14 240194 | Neisseria meningitidis | C | ST-11 complex | No value | Canada | SV-2 | Opc- |
| 30298 | M14 240195 | Neisseria meningitidis | C | ST-11 complex | No value | Canada | SV-2 | Opc- |
| 30299 | M14 240196 | Neisseria meningitidis | C | ST-11 complex | No value | Canada | SV-2 | Opc- |
| 30300 | M14 240197 | Neisseria meningitidis | C | ST-11 complex | No value | Canada | SV-2 | Opc- |
| 30301 | M14 240198 | Neisseria meningitidis | C | ST-11 complex | No value | Canada | SV-2 | Opc- |
| 30302 | OVG_12 | Neisseria meningitidis | NG | No value | No value | Unknown | SV-5 | Opc+ |
| 30303 | OVG_MenP1_1 | Neisseria meningitidis | NG | ST-32 complex | No value | Unknown | SV-1 | Opc+ |
| 30304 | OVG_14 | Neisseria meningitidis | NG | No value | No value | Unknown | SV-5 | Opc+ |
| 30305 | OVG_11 | Neisseria meningitidis | NG | ST-181 complex | No value | Unknown | SV-5 | Opc+ |
| 30306 | OVG_7 | Neisseria meningitidis | NG | ST-11 complex | No value | Unknown | SV-2 | Opc- |
| 30307 | NM8250 | Neisseria meningitidis | B | ST-41/44 complex | No value | UK | SV-6 | Opc+ |
| 30308 | NM8307 | Neisseria meningitidis | NG | No value | No value | UK | SV-2 | Opc- |
| 30310 | NM8468 | Neisseria meningitidis | NG | ST-269 complex | No value | UK | SV-8 | Opc+ |
| 30311 | NM8525 | Neisseria meningitidis | NG | ST-269 complex | No value | UK | SV-1 | Opc+ |
| 30312 | NM8558 | Neisseria meningitidis | NG | No value | No value | UK | SV-3 | Opc+ |
| 30313 | NM8572 | Neisseria meningitidis | NG | No value | No value | UK | SV-1 | Opc+ |
| 30314 | NM8583 | Neisseria meningitidis | NG | ST-269 complex | No value | UK | SV-8 | Opc+ |
| 30315 | NM8602 | Neisseria meningitidis | NG | ST-162 complex | No value | UK | SV-1 | Opc+ |
| 30316 | NM8633 | Neisseria meningitidis | NG | ST-11 complex | No value | UK | SV-2 | Opc- |
| 30317 | NM8652 | Neisseria meningitidis | NG | ST-4821 complex | No value | UK | SV-19 | Opc+ |
| 30318 | NM8674 | Neisseria meningitidis | NG | ST-1136 complex | No value | UK | SV-2 | Opc+ |
| 30319 | NM8726 | Neisseria meningitidis | NG | ST-269 complex | No value | UK | SV-8 | Opc+ |
| 30320 | NM8736 | Neisseria meningitidis | NG | ST-41/44 complex | No value | UK | SV-10 | Opc+ |
| 30321 | NM9062 | Neisseria meningitidis | B | ST-41/44 complex | No value | UK | SV-6 | Opc+ |
| 30322 | NM9071 | Neisseria meningitidis | NG | No value | No value | UK | SV-1 | Opc- |
| 30323 | NM9124 | Neisseria meningitidis | NG | No value | No value | UK | SV-5 | Opc+ |
| 30325 | NM9565 | Neisseria meningitidis | B | ST-4821 complex | No value | UK | SV-18 | Opc+ |
| 30326 | NM9658 | Neisseria meningitidis | NG | ST-4821 complex | No value | UK | SV-18 | Opc+ |
| 30327 | NM9853 | Neisseria meningitidis | NG | ST-41/44 complex | No value | UK | SV-6 | Opc+ |
| 30328 | NM9905 | Neisseria meningitidis | B | No value | No value | UK | SV-1 | Opc- |
| 30329 | NM9954 | Neisseria meningitidis | C | ST-11 complex | No value | UK | SV-2 | Opc- |
| 30330 | NM10052 | Neisseria meningitidis | B | ST-269 complex | No value | UK | SV-8 | Opc+ |
| 30331 | NM10053 | Neisseria meningitidis | B | ST-269 complex | No value | UK | SV-8 | Opc+ |
| 30332 | NM10313 | Neisseria meningitidis | Y | ST-23 complex | No value | UK | SV-11 | Opc+ |
| 30333 | NM10364 | Neisseria meningitidis | NG | ST-4821 complex | No value | UK | SV-20 | Opc+ |
| 30334 | NM10421 | Neisseria meningitidis | NG | ST-1157 complex | No value | UK | SV-1 | Opc+ |
| 30335 | NM10492 | Neisseria meningitidis | NG | No value | No value | UK | SV-5 | Opc+ |
| 30336 | NM10762 | Neisseria meningitidis | NG | ST-23 complex | No value | UK | SV-11 | Opc+ |
| 30337 | NM10763 | Neisseria meningitidis | NG | No value | No value | UK | SV-5 | Opc+ |
| 30338 | NM10833 | Neisseria meningitidis | NG | ST-41/44 complex | No value | UK | SV-6 | Opc+ |
| 30339 | NM10853 | Neisseria meningitidis | NG | ST-53 complex | No value | UK | SV-2 | Opc- |
| 30340 | NM10863 | Neisseria meningitidis | B | ST-41/44 complex | No value | UK | SV-5 | Opc+ |
| 30341 | NM10864 | Neisseria meningitidis | B | ST-41/44 complex | No value | UK | SV-2 | Opc+ |
| 30342 | NM10989 | Neisseria meningitidis | NG | ST-1157 complex | No value | UK | SV-1 | Opc+ |
| 30343 | NM11067 | Neisseria meningitidis | B | ST-41/44 complex | No value | UK | SV-5 | Opc- |
| 30344 | NM11099 | Neisseria meningitidis | B | ST-213 complex | No value | UK | SV-2 | Opc- |
| 30457 | OVG_MenP1_1 | Neisseria meningitidis | NG | ST-32 complex | No value | Unknown | SV-1 | Opc+ |
| 30466 | NMA510612 | Neisseria meningitidis | A | ST-5 complex | Sporadic case | China | SV-5 | Opc+ |
| 30680 | 12001-10 | Neisseria meningitidis | B | ST-269 complex | No value | Ireland | SV-1 | Opc+ |
| 30681 | 12001-14 | Neisseria meningitidis | B | No value | No value | Ireland | SV-7 | Opc+ |
| 30682 | 12002-10 | Neisseria meningitidis | B | ST-269 complex | No value | Ireland | SV-1 | Opc+ |
| 30683 | 12003-10 | Neisseria meningitidis | B | ST-269 complex | No value | Ireland | SV-1 | Opc+ |
| 30684 | 12003-14 | Neisseria meningitidis | B | ST-41/44 complex | No value | Ireland | SV-2 | Opc+ |
| 30685 | 12004-10 | Neisseria meningitidis | B | ST-269 complex | No value | Ireland | SV-1 | Opc+ |
| 30686 | 12004-14 | Neisseria meningitidis | B | ST-41/44 complex | No value | Ireland | SV-10 | Opc+ |
| 30687 | 12005-10 | Neisseria meningitidis | B | ST-41/44 complex | No value | Ireland | SV-2 | Opc+ |
| 30688 | 12005-14 | Neisseria meningitidis | B | ST-41/44 complex | No value | Ireland | SV-2 | Opc+ |
| 30689 | 12006-14 | Neisseria meningitidis | B | ST-41/44 complex | No value | Ireland | SV-2 | Opc+ |
| 30690 | 12007-10 | Neisseria meningitidis | B | ST-32 complex | No value | Ireland | SV-1 | Opc+ |
| 30691 | 12007-14 | Neisseria meningitidis | B | ST-41/44 complex | No value | Ireland | SV-2 | Opc+ |
| 30692 | 12008-14 | Neisseria meningitidis | B | ST-41/44 complex | No value | Ireland | SV-6 | Opc+ |
| 30693 | 12009-10 | Neisseria meningitidis | B | ST-41/44 complex | No value | Ireland | SV-6 | Opc+ |
| 30694 | 12009-14 | Neisseria meningitidis | B | ST-11 complex | No value | Ireland | SV-2 | Opc- |
| 30696 | 12011-09 | Neisseria meningitidis | B | ST-41/44 complex | No value | Ireland | SV-2 | Opc+ |
| 30697 | 12011-10 | Neisseria meningitidis | B | ST-461 complex | No value | Ireland | SV-5 | Opc- |
| 30698 | 12011-14 | Neisseria meningitidis | NG | ST-11 complex | No value | Ireland | SV-2 | Opc- |
| 30699 | 12012-10 | Neisseria meningitidis | B | ST-162 complex | No value | Ireland | SV-1 | Opc+ |
| 30700 | 12013-14 | Neisseria meningitidis | NG | ST-32 complex | No value | Ireland | SV-1 | Opc+ |
| 30701 | 12015-10 | Neisseria meningitidis | B | ST-41/44 complex | No value | Ireland | SV-2 | Opc+ |
| 30702 | 12016-10 | Neisseria meningitidis | B | ST-254 complex | No value | Ireland | SV-1 | Opc+ |
| 30703 | 12017-10 | Neisseria meningitidis | B | ST-269 complex | No value | Ireland | SV-8 | Opc+ |
| 30704 | 12018-10 | Neisseria meningitidis | B | ST-269 complex | No value | Ireland | SV-8 | Opc+ |
| 30706 | 12020-10 | Neisseria meningitidis | B | ST-41/44 complex | No value | Ireland | SV-2 | Opc+ |
| 30707 | 12021-10 | Neisseria meningitidis | B | ST-41/44 complex | No value | Ireland | SV-2 | Opc+ |

| | | | | | | | | |
|---|---|---|---|---|---|---|---|---|
| 30708 | 12021-13 | Neisseria meningitidis | B | ST-461 complex | No value | Ireland | SV-1 | Opc- |
| 30709 | 12022-10 | Neisseria meningitidis | B | ST-41/44 complex | No value | Ireland | SV-2 | Opc+ |
| 30710 | 12022-13 | Neisseria meningitidis | B | ST-269 complex | No value | Ireland | SV-8 | Opc+ |
| 30711 | 12023-10 | Neisseria meningitidis | B | ST-269 complex | No value | Ireland | SV-8 | Opc+ |
| 30712 | 12024-13 | Neisseria meningitidis | B | No value | No value | Ireland | SV-12 | Opc+ |
| 30713 | 12025-13 | Neisseria meningitidis | B | ST-32 complex | No value | Ireland | SV-1 | Opc+ |
| 30714 | 12027-13 | Neisseria meningitidis | B | ST-41/44 complex | No value | Ireland | SV-10 | Opc+ |
| 30715 | 12028-13 | Neisseria meningitidis | B | ST-213 complex | No value | Ireland | SV-12 | Opc- |
| 30716 | 12029-13 | Neisseria meningitidis | B | ST-41/44 complex | No value | Ireland | SV-2 | Opc+ |
| 30718 | 12031-13 | Neisseria meningitidis | B | ST-60 complex | No value | Ireland | SV-11 | Opc+ |
| 30719 | 12032-13 | Neisseria meningitidis | B | ST-41/44 complex | No value | Ireland | SV-2 | Opc+ |
| 30720 | 12033-13 | Neisseria meningitidis | B | ST-41/44 complex | No value | Ireland | SV-2 | Opc+ |
| 30721 | 12034-13 | Neisseria meningitidis | B | ST-213 complex | No value | Ireland | SV-12 | Opc- |
| 30722 | 12043-13 | Neisseria meningitidis | B | ST-41/44 complex | No value | Ireland | SV-2 | Opc+ |
| 30723 | 12036-13 | Neisseria meningitidis | B | ST-269 complex | No value | Ireland | SV-1 | Opc+ |
| 30724 | 12037-09 | Neisseria meningitidis | B | ST-269 complex | No value | Ireland | SV-1 | Opc+ |
| 30725 | 12037-13 | Neisseria meningitidis | C | No value | No value | Ireland | SV-8 | Opc+ |
| 30726 | 12038-09 | Neisseria meningitidis | B | ST-162 complex | No value | Ireland | SV-1 | Opc+ |
| 30727 | 12038-13 | Neisseria meningitidis | B | ST-11 complex | No value | Ireland | SV-2 | Opc- |
| 30728 | 12039-09 | Neisseria meningitidis | B | ST-41/44 complex | No value | Ireland | SV-2 | Opc+ |
| 30729 | 12039-13 | Neisseria meningitidis | B | ST-213 complex | No value | Ireland | SV-12 | Opc- |
| 30730 | 12040-09 | Neisseria meningitidis | B | ST-41/44 complex | No value | Ireland | SV-2 | Opc+ |
| 30731 | 12040-13 | Neisseria meningitidis | B | ST-162 complex | No value | Ireland | SV-1 | Opc+ |
| 30732 | 12041-09 | Neisseria meningitidis | B | ST-41/44 complex | No value | Ireland | SV-2 | Opc+ |
| 30733 | 12041-13 | Neisseria meningitidis | B | ST-162 complex | No value | Ireland | SV-1 | Opc+ |
| 30734 | 12042-13 | Neisseria meningitidis | B | ST-35 complex | No value | Ireland | SV-12 | Opc+ |
| 30735 | 12043-09 | Neisseria meningitidis | B | ST-269 complex | No value | Ireland | SV-11 | Opc+ |
| 30736 | 12035-13 | Neisseria meningitidis | B | ST-32 complex | No value | Ireland | SV-1 | Opc+ |
| 30737 | 12044-09 | Neisseria meningitidis | B | ST-41/44 complex | No value | Ireland | SV-2 | Opc+ |
| 30738 | 12044-13 | Neisseria meningitidis | B | ST-41/44 complex | No value | Ireland | SV-2 | Opc+ |
| 30739 | 12045-09 | Neisseria meningitidis | B | ST-41/44 complex | No value | Ireland | SV-2 | Opc+ |
| 30740 | 12045-13 | Neisseria meningitidis | E | ST-1157 complex | No value | Ireland | SV-1 | Opc+ |
| 30741 | 12046-09 | Neisseria meningitidis | B | ST-41/44 complex | No value | Ireland | SV-6 | Opc+ |
| 30742 | 12046-13 | Neisseria meningitidis | B | ST-269 complex | No value | Ireland | SV-1 | Opc+ |
| 30743 | 12047-13 | Neisseria meningitidis | B | ST-41/44 complex | No value | Ireland | SV-2 | Opc+ |
| 30745 | 12050-09 | Neisseria meningitidis | B | ST-18 complex | No value | Ireland | SV-7 | Opc- |
| 30746 | 12051-09 | Neisseria meningitidis | B | ST-41/44 complex | No value | Ireland | SV-2 | Opc+ |
| 30748 | 12054-09 | Neisseria meningitidis | B | ST-162 complex | No value | Ireland | SV-1 | Opc+ |
| 30749 | 12054-13 | Neisseria meningitidis | B | ST-162 complex | No value | Ireland | SV-1 | Opc+ |
| 30750 | 12055-13 | Neisseria meningitidis | B | ST-213 complex | No value | Ireland | SV-12 | Opc- |
| 30751 | 12056-09 | Neisseria meningitidis | B | ST-269 complex | No value | Ireland | SV-1 | Opc+ |
| 30753 | 12057-09 | Neisseria meningitidis | B | ST-213 complex | No value | Ireland | SV-11 | Opc- |
| 30754 | 12059-09 | Neisseria meningitidis | B | ST-32 complex | No value | Ireland | SV-1 | Opc+ |
| 30755 | 12060-09 | Neisseria meningitidis | B | No value | No value | Ireland | SV-12 | Opc- |
| 30756 | 12087-06 | Neisseria meningitidis | B | ST-41/44 complex | No value | Ireland | SV-2 | Opc+ |
| 30757 | 3193-99 | Neisseria meningitidis | B | ST-41/44 complex | No value | Ireland | SV-2 | Opc+ |
| 30758 | M1.1 | Neisseria meningitidis | B | ST-41/44 complex | No value | Ireland | SV-2 | Opc+ |
| 30759 | M13.1 | Neisseria meningitidis | B | ST-41/44 complex | No value | Ireland | SV-2 | Opc+ |
| 30760 | M14.1 | Neisseria meningitidis | B | ST-461 complex | No value | Ireland | SV-1 | Opc- |
| 30761 | M16.1 | Neisseria meningitidis | B | No value | No value | Ireland | SV-8 | Opc+ |
| 30762 | M2.1 | Neisseria meningitidis | B | ST-41/44 complex | No value | Ireland | SV-2 | Opc+ |
| 30763 | M20.1 | Neisseria meningitidis | B | ST-269 complex | No value | Ireland | SV-8 | Opc+ |
| 30764 | M30.1 | Neisseria meningitidis | W | ST-22 complex | No value | Ireland | SV-12 | Opc+ |
| 30765 | M31.1 | Neisseria meningitidis | B | ST-41/44 complex | No value | Ireland | SV-2 | Opc+ |
| 30766 | M32.1 | Neisseria meningitidis | B | No value | No value | Ireland | SV-8 | Opc+ |
| 30767 | M34.1 | Neisseria meningitidis | B | ST-41/44 complex | No value | Ireland | SV-2 | Opc+ |
| 30768 | M35.1 | Neisseria meningitidis | B | ST-41/44 complex | No value | Ireland | SV-2 | Opc+ |
| 30769 | M42.1 | Neisseria meningitidis | NG | ST-1157 complex | No value | Ireland | SV-1 | Opc+ |
| 30772 | M46.3 | Neisseria meningitidis | B | ST-41/44 complex | No value | Ireland | SV-2 | Opc+ |
| 31148 | M14 240411 | Neisseria meningitidis | W | ST-11 complex | No value | Unknown | SV-2 | Opc- |
| 31149 | M14 240412 | Neisseria meningitidis | W | ST-11 complex | No value | Unknown | SV-2 | Opc- |
| 31150 | M14 240413 | Neisseria meningitidis | W | ST-11 complex | No value | Unknown | SV-2 | Opc- |
| 31151 | M14 240414 | Neisseria meningitidis | W | ST-11 complex | No value | Unknown | SV-2 | Opc- |
| 31152 | M14 240415 | Neisseria meningitidis | W | ST-11 complex | No value | Unknown | SV-2 | Opc- |
| 31155 | M14 240416 | Neisseria meningitidis | W | ST-11 complex | No value | Unknown | SV-2 | Opc- |
| 31156 | M14 240417 | Neisseria meningitidis | W | ST-11 complex | No value | Unknown | SV-2 | Opc- |
| 31157 | M14 240418 | Neisseria meningitidis | W | ST-11 complex | No value | Unknown | SV-2 | Opc- |
| 31158 | M14 240419 | Neisseria meningitidis | W | ST-11 complex | No value | Unknown | SV-2 | Opc- |
| 31159 | M14 240420 | Neisseria meningitidis | W | ST-11 complex | No value | Unknown | SV-2 | Opc- |
| 31160 | M14 240421 | Neisseria meningitidis | W | No value | No value | Unknown | SV-2 | Opc- |
| 31161 | M14 240422 | Neisseria meningitidis | W | ST-11 complex | No value | Unknown | SV-2 | Opc- |
| 31162 | M14 240329 | Neisseria meningitidis | B | ST-11 complex | No value | UK | SV-2 | Opc- |
| 31163 | M14 240380 | Neisseria meningitidis | B | ST-41/44 complex | No value | UK | SV-2 | Opc+ |
| 31164 | M14 240443 | Neisseria meningitidis | W | ST-11 complex | No value | France | SV-2 | Opc- |

| | | | | | | | | |
|---|---|---|---|---|---|---|---|---|
| 31165 | M14 240444 | Neisseria meningitidis | W | ST-11 complex | No value | France | SV-2 | Opc- |
| 31166 | M14 240445 | Neisseria meningitidis | W | ST-11 complex | No value | France | SV-2 | Opc- |
| 31167 | M14 240446 | Neisseria meningitidis | W | ST-11 complex | No value | France | SV-2 | Opc- |
| 31168 | M14 240447 | Neisseria meningitidis | W | ST-11 complex | No value | UK | SV-2 | Opc- |
| 31169 | M14 240448 | Neisseria meningitidis | W | ST-11 complex | No value | UK | SV-2 | Opc- |
| 31170 | M14 240449 | Neisseria meningitidis | B | ST-41/44 complex | No value | Ireland | SV-6 | Opc+ |
| 31177 | NM8307 | Neisseria meningitidis | NG | No value | MSM | UK | SV-2 | Opc- |
| 31179 | NM8468 | Neisseria meningitidis | NG | ST-269 complex | MSM | UK | SV-8 | Opc+ |
| 31180 | NM8525 | Neisseria meningitidis | NG | ST-269 complex | MSM | UK | SV-1 | Opc+ |
| 31181 | NM8558 | Neisseria meningitidis | NG | No value | MSM | UK | SV-3 | Opc+ |
| 31182 | NM8572 | Neisseria meningitidis | NG | No value | MSM | UK | SV-1 | Opc+ |
| 31183 | NM8583 | Neisseria meningitidis | NG | ST-269 complex | MSM | UK | SV-8 | Opc+ |
| 31184 | NM8602 | Neisseria meningitidis | NG | ST-162 complex | MSM | UK | SV-1 | Opc+ |
| 31185 | NM8633 | Neisseria meningitidis | NG | ST-11 complex | MSM | UK | SV-2 | Opc- |
| 31186 | NM8652 | Neisseria meningitidis | NG | ST-4821 complex | MSM | UK | SV-19 | Opc+ |
| 31187 | NM8674 | Neisseria meningitidis | NG | ST-1136 complex | MSM | UK | SV-2 | Opc+ |
| 31188 | NM8726 | Neisseria meningitidis | NG | ST-269 complex | MSM | UK | SV-8 | Opc+ |
| 31189 | NM8736 | Neisseria meningitidis | NG | ST-41/44 complex | MSM | UK | SV-10 | Opc+ |
| 31191 | NM9071 | Neisseria meningitidis | NG | No value | MSM | UK | SV-1 | Opc- |
| 31192 | NM9124 | Neisseria meningitidis | NG | No value | MSM | UK | SV-5 | Opc+ |
| 31194 | NM9565 | Neisseria meningitidis | B | ST-4821 complex | No value | UK | SV-14 | Opc+ |
| 31195 | NM9658 | Neisseria meningitidis | NG | ST-4821 complex | MSM | UK | SV-14 | Opc+ |
| 31197 | NM9905 | Neisseria meningitidis | B | No value | No value | UK | SV-1 | Opc- |
| 31198 | NM9954 | Neisseria meningitidis | C | ST-11 complex | No value | UK | SV-2 | Opc- |
| 31199 | NM10052 | Neisseria meningitidis | B | ST-269 complex | No value | UK | SV-8 | Opc+ |
| 31200 | NM10053 | Neisseria meningitidis | B | ST-269 complex | No value | UK | SV-8 | Opc+ |
| 31201 | NM10313 | Neisseria meningitidis | Y | ST-23 complex | No value | UK | SV-11 | Opc+ |
| 31202 | NM10364 | Neisseria meningitidis | NG | ST-4821 complex | MSM | UK | SV-20 | Opc+ |
| 31203 | NM10421 | Neisseria meningitidis | NG | ST-1157 complex | MSM | UK | SV-1 | Opc+ |
| 31204 | NM10492 | Neisseria meningitidis | NG | No value | MSM | UK | SV-5 | Opc+ |
| 31205 | NM10762 | Neisseria meningitidis | NG | ST-23 complex | MSM | UK | SV-11 | Opc+ |
| 31206 | NM10763 | Neisseria meningitidis | NG | No value | MSM | UK | SV-5 | Opc+ |
| 31208 | NM10853 | Neisseria meningitidis | NG | ST-53 complex | No value | UK | SV-2 | Opc- |
| 31209 | NM10863 | Neisseria meningitidis | B | ST-41/44 complex | No value | UK | SV-5 | Opc+ |
| 31210 | NM10864 | Neisseria meningitidis | B | ST-41/44 complex | No value | UK | SV-2 | Opc+ |
| 31211 | NM10989 | Neisseria meningitidis | NG | ST-1157 complex | MSM | UK | SV-1 | Opc+ |
| 31212 | NM11067 | Neisseria meningitidis | B | ST-41/44 complex | No value | UK | SV-5 | Opc- |
| 31213 | NM11099 | Neisseria meningitidis | B | ST-213 complex | No value | UK | SV-12 | Opc- |
| 31214 | LIM707 | Neisseria meningitidis | C | ST-11 complex | No value | France | SV-2 | Opc- |
| 31215 | LIM534 | Neisseria meningitidis | A | ST-5 complex | No value | France | SV-5 | Opc+ |
| 31310 | NM174 | Neisseria meningitidis | NG | ST-11 complex | Sporadic case | USA | SV-2 | Opc- |
| 31311 | NM3147 | Neisseria meningitidis | NG | ST-11 complex | Endemic | USA | SV-2 | Opc- |
| 31312 | 2001072 | Neisseria meningitidis | NG | ST-11 complex | Endemic | Mauritius | SV-2 | Opc- |
| 31313 | 2001073 | Neisseria meningitidis | NG | No value | Endemic | Mauritius | SV-2 | Opc- |
| 31314 | 2005079 | Neisseria meningitidis | NG | ST-11 complex | Endemic | Chad | SV-2 | Opc- |
| 31315 | 2005040 | Neisseria meningitidis | NG | ST-11 complex | Endemic | Chad | SV-2 | Opc- |
| 31316 | 2002004 | Neisseria meningitidis | NG | ST-11 complex | Endemic | France | SV-2 | Opc- |
| 31317 | NM3686 | Neisseria meningitidis | NG | ST-11 complex | Endemic | Brazil | SV-2 | Opc- |
| 31318 | NM3682 | Neisseria meningitidis | NG | ST-11 complex | Sporadic case | UK | SV-2 | Opc- |
| 31319 | M10208 | Neisseria meningitidis | NG | ST-11 complex | Endemic | Chile | SV-2 | Opc- |
| 31320 | NM3687 | Neisseria meningitidis | NG | ST-11 complex | Sporadic case | Brazil | SV-2 | Opc- |
| 31321 | NM3681 | Neisseria meningitidis | NG | ST-11 complex | Sporadic case | The Gambia | SV-2 | Opc- |
| 31322 | M20599 | Neisseria meningitidis | NG | ST-11 complex | Sporadic case | Chile | SV-2 | Opc- |
| 31323 | M1412 | Neisseria meningitidis | NG | ST-11 complex | Endemic | Chile | SV-2 | Opc- |
| 31324 | M12611 | Neisseria meningitidis | NG | No value | Endemic | Chile | SV-2 | Opc- |
| 31325 | NM3680 | Neisseria meningitidis | NG | No value | Sporadic case | Mali | SV-2 | Opc- |
| 31326 | NM3684 | Neisseria meningitidis | NG | No value | Sporadic case | The Netherlands | SV-2 | Opc- |
| 31328 | NM3688 | Neisseria meningitidis | NG | No value | Endemic | Brazil | SV-2 | Opc- |
| 31332 | NM3683 | Neisseria meningitidis | NG | ST-11 complex | No value | Canada | SV-2 | Opc- |
| 34502 | M13 240484 | Neisseria meningitidis | Y | ST-23 complex | No value | UK | SV-11 | Opc+ |
| 34503 | M13 240485 | Neisseria meningitidis | Y | ST-23 complex | No value | UK | SV-11 | Opc+ |
| 34504 | M13 240486 | Neisseria meningitidis | B | ST-269 complex | No value | UK | SV-1 | Opc+ |
| 34505 | M13 240488 | Neisseria meningitidis | B | ST-32 complex | No value | UK | SV-1 | Opc+ |
| 34506 | M13 240490 | Neisseria meningitidis | B | ST-269 complex | No value | UK | SV-8 | Opc+ |
| 34507 | M13 240495 | Neisseria meningitidis | B | ST-41/44 complex | No value | UK | SV-2 | Opc+ |
| 34508 | M13 240497 | Neisseria meningitidis | B | ST-41/44 complex | No value | UK | SV-10 | Opc+ |
| 34509 | M13 240499 | Neisseria meningitidis | B | ST-269 complex | No value | UK | SV-1 | Opc+ |
| 34510 | M13 240501 | Neisseria meningitidis | B | ST-41/44 complex | No value | UK | SV-2 | Opc+ |
| 34511 | M13 240502 | Neisseria meningitidis | B | ST-32 complex | No value | UK | SV-1 | Opc+ |
| 34512 | M13 240503 | Neisseria meningitidis | W | ST-22 complex | No value | UK | SV-12 | Opc+ |
| 34513 | M13 240507 | Neisseria meningitidis | B | ST-174 complex | No value | UK | SV-7 | Opc+ |
| 34514 | M13 240508 | Neisseria meningitidis | B | ST-269 complex | No value | UK | SV-1 | Opc+ |
| 34515 | M13 240516 | Neisseria meningitidis | B | ST-32 complex | No value | UK | SV-1 | Opc+ |
| 34516 | M13 240519 | Neisseria meningitidis | B | ST-213 complex | No value | UK | SV-12 | Opc- |

| | | | | | | | | |
|---|---|---|---|---|---|---|---|---|
| 34517 | M13 240525 | Neisseria meningitidis | B | ST-41/44 complex | No value | UK | SV-2 | Opc+ |
| 34540 | M07 240954 | Neisseria meningitidis | C | ST-11 complex | No value | UK | SV-2 | Opc- |
| 34541 | M00 243289 | Neisseria meningitidis | C | ST-11 complex | No value | UK | SV-2 | Opc- |
| 34542 | NZ98/254 | Neisseria meningitidis | B | ST-41/44 complex | No value | New Zealand | SV-6 | Opc+ |
| 34543 | M01 240070 | Neisseria meningitidis | W | ST-22 complex | No value | UK | SV-12 | Opc+ |
| 34544 | M14 240183 | Neisseria meningitidis | C | ST-11 complex | No value | Canada | SV-2 | Opc- |
| 34545 | M14 240188 | Neisseria meningitidis | C | ST-11 complex | No value | Canada | SV-2 | Opc- |
| 34546 | M14 240191 | Neisseria meningitidis | C | ST-11 complex | No value | Canada | SV-2 | Opc- |
| 34547 | 4119 | Neisseria meningitidis | B | ST-32 complex | No value | Brazil | SV-1 | Opc+ |
| 34548 | 9506 | Neisseria meningitidis | C | ST-32 complex | No value | Brazil | SV-1 | Opc+ |
| 34549 | 9757 | Neisseria meningitidis | B | ST-32 complex | No value | Brazil | SV-1 | Opc+ |
| 34550 | 12888 | Neisseria meningitidis | C | ST-32 complex | No value | Brazil | SV-1 | Opc+ |
| 34551 | 61103 | Neisseria meningitidis | A | ST-1 complex | No value | Niger | SV-5 | Opc+ |
| 34552 | 61106 | Neisseria meningitidis | A | ST-1 complex | No value | Niger | SV-5 | Opc+ |
| 34553 | 63006 | Neisseria meningitidis | A | ST-4 complex | No value | Burkina Faso | SV-5 | Opc+ |
| 34554 | 63023 | Neisseria meningitidis | A | ST-1 complex | No value | Niger | SV-5 | Opc+ |
| 34555 | 63041 | Neisseria meningitidis | A | ST-4 complex | No value | Chad | SV-5 | Opc+ |
| 34556 | 63049 | Neisseria meningitidis | A | ST-4 complex | No value | USA | SV-5 | Opc+ |
| 34557 | 64182 | Neisseria meningitidis | A | ST-4 complex | No value | Niger | SV-5 | Opc+ |
| 34558 | 65012 | Neisseria meningitidis | A | ST-4 complex | No value | Niger | SV-5 | Opc+ |
| 34559 | 65014 | Neisseria meningitidis | A | ST-4 complex | No value | Niger | SV-5 | Opc+ |
| 34560 | 68094 | Neisseria meningitidis | B | ST-1 complex | No value | New Zealand | SV-5 | Opc+ |
| 34561 | 69096 | Neisseria meningitidis | A | ST-1 complex | No value | Mali | SV-5 | Opc+ |
| 34562 | 69100 | Neisseria meningitidis | A | ST-1 complex | No value | Mali | SV-5 | Opc+ |
| 34563 | 69155 | Neisseria meningitidis | A | ST-1 complex | No value | Algeria | SV-5 | Opc+ |
| 34564 | 69166 | Neisseria meningitidis | Z | ST-1 complex | No value | USA | SV-5 | Opc+ |
| 34565 | 69176 | Neisseria meningitidis | A | ST-1 complex | No value | Algeria | SV-5 | Opc+ |
| 34566 | 70012 | Neisseria meningitidis | A | ST-1 complex | No value | Chad | SV-5 | Opc+ |
| 34567 | 70021 | Neisseria meningitidis | A | ST-1 complex | No value | Algeria | SV-5 | Opc+ |
| 34568 | 70030 | Neisseria meningitidis | A | ST-1 complex | No value | Burkina Faso | SV-5 | Opc+ |
| 34569 | 70082 | Neisseria meningitidis | A | ST-1 complex | No value | Niger | SV-5 | Opc+ |
| 34570 | 73696 | Neisseria meningitidis | C | No value | No value | Brazil | SV-5 | Opc+ |
| 34571 | 73704 | Neisseria meningitidis | C | ST-11 complex | No value | Brazil | SV-2 | Opc- |
| 34572 | 75643 | Neisseria meningitidis | A | ST-5 complex | No value | Brazil | SV-5 | Opc+ |
| 34573 | 75689 | Neisseria meningitidis | A | ST-5 complex | No value | Brazil | SV-5 | Opc+ |
| 34574 | 77221 | Neisseria meningitidis | A | No value | No value | Burkina Faso | SV-5 | Opc+ |
| 34575 | 80179 | Neisseria meningitidis | E | ST-178 complex | No value | France | SV-1 | Opc- |
| 34576 | 81858 | Neisseria meningitidis | C | No value | No value | Vietnam | SV-7 | Opc- |
| 34577 | 87255 | Neisseria meningitidis | B | ST-11 complex | No value | Tunisia | SV-10 | Opc- |
| 34578 | 88050 | Neisseria meningitidis | A | ST-5 complex | No value | Chad | SV-5 | Opc+ |
| 34579 | 92045 | Neisseria meningitidis | E | No value | No value | France | SV-1 | Opc+ |
| 34580 | 93003 | Neisseria meningitidis | H | No value | No value | France | SV-8 | Opc+ |
| 34581 | 93004 | Neisseria meningitidis | NG | No value | No value | France | SV-2 | Opc- |
| 34582 | 94018 | Neisseria meningitidis | A | No value | No value | Congo | SV-5 | Opc+ |
| 34583 | 96023 | Neisseria meningitidis | A | ST-5 complex | No value | Niger | SV-5 | Opc+ |
| 34584 | 96024 | Neisseria meningitidis | A | ST-5 complex | No value | Niger | SV-5 | Opc+ |
| 34585 | 96037 | Neisseria meningitidis | B | ST-41/44 complex | No value | Cameroon | SV-10 | Opc- |
| 34586 | 96060 | Neisseria meningitidis | A | ST-1 complex | No value | Algeria | SV-5 | Opc+ |
| 34587 | 97008 | Neisseria meningitidis | A | ST-5 complex | No value | Niger | SV-5 | Opc+ |
| 34588 | 97014 | Neisseria meningitidis | X | ST-181 complex | No value | Niger | SV-5 | Opc+ |
| 34589 | 97018 | Neisseria meningitidis | A | ST-5 complex | No value | Mali | SV-5 | Opc+ |
| 34590 | 97020 | Neisseria meningitidis | A | ST-5 complex | No value | Mali | SV-5 | Opc+ |
| 34591 | 97021 | Neisseria meningitidis | A | ST-181 complex | No value | Chad | SV-5 | Opc+ |
| 34592 | 97027 | Neisseria meningitidis | A | ST-4 complex | No value | Niger | SV-5 | Opc+ |
| 34593 | 98002 | Neisseria meningitidis | X | ST-181 complex | No value | Niger | SV-5 | Opc+ |
| 34594 | 98005 | Neisseria meningitidis | A | ST-5 complex | No value | Niger | SV-5 | Opc+ |
| 34595 | 98008 | Neisseria meningitidis | E | ST-461 complex | No value | France | SV-5 | Opc- |
| 34596 | 98080 | Neisseria meningitidis | B | ST-11 complex | No value | Saudi Arabia | SV-10 | Opc- |
| 34597 | 2000063 | Neisseria meningitidis | A | ST-5 complex | No value | Niger | SV-5 | Opc+ |
| 34598 | 2000080 | Neisseria meningitidis | A | ST-5 complex | No value | Algeria | SV-5 | Opc+ |
| 34599 | 2001212 | Neisseria meningitidis | A | ST-5 complex | No value | Burkina Faso | SV-5 | Opc+ |
| 34600 | 2002007 | Neisseria meningitidis | A | ST-5 complex | No value | Niger | SV-5 | Opc+ |
| 34601 | 2002020 | Neisseria meningitidis | C | ST-32 complex | No value | Cameroon | SV-1 | Opc+ |
| 34602 | 2002030 | Neisseria meningitidis | B | ST-32 complex | No value | Cameroon | SV-1 | Opc+ |
| 34603 | 2002038 | Neisseria meningitidis | X | ST-181 complex | No value | Niger | SV-5 | Opc+ |
| 34604 | 2003022 | Neisseria meningitidis | A | ST-5 complex | No value | Niger | SV-5 | Opc+ |
| 34605 | 2003051 | Neisseria meningitidis | Y | No value | No value | Niger | SV-5 | Opc+ |
| 34606 | 2004085 | Neisseria meningitidis | A | ST-5 complex | No value | Niger | SV-5 | Opc+ |
| 34607 | 2004090 | Neisseria meningitidis | A | ST-5 complex | No value | Niger | SV-5 | Opc+ |
| 34608 | 2005172 | Neisseria meningitidis | X | ST-181 complex | No value | Niger | SV-5 | Opc+ |
| 34609 | 2006087 | Neisseria meningitidis | X | ST-181 complex | No value | Niger | SV-5 | Opc+ |
| 34610 | 2007056 | Neisseria meningitidis | A | ST-5 complex | No value | Burkina Faso | SV-5 | Opc+ |
| 34611 | 2007461 | Neisseria meningitidis | W | No value | No value | Togo | SV-5 | Opc+ |
| 34612 | 2008223 | Neisseria meningitidis | X | ST-181 complex | No value | Burkina Faso | SV-5 | Opc+ |

| | | | | | | | | |
|---|---|---|---|---|---|---|---|---|
| 34613 | M13255 | Neisseria meningitidis | C | ST-32 complex | No value | USA | SV-1 | Opc+ |
| 34614 | M13265 | Neisseria meningitidis | C | ST-32 complex | No value | USA | SV-1 | Opc+ |
| 34615 | NM003 | Neisseria meningitidis | B | ST-41/44 complex | No value | USA | SV-10 | Opc+ |
| 34616 | NM045 | Neisseria meningitidis | B | ST-41/44 complex | No value | USA | SV-5 | Opc- |
| 34617 | NM0552 | Neisseria meningitidis | B | ST-41/44 complex | No value | USA | SV-10 | Opc+ |
| 34618 | NM115 | Neisseria meningitidis | Y | ST-23 complex | No value | USA | SV-11 | Opc+ |
| 34619 | NM126 | Neisseria meningitidis | C | ST-11 complex | No value | USA | SV-2 | Opc- |
| 34620 | NM133 | Neisseria meningitidis | C | ST-11 complex | No value | USA | SV-2 | Opc- |
| 34621 | NM134 | Neisseria meningitidis | W | ST-174 complex | No value | USA | SV-1 | Opc+ |
| 34622 | NM140 | Neisseria meningitidis | Z | ST-103 complex | No value | USA | SV-5 | Opc+ |
| 34623 | NM1476 | Neisseria meningitidis | B | ST-32 complex | No value | USA | SV-1 | Opc+ |
| 34624 | NM1482 | Neisseria meningitidis | C | ST-11 complex | No value | USA | SV-2 | Opc- |
| 34625 | NM1495 | Neisseria meningitidis | C | ST-11 complex | No value | USA | SV-2 | Opc- |
| 34626 | NM151 | Neisseria meningitidis | B | No value | No value | USA | SV-2 | Opc+ |
| 34627 | NM165 | Neisseria meningitidis | Y | ST-23 complex | No value | USA | SV-11 | Opc+ |
| 34628 | NM183 | Neisseria meningitidis | Z | ST-103 complex | No value | USA | SV-5 | Opc+ |
| 34629 | NM23 | Neisseria meningitidis | C | ST-11 complex | No value | USA | SV-2 | Opc- |
| 34630 | NM255 | Neisseria meningitidis | X | ST-175 complex | No value | USA | SV-5 | Opc+ |
| 34631 | NM2657 | Neisseria meningitidis | NG | ST-60 complex | No value | USA | SV-11 | Opc+ |
| 34632 | NM27 | Neisseria meningitidis | Y | ST-23 complex | No value | USA | SV-11 | Opc+ |
| 34633 | NM271 | Neisseria meningitidis | Y | ST-23 complex | No value | USA | SV-11 | Opc+ |
| 34634 | NM2781 | Neisseria meningitidis | Z | ST-103 complex | No value | USA | SV-5 | Opc+ |
| 34635 | NM2795 | Neisseria meningitidis | NG | ST-198 complex | No value | USA | SV-7 | Opc+ |
| 34637 | NM3001 | Neisseria meningitidis | NG | ST-1157 complex | No value | USA | SV-1 | Opc+ |
| 34639 | NM3081 | Neisseria meningitidis | NG | No value | No value | USA | SV-10 | Opc+ |
| 34640 | NM313 | Neisseria meningitidis | C | ST-11 complex | No value | USA | SV-2 | Opc- |
| 34641 | NM3131 | Neisseria meningitidis | Y | ST-23 complex | No value | USA | SV-11 | Opc+ |
| 34642 | NM3139 | Neisseria meningitidis | B | ST-41/44 complex | No value | USA | SV-10 | Opc+ |
| 34643 | NM3141 | Neisseria meningitidis | B | ST-32 complex | No value | USA | SV-1 | Opc+ |
| 34644 | NM3144 | Neisseria meningitidis | Y | ST-23 complex | No value | USA | SV-11 | Opc+ |
| 34645 | NM3158 | Neisseria meningitidis | Y | ST-23 complex | No value | USA | SV-11 | Opc+ |
| 34646 | NM3164 | Neisseria meningitidis | Y | ST-23 complex | No value | USA | SV-11 | Opc+ |
| 34647 | NM3173 | Neisseria meningitidis | B | ST-32 complex | No value | USA | SV-1 | Opc+ |
| 34648 | NM32 | Neisseria meningitidis | C | ST-11 complex | No value | USA | SV-2 | Opc- |
| 34649 | NM3222 | Neisseria meningitidis | Y | ST-23 complex | No value | USA | SV-11 | Opc+ |
| 34650 | NM3223 | Neisseria meningitidis | Y | ST-23 complex | No value | USA | SV-11 | Opc+ |
| 34651 | NM3230 | Neisseria meningitidis | B | ST-41/44 complex | No value | USA | SV-2 | Opc+ |
| 34652 | NM35 | Neisseria meningitidis | C | ST-11 complex | No value | USA | SV-2 | Opc- |
| 34653 | NM36 | Neisseria meningitidis | C | ST-11 complex | No value | USA | SV-2 | Opc- |
| 34654 | NM3642 | Neisseria meningitidis | A | ST-5 complex | No value | Bangladesh | SV-5 | Opc+ |
| 34655 | NM3652 | Neisseria meningitidis | A | ST-5 complex | No value | Bangladesh | SV-5 | Opc+ |
| 34656 | NM418 | Neisseria meningitidis | B | ST-32 complex | No value | USA | SV-1 | Opc+ |
| 34657 | NM422 | Neisseria meningitidis | B | ST-32 complex | No value | USA | SV-1 | Opc+ |
| 34658 | NM43 | Neisseria meningitidis | C | ST-11 complex | No value | USA | SV-2 | Opc- |
| 34659 | NM477 | Neisseria meningitidis | B | ST-32 complex | No value | USA | SV-1 | Opc+ |
| 34660 | NM51 | Neisseria meningitidis | Y | ST-23 complex | No value | USA | SV-11 | Opc+ |
| 34661 | NM518 | Neisseria meningitidis | B | ST-41/44 complex | No value | USA | SV-10 | Opc+ |
| 34662 | NM576 | Neisseria meningitidis | Z | ST-103 complex | No value | USA | SV-5 | Opc+ |
| 34663 | NM604 | Neisseria meningitidis | A | ST-5 complex | No value | USA | SV-5 | Opc+ |
| 34664 | NM606 | Neisseria meningitidis | A | No value | No value | USA | SV-5 | Opc+ |
| 34665 | NM607 | Neisseria meningitidis | A | ST-5 complex | No value | USA | SV-5 | Opc+ |
| 34666 | NM80 | Neisseria meningitidis | Y | ST-23 complex | No value | USA | SV-11 | Opc+ |
| 34667 | NM82 | Neisseria meningitidis | C | ST-11 complex | No value | USA | SV-2 | Opc- |
| 34668 | NM90 | Neisseria meningitidis | Y | ST-23 complex | No value | USA | SV-11 | Opc+ |
| 34669 | NM94 | Neisseria meningitidis | C | ST-11 complex | No value | USA | SV-2 | Opc- |
| 34670 | NM95 | Neisseria meningitidis | C | No value | No value | USA | SV-2 | Opc- |
| 34731 | LNP13407 | Neisseria meningitidis | X | ST-181 complex | No value | Chad | SV-5 | Opc+ |
| 34732 | LNP14354 | Neisseria meningitidis | X | ST-181 complex | No value | Niger | SV-5 | Opc+ |
| 34733 | LNP15075 | Neisseria meningitidis | NG | No value | No value | Burkina Faso | SV-5 | Opc+ |
| 34734 | LNP23552 | Neisseria meningitidis | X | ST-181 complex | No value | Niger | SV-5 | Opc+ |
| 34735 | LNP23557 | Neisseria meningitidis | X | ST-181 complex | No value | Niger | SV-5 | Opc+ |
| 34736 | LNP19504 | Neisseria meningitidis | X | ST-254 complex | No value | France | SV-1 | Opc+ |
| 34737 | LNP24196 | Neisseria meningitidis | X | No value | No value | France | SV-1 | Opc+ |
| 34738 | LNP24287 | Neisseria meningitidis | X | ST-750 complex | No value | France | SV-1 | Opc+ |
| 34739 | 2008112 | Neisseria meningitidis | X | ST-181 complex | No value | Benin | SV-5 | Opc+ |
| 34740 | 2005166 | Neisseria meningitidis | X | ST-181 complex | No value | Niger | SV-5 | Opc+ |
| 34741 | 2006100 | Neisseria meningitidis | X | ST-181 complex | No value | Niger | SV-5 | Opc+ |
| 34742 | LNP27821 | Neisseria meningitidis | C | ST-11 complex | No value | France | SV-2 | Opc- |
| 34743 | LNP27684 | Neisseria meningitidis | B | ST-11 complex | No value | France | SV-2 | Opc- |
| 34744 | LNP27572 | Neisseria meningitidis | C | ST-11 complex | No value | France | SV-2 | Opc- |
| 34745 | 2008223B | Neisseria meningitidis | X | ST-181 complex | No value | Burkina Faso | SV-5 | Opc+ |
| 34805 | ERR051676 | Neisseria meningitidis | A | ST-5 complex | Carrier | Ghana | SV-5 | Opc+ |
| 34806 | ERR051677 | Neisseria meningitidis | A | ST-5 complex | Carrier | Ghana | SV-5 | Opc+ |
| 34807 | ERR051678 | Neisseria meningitidis | A | ST-5 complex | Carrier | Ghana | SV-5 | Opc+ |

| | | | | | | | | |
|---|---|---|---|---|---|---|---|---|
| 34808 | ERR052799 | Neisseria meningitidis | A | ST-5 complex | No value | Burkina Faso | SV-5 | Opc+ |
| 34809 | ERR052812 | Neisseria meningitidis | A | ST-5 complex | Carrier | Burkina Faso | SV-5 | Opc+ |
| 34810 | ERR052817 | Neisseria meningitidis | A | ST-5 complex | Carrier | Burkina Faso | SV-5 | Opc+ |
| 34811 | ERR052816 | Neisseria meningitidis | A | ST-5 complex | No value | Burkina Faso | SV-5 | Opc+ |
| 34812 | ERR052813 | Neisseria meningitidis | A | ST-5 complex | Carrier | Burkina Faso | SV-5 | Opc+ |
| 34813 | ERR052829 | Neisseria meningitidis | A | ST-5 complex | Carrier | Burkina Faso | SV-5 | Opc+ |
| 34814 | ERR052831 | Neisseria meningitidis | A | ST-5 complex | Carrier | Ghana | SV-5 | Opc+ |
| 34815 | ERR052822 | Neisseria meningitidis | A | ST-5 complex | Carrier | Ghana | SV-5 | Opc+ |
| 34816 | ERR052826 | Neisseria meningitidis | A | ST-5 complex | Carrier | Ghana | SV-5 | Opc+ |
| 34817 | ERR052811 | Neisseria meningitidis | A | ST-5 complex | Carrier | Burkina Faso | SV-5 | Opc+ |
| 34818 | ERR052800 | Neisseria meningitidis | A | ST-5 complex | No value | Burkina Faso | SV-5 | Opc+ |
| 34819 | ERR052821 | Neisseria meningitidis | A | ST-5 complex | Carrier | Burkina Faso | SV-5 | Opc+ |
| 34820 | ERR052795 | Neisseria meningitidis | A | ST-5 complex | Carrier | Burkina Faso | SV-5 | Opc+ |
| 34821 | ERR052823 | Neisseria meningitidis | A | ST-5 complex | Carrier | Ghana | SV-5 | Opc+ |
| 34822 | ERR052787 | Neisseria meningitidis | A | ST-5 complex | No value | Burkina Faso | SV-5 | Opc+ |
| 34823 | ERR052828 | Neisseria meningitidis | A | ST-5 complex | Carrier | Ghana | SV-5 | Opc+ |
| 34824 | ERR052792 | Neisseria meningitidis | A | ST-5 complex | Carrier | Burkina Faso | SV-5 | Opc+ |
| 34825 | ERR052767 | Neisseria meningitidis | A | ST-5 complex | No value | Ghana | SV-5 | Opc+ |
| 34826 | ERR052775 | Neisseria meningitidis | A | ST-5 complex | Carrier | Ghana | SV-5 | Opc+ |
| 34827 | ERR052754 | Neisseria meningitidis | A | ST-5 complex | Carrier | Ghana | SV-5 | Opc+ |
| 34828 | ERR052768 | Neisseria meningitidis | A | ST-5 complex | Carrier | Ghana | SV-5 | Opc+ |
| 34829 | ERR052746 | Neisseria meningitidis | A | ST-5 complex | Carrier | Ghana | SV-5 | Opc+ |
| 34830 | ERR052814 | Neisseria meningitidis | A | ST-5 complex | Carrier | Ghana | SV-5 | Opc+ |
| 34831 | ERR052819 | Neisseria meningitidis | A | ST-5 complex | Carrier | Ghana | SV-5 | Opc+ |
| 34832 | ERR052827 | Neisseria meningitidis | A | ST-5 complex | Carrier | Ghana | SV-5 | Opc+ |
| 34833 | ERR052825 | Neisseria meningitidis | A | ST-5 complex | Carrier | Ghana | SV-5 | Opc+ |
| 34834 | ERR052796 | Neisseria meningitidis | A | ST-5 complex | Carrier | Burkina Faso | SV-5 | Opc+ |
| 34835 | ERR052820 | Neisseria meningitidis | A | ST-5 complex | Carrier | Burkina Faso | SV-5 | Opc+ |
| 34836 | ERR052737 | Neisseria meningitidis | A | ST-5 complex | No value | Ghana | SV-5 | Opc+ |
| 34837 | ERR052779 | Neisseria meningitidis | A | ST-5 complex | No value | Ghana | SV-5 | Opc+ |
| 34838 | ERR052793 | Neisseria meningitidis | A | ST-5 complex | Carrier | Burkina Faso | SV-5 | Opc+ |
| 34839 | ERR052824 | Neisseria meningitidis | A | ST-5 complex | Carrier | Ghana | SV-5 | Opc+ |
| 34840 | ERR052747 | Neisseria meningitidis | A | ST-5 complex | No value | Ghana | SV-5 | Opc+ |
| 34841 | ERR052807 | Neisseria meningitidis | A | ST-5 complex | Carrier | Burkina Faso | SV-5 | Opc+ |
| 34842 | ERR052806 | Neisseria meningitidis | A | ST-5 complex | Carrier | Burkina Faso | SV-5 | Opc+ |
| 34843 | ERR052789 | Neisseria meningitidis | A | ST-5 complex | No value | Burkina Faso | SV-5 | Opc+ |
| 34844 | ERR052739 | Neisseria meningitidis | A | ST-5 complex | No value | Ghana | SV-5 | Opc+ |
| 34845 | ERR052763 | Neisseria meningitidis | A | ST-5 complex | No value | Ghana | SV-5 | Opc+ |
| 34846 | ERR052832 | Neisseria meningitidis | A | ST-5 complex | Carrier | Ghana | SV-5 | Opc+ |
| 34847 | ERR052790 | Neisseria meningitidis | A | ST-5 complex | No value | Burkina Faso | SV-5 | Opc+ |
| 34848 | ERR052764 | Neisseria meningitidis | A | ST-5 complex | No value | Ghana | SV-5 | Opc+ |
| 34849 | ERR052781 | Neisseria meningitidis | A | ST-5 complex | Carrier | Ghana | SV-5 | Opc+ |
| 34850 | ERR052749 | Neisseria meningitidis | A | ST-5 complex | No value | Ghana | SV-5 | Opc+ |
| 34851 | ERR052818 | Neisseria meningitidis | A | ST-5 complex | Carrier | Ghana | SV-5 | Opc+ |
| 34852 | ERR052750 | Neisseria meningitidis | A | ST-5 complex | No value | Ghana | SV-5 | Opc+ |
| 34853 | ERR052766 | Neisseria meningitidis | A | ST-5 complex | No value | Ghana | SV-5 | Opc+ |
| 34854 | ERR052815 | Neisseria meningitidis | A | ST-5 complex | No value | Burkina Faso | SV-5 | Opc+ |
| 34855 | ERR052751 | Neisseria meningitidis | A | ST-5 complex | No value | Ghana | SV-5 | Opc+ |
| 34856 | ERR052808 | Neisseria meningitidis | A | ST-5 complex | Carrier | Burkina Faso | SV-5 | Opc+ |
| 34857 | ERR052738 | Neisseria meningitidis | A | ST-5 complex | No value | Russia | SV-5 | Opc+ |
| 34858 | ERR052758 | Neisseria meningitidis | A | ST-5 complex | Carrier | Ghana | SV-5 | Opc+ |
| 34859 | ERR052743 | Neisseria meningitidis | A | ST-5 complex | No value | Ghana | SV-5 | Opc+ |
| 34860 | ERR052757 | Neisseria meningitidis | A | ST-5 complex | Carrier | Ghana | SV-5 | Opc+ |
| 34861 | ERR052745 | Neisseria meningitidis | A | ST-5 complex | Carrier | Ghana | SV-5 | Opc+ |
| 34862 | ERR052774 | Neisseria meningitidis | A | ST-5 complex | Carrier | Ghana | SV-5 | Opc+ |
| 34863 | ERR052791 | Neisseria meningitidis | A | ST-5 complex | Carrier | Burkina Faso | SV-5 | Opc+ |
| 34864 | ERR052810 | Neisseria meningitidis | A | ST-5 complex | Carrier | Burkina Faso | SV-5 | Opc+ |
| 34865 | ERR052778 | Neisseria meningitidis | A | ST-5 complex | No value | Ghana | SV-5 | Opc+ |
| 34866 | ERR052804 | Neisseria meningitidis | A | ST-5 complex | Carrier | Burkina Faso | SV-5 | Opc+ |
| 34867 | ERR052771 | Neisseria meningitidis | A | ST-5 complex | Carrier | Ghana | SV-5 | Opc+ |
| 34868 | ERR052765 | Neisseria meningitidis | A | ST-5 complex | No value | Ghana | SV-5 | Opc+ |
| 34869 | ERR052741 | Neisseria meningitidis | A | ST-5 complex | No value | Ghana | SV-5 | Opc+ |
| 34870 | ERR052786 | Neisseria meningitidis | A | ST-5 complex | Carrier | Ghana | SV-5 | Opc+ |
| 34871 | ERR052752 | Neisseria meningitidis | A | ST-5 complex | No value | Ghana | SV-5 | Opc+ |
| 34872 | ERR052805 | Neisseria meningitidis | A | ST-5 complex | Carrier | Burkina Faso | SV-5 | Opc+ |
| 34873 | ERR052780 | Neisseria meningitidis | A | ST-5 complex | Carrier | Ghana | SV-5 | Opc+ |
| 34874 | ERR052782 | Neisseria meningitidis | A | ST-5 complex | No value | Ghana | SV-5 | Opc+ |
| 34875 | ERR052744 | Neisseria meningitidis | A | ST-5 complex | No value | Ghana | SV-5 | Opc+ |
| 34876 | ERR052809 | Neisseria meningitidis | A | ST-5 complex | Carrier | Burkina Faso | SV-5 | Opc+ |
| 34877 | ERR052802 | Neisseria meningitidis | A | ST-5 complex | No value | Burkina Faso | SV-5 | Opc+ |
| 34878 | ERR052759 | Neisseria meningitidis | A | ST-5 complex | Carrier | Ghana | SV-5 | Opc+ |
| 34879 | ERR052773 | Neisseria meningitidis | A | ST-5 complex | Carrier | Ghana | SV-5 | Opc+ |
| 34880 | ERR052740 | Neisseria meningitidis | A | ST-5 complex | No value | Ghana | SV-5 | Opc+ |
| 34881 | ERR052756 | Neisseria meningitidis | A | ST-5 complex | Carrier | Ghana | SV-5 | Opc+ |

| | | | | | | | | |
|---|---|---|---|---|---|---|---|---|
| 34882 | ERR052830 | Neisseria meningitidis | A | ST-5 complex | Carrier | Burkina Faso | SV-5 | Opc+ |
| 34883 | ERR052794 | Neisseria meningitidis | A | ST-5 complex | Carrier | Burkina Faso | SV-5 | Opc+ |
| 34884 | ERR052755 | Neisseria meningitidis | A | ST-5 complex | Carrier | Ghana | SV-5 | Opc+ |
| 34885 | ERR052784 | Neisseria meningitidis | A | ST-5 complex | Carrier | Ghana | SV-5 | Opc+ |
| 34886 | ERR052761 | Neisseria meningitidis | A | ST-5 complex | No value | Ghana | SV-5 | Opc+ |
| 34887 | ERR052753 | Neisseria meningitidis | A | ST-5 complex | No value | Ghana | SV-5 | Opc+ |
| 34888 | ERR052801 | Neisseria meningitidis | A | ST-5 complex | No value | Burkina Faso | SV-5 | Opc+ |
| 34889 | ERR052783 | Neisseria meningitidis | A | ST-5 complex | No value | Ghana | SV-5 | Opc+ |
| 34890 | ERR052798 | Neisseria meningitidis | A | ST-5 complex | No value | Burkina Faso | SV-5 | Opc+ |
| 34891 | ERR052770 | Neisseria meningitidis | A | ST-5 complex | Carrier | Ghana | SV-5 | Opc+ |
| 34892 | ERR052788 | Neisseria meningitidis | A | ST-5 complex | No value | Burkina Faso | SV-5 | Opc+ |
| 34893 | ERR052803 | Neisseria meningitidis | A | ST-5 complex | Carrier | Burkina Faso | SV-5 | Opc+ |
| 34894 | ERR052797 | Neisseria meningitidis | A | ST-5 complex | No value | Burkina Faso | SV-5 | Opc+ |
| 34895 | ERR052760 | Neisseria meningitidis | A | ST-5 complex | No value | Ghana | SV-5 | Opc+ |
| 34896 | ERR052777 | Neisseria meningitidis | A | ST-5 complex | Carrier | Ghana | SV-5 | Opc+ |
| 34897 | ERR052785 | Neisseria meningitidis | A | ST-5 complex | Carrier | Ghana | SV-5 | Opc+ |
| 34898 | ERR052776 | Neisseria meningitidis | A | ST-5 complex | Carrier | Ghana | SV-5 | Opc+ |
| 34899 | ERR052762 | Neisseria meningitidis | A | ST-5 complex | No value | Ghana | SV-5 | Opc+ |
| 34900 | ERR052742 | Neisseria meningitidis | A | ST-5 complex | No value | Ghana | SV-5 | Opc+ |
| 34901 | ERR052772 | Neisseria meningitidis | A | ST-5 complex | Carrier | Ghana | SV-5 | Opc+ |
| 34902 | ERR052748 | Neisseria meningitidis | A | ST-5 complex | No value | Ghana | SV-5 | Opc+ |
| 35094 | ERR051675 | Neisseria meningitidis | A | ST-5 complex | Carrier | Ghana | SV-5 | Opc+ |
| 35095 | ERR052769 | Neisseria meningitidis | A | ST-5 complex | Carrier | Ghana | SV-5 | Opc+ |
| 35120 | 0001/84 | Neisseria meningitidis | B | ST-11 complex | No value | Czech Republic | SV-2 | Opc- |
| 35121 | 0016/84 | Neisseria meningitidis | B | ST-11 complex | No value | Czech Republic | SV-2 | Opc- |
| 35122 | 0364/95 | Neisseria meningitidis | B | ST-11 complex | No value | Czech Republic | SV-2 | Opc- |
| 35123 | 0448/95 | Neisseria meningitidis | B | ST-11 complex | No value | Czech Republic | SV-2 | Opc- |
| 35124 | 0501/95 | Neisseria meningitidis | B | ST-11 complex | No value | Czech Republic | SV-2 | Opc- |
| 35125 | 0018/99 | Neisseria meningitidis | B | ST-11 complex | No value | Czech Republic | SV-2 | Opc- |
| 35126 | 0287/99 | Neisseria meningitidis | B | ST-11 complex | No value | Czech Republic | SV-2 | Opc- |
| 35127 | 0010/00 | Neisseria meningitidis | B | ST-11 complex | No value | Czech Republic | SV-2 | Opc- |
| 35128 | 0138/00 | Neisseria meningitidis | B | ST-11 complex | No value | Czech Republic | SV-2 | Opc- |
| 35129 | 0010/01 | Neisseria meningitidis | B | ST-11 complex | No value | Czech Republic | SV-2 | Opc- |
| 35130 | 0046/02 | Neisseria meningitidis | B | ST-11 complex | No value | Czech Republic | SV-2 | Opc- |
| 35131 | 0039/96 | Neisseria meningitidis | W | ST-11 complex | No value | Czech Republic | SV-2 | Opc- |
| 35227 | 09.1126.Y | Neisseria meningitidis | B | ST-269 complex | No value | UK | SV-8 | Opc+ |
| 35228 | 09.1208.P | Neisseria meningitidis | B | ST-162 complex | No value | UK | SV-1 | Opc+ |
| 35229 | 09.1211.Y | Neisseria meningitidis | B | ST-269 complex | No value | UK | SV-8 | Opc+ |
| 35230 | 09.1292.T | Neisseria meningitidis | B | ST-162 complex | No value | UK | SV-1 | Opc+ |
| 35231 | 09.1331.Y | Neisseria meningitidis | B | ST-41/44 complex | No value | UK | SV-2 | Opc+ |
| 35232 | 09.1368.F | Neisseria meningitidis | B | ST-41/44 complex | No value | UK | SV-10 | Opc+ |
| 35233 | 09.1385.H | Neisseria meningitidis | Y | No value | No value | UK | SV-11 | Opc+ |
| 35234 | 09.1402.B | Neisseria meningitidis | B | ST-213 complex | No value | UK | SV-12 | Opc- |
| 35235 | 09.1453.F | Neisseria meningitidis | B | ST-41/44 complex | No value | UK | SV-2 | Opc+ |
| 35236 | 09.1507.Q | Neisseria meningitidis | B | ST-41/44 complex | No value | UK | SV-2 | Opc+ |
| 35237 | 09.1514.A | Neisseria meningitidis | B | ST-269 complex | No value | UK | SV-8 | Opc+ |
| 35238 | 09.1557.B | Neisseria meningitidis | B | ST-213 complex | No value | UK | SV-12 | Opc- |
| 35239 | 09.1580.V | Neisseria meningitidis | B | ST-32 complex | No value | UK | SV-1 | Opc+ |
| 35240 | 09.1595.J | Neisseria meningitidis | B | ST-35 complex | No value | UK | SV-1 | Opc+ |
| 35241 | 09.1619.R | Neisseria meningitidis | B | ST-41/44 complex | No value | UK | SV-2 | Opc+ |
| 35242 | 09.1857.M | Neisseria meningitidis | B | ST-1157 complex | No value | UK | SV-1 | Opc+ |
| 35243 | 09.1858.V | Neisseria meningitidis | B | ST-269 complex | No value | UK | SV-1 | Opc+ |
| 35244 | 09.1893.P | Neisseria meningitidis | B | ST-269 complex | No value | UK | SV-1 | Opc+ |
| 35245 | 09.1976.T | Neisseria meningitidis | B | No value | No value | UK | SV-8 | Opc+ |
| 35246 | 09.1999.X | Neisseria meningitidis | B | ST-162 complex | No value | UK | SV-1 | Opc+ |
| 35247 | 09.2014.G | Neisseria meningitidis | B | ST-41/44 complex | No value | UK | SV-6 | Opc+ |
| 35248 | 09.2045.J | Neisseria meningitidis | B | ST-41/44 complex | No value | UK | SV-2 | Opc+ |
| 35249 | 09.2061.P | Neisseria meningitidis | Y | ST-23 complex | No value | UK | SV-11 | Opc+ |
| 35250 | 09.2082.C | Neisseria meningitidis | B | ST-41/44 complex | No value | UK | SV-7 | Opc- |
| 35251 | 09.2204.A | Neisseria meningitidis | B | ST-60 complex | No value | UK | SV-11 | Opc+ |
| 35252 | 09.2270.V | Neisseria meningitidis | B | ST-41/44 complex | No value | UK | SV-5 | Opc+ |
| 35253 | 09.2386.V | Neisseria meningitidis | B | ST-41/44 complex | No value | UK | SV-6 | Opc+ |
| 35254 | 09.2439.W | Neisseria meningitidis | B | ST-213 complex | No value | UK | SV-12 | Opc- |
| 35255 | 09.2522.Q | Neisseria meningitidis | B | ST-41/44 complex | No value | UK | SV-6 | Opc+ |
| 35256 | 09.2622.N | Neisseria meningitidis | B | ST-269 complex | No value | UK | SV-1 | Opc+ |
| 35257 | 09.2658.E | Neisseria meningitidis | B | ST-41/44 complex | No value | UK | SV-2 | Opc+ |
| 35258 | 09.2799.W | Neisseria meningitidis | W | ST-22 complex | No value | UK | SV-12 | Opc+ |
| 35259 | 09.2935.B | Neisseria meningitidis | B | ST-103 complex | No value | UK | SV-5 | Opc+ |
| 35260 | 09.2939.N | Neisseria meningitidis | B | ST-213 complex | No value | UK | SV-12 | Opc- |
| 35261 | 09.2961.S | Neisseria meningitidis | B | ST-41/44 complex | No value | UK | SV-2 | Opc+ |
| 35262 | 09.3026.N | Neisseria meningitidis | B | No value | No value | UK | SV-8 | Opc+ |
| 35263 | 09.3029.P | Neisseria meningitidis | B | No value | No value | UK | SV-8 | Opc+ |
| 35264 | 09.3063.L | Neisseria meningitidis | B | ST-162 complex | No value | UK | SV-1 | Opc+ |
| 35265 | 09.3091.W | Neisseria meningitidis | B | ST-269 complex | No value | UK | SV-8 | Opc+ |

| | | | | | | | | |
|---|---|---|---|---|---|---|---|---|
| 35266 | 09.3167.H | Neisseria meningitidis | Y | ST-174 complex | No value | UK | SV-1 | Opc+ |
| 35267 | 09.3271.E | Neisseria meningitidis | B | ST-41/44 complex | No value | Iceland | SV-2 | Opc+ |
| 35268 | 09.3302.K | Neisseria meningitidis | Y | ST-174 complex | No value | UK | SV-1 | Opc+ |
| 35269 | 09.3351.N | Neisseria meningitidis | B | ST-269 complex | No value | UK | SV-1 | Opc+ |
| 35270 | 09.3368.H | Neisseria meningitidis | B | ST-41/44 complex | No value | UK | SV-2 | Opc+ |
| 35271 | 10.1171.X | Neisseria meningitidis | Y | ST-23 complex | No value | UK | SV-11 | Opc+ |
| 35272 | 10.1177.P | Neisseria meningitidis | B | ST-32 complex | No value | UK | SV-1 | Opc+ |
| 35273 | 10.1199.K | Neisseria meningitidis | B | ST-269 complex | No value | UK | SV-1 | Opc+ |
| 35274 | 10.1352.R | Neisseria meningitidis | B | ST-213 complex | No value | UK | SV-12 | Opc- |
| 35275 | 10.1430.T | Neisseria meningitidis | B | ST-32 complex | No value | UK | SV-1 | Opc+ |
| 35276 | 10.1516.S | Neisseria meningitidis | B | ST-269 complex | No value | UK | SV-1 | Opc+ |
| 35277 | 10.1718.Z | Neisseria meningitidis | B | ST-60 complex | No value | UK | SV-11 | Opc+ |
| 35278 | 10.1746.F | Neisseria meningitidis | B | ST-269 complex | No value | UK | SV-8 | Opc+ |
| 35279 | 10.1962.Z | Neisseria meningitidis | B | No value | No value | UK | SV-8 | Opc+ |
| 35280 | 10.2012.H | Neisseria meningitidis | B | ST-41/44 complex | No value | UK | SV-2 | Opc+ |
| 35281 | 10.2126.Z | Neisseria meningitidis | B | ST-269 complex | No value | UK | SV-1 | Opc+ |
| 35282 | 10.2240.T | Neisseria meningitidis | B | ST-269 complex | No value | UK | SV-8 | Opc+ |
| 35283 | 10.2273.P | Neisseria meningitidis | W | ST-22 complex | No value | UK | SV-12 | Opc+ |
| 35284 | 10.2289.A | Neisseria meningitidis | B | ST-269 complex | No value | UK | SV-8 | Opc+ |
| 35285 | 10.2352.E | Neisseria meningitidis | B | ST-269 complex | No value | UK | SV-8 | Opc+ |
| 35286 | 10.2397.V | Neisseria meningitidis | B | ST-35 complex | No value | UK | SV-2 | Opc+ |
| 35287 | 10.2422.K | Neisseria meningitidis | B | ST-41/44 complex | No value | UK | SV-6 | Opc+ |
| 35288 | 10.2509.N | Neisseria meningitidis | B | ST-269 complex | No value | UK | SV-1 | Opc+ |
| 35289 | 10.2631.L | Neisseria meningitidis | Z | ST-103 complex | No value | UK | SV-5 | Opc+ |
| 35290 | 10.2763.M | Neisseria meningitidis | B | ST-41/44 complex | No value | UK | SV-2 | Opc+ |
| 35291 | 10.2850.R | Neisseria meningitidis | B | ST-41/44 complex | No value | UK | SV-2 | Opc+ |
| 35292 | 10.2854.Q | Neisseria meningitidis | B | ST-213 complex | No value | UK | SV-12 | Opc- |
| 35293 | 10.2884.V | Neisseria meningitidis | B | ST-269 complex | No value | UK | SV-8 | Opc+ |
| 35294 | 10.3051.M | Neisseria meningitidis | B | ST-41/44 complex | No value | UK | SV-10 | Opc+ |
| 35295 | 10.3134.R | Neisseria meningitidis | Y | ST-22 complex | No value | UK | SV-12 | Opc+ |
| 35296 | 11.1071.G | Neisseria meningitidis | B | ST-41/44 complex | No value | UK | SV-6 | Opc+ |
| 35297 | 11.1190.L | Neisseria meningitidis | B | ST-41/44 complex | No value | UK | SV-2 | Opc+ |
| 35298 | 11.1286.D | Neisseria meningitidis | B | ST-41/44 complex | No value | UK | SV-2 | Opc+ |
| 35299 | 11.1312.X | Neisseria meningitidis | B | ST-41/44 complex | No value | UK | SV-7 | Opc+ |
| 35300 | 11.1376.W | Neisseria meningitidis | B | ST-32 complex | No value | UK | SV-1 | Opc+ |
| 35301 | 11.1392.G | Neisseria meningitidis | B | ST-269 complex | No value | UK | SV-1 | Opc+ |
| 35302 | 11.1426.J | Neisseria meningitidis | B | ST-41/44 complex | No value | UK | SV-2 | Opc+ |
| 35303 | 11.1479.T | Neisseria meningitidis | B | ST-41/44 complex | No value | UK | SV-2 | Opc+ |
| 35304 | 11.1489.Z | Neisseria meningitidis | B | ST-60 complex | No value | UK | SV-11 | Opc+ |
| 35305 | 11.1575.Q | Neisseria meningitidis | B | No value | No value | UK | SV-12 | Opc+ |
| 35306 | 11.1688.D | Neisseria meningitidis | B | ST-213 complex | No value | UK | SV-12 | Opc- |
| 35307 | 11.1709.B | Neisseria meningitidis | B | ST-213 complex | No value | UK | SV-12 | Opc- |
| 35308 | 11.1825.A | Neisseria meningitidis | B | ST-41/44 complex | No value | UK | SV-10 | Opc+ |
| 35309 | 11.1826.C | Neisseria meningitidis | B | ST-41/44 complex | No value | UK | SV-10 | Opc+ |
| 35310 | 11.2087.F | Neisseria meningitidis | B | ST-213 complex | No value | UK | SV-12 | Opc- |
| 35311 | 11.2169.T | Neisseria meningitidis | B | ST-269 complex | No value | UK | SV-8 | Opc+ |
| 35312 | 11.2463.D | Neisseria meningitidis | B | ST-41/44 complex | No value | UK | SV-2 | Opc+ |
| 35313 | 11.2525.L | Neisseria meningitidis | B | ST-269 complex | No value | UK | SV-8 | Opc+ |
| 35314 | 11.2909.Z | Neisseria meningitidis | Y | ST-23 complex | No value | UK | SV-11 | Opc+ |
| 35315 | 11.3133.X | Neisseria meningitidis | B | ST-162 complex | No value | UK | SV-1 | Opc+ |
| 35316 | 11.3808.A | Neisseria meningitidis | B | ST-269 complex | No value | UK | SV-1 | Opc+ |
| 35317 | 11.3888.K | Neisseria meningitidis | Y | ST-23 complex | No value | UK | SV-11 | Opc+ |
| 35318 | 11.4891.D | Neisseria meningitidis | B | ST-269 complex | No value | UK | SV-8 | Opc+ |
| 35319 | 11.4999.T | Neisseria meningitidis | B | ST-41/44 complex | No value | UK | SV-6 | Opc+ |
| 35320 | 11.5380.D | Neisseria meningitidis | B | No value | No value | UK | SV-10 | Opc+ |
| 35321 | 11.5384.H | Neisseria meningitidis | B | ST-269 complex | No value | UK | SV-8 | Opc+ |
| 35322 | 11.5445.E | Neisseria meningitidis | B | ST-1157 complex | No value | UK | SV-1 | Opc+ |
| 35323 | 11.5563.G | Neisseria meningitidis | W | ST-22 complex | No value | UK | SV-12 | Opc+ |
| 35324 | 11.5842.B | Neisseria meningitidis | B | ST-41/44 complex | No value | UK | SV-6 | Opc+ |
| 35325 | 11.5915.A | Neisseria meningitidis | B | No value | No value | UK | SV-12 | Opc- |
| 35326 | 11.6644.A | Neisseria meningitidis | B | ST-269 complex | No value | UK | SV-8 | Opc+ |
| 35327 | 11.6835.D | Neisseria meningitidis | B | ST-35 complex | No value | UK | SV-12 | Opc+ |
| 35328 | 11.7141.B | Neisseria meningitidis | B | ST-269 complex | No value | UK | SV-1 | Opc+ |
| 35329 | 12.1092.Y | Neisseria meningitidis | B | ST-103 complex | No value | UK | SV-5 | Opc+ |
| 35330 | 12.1192.H | Neisseria meningitidis | B | ST-461 complex | No value | UK | SV-1 | Opc- |
| 35331 | 12.1222.T | Neisseria meningitidis | B | ST-60 complex | No value | UK | SV-12 | Opc+ |
| 35332 | 12.1512.D | Neisseria meningitidis | Y | ST-23 complex | No value | UK | SV-11 | Opc+ |
| 35333 | 12.1555.H | Neisseria meningitidis | W | ST-22 complex | No value | UK | SV-12 | Opc+ |
| 35334 | 12.8515.D | Neisseria meningitidis | B | ST-269 complex | No value | UK | SV-8 | Opc+ |
| 35335 | 12.8534.X | Neisseria meningitidis | B | ST-41/44 complex | No value | UK | SV-2 | Opc+ |
| 35336 | 12.8840.D | Neisseria meningitidis | Y | ST-23 complex | No value | UK | SV-11 | Opc+ |
| 35337 | 13.1012.H | Neisseria meningitidis | B | ST-269 complex | No value | UK | SV-8 | Opc+ |
| 35338 | 13.1296.J | Neisseria meningitidis | Y | ST-23 complex | No value | UK | SV-11 | Opc+ |
| 35339 | 13.1297.B | Neisseria meningitidis | B | ST-269 complex | No value | UK | SV-8 | Opc+ |

| | | | | | | | | |
|---|---|---|---|---|---|---|---|---|
| 35340 | 13.1370.Z | Neisseria meningitidis | B | ST-213 complex | No value | UK | SV-12 | Opc- |
| 35341 | 13.1395.T | Neisseria meningitidis | B | ST-269 complex | No value | UK | SV-8 | Opc+ |
| 35342 | 13.1396.M | Neisseria meningitidis | B | ST-269 complex | No value | UK | SV-1 | Opc+ |
| 35343 | 13.1682.M | Neisseria meningitidis | B | ST-269 complex | No value | UK | SV-8 | Opc+ |
| 35344 | 13.2342.Z | Neisseria meningitidis | B | ST-269 complex | No value | UK | SV-8 | Opc+ |
| 35345 | 13.2973.F | Neisseria meningitidis | B | ST-461 complex | No value | UK | SV-5 | Opc- |
| 35346 | 13.3065.D | Neisseria meningitidis | B | ST-461 complex | No value | UK | SV-1 | Opc- |
| 35347 | 13.3207.X | Neisseria meningitidis | B | ST-213 complex | No value | UK | SV-12 | Opc- |
| 35348 | 13.3348.S | Neisseria meningitidis | Y | ST-23 complex | No value | UK | SV-11 | Opc+ |
| 35349 | 13.3789.S | Neisseria meningitidis | Y | ST-23 complex | No value | UK | SV-11 | Opc+ |
| 35350 | 13.3813.B | Neisseria meningitidis | B | ST-269 complex | No value | UK | SV-8 | Opc+ |
| 35351 | 13.3867.V | Neisseria meningitidis | B | ST-269 complex | No value | UK | SV-8 | Opc+ |
| 35352 | 13.4061.L | Neisseria meningitidis | B | ST-269 complex | No value | UK | SV-8 | Opc+ |
| 35353 | 13.4142.L | Neisseria meningitidis | B | ST-269 complex | No value | UK | SV-1 | Opc+ |
| 35354 | 13.4278.S | Neisseria meningitidis | B | ST-41/44 complex | No value | UK | SV-2 | Opc+ |
| 35355 | 13.4514.P | Neisseria meningitidis | B | ST-32 complex | No value | UK | SV-1 | Opc+ |
| 35356 | 12.1860.E | Neisseria meningitidis | B | ST-269 complex | No value | UK | SV-8 | Opc+ |
| 35357 | 12.1954.S | Neisseria meningitidis | B | ST-269 complex | No value | UK | SV-8 | Opc+ |
| 35358 | 12.2326.H | Neisseria meningitidis | B | No value | No value | UK | SV-10 | Opc+ |
| 35359 | 12.2412.W | Neisseria meningitidis | Y | ST-23 complex | No value | UK | SV-11 | Opc+ |
| 35360 | 12.2589.T | Neisseria meningitidis | B | ST-162 complex | No value | UK | SV-1 | Opc+ |
| 35361 | 12.2953.Y | Neisseria meningitidis | B | No value | No value | UK | SV-10 | Opc+ |
| 35362 | 12.3021.W | Neisseria meningitidis | B | ST-41/44 complex | No value | UK | SV-2 | Opc+ |
| 35363 | 12.3084.V | Neisseria meningitidis | B | No value | No value | UK | SV-6 | Opc+ |
| 35364 | 12.4811.X | Neisseria meningitidis | B | ST-269 complex | No value | UK | SV-8 | Opc+ |
| 35365 | 12.4819.T | Neisseria meningitidis | B | ST-269 complex | No value | UK | SV-1 | Opc+ |
| 35366 | 12.4885.X | Neisseria meningitidis | C | No value | No value | UK | SV-2 | Opc+ |
| 35367 | 12.4886.L | Neisseria meningitidis | C | No value | No value | UK | SV-2 | Opc+ |
| 35368 | 12.6364.V | Neisseria meningitidis | B | No value | No value | UK | SV-10 | Opc+ |
| 35369 | 12.6501.H | Neisseria meningitidis | B | ST-213 complex | No value | UK | SV-12 | Opc- |
| 35370 | 12.7012.F | Neisseria meningitidis | Y | ST-23 complex | No value | UK | SV-5 | Opc+ |
| 35371 | 12.7350.H | Neisseria meningitidis | W | ST-11 complex | No value | UK | SV-2 | Opc- |
| 35372 | 12.7377.D | Neisseria meningitidis | B | No value | No value | UK | SV-8 | Opc+ |
| 35373 | 12.7548.W | Neisseria meningitidis | B | ST-269 complex | No value | UK | SV-8 | Opc+ |
| 35374 | 12.8180.Y | Neisseria meningitidis | B | ST-269 complex | No value | UK | SV-8 | Opc+ |
| 35375 | 12.8381.Y | Neisseria meningitidis | B | ST-269 complex | No value | UK | SV-8 | Opc+ |
| 35376 | 12.8408.J | Neisseria meningitidis | Y | ST-23 complex | No value | UK | SV-11 | Opc+ |
| 35377 | 12.8444.A | Neisseria meningitidis | C | ST-8 complex | No value | UK | SV-10 | Opc- |
| 35378 | 09.1936.F | Neisseria meningitidis | B | ST-41/44 complex | No value | UK | SV-2 | Opc+ |
| 35379 | 09.2807.A | Neisseria meningitidis | B | ST-213 complex | No value | UK | SV-12 | Opc- |
| 35380 | 12.3882.E | Neisseria meningitidis | Y | ST-22 complex | No value | UK | SV-12 | Opc+ |
| 35381 | 13.4765.S | Neisseria meningitidis | B | ST-41/44 complex | No value | UK | SV-2 | Opc+ |
| 35382 | 13.4767.Q | Neisseria meningitidis | Y | ST-23 complex | No value | UK | SV-11 | Opc+ |
| 35383 | 13.5135.Z | Neisseria meningitidis | B | ST-35 complex | No value | UK | SV-12 | Opc+ |
| 35384 | 13.5154.G | Neisseria meningitidis | Y | ST-23 complex | No value | UK | SV-11 | Opc+ |
| 35385 | 13.5226.K | Neisseria meningitidis | B | No value | No value | UK | SV-8 | Opc+ |
| 35386 | 13.5556.G | Neisseria meningitidis | B | ST-213 complex | No value | UK | SV-12 | Opc- |
| 35387 | 13.5557.N | Neisseria meningitidis | B | ST-213 complex | No value | UK | SV-12 | Opc- |
| 35388 | 13.6067.H | Neisseria meningitidis | B | ST-41/44 complex | No value | UK | SV-5 | Opc+ |
| 35389 | 13.6117.K | Neisseria meningitidis | B | ST-1157 complex | No value | UK | SV-1 | Opc+ |
| 35390 | 13.6161.J | Neisseria meningitidis | B | ST-41/44 complex | No value | UK | SV-7 | Opc+ |
| 35391 | 13.6352.Q | Neisseria meningitidis | Y | ST-23 complex | No value | UK | SV-11 | Opc+ |
| 35392 | 13.6756.W | Neisseria meningitidis | B | ST-41/44 complex | No value | UK | SV-6 | Opc+ |
| 35393 | 13.7214.J | Neisseria meningitidis | W | ST-22 complex | No value | UK | SV-12 | Opc+ |
| 35394 | 13.7552.F | Neisseria meningitidis | B | ST-41/44 complex | No value | UK | SV-2 | Opc+ |
| 35395 | 13.7896.B | Neisseria meningitidis | B | ST-18 complex | No value | UK | SV-7 | Opc- |
| 35396 | 13.8092.Q | Neisseria meningitidis | B | ST-1157 complex | No value | UK | SV-1 | Opc+ |
| 35397 | 13.8552.X | Neisseria meningitidis | W | ST-11 complex | No value | UK | SV-2 | Opc- |
| 35404 | M07 240834 | Neisseria meningitidis | B | ST-269 complex | No value | UK | SV-1 | Opc+ |
| 35405 | M07 241002 | Neisseria meningitidis | B | ST-269 complex | No value | UK | SV-1 | Opc+ |
| 35406 | M07 241143 | Neisseria meningitidis | B | ST-269 complex | No value | UK | SV-1 | Opc+ |
| 35407 | M08 240183 | Neisseria meningitidis | B | ST-269 complex | No value | UK | SV-1 | Opc+ |
| 35408 | M08 240430 | Neisseria meningitidis | B | ST-269 complex | No value | UK | SV-1 | Opc+ |
| 35409 | M08 240363 | Neisseria meningitidis | B | No value | No value | UK | SV-1 | Opc+ |
| 35410 | M07 240836 | Neisseria meningitidis | B | No value | No value | UK | SV-8 | Opc+ |
| 35411 | M07 240904 | Neisseria meningitidis | B | ST-269 complex | No value | UK | SV-8 | Opc+ |
| 35412 | M07 240980 | Neisseria meningitidis | B | ST-269 complex | No value | UK | SV-8 | Opc+ |
| 35413 | M08 240017 | Neisseria meningitidis | B | ST-269 complex | No value | UK | SV-8 | Opc+ |
| 35414 | M08 240056 | Neisseria meningitidis | B | ST-269 complex | No value | UK | SV-8 | Opc+ |
| 35415 | M08 240234 | Neisseria meningitidis | B | ST-269 complex | No value | UK | SV-8 | Opc+ |
| 35416 | M03 240924 | Neisseria meningitidis | NG | No value | No value | Burkina Faso | SV-1 | Opc- |
| 35417 | M03 240926 | Neisseria meningitidis | NG | No value | No value | Burkina Faso | SV-1 | Opc- |
| 35418 | M04 241084 | Neisseria meningitidis | NG | No value | No value | Burkina Faso | SV-1 | Opc- |
| 35419 | M04 241036 | Neisseria meningitidis | NG | No value | No value | Burkina Faso | SV-1 | Opc- |

| | | | | | | | | |
|---|---|---|---|---|---|---|---|---|
| 35420 | M14 240366 | Neisseria meningitidis | W | No value | No value | UK | SV-12 | Opc+ |
| 35421 | M13 240557 | Neisseria meningitidis | B | No value | No value | UK | SV-12 | Opc- |
| 35422 | M13 240526 | Neisseria meningitidis | B | No value | No value | UK | SV-2 | Opc+ |
| 35423 | M13 240528 | Neisseria meningitidis | W | ST-22 complex | No value | UK | SV-12 | Opc+ |
| 35424 | M13 240529 | Neisseria meningitidis | Y | ST-23 complex | No value | UK | SV-11 | Opc+ |
| 35425 | M13 240531 | Neisseria meningitidis | W | ST-11 complex | No value | UK | SV-2 | Opc- |
| 35426 | M13 240532 | Neisseria meningitidis | B | ST-41/44 complex | No value | UK | SV-2 | Opc+ |
| 35427 | M13 240534 | Neisseria meningitidis | B | ST-461 complex | No value | UK | SV-1 | Opc- |
| 35428 | M13 240535 | Neisseria meningitidis | B | ST-32 complex | No value | UK | SV-1 | Opc+ |
| 35429 | M13 240536 | Neisseria meningitidis | B | ST-269 complex | No value | UK | SV-8 | Opc+ |
| 35430 | M13 240540 | Neisseria meningitidis | B | ST-269 complex | No value | UK | SV-8 | Opc+ |
| 35431 | M13 240542 | Neisseria meningitidis | B | ST-213 complex | No value | UK | SV-12 | Opc- |
| 35432 | M13 240543 | Neisseria meningitidis | Y | ST-23 complex | No value | UK | SV-11 | Opc+ |
| 35433 | M13 240546 | Neisseria meningitidis | B | ST-213 complex | No value | UK | SV-12 | Opc- |
| 35434 | M13 240548 | Neisseria meningitidis | B | ST-41/44 complex | No value | UK | SV-2 | Opc+ |
| 35435 | M13 240549 | Neisseria meningitidis | Y | ST-23 complex | No value | UK | SV-11 | Opc+ |
| 35436 | M13 240554 | Neisseria meningitidis | B | ST-461 complex | No value | UK | SV-1 | Opc- |
| 35437 | M13 240560 | Neisseria meningitidis | B | ST-41/44 complex | No value | UK | SV-14 | Opc+ |
| 35438 | M13 240561 | Neisseria meningitidis | B | ST-41/44 complex | No value | UK | SV-2 | Opc+ |
| 35439 | M13 240562 | Neisseria meningitidis | B | ST-269 complex | No value | UK | SV-8 | Opc+ |
| 35440 | M13 240564 | Neisseria meningitidis | B | ST-41/44 complex | No value | UK | SV-2 | Opc+ |
| 35441 | M13 240565 | Neisseria meningitidis | B | ST-269 complex | No value | UK | SV-1 | Opc+ |
| 35442 | M13 240566 | Neisseria meningitidis | B | ST-213 complex | No value | UK | SV-11 | Opc- |
| 35443 | M13 240568 | Neisseria meningitidis | B | ST-269 complex | No value | UK | SV-1 | Opc+ |
| 35444 | M13 240572 | Neisseria meningitidis | B | ST-269 complex | No value | UK | SV-1 | Opc+ |
| 35445 | M13 240573 | Neisseria meningitidis | Y | ST-23 complex | No value | UK | SV-11 | Opc+ |
| 35446 | M13 240574 | Neisseria meningitidis | Y | ST-23 complex | No value | UK | SV-11 | Opc+ |
| 35447 | M13 240579 | Neisseria meningitidis | B | ST-269 complex | No value | UK | SV-1 | Opc+ |
| 35448 | M13 240581 | Neisseria meningitidis | B | ST-461 complex | No value | UK | SV-1 | Opc- |
| 35449 | M13 240588 | Neisseria meningitidis | Y | ST-167 complex | No value | UK | SV-5 | Opc+ |
| 35450 | M13 240590 | Neisseria meningitidis | Y | No value | No value | UK | SV-11 | Opc+ |
| 35451 | M13 240592 | Neisseria meningitidis | B | ST-269 complex | No value | UK | SV-8 | Opc+ |
| 35452 | M13 240593 | Neisseria meningitidis | B | ST-162 complex | No value | UK | SV-1 | Opc+ |
| 35453 | M13 240595 | Neisseria meningitidis | W | ST-22 complex | No value | UK | SV-12 | Opc+ |
| 35454 | M13 240596 | Neisseria meningitidis | B | ST-269 complex | No value | UK | SV-1 | Opc+ |
| 35455 | M13 240598 | Neisseria meningitidis | Y | ST-41/44 complex | No value | UK | SV-2 | Opc+ |
| 35456 | M13 240599 | Neisseria meningitidis | Y | ST-23 complex | No value | UK | SV-11 | Opc+ |
| 35457 | M13 240601 | Neisseria meningitidis | B | ST-41/44 complex | No value | UK | SV-2 | Opc+ |
| 35458 | M13 240605 | Neisseria meningitidis | B | No value | No value | UK | SV-8 | Opc+ |
| 35459 | M13 240608 | Neisseria meningitidis | B | ST-41/44 complex | No value | UK | SV-10 | Opc+ |
| 35461 | M13 240611 | Neisseria meningitidis | B | ST-269 complex | No value | UK | SV-8 | Opc+ |
| 35462 | M13 240613 | Neisseria meningitidis | B | ST-41/44 complex | No value | UK | SV-2 | Opc+ |
| 35463 | M13 240614 | Neisseria meningitidis | B | ST-41/44 complex | No value | UK | SV-5 | Opc+ |
| 35464 | M13 240615 | Neisseria meningitidis | B | No value | No value | UK | SV-2 | Opc+ |
| 35465 | M13 240618 | Neisseria meningitidis | B | No value | No value | UK | SV-1 | Opc+ |
| 35466 | M13 240619 | Neisseria meningitidis | B | ST-213 complex | No value | UK | SV-12 | Opc- |
| 35467 | M13 240620 | Neisseria meningitidis | B | ST-41/44 complex | No value | UK | SV-2 | Opc+ |
| 35468 | M13 240622 | Neisseria meningitidis | Y | ST-23 complex | No value | UK | SV-11 | Opc+ |
| 35469 | M13 240623 | Neisseria meningitidis | B | ST-35 complex | No value | UK | SV-12 | Opc+ |
| 35470 | M13 240624 | Neisseria meningitidis | B | ST-41/44 complex | No value | UK | SV-2 | Opc+ |
| 35471 | M13 240626 | Neisseria meningitidis | B | ST-41/44 complex | No value | UK | SV-2 | Opc+ |
| 35472 | M13 240627 | Neisseria meningitidis | Y | ST-167 complex | No value | UK | SV-5 | Opc+ |
| 35473 | M13 240632 | Neisseria meningitidis | B | No value | No value | UK | SV-12 | Opc- |
| 35474 | M13 240636 | Neisseria meningitidis | B | ST-41/44 complex | No value | UK | SV-2 | Opc+ |
| 35475 | M13 240638 | Neisseria meningitidis | B | No value | No value | UK | SV-8 | Opc+ |
| 35476 | M13 240639 | Neisseria meningitidis | B | No value | No value | UK | SV-12 | Opc- |
| 35477 | M13 240640 | Neisseria meningitidis | B | ST-269 complex | No value | UK | SV-8 | Opc+ |
| 35478 | M13 240641 | Neisseria meningitidis | B | ST-41/44 complex | No value | UK | SV-2 | Opc+ |
| 35479 | M13 240642 | Neisseria meningitidis | B | ST-213 complex | No value | UK | SV-12 | Opc- |
| 35480 | M13 240644 | Neisseria meningitidis | B | ST-461 complex | No value | UK | SV-1 | Opc- |
| 35481 | M13 240646 | Neisseria meningitidis | B | No value | No value | UK | SV-8 | Opc+ |
| 35482 | M13 240647 | Neisseria meningitidis | B | ST-41/44 complex | No value | UK | SV-2 | Opc+ |
| 35483 | M13 240648 | Neisseria meningitidis | B | ST-41/44 complex | No value | UK | SV-2 | Opc+ |
| 35484 | M13 240652 | Neisseria meningitidis | Y | ST-23 complex | No value | UK | SV-11 | Opc+ |
| 35485 | M13 240658 | Neisseria meningitidis | B | ST-269 complex | No value | UK | SV-8 | Opc+ |
| 35486 | M13 240662 | Neisseria meningitidis | B | No value | No value | UK | SV-1 | Opc- |
| 35487 | M13 240663 | Neisseria meningitidis | Y | ST-23 complex | No value | UK | SV-11 | Opc+ |
| 35488 | M13 240665 | Neisseria meningitidis | B | ST-213 complex | No value | UK | SV-12 | Opc- |
| 35489 | M13 240670 | Neisseria meningitidis | B | ST-41/44 complex | No value | UK | SV-2 | Opc+ |
| 35490 | M13 240671 | Neisseria meningitidis | B | ST-461 complex | No value | UK | SV-1 | Opc- |
| 35491 | M13 240673 | Neisseria meningitidis | Y | ST-23 complex | No value | UK | SV-11 | Opc+ |
| 35492 | M13 240675 | Neisseria meningitidis | B | ST-41/44 complex | No value | UK | SV-6 | Opc+ |
| 35493 | M13 240677 | Neisseria meningitidis | B | No value | No value | UK | SV-10 | Opc+ |
| 35494 | M13 240684 | Neisseria meningitidis | B | ST-269 complex | No value | UK | SV-1 | Opc+ |

| | | | | | | | | |
|---|---|---|---|---|---|---|---|---|
| 35495 | M13 240686 | Neisseria meningitidis | B | ST-269 complex | No value | UK | SV-8 | Opc+ |
| 35496 | M13 240690 | Neisseria meningitidis | B | No value | No value | UK | SV-2 | Opc+ |
| 35497 | M13 240692 | Neisseria meningitidis | B | ST-41/44 complex | No value | UK | SV-2 | Opc+ |
| 35498 | M13 240693 | Neisseria meningitidis | B | ST-32 complex | No value | UK | SV-1 | Opc+ |
| 35499 | M13 240694 | Neisseria meningitidis | Y | No value | No value | UK | SV-11 | Opc+ |
| 35500 | M13 240695 | Neisseria meningitidis | B | ST-11 complex | No value | UK | SV-2 | Opc- |
| 35501 | M13 240696 | Neisseria meningitidis | Y | ST-167 complex | No value | UK | SV-5 | Opc+ |
| 35502 | M13 240697 | Neisseria meningitidis | B | ST-11 complex | No value | UK | SV-2 | Opc- |
| 35503 | M13 240698 | Neisseria meningitidis | B | ST-41/44 complex | No value | UK | SV-2 | Opc+ |
| 35504 | M13 240699 | Neisseria meningitidis | B | ST-41/44 complex | No value | UK | SV-2 | Opc+ |
| 35505 | M13 240702 | Neisseria meningitidis | B | ST-35 complex | No value | UK | SV-12 | Opc+ |
| 35506 | M13 240703 | Neisseria meningitidis | B | ST-269 complex | No value | UK | SV-1 | Opc+ |
| 35507 | M13 240705 | Neisseria meningitidis | W | ST-11 complex | No value | UK | SV-2 | Opc- |
| 35508 | M13 240706 | Neisseria meningitidis | Y | ST-23 complex | No value | UK | SV-11 | Opc+ |
| 35509 | M13 240708 | Neisseria meningitidis | B | ST-41/44 complex | No value | UK | SV-6 | Opc+ |
| 35510 | M13 240709 | Neisseria meningitidis | B | ST-41/44 complex | No value | UK | SV-5 | Opc- |
| 35511 | M13 240710 | Neisseria meningitidis | B | ST-60 complex | No value | UK | SV-6 | Opc+ |
| 35512 | M13 240715 | Neisseria meningitidis | B | ST-213 complex | No value | UK | SV-12 | Opc- |
| 35513 | M13 240716 | Neisseria meningitidis | Y | ST-23 complex | No value | UK | SV-11 | Opc+ |
| 35514 | M13 240717 | Neisseria meningitidis | B | ST-213 complex | No value | UK | SV-12 | Opc- |
| 35515 | M13 240718 | Neisseria meningitidis | B | ST-269 complex | No value | UK | SV-1 | Opc+ |
| 35516 | M13 240719 | Neisseria meningitidis | B | ST-213 complex | No value | UK | SV-12 | Opc- |
| 35517 | M13 240720 | Neisseria meningitidis | Y | ST-22 complex | No value | UK | SV-12 | Opc+ |
| 35518 | M13 240721 | Neisseria meningitidis | Y | ST-23 complex | No value | UK | SV-11 | Opc+ |
| 35519 | M13 240723 | Neisseria meningitidis | B | ST-32 complex | No value | UK | SV-1 | Opc+ |
| 35520 | M13 240725 | Neisseria meningitidis | Y | ST-23 complex | No value | UK | SV-11 | Opc+ |
| 35521 | M13 240729 | Neisseria meningitidis | B | ST-213 complex | No value | UK | SV-12 | Opc- |
| 35522 | M13 240731 | Neisseria meningitidis | B | ST-213 complex | No value | UK | SV-12 | Opc- |
| 35523 | M13 240733 | Neisseria meningitidis | Y | ST-167 complex | No value | UK | SV-5 | Opc+ |
| 35524 | M13 240734 | Neisseria meningitidis | C | ST-60 complex | No value | UK | SV-11 | Opc+ |
| 35525 | M13 240737 | Neisseria meningitidis | B | ST-269 complex | No value | UK | SV-8 | Opc+ |
| 35526 | M13 240738 | Neisseria meningitidis | B | ST-41/44 complex | No value | UK | SV-2 | Opc+ |
| 35527 | M13 240739 | Neisseria meningitidis | B | ST-213 complex | No value | UK | SV-12 | Opc- |
| 35528 | M13 240740 | Neisseria meningitidis | B | ST-103 complex | No value | UK | SV-3 | Opc+ |
| 35529 | M13 240742 | Neisseria meningitidis | B | No value | No value | UK | SV-10 | Opc+ |
| 35530 | M13 240743 | Neisseria meningitidis | B | No value | No value | UK | SV-5 | Opc+ |
| 35531 | M13 240744 | Neisseria meningitidis | Y | No value | No value | UK | SV-11 | Opc+ |
| 35532 | M13 240745 | Neisseria meningitidis | B | ST-41/44 complex | No value | UK | SV-2 | Opc+ |
| 35533 | M13 240746 | Neisseria meningitidis | B | ST-41/44 complex | No value | UK | SV-2 | Opc+ |
| 35534 | M13 240748 | Neisseria meningitidis | B | ST-269 complex | No value | UK | SV-8 | Opc+ |
| 35535 | M13 240749 | Neisseria meningitidis | Y | ST-23 complex | No value | UK | SV-11 | Opc+ |
| 35536 | M13 240750 | Neisseria meningitidis | Y | ST-23 complex | No value | UK | SV-11 | Opc+ |
| 35537 | M13 240751 | Neisseria meningitidis | Y | ST-23 complex | No value | UK | SV-11 | Opc+ |
| 35538 | M13 240752 | Neisseria meningitidis | Y | ST-23 complex | No value | UK | SV-11 | Opc+ |
| 35539 | M13 240753 | Neisseria meningitidis | B | ST-41/44 complex | No value | UK | SV-2 | Opc+ |
| 35540 | M13 240755 | Neisseria meningitidis | B | ST-213 complex | No value | UK | SV-12 | Opc- |
| 35541 | M14 240003 | Neisseria meningitidis | B | ST-41/44 complex | No value | UK | SV-10 | Opc+ |
| 35542 | M14 240008 | Neisseria meningitidis | B | ST-41/44 complex | No value | UK | SV-6 | Opc+ |
| 35543 | M14 240010 | Neisseria meningitidis | Y | ST-23 complex | No value | UK | SV-11 | Opc+ |
| 35544 | M14 240011 | Neisseria meningitidis | B | ST-269 complex | No value | UK | SV-8 | Opc+ |
| 35545 | M14 240012 | Neisseria meningitidis | Y | ST-23 complex | No value | UK | SV-11 | Opc+ |
| 35546 | M14 240014 | Neisseria meningitidis | B | ST-32 complex | No value | UK | SV-1 | Opc+ |
| 35547 | M14 240015 | Neisseria meningitidis | Y | ST-167 complex | No value | UK | SV-5 | Opc+ |
| 35548 | M14 240016 | Neisseria meningitidis | B | ST-41/44 complex | No value | UK | SV-6 | Opc+ |
| 35549 | M14 240017 | Neisseria meningitidis | B | ST-41/44 complex | No value | UK | SV-2 | Opc+ |
| 35550 | M14 240018 | Neisseria meningitidis | B | ST-41/44 complex | No value | UK | SV-6 | Opc+ |
| 35551 | M14 240020 | Neisseria meningitidis | C | ST-103 complex | No value | UK | SV-5 | Opc+ |
| 35552 | M14 240021 | Neisseria meningitidis | B | ST-41/44 complex | No value | UK | SV-2 | Opc+ |
| 35553 | M14 240023 | Neisseria meningitidis | Y | No value | No value | UK | SV-11 | Opc+ |
| 35555 | M14 240025 | Neisseria meningitidis | B | ST-269 complex | No value | UK | SV-8 | Opc+ |
| 35556 | M14 240027 | Neisseria meningitidis | B | ST-41/44 complex | No value | UK | SV-6 | Opc+ |
| 35557 | M14 240028 | Neisseria meningitidis | B | ST-32 complex | No value | UK | SV-1 | Opc+ |
| 35558 | M14 240030 | Neisseria meningitidis | B | ST-213 complex | No value | UK | SV-12 | Opc- |
| 35559 | M14 240037 | Neisseria meningitidis | B | ST-269 complex | No value | UK | SV-8 | Opc+ |
| 35560 | M14 240038 | Neisseria meningitidis | W | ST-22 complex | No value | UK | SV-12 | Opc+ |
| 35561 | M14 240039 | Neisseria meningitidis | B | No value | No value | UK | SV-2 | Opc+ |
| 35562 | M14 240045 | Neisseria meningitidis | B | ST-41/44 complex | No value | UK | SV-2 | Opc+ |
| 35563 | M14 240046 | Neisseria meningitidis | B | ST-41/44 complex | No value | UK | SV-2 | Opc+ |
| 35564 | M14 240048 | Neisseria meningitidis | B | ST-32 complex | No value | UK | SV-1 | Opc+ |
| 35565 | M14 240049 | Neisseria meningitidis | Y | ST-23 complex | No value | UK | SV-11 | Opc+ |
| 35566 | M14 240055 | Neisseria meningitidis | B | ST-41/44 complex | No value | UK | SV-2 | Opc+ |
| 35567 | M14 240056 | Neisseria meningitidis | Y | ST-23 complex | No value | UK | SV-11 | Opc+ |
| 35568 | M14 240059 | Neisseria meningitidis | B | ST-269 complex | No value | UK | SV-8 | Opc+ |
| 35569 | M14 240060 | Neisseria meningitidis | Y | ST-23 complex | No value | UK | SV-11 | Opc+ |

| | | | | | | | | |
|---|---|---|---|---|---|---|---|---|
| 35570 | M14 240061 | Neisseria meningitidis | B | ST-269 complex | No value | UK | SV-1 | Opc+ |
| 35571 | M14 240062 | Neisseria meningitidis | B | ST-32 complex | No value | UK | SV-1 | Opc+ |
| 35572 | M14 240073 | Neisseria meningitidis | B | ST-269 complex | No value | UK | SV-8 | Opc+ |
| 35573 | M14 240074 | Neisseria meningitidis | B | ST-41/44 complex | No value | UK | SV-6 | Opc+ |
| 35574 | M14 240075 | Neisseria meningitidis | Y | ST-23 complex | No value | UK | SV-11 | Opc+ |
| 35575 | M14 240077 | Neisseria meningitidis | B | ST-32 complex | No value | UK | SV-1 | Opc+ |
| 35576 | M14 240078 | Neisseria meningitidis | B | ST-213 complex | No value | UK | SV-12 | Opc- |
| 35579 | M14 240087 | Neisseria meningitidis | B | ST-213 complex | No value | UK | SV-12 | Opc- |
| 35580 | M14 240088 | Neisseria meningitidis | B | ST-461 complex | No value | UK | SV-1 | Opc- |
| 35581 | M14 240090 | Neisseria meningitidis | W | ST-11 complex | No value | UK | SV-2 | Opc- |
| 35582 | M14 240091 | Neisseria meningitidis | Y | ST-23 complex | No value | UK | SV-11 | Opc+ |
| 35583 | M14 240093 | Neisseria meningitidis | B | ST-18 complex | No value | UK | SV-1 | Opc- |
| 35585 | M14 240096 | Neisseria meningitidis | B | ST-41/44 complex | No value | UK | SV-6 | Opc+ |
| 35586 | M14 240098 | Neisseria meningitidis | B | ST-41/44 complex | No value | UK | SV-2 | Opc+ |
| 35587 | M14 240099 | Neisseria meningitidis | B | ST-269 complex | No value | UK | SV-8 | Opc+ |
| 35588 | M14 240101 | Neisseria meningitidis | B | ST-32 complex | No value | UK | SV-1 | Opc+ |
| 35589 | M14 240103 | Neisseria meningitidis | B | ST-32 complex | No value | UK | SV-1 | Opc+ |
| 35592 | M14 240108 | Neisseria meningitidis | Y | ST-23 complex | No value | UK | SV-11 | Opc+ |
| 35593 | M14 240109 | Neisseria meningitidis | C | ST-269 complex | No value | UK | SV-1 | Opc+ |
| 35594 | M14 240110 | Neisseria meningitidis | B | ST-41/44 complex | No value | UK | SV-6 | Opc+ |
| 35595 | M14 240111 | Neisseria meningitidis | Y | ST-23 complex | No value | UK | SV-11 | Opc+ |
| 35596 | M14 240112 | Neisseria meningitidis | Y | ST-23 complex | No value | UK | SV-11 | Opc+ |
| 35598 | M14 240116 | Neisseria meningitidis | W | ST-11 complex | No value | UK | SV-2 | Opc- |
| 35599 | M14 240117 | Neisseria meningitidis | Y | ST-23 complex | No value | UK | SV-11 | Opc+ |
| 35600 | M14 240118 | Neisseria meningitidis | Y | ST-23 complex | No value | UK | SV-11 | Opc+ |
| 35601 | M14 240119 | Neisseria meningitidis | B | ST-282 complex | No value | UK | SV-9 | Opc+ |
| 35602 | M14 240120 | Neisseria meningitidis | Y | No value | No value | UK | SV-5 | Opc+ |
| 35603 | M14 240121 | Neisseria meningitidis | B | ST-213 complex | No value | UK | SV-12 | Opc- |
| 35604 | M14 240122 | Neisseria meningitidis | B | ST-32 complex | No value | UK | SV-1 | Opc+ |
| 35605 | M14 240123 | Neisseria meningitidis | B | ST-60 complex | No value | UK | SV-11 | Opc+ |
| 35606 | M14 240124 | Neisseria meningitidis | B | ST-41/44 complex | No value | UK | SV-2 | Opc+ |
| 35609 | M14 240127 | Neisseria meningitidis | B | ST-41/44 complex | No value | UK | SV-6 | Opc+ |
| 35610 | M14 240129 | Neisseria meningitidis | W | ST-22 complex | No value | UK | SV-7 | Opc+ |
| 35611 | M14 240130 | Neisseria meningitidis | Y | No value | No value | UK | SV-11 | Opc+ |
| 35612 | M14 240133 | Neisseria meningitidis | B | ST-461 complex | No value | UK | SV-1 | Opc- |
| 35613 | M14 240134 | Neisseria meningitidis | B | ST-41/44 complex | No value | UK | SV-6 | Opc+ |
| 35614 | M14 240135 | Neisseria meningitidis | W | ST-11 complex | No value | UK | SV-2 | Opc- |
| 35615 | M14 240136 | Neisseria meningitidis | Y | ST-23 complex | No value | UK | SV-11 | Opc+ |
| 35616 | M14 240140 | Neisseria meningitidis | Y | ST-23 complex | No value | UK | SV-11 | Opc+ |
| 35617 | M14 240141 | Neisseria meningitidis | B | No value | No value | UK | SV-2 | Opc+ |
| 35618 | M14 240142 | Neisseria meningitidis | W | ST-11 complex | No value | UK | SV-2 | Opc- |
| 35619 | M14 240143 | Neisseria meningitidis | B | ST-32 complex | No value | UK | SV-1 | Opc+ |
| 35620 | M14 240144 | Neisseria meningitidis | Y | ST-23 complex | No value | UK | SV-11 | Opc+ |
| 35621 | M14 240145 | Neisseria meningitidis | W | ST-11 complex | No value | UK | SV-2 | Opc- |
| 35622 | M14 240147 | Neisseria meningitidis | W | ST-11 complex | No value | UK | SV-2 | Opc- |
| 35623 | M14 240149 | Neisseria meningitidis | B | ST-269 complex | No value | UK | SV-8 | Opc+ |
| 35624 | M14 240150 | Neisseria meningitidis | W | ST-11 complex | No value | UK | SV-1 | Opc- |
| 35625 | M14 240153 | Neisseria meningitidis | B | ST-41/44 complex | No value | UK | SV-1 | Opc+ |
| 35626 | M14 240154 | Neisseria meningitidis | B | No value | No value | UK | SV-6 | Opc+ |
| 35627 | M14 240157 | Neisseria meningitidis | Y | ST-23 complex | No value | UK | SV-11 | Opc+ |
| 35628 | M14 240168 | Neisseria meningitidis | C | ST-11 complex | No value | UK | SV-2 | Opc- |
| 35629 | M14 240174 | Neisseria meningitidis | B | ST-41/44 complex | No value | UK | SV-2 | Opc+ |
| 35630 | M14 240175 | Neisseria meningitidis | B | ST-269 complex | No value | UK | SV-1 | Opc+ |
| 35631 | M14 240177 | Neisseria meningitidis | B | ST-269 complex | No value | UK | SV-8 | Opc+ |
| 35632 | M14 240178 | Neisseria meningitidis | W | ST-11 complex | No value | UK | SV-2 | Opc- |
| 35633 | M14 240179 | Neisseria meningitidis | B | ST-269 complex | No value | UK | SV-1 | Opc+ |
| 35634 | M14 240180 | Neisseria meningitidis | W | ST-11 complex | No value | UK | SV-2 | Opc- |
| 35635 | M14 240199 | Neisseria meningitidis | W | ST-11 complex | No value | UK | SV-2 | Opc- |
| 35636 | M14 240201 | Neisseria meningitidis | B | ST-269 complex | No value | UK | SV-1 | Opc+ |
| 35637 | M14 240202 | Neisseria meningitidis | Y | ST-23 complex | No value | UK | SV-11 | Opc+ |
| 35638 | M14 240204 | Neisseria meningitidis | W | ST-11 complex | No value | UK | SV-2 | Opc- |
| 35639 | M14 240205 | Neisseria meningitidis | B | ST-213 complex | No value | UK | SV-12 | Opc- |
| 35640 | M14 240206 | Neisseria meningitidis | B | ST-269 complex | No value | UK | SV-8 | Opc+ |
| 35641 | M14 240208 | Neisseria meningitidis | NG | ST-1157 complex | No value | UK | SV-1 | Opc+ |
| 35642 | M14 240210 | Neisseria meningitidis | Y | ST-23 complex | No value | UK | SV-11 | Opc+ |
| 35643 | M14 240211 | Neisseria meningitidis | W | ST-11 complex | No value | UK | SV-2 | Opc- |
| 35644 | M14 240213 | Neisseria meningitidis | B | No value | No value | UK | SV-1 | Opc+ |
| 35645 | M14 240215 | Neisseria meningitidis | B | ST-213 complex | No value | UK | SV-12 | Opc- |
| 35646 | M14 240217 | Neisseria meningitidis | B | ST-41/44 complex | No value | UK | SV-10 | Opc+ |
| 35647 | M14 240218 | Neisseria meningitidis | B | ST-32 complex | No value | UK | SV-1 | Opc+ |
| 35648 | M14 240220 | Neisseria meningitidis | Y | No value | No value | UK | SV-11 | Opc+ |
| 35649 | M14 240221 | Neisseria meningitidis | Y | No value | No value | UK | SV-11 | Opc+ |
| 35650 | M14 240222 | Neisseria meningitidis | C | ST-11 complex | No value | UK | SV-2 | Opc- |
| 35651 | M14 240223 | Neisseria meningitidis | W | ST-11 complex | No value | UK | SV-2 | Opc- |

| | | | | | | | | |
|---|---|---|---|---|---|---|---|---|
| 35652 | M14 240224 | Neisseria meningitidis | C | No value | No value | UK | SV-2 | Opc+ |
| 35653 | M14 240225 | Neisseria meningitidis | Y | ST-23 complex | No value | UK | SV-11 | Opc+ |
| 35654 | M14 240226 | Neisseria meningitidis | Y | No value | No value | UK | SV-11 | Opc+ |
| 35655 | M14 240227 | Neisseria meningitidis | B | ST-32 complex | No value | UK | SV-1 | Opc+ |
| 35656 | M14 240232 | Neisseria meningitidis | B | ST-41/44 complex | No value | UK | SV-10 | Opc+ |
| 35657 | M14 240233 | Neisseria meningitidis | B | ST-41/44 complex | No value | UK | SV-10 | Opc+ |
| 35658 | M14 240234 | Neisseria meningitidis | B | ST-269 complex | No value | UK | SV-1 | Opc+ |
| 35659 | M14 240236 | Neisseria meningitidis | C | ST-11 complex | No value | UK | SV-2 | Opc- |
| 35660 | M14 240237 | Neisseria meningitidis | Y | ST-23 complex | No value | UK | SV-11 | Opc+ |
| 35661 | M14 240238 | Neisseria meningitidis | W | ST-22 complex | No value | UK | SV-12 | Opc+ |
| 35662 | M14 240241 | Neisseria meningitidis | B | ST-269 complex | No value | UK | SV-8 | Opc+ |
| 35663 | M14 240242 | Neisseria meningitidis | B | ST-162 complex | No value | UK | SV-1 | Opc+ |
| 35664 | M14 240245 | Neisseria meningitidis | W | ST-11 complex | No value | UK | SV-2 | Opc- |
| 35665 | M14 240246 | Neisseria meningitidis | Y | ST-23 complex | No value | UK | SV-11 | Opc+ |
| 35666 | M14 240248 | Neisseria meningitidis | B | ST-32 complex | No value | UK | SV-1 | Opc+ |
| 35667 | M14 240249 | Neisseria meningitidis | B | ST-11 complex | No value | UK | SV-2 | Opc- |
| 35668 | M14 240251 | Neisseria meningitidis | B | ST-162 complex | No value | UK | SV-1 | Opc+ |
| 35669 | M14 240252 | Neisseria meningitidis | B | ST-269 complex | No value | UK | SV-8 | Opc+ |
| 35670 | M14 240253 | Neisseria meningitidis | W | ST-11 complex | No value | UK | SV-2 | Opc- |
| 35671 | M14 240254 | Neisseria meningitidis | B | No value | No value | UK | SV-6 | Opc+ |
| 35672 | M14 240255 | Neisseria meningitidis | C | ST-11 complex | No value | UK | SV-2 | Opc- |
| 35673 | M14 240256 | Neisseria meningitidis | B | ST-41/44 complex | No value | UK | SV-2 | Opc+ |
| 35674 | M14 240257 | Neisseria meningitidis | W | ST-11 complex | No value | UK | SV-2 | Opc- |
| 35675 | M14 240258 | Neisseria meningitidis | W | ST-11 complex | No value | UK | SV-2 | Opc- |
| 35676 | M14 240259 | Neisseria meningitidis | B | ST-162 complex | No value | UK | SV-1 | Opc+ |
| 35677 | M14 240261 | Neisseria meningitidis | B | ST-269 complex | No value | UK | SV-8 | Opc+ |
| 35678 | M14 240264 | Neisseria meningitidis | B | No value | No value | UK | SV-12 | Opc- |
| 35679 | M14 240265 | Neisseria meningitidis | B | ST-269 complex | No value | UK | SV-1 | Opc+ |
| 35680 | M14 240266 | Neisseria meningitidis | B | ST-269 complex | No value | UK | SV-8 | Opc+ |
| 35681 | M14 240267 | Neisseria meningitidis | B | ST-41/44 complex | No value | UK | SV-2 | Opc+ |
| 35682 | M14 240271 | Neisseria meningitidis | B | ST-32 complex | No value | UK | SV-1 | Opc+ |
| 35683 | M14 240273 | Neisseria meningitidis | B | ST-32 complex | No value | UK | SV-1 | Opc+ |
| 35684 | M14 240276 | Neisseria meningitidis | Y | ST-23 complex | No value | UK | SV-11 | Opc+ |
| 35685 | M14 240277 | Neisseria meningitidis | Y | ST-167 complex | No value | UK | SV-5 | Opc+ |
| 35686 | M14 240278 | Neisseria meningitidis | W | ST-11 complex | No value | UK | SV-2 | Opc- |
| 35687 | M14 240279 | Neisseria meningitidis | W | ST-11 complex | No value | UK | SV-2 | Opc- |
| 35688 | M14 240280 | Neisseria meningitidis | C | ST-11 complex | No value | UK | SV-2 | Opc- |
| 35689 | M14 240282 | Neisseria meningitidis | W | ST-11 complex | No value | UK | SV-2 | Opc- |
| 35690 | M14 240283 | Neisseria meningitidis | W | ST-11 complex | No value | UK | SV-2 | Opc- |
| 35691 | M14 240284 | Neisseria meningitidis | Y | No value | No value | UK | SV-11 | Opc+ |
| 35692 | M14 240285 | Neisseria meningitidis | Y | ST-23 complex | No value | UK | SV-11 | Opc+ |
| 35693 | M14 240286 | Neisseria meningitidis | C | ST-11 complex | No value | UK | SV-2 | Opc- |
| 35694 | M14 240287 | Neisseria meningitidis | Y | ST-23 complex | No value | UK | SV-11 | Opc+ |
| 35695 | M14 240289 | Neisseria meningitidis | B | No value | No value | UK | SV-6 | Opc+ |
| 35696 | M14 240291 | Neisseria meningitidis | Y | ST-23 complex | No value | UK | SV-11 | Opc+ |
| 35697 | M14 240293 | Neisseria meningitidis | Y | ST-22 complex | No value | UK | SV-1 | Opc+ |
| 35698 | M14 240295 | Neisseria meningitidis | B | ST-60 complex | No value | UK | SV-11 | Opc+ |
| 35699 | M14 240296 | Neisseria meningitidis | B | ST-32 complex | No value | UK | SV-1 | Opc+ |
| 35700 | M14 240299 | Neisseria meningitidis | W | ST-11 complex | No value | UK | SV-2 | Opc- |
| 35701 | M14 240300 | Neisseria meningitidis | B | ST-269 complex | No value | UK | SV-8 | Opc+ |
| 35702 | M14 240304 | Neisseria meningitidis | W | ST-11 complex | No value | UK | SV-2 | Opc- |
| 35703 | M14 240305 | Neisseria meningitidis | B | ST-213 complex | No value | UK | SV-12 | Opc- |
| 35704 | M14 240306 | Neisseria meningitidis | B | ST-41/44 complex | No value | UK | SV-6 | Opc+ |
| 35705 | M14 240309 | Neisseria meningitidis | B | ST-41/44 complex | No value | UK | SV-6 | Opc+ |
| 35706 | M14 240310 | Neisseria meningitidis | C | ST-11 complex | No value | UK | SV-2 | Opc- |
| 35707 | M14 240311 | Neisseria meningitidis | C | ST-334 complex | No value | UK | SV-11 | Opc- |
| 35708 | M14 240312 | Neisseria meningitidis | B | ST-41/44 complex | No value | UK | SV-6 | Opc+ |
| 35709 | M14 240313 | Neisseria meningitidis | Y | ST-23 complex | No value | UK | SV-11 | Opc+ |
| 35710 | M14 240314 | Neisseria meningitidis | B | ST-60 complex | No value | UK | SV-2 | Opc+ |
| 35712 | M14 240316 | Neisseria meningitidis | B | ST-213 complex | No value | UK | SV-17 | Opc- |
| 35713 | M14 240317 | Neisseria meningitidis | B | ST-461 complex | No value | UK | SV-1 | Opc- |
| 35714 | M14 240319 | Neisseria meningitidis | B | No value | No value | UK | SV-8 | Opc+ |
| 35715 | M14 240321 | Neisseria meningitidis | B | ST-213 complex | No value | UK | SV-12 | Opc- |
| 35716 | M14 240325 | Neisseria meningitidis | B | ST-269 complex | No value | UK | SV-8 | Opc+ |
| 35717 | M14 240327 | Neisseria meningitidis | Y | No value | No value | UK | SV-12 | Opc+ |
| 35718 | M14 240328 | Neisseria meningitidis | B | ST-213 complex | No value | UK | SV-12 | Opc- |
| 35719 | M14 240331 | Neisseria meningitidis | B | ST-41/44 complex | No value | UK | SV-2 | Opc+ |
| 35720 | M14 240333 | Neisseria meningitidis | B | No value | No value | UK | SV-11 | Opc- |
| 35721 | M14 240335 | Neisseria meningitidis | Y | ST-23 complex | No value | UK | SV-11 | Opc+ |
| 35722 | M14 240337 | Neisseria meningitidis | W | ST-11 complex | No value | UK | SV-2 | Opc- |
| 35723 | M14 240338 | Neisseria meningitidis | B | No value | No value | UK | SV-1 | Opc+ |
| 35724 | M14 240342 | Neisseria meningitidis | B | ST-32 complex | No value | UK | SV-1 | Opc+ |
| 35725 | M14 240343 | Neisseria meningitidis | W | ST-11 complex | No value | UK | SV-2 | Opc- |
| 35726 | M14 240344 | Neisseria meningitidis | B | ST-269 complex | No value | UK | SV-8 | Opc+ |

| | | | | | | | | |
|---|---|---|---|---|---|---|---|---|
| 35727 | M14 240345 | Neisseria meningitidis | B | ST-461 complex | No value | UK | SV-1 | Opc- |
| 35728 | M14 240346 | Neisseria meningitidis | W | ST-11 complex | No value | UK | SV-2 | Opc- |
| 35729 | M14 240347 | Neisseria meningitidis | B | No value | No value | UK | SV-6 | Opc+ |
| 35730 | M14 240350 | Neisseria meningitidis | B | No value | No value | UK | SV-1 | Opc+ |
| 35731 | M14 240351 | Neisseria meningitidis | W | ST-11 complex | No value | UK | SV-2 | Opc- |
| 35732 | M14 240353 | Neisseria meningitidis | B | ST-41/44 complex | No value | UK | SV-2 | Opc+ |
| 35733 | M14 240354 | Neisseria meningitidis | B | ST-213 complex | No value | UK | SV-12 | Opc- |
| 35734 | M14 240356 | Neisseria meningitidis | B | ST-461 complex | No value | UK | SV-5 | Opc- |
| 35735 | M14 240357 | Neisseria meningitidis | B | ST-41/44 complex | No value | UK | SV-6 | Opc+ |
| 35736 | M14 240359 | Neisseria meningitidis | B | No value | No value | UK | SV-2 | Opc- |
| 35737 | M14 240360 | Neisseria meningitidis | B | ST-269 complex | No value | UK | SV-8 | Opc+ |
| 35738 | M14 240361 | Neisseria meningitidis | W | No value | No value | UK | SV-12 | Opc+ |
| 35739 | M14 240365 | Neisseria meningitidis | B | ST-461 complex | No value | UK | SV-1 | Opc- |
| 35740 | M14 240367 | Neisseria meningitidis | B | ST-269 complex | No value | UK | SV-1 | Opc+ |
| 35741 | M14 240368 | Neisseria meningitidis | B | No value | No value | UK | SV-6 | Opc+ |
| 35742 | M14 240369 | Neisseria meningitidis | B | No value | No value | UK | SV-5 | Opc+ |
| 35744 | M14 240374 | Neisseria meningitidis | B | No value | No value | UK | SV-5 | Opc- |
| 35745 | M14 240377 | Neisseria meningitidis | W | No value | No value | UK | SV-2 | Opc- |
| 35746 | M14 240378 | Neisseria meningitidis | Y | ST-23 complex | No value | UK | SV-11 | Opc+ |
| 35747 | M14 240379 | Neisseria meningitidis | B | ST-213 complex | No value | UK | SV-12 | Opc- |
| 35748 | M14 240382 | Neisseria meningitidis | B | No value | No value | UK | SV-8 | Opc+ |
| 35749 | M14 240383 | Neisseria meningitidis | B | ST-32 complex | No value | UK | SV-1 | Opc+ |
| 35750 | M14 240384 | Neisseria meningitidis | B | ST-461 complex | No value | UK | SV-1 | Opc- |
| 35751 | M14 240386 | Neisseria meningitidis | B | No value | No value | UK | SV-2 | Opc+ |
| 35752 | M14 240387 | Neisseria meningitidis | B | ST-41/44 complex | No value | UK | SV-2 | Opc+ |
| 35753 | M14 240388 | Neisseria meningitidis | B | No value | No value | UK | SV-2 | Opc+ |
| 35754 | M14 240389 | Neisseria meningitidis | W | ST-11 complex | No value | UK | SV-2 | Opc- |
| 35755 | M14 240390 | Neisseria meningitidis | B | ST-269 complex | No value | UK | SV-8 | Opc+ |
| 35757 | M14 240393 | Neisseria meningitidis | B | ST-213 complex | No value | UK | SV-12 | Opc- |
| 35758 | M14 240396 | Neisseria meningitidis | B | ST-41/44 complex | No value | UK | SV-10 | Opc+ |
| 35759 | M14 240400 | Neisseria meningitidis | B | ST-41/44 complex | No value | UK | SV-2 | Opc+ |
| 35760 | M14 240403 | Neisseria meningitidis | Y | No value | No value | UK | SV-11 | Opc+ |
| 35761 | M14 240404 | Neisseria meningitidis | Y | ST-23 complex | No value | UK | SV-11 | Opc+ |
| 35762 | M14 240405 | Neisseria meningitidis | C | ST-11 complex | No value | UK | SV-2 | Opc- |
| 35763 | M14 240406 | Neisseria meningitidis | W | ST-11 complex | No value | UK | SV-2 | Opc- |
| 35764 | M14 240407 | Neisseria meningitidis | B | No value | No value | UK | SV-6 | Opc+ |
| 35765 | M14 240408 | Neisseria meningitidis | B | ST-269 complex | No value | UK | SV-1 | Opc+ |
| 35766 | M14 240410 | Neisseria meningitidis | B | ST-41/44 complex | No value | UK | SV-2 | Opc+ |
| 35767 | M14 240423 | Neisseria meningitidis | Y | ST-23 complex | No value | UK | SV-11 | Opc+ |
| 35768 | M14 240425 | Neisseria meningitidis | B | ST-41/44 complex | No value | UK | SV-2 | Opc+ |
| 35770 | M14 240428 | Neisseria meningitidis | B | ST-269 complex | No value | UK | SV-8 | Opc+ |
| 35771 | M14 240429 | Neisseria meningitidis | B | ST-41/44 complex | No value | UK | SV-2 | Opc+ |
| 35772 | M14 240431 | Neisseria meningitidis | Y | No value | No value | UK | SV-11 | Opc+ |
| 35773 | M14 240432 | Neisseria meningitidis | Y | No value | No value | UK | SV-11 | Opc+ |
| 35774 | M14 240434 | Neisseria meningitidis | B | ST-269 complex | No value | UK | SV-1 | Opc+ |
| 35775 | M14 240436 | Neisseria meningitidis | C | ST-11 complex | No value | UK | SV-2 | Opc- |
| 35776 | M14 240437 | Neisseria meningitidis | B | ST-1157 complex | No value | UK | SV-1 | Opc+ |
| 35777 | M14 240441 | Neisseria meningitidis | C | ST-116 complex | No value | UK | SV-7 | Opc+ |
| 35778 | M14 240451 | Neisseria meningitidis | B | ST-269 complex | No value | UK | SV-8 | Opc+ |
| 35779 | M14 240452 | Neisseria meningitidis | B | ST-269 complex | No value | UK | SV-1 | Opc+ |
| 35780 | M14 240455 | Neisseria meningitidis | B | ST-41/44 complex | No value | UK | SV-2 | Opc+ |
| 35781 | M14 240456 | Neisseria meningitidis | B | ST-461 complex | No value | UK | SV-1 | Opc- |
| 35782 | M14 240463 | Neisseria meningitidis | W | ST-11 complex | No value | UK | SV-2 | Opc- |
| 35783 | M14 240465 | Neisseria meningitidis | B | No value | No value | UK | SV-8 | Opc+ |
| 35784 | M14 240466 | Neisseria meningitidis | B | No value | No value | UK | SV-8 | Opc+ |
| 35785 | M14 240467 | Neisseria meningitidis | B | ST-32 complex | No value | UK | SV-1 | Opc+ |
| 35786 | M14 240468 | Neisseria meningitidis | W | ST-11 complex | No value | UK | SV-2 | Opc- |
| 35787 | M14 240469 | Neisseria meningitidis | B | ST-269 complex | No value | UK | SV-1 | Opc+ |
| 35788 | M14 240470 | Neisseria meningitidis | W | ST-11 complex | No value | UK | SV-2 | Opc- |
| 35789 | M14 240471 | Neisseria meningitidis | B | ST-41/44 complex | No value | UK | SV-6 | Opc+ |
| 35790 | M14 240472 | Neisseria meningitidis | B | ST-41/44 complex | No value | UK | SV-6 | Opc+ |
| 35791 | M14 240473 | Neisseria meningitidis | W | ST-22 complex | No value | UK | SV-12 | Opc+ |
| 35792 | M14 240474 | Neisseria meningitidis | W | ST-11 complex | No value | UK | SV-2 | Opc- |
| 35793 | M14 240475 | Neisseria meningitidis | B | ST-41/44 complex | No value | UK | SV-6 | Opc+ |
| 35794 | M14 240476 | Neisseria meningitidis | B | ST-41/44 complex | No value | UK | SV-6 | Opc+ |
| 35795 | M14 240477 | Neisseria meningitidis | B | ST-41/44 complex | No value | UK | SV-2 | Opc+ |
| 35796 | M14 240478 | Neisseria meningitidis | W | ST-11 complex | No value | UK | SV-2 | Opc- |
| 35797 | M14 240480 | Neisseria meningitidis | B | ST-32 complex | No value | UK | SV-1 | Opc+ |
| 35798 | M14 240481 | Neisseria meningitidis | B | ST-162 complex | No value | UK | SV-1 | Opc+ |
| 35799 | M14 240482 | Neisseria meningitidis | W | ST-11 complex | No value | UK | SV-2 | Opc- |
| 35800 | M14 240485 | Neisseria meningitidis | W | ST-11 complex | No value | UK | SV-2 | Opc- |
| 35801 | M14 240486 | Neisseria meningitidis | W | ST-11 complex | No value | UK | SV-2 | Opc- |
| 35802 | M14 240487 | Neisseria meningitidis | Y | ST-23 complex | No value | UK | SV-11 | Opc+ |
| 35803 | M14 240488 | Neisseria meningitidis | B | ST-41/44 complex | No value | UK | SV-10 | Opc+ |

| | | | | | | | | |
|---|---|---|---|---|---|---|---|---|
| 35804 | M14 240489 | Neisseria meningitidis | Y | ST-23 complex | No value | UK | SV-11 | Opc+ |
| 35805 | M14 240490 | Neisseria meningitidis | B | ST-41/44 complex | No value | UK | SV-6 | Opc+ |
| 35806 | M14 240491 | Neisseria meningitidis | W | ST-11 complex | No value | UK | SV-2 | Opc- |
| 35807 | M14 240492 | Neisseria meningitidis | W | ST-11 complex | No value | UK | SV-2 | Opc- |
| 35808 | M14 240494 | Neisseria meningitidis | W | ST-11 complex | No value | UK | SV-2 | Opc- |
| 35809 | M14 240495 | Neisseria meningitidis | W | ST-11 complex | No value | UK | SV-2 | Opc- |
| 35810 | M14 240496 | Neisseria meningitidis | W | ST-11 complex | No value | UK | SV-2 | Opc- |
| 35811 | M14 240500 | Neisseria meningitidis | W | ST-11 complex | No value | UK | SV-2 | Opc- |
| 35819 | 1126 | Neisseria meningitidis | NG | ST-32 complex | No value | France | SV-1 | Opc+ |
| 35820 | 2963 | Neisseria meningitidis | NG | ST-32 complex | No value | France | SV-1 | Opc+ |
| 35821 | 3141 | Neisseria meningitidis | B | ST-32 complex | No value | France | SV-1 | Opc+ |
| 35822 | 3644 | Neisseria meningitidis | B | ST-32 complex | No value | France | SV-1 | Opc+ |
| 35823 | 1483 | Neisseria meningitidis | B | ST-32 complex | No value | France | SV-1 | Opc+ |
| 35824 | 2018 | Neisseria meningitidis | B | ST-32 complex | No value | France | SV-1 | Opc+ |
| 35829 | M01 240100 | Neisseria meningitidis | B | ST-269 complex | No value | UK | SV-1 | Opc+ |
| 35830 | M13 240674 | Neisseria meningitidis | Y | ST-23 complex | No value | UK | SV-11 | Opc+ |
| 35956 | 255 | Neisseria meningitidis | A | ST-4 complex | Endemic | Burkina Faso | SV-5 | Opc+ |
| 35957 | 890326 | Neisseria meningitidis | Z | ST-103 complex | No value | The Netherlands | SV-5 | Opc- |
| 35958 | BZ 133 | Neisseria meningitidis | B | ST-1 complex | No value | The Netherlands | SV-5 | Opc+ |
| 35959 | EG 328 | Neisseria meningitidis | B | ST-18 complex | No value | Germany | SV-7 | Opc- |
| 35960 | F1576 | Neisseria meningitidis | C | ST-11 complex | Endemic | Ghana | SV-2 | Opc- |
| 35961 | NG E28 | Neisseria meningitidis | B | No value | Carrier | Norway | SV-1 | Opc- |
| 35962 | NG H41 | Neisseria meningitidis | B | No value | No value | Norway | SV-5 | Opc+ |
| 35963 | NG P20 | Neisseria meningitidis | B | ST-11 complex | No value | Norway | SV-2 | Opc- |
| 35964 | SWZ107 | Neisseria meningitidis | B | ST-35 complex | No value | Switzerland | SV-12 | Opc+ |
| 36127 | 12000_2015 | Neisseria meningitidis | Y | ST-167 complex | No value | Ireland | SV-5 | Opc+ |
| 36128 | 12001_2015 | Neisseria meningitidis | B | ST-213 complex | No value | Ireland | SV-12 | Opc- |
| 36129 | 12002_2015 | Neisseria meningitidis | B | ST-269 complex | No value | Ireland | SV-8 | Opc+ |
| 36130 | 12003_2015 | Neisseria meningitidis | B | ST-269 complex | No value | Ireland | SV-1 | Opc+ |
| 36131 | 12004_2015 | Neisseria meningitidis | Y | ST-11 complex | No value | Ireland | SV-2 | Opc- |
| 36132 | 12005_2015 | Neisseria meningitidis | NG | No value | No value | Ireland | SV-8 | Opc+ |
| 36133 | 12006_2015 | Neisseria meningitidis | NG | ST-1136 complex | No value | Ireland | SV-2 | Opc+ |
| 36134 | 12010_2010 | Neisseria meningitidis | B | ST-41/44 complex | No value | Ireland | SV-6 | Opc+ |
| 36135 | 12010_2012 | Neisseria meningitidis | NG | ST-53 complex | No value | Ireland | SV-2 | Opc- |
| 36136 | 12010_2014 | Neisseria meningitidis | B | ST-269 complex | No value | Ireland | SV-8 | Opc+ |
| 36137 | 12013_2014 | Neisseria meningitidis | B | ST-32 complex | No value | Ireland | SV-1 | Opc+ |
| 36138 | 12014_2014 | Neisseria meningitidis | W | ST-22 complex | No value | Ireland | SV-12 | Opc+ |
| 36139 | 12014_2010 | Neisseria meningitidis | B | ST-41/44 complex | No value | Ireland | SV-6 | Opc+ |
| 36141 | 12016_2014 | Neisseria meningitidis | NG | ST-269 complex | No value | Ireland | SV-1 | Opc+ |
| 36143 | 12018_2014 | Neisseria meningitidis | Y | ST-174 complex | No value | Ireland | SV-1 | Opc+ |
| 36144 | 12019_2010 | Neisseria meningitidis | B | ST-41/44 complex | No value | Ireland | SV-6 | Opc+ |
| 36145 | 12019_2014 | Neisseria meningitidis | B | ST-41/44 complex | No value | Ireland | SV-6 | Opc+ |
| 36146 | 12020_2014 | Neisseria meningitidis | B | ST-41/44 complex | No value | Ireland | SV-2 | Opc+ |
| 36147 | 12023_2014 | Neisseria meningitidis | B | ST-269 complex | No value | Ireland | SV-1 | Opc+ |
| 36149 | 12025_2014 | Neisseria meningitidis | B | ST-461 complex | No value | Ireland | SV-1 | Opc- |
| 36150 | 12026_2013 | Neisseria meningitidis | B | ST-213 complex | No value | Ireland | SV-12 | Opc- |
| 36151 | 12026_2014 | Neisseria meningitidis | Y | ST-174 complex | No value | Ireland | SV-1 | Opc+ |
| 36152 | 12030_2013 | Neisseria meningitidis | B | ST-1157 complex | No value | Ireland | SV-1 | Opc+ |
| 36154 | 12031_2014 | Neisseria meningitidis | B | ST-32 complex | No value | Ireland | SV-1 | Opc+ |
| 36155 | 12033_2014 | Neisseria meningitidis | B | No value | No value | Ireland | SV-8 | Opc+ |
| 36156 | 12034_2014 | Neisseria meningitidis | C | ST-11 complex | No value | Ireland | SV-2 | Opc- |
| 36157 | 12035_2014 | Neisseria meningitidis | C | ST-11 complex | No value | Ireland | SV-2 | Opc- |
| 36158 | 12037_2014 | Neisseria meningitidis | B | ST-60 complex | No value | Ireland | SV-11 | Opc+ |
| 36159 | 12038_2014 | Neisseria meningitidis | B | ST-461 complex | No value | Ireland | SV-1 | Opc- |
| 36160 | 12039_2014 | Neisseria meningitidis | B | No value | No value | Ireland | SV-10 | Opc+ |
| 36162 | 12041_2014 | Neisseria meningitidis | B | ST-41/44 complex | No value | Ireland | SV-2 | Opc+ |
| 36163 | 12042_2014 | Neisseria meningitidis | B | ST-269 complex | No value | Ireland | SV-8 | Opc+ |
| 36164 | 12043_2014 | Neisseria meningitidis | B | No value | No value | Ireland | SV-1 | Opc+ |
| 36165 | 12044_2014 | Neisseria meningitidis | B | ST-41/44 complex | No value | Ireland | SV-6 | Opc+ |
| 36166 | 12045_2014 | Neisseria meningitidis | NG | ST-461 complex | No value | Ireland | SV-1 | Opc- |
| 36168 | 12047_2014 | Neisseria meningitidis | B | ST-41/44 complex | No value | Ireland | SV-2 | Opc+ |
| 36169 | 12048_2014 | Neisseria meningitidis | B | ST-41/44 complex | No value | Ireland | SV-2 | Opc- |
| 36170 | 12049_2009 | Neisseria meningitidis | B | No value | No value | Ireland | SV-12 | Opc+ |
| 36171 | 12053_2009 | Neisseria meningitidis | B | ST-269 complex | No value | Ireland | SV-1 | Opc+ |
| 36202 | BM48 | Neisseria meningitidis | C | ST-11 complex | Sporadic case | Greece | SV-2 | Opc- |
| 36203 | BM48a | Neisseria meningitidis | C | ST-11 complex | Carrier | Greece | SV-2 | Opc- |
| 36204 | BM59 | Neisseria meningitidis | B | ST-41/44 complex | Sporadic case | Greece | SV-6 | Opc+ |
| 36205 | BM59a | Neisseria meningitidis | B | ST-41/44 complex | Carrier | Greece | SV-6 | Opc+ |
| 36206 | BM59b | Neisseria meningitidis | B | ST-41/44 complex | Carrier | Greece | SV-6 | Opc+ |
| 36207 | BM59c | Neisseria meningitidis | B | ST-41/44 complex | Carrier | Greece | SV-6 | Opc+ |
| 36208 | BM65 | Neisseria meningitidis | B | No value | Sporadic case | Greece | SV-10 | Opc+ |
| 36209 | BM65a | Neisseria meningitidis | B | No value | Carrier | Greece | SV-10 | Opc+ |
| 36210 | BM65b | Neisseria meningitidis | B | No value | Carrier | Greece | SV-14 | Opc+ |
| 36211 | BM65c | Neisseria meningitidis | B | No value | Carrier | Greece | SV-10 | Opc+ |

| | | | | | | | | |
|---|---|---|---|---|---|---|---|---|
| 36314 | 1459000516 | Neisseria meningitidis | NG | ST-11 complex | No value | Sweden | SV-2 | Opc- |
| 36315 | 1459000524 | Neisseria meningitidis | NG | ST-269 complex | No value | Sweden | SV-8 | Opc+ |
| 36386 | 2654 | Neisseria meningitidis | C | No value | Endemic | Italy | SV-2 | Opc- |
| 36402 | 2669 | Neisseria meningitidis | C | ST-11 complex | Endemic | Italy | SV-2 | Opc- |
| 36444 | 2417 | Neisseria meningitidis | C | ST-11 complex | Endemic | Italy | SV-2 | Opc- |
| 36445 | 2418 | Neisseria meningitidis | C | ST-11 complex | Endemic | Italy | SV-2 | Opc- |
| 36446 | 2419 | Neisseria meningitidis | C | ST-11 complex | Endemic | Italy | SV-2 | Opc- |
| 36447 | 2420 | Neisseria meningitidis | C | ST-11 complex | Endemic | Italy | SV-2 | Opc- |
| 36448 | 2636 | Neisseria meningitidis | C | ST-11 complex | Endemic | Italy | SV-2 | Opc- |
| 36449 | 2639 | Neisseria meningitidis | C | ST-11 complex | Endemic | Italy | SV-2 | Opc- |
| 36450 | 2644 | Neisseria meningitidis | C | ST-11 complex | Endemic | Italy | SV-2 | Opc- |
| 36451 | 2652 | Neisseria meningitidis | C | ST-11 complex | Endemic | Italy | SV-2 | Opc- |
| 36452 | 2664 | Neisseria meningitidis | C | ST-11 complex | Endemic | Italy | SV-2 | Opc- |
| 36453 | 2665 | Neisseria meningitidis | C | ST-11 complex | Endemic | Italy | SV-2 | Opc- |
| 36454 | 1982 | Neisseria meningitidis | C | ST-11 complex | Endemic | Italy | SV-2 | Opc- |
| 36455 | 1983 | Neisseria meningitidis | C | ST-11 complex | Endemic | Italy | SV-2 | Opc- |
| 36456 | 1984 | Neisseria meningitidis | C | ST-11 complex | Endemic | Italy | SV-2 | Opc- |
| 36457 | 1985 | Neisseria meningitidis | C | ST-11 complex | Endemic | Italy | SV-2 | Opc- |
| 36458 | 1986 | Neisseria meningitidis | C | ST-11 complex | Endemic | Italy | SV-2 | Opc- |
| 36459 | 1987 | Neisseria meningitidis | C | ST-11 complex | Endemic | Italy | SV-2 | Opc- |
| 36460 | 2421 | Neisseria meningitidis | C | ST-11 complex | Endemic | Italy | SV-2 | Opc- |
| 36461 | 2560 | Neisseria meningitidis | C | ST-11 complex | Endemic | Italy | SV-2 | Opc- |
| 36462 | 1981 | Neisseria meningitidis | C | ST-11 complex | Endemic | Italy | SV-2 | Opc- |
| 36463 | 2380 | Neisseria meningitidis | C | ST-11 complex | Endemic | Italy | SV-2 | Opc- |
| 36464 | 2411 | Neisseria meningitidis | C | ST-11 complex | Endemic | Italy | SV-2 | Opc- |
| 36465 | 2387 | Neisseria meningitidis | C | ST-11 complex | Endemic | Italy | SV-2 | Opc- |
| 36466 | 2415 | Neisseria meningitidis | C | ST-11 complex | Endemic | Italy | SV-2 | Opc- |
| 36467 | 2393 | Neisseria meningitidis | C | ST-11 complex | Endemic | Italy | SV-2 | Opc- |
| 36468 | 2405 | Neisseria meningitidis | C | ST-11 complex | Endemic | Italy | SV-2 | Opc- |
| 36469 | 2433 | Neisseria meningitidis | C | ST-11 complex | Endemic | Italy | SV-2 | Opc- |
| 36470 | 2394 | Neisseria meningitidis | C | ST-11 complex | Endemic | Italy | SV-2 | Opc- |
| 36471 | 2436 | Neisseria meningitidis | C | ST-11 complex | Endemic | Italy | SV-2 | Opc- |
| 36472 | 2437 | Neisseria meningitidis | C | ST-11 complex | Endemic | Italy | SV-2 | Opc- |
| 36473 | 2444 | Neisseria meningitidis | C | ST-11 complex | Endemic | Italy | SV-2 | Opc- |
| 36668 | 09.1347.W | Neisseria meningitidis | B | No value | No value | UK | SV-8 | Opc+ |
| 36669 | 11.2796.K | Neisseria meningitidis | B | ST-461 complex | No value | UK | SV-5 | Opc- |
| 36670 | 2670 | Neisseria meningitidis | C | ST-11 complex | Endemic | Italy | SV-2 | Opc- |
| 36764 | 2506 | Neisseria meningitidis | C | ST-11 complex | Endemic | Italy | SV-2 | Opc- |
| 36765 | 2569 | Neisseria meningitidis | C | ST-11 complex | Endemic | Italy | SV-2 | Opc- |
| 36766 | 2662 | Neisseria meningitidis | C | ST-11 complex | Endemic | Italy | SV-2 | Opc- |
| 36767 | 2691 | Neisseria meningitidis | C | ST-11 complex | Endemic | Italy | SV-2 | Opc- |
| 36768 | 2690 | Neisseria meningitidis | C | ST-11 complex | Endemic | Italy | SV-2 | Opc- |
| 36769 | 2656 | Neisseria meningitidis | C | ST-11 complex | Endemic | Italy | SV-2 | Opc- |
| 36770 | 2646 | Neisseria meningitidis | C | ST-11 complex | Endemic | Italy | SV-2 | Opc- |
| 36771 | IT2632 | Neisseria meningitidis | C | ST-11 complex | Endemic | Italy | SV-2 | Opc- |
| 36772 | IT2625 | Neisseria meningitidis | C | ST-11 complex | Endemic | Italy | SV-2 | Opc- |
| 36773 | IT2592 | Neisseria meningitidis | C | ST-11 complex | Endemic | Italy | SV-2 | Opc- |
| 36774 | IT2580 | Neisseria meningitidis | C | ST-11 complex | Endemic | Italy | SV-2 | Opc- |
| 36775 | IT2577 | Neisseria meningitidis | C | ST-11 complex | Endemic | Italy | SV-2 | Opc- |
| 36776 | IT2576 | Neisseria meningitidis | C | ST-11 complex | Endemic | Italy | SV-2 | Opc- |
| 36777 | IT2561 | Neisseria meningitidis | C | ST-11 complex | Endemic | Italy | SV-2 | Opc- |
| 36779 | 107507 | Neisseria meningitidis | W | ST-11 complex | No value | Finland | SV-2 | Opc- |
| 36780 | 107499 | Neisseria meningitidis | W | ST-11 complex | No value | Finland | SV-2 | Opc- |
| 36781 | 107497 | Neisseria meningitidis | W | ST-11 complex | No value | Finland | SV-2 | Opc- |
| 36782 | 2677 | Neisseria meningitidis | C | ST-11 complex | Endemic | Italy | SV-2 | Opc- |
| 36783 | 2640 | Neisseria meningitidis | C | ST-334 complex | Endemic | Italy | SV-15 | Opc- |
| 36784 | 2701 | Neisseria meningitidis | C | ST-11 complex | Endemic | Italy | SV-2 | Opc- |
| 36785 | WS71210_M01_242679 | Neisseria meningitidis | W | ST-11 complex | No value | UK | SV-2 | Opc- |
| 36786 | WS71210_M98_250375 | Neisseria meningitidis | B | ST-32 complex | No value | UK | SV-1 | Opc+ |
| 36787 | WS71210_M98_250840 | Neisseria meningitidis | B | ST-60 complex | No value | UK | SV-11 | Opc+ |
| 36788 | WS71210_M98_252086 | Neisseria meningitidis | B | ST-41/44 complex | No value | UK | SV-5 | Opc- |
| 36789 | WS71210_M00_240207 | Neisseria meningitidis | NG | ST-41/44 complex | No value | UK | SV-2 | Opc+ |
| 36790 | WS71210_M00_240993 | Neisseria meningitidis | B | ST-41/44 complex | No value | UK | SV-2 | Opc+ |
| 36791 | WS71210_M02_240168 | Neisseria meningitidis | B | ST-269 complex | No value | UK | SV-8 | Opc+ |
| 36792 | WS71210_M05_240177 | Neisseria meningitidis | B | ST-32 complex | No value | UK | SV-1 | Opc+ |
| 36793 | WS71210_M09_240022 | Neisseria meningitidis | B | ST-32 complex | No value | UK | SV-1 | Opc+ |
| 36794 | WS71210_M98_253069 | Neisseria meningitidis | B | ST-41/44 complex | No value | UK | SV-2 | Opc+ |
| 36795 | WS71210_M98_253766 | Neisseria meningitidis | B | ST-8 complex | No value | UK | SV-14 | Opc- |
| 36796 | WS71210_F85_0000045 | Neisseria meningitidis | C | ST-11 complex | No value | UK | SV-2 | Opc- |
| 36797 | WS71210_F85_0000704 | Neisseria meningitidis | C | ST-11 complex | No value | UK | SV-2 | Opc- |
| 36798 | WS71210_F85_000311 | Neisseria meningitidis | W | No value | No value | UK | SV-1 | Opc+ |
| 36799 | WS71004_M99_241396 | Neisseria meningitidis | NG | ST-60 complex | No value | UK | SV-11 | Opc+ |
| 36800 | WS71004_M01_242679 | Neisseria meningitidis | W | ST-11 complex | No value | UK | SV-2 | Opc- |
| 36801 | WS71004_M98_250375 | Neisseria meningitidis | B | ST-32 complex | No value | UK | SV-1 | Opc+ |

| | | | | | | | | |
|---|---|---|---|---|---|---|---|---|
| 36802 | WS71004_M98_250840 | Neisseria meningitidis | B | ST-60 complex | No value | UK | SV-11 | Opc+ |
| 36803 | WS71004_M98_252086 | Neisseria meningitidis | B | ST-41/44 complex | No value | UK | SV-5 | Opc- |
| 36804 | WS71004_M00_240207 | Neisseria meningitidis | NG | ST-41/44 complex | No value | UK | SV-2 | Opc+ |
| 36805 | WS71004_M00_240993 | Neisseria meningitidis | B | ST-41/44 complex | No value | UK | SV-2 | Opc+ |
| 36806 | WS71004_M02_240168 | Neisseria meningitidis | B | ST-269 complex | No value | UK | SV-8 | Opc+ |
| 36807 | WS71004_M05_240177 | Neisseria meningitidis | B | ST-32 complex | No value | UK | SV-1 | Opc+ |
| 36808 | WS71004_M09_240022 | Neisseria meningitidis | B | ST-32 complex | No value | UK | SV-1 | Opc+ |
| 36809 | WS71004_M98_253069 | Neisseria meningitidis | B | ST-41/44 complex | No value | UK | SV-2 | Opc+ |
| 36810 | WS71004_M98_253766 | Neisseria meningitidis | B | ST-8 complex | No value | UK | SV-14 | Opc- |
| 36811 | WS71004_F85_0000045 | Neisseria meningitidis | C | ST-11 complex | No value | UK | SV-2 | Opc- |
| 36812 | WS71004_F85_0000704 | Neisseria meningitidis | C | ST-11 complex | No value | UK | SV-2 | Opc- |
| 36813 | WS71004_F85_000311 | Neisseria meningitidis | W | No value | No value | UK | SV-1 | Opc+ |
| 36814 | 12005_2015b | Neisseria meningitidis | NG | No value | No value | Ireland | SV-8 | Opc+ |
| 36815 | 12006_2015b | Neisseria meningitidis | NG | ST-1136 complex | No value | Ireland | SV-2 | Opc+ |
| 36816 | 12007_2015 | Neisseria meningitidis | NG | ST-461 complex | No value | Ireland | SV-1 | Opc- |
| 36817 | 12008_2015 | Neisseria meningitidis | NG | ST-11 complex | No value | Ireland | SV-2 | Opc- |
| 36818 | 12009_2015 | Neisseria meningitidis | NG | No value | No value | Ireland | SV-2 | Opc+ |
| 36819 | 12010_2015 | Neisseria meningitidis | NG | ST-269 complex | No value | Ireland | SV-1 | Opc+ |
| 36820 | 12011_2015 | Neisseria meningitidis | NG | ST-32 complex | No value | Ireland | SV-1 | Opc+ |
| 36821 | 12012_2014 | Neisseria meningitidis | B | ST-11 complex | No value | Ireland | SV-2 | Opc- |
| 36822 | 12012_2015 | Neisseria meningitidis | NG | ST-11 complex | No value | Ireland | SV-2 | Opc- |
| 36823 | 12014_2015 | Neisseria meningitidis | NG | ST-11 complex | No value | Ireland | SV-2 | Opc- |
| 36824 | 12015_2015 | Neisseria meningitidis | NG | ST-22 complex | No value | Ireland | SV-12 | Opc+ |
| 36825 | 12016_2015 | Neisseria meningitidis | NG | ST-11 complex | No value | Ireland | SV-2 | Opc- |
| 36826 | 12017_2015 | Neisseria meningitidis | NG | ST-41/44 complex | No value | Ireland | SV-6 | Opc+ |
| 36827 | 12018_2015 | Neisseria meningitidis | NG | ST-11 complex | No value | Ireland | SV-2 | Opc- |
| 36828 | 12020_2015 | Neisseria meningitidis | NG | ST-11 complex | No value | Ireland | SV-2 | Opc- |
| 36829 | 12021_2015 | Neisseria meningitidis | NG | ST-23 complex | No value | Ireland | SV-11 | Opc+ |
| 36830 | 12028_2014 | Neisseria meningitidis | B | ST-11 complex | No value | Ireland | SV-2 | Opc- |
| 36844 | 2670 | Neisseria meningitidis | C | ST-11 complex | Endemic | Italy | SV-2 | Opc- |
| 36845 | 2685 | Neisseria meningitidis | C | ST-11 complex | Endemic | Italy | SV-2 | Opc- |
| 36846 | 2692 | Neisseria meningitidis | C | ST-11 complex | Endemic | Italy | SV-2 | Opc- |
| 36847 | 2693 | Neisseria meningitidis | C | ST-11 complex | Endemic | Italy | SV-2 | Opc- |
| 36848 | 2593 | Neisseria meningitidis | C | ST-11 complex | Endemic | Italy | SV-2 | Opc- |
| 36849 | 2585 | Neisseria meningitidis | C | ST-11 complex | Endemic | Italy | SV-2 | Opc- |
| 36850 | 2435 | Neisseria meningitidis | C | ST-8 complex | Endemic | Italy | SV-10 | Opc- |
| 36851 | 2157 | Neisseria meningitidis | C | ST-11 complex | Endemic | Italy | SV-2 | Opc- |
| 36852 | 2020 | Neisseria meningitidis | C | ST-8 complex | Endemic | Italy | SV-10 | Opc- |
| 36853 | 1953 | Neisseria meningitidis | C | ST-8 complex | Endemic | Italy | SV-10 | Opc- |
| 37019 | 2571 | Neisseria meningitidis | C | ST-11 complex | Endemic | Italy | SV-2 | Opc- |
| 37020 | 2605 | Neisseria meningitidis | C | ST-11 complex | Endemic | Italy | SV-2 | Opc- |
| 37593 | 2459 | Neisseria meningitidis | C | ST-11 complex | Endemic | Italy | SV-2 | Opc- |
| 37594 | 2493 | Neisseria meningitidis | C | ST-11 complex | Endemic | Italy | SV-2 | Opc- |
| 37595 | 2505 | Neisseria meningitidis | C | ST-11 complex | Endemic | Italy | SV-2 | Opc- |
| 37596 | 2506 | Neisseria meningitidis | C | ST-11 complex | Endemic | Italy | SV-2 | Opc- |
| 37597 | 2530 | Neisseria meningitidis | C | ST-11 complex | Endemic | Italy | SV-2 | Opc- |
| 37598 | 2531 | Neisseria meningitidis | C | ST-11 complex | Endemic | Italy | SV-2 | Opc- |
| 37599 | 2532 | Neisseria meningitidis | C | ST-11 complex | Endemic | Italy | SV-2 | Opc- |
| 37600 | 2539 | Neisseria meningitidis | C | ST-11 complex | Endemic | Italy | SV-2 | Opc- |
| 37601 | 2555 | Neisseria meningitidis | C | ST-11 complex | Endemic | Italy | SV-2 | Opc- |
| 37602 | 2603 | Neisseria meningitidis | C | ST-11 complex | Endemic | Italy | SV-2 | Opc- |
| 37603 | A4BZ577 | Neisseria meningitidis | NG | No value | No value | Canada | SV-7 | Opc+ |
| 37611 | 21525 | Neisseria meningitidis | NG | ST-60 complex | No value | South Africa | SV-11 | Opc+ |
| 37612 | 24267 | Neisseria meningitidis | NG | ST-254 complex | No value | South Africa | SV-1 | Opc+ |
| 37613 | 23151 | Neisseria meningitidis | NG | ST-254 complex | No value | South Africa | SV-1 | Opc+ |
| 37614 | 38699 | Neisseria meningitidis | NG | ST-254 complex | Endemic | South Africa | SV-1 | Opc+ |
| 37615 | 37066 | Neisseria meningitidis | NG | ST-60 complex | Endemic | South Africa | SV-11 | Opc+ |
| 37616 | 38465 | Neisseria meningitidis | NG | ST-41/44 complex | Endemic | South Africa | SV-6 | Opc+ |
| 37662 | M14 240138 | Neisseria meningitidis | B | ST-213 complex | No value | UK | SV-12 | Opc- |
| 37663 | M14 240148 | Neisseria meningitidis | B | ST-41/44 complex | No value | UK | SV-2 | Opc+ |
| 37664 | M14 240156 | Neisseria meningitidis | Y | ST-23 complex | No value | UK | SV-11 | Opc+ |
| 37665 | M14 240166 | Neisseria meningitidis | B | ST-269 complex | No value | UK | SV-8 | Opc+ |
| 37666 | M14 240170 | Neisseria meningitidis | Y | ST-23 complex | No value | UK | SV-11 | Opc+ |
| 37667 | M14 240172 | Neisseria meningitidis | Y | ST-23 complex | No value | UK | SV-11 | Opc+ |
| 37668 | M14 240229 | Neisseria meningitidis | C | ST-11 complex | No value | UK | SV-2 | Opc- |
| 37669 | M14 240230 | Neisseria meningitidis | B | ST-41/44 complex | No value | UK | SV-2 | Opc+ |
| 37670 | M14 240394 | Neisseria meningitidis | W | ST-11 complex | No value | UK | SV-2 | Opc- |
| 37671 | M14 240439 | Neisseria meningitidis | NG | ST-167 complex | No value | UK | SV-5 | Opc+ |
| 37672 | M14 240501 | Neisseria meningitidis | Y | ST-23 complex | No value | UK | SV-11 | Opc+ |
| 37673 | M14 240502 | Neisseria meningitidis | W | ST-11 complex | No value | UK | SV-2 | Opc- |
| 37674 | M14 240503 | Neisseria meningitidis | B | ST-269 complex | No value | UK | SV-8 | Opc+ |
| 37675 | M14 240504 | Neisseria meningitidis | W | ST-11 complex | No value | UK | SV-2 | Opc- |
| 37676 | M14 240506 | Neisseria meningitidis | Y | ST-167 complex | No value | UK | SV-5 | Opc+ |
| 37677 | M14 240507 | Neisseria meningitidis | B | ST-269 complex | No value | UK | SV-8 | Opc+ |

| | | | | | | | | |
|---|---|---|---|---|---|---|---|---|
| 37678 | M14 240508 | Neisseria meningitidis | B | ST-41/44 complex | No value | UK | SV-1 | Opc+ |
| 37679 | M14 240509 | Neisseria meningitidis | C | ST-11 complex | No value | UK | SV-2 | Opc- |
| 37680 | M14 240510 | Neisseria meningitidis | B | ST-213 complex | No value | UK | SV-12 | Opc- |
| 37681 | M14 240511 | Neisseria meningitidis | W | ST-11 complex | No value | UK | SV-2 | Opc- |
| 37682 | M14 240513 | Neisseria meningitidis | B | ST-32 complex | No value | UK | SV-1 | Opc+ |
| 37683 | M14 240514 | Neisseria meningitidis | B | ST-162 complex | No value | UK | SV-1 | Opc+ |
| 37684 | M14 240516 | Neisseria meningitidis | B | ST-41/44 complex | No value | UK | SV-2 | Opc+ |
| 37685 | M14 240517 | Neisseria meningitidis | W | ST-11 complex | No value | UK | SV-2 | Opc- |
| 37686 | M14 240518 | Neisseria meningitidis | B | ST-41/44 complex | No value | UK | SV-2 | Opc+ |
| 37687 | M14 240519 | Neisseria meningitidis | C | ST-32 complex | No value | UK | SV-1 | Opc+ |
| 37688 | M14 240520 | Neisseria meningitidis | B | ST-269 complex | No value | UK | SV-8 | Opc+ |
| 37689 | M14 240521 | Neisseria meningitidis | Y | ST-23 complex | No value | UK | SV-11 | Opc+ |
| 37690 | M14 240522 | Neisseria meningitidis | W | ST-11 complex | No value | UK | SV-2 | Opc- |
| 37691 | M14 240523 | Neisseria meningitidis | Y | ST-23 complex | No value | UK | SV-11 | Opc+ |
| 37692 | M14 240524 | Neisseria meningitidis | W | ST-11 complex | No value | UK | SV-2 | Opc- |
| 37693 | M14 240525 | Neisseria meningitidis | W | ST-11 complex | No value | UK | SV-2 | Opc- |
| 37694 | M14 240526 | Neisseria meningitidis | Y | ST-23 complex | No value | UK | SV-11 | Opc+ |
| 37695 | M14 240527 | Neisseria meningitidis | B | ST-32 complex | No value | UK | SV-1 | Opc+ |
| 37696 | M14 240528 | Neisseria meningitidis | B | ST-32 complex | No value | UK | SV-1 | Opc+ |
| 37697 | M14 240530 | Neisseria meningitidis | Y | ST-23 complex | No value | UK | SV-11 | Opc+ |
| 37698 | M14 240531 | Neisseria meningitidis | B | ST-269 complex | No value | UK | SV-1 | Opc+ |
| 37699 | M14 240532 | Neisseria meningitidis | W | ST-11 complex | No value | UK | SV-2 | Opc- |
| 37700 | M14 240533 | Neisseria meningitidis | B | ST-41/44 complex | No value | UK | SV-2 | Opc+ |
| 37701 | M14 240534 | Neisseria meningitidis | W | ST-11 complex | No value | UK | SV-2 | Opc- |
| 37702 | M14 240535 | Neisseria meningitidis | B | ST-41/44 complex | No value | UK | SV-5 | Opc- |
| 37703 | M14 240536 | Neisseria meningitidis | Y | ST-23 complex | No value | UK | SV-11 | Opc+ |
| 37704 | M14 240539 | Neisseria meningitidis | B | ST-269 complex | No value | UK | SV-8 | Opc+ |
| 37705 | M14 240541 | Neisseria meningitidis | B | No value | No value | UK | SV-1 | Opc- |
| 37706 | M14 240542 | Neisseria meningitidis | B | ST-269 complex | No value | UK | SV-1 | Opc+ |
| 37707 | M14 240543 | Neisseria meningitidis | B | ST-41/44 complex | No value | UK | SV-2 | Opc+ |
| 37708 | M14 240545 | Neisseria meningitidis | W | ST-11 complex | No value | UK | SV-2 | Opc- |
| 37709 | M14 240546 | Neisseria meningitidis | C | ST-11 complex | No value | UK | SV-2 | Opc- |
| 37710 | M14 240548 | Neisseria meningitidis | W | ST-11 complex | No value | UK | SV-2 | Opc- |
| 37711 | M14 240550 | Neisseria meningitidis | W | ST-11 complex | No value | UK | SV-2 | Opc- |
| 37712 | M14 240551 | Neisseria meningitidis | B | ST-41/44 complex | No value | UK | SV-2 | Opc+ |
| 37713 | M14 240552 | Neisseria meningitidis | Y | No value | No value | UK | SV-5 | Opc+ |
| 37714 | M14 240553 | Neisseria meningitidis | Y | ST-23 complex | No value | UK | SV-11 | Opc+ |
| 37715 | M14 240554 | Neisseria meningitidis | B | ST-60 complex | No value | UK | SV-11 | Opc+ |
| 37716 | M14 240555 | Neisseria meningitidis | W | ST-11 complex | No value | UK | SV-2 | Opc- |
| 37717 | M14 240556 | Neisseria meningitidis | W | ST-11 complex | No value | UK | SV-2 | Opc- |
| 37718 | M14 240557 | Neisseria meningitidis | B | ST-41/44 complex | No value | UK | SV-2 | Opc+ |
| 37719 | M14 240558 | Neisseria meningitidis | B | ST-41/44 complex | No value | UK | SV-14 | Opc+ |
| 37720 | M14 240560 | Neisseria meningitidis | B | No value | No value | UK | SV-1 | Opc+ |
| 37721 | M14 240561 | Neisseria meningitidis | B | ST-461 complex | No value | UK | SV-1 | Opc- |
| 37722 | M14 240562 | Neisseria meningitidis | W | ST-11 complex | No value | UK | SV-2 | Opc- |
| 37723 | M14 240563 | Neisseria meningitidis | B | ST-60 complex | No value | UK | SV-11 | Opc+ |
| 37724 | M14 240564 | Neisseria meningitidis | B | ST-41/44 complex | No value | UK | SV-6 | Opc+ |
| 37725 | M14 240565 | Neisseria meningitidis | B | ST-41/44 complex | No value | UK | SV-2 | Opc+ |
| 37726 | M14 240566 | Neisseria meningitidis | B | ST-213 complex | No value | UK | SV-12 | Opc- |
| 37727 | M14 240567 | Neisseria meningitidis | B | ST-41/44 complex | No value | UK | SV-10 | Opc+ |
| 37728 | M14 240568 | Neisseria meningitidis | C | ST-11 complex | No value | UK | SV-2 | Opc- |
| 37729 | M14 240571 | Neisseria meningitidis | Y | ST-174 complex | No value | UK | SV-1 | Opc+ |
| 37730 | M14 240572 | Neisseria meningitidis | W | ST-11 complex | No value | UK | SV-2 | Opc- |
| 37731 | M14 240573 | Neisseria meningitidis | W | ST-11 complex | No value | UK | SV-2 | Opc- |
| 37732 | M14 240574 | Neisseria meningitidis | W | ST-11 complex | No value | UK | SV-2 | Opc- |
| 37733 | M14 240575 | Neisseria meningitidis | B | ST-213 complex | No value | UK | SV-12 | Opc- |
| 37734 | M14 240576 | Neisseria meningitidis | B | ST-32 complex | No value | UK | SV-1 | Opc+ |
| 37735 | M14 240580 | Neisseria meningitidis | B | ST-4821 complex | No value | UK | SV-19 | Opc+ |
| 37736 | M14 240581 | Neisseria meningitidis | W | ST-11 complex | No value | UK | SV-2 | Opc- |
| 37737 | M14 240582 | Neisseria meningitidis | B | ST-41/44 complex | No value | UK | SV-10 | Opc+ |
| 37738 | M14 240585 | Neisseria meningitidis | W | ST-11 complex | No value | UK | SV-2 | Opc- |
| 37739 | M14 240587 | Neisseria meningitidis | W | ST-11 complex | No value | UK | SV-2 | Opc- |
| 37740 | M14 240590 | Neisseria meningitidis | W | ST-22 complex | No value | UK | SV-12 | Opc+ |
| 37741 | M14 240591 | Neisseria meningitidis | B | ST-32 complex | No value | UK | SV-1 | Opc+ |
| 37742 | M14 240593 | Neisseria meningitidis | W | ST-11 complex | No value | UK | SV-2 | Opc- |
| 37743 | M14 240595 | Neisseria meningitidis | B | ST-41/44 complex | No value | UK | SV-2 | Opc+ |
| 37744 | M14 240596 | Neisseria meningitidis | B | No value | No value | UK | SV-8 | Opc+ |
| 37745 | M14 240598 | Neisseria meningitidis | B | ST-32 complex | No value | UK | SV-1 | Opc+ |
| 37746 | M14 240601 | Neisseria meningitidis | W | ST-11 complex | No value | UK | SV-2 | Opc- |
| 37747 | M14 240602 | Neisseria meningitidis | W | ST-11 complex | No value | UK | SV-2 | Opc- |
| 37748 | M14 240604 | Neisseria meningitidis | W | ST-11 complex | No value | UK | SV-2 | Opc- |
| 37749 | M14 240605 | Neisseria meningitidis | W | ST-11 complex | No value | UK | SV-2 | Opc- |
| 37750 | M14 240606 | Neisseria meningitidis | B | ST-32 complex | No value | UK | SV-1 | Opc+ |
| 37751 | M14 240607 | Neisseria meningitidis | Y | ST-23 complex | No value | UK | SV-11 | Opc+ |

| | | | | | | | | |
|---|---|---|---|---|---|---|---|---|
| 37752 | M14 240616 | Neisseria meningitidis | C | ST-11 complex | No value | UK | SV-2 | Opc- |
| 37753 | M14 240617 | Neisseria meningitidis | B | ST-269 complex | No value | UK | SV-1 | Opc+ |
| 37754 | M14 240618 | Neisseria meningitidis | W | ST-11 complex | No value | UK | SV-2 | Opc- |
| 37755 | M14 240619 | Neisseria meningitidis | B | ST-269 complex | No value | UK | SV-8 | Opc+ |
| 37756 | M14 240620 | Neisseria meningitidis | B | ST-32 complex | No value | UK | SV-1 | Opc+ |
| 37757 | M14 240621 | Neisseria meningitidis | W | ST-11 complex | No value | UK | SV-2 | Opc- |
| 37758 | M14 240622 | Neisseria meningitidis | C | ST-11 complex | No value | UK | SV-2 | Opc- |
| 37759 | M14 240623 | Neisseria meningitidis | B | ST-32 complex | No value | UK | SV-1 | Opc+ |
| 37760 | M14 240624 | Neisseria meningitidis | W | ST-11 complex | No value | UK | SV-2 | Opc- |
| 37761 | M14 240625 | Neisseria meningitidis | B | ST-41/44 complex | No value | UK | SV-2 | Opc+ |
| 37762 | M14 240626 | Neisseria meningitidis | B | ST-269 complex | No value | UK | SV-1 | Opc+ |
| 37763 | M14 240627 | Neisseria meningitidis | C | ST-11 complex | No value | UK | SV-2 | Opc- |
| 37764 | M14 240628 | Neisseria meningitidis | W | ST-11 complex | No value | UK | SV-2 | Opc- |
| 37765 | M14 240629 | Neisseria meningitidis | Y | ST-23 complex | No value | UK | SV-11 | Opc+ |
| 37766 | M14 240630 | Neisseria meningitidis | B | ST-269 complex | No value | UK | SV-8 | Opc+ |
| 37767 | M14 240631 | Neisseria meningitidis | B | ST-41/44 complex | No value | UK | SV-2 | Opc+ |
| 37768 | M14 240632 | Neisseria meningitidis | Y | ST-23 complex | No value | UK | SV-11 | Opc+ |
| 37769 | M14 240633 | Neisseria meningitidis | W | ST-11 complex | No value | UK | SV-2 | Opc- |
| 37770 | M14 240634 | Neisseria meningitidis | W | ST-11 complex | No value | UK | SV-2 | Opc- |
| 37771 | M14 240635 | Neisseria meningitidis | Y | ST-23 complex | No value | UK | SV-11 | Opc+ |
| 37772 | M14 240636 | Neisseria meningitidis | B | ST-41/44 complex | No value | UK | SV-2 | Opc+ |
| 37773 | M14 240637 | Neisseria meningitidis | B | ST-18 complex | No value | UK | SV-7 | Opc- |
| 37774 | M14 240638 | Neisseria meningitidis | W | ST-11 complex | No value | UK | SV-2 | Opc- |
| 37775 | M14 240639 | Neisseria meningitidis | B | ST-41/44 complex | No value | UK | SV-2 | Opc+ |
| 37776 | M14 240640 | Neisseria meningitidis | B | ST-269 complex | No value | UK | SV-8 | Opc+ |
| 37777 | M14 240641 | Neisseria meningitidis | B | ST-1157 complex | No value | UK | SV-1 | Opc+ |
| 37779 | M14 240643 | Neisseria meningitidis | B | ST-41/44 complex | No value | UK | SV-2 | Opc+ |
| 37780 | M14 240645 | Neisseria meningitidis | B | ST-461 complex | No value | UK | SV-1 | Opc- |
| 37781 | M14 240646 | Neisseria meningitidis | B | ST-269 complex | No value | UK | SV-8 | Opc+ |
| 37782 | M14 240647 | Neisseria meningitidis | B | ST-269 complex | No value | UK | SV-8 | Opc+ |
| 37783 | M14 240648 | Neisseria meningitidis | B | ST-35 complex | No value | UK | SV-16 | Opc+ |
| 37784 | M14 240649 | Neisseria meningitidis | W | ST-11 complex | No value | UK | SV-2 | Opc- |
| 37785 | M14 240650 | Neisseria meningitidis | B | No value | No value | UK | SV-5 | Opc- |
| 37786 | M14 240651 | Neisseria meningitidis | Y | ST-167 complex | No value | UK | SV-5 | Opc+ |
| 37787 | M14 240652 | Neisseria meningitidis | C | ST-11 complex | No value | UK | SV-2 | Opc- |
| 37788 | M14 240653 | Neisseria meningitidis | B | ST-213 complex | No value | UK | SV-12 | Opc- |
| 37789 | M15 240001 | Neisseria meningitidis | B | ST-32 complex | No value | UK | SV-1 | Opc+ |
| 37790 | M15 240002 | Neisseria meningitidis | B | ST-269 complex | No value | UK | SV-8 | Opc+ |
| 37791 | M15 240003 | Neisseria meningitidis | B | ST-269 complex | No value | UK | SV-1 | Opc+ |
| 37792 | M15 240004 | Neisseria meningitidis | B | ST-1157 complex | No value | UK | SV-1 | Opc+ |
| 37793 | M15 240005 | Neisseria meningitidis | W | ST-11 complex | No value | UK | SV-2 | Opc- |
| 37794 | M15 240006 | Neisseria meningitidis | Y | ST-23 complex | No value | UK | SV-11 | Opc+ |
| 37795 | M15 240007 | Neisseria meningitidis | Y | ST-23 complex | No value | UK | SV-11 | Opc+ |
| 37796 | M15 240009 | Neisseria meningitidis | Y | ST-23 complex | No value | UK | SV-11 | Opc+ |
| 37797 | M15 240010 | Neisseria meningitidis | W | ST-11 complex | No value | UK | SV-2 | Opc- |
| 37798 | M15 240011 | Neisseria meningitidis | W | ST-11 complex | No value | UK | SV-2 | Opc- |
| 37799 | M15 240012 | Neisseria meningitidis | W | ST-11 complex | No value | UK | SV-2 | Opc- |
| 37800 | M15 240014 | Neisseria meningitidis | B | ST-269 complex | No value | UK | SV-8 | Opc+ |
| 37801 | M15 240015 | Neisseria meningitidis | W | ST-11 complex | No value | UK | SV-2 | Opc- |
| 37802 | M15 240016 | Neisseria meningitidis | B | ST-41/44 complex | No value | UK | SV-2 | Opc+ |
| 37803 | M15 240017 | Neisseria meningitidis | B | ST-41/44 complex | No value | UK | SV-2 | Opc+ |
| 37804 | M15 240020 | Neisseria meningitidis | B | No value | No value | UK | SV-1 | Opc+ |
| 37805 | M15 240021 | Neisseria meningitidis | W | ST-11 complex | No value | UK | SV-2 | Opc- |
| 37806 | M15 240022 | Neisseria meningitidis | W | ST-11 complex | No value | UK | SV-2 | Opc- |
| 37807 | M15 240024 | Neisseria meningitidis | B | ST-162 complex | No value | UK | SV-1 | Opc+ |
| 37808 | M15 240025 | Neisseria meningitidis | Y | ST-167 complex | No value | UK | SV-5 | Opc+ |
| 37809 | M15 240027 | Neisseria meningitidis | W | ST-11 complex | No value | UK | SV-2 | Opc- |
| 37810 | M15 240028 | Neisseria meningitidis | Y | ST-167 complex | No value | UK | SV-5 | Opc+ |
| 37811 | M15 240029 | Neisseria meningitidis | W | ST-11 complex | No value | UK | SV-2 | Opc- |
| 37812 | M15 240030 | Neisseria meningitidis | Y | ST-23 complex | No value | UK | SV-11 | Opc+ |
| 37813 | M15 240031 | Neisseria meningitidis | Y | ST-167 complex | No value | UK | SV-5 | Opc+ |
| 37814 | M15 240034 | Neisseria meningitidis | B | ST-41/44 complex | No value | UK | SV-2 | Opc+ |
| 37815 | M15 240035 | Neisseria meningitidis | C | ST-11 complex | No value | UK | SV-2 | Opc- |
| 37816 | M15 240036 | Neisseria meningitidis | C | ST-11 complex | No value | UK | SV-2 | Opc- |
| 37817 | M15 240037 | Neisseria meningitidis | B | No value | No value | UK | SV-7 | Opc- |
| 37818 | M15 240038 | Neisseria meningitidis | B | ST-213 complex | No value | UK | SV-12 | Opc- |
| 37819 | M15 240039 | Neisseria meningitidis | B | ST-269 complex | No value | UK | SV-1 | Opc+ |
| 37820 | M15 240040 | Neisseria meningitidis | Y | ST-23 complex | No value | UK | SV-11 | Opc+ |
| 37821 | M15 240041 | Neisseria meningitidis | W | ST-11 complex | No value | UK | SV-2 | Opc- |
| 37822 | M15 240042 | Neisseria meningitidis | W | ST-11 complex | No value | UK | SV-2 | Opc- |
| 37823 | M15 240043 | Neisseria meningitidis | B | ST-32 complex | No value | UK | SV-1 | Opc+ |
| 37824 | M15 240044 | Neisseria meningitidis | B | ST-213 complex | No value | UK | SV-12 | Opc- |
| 37825 | M15 240045 | Neisseria meningitidis | Y | No value | No value | UK | SV-11 | Opc+ |
| 37826 | M15 240046 | Neisseria meningitidis | B | ST-213 complex | No value | UK | SV-12 | Opc- |

| | | | | | | | | |
|---|---|---|---|---|---|---|---|---|
| 37828 | M15 240048 | Neisseria meningitidis | W | ST-11 complex | No value | UK | SV-2 | Opc- |
| 37829 | M15 240049 | Neisseria meningitidis | B | ST-269 complex | No value | UK | SV-8 | Opc+ |
| 37830 | M15 240050 | Neisseria meningitidis | B | ST-269 complex | No value | UK | SV-8 | Opc+ |
| 37831 | M15 240051 | Neisseria meningitidis | B | ST-162 complex | No value | UK | SV-1 | Opc+ |
| 37832 | M15 240053 | Neisseria meningitidis | B | ST-103 complex | No value | UK | SV-5 | Opc+ |
| 37833 | M15 240054 | Neisseria meningitidis | Y | ST-23 complex | No value | UK | SV-11 | Opc+ |
| 37834 | M15 240055 | Neisseria meningitidis | W | ST-11 complex | No value | UK | SV-2 | Opc- |
| 37835 | M15 240056 | Neisseria meningitidis | B | ST-269 complex | No value | UK | SV-8 | Opc+ |
| 37836 | M15 240057 | Neisseria meningitidis | Y | ST-23 complex | No value | UK | SV-11 | Opc+ |
| 37837 | M15 240058 | Neisseria meningitidis | B | ST-269 complex | No value | UK | SV-8 | Opc+ |
| 37838 | M15 240059 | Neisseria meningitidis | Y | ST-23 complex | No value | UK | SV-11 | Opc+ |
| 37839 | M15 240060 | Neisseria meningitidis | B | No value | No value | UK | SV-5 | Opc+ |
| 37840 | M15 240061 | Neisseria meningitidis | W | ST-22 complex | No value | UK | SV-12 | Opc+ |
| 37841 | M15 240062 | Neisseria meningitidis | Y | ST-23 complex | No value | UK | SV-11 | Opc+ |
| 37842 | M15 240063 | Neisseria meningitidis | B | ST-41/44 complex | No value | UK | SV-6 | Opc+ |
| 37843 | M15 240064 | Neisseria meningitidis | Y | ST-23 complex | No value | UK | SV-11 | Opc+ |
| 37844 | M15 240065 | Neisseria meningitidis | B | ST-32 complex | No value | UK | SV-1 | Opc+ |
| 37845 | M15 240066 | Neisseria meningitidis | W | ST-11 complex | No value | UK | SV-2 | Opc- |
| 37846 | M15 240067 | Neisseria meningitidis | B | ST-213 complex | No value | UK | SV-12 | Opc- |
| 37847 | M15 240068 | Neisseria meningitidis | Y | ST-23 complex | No value | UK | SV-11 | Opc+ |
| 37848 | M15 240069 | Neisseria meningitidis | B | ST-32 complex | No value | UK | SV-1 | Opc+ |
| 37849 | M15 240071 | Neisseria meningitidis | B | ST-41/44 complex | No value | UK | SV-6 | Opc+ |
| 37850 | M15 240072 | Neisseria meningitidis | Y | ST-23 complex | No value | UK | SV-11 | Opc+ |
| 37851 | M15 240073 | Neisseria meningitidis | Y | ST-167 complex | No value | UK | SV-5 | Opc+ |
| 37852 | M15 240074 | Neisseria meningitidis | B | ST-41/44 complex | No value | UK | SV-2 | Opc+ |
| 37853 | M15 240077 | Neisseria meningitidis | Y | ST-23 complex | No value | UK | SV-11 | Opc+ |
| 37854 | M15 240078 | Neisseria meningitidis | W | ST-11 complex | No value | UK | SV-2 | Opc- |
| 37855 | M15 240079 | Neisseria meningitidis | W | No value | No value | UK | SV-2 | Opc- |
| 37856 | M15 240080 | Neisseria meningitidis | W | ST-11 complex | No value | UK | SV-2 | Opc- |
| 37857 | M15 240081 | Neisseria meningitidis | W | ST-11 complex | No value | UK | SV-2 | Opc- |
| 37858 | M15 240082 | Neisseria meningitidis | Y | ST-23 complex | No value | UK | SV-11 | Opc+ |
| 37859 | M15 240083 | Neisseria meningitidis | Y | ST-23 complex | No value | UK | SV-11 | Opc+ |
| 37860 | M15 240085 | Neisseria meningitidis | W | ST-11 complex | No value | UK | SV-2 | Opc- |
| 37861 | M15 240089 | Neisseria meningitidis | W | ST-11 complex | No value | UK | SV-2 | Opc- |
| 37862 | M15 240091 | Neisseria meningitidis | W | ST-11 complex | No value | UK | SV-2 | Opc- |
| 37863 | M15 240094 | Neisseria meningitidis | Y | ST-174 complex | No value | UK | SV-1 | Opc+ |
| 37864 | M15 240095 | Neisseria meningitidis | W | ST-22 complex | No value | UK | SV-12 | Opc+ |
| 37865 | M15 240096 | Neisseria meningitidis | B | ST-41/44 complex | No value | UK | SV-12 | Opc+ |
| 37866 | M15 240097 | Neisseria meningitidis | W | ST-11 complex | No value | UK | SV-2 | Opc- |
| 37867 | M15 240098 | Neisseria meningitidis | Y | ST-23 complex | No value | UK | SV-11 | Opc+ |
| 37868 | M15 240099 | Neisseria meningitidis | W | ST-11 complex | No value | UK | SV-2 | Opc- |
| 37869 | M15 240100 | Neisseria meningitidis | Y | ST-23 complex | No value | UK | SV-11 | Opc+ |
| 37870 | M15 240101 | Neisseria meningitidis | W | ST-11 complex | No value | UK | SV-2 | Opc- |
| 37871 | M15 240102 | Neisseria meningitidis | B | ST-269 complex | No value | UK | SV-1 | Opc+ |
| 37872 | M15 240103 | Neisseria meningitidis | B | ST-461 complex | No value | UK | SV-1 | Opc- |
| 37873 | M15 240105 | Neisseria meningitidis | W | ST-11 complex | No value | UK | SV-2 | Opc- |
| 37874 | M15 240106 | Neisseria meningitidis | B | ST-41/44 complex | No value | UK | SV-2 | Opc+ |
| 37875 | M15 240107 | Neisseria meningitidis | W | ST-11 complex | No value | UK | SV-2 | Opc- |
| 37876 | M15 240109 | Neisseria meningitidis | B | ST-269 complex | No value | UK | SV-1 | Opc+ |
| 37877 | M15 240110 | Neisseria meningitidis | B | ST-162 complex | No value | UK | SV-1 | Opc+ |
| 37878 | M15 240111 | Neisseria meningitidis | B | ST-213 complex | No value | UK | SV-12 | Opc- |
| 37879 | M15 240112 | Neisseria meningitidis | W | ST-11 complex | No value | UK | SV-2 | Opc- |
| 37880 | M15 240113 | Neisseria meningitidis | B | ST-32 complex | No value | UK | SV-1 | Opc+ |
| 37881 | M15 240114 | Neisseria meningitidis | W | ST-11 complex | No value | UK | SV-2 | Opc- |
| 37882 | M15 240115 | Neisseria meningitidis | W | ST-11 complex | No value | UK | SV-2 | Opc- |
| 37883 | M15 240116 | Neisseria meningitidis | W | ST-11 complex | No value | UK | SV-2 | Opc- |
| 37884 | M15 240118 | Neisseria meningitidis | B | ST-41/44 complex | No value | UK | SV-2 | Opc+ |
| 37885 | M15 240119 | Neisseria meningitidis | Y | ST-23 complex | No value | UK | SV-11 | Opc+ |
| 37886 | M15 240120 | Neisseria meningitidis | B | ST-213 complex | No value | UK | SV-12 | Opc- |
| 37887 | M15 240121 | Neisseria meningitidis | B | No value | No value | UK | SV-11 | Opc+ |
| 37888 | M15 240122 | Neisseria meningitidis | B | ST-269 complex | No value | UK | SV-1 | Opc+ |
| 37889 | M15 240126 | Neisseria meningitidis | Y | ST-23 complex | No value | UK | SV-11 | Opc+ |
| 37890 | M15 240127 | Neisseria meningitidis | W | ST-11 complex | No value | UK | SV-2 | Opc- |
| 37891 | M15 240128 | Neisseria meningitidis | W | ST-11 complex | No value | UK | SV-2 | Opc- |
| 37892 | M15 240129 | Neisseria meningitidis | NG | ST-23 complex | No value | UK | SV-11 | Opc+ |
| 37893 | M15 240130 | Neisseria meningitidis | W | ST-11 complex | No value | UK | SV-2 | Opc- |
| 37894 | M15 240131 | Neisseria meningitidis | C | ST-11 complex | No value | UK | SV-2 | Opc- |
| 37895 | M15 240132 | Neisseria meningitidis | Y | ST-23 complex | No value | UK | SV-11 | Opc+ |
| 37896 | M15 240133 | Neisseria meningitidis | B | ST-60 complex | No value | UK | SV-11 | Opc+ |
| 37897 | M15 240134 | Neisseria meningitidis | Y | ST-23 complex | No value | UK | SV-11 | Opc+ |
| 37898 | M15 240135 | Neisseria meningitidis | B | ST-41/44 complex | No value | UK | SV-5 | Opc- |
| 37899 | M15 240136 | Neisseria meningitidis | Y | ST-23 complex | No value | UK | SV-11 | Opc+ |
| 37900 | M15 240139 | Neisseria meningitidis | B | No value | No value | UK | SV-5 | Opc+ |
| 37901 | M15 240140 | Neisseria meningitidis | B | ST-213 complex | No value | UK | SV-12 | Opc- |

| | | | | | | | | |
|---|---|---|---|---|---|---|---|---|
| 37902 | M15 240141 | Neisseria meningitidis | W | ST-11 complex | No value | UK | SV-2 | Opc- |
| 37903 | M15 240142 | Neisseria meningitidis | B | ST-32 complex | No value | UK | SV-1 | Opc+ |
| 37904 | M15 240144 | Neisseria meningitidis | W | ST-11 complex | No value | UK | SV-2 | Opc- |
| 37905 | M15 240145 | Neisseria meningitidis | B | ST-213 complex | No value | UK | SV-12 | Opc- |
| 37906 | M15 240147 | Neisseria meningitidis | B | ST-41/44 complex | No value | UK | SV-6 | Opc+ |
| 37907 | M15 240148 | Neisseria meningitidis | W | ST-11 complex | No value | UK | SV-2 | Opc- |
| 37908 | M15 240149 | Neisseria meningitidis | B | No value | No value | UK | SV-8 | Opc+ |
| 37909 | M15 240150 | Neisseria meningitidis | B | ST-213 complex | No value | UK | SV-12 | Opc- |
| 37910 | M15 240151 | Neisseria meningitidis | W | ST-11 complex | No value | UK | SV-2 | Opc- |
| 37911 | M15 240152 | Neisseria meningitidis | Y | ST-23 complex | No value | UK | SV-11 | Opc+ |
| 37912 | M15 240154 | Neisseria meningitidis | B | ST-35 complex | No value | UK | SV-2 | Opc+ |
| 37913 | M15 240155 | Neisseria meningitidis | B | ST-41/44 complex | No value | UK | SV-2 | Opc+ |
| 37914 | M15 240157 | Neisseria meningitidis | W | ST-23 complex | No value | UK | SV-11 | Opc+ |
| 37915 | M15 240158 | Neisseria meningitidis | W | ST-11 complex | No value | UK | SV-2 | Opc- |
| 37916 | M15 240159 | Neisseria meningitidis | B | ST-41/44 complex | No value | UK | SV-2 | Opc+ |
| 37917 | M15 240160 | Neisseria meningitidis | Y | ST-23 complex | No value | UK | SV-11 | Opc+ |
| 37918 | M15 240161 | Neisseria meningitidis | C | ST-11 complex | No value | UK | SV-2 | Opc- |
| 37919 | M15 240168 | Neisseria meningitidis | B | No value | No value | UK | SV-8 | Opc+ |
| 37920 | M15 240169 | Neisseria meningitidis | B | ST-269 complex | No value | UK | SV-1 | Opc+ |
| 37921 | M15 240171 | Neisseria meningitidis | B | ST-41/44 complex | No value | UK | SV-6 | Opc+ |
| 37922 | M15 240172 | Neisseria meningitidis | W | ST-11 complex | No value | UK | SV-2 | Opc- |
| 37923 | M15 240174 | Neisseria meningitidis | Y | ST-23 complex | No value | UK | SV-11 | Opc+ |
| 37924 | M15 240175 | Neisseria meningitidis | B | ST-269 complex | No value | UK | SV-8 | Opc+ |
| 37925 | M15 240176 | Neisseria meningitidis | B | ST-41/44 complex | No value | UK | SV-2 | Opc+ |
| 37926 | M15 240177 | Neisseria meningitidis | Y | ST-23 complex | No value | UK | SV-11 | Opc+ |
| 37927 | M15 240178 | Neisseria meningitidis | B | ST-35 complex | No value | UK | SV-12 | Opc+ |
| 37928 | M15 240179 | Neisseria meningitidis | B | ST-269 complex | No value | UK | SV-1 | Opc+ |
| 37929 | M15 240180 | Neisseria meningitidis | B | ST-162 complex | No value | UK | SV-1 | Opc+ |
| 37930 | M15 240181 | Neisseria meningitidis | W | ST-11 complex | No value | UK | SV-2 | Opc- |
| 37931 | M15 240182 | Neisseria meningitidis | B | ST-269 complex | No value | UK | SV-1 | Opc+ |
| 37932 | M15 240183 | Neisseria meningitidis | B | ST-41/44 complex | No value | UK | SV-6 | Opc+ |
| 37933 | M15 240185 | Neisseria meningitidis | W | ST-11 complex | No value | UK | SV-2 | Opc- |
| 37934 | M15 240187 | Neisseria meningitidis | W | ST-11 complex | No value | UK | SV-2 | Opc- |
| 37935 | M15 240188 | Neisseria meningitidis | B | ST-213 complex | No value | UK | SV-12 | Opc- |
| 37936 | M15 240189 | Neisseria meningitidis | W | ST-11 complex | No value | UK | SV-2 | Opc- |
| 37937 | M15 240190 | Neisseria meningitidis | Y | ST-11 complex | No value | UK | SV-2 | Opc- |
| 37938 | M15 240191 | Neisseria meningitidis | Y | ST-23 complex | No value | UK | SV-11 | Opc+ |
| 37939 | M15 240192 | Neisseria meningitidis | Y | ST-23 complex | No value | UK | SV-11 | Opc+ |
| 37940 | M15 240193 | Neisseria meningitidis | W | ST-11 complex | No value | UK | SV-2 | Opc- |
| 37941 | M15 240194 | Neisseria meningitidis | Y | ST-23 complex | No value | UK | SV-11 | Opc+ |
| 37942 | M15 240195 | Neisseria meningitidis | W | ST-11 complex | No value | UK | SV-2 | Opc- |
| 37943 | M15 240196 | Neisseria meningitidis | B | ST-269 complex | No value | UK | SV-1 | Opc+ |
| 37944 | M15 240199 | Neisseria meningitidis | Y | ST-23 complex | No value | UK | SV-11 | Opc+ |
| 37945 | M15 240201 | Neisseria meningitidis | B | ST-41/44 complex | No value | UK | SV-2 | Opc+ |
| 37946 | M15 240202 | Neisseria meningitidis | NG | No value | No value | UK | SV-5 | Opc- |
| 37947 | M15 240203 | Neisseria meningitidis | B | ST-364 complex | No value | UK | SV-1 | Opc- |
| 37948 | M15 240204 | Neisseria meningitidis | C | ST-269 complex | No value | UK | SV-1 | Opc+ |
| 37949 | M15 240205 | Neisseria meningitidis | C | ST-11 complex | No value | UK | SV-2 | Opc- |
| 37950 | M15 240206 | Neisseria meningitidis | B | ST-213 complex | No value | UK | SV-12 | Opc- |
| 37951 | M15 240207 | Neisseria meningitidis | W | ST-11 complex | No value | UK | SV-2 | Opc- |
| 37952 | M15 240208 | Neisseria meningitidis | B | ST-269 complex | No value | UK | SV-8 | Opc+ |
| 37953 | M15 240209 | Neisseria meningitidis | B | ST-41/44 complex | No value | UK | SV-2 | Opc+ |
| 37954 | M15 240211 | Neisseria meningitidis | B | ST-269 complex | No value | UK | SV-8 | Opc+ |
| 37955 | M15 240212 | Neisseria meningitidis | Y | ST-23 complex | No value | UK | SV-11 | Opc+ |
| 37956 | M15 240213 | Neisseria meningitidis | B | ST-213 complex | No value | UK | SV-12 | Opc- |
| 37957 | M15 240215 | Neisseria meningitidis | B | ST-213 complex | No value | UK | SV-12 | Opc- |
| 37958 | M15 240216 | Neisseria meningitidis | W | ST-11 complex | No value | UK | SV-2 | Opc- |
| 37959 | M15 240219 | Neisseria meningitidis | B | ST-461 complex | No value | UK | SV-1 | Opc- |
| 37960 | M15 240220 | Neisseria meningitidis | B | ST-41/44 complex | No value | UK | SV-2 | Opc+ |
| 37961 | M15 240221 | Neisseria meningitidis | W | ST-11 complex | No value | UK | SV-2 | Opc- |
| 37962 | M15 240222 | Neisseria meningitidis | W | No value | No value | UK | SV-2 | Opc- |
| 37963 | M15 240223 | Neisseria meningitidis | Y | ST-23 complex | No value | UK | SV-11 | Opc+ |
| 37964 | M15 240224 | Neisseria meningitidis | W | No value | No value | UK | SV-7 | Opc+ |
| 37965 | M15 240225 | Neisseria meningitidis | B | ST-41/44 complex | No value | UK | SV-2 | Opc+ |
| 37966 | M15 240226 | Neisseria meningitidis | B | ST-35 complex | No value | UK | SV-12 | Opc+ |
| 37967 | M15 240227 | Neisseria meningitidis | C | ST-11 complex | No value | UK | SV-2 | Opc- |
| 37968 | M15 240229 | Neisseria meningitidis | B | ST-35 complex | No value | UK | SV-12 | Opc+ |
| 37969 | M15 240231 | Neisseria meningitidis | B | ST-213 complex | No value | UK | SV-12 | Opc- |
| 37970 | M15 240232 | Neisseria meningitidis | B | ST-41/44 complex | No value | UK | SV-2 | Opc+ |
| 37971 | M15 240233 | Neisseria meningitidis | B | ST-32 complex | No value | UK | SV-1 | Opc+ |
| 37972 | M15 240235 | Neisseria meningitidis | B | ST-269 complex | No value | UK | SV-8 | Opc+ |
| 37973 | M15 240236 | Neisseria meningitidis | B | No value | No value | UK | SV-16 | Opc+ |
| 37974 | M15 240239 | Neisseria meningitidis | C | ST-269 complex | No value | UK | SV-1 | Opc+ |
| 37975 | M15 240240 | Neisseria meningitidis | B | ST-269 complex | No value | UK | SV-8 | Opc+ |

| | | | | | | | | |
|---|---|---|---|---|---|---|---|---|
| 37976 | M15 240241 | Neisseria meningitidis | B | ST-269 complex | No value | UK | SV-1 | Opc+ |
| 37977 | M15 240242 | Neisseria meningitidis | W | ST-11 complex | No value | UK | SV-2 | Opc- |
| 37978 | M15 240244 | Neisseria meningitidis | W | ST-11 complex | No value | UK | SV-2 | Opc- |
| 37979 | M15 240246 | Neisseria meningitidis | Y | ST-23 complex | No value | UK | SV-11 | Opc+ |
| 37980 | M15 240247 | Neisseria meningitidis | Y | ST-23 complex | No value | UK | SV-11 | Opc+ |
| 37981 | M15 240248 | Neisseria meningitidis | W | ST-11 complex | No value | UK | SV-2 | Opc- |
| 37982 | M15 240250 | Neisseria meningitidis | Y | ST-23 complex | No value | UK | SV-11 | Opc+ |
| 37983 | M15 240251 | Neisseria meningitidis | W | ST-11 complex | No value | UK | SV-2 | Opc- |
| 37984 | M15 240252 | Neisseria meningitidis | B | ST-35 complex | No value | UK | SV-16 | Opc+ |
| 37985 | M15 240253 | Neisseria meningitidis | B | ST-269 complex | No value | UK | SV-8 | Opc+ |
| 37986 | M15 240256 | Neisseria meningitidis | W | ST-11 complex | No value | UK | SV-2 | Opc- |
| 37987 | M15 240257 | Neisseria meningitidis | W | ST-11 complex | No value | UK | SV-2 | Opc- |
| 37988 | M15 240258 | Neisseria meningitidis | W | ST-11 complex | No value | UK | SV-2 | Opc- |
| 37989 | M15 240259 | Neisseria meningitidis | B | ST-269 complex | No value | UK | SV-1 | Opc+ |
| 37990 | M15 240260 | Neisseria meningitidis | W | ST-11 complex | No value | UK | SV-2 | Opc- |
| 37991 | M15 240262 | Neisseria meningitidis | B | ST-41/44 complex | No value | UK | SV-2 | Opc+ |
| 37992 | M15 240263 | Neisseria meningitidis | W | ST-11 complex | No value | UK | SV-2 | Opc- |
| 37993 | M15 240265 | Neisseria meningitidis | Y | ST-23 complex | No value | UK | SV-11 | Opc+ |
| 37994 | M15 240270 | Neisseria meningitidis | B | No value | No value | UK | SV-11 | Opc+ |
| 37995 | M15 240271 | Neisseria meningitidis | W | ST-11 complex | No value | UK | SV-2 | Opc- |
| 37996 | M15 240273 | Neisseria meningitidis | B | No value | No value | UK | SV-8 | Opc+ |
| 37997 | M15 240276 | Neisseria meningitidis | B | ST-213 complex | No value | UK | SV-12 | Opc- |
| 37998 | M15 240277 | Neisseria meningitidis | W | ST-11 complex | No value | UK | SV-2 | Opc- |
| 37999 | M15 240278 | Neisseria meningitidis | W | ST-11 complex | No value | UK | SV-2 | Opc- |
| 38000 | M15 240279 | Neisseria meningitidis | B | ST-32 complex | No value | UK | SV-1 | Opc+ |
| 38001 | M15 240280 | Neisseria meningitidis | W | ST-11 complex | No value | UK | SV-2 | Opc- |
| 38002 | M15 240281 | Neisseria meningitidis | B | No value | No value | UK | SV-15 | Opc- |
| 38003 | M15 240282 | Neisseria meningitidis | W | ST-11 complex | No value | UK | SV-2 | Opc- |
| 38004 | M15 240284 | Neisseria meningitidis | W | ST-11 complex | No value | UK | SV-2 | Opc- |
| 38005 | M15 240285 | Neisseria meningitidis | Y | ST-23 complex | No value | UK | SV-11 | Opc+ |
| 38006 | M15 240286 | Neisseria meningitidis | B | ST-269 complex | No value | UK | SV-8 | Opc+ |
| 38007 | M15 240287 | Neisseria meningitidis | W | ST-11 complex | No value | UK | SV-2 | Opc- |
| 38008 | M15 240288 | Neisseria meningitidis | W | ST-11 complex | No value | UK | SV-2 | Opc- |
| 38009 | M15 240289 | Neisseria meningitidis | B | ST-41/44 complex | No value | UK | SV-6 | Opc+ |
| 38010 | M15 240290 | Neisseria meningitidis | Y | ST-23 complex | No value | UK | SV-11 | Opc+ |
| 38011 | M15 240291 | Neisseria meningitidis | W | ST-11 complex | No value | UK | SV-2 | Opc- |
| 38012 | M15 240292 | Neisseria meningitidis | Y | ST-23 complex | No value | UK | SV-11 | Opc+ |
| 38013 | M15 240294 | Neisseria meningitidis | Y | ST-23 complex | No value | UK | SV-11 | Opc+ |
| 38014 | M15 240295 | Neisseria meningitidis | Y | ST-23 complex | No value | UK | SV-11 | Opc+ |
| 38015 | M15 240296 | Neisseria meningitidis | B | No value | No value | UK | SV-11 | Opc- |
| 38016 | M15 240297 | Neisseria meningitidis | B | ST-269 complex | No value | UK | SV-8 | Opc+ |
| 38017 | M15 240300 | Neisseria meningitidis | W | ST-11 complex | No value | UK | SV-2 | Opc- |
| 38018 | M15 240301 | Neisseria meningitidis | W | ST-11 complex | No value | UK | SV-2 | Opc- |
| 38019 | M15 240302 | Neisseria meningitidis | B | ST-213 complex | No value | UK | SV-12 | Opc- |
| 38020 | M15 240304 | Neisseria meningitidis | B | No value | No value | UK | SV-1 | Opc+ |
| 38021 | M15 240305 | Neisseria meningitidis | Y | ST-23 complex | No value | UK | SV-11 | Opc+ |
| 38022 | M15 240307 | Neisseria meningitidis | B | No value | No value | UK | SV-7 | Opc- |
| 38023 | M15 240309 | Neisseria meningitidis | B | ST-41/44 complex | No value | UK | SV-2 | Opc+ |
| 38024 | M15 240310 | Neisseria meningitidis | W | ST-11 complex | No value | UK | SV-2 | Opc- |
| 38025 | M15 240312 | Neisseria meningitidis | B | ST-41/44 complex | No value | UK | SV-10 | Opc+ |
| 38026 | M15 240313 | Neisseria meningitidis | B | ST-41/44 complex | No value | UK | SV-6 | Opc+ |
| 38027 | M15 240316 | Neisseria meningitidis | W | ST-11 complex | No value | UK | SV-2 | Opc- |
| 38028 | M15 240319 | Neisseria meningitidis | Y | ST-23 complex | No value | UK | SV-11 | Opc+ |
| 38029 | M15 240320 | Neisseria meningitidis | W | No value | No value | UK | SV-2 | Opc- |
| 38030 | M15 240321 | Neisseria meningitidis | B | ST-269 complex | No value | UK | SV-8 | Opc+ |
| 38031 | M15 240322 | Neisseria meningitidis | B | ST-41/44 complex | No value | UK | SV-2 | Opc+ |
| 38032 | M15 240323 | Neisseria meningitidis | B | No value | No value | UK | SV-12 | Opc+ |
| 38033 | M15 240325 | Neisseria meningitidis | B | ST-269 complex | No value | UK | SV-8 | Opc+ |
| 38034 | M15 240326 | Neisseria meningitidis | W | ST-11 complex | No value | UK | SV-2 | Opc- |
| 38035 | M15 240327 | Neisseria meningitidis | W | No value | No value | UK | SV-2 | Opc- |
| 38036 | M15 240328 | Neisseria meningitidis | B | ST-41/44 complex | No value | UK | SV-6 | Opc+ |
| 38037 | M15 240329 | Neisseria meningitidis | W | ST-11 complex | No value | UK | SV-2 | Opc- |
| 38038 | M15 240332 | Neisseria meningitidis | W | ST-11 complex | No value | UK | SV-2 | Opc- |
| 38039 | M15 240333 | Neisseria meningitidis | B | ST-41/44 complex | No value | UK | SV-2 | Opc+ |
| 38040 | M15 240553 | Neisseria meningitidis | C | ST-32 complex | No value | UK | SV-1 | Opc+ |
| 38041 | M15 240075 | Neisseria meningitidis | B | ST-41/44 complex | No value | UK | SV-2 | Opc+ |
| 38042 | M15 240076 | Neisseria meningitidis | B | ST-269 complex | No value | UK | SV-8 | Opc+ |
| 38043 | M15 240084 | Neisseria meningitidis | B | ST-269 complex | No value | UK | SV-8 | Opc+ |
| 38044 | M15 240087 | Neisseria meningitidis | B | ST-41/44 complex | No value | UK | SV-2 | Opc+ |
| 38045 | M15 240088 | Neisseria meningitidis | B | ST-269 complex | No value | UK | SV-1 | Opc+ |
| 38046 | M15 240090 | Neisseria meningitidis | B | ST-269 complex | No value | UK | SV-8 | Opc+ |
| 38047 | M15 240092 | Neisseria meningitidis | B | ST-213 complex | No value | UK | SV-6 | Opc- |
| 38048 | M15 240237 | Neisseria meningitidis | NG | ST-198 complex | No value | UK | SV-4 | Opc+ |
| 38049 | M15 240334 | Neisseria meningitidis | W | ST-11 complex | No value | UK | SV-2 | Opc- |

| | | | | | | | | |
|---|---|---|---|---|---|---|---|---|
| 38050 | M15 240335 | Neisseria meningitidis | B | ST-213 complex | No value | UK | SV-12 | Opc- |
| 38051 | M15 240336 | Neisseria meningitidis | B | ST-461 complex | No value | UK | SV-1 | Opc- |
| 38052 | M15 240337 | Neisseria meningitidis | B | ST-41/44 complex | No value | UK | SV-2 | Opc+ |
| 38053 | M15 240352 | Neisseria meningitidis | Y | ST-23 complex | No value | UK | SV-11 | Opc+ |
| 38054 | M15 240354 | Neisseria meningitidis | Y | No value | No value | UK | SV-11 | Opc+ |
| 38055 | M15 240355 | Neisseria meningitidis | C | ST-32 complex | No value | UK | SV-1 | Opc+ |
| 38056 | M15 240356 | Neisseria meningitidis | B | ST-41/44 complex | No value | UK | SV-6 | Opc+ |
| 38057 | M15 240357 | Neisseria meningitidis | B | ST-41/44 complex | No value | UK | SV-2 | Opc+ |
| 38058 | M15 240373 | Neisseria meningitidis | B | ST-269 complex | No value | UK | SV-8 | Opc+ |
| 38059 | M15 240375 | Neisseria meningitidis | W | ST-11 complex | No value | UK | SV-2 | Opc- |
| 38060 | M15 240376 | Neisseria meningitidis | W | ST-11 complex | No value | UK | SV-2 | Opc- |
| 38061 | M15 240378 | Neisseria meningitidis | W | ST-22 complex | No value | UK | SV-12 | Opc+ |
| 38062 | M15 240379 | Neisseria meningitidis | B | No value | No value | UK | SV-16 | Opc+ |
| 38063 | M15 240380 | Neisseria meningitidis | W | ST-11 complex | No value | UK | SV-2 | Opc- |
| 38064 | M15 240381 | Neisseria meningitidis | W | ST-11 complex | No value | UK | SV-2 | Opc- |
| 38065 | M15 240382 | Neisseria meningitidis | W | ST-11 complex | No value | UK | SV-2 | Opc- |
| 38066 | M15 240383 | Neisseria meningitidis | W | ST-11 complex | No value | UK | SV-2 | Opc- |
| 38067 | M15 240384 | Neisseria meningitidis | W | ST-11 complex | No value | UK | SV-2 | Opc- |
| 38068 | M15 240385 | Neisseria meningitidis | B | ST-269 complex | No value | UK | SV-1 | Opc+ |
| 38069 | M15 240386 | Neisseria meningitidis | B | ST-461 complex | No value | UK | SV-1 | Opc- |
| 38070 | M15 240388 | Neisseria meningitidis | B | ST-461 complex | No value | UK | SV-1 | Opc- |
| 38071 | M15 240422 | Neisseria meningitidis | Y | No value | No value | UK | SV-5 | Opc+ |
| 38072 | M15 240423 | Neisseria meningitidis | B | ST-162 complex | No value | UK | SV-1 | Opc+ |
| 38073 | M15 240424 | Neisseria meningitidis | B | ST-269 complex | No value | UK | SV-1 | Opc+ |
| 38074 | M15 240425 | Neisseria meningitidis | B | ST-32 complex | No value | UK | SV-1 | Opc+ |
| 38075 | M15 240437 | Neisseria meningitidis | B | ST-269 complex | No value | UK | SV-8 | Opc+ |
| 38076 | M15 240438 | Neisseria meningitidis | W | ST-11 complex | No value | UK | SV-2 | Opc- |
| 38077 | M15 240439 | Neisseria meningitidis | B | ST-41/44 complex | No value | UK | SV-2 | Opc+ |
| 38078 | M15 240440 | Neisseria meningitidis | Y | ST-23 complex | No value | UK | SV-11 | Opc+ |
| 38079 | M15 240441 | Neisseria meningitidis | W | ST-11 complex | No value | UK | SV-2 | Opc- |
| 38080 | M15 240443 | Neisseria meningitidis | B | No value | No value | UK | SV-6 | Opc+ |
| 38081 | M15 240445 | Neisseria meningitidis | B | ST-41/44 complex | No value | UK | SV-2 | Opc+ |
| 38082 | M15 240446 | Neisseria meningitidis | B | ST-35 complex | No value | UK | SV-12 | Opc+ |
| 38083 | M15 240459 | Neisseria meningitidis | B | ST-213 complex | No value | UK | SV-12 | Opc- |
| 38084 | M15 240460 | Neisseria meningitidis | B | ST-213 complex | No value | UK | SV-12 | Opc- |
| 38085 | M15 240461 | Neisseria meningitidis | W | ST-11 complex | No value | UK | SV-2 | Opc- |
| 38086 | M15 240463 | Neisseria meningitidis | B | ST-461 complex | No value | UK | SV-1 | Opc- |
| 38087 | M15 240464 | Neisseria meningitidis | Y | ST-23 complex | No value | UK | SV-11 | Opc+ |
| 38088 | M15 240465 | Neisseria meningitidis | W | ST-11 complex | No value | UK | SV-2 | Opc- |
| 38089 | M15 240466 | Neisseria meningitidis | B | ST-269 complex | No value | UK | SV-1 | Opc+ |
| 38090 | M15 240468 | Neisseria meningitidis | B | No value | No value | UK | SV-8 | Opc+ |
| 38091 | M15 240469 | Neisseria meningitidis | W | ST-22 complex | No value | UK | SV-12 | Opc+ |
| 38092 | M15 240472 | Neisseria meningitidis | B | ST-41/44 complex | No value | UK | SV-10 | Opc+ |
| 38093 | M15 240473 | Neisseria meningitidis | Y | No value | No value | UK | SV-11 | Opc+ |
| 38094 | M15 240474 | Neisseria meningitidis | B | ST-41/44 complex | No value | UK | SV-11 | Opc+ |
| 38095 | M15 240476 | Neisseria meningitidis | B | ST-269 complex | No value | UK | SV-8 | Opc+ |
| 38096 | M15 240477 | Neisseria meningitidis | W | ST-11 complex | No value | UK | SV-2 | Opc- |
| 38097 | M15 240496 | Neisseria meningitidis | B | ST-32 complex | No value | UK | SV-1 | Opc+ |
| 38098 | M15 240497 | Neisseria meningitidis | B | ST-41/44 complex | No value | UK | SV-2 | Opc+ |
| 38099 | M15 240498 | Neisseria meningitidis | Y | ST-23 complex | No value | UK | SV-11 | Opc+ |
| 38100 | M15 240499 | Neisseria meningitidis | B | ST-41/44 complex | No value | UK | SV-2 | Opc+ |
| 38101 | M15 240511 | Neisseria meningitidis | W | ST-11 complex | No value | UK | SV-2 | Opc- |
| 38102 | M15 240513 | Neisseria meningitidis | W | No value | No value | UK | SV-2 | Opc- |
| 38103 | M15 240514 | Neisseria meningitidis | B | ST-41/44 complex | No value | UK | SV-2 | Opc+ |
| 38104 | M15 240525 | Neisseria meningitidis | Y | ST-23 complex | No value | UK | SV-11 | Opc+ |
| 38105 | M15 240541 | Neisseria meningitidis | B | ST-269 complex | No value | UK | SV-8 | Opc+ |
| 38106 | M15 240554 | Neisseria meningitidis | W | ST-11 complex | No value | UK | SV-2 | Opc- |
| 38107 | M15 240555 | Neisseria meningitidis | B | ST-269 complex | No value | UK | SV-8 | Opc+ |
| 38108 | M15 240556 | Neisseria meningitidis | W | ST-11 complex | No value | UK | SV-2 | Opc- |
| 38109 | M15 240557 | Neisseria meningitidis | W | ST-11 complex | No value | UK | SV-2 | Opc- |
| 38110 | M15 240570 | Neisseria meningitidis | B | ST-269 complex | No value | UK | SV-1 | Opc+ |
| 38111 | M15 240572 | Neisseria meningitidis | NG | ST-198 complex | No value | UK | SV-4 | Opc+ |
| 38112 | M15 240573 | Neisseria meningitidis | B | ST-41/44 complex | No value | UK | SV-2 | Opc+ |
| 38113 | M15 240574 | Neisseria meningitidis | B | No value | No value | UK | SV-1 | Opc+ |
| 38114 | M15 240575 | Neisseria meningitidis | Y | ST-167 complex | No value | UK | SV-5 | Opc+ |
| 38115 | M15 240576 | Neisseria meningitidis | W | ST-11 complex | No value | UK | SV-2 | Opc- |
| 38116 | M15 240600 | Neisseria meningitidis | Y | ST-23 complex | No value | UK | SV-15 | Opc+ |
| 38117 | M15 240602 | Neisseria meningitidis | B | ST-162 complex | No value | UK | SV-1 | Opc+ |
| 38118 | M14 240523b | Neisseria meningitidis | Y | ST-23 complex | No value | UK | SV-11 | Opc+ |
| 38119 | M14 240380b | Neisseria meningitidis | B | ST-41/44 complex | No value | UK | SV-2 | Opc+ |
| 38120 | M14 240447b | Neisseria meningitidis | W | ST-11 complex | No value | UK | SV-2 | Opc- |
| 38121 | M14 240448b | Neisseria meningitidis | W | ST-11 complex | No value | UK | SV-2 | Opc- |
| 38122 | M13 240546 | Neisseria meningitidis | B | ST-213 complex | No value | UK | SV-12 | Opc- |
| 38123 | M13 240639 | Neisseria meningitidis | B | ST-213 complex | No value | UK | SV-12 | Opc- |

| | | | | | | | | |
|---|---|---|---|---|---|---|---|---|
| 38124 | M13 240642 | Neisseria meningitidis | B | ST-213 complex | No value | UK | SV-12 | Opc- |
| 38125 | M13 240671 | Neisseria meningitidis | B | ST-461 complex | No value | UK | SV-1 | Opc- |
| 38126 | M14 240449b | Neisseria meningitidis | B | ST-41/44 complex | No value | Ireland | SV-6 | Opc+ |
| 38127 | M14 240515 | Neisseria meningitidis | W | ST-11 complex | No value | UK | SV-2 | Opc- |
| 38128 | M15 240032 | Neisseria meningitidis | B | ST-41/44 complex | No value | UK | SV-10 | Opc+ |
| 38129 | M15 240033 | Neisseria meningitidis | Y | ST-174 complex | No value | UK | SV-1 | Opc+ |
| 38130 | M01 240007 | Neisseria meningitidis | B | ST-269 complex | No value | UK | SV-1 | Opc+ |
| 38131 | M01 240601 | Neisseria meningitidis | B | ST-269 complex | No value | UK | SV-1 | Opc+ |
| 38132 | M01 241271 | Neisseria meningitidis | B | ST-41/44 complex | No value | UK | SV-2 | Opc+ |
| 38133 | M01 241299 | Neisseria meningitidis | B | ST-41/44 complex | No value | UK | SV-10 | Opc+ |
| 38134 | M01 241451 | Neisseria meningitidis | B | ST-41/44 complex | No value | UK | SV-10 | Opc+ |
| 38135 | M01 241601 | Neisseria meningitidis | B | ST-41/44 complex | No value | UK | SV-6 | Opc+ |
| 38136 | M02 240039 | Neisseria meningitidis | B | ST-269 complex | No value | UK | SV-8 | Opc+ |
| 38137 | M02 240210 | Neisseria meningitidis | B | ST-269 complex | No value | UK | SV-1 | Opc+ |
| 38138 | M03 240151 | Neisseria meningitidis | B | ST-269 complex | No value | UK | SV-8 | Opc+ |
| 38139 | M03 240774 | Neisseria meningitidis | B | ST-41/44 complex | No value | UK | SV-10 | Opc+ |
| 38140 | M03 240823 | Neisseria meningitidis | B | ST-269 complex | No value | UK | SV-1 | Opc+ |
| 38141 | M04 240588 | Neisseria meningitidis | B | ST-213 complex | No value | UK | SV-12 | Opc- |
| 38142 | M04 240731 | Neisseria meningitidis | B | ST-41/44 complex | No value | UK | SV-6 | Opc+ |
| 38143 | M04 241215 | Neisseria meningitidis | B | ST-41/44 complex | No value | UK | SV-2 | Opc+ |
| 38144 | M04 241245 | Neisseria meningitidis | B | ST-212 complex | No value | UK | SV-11 | Opc+ |
| 38145 | M05 240300 | Neisseria meningitidis | B | ST-213 complex | No value | UK | SV-12 | Opc- |
| 38146 | M06 240928 | Neisseria meningitidis | B | No value | No value | UK | SV-1 | Opc+ |
| 38147 | M06 241112 | Neisseria meningitidis | B | ST-269 complex | No value | UK | SV-1 | Opc+ |
| 38148 | M15 240541B | Neisseria meningitidis | B | ST-269 complex | No value | UK | SV-8 | Opc+ |
| 38149 | M15 240541C | Neisseria meningitidis | B | ST-269 complex | No value | UK | SV-8 | Opc+ |
| 38150 | M15 240541D | Neisseria meningitidis | B | ST-269 complex | No value | UK | SV-8 | Opc+ |
| 38151 | M15 240605 | Neisseria meningitidis | W | ST-11 complex | No value | UK | SV-2 | Opc- |
| 38152 | 85Mo | Neisseria meningitidis | C | ST-11 complex | No value | Slovenia | SV-2 | Opc- |
| 38153 | 87Mo | Neisseria meningitidis | C | ST-11 complex | No value | Slovenia | SV-2 | Opc- |
| 38154 | 89Mo | Neisseria meningitidis | B | ST-11 complex | No value | Slovenia | SV-2 | Opc- |
| 38155 | 90Mo | Neisseria meningitidis | C | ST-11 complex | No value | Slovenia | SV-2 | Opc- |
| 38156 | 92Mo | Neisseria meningitidis | B | No value | No value | Slovenia | SV-1 | Opc- |
| 38157 | 93Mo | Neisseria meningitidis | Y | ST-23 complex | No value | Slovenia | SV-11 | Opc+ |
| 38158 | 94Mo | Neisseria meningitidis | B | ST-269 complex | No value | Slovenia | SV-8 | Opc+ |
| 38159 | 95Mo | Neisseria meningitidis | C | ST-11 complex | No value | Slovenia | SV-2 | Opc- |
| 38160 | 96Mo | Neisseria meningitidis | B | ST-41/44 complex | No value | Slovenia | SV-6 | Opc+ |
| 38161 | 97Mo | Neisseria meningitidis | C | ST-11 complex | No value | Slovenia | SV-2 | Opc- |
| 38176 | M37087 | Neisseria meningitidis | NG | No value | Carrier | Brazil | SV-5 | Opc- |
| 38177 | M37113 | Neisseria meningitidis | NG | ST-1136 complex | Carrier | Brazil | SV-2 | Opc+ |
| 38178 | M37959 | Neisseria meningitidis | NG | ST-1136 complex | Carrier | Brazil | SV-2 | Opc+ |
| 38179 | M37128 | Neisseria meningitidis | NG | ST-23 complex | Carrier | Brazil | SV-11 | Opc+ |
| 38180 | M37958 | Neisseria meningitidis | NG | ST-198 complex | Carrier | Brazil | SV-4 | Opc+ |
| 38221 | 107498 | Neisseria meningitidis | B | ST-41/44 complex | No value | Finland | SV-6 | Opc+ |
| 38222 | 107500 | Neisseria meningitidis | C | ST-269 complex | No value | Finland | SV-1 | Opc+ |
| 38223 | 107501 | Neisseria meningitidis | C | ST-32 complex | No value | Finland | SV-1 | Opc+ |
| 38224 | 107502 | Neisseria meningitidis | B | ST-41/44 complex | No value | Finland | SV-6 | Opc+ |
| 38225 | 107503 | Neisseria meningitidis | Y | ST-23 complex | No value | Finland | SV-11 | Opc+ |
| 38226 | 107504 | Neisseria meningitidis | B | ST-41/44 complex | No value | Finland | SV-6 | Opc+ |
| 38227 | 107505 | Neisseria meningitidis | NG | ST-198 complex | No value | Finland | SV-7 | Opc+ |
| 38228 | 107506 | Neisseria meningitidis | B | No value | No value | Finland | SV-1 | Opc- |
| 38229 | 107508 | Neisseria meningitidis | B | No value | No value | Finland | SV-1 | Opc- |
| 38230 | 107509 | Neisseria meningitidis | C | ST-32 complex | No value | Finland | SV-1 | Opc+ |
| 38231 | 107510 | Neisseria meningitidis | Y | ST-23 complex | No value | Finland | SV-11 | Opc+ |
| 38232 | 107511 | Neisseria meningitidis | C | ST-32 complex | No value | Finland | SV-1 | Opc+ |
| 38233 | 107512 | Neisseria meningitidis | W | ST-22 complex | No value | Finland | SV-12 | Opc+ |
| 38234 | 107513 | Neisseria meningitidis | B | No value | No value | Finland | SV-1 | Opc+ |
| 38305 | 88/03415 | Neisseria meningitidis | B | ST-41/44 complex | No value | UK | SV-1 | Opc+ |
| 38306 | 15.8705948 | Neisseria meningitidis | W | ST-11 complex | No value | UK | SV-2 | Opc- |
| 38307 | 15.8706012 | Neisseria meningitidis | W | ST-11 complex | No value | UK | SV-2 | Opc- |
| 38308 | 15.870609 | Neisseria meningitidis | W | ST-11 complex | No value | UK | SV-2 | Opc- |
| 38309 | 15.8706113 | Neisseria meningitidis | W | ST-11 complex | No value | UK | SV-2 | Opc- |
| 38520 | M15 240722 | Neisseria meningitidis | W | ST-11 complex | No value | UK | SV-2 | Opc- |
| 38600 | 1658 15 | Neisseria meningitidis | NG | ST-11 complex | No value | Niger | SV-2 | Opc- |
| 38601 | NEIS15-107 | Neisseria meningitidis | W | ST-11 complex | No value | Sweden | SV-2 | Opc- |
| 38602 | NEIS15-193 | Neisseria meningitidis | W | ST-11 complex | No value | Sweden | SV-2 | Opc- |
| 38603 | NEIS15-198 | Neisseria meningitidis | W | ST-11 complex | No value | Sweden | SV-2 | Opc- |
| 38604 | NEIS15-199 | Neisseria meningitidis | W | ST-11 complex | No value | Sweden | SV-2 | Opc- |
| 38605 | NEIS15-201 | Neisseria meningitidis | W | ST-11 complex | No value | Sweden | SV-2 | Opc- |
| 38606 | NEIS15-207 | Neisseria meningitidis | W | ST-11 complex | No value | Sweden | SV-2 | Opc- |
| 38607 | NEIS15-215 | Neisseria meningitidis | W | ST-11 complex | No value | Sweden | SV-2 | Opc- |
| 38608 | NEIS15-217 | Neisseria meningitidis | W | ST-11 complex | No value | Sweden | SV-2 | Opc- |
| 38609 | NEIS15-227 | Neisseria meningitidis | W | ST-11 complex | No value | Sweden | SV-2 | Opc- |
| 38610 | NEIS15-228 | Neisseria meningitidis | W | ST-11 complex | No value | Sweden | SV-2 | Opc- |

| | | | | | | | | |
|---|---|---|---|---|---|---|---|---|
| 38611 | NEIS15-229 | Neisseria meningitidis | W | ST-11 complex | No value | Sweden | SV-2 | Opc- |
| 38612 | NEIS15-230 | Neisseria meningitidis | W | ST-11 complex | No value | Sweden | SV-2 | Opc- |
| 38613 | NEIS15-236 | Neisseria meningitidis | W | ST-11 complex | No value | Sweden | SV-2 | Opc- |
| 38614 | NEIS15-244 | Neisseria meningitidis | W | ST-11 complex | No value | Sweden | SV-2 | Opc- |
| 38615 | NEIS15-303 | Neisseria meningitidis | W | ST-11 complex | No value | Sweden | SV-2 | Opc- |
| 38616 | 608-9901 | Neisseria meningitidis | W | ST-11 complex | No value | Sweden | SV-2 | Opc- |
| 38617 | 90-137 | Neisseria meningitidis | W | ST-11 complex | No value | Sweden | SV-2 | Opc- |
| 38618 | 90-7 | Neisseria meningitidis | W | ST-11 complex | No value | Sweden | SV-2 | Opc- |
| 38619 | 90-188 | Neisseria meningitidis | W | ST-11 complex | No value | Sweden | SV-2 | Opc- |
| 38620 | 90-410 | Neisseria meningitidis | W | ST-11 complex | No value | Sweden | SV-2 | Opc- |
| 38621 | 590-26 | Neisseria meningitidis | W | ST-60 complex | No value | Sweden | SV-11 | Opc+ |
| 38622 | 590-66 | Neisseria meningitidis | W | ST-11 complex | No value | Sweden | SV-2 | Opc- |
| 38690 | 1559000123 | Neisseria meningitidis | W | ST-11 complex | No value | Sweden | SV-2 | Opc- |
| 38691 | 1559000163 | Neisseria meningitidis | W | ST-11 complex | No value | Sweden | SV-2 | Opc- |
| 38692 | NEIS15-33 | Neisseria meningitidis | W | ST-11 complex | No value | Sweden | SV-2 | Opc- |
| 38693 | NEIS15-69 | Neisseria meningitidis | W | No value | No value | Sweden | SV-10 | Opc+ |
| 38694 | NEIS15-71 | Neisseria meningitidis | W | ST-11 complex | No value | Sweden | SV-2 | Opc- |
| 38837 | 2711 | Neisseria meningitidis | C | ST-334 complex | Endemic | Italy | SV-15 | Opc- |
| 38838 | 2717 | Neisseria meningitidis | C | ST-11 complex | Endemic | Italy | SV-2 | Opc- |
| 38839 | 2718 | Neisseria meningitidis | C | ST-11 complex | Endemic | Italy | SV-2 | Opc- |
| 38840 | 2722 | Neisseria meningitidis | C | ST-334 complex | Endemic | Italy | SV-15 | Opc- |
| 38841 | 2723 | Neisseria meningitidis | C | ST-11 complex | Endemic | Italy | SV-2 | Opc- |
| 38843 | 2406 | Neisseria meningitidis | NG | ST-334 complex | Endemic | Italy | SV-15 | Opc- |
| 38844 | 2413 | Neisseria meningitidis | NG | ST-334 complex | Endemic | Italy | SV-15 | Opc- |
| 38845 | 2422 | Neisseria meningitidis | NG | ST-334 complex | Endemic | Italy | SV-15 | Opc- |
| 38846 | 2443 | Neisseria meningitidis | NG | No value | Endemic | Italy | SV-1 | Opc+ |
| 38847 | 2460 | Neisseria meningitidis | NG | ST-11 complex | Endemic | Italy | SV-2 | Opc- |
| 38848 | 2461 | Neisseria meningitidis | NG | ST-11 complex | Endemic | Italy | SV-2 | Opc- |
| 38849 | 2462 | Neisseria meningitidis | NG | ST-11 complex | Endemic | Italy | SV-2 | Opc- |
| 38850 | 2468 | Neisseria meningitidis | NG | ST-334 complex | Endemic | Italy | SV-15 | Opc- |
| 38851 | 2476 | Neisseria meningitidis | NG | ST-11 complex | Endemic | Italy | SV-2 | Opc- |
| 38852 | 2479 | Neisseria meningitidis | NG | ST-231 complex | Endemic | Italy | SV-7 | Opc- |
| 38853 | 2481 | Neisseria meningitidis | NG | ST-334 complex | Endemic | Italy | SV-15 | Opc- |
| 38854 | 2482 | Neisseria meningitidis | NG | ST-334 complex | Endemic | Italy | SV-15 | Opc- |
| 38855 | 2484 | Neisseria meningitidis | NG | ST-334 complex | Endemic | Italy | SV-15 | Opc- |
| 38856 | 2497 | Neisseria meningitidis | NG | ST-23 complex | Endemic | Italy | SV-11 | Opc+ |
| 38857 | 2499 | Neisseria meningitidis | NG | ST-334 complex | Endemic | Italy | SV-15 | Opc- |
| 38858 | 2501 | Neisseria meningitidis | NG | ST-11 complex | Endemic | Italy | SV-2 | Opc- |
| 38859 | 2504 | Neisseria meningitidis | NG | ST-11 complex | Endemic | Italy | SV-2 | Opc- |
| 38860 | 2511 | Neisseria meningitidis | NG | ST-334 complex | Endemic | Italy | SV-15 | Opc- |
| 38861 | 2512 | Neisseria meningitidis | NG | ST-334 complex | Endemic | Italy | SV-15 | Opc- |
| 38862 | 2513 | Neisseria meningitidis | NG | ST-334 complex | Endemic | Italy | SV-15 | Opc- |
| 38863 | 2527 | Neisseria meningitidis | NG | ST-11 complex | Endemic | Italy | SV-2 | Opc- |
| 38864 | 2529 | Neisseria meningitidis | NG | ST-334 complex | Endemic | Italy | SV-15 | Opc- |
| 38865 | 2533 | Neisseria meningitidis | NG | ST-11 complex | Endemic | Italy | SV-2 | Opc- |
| 38866 | 2535 | Neisseria meningitidis | NG | ST-11 complex | Endemic | Italy | SV-2 | Opc- |
| 38867 | 2541 | Neisseria meningitidis | NG | ST-11 complex | Endemic | Italy | SV-2 | Opc- |
| 38868 | 2548 | Neisseria meningitidis | NG | No value | Endemic | Italy | SV-11 | Opc- |
| 38869 | 2549 | Neisseria meningitidis | NG | ST-334 complex | Endemic | Italy | SV-15 | Opc- |
| 38870 | 2553 | Neisseria meningitidis | NG | ST-334 complex | Endemic | Italy | SV-15 | Opc- |
| 38871 | 2557 | Neisseria meningitidis | NG | ST-11 complex | Endemic | Italy | SV-2 | Opc- |
| 38872 | 2568 | Neisseria meningitidis | NG | ST-334 complex | Endemic | Italy | SV-15 | Opc- |
| 38873 | 2575 | Neisseria meningitidis | NG | ST-334 complex | Endemic | Italy | SV-15 | Opc- |
| 38874 | 2633 | Neisseria meningitidis | NG | ST-41/44 complex | Endemic | Italy | SV-7 | Opc+ |
| 38875 | P6 | Neisseria meningitidis | B | ST-1136 complex | Carrier | Italy | SV-2 | Opc+ |
| 38876 | P10 | Neisseria meningitidis | B | ST-32 complex | Carrier | Italy | SV-1 | Opc+ |
| 38877 | P11 | Neisseria meningitidis | NG | ST-1136 complex | Carrier | Italy | SV-2 | Opc+ |
| 38878 | P12 | Neisseria meningitidis | NG | ST-198 complex | Carrier | Italy | SV-4 | Opc+ |
| 38879 | P14 | Neisseria meningitidis | NG | No value | Carrier | Italy | SV-5 | Opc+ |
| 38880 | P15 | Neisseria meningitidis | NG | ST-1136 complex | Carrier | Italy | SV-2 | Opc+ |
| 38881 | P16 | Neisseria meningitidis | NG | ST-198 complex | Carrier | Italy | SV-4 | Opc+ |
| 38882 | P17 | Neisseria meningitidis | NG | ST-865 complex | Carrier | Italy | SV-2 | Opc+ |
| 38883 | P18 | Neisseria meningitidis | NG | No value | Carrier | Italy | SV-2 | Opc+ |
| 38884 | P19 | Neisseria meningitidis | NG | No value | Carrier | Italy | SV-2 | Opc+ |
| 38885 | P20 | Neisseria meningitidis | NG | No value | Carrier | Italy | SV-2 | Opc+ |
| 38886 | P21 | Neisseria meningitidis | NG | No value | Carrier | Italy | SV-2 | Opc+ |
| 38887 | P22 | Neisseria meningitidis | NG | ST-167 complex | Carrier | Italy | SV-5 | Opc+ |
| 38888 | P24 | Neisseria meningitidis | NG | ST-198 complex | Carrier | Italy | SV-4 | Opc+ |
| 38889 | P27 | Neisseria meningitidis | NG | ST-53 complex | Carrier | Italy | SV-2 | Opc- |
| 38890 | P28 | Neisseria meningitidis | C | ST-41/44 complex | Carrier | Italy | SV-7 | Opc+ |
| 38891 | P29 | Neisseria meningitidis | NG | ST-198 complex | Carrier | Italy | SV-4 | Opc+ |
| 38892 | P30 | Neisseria meningitidis | NG | ST-1136 complex | Carrier | Italy | SV-2 | Opc+ |
| 38893 | P32 | Neisseria meningitidis | C | ST-41/44 complex | Carrier | Italy | SV-10 | Opc+ |
| 38894 | P35 | Neisseria meningitidis | C | ST-41/44 complex | Carrier | Italy | SV-7 | Opc+ |

| | | | | | | | | |
|---|---|---|---|---|---|---|---|---|
| 38902 | P36 | Neisseria meningitidis | C | ST-41/44 complex | Carrier | Italy | SV-10 | Opc+ |
| 38903 | P38 | Neisseria meningitidis | NG | ST-198 complex | Carrier | Italy | SV-7 | Opc+ |
| 38904 | P43 | Neisseria meningitidis | C | ST-41/44 complex | Carrier | Italy | SV-10 | Opc+ |
| 38905 | P44 | Neisseria meningitidis | NG | ST-1136 complex | Carrier | Italy | SV-2 | Opc+ |
| 38906 | P51 | Neisseria meningitidis | E | ST-60 complex | Carrier | Italy | SV-11 | Opc+ |
| 38907 | P54 | Neisseria meningitidis | NG | ST-198 complex | Carrier | Italy | SV-4 | Opc+ |
| 38908 | P55 | Neisseria meningitidis | Y | ST-23 complex | Carrier | Italy | SV-11 | Opc+ |
| 38909 | P58 | Neisseria meningitidis | NG | ST-198 complex | Carrier | Italy | SV-7 | Opc+ |
| 38910 | P62 | Neisseria meningitidis | C | ST-41/44 complex | Carrier | Italy | SV-10 | Opc+ |
| 38911 | P66 | Neisseria meningitidis | NG | ST-1136 complex | Carrier | Italy | SV-2 | Opc+ |
| 38912 | P67 | Neisseria meningitidis | NG | ST-1136 complex | Carrier | Italy | SV-2 | Opc+ |
| 38913 | P69 | Neisseria meningitidis | NG | ST-1136 complex | Carrier | Italy | SV-2 | Opc+ |
| 38914 | P74 | Neisseria meningitidis | B | ST-461 complex | Carrier | Italy | SV-1 | Opc- |
| 38915 | P75 | Neisseria meningitidis | NG | ST-198 complex | Carrier | Italy | SV-4 | Opc+ |
| 38916 | P76 | Neisseria meningitidis | NG | ST-1136 complex | Carrier | Italy | SV-2 | Opc+ |
| 38917 | P77 | Neisseria meningitidis | NG | ST-1136 complex | Carrier | Italy | SV-2 | Opc+ |
| 38918 | P78 | Neisseria meningitidis | NG | ST-1136 complex | Carrier | Italy | SV-2 | Opc+ |
| 38919 | P79 | Neisseria meningitidis | B | ST-41/44 complex | Carrier | Italy | SV-6 | Opc+ |
| 38920 | P80 | Neisseria meningitidis | B | ST-41/44 complex | Carrier | Italy | SV-12 | Opc+ |
| 38921 | P81 | Neisseria meningitidis | NG | ST-198 complex | Carrier | Italy | SV-4 | Opc+ |
| 38922 | P82 | Neisseria meningitidis | B | ST-198 complex | Carrier | Italy | SV-4 | Opc+ |
| 38923 | P83 | Neisseria meningitidis | B | ST-41/44 complex | Carrier | Italy | SV-6 | Opc+ |
| 38924 | P85 | Neisseria meningitidis | NG | ST-32 complex | Carrier | Italy | SV-1 | Opc+ |
| 38925 | P87 | Neisseria meningitidis | B | ST-41/44 complex | Carrier | Italy | SV-5 | Opc+ |
| 38926 | P88 | Neisseria meningitidis | NG | ST-198 complex | Carrier | Italy | SV-7 | Opc+ |
| 38927 | P89 | Neisseria meningitidis | NG | ST-41/44 complex | Carrier | Italy | SV-6 | Opc+ |
| 38928 | P90 | Neisseria meningitidis | NG | ST-1136 complex | Carrier | Italy | SV-2 | Opc+ |
| 38929 | P92 | Neisseria meningitidis | NG | ST-1136 complex | Carrier | Italy | SV-2 | Opc+ |
| 38931 | P94 | Neisseria meningitidis | NG | ST-1136 complex | Carrier | Italy | SV-2 | Opc+ |
| 38932 | P95 | Neisseria meningitidis | B | No value | Carrier | Italy | SV-1 | Opc+ |
| 38933 | P96 | Neisseria meningitidis | B | ST-461 complex | Carrier | Italy | SV-1 | Opc- |
| 38934 | P97 | Neisseria meningitidis | A | ST-23 complex | Carrier | Italy | SV-11 | Opc+ |
| 38935 | P98 | Neisseria meningitidis | A | ST-23 complex | Carrier | Italy | SV-11 | Opc+ |
| 38936 | P99 | Neisseria meningitidis | NG | ST-1157 complex | Carrier | Italy | SV-1 | Opc+ |
| 38937 | P100 | Neisseria meningitidis | NG | ST-1157 complex | Carrier | Italy | SV-1 | Opc+ |
| 38938 | P101 | Neisseria meningitidis | C | ST-22 complex | Carrier | Italy | SV-12 | Opc+ |
| 38939 | P102 | Neisseria meningitidis | NG | ST-198 complex | Carrier | Italy | SV-4 | Opc+ |
| 38940 | P103 | Neisseria meningitidis | NG | ST-1136 complex | Carrier | Italy | SV-2 | Opc+ |
| 38941 | P104 | Neisseria meningitidis | C | ST-22 complex | Carrier | Italy | SV-12 | Opc+ |
| 38942 | P105 | Neisseria meningitidis | C | ST-22 complex | Carrier | Italy | SV-12 | Opc+ |
| 38943 | P106 | Neisseria meningitidis | NG | ST-53 complex | Carrier | Italy | SV-2 | Opc- |
| 38944 | P107 | Neisseria meningitidis | NG | ST-198 complex | Carrier | Italy | SV-4 | Opc+ |
| 38945 | P108 | Neisseria meningitidis | B | No value | Carrier | Italy | SV-2 | Opc+ |
| 38946 | P109 | Neisseria meningitidis | NG | ST-53 complex | Carrier | Italy | SV-2 | Opc- |
| 38947 | P110 | Neisseria meningitidis | NG | ST-1117 complex | Carrier | Italy | SV-10 | Opc- |
| 38948 | P111 | Neisseria meningitidis | NG | ST-198 complex | Carrier | Italy | SV-4 | Opc+ |
| 38949 | P113 | Neisseria meningitidis | NG | ST-198 complex | Carrier | Italy | SV-4 | Opc+ |
| 38950 | P114 | Neisseria meningitidis | NG | ST-198 complex | Carrier | Italy | SV-7 | Opc+ |
| 38951 | P115 | Neisseria meningitidis | NG | ST-198 complex | Carrier | Italy | SV-4 | Opc+ |
| 38952 | P116 | Neisseria meningitidis | Y | ST-23 complex | Carrier | Italy | SV-11 | Opc+ |
| 38953 | P117 | Neisseria meningitidis | NG | ST-53 complex | Carrier | Italy | SV-2 | Opc- |
| 38954 | P118 | Neisseria meningitidis | NG | ST-53 complex | Carrier | Italy | SV-2 | Opc- |
| 38955 | P119 | Neisseria meningitidis | NG | ST-198 complex | Carrier | Italy | SV-4 | Opc+ |
| 38956 | P120 | Neisseria meningitidis | NG | ST-53 complex | Carrier | Italy | SV-2 | Opc- |
| 38957 | P121 | Neisseria meningitidis | B | ST-35 complex | Carrier | Italy | SV-12 | Opc+ |
| 38995 | 15.8700107 | Neisseria meningitidis | W | No value | No value | UK | SV-2 | Opc- |
| 38996 | 15.8700914 | Neisseria meningitidis | W | ST-11 complex | No value | UK | SV-2 | Opc- |
| 38997 | 15.8701412 | Neisseria meningitidis | W | ST-11 complex | No value | UK | SV-2 | Opc- |
| 38998 | 15.8703241 | Neisseria meningitidis | W | ST-11 complex | No value | UK | SV-2 | Opc- |
| 38999 | 15.8703378 | Neisseria meningitidis | W | ST-11 complex | No value | UK | SV-2 | Opc- |
| 39000 | 15.8703957 | Neisseria meningitidis | W | ST-11 complex | No value | UK | SV-2 | Opc- |
| 39001 | 15.8705087 | Neisseria meningitidis | W | ST-11 complex | No value | UK | SV-2 | Opc- |
| 39002 | M15 240552 | Neisseria meningitidis | B | ST-41/44 complex | No value | UK | SV-2 | Opc+ |
| 39147 | IH167468 | Neisseria meningitidis | B | No value | No value | Finland | SV-1 | Opc+ |
| 39148 | IH167475 | Neisseria meningitidis | B | ST-41/44 complex | No value | Finland | SV-2 | Opc+ |
| 39149 | 107457 | Neisseria meningitidis | B | ST-41/44 complex | No value | Finland | SV-10 | Opc+ |
| 39150 | 107458 | Neisseria meningitidis | B | ST-41/44 complex | No value | Finland | SV-10 | Opc+ |
| 39152 | PE5 | Neisseria meningitidis | C | ST-11 complex | Carrier | Italy | SV-2 | Opc- |
| 39153 | PE6 | Neisseria meningitidis | C | ST-11 complex | Carrier | Italy | SV-2 | Opc- |
| 39154 | PE7 | Neisseria meningitidis | C | ST-11 complex | Carrier | Italy | SV-2 | Opc- |
| 39318 | M15 240625 | Neisseria meningitidis | B | No value | No value | UK | SV-5 | Opc+ |
| 39319 | M15 240626 | Neisseria meningitidis | C | ST-11 complex | No value | UK | SV-2 | Opc- |
| 39320 | M15 240629 | Neisseria meningitidis | W | ST-11 complex | No value | UK | SV-2 | Opc- |
| 39321 | M15 240631 | Neisseria meningitidis | Y | ST-23 complex | No value | UK | SV-11 | Opc+ |

| | | | | | | | | |
|---|---|---|---|---|---|---|---|---|
| 39322 | M15 240634 | Neisseria meningitidis | B | ST-269 complex | No value | UK | SV-1 | Opc+ |
| 39323 | M15 240635 | Neisseria meningitidis | Y | No value | No value | UK | SV-11 | Opc+ |
| 39324 | M15 240636 | Neisseria meningitidis | B | ST-41/44 complex | No value | UK | SV-10 | Opc+ |
| 39325 | M15 240637 | Neisseria meningitidis | B | No value | No value | UK | SV-2 | Opc+ |
| 39326 | M15 240638 | Neisseria meningitidis | W | ST-11 complex | No value | UK | SV-2 | Opc- |
| 39327 | M15 240639 | Neisseria meningitidis | W | ST-22 complex | No value | UK | SV-12 | Opc+ |
| 39328 | M15 240640 | Neisseria meningitidis | W | ST-11 complex | No value | UK | SV-2 | Opc- |
| 39329 | M15 240641 | Neisseria meningitidis | B | No value | No value | UK | SV-8 | Opc+ |
| 39330 | M15 240642 | Neisseria meningitidis | Y | ST-23 complex | No value | UK | SV-11 | Opc+ |
| 39331 | M15 240643 | Neisseria meningitidis | W | ST-11 complex | No value | UK | SV-2 | Opc- |
| 39332 | M15 240644 | Neisseria meningitidis | Y | ST-23 complex | No value | UK | SV-11 | Opc+ |
| 39333 | M15 240645 | Neisseria meningitidis | W | No value | No value | UK | SV-12 | Opc+ |
| 39334 | M15 240646 | Neisseria meningitidis | Y | No value | No value | UK | SV-11 | Opc+ |
| 39335 | M15 240648 | Neisseria meningitidis | W | ST-11 complex | No value | UK | SV-2 | Opc- |
| 39336 | M15 240650 | Neisseria meningitidis | B | ST-269 complex | No value | UK | SV-1 | Opc+ |
| 39337 | M15 240651 | Neisseria meningitidis | W | ST-11 complex | No value | UK | SV-2 | Opc- |
| 39338 | M15 240652 | Neisseria meningitidis | B | No value | No value | UK | SV-5 | Opc+ |
| 39339 | M15 240653 | Neisseria meningitidis | Y | ST-23 complex | No value | UK | SV-11 | Opc+ |
| 39340 | M15 240657 | Neisseria meningitidis | W | ST-11 complex | No value | UK | SV-2 | Opc- |
| 39341 | M15 240658 | Neisseria meningitidis | W | ST-11 complex | No value | UK | SV-2 | Opc- |
| 39342 | M15 240659 | Neisseria meningitidis | W | ST-11 complex | No value | UK | SV-2 | Opc- |
| 39343 | M15 240660 | Neisseria meningitidis | Y | No value | No value | UK | SV-11 | Opc+ |
| 39344 | M15 240661 | Neisseria meningitidis | W | ST-11 complex | No value | UK | SV-2 | Opc- |
| 39345 | M15 240662 | Neisseria meningitidis | W | ST-11 complex | No value | UK | SV-2 | Opc- |
| 39346 | M15 240664 | Neisseria meningitidis | W | ST-11 complex | No value | UK | SV-2 | Opc- |
| 39347 | M15 240697 | Neisseria meningitidis | Y | ST-23 complex | No value | UK | SV-11 | Opc+ |
| 39348 | M15 240698 | Neisseria meningitidis | B | ST-269 complex | No value | UK | SV-8 | Opc+ |
| 39349 | M15 240699 | Neisseria meningitidis | W | No value | No value | UK | SV-2 | Opc- |
| 39350 | M15 240701 | Neisseria meningitidis | B | ST-269 complex | No value | UK | SV-1 | Opc+ |
| 39351 | M15 240703 | Neisseria meningitidis | B | ST-269 complex | No value | UK | SV-8 | Opc+ |
| 39352 | M15 240704 | Neisseria meningitidis | W | ST-11 complex | No value | UK | SV-2 | Opc- |
| 39353 | M15 240705 | Neisseria meningitidis | C | ST-11 complex | No value | UK | SV-2 | Opc- |
| 39354 | M15 240716 | Neisseria meningitidis | NG | ST-1157 complex | No value | UK | SV-1 | Opc+ |
| 39355 | M15 240717 | Neisseria meningitidis | B | No value | No value | UK | SV-1 | Opc+ |
| 39356 | M15 240718 | Neisseria meningitidis | W | ST-11 complex | No value | UK | SV-2 | Opc- |
| 39357 | M15 240719 | Neisseria meningitidis | B | No value | No value | UK | SV-1 | Opc+ |
| 39358 | M15 240720 | Neisseria meningitidis | W | ST-11 complex | No value | UK | SV-2 | Opc- |
| 39359 | M15 240723 | Neisseria meningitidis | Y | ST-23 complex | No value | UK | SV-11 | Opc+ |
| 39360 | M15 240724 | Neisseria meningitidis | B | ST-41/44 complex | No value | UK | SV-10 | Opc+ |
| 39361 | M15 240725 | Neisseria meningitidis | C | ST-11 complex | No value | UK | SV-2 | Opc- |
| 39362 | M15 240727 | Neisseria meningitidis | B | ST-41/44 complex | No value | UK | SV-2 | Opc+ |
| 39363 | M15 240728 | Neisseria meningitidis | Y | ST-23 complex | No value | UK | SV-11 | Opc+ |
| 39364 | M15 240729 | Neisseria meningitidis | B | ST-41/44 complex | No value | UK | SV-2 | Opc+ |
| 39365 | M15 240730 | Neisseria meningitidis | W | ST-11 complex | No value | UK | SV-2 | Opc- |
| 39366 | M15 240737 | Neisseria meningitidis | B | ST-41/44 complex | No value | UK | SV-2 | Opc+ |
| 39367 | M15 240738 | Neisseria meningitidis | B | ST-461 complex | No value | UK | SV-1 | Opc- |
| 39368 | M15 240740 | Neisseria meningitidis | W | ST-11 complex | No value | UK | SV-2 | Opc- |
| 39369 | M15 240742 | Neisseria meningitidis | W | ST-11 complex | No value | UK | SV-2 | Opc- |
| 39370 | M15 240743 | Neisseria meningitidis | B | ST-41/44 complex | No value | UK | SV-2 | Opc+ |
| 39371 | M15 240744 | Neisseria meningitidis | B | ST-269 complex | No value | UK | SV-1 | Opc+ |
| 39372 | M15 240745 | Neisseria meningitidis | B | ST-41/44 complex | No value | UK | SV-2 | Opc+ |
| 39373 | M15 240746 | Neisseria meningitidis | W | ST-11 complex | No value | UK | SV-2 | Opc- |
| 39374 | M15 240747 | Neisseria meningitidis | B | No value | No value | UK | SV-1 | Opc+ |
| 39375 | M15 240748 | Neisseria meningitidis | B | ST-41/44 complex | No value | UK | SV-2 | Opc+ |
| 39376 | M15 240749 | Neisseria meningitidis | Y | ST-23 complex | No value | UK | SV-11 | Opc+ |
| 39377 | M15 240750 | Neisseria meningitidis | Y | ST-174 complex | No value | UK | SV-1 | Opc+ |
| 39378 | M15 240751 | Neisseria meningitidis | C | ST-11 complex | No value | UK | SV-2 | Opc- |
| 39379 | M15 240752 | Neisseria meningitidis | Y | ST-23 complex | No value | UK | SV-11 | Opc+ |
| 39380 | M15 240755 | Neisseria meningitidis | NG | ST-162 complex | No value | UK | SV-1 | Opc+ |
| 39381 | M15 240757 | Neisseria meningitidis | B | ST-22 complex | No value | UK | SV-12 | Opc+ |
| 39382 | M15 240758 | Neisseria meningitidis | B | No value | No value | UK | SV-11 | Opc- |
| 39383 | M15 240759 | Neisseria meningitidis | NG | ST-41/44 complex | No value | UK | SV-6 | Opc+ |
| 39384 | M15 240760 | Neisseria meningitidis | B | ST-269 complex | No value | UK | SV-1 | Opc+ |
| 39385 | M15 240762 | Neisseria meningitidis | W | ST-11 complex | No value | UK | SV-2 | Opc- |
| 39386 | M15 240763 | Neisseria meningitidis | B | No value | No value | UK | SV-2 | Opc+ |
| 39387 | M15 240765 | Neisseria meningitidis | W | ST-22 complex | No value | UK | SV-12 | Opc+ |
| 39388 | M15 240766 | Neisseria meningitidis | C | ST-11 complex | No value | UK | SV-2 | Opc- |
| 39389 | M15 240767 | Neisseria meningitidis | B | ST-32 complex | No value | UK | SV-1 | Opc+ |
| 39391 | M15 240770 | Neisseria meningitidis | B | ST-32 complex | No value | UK | SV-1 | Opc+ |
| 39392 | M15 240772 | Neisseria meningitidis | B | ST-213 complex | No value | UK | SV-1 | Opc- |
| 39393 | M15 240773 | Neisseria meningitidis | B | No value | No value | UK | SV-1 | Opc+ |
| 39394 | M15 240775 | Neisseria meningitidis | B | ST-41/44 complex | No value | UK | SV-2 | Opc+ |
| 39395 | M15 240778 | Neisseria meningitidis | W | ST-11 complex | No value | UK | SV-2 | Opc- |
| 39396 | M15 240779 | Neisseria meningitidis | W | ST-11 complex | No value | UK | SV-2 | Opc- |

| | | | | | | | | |
|---|---|---|---|---|---|---|---|---|
| 39397 | M15 240781 | Neisseria meningitidis | B | No value | No value | UK | SV-11 | Opc+ |
| 39398 | M15 240782 | Neisseria meningitidis | W | ST-11 complex | No value | UK | SV-2 | Opc- |
| 39399 | M15 240783 | Neisseria meningitidis | B | ST-269 complex | No value | UK | SV-8 | Opc+ |
| 39400 | M15 240784 | Neisseria meningitidis | B | ST-41/44 complex | No value | UK | SV-2 | Opc+ |
| 39401 | M15 240785 | Neisseria meningitidis | W | ST-11 complex | No value | UK | SV-2 | Opc- |
| 39402 | M15 240786 | Neisseria meningitidis | B | ST-41/44 complex | No value | UK | SV-2 | Opc+ |
| 39403 | M15 240787 | Neisseria meningitidis | C | ST-11 complex | No value | UK | SV-2 | Opc- |
| 39404 | M15 240788 | Neisseria meningitidis | W | ST-11 complex | No value | UK | SV-2 | Opc- |
| 39405 | M15 240789 | Neisseria meningitidis | B | ST-1157 complex | No value | UK | SV-1 | Opc+ |
| 39406 | M15 240790 | Neisseria meningitidis | B | ST-269 complex | No value | UK | SV-8 | Opc+ |
| 39407 | M15 240793 | Neisseria meningitidis | B | ST-41/44 complex | No value | UK | SV-10 | Opc+ |
| 39408 | M15 240794 | Neisseria meningitidis | W | ST-11 complex | No value | UK | SV-2 | Opc- |
| 39409 | M15 240795 | Neisseria meningitidis | C | ST-11 complex | No value | UK | SV-2 | Opc- |
| 39410 | M15 240796 | Neisseria meningitidis | W | ST-11 complex | No value | UK | SV-2 | Opc- |
| 39411 | M15 240797 | Neisseria meningitidis | W | ST-11 complex | No value | UK | SV-2 | Opc- |
| 39412 | M15 240798 | Neisseria meningitidis | W | ST-11 complex | No value | UK | SV-2 | Opc- |
| 39413 | M15 240799 | Neisseria meningitidis | B | No value | No value | UK | SV-5 | Opc+ |
| 39414 | M15 240801 | Neisseria meningitidis | B | ST-103 complex | No value | UK | SV-1 | Opc+ |
| 39415 | M15 240802 | Neisseria meningitidis | B | ST-32 complex | No value | UK | SV-1 | Opc+ |
| 39416 | M15 240803 | Neisseria meningitidis | W | ST-22 complex | No value | UK | SV-12 | Opc+ |
| 39417 | M15 240805 | Neisseria meningitidis | B | ST-213 complex | No value | UK | SV-12 | Opc- |
| 39418 | M15 240806 | Neisseria meningitidis | B | No value | No value | UK | SV-5 | Opc- |
| 39419 | M15 240807 | Neisseria meningitidis | Y | ST-23 complex | No value | UK | SV-11 | Opc+ |
| 39420 | M15 240808 | Neisseria meningitidis | Y | ST-23 complex | No value | UK | SV-11 | Opc+ |
| 39421 | M15 240816 | Neisseria meningitidis | W | ST-11 complex | No value | UK | SV-2 | Opc- |
| 39422 | M15 240818 | Neisseria meningitidis | W | ST-11 complex | No value | UK | SV-2 | Opc- |
| 39423 | M15 240819 | Neisseria meningitidis | W | ST-11 complex | No value | UK | SV-2 | Opc- |
| 39424 | M15 240820 | Neisseria meningitidis | W | No value | No value | UK | SV-7 | Opc+ |
| 39425 | M15 240821 | Neisseria meningitidis | B | ST-461 complex | No value | UK | SV-1 | Opc- |
| 39426 | M15 240822 | Neisseria meningitidis | Y | ST-23 complex | No value | UK | SV-11 | Opc+ |
| 39427 | M15 240826 | Neisseria meningitidis | W | ST-11 complex | No value | UK | SV-2 | Opc- |
| 39429 | M15 240829 | Neisseria meningitidis | W | ST-11 complex | No value | UK | SV-2 | Opc- |
| 39430 | M15 240830 | Neisseria meningitidis | W | ST-11 complex | No value | UK | SV-2 | Opc- |
| 39431 | M15 240831 | Neisseria meningitidis | W | ST-11 complex | No value | UK | SV-2 | Opc- |
| 39432 | M15 240832 | Neisseria meningitidis | Y | ST-23 complex | No value | UK | SV-11 | Opc+ |
| 39433 | M15 240833 | Neisseria meningitidis | W | ST-11 complex | No value | UK | SV-2 | Opc- |
| 39434 | M15 240835 | Neisseria meningitidis | B | ST-269 complex | No value | UK | SV-1 | Opc+ |
| 39435 | M15 240844 | Neisseria meningitidis | W | ST-11 complex | No value | UK | SV-2 | Opc- |
| 39436 | M15 240845 | Neisseria meningitidis | W | ST-11 complex | No value | UK | SV-2 | Opc- |
| 39437 | M15 240847 | Neisseria meningitidis | Y | ST-23 complex | No value | UK | SV-11 | Opc+ |
| 39438 | M15 240848 | Neisseria meningitidis | W | ST-11 complex | No value | UK | SV-2 | Opc- |
| 39439 | M15 240849 | Neisseria meningitidis | B | ST-41/44 complex | No value | UK | SV-2 | Opc+ |
| 39440 | M15 240850 | Neisseria meningitidis | Y | ST-23 complex | No value | UK | SV-11 | Opc+ |
| 39441 | M15 240722b | Neisseria meningitidis | W | ST-11 complex | No value | UK | SV-2 | Opc- |
| 39442 | M07 240646 | Neisseria meningitidis | B | ST-269 complex | No value | UK | SV-8 | Opc+ |
| 39443 | M07 240657 | Neisseria meningitidis | B | ST-213 complex | No value | UK | SV-12 | Opc- |
| 39444 | M07 240669 | Neisseria meningitidis | B | ST-41/44 complex | No value | UK | SV-6 | Opc+ |
| 39445 | M07 240680 | Neisseria meningitidis | B | ST-41/44 complex | No value | UK | SV-10 | Opc+ |
| 39446 | M07 240789 | Neisseria meningitidis | B | ST-282 complex | No value | UK | SV-9 | Opc+ |
| 39447 | M07 240850 | Neisseria meningitidis | B | No value | No value | UK | SV-10 | Opc- |
| 39448 | M07 240889 | Neisseria meningitidis | B | ST-269 complex | No value | UK | SV-8 | Opc+ |
| 39449 | M07 240909 | Neisseria meningitidis | B | No value | No value | UK | SV-12 | Opc- |
| 39450 | M07 240949 | Neisseria meningitidis | B | ST-41/44 complex | No value | UK | SV-3 | Opc+ |
| 39451 | M07 241052 | Neisseria meningitidis | B | No value | No value | UK | SV-1 | Opc+ |
| 39452 | M07 241084 | Neisseria meningitidis | B | ST-41/44 complex | No value | UK | SV-10 | Opc+ |
| 39453 | M07 241109 | Neisseria meningitidis | B | ST-364 complex | No value | UK | SV-1 | Opc- |
| 39454 | M07 241117 | Neisseria meningitidis | B | ST-269 complex | No value | UK | SV-8 | Opc+ |
| 39455 | M08 240035 | Neisseria meningitidis | B | ST-269 complex | No value | UK | SV-8 | Opc+ |
| 39456 | M08 240169 | Neisseria meningitidis | NG | ST-750 complex | No value | UK | SV-5 | Opc- |
| 39457 | M08 240220 | Neisseria meningitidis | B | ST-41/44 complex | No value | UK | SV-10 | Opc+ |
| 39458 | M08 240222 | Neisseria meningitidis | B | ST-41/44 complex | No value | UK | SV-10 | Opc+ |
| 39459 | M08 240255 | Neisseria meningitidis | B | ST-41/44 complex | No value | UK | SV-6 | Opc+ |
| 39460 | M08 240276 | Neisseria meningitidis | B | ST-213 complex | No value | UK | SV-12 | Opc- |
| 39461 | M08 240293 | Neisseria meningitidis | B | ST-269 complex | No value | UK | SV-8 | Opc+ |
| 39462 | M08 240302 | Neisseria meningitidis | B | ST-269 complex | No value | UK | SV-8 | Opc+ |
| 39463 | M08 240498 | Neisseria meningitidis | B | ST-282 complex | No value | UK | SV-9 | Opc+ |
| 39569 | 2728 | Neisseria meningitidis | NG | ST-11 complex | Endemic | Italy | SV-2 | Opc- |
| 39570 | 2736 | Neisseria meningitidis | NG | ST-11 complex | Endemic | Italy | SV-2 | Opc- |
| 39571 | PE12 | Neisseria meningitidis | NG | ST-11 complex | Carrier | Italy | SV-2 | Opc- |
| 39584 | LNP28126 | Neisseria meningitidis | C | ST-11 complex | No value | France | SV-2 | Opc- |
| 39585 | LNP28208 | Neisseria meningitidis | C | ST-11 complex | No value | France | SV-2 | Opc- |
| 39625 | M15141 | Neisseria meningitidis | C | ST-11 complex | No value | USA | SV-2 | Opc- |
| 39696 | LNP27270 | Neisseria meningitidis | B | ST-41/44 complex | No value | France | SV-2 | Opc+ |
| 39697 | LNP27272 | Neisseria meningitidis | B | ST-461 complex | No value | France | SV-1 | Opc- |

| | | | | | | | | |
|---|---|---|---|---|---|---|---|---|
| 39698 | LNP27275 | Neisseria meningitidis | B | ST-461 complex | No value | France | SV-1 | Opc- |
| 39699 | LNP27274 | Neisseria meningitidis | B | ST-60 complex | No value | France | SV-11 | Opc+ |
| 39700 | LNP27286 | Neisseria meningitidis | B | ST-32 complex | No value | France | SV-1 | Opc+ |
| 39701 | LNP27291 | Neisseria meningitidis | B | ST-162 complex | No value | France | SV-1 | Opc+ |
| 39702 | LNP27290 | Neisseria meningitidis | B | ST-1157 complex | No value | France | SV-1 | Opc+ |
| 39703 | LNP27293 | Neisseria meningitidis | B | ST-32 complex | No value | France | SV-1 | Opc+ |
| 39704 | LNP27296 | Neisseria meningitidis | B | ST-32 complex | No value | France | SV-1 | Opc+ |
| 39705 | LNP27297 | Neisseria meningitidis | B | ST-35 complex | No value | France | SV-12 | Opc+ |
| 39706 | LNP27302 | Neisseria meningitidis | B | ST-41/44 complex | No value | France | SV-2 | Opc+ |
| 39707 | LNP27305 | Neisseria meningitidis | B | ST-41/44 complex | No value | France | SV-10 | Opc+ |
| 39708 | LNP27341 | Neisseria meningitidis | B | ST-32 complex | No value | France | SV-1 | Opc+ |
| 39709 | LNP27306 | Neisseria meningitidis | B | ST-461 complex | No value | France | SV-1 | Opc- |
| 39710 | LNP27311 | Neisseria meningitidis | B | ST-41/44 complex | No value | France | SV-6 | Opc+ |
| 39711 | LNP27313 | Neisseria meningitidis | B | ST-32 complex | No value | France | SV-1 | Opc+ |
| 39712 | LNP27322 | Neisseria meningitidis | B | No value | No value | France | SV-6 | Opc+ |
| 39940 | LNP28306 | Neisseria meningitidis | C | ST-11 complex | No value | France | SV-2 | Opc- |
| 39941 | LNP28314 | Neisseria meningitidis | C | ST-11 complex | No value | France | SV-2 | Opc- |
| 39943 | LNP27321 | Neisseria meningitidis | B | ST-35 complex | No value | France | SV-12 | Opc+ |
| 39944 | LNP27325 | Neisseria meningitidis | B | ST-41/44 complex | No value | France | SV-10 | Opc+ |
| 39945 | LNP27327 | Neisseria meningitidis | B | ST-41/44 complex | No value | France | SV-6 | Opc+ |
| 39946 | LNP27329 | Neisseria meningitidis | B | ST-269 complex | No value | France | SV-2 | Opc+ |
| 39947 | LNP27334 | Neisseria meningitidis | B | ST-32 complex | No value | France | SV-1 | Opc+ |
| 39948 | LNP27332 | Neisseria meningitidis | B | ST-41/44 complex | No value | France | SV-6 | Opc+ |
| 39949 | LNP27336 | Neisseria meningitidis | B | ST-35 complex | No value | France | SV-12 | Opc+ |
| 39950 | LNP27338 | Neisseria meningitidis | B | ST-32 complex | No value | France | SV-1 | Opc+ |
| 39951 | LNP27337 | Neisseria meningitidis | B | ST-32 complex | No value | France | SV-1 | Opc+ |
| 39952 | LNP27340 | Neisseria meningitidis | B | ST-269 complex | No value | France | SV-8 | Opc+ |
| 39953 | LNP27342 | Neisseria meningitidis | B | No value | No value | France | SV-2 | Opc+ |
| 39954 | LNP27344 | Neisseria meningitidis | B | ST-269 complex | No value | France | SV-8 | Opc+ |
| 39955 | LNP27347 | Neisseria meningitidis | B | ST-41/44 complex | No value | France | SV-6 | Opc+ |
| 39956 | LNP27349 | Neisseria meningitidis | B | ST-269 complex | No value | France | SV-1 | Opc+ |
| 39957 | LNP27348 | Neisseria meningitidis | B | ST-41/44 complex | No value | France | SV-10 | Opc+ |
| 39958 | LNP27350 | Neisseria meningitidis | B | ST-32 complex | No value | France | SV-1 | Opc+ |
| 39959 | LNP27353 | Neisseria meningitidis | B | ST-32 complex | No value | France | SV-1 | Opc+ |
| 39960 | LNP27356 | Neisseria meningitidis | B | ST-60 complex | No value | France | SV-5 | Opc+ |
| 39961 | LNP27354 | Neisseria meningitidis | B | ST-60 complex | No value | France | SV-5 | Opc+ |
| 39962 | LNP27355 | Neisseria meningitidis | B | No value | No value | France | SV-1 | Opc+ |
| 39963 | LNP27359 | Neisseria meningitidis | B | ST-41/44 complex | No value | France | SV-10 | Opc+ |
| 39964 | LNP27361 | Neisseria meningitidis | B | No value | No value | France | SV-1 | Opc+ |
| 39965 | LNP27363 | Neisseria meningitidis | B | ST-213 complex | No value | France | SV-12 | Opc- |
| 39966 | LNP27364 | Neisseria meningitidis | B | ST-41/44 complex | No value | France | SV-10 | Opc+ |
| 39967 | LNP27367 | Neisseria meningitidis | B | No value | No value | France | SV-11 | Opc+ |
| 39968 | LNP27372 | Neisseria meningitidis | B | ST-32 complex | No value | France | SV-1 | Opc+ |
| 39969 | LNP27369 | Neisseria meningitidis | B | ST-162 complex | No value | France | SV-1 | Opc+ |
| 39970 | LNP27374 | Neisseria meningitidis | B | ST-41/44 complex | No value | France | SV-10 | Opc+ |
| 39971 | LNP27382 | Neisseria meningitidis | B | ST-32 complex | No value | France | SV-1 | Opc+ |
| 39972 | LNP27384 | Neisseria meningitidis | B | ST-41/44 complex | No value | France | SV-2 | Opc+ |
| 39973 | LNP27383 | Neisseria meningitidis | B | ST-269 complex | No value | France | SV-1 | Opc+ |
| 39974 | LNP27386 | Neisseria meningitidis | B | ST-41/44 complex | No value | France | SV-10 | Opc+ |
| 39975 | LNP27387 | Neisseria meningitidis | B | ST-41/44 complex | No value | France | SV-2 | Opc+ |
| 39976 | LNP27388 | Neisseria meningitidis | B | ST-41/44 complex | No value | France | SV-6 | Opc+ |
| 39977 | LNP27391 | Neisseria meningitidis | B | ST-41/44 complex | No value | France | SV-10 | Opc+ |
| 39978 | LNP27389 | Neisseria meningitidis | B | ST-461 complex | No value | France | SV-1 | Opc- |
| 39979 | LNP27392 | Neisseria meningitidis | B | ST-41/44 complex | No value | France | SV-6 | Opc+ |
| 39980 | LNP27393 | Neisseria meningitidis | B | ST-32 complex | No value | France | SV-1 | Opc+ |
| 39981 | LNP27403 | Neisseria meningitidis | B | ST-162 complex | No value | France | SV-1 | Opc+ |
| 39982 | LNP27406 | Neisseria meningitidis | B | No value | No value | France | SV-8 | Opc+ |
| 39983 | LNP27402 | Neisseria meningitidis | B | ST-32 complex | No value | France | SV-1 | Opc+ |
| 39984 | LNP27408 | Neisseria meningitidis | B | ST-461 complex | No value | France | SV-1 | Opc- |
| 39985 | LNP27407 | Neisseria meningitidis | B | ST-32 complex | No value | France | SV-1 | Opc+ |
| 39986 | LNP27415 | Neisseria meningitidis | B | No value | No value | France | SV-1 | Opc+ |
| 39987 | LNP27419 | Neisseria meningitidis | B | ST-41/44 complex | No value | France | SV-2 | Opc+ |
| 39988 | LNP27420 | Neisseria meningitidis | B | ST-41/44 complex | No value | France | SV-6 | Opc+ |
| 39989 | LNP27422 | Neisseria meningitidis | B | ST-32 complex | No value | France | SV-1 | Opc+ |
| 39990 | LNP27423 | Neisseria meningitidis | B | ST-32 complex | No value | France | SV-1 | Opc+ |
| 39991 | LNP27425 | Neisseria meningitidis | B | ST-32 complex | No value | France | SV-1 | Opc+ |
| 39992 | LNP27427 | Neisseria meningitidis | B | ST-32 complex | No value | France | SV-1 | Opc+ |
| 39993 | LNP27432 | Neisseria meningitidis | B | ST-41/44 complex | No value | France | SV-6 | Opc+ |
| 39994 | LNP27438 | Neisseria meningitidis | B | ST-32 complex | No value | France | SV-1 | Opc+ |
| 39995 | LNP27439 | Neisseria meningitidis | B | ST-461 complex | No value | France | SV-1 | Opc- |
| 39996 | LNP27437 | Neisseria meningitidis | B | ST-35 complex | No value | France | SV-16 | Opc+ |
| 39997 | LNP27441 | Neisseria meningitidis | B | ST-269 complex | No value | France | SV-1 | Opc+ |
| 39998 | LNP27451 | Neisseria meningitidis | B | ST-22 complex | No value | France | SV-12 | Opc+ |
| 39999 | LNP27455 | Neisseria meningitidis | B | ST-162 complex | No value | France | SV-1 | Opc+ |

| | | | | | | | | |
|---|---|---|---|---|---|---|---|---|
| 40000 | LNP27458 | Neisseria meningitidis | B | ST-162 complex | No value | France | SV-1 | Opc+ |
| 40001 | LNP27462 | Neisseria meningitidis | B | ST-32 complex | No value | France | SV-1 | Opc+ |
| 40002 | LNP27461 | Neisseria meningitidis | B | ST-162 complex | No value | France | SV-6 | Opc+ |
| 40003 | LNP27468 | Neisseria meningitidis | B | ST-32 complex | No value | France | SV-1 | Opc+ |
| 40004 | LNP27469 | Neisseria meningitidis | B | ST-32 complex | No value | France | SV-1 | Opc+ |
| 40005 | LNP27466 | Neisseria meningitidis | B | ST-41/44 complex | No value | France | SV-10 | Opc+ |
| 40006 | LNP27472 | Neisseria meningitidis | B | ST-11 complex | No value | France | SV-2 | Opc- |
| 40008 | LNP27477 | Neisseria meningitidis | B | ST-32 complex | No value | France | SV-1 | Opc+ |
| 40009 | LNP27478 | Neisseria meningitidis | B | ST-41/44 complex | No value | France | SV-6 | Opc+ |
| 40010 | LNP27485 | Neisseria meningitidis | B | ST-41/44 complex | No value | France | SV-10 | Opc+ |
| 40011 | LNP27486 | Neisseria meningitidis | B | ST-41/44 complex | No value | France | SV-2 | Opc+ |
| 40012 | LNP27491 | Neisseria meningitidis | B | ST-269 complex | No value | France | SV-1 | Opc+ |
| 40014 | LNP27496 | Neisseria meningitidis | B | ST-269 complex | No value | France | SV-1 | Opc+ |
| 40015 | LNP27501 | Neisseria meningitidis | B | ST-461 complex | No value | France | SV-1 | Opc- |
| 40016 | LNP27499 | Neisseria meningitidis | B | ST-269 complex | No value | France | SV-8 | Opc+ |
| 40017 | LNP27502 | Neisseria meningitidis | B | ST-41/44 complex | No value | France | SV-6 | Opc+ |
| 40018 | LNP27507 | Neisseria meningitidis | B | ST-32 complex | No value | France | SV-1 | Opc+ |
| 40019 | LNP27506 | Neisseria meningitidis | B | No value | No value | France | SV-5 | Opc- |
| 40020 | LNP27512 | Neisseria meningitidis | B | ST-461 complex | No value | France | SV-1 | Opc- |
| 40021 | LNP27509 | Neisseria meningitidis | B | ST-269 complex | No value | France | SV-1 | Opc+ |
| 40022 | LNP27523 | Neisseria meningitidis | B | ST-41/44 complex | No value | France | SV-6 | Opc+ |
| 40023 | LNP27529 | Neisseria meningitidis | B | ST-32 complex | No value | France | SV-1 | Opc+ |
| 40024 | LNP27525 | Neisseria meningitidis | B | ST-41/44 complex | No value | France | SV-6 | Opc+ |
| 40025 | LNP27532 | Neisseria meningitidis | B | ST-60 complex | No value | France | SV-11 | Opc+ |
| 40026 | LNP27533 | Neisseria meningitidis | B | ST-41/44 complex | No value | France | SV-11 | Opc- |
| 40027 | LNP27537 | Neisseria meningitidis | B | ST-41/44 complex | No value | France | SV-10 | Opc+ |
| 40028 | LNP27538 | Neisseria meningitidis | B | ST-41/44 complex | No value | France | SV-10 | Opc+ |
| 40029 | LNP27539 | Neisseria meningitidis | B | ST-32 complex | No value | France | SV-1 | Opc+ |
| 40030 | LNP27536 | Neisseria meningitidis | B | ST-41/44 complex | No value | France | SV-2 | Opc+ |
| 40031 | LNP27540 | Neisseria meningitidis | B | ST-32 complex | No value | France | SV-1 | Opc+ |
| 40032 | LNP27546 | Neisseria meningitidis | B | ST-461 complex | No value | France | SV-1 | Opc- |
| 40033 | LNP27547 | Neisseria meningitidis | B | ST-11 complex | No value | France | SV-2 | Opc- |
| 40034 | LNP27555 | Neisseria meningitidis | B | No value | No value | France | SV-1 | Opc+ |
| 40035 | LNP27559 | Neisseria meningitidis | B | ST-269 complex | No value | France | SV-1 | Opc+ |
| 40036 | LNP27557 | Neisseria meningitidis | B | ST-11 complex | No value | France | SV-2 | Opc- |
| 40037 | LNP27561 | Neisseria meningitidis | B | No value | No value | France | SV-6 | Opc+ |
| 40038 | LNP27560 | Neisseria meningitidis | B | ST-269 complex | No value | France | SV-8 | Opc+ |
| 40039 | LNP27567 | Neisseria meningitidis | B | ST-41/44 complex | No value | France | SV-6 | Opc+ |
| 40040 | LNP27569 | Neisseria meningitidis | B | ST-41/44 complex | No value | France | SV-10 | Opc+ |
| 40041 | LNP27571 | Neisseria meningitidis | B | ST-32 complex | No value | France | SV-1 | Opc+ |
| 40042 | LNP27575 | Neisseria meningitidis | B | No value | No value | France | SV-12 | Opc- |
| 40043 | LNP27573 | Neisseria meningitidis | B | ST-865 complex | No value | France | SV-5 | Opc+ |
| 40044 | LNP27576 | Neisseria meningitidis | B | ST-269 complex | No value | France | SV-1 | Opc+ |
| 40045 | LNP27584 | Neisseria meningitidis | B | ST-32 complex | No value | France | SV-1 | Opc+ |
| 40046 | LNP27580 | Neisseria meningitidis | B | ST-213 complex | No value | France | SV-2 | Opc- |
| 40047 | LNP27585 | Neisseria meningitidis | B | ST-41/44 complex | No value | France | SV-6 | Opc+ |
| 40048 | LNP27586 | Neisseria meningitidis | B | ST-32 complex | No value | France | SV-1 | Opc+ |
| 40049 | LNP27587 | Neisseria meningitidis | B | ST-11 complex | No value | France | SV-2 | Opc- |
| 40050 | LNP27589 | Neisseria meningitidis | B | ST-32 complex | No value | France | SV-1 | Opc+ |
| 40051 | LNP27588 | Neisseria meningitidis | B | ST-41/44 complex | No value | France | SV-6 | Opc+ |
| 40052 | LNP27593 | Neisseria meningitidis | B | ST-41/44 complex | No value | France | SV-10 | Opc+ |
| 40053 | LNP27595 | Neisseria meningitidis | B | ST-213 complex | No value | France | SV-12 | Opc- |
| 40054 | LNP27598 | Neisseria meningitidis | B | ST-32 complex | No value | France | SV-1 | Opc+ |
| 40055 | LNP27601 | Neisseria meningitidis | B | ST-213 complex | No value | France | SV-1 | Opc- |
| 40056 | LNP27599 | Neisseria meningitidis | B | ST-269 complex | No value | France | SV-8 | Opc+ |
| 40057 | LNP27604 | Neisseria meningitidis | B | ST-213 complex | No value | France | SV-12 | Opc- |
| 40058 | LNP27603 | Neisseria meningitidis | B | ST-162 complex | No value | France | SV-1 | Opc+ |
| 40059 | LNP27610 | Neisseria meningitidis | B | ST-41/44 complex | No value | France | SV-2 | Opc+ |
| 40060 | LNP27612 | Neisseria meningitidis | B | ST-213 complex | No value | France | SV-2 | Opc- |
| 40061 | LNP27613 | Neisseria meningitidis | B | ST-41/44 complex | No value | France | SV-10 | Opc+ |
| 40062 | LNP27615 | Neisseria meningitidis | B | ST-461 complex | No value | France | SV-1 | Opc- |
| 40063 | LNP27629 | Neisseria meningitidis | B | ST-32 complex | No value | France | SV-1 | Opc+ |
| 40064 | LNP27627 | Neisseria meningitidis | B | ST-41/44 complex | No value | France | SV-6 | Opc+ |
| 40065 | LNP27626 | Neisseria meningitidis | B | ST-269 complex | No value | France | SV-1 | Opc+ |
| 40066 | LNP27631 | Neisseria meningitidis | B | No value | No value | France | SV-1 | Opc+ |
| 40067 | LNP27633 | Neisseria meningitidis | B | ST-41/44 complex | No value | France | SV-2 | Opc+ |
| 40068 | LNP27634 | Neisseria meningitidis | B | ST-41/44 complex | No value | France | SV-2 | Opc+ |
| 40069 | LNP27637 | Neisseria meningitidis | B | No value | No value | France | SV-10 | Opc+ |
| 40070 | LNP27642 | Neisseria meningitidis | B | ST-41/44 complex | No value | France | SV-5 | Opc+ |
| 40071 | LNP27646 | Neisseria meningitidis | B | ST-213 complex | No value | France | SV-12 | Opc- |
| 40072 | LNP27649 | Neisseria meningitidis | B | ST-32 complex | No value | France | SV-1 | Opc+ |
| 40073 | LNP27650 | Neisseria meningitidis | B | ST-269 complex | No value | France | SV-1 | Opc+ |
| 40074 | LNP27647 | Neisseria meningitidis | B | No value | No value | France | SV-2 | Opc+ |
| 40075 | LNP27652 | Neisseria meningitidis | B | ST-41/44 complex | No value | France | SV-10 | Opc+ |

| | | | | | | | | |
|---|---|---|---|---|---|---|---|---|
| 40076 | LNP27653 | Neisseria meningitidis | B | ST-32 complex | No value | France | SV-1 | Opc+ |
| 40077 | LNP27654 | Neisseria meningitidis | B | ST-32 complex | No value | France | SV-1 | Opc+ |
| 40078 | LNP27656 | Neisseria meningitidis | B | ST-41/44 complex | No value | France | SV-6 | Opc+ |
| 40079 | LNP27662 | Neisseria meningitidis | B | ST-32 complex | No value | France | SV-1 | Opc+ |
| 40080 | LNP27661 | Neisseria meningitidis | B | No value | No value | France | SV-5 | Opc+ |
| 40081 | LNP27666 | Neisseria meningitidis | B | ST-32 complex | No value | France | SV-1 | Opc+ |
| 40082 | LNP27673 | Neisseria meningitidis | B | ST-41/44 complex | No value | France | SV-6 | Opc+ |
| 40083 | LNP27674 | Neisseria meningitidis | B | No value | No value | France | SV-1 | Opc+ |
| 40084 | LNP27675 | Neisseria meningitidis | B | ST-213 complex | No value | France | SV-12 | Opc- |
| 40085 | LNP27678 | Neisseria meningitidis | B | ST-41/44 complex | No value | France | SV-2 | Opc+ |
| 40086 | LNP27676 | Neisseria meningitidis | B | ST-32 complex | No value | France | SV-1 | Opc+ |
| 40087 | LNP27685 | Neisseria meningitidis | B | ST-32 complex | No value | France | SV-1 | Opc+ |
| 40088 | LNP27690 | Neisseria meningitidis | B | ST-41/44 complex | No value | France | SV-2 | Opc+ |
| 40089 | LNP27695 | Neisseria meningitidis | B | No value | No value | France | SV-1 | Opc- |
| 40090 | LNP27694 | Neisseria meningitidis | B | No value | No value | France | SV-5 | Opc+ |
| 40091 | LNP27697 | Neisseria meningitidis | B | ST-162 complex | No value | France | SV-1 | Opc+ |
| 40092 | LNP27700 | Neisseria meningitidis | B | No value | No value | France | SV-1 | Opc+ |
| 40093 | LNP27708 | Neisseria meningitidis | B | ST-1136 complex | No value | France | SV-2 | Opc+ |
| 40094 | LNP27712 | Neisseria meningitidis | B | ST-32 complex | No value | France | SV-1 | Opc+ |
| 40095 | LNP27725 | Neisseria meningitidis | B | ST-32 complex | No value | France | SV-1 | Opc+ |
| 40096 | LNP27732 | Neisseria meningitidis | B | ST-213 complex | No value | France | SV-12 | Opc- |
| 40099 | LNP27321 | Neisseria meningitidis | B | ST-35 complex | No value | France | SV-12 | Opc+ |
| 40100 | LNP27322 | Neisseria meningitidis | B | No value | No value | France | SV-6 | Opc+ |
| 40101 | LNP27406 | Neisseria meningitidis | B | No value | No value | France | SV-8 | Opc+ |
| 40102 | LNP27329 | Neisseria meningitidis | B | ST-269 complex | No value | France | SV-2 | Opc+ |
| 40103 | LNP27439 | Neisseria meningitidis | B | ST-461 complex | No value | France | SV-1 | Opc- |
| 40104 | LNP27305 | Neisseria meningitidis | B | ST-41/44 complex | No value | France | SV-10 | Opc+ |
| 40105 | LNP27340 | Neisseria meningitidis | B | ST-269 complex | No value | France | SV-8 | Opc+ |
| 40106 | LNP27338 | Neisseria meningitidis | B | ST-32 complex | No value | France | SV-1 | Opc+ |
| 40107 | LNP27337 | Neisseria meningitidis | B | ST-32 complex | No value | France | SV-1 | Opc+ |
| 40108 | LNP27336 | Neisseria meningitidis | B | ST-35 complex | No value | France | SV-12 | Opc+ |
| 40109 | LNP27407 | Neisseria meningitidis | B | ST-32 complex | No value | France | SV-1 | Opc+ |
| 40110 | LNP27296 | Neisseria meningitidis | B | ST-32 complex | No value | France | SV-1 | Opc+ |
| 40111 | LNP27334 | Neisseria meningitidis | B | ST-32 complex | No value | France | SV-1 | Opc+ |
| 40112 | LNP27363 | Neisseria meningitidis | B | ST-213 complex | No value | France | SV-12 | Opc- |
| 40113 | LNP27372 | Neisseria meningitidis | B | ST-32 complex | No value | France | SV-1 | Opc+ |
| 40114 | LNP27382 | Neisseria meningitidis | B | ST-32 complex | No value | France | SV-1 | Opc+ |
| 40115 | LNP27393 | Neisseria meningitidis | B | ST-32 complex | No value | France | SV-1 | Opc+ |
| 40116 | LNP27419 | Neisseria meningitidis | B | ST-41/44 complex | No value | France | SV-2 | Opc+ |
| 40117 | LNP27420 | Neisseria meningitidis | B | ST-41/44 complex | No value | France | SV-6 | Opc+ |
| 40118 | LNP27422 | Neisseria meningitidis | B | ST-32 complex | No value | France | SV-1 | Opc+ |
| 40119 | LNP27437 | Neisseria meningitidis | B | ST-35 complex | No value | France | SV-16 | Opc+ |
| 40120 | LNP27438 | Neisseria meningitidis | B | ST-32 complex | No value | France | SV-1 | Opc+ |
| 40121 | LNP27441 | Neisseria meningitidis | B | ST-269 complex | No value | France | SV-1 | Opc+ |
| 40122 | LNP27462 | Neisseria meningitidis | B | ST-32 complex | No value | France | SV-1 | Opc+ |
| 40123 | LNP27469 | Neisseria meningitidis | B | ST-32 complex | No value | France | SV-1 | Opc+ |
| 40124 | LNP27472 | Neisseria meningitidis | B | ST-11 complex | No value | France | SV-2 | Opc- |
| 40125 | LNP27477 | Neisseria meningitidis | B | ST-32 complex | No value | France | SV-1 | Opc+ |
| 40126 | LNP27525 | Neisseria meningitidis | B | ST-41/44 complex | No value | France | SV-6 | Opc+ |
| 40127 | LNP27532 | Neisseria meningitidis | B | ST-60 complex | No value | France | SV-11 | Opc+ |
| 40128 | LNP27547 | Neisseria meningitidis | B | ST-11 complex | No value | France | SV-2 | Opc- |
| 40129 | LNP27571 | Neisseria meningitidis | B | ST-32 complex | No value | France | SV-1 | Opc+ |
| 40130 | LNP27573 | Neisseria meningitidis | B | ST-865 complex | No value | France | SV-5 | Opc+ |
| 40131 | LNP27584 | Neisseria meningitidis | B | ST-32 complex | No value | France | SV-1 | Opc+ |
| 40132 | LNP27587 | Neisseria meningitidis | B | ST-11 complex | No value | France | SV-2 | Opc- |
| 40133 | LNP27589 | Neisseria meningitidis | B | ST-32 complex | No value | France | SV-1 | Opc+ |
| 40134 | LNP27598 | Neisseria meningitidis | B | ST-32 complex | No value | France | SV-1 | Opc+ |
| 40135 | LNP27652 | Neisseria meningitidis | B | ST-41/44 complex | No value | France | SV-10 | Opc+ |
| 40136 | LNP27653 | Neisseria meningitidis | B | ST-32 complex | No value | France | SV-1 | Opc+ |
| 40137 | LNP27654 | Neisseria meningitidis | B | ST-32 complex | No value | France | SV-1 | Opc+ |
| 40138 | LNP27656 | Neisseria meningitidis | B | ST-41/44 complex | No value | France | SV-6 | Opc+ |
| 40139 | LNP27661 | Neisseria meningitidis | B | No value | No value | France | SV-5 | Opc- |
| 40140 | LNP27662 | Neisseria meningitidis | B | ST-32 complex | No value | France | SV-1 | Opc+ |
| 40141 | LNP27678 | Neisseria meningitidis | B | ST-41/44 complex | No value | France | SV-2 | Opc+ |
| 40142 | LNP27685 | Neisseria meningitidis | B | ST-32 complex | No value | France | SV-1 | Opc+ |
| 40143 | LNP27712 | Neisseria meningitidis | B | ST-32 complex | No value | France | SV-1 | Opc+ |
| 40144 | LNP27355 | Neisseria meningitidis | B | No value | No value | France | SV-1 | Opc+ |
| 40145 | LNP27694 | Neisseria meningitidis | B | No value | No value | France | SV-5 | Opc+ |
| 40146 | LNP27408 | Neisseria meningitidis | B | ST-461 complex | No value | France | SV-1 | Opc- |
| 40147 | LNP27367 | Neisseria meningitidis | B | No value | No value | France | SV-11 | Opc+ |
| 40148 | LNP27725 | Neisseria meningitidis | B | ST-32 complex | No value | France | SV-1 | Opc+ |
| 40149 | LNP27485 | Neisseria meningitidis | B | ST-41/44 complex | No value | France | SV-10 | Opc+ |
| 40150 | LNP27690 | Neisseria meningitidis | B | ST-41/44 complex | No value | France | SV-2 | Opc+ |
| 40151 | LNP27540 | Neisseria meningitidis | B | ST-32 complex | No value | France | SV-1 | Opc+ |

| | | | | | | | | |
|---|---|---|---|---|---|---|---|---|
| 40152 | LNP27540 | Neisseria meningitidis | B | ST-32 complex | No value | France | SV-1 | Opc+ |
| 40153 | LNP27557 | Neisseria meningitidis | B | ST-11 complex | No value | France | SV-2 | Opc- |
| 40154 | LNP27415 | Neisseria meningitidis | B | No value | No value | France | SV-1 | Opc+ |
| 40155 | LNP27676 | Neisseria meningitidis | B | ST-32 complex | No value | France | SV-1 | Opc+ |
| 40156 | LNP27631 | Neisseria meningitidis | B | ST-32 complex | No value | France | SV-1 | Opc+ |
| 40157 | LNP27612 | Neisseria meningitidis | B | ST-213 complex | No value | France | SV-2 | Opc- |
| 40158 | LNP27629 | Neisseria meningitidis | B | ST-32 complex | No value | France | SV-1 | Opc+ |
| 40159 | LNP27313 | Neisseria meningitidis | B | ST-32 complex | No value | France | SV-1 | Opc+ |
| 40160 | LNP27700 | Neisseria meningitidis | B | No value | No value | France | SV-1 | Opc+ |
| 40161 | LNP27468 | Neisseria meningitidis | B | ST-32 complex | No value | France | SV-1 | Opc+ |
| 40162 | LNP27575 | Neisseria meningitidis | B | ST-213 complex | No value | France | SV-12 | Opc- |
| 40163 | LNP27576 | Neisseria meningitidis | B | ST-269 complex | No value | France | SV-1 | Opc+ |
| 40164 | LNP27389 | Neisseria meningitidis | B | ST-461 complex | No value | France | SV-1 | Opc- |
| 40165 | LNP27391 | Neisseria meningitidis | B | ST-41/44 complex | No value | France | SV-10 | Opc+ |
| 40166 | LNP27392 | Neisseria meningitidis | B | ST-41/44 complex | No value | France | SV-6 | Opc+ |
| 40167 | LNP27275 | Neisseria meningitidis | B | ST-461 complex | No value | France | SV-1 | Opc- |
| 40168 | LNP27402 | Neisseria meningitidis | B | ST-32 complex | No value | France | SV-1 | Opc+ |
| 40169 | LNP27349 | Neisseria meningitidis | B | ST-269 complex | No value | France | SV-1 | Opc+ |
| 40170 | LNP27274 | Neisseria meningitidis | B | ST-60 complex | No value | France | SV-11 | Opc+ |
| 40171 | LNP27403 | Neisseria meningitidis | B | ST-162 complex | No value | France | SV-1 | Opc+ |
| 40172 | LNP27270 | Neisseria meningitidis | B | ST-41/44 complex | No value | France | SV-2 | Opc+ |
| 40173 | LNP27272 | Neisseria meningitidis | B | ST-461 complex | No value | France | SV-1 | Opc- |
| 40174 | LNP27342 | Neisseria meningitidis | B | No value | No value | France | SV-2 | Opc+ |
| 40175 | LNP27344 | Neisseria meningitidis | B | ST-269 complex | No value | France | SV-8 | Opc+ |
| 40176 | LNP27347 | Neisseria meningitidis | B | ST-41/44 complex | No value | France | SV-6 | Opc+ |
| 40177 | LNP27348 | Neisseria meningitidis | B | ST-41/44 complex | No value | France | SV-10 | Opc+ |
| 40178 | LNP27350 | Neisseria meningitidis | B | ST-32 complex | No value | France | SV-1 | Opc+ |
| 40179 | LNP27386 | Neisseria meningitidis | B | ST-41/44 complex | No value | France | SV-10 | Opc+ |
| 40180 | LNP27384 | Neisseria meningitidis | B | ST-41/44 complex | No value | France | SV-2 | Opc+ |
| 40181 | LNP27286 | Neisseria meningitidis | B | ST-32 complex | No value | France | SV-1 | Opc+ |
| 40182 | LNP27369 | Neisseria meningitidis | B | ST-162 complex | No value | France | SV-1 | Opc+ |
| 40183 | LNP27332 | Neisseria meningitidis | B | ST-41/44 complex | No value | France | SV-6 | Opc+ |
| 40184 | LNP27387 | Neisseria meningitidis | B | ST-41/44 complex | No value | France | SV-2 | Opc+ |
| 40185 | LNP27466 | Neisseria meningitidis | B | ST-41/44 complex | No value | France | SV-10 | Opc+ |
| 40186 | LNP27461 | Neisseria meningitidis | B | ST-162 complex | No value | France | SV-6 | Opc+ |
| 40187 | LNP27458 | Neisseria meningitidis | B | ST-162 complex | No value | France | SV-1 | Opc+ |
| 40188 | LNP27325 | Neisseria meningitidis | B | ST-41/44 complex | No value | France | SV-10 | Opc+ |
| 40189 | LNP27327 | Neisseria meningitidis | B | ST-41/44 complex | No value | France | SV-6 | Opc+ |
| 40190 | LNP27451 | Neisseria meningitidis | B | ST-22 complex | No value | France | SV-12 | Opc+ |
| 40191 | LNP27311 | Neisseria meningitidis | B | ST-41/44 complex | No value | France | SV-6 | Opc+ |
| 40192 | LNP27306 | Neisseria meningitidis | B | ST-461 complex | No value | France | SV-1 | Opc- |
| 40193 | LNP27302 | Neisseria meningitidis | B | ST-41/44 complex | No value | France | SV-2 | Opc+ |
| 40194 | LNP27297 | Neisseria meningitidis | B | ST-35 complex | No value | France | SV-12 | Opc+ |
| 40195 | LNP27293 | Neisseria meningitidis | B | ST-32 complex | No value | France | SV-1 | Opc+ |
| 40196 | LNP27291 | Neisseria meningitidis | B | ST-162 complex | No value | France | SV-1 | Opc+ |
| 40197 | LNP27561 | Neisseria meningitidis | B | ST-41/44 complex | No value | France | SV-6 | Opc+ |
| 40202 | LNP27684 | Neisseria meningitidis | B | ST-11 complex | No value | France | SV-2 | Opc- |
| 40203 | LNP28262 | Neisseria meningitidis | B | ST-32 complex | No value | France | SV-1 | Opc+ |
| 40204 | LNP28263 | Neisseria meningitidis | B | ST-41/44 complex | No value | France | SV-14 | Opc+ |
| 40205 | LNP28264 | Neisseria meningitidis | B | ST-269 complex | No value | France | SV-8 | Opc+ |
| 40206 | LNP28265 | Neisseria meningitidis | Y | ST-23 complex | No value | France | SV-11 | Opc+ |
| 40207 | LNP28266 | Neisseria meningitidis | NG | No value | No value | France | SV-7 | Opc+ |
| 40208 | LNP28267 | Neisseria meningitidis | W | ST-53 complex | No value | France | SV-2 | Opc- |
| 40209 | LNP28268 | Neisseria meningitidis | Y | ST-23 complex | No value | France | SV-11 | Opc+ |
| 40210 | LNP28270 | Neisseria meningitidis | C | No value | No value | France | SV-2 | Opc- |
| 40211 | LNP28271 | Neisseria meningitidis | B | ST-269 complex | No value | France | SV-1 | Opc+ |
| 40212 | LNP28274 | Neisseria meningitidis | Y | ST-23 complex | No value | France | SV-7 | Opc+ |
| 40213 | LNP28276 | Neisseria meningitidis | C | ST-11 complex | No value | France | SV-2 | Opc- |
| 40214 | LNP28277 | Neisseria meningitidis | B | ST-41/44 complex | No value | France | SV-6 | Opc+ |
| 40215 | LNP28278 | Neisseria meningitidis | B | ST-162 complex | No value | France | SV-1 | Opc+ |
| 40216 | LNP28279 | Neisseria meningitidis | C | ST-162 complex | No value | France | SV-1 | Opc+ |
| 40217 | LNP28281 | Neisseria meningitidis | Y | ST-103 complex | No value | France | SV-5 | Opc+ |
| 40218 | LNP28282 | Neisseria meningitidis | B | ST-461 complex | No value | France | SV-1 | Opc- |
| 40219 | LNP28283 | Neisseria meningitidis | B | ST-32 complex | No value | France | SV-1 | Opc+ |
| 40220 | LNP28284 | Neisseria meningitidis | C | ST-461 complex | No value | France | SV-1 | Opc- |
| 40221 | LNP28285 | Neisseria meningitidis | B | ST-32 complex | No value | France | SV-1 | Opc+ |
| 40222 | LNP28286 | Neisseria meningitidis | C | ST-11 complex | No value | France | SV-2 | Opc- |
| 40223 | LNP28287 | Neisseria meningitidis | B | ST-269 complex | No value | France | SV-1 | Opc+ |
| 40224 | LNP28288 | Neisseria meningitidis | B | ST-269 complex | No value | France | SV-1 | Opc+ |
| 40225 | LNP28289 | Neisseria meningitidis | C | ST-11 complex | No value | France | SV-2 | Opc- |
| 40226 | LNP28290 | Neisseria meningitidis | C | ST-213 complex | No value | France | SV-12 | Opc- |
| 40227 | LNP28291 | Neisseria meningitidis | B | ST-11 complex | No value | France | SV-2 | Opc- |
| 40228 | LNP28292 | Neisseria meningitidis | C | ST-11 complex | No value | France | SV-2 | Opc- |
| 40229 | LNP28293 | Neisseria meningitidis | W | ST-22 complex | No value | France | SV-12 | Opc+ |

| | | | | | | | | |
|---|---|---|---|---|---|---|---|---|
| 40230 | LNP28294 | Neisseria meningitidis | B | ST-41/44 complex | No value | France | SV-6 | Opc+ |
| 40231 | LNP28295 | Neisseria meningitidis | C | ST-11 complex | No value | France | SV-2 | Opc- |
| 40232 | LNP28296 | Neisseria meningitidis | B | ST-213 complex | No value | France | SV-12 | Opc- |
| 40233 | LNP28297 | Neisseria meningitidis | B | ST-269 complex | No value | France | SV-1 | Opc+ |
| 40234 | LNP28298 | Neisseria meningitidis | B | ST-11 complex | No value | France | SV-2 | Opc- |
| 40235 | LNP28299 | Neisseria meningitidis | Y | ST-23 complex | No value | France | SV-11 | Opc+ |
| 40236 | LNP28300 | Neisseria meningitidis | NG | ST-1136 complex | No value | France | SV-2 | Opc+ |
| 40237 | LNP28301 | Neisseria meningitidis | C | ST-11 complex | No value | France | SV-2 | Opc- |
| 40238 | LNP28302 | Neisseria meningitidis | B | ST-41/44 complex | No value | France | SV-2 | Opc+ |
| 40239 | LNP28303 | Neisseria meningitidis | C | ST-11 complex | No value | France | SV-2 | Opc- |
| 40240 | LNP28305 | Neisseria meningitidis | B | ST-41/44 complex | No value | France | SV-11 | Opc+ |
| 40241 | LNP28307 | Neisseria meningitidis | B | ST-41/44 complex | No value | France | SV-2 | Opc+ |
| 40242 | LNP28308 | Neisseria meningitidis | E | ST-60 complex | No value | France | SV-11 | Opc+ |
| 40243 | LNP28309 | Neisseria meningitidis | W | ST-22 complex | No value | France | SV-12 | Opc+ |
| 40244 | LNP28310 | Neisseria meningitidis | B | ST-41/44 complex | No value | France | SV-6 | Opc+ |
| 40245 | LNP28311 | Neisseria meningitidis | Y | ST-23 complex | No value | France | SV-11 | Opc+ |
| 40247 | LNP28313 | Neisseria meningitidis | B | ST-32 complex | No value | France | SV-1 | Opc+ |
| 40249 | LNP28316 | Neisseria meningitidis | Y | ST-174 complex | No value | France | SV-1 | Opc+ |
| 40250 | LNP28317 | Neisseria meningitidis | Y | ST-23 complex | No value | France | SV-11 | Opc+ |
| 40251 | LNP28318 | Neisseria meningitidis | Y | ST-167 complex | No value | France | SV-5 | Opc+ |
| 40252 | LNP28319 | Neisseria meningitidis | B | ST-269 complex | No value | France | SV-1 | Opc+ |
| 40253 | LNP28320 | Neisseria meningitidis | B | ST-32 complex | No value | France | SV-1 | Opc+ |
| 40254 | LNP28321 | Neisseria meningitidis | B | ST-162 complex | No value | France | SV-1 | Opc+ |
| 40255 | LNP28322 | Neisseria meningitidis | B | ST-213 complex | No value | France | SV-12 | Opc- |
| 40256 | LNP28323 | Neisseria meningitidis | B | ST-269 complex | No value | France | SV-8 | Opc+ |
| 40257 | LNP28324 | Neisseria meningitidis | B | ST-1136 complex | No value | France | SV-2 | Opc+ |
| 40258 | LNP28327 | Neisseria meningitidis | B | ST-162 complex | No value | France | SV-1 | Opc+ |
| 40259 | LNP28329 | Neisseria meningitidis | Y | ST-23 complex | No value | France | SV-11 | Opc+ |
| 40260 | LNP28330 | Neisseria meningitidis | B | ST-32 complex | No value | France | SV-1 | Opc+ |
| 40262 | LNP28332 | Neisseria meningitidis | W | No value | No value | France | SV-12 | Opc+ |
| 40263 | LNP28333 | Neisseria meningitidis | B | ST-865 complex | No value | France | SV-2 | Opc+ |
| 40264 | LNP28334 | Neisseria meningitidis | B | No value | No value | France | SV-1 | Opc+ |
| 40265 | LNP28335 | Neisseria meningitidis | B | ST-213 complex | No value | France | SV-12 | Opc- |
| 40266 | LNP28336 | Neisseria meningitidis | B | No value | No value | France | SV-1 | Opc+ |
| 40267 | LNP28337 | Neisseria meningitidis | C | ST-11 complex | No value | France | SV-2 | Opc- |
| 40268 | LNP28338 | Neisseria meningitidis | C | ST-11 complex | No value | France | SV-2 | Opc- |
| 40269 | LNP28339 | Neisseria meningitidis | Y | ST-23 complex | No value | France | SV-11 | Opc+ |
| 40271 | LNP28341 | Neisseria meningitidis | Y | No value | No value | France | SV-5 | Opc+ |
| 40272 | LNP28342 | Neisseria meningitidis | B | ST-11 complex | No value | France | SV-2 | Opc- |
| 40273 | LNP28343 | Neisseria meningitidis | Y | ST-167 complex | No value | France | SV-5 | Opc+ |
| 40274 | LNP28344 | Neisseria meningitidis | C | ST-11 complex | No value | France | SV-2 | Opc- |
| 40275 | LNP28345 | Neisseria meningitidis | NG | ST-1136 complex | No value | France | SV-2 | Opc+ |
| 40276 | LNP28346 | Neisseria meningitidis | C | ST-11 complex | No value | France | SV-2 | Opc- |
| 40277 | LNP28347 | Neisseria meningitidis | B | ST-41/44 complex | No value | France | SV-2 | Opc+ |
| 40278 | LNP28348 | Neisseria meningitidis | C | ST-11 complex | No value | France | SV-2 | Opc- |
| 40279 | LNP28349 | Neisseria meningitidis | W | ST-11 complex | No value | France | SV-2 | Opc- |
| 40280 | LNP28350 | Neisseria meningitidis | B | No value | No value | France | SV-8 | Opc+ |
| 40281 | LNP28351 | Neisseria meningitidis | B | ST-269 complex | No value | France | SV-1 | Opc+ |
| 40287 | 12-539 | Neisseria meningitidis | Y | ST-23 complex | No value | Sweden | SV-11 | Opc+ |
| 40288 | 12-543 | Neisseria meningitidis | Y | ST-23 complex | No value | Sweden | SV-11 | Opc+ |
| 40289 | 12-588 | Neisseria meningitidis | Y | ST-23 complex | No value | Sweden | SV-11 | Opc+ |
| 40290 | LNP28352 | Neisseria meningitidis | Y | ST-23 complex | No value | France | SV-11 | Opc+ |
| 40291 | Nov-54 | Neisseria meningitidis | Y | ST-23 complex | No value | Sweden | SV-11 | Opc+ |
| 40292 | 05-231 | Neisseria meningitidis | Y | ST-23 complex | No value | Sweden | SV-11 | Opc+ |
| 40293 | LNP28126 | Neisseria meningitidis | C | ST-11 complex | No value | France | SV-2 | Opc- |
| 40294 | LNP27575 | Neisseria meningitidis | B | ST-213 complex | No value | France | SV-12 | Opc- |
| 40296 | LNP27494 | Neisseria meningitidis | B | ST-32 complex | No value | France | SV-1 | Opc+ |
| 40297 | 2551 | Neisseria meningitidis | Y | ST-23 complex | Sporadic case | Italy | SV-11 | Opc+ |
| 40298 | 2581 | Neisseria meningitidis | W | ST-22 complex | Sporadic case | Italy | SV-12 | Opc+ |
| 40299 | LNP27419 | Neisseria meningitidis | B | ST-41/44 complex | No value | France | SV-6 | Opc+ |
| 40300 | LNP27420 | Neisseria meningitidis | B | ST-41/44 complex | No value | France | SV-2 | Opc+ |
| 40301 | WS74947 M99241396 | Neisseria meningitidis | NG | ST-60 complex | No value | UK | SV-11 | Opc+ |
| 40302 | WS74947 M01242679 | Neisseria meningitidis | W | ST-11 complex | No value | UK | SV-2 | Opc- |
| 40303 | WS74947 M98250375 | Neisseria meningitidis | B | ST-32 complex | No value | UK | SV-1 | Opc+ |
| 40304 | WS74947 M98250840 | Neisseria meningitidis | B | ST-60 complex | No value | UK | SV-11 | Opc+ |
| 40305 | WS74947 M98252086 | Neisseria meningitidis | B | ST-41/44 complex | No value | UK | SV-5 | Opc- |
| 40306 | WS74947 M00240207 | Neisseria meningitidis | NG | ST-41/44 complex | No value | UK | SV-2 | Opc+ |
| 40307 | WS74947 M00240993 | Neisseria meningitidis | B | ST-41/44 complex | No value | UK | SV-2 | Opc+ |
| 40308 | WS74947 M02240168 | Neisseria meningitidis | B | ST-269 complex | No value | UK | SV-8 | Opc+ |
| 40309 | WS74947 M05240177 | Neisseria meningitidis | B | ST-32 complex | No value | UK | SV-1 | Opc+ |
| 40310 | WS74947 M09240022 | Neisseria meningitidis | B | ST-32 complex | No value | UK | SV-1 | Opc+ |
| 40311 | WS74947 M98253069 | Neisseria meningitidis | B | ST-41/44 complex | No value | UK | SV-2 | Opc+ |
| 40312 | WS74947 M98253766 | Neisseria meningitidis | B | ST-8 complex | No value | UK | SV-14 | Opc- |
| 40313 | WS74947 F850000045 | Neisseria meningitidis | C | ST-11 complex | No value | UK | SV-2 | Opc- |

| | | | | | | | | |
|---|---|---|---|---|---|---|---|---|
| 40314 | WS74947 F850000704 | Neisseria meningitidis | C | ST-11 complex | No value | UK | SV-2 | Opc- |
| 40315 | WS74947 F85000311 | Neisseria meningitidis | W | No value | No value | UK | SV-1 | Opc+ |
| 40316 | M15 240907 | Neisseria meningitidis | W | ST-11 complex | No value | UK | SV-2 | Opc- |
| 40317 | M15 240908 | Neisseria meningitidis | C | ST-1157 complex | No value | UK | SV-1 | Opc+ |
| 40318 | M15 240909 | Neisseria meningitidis | NG | ST-11 complex | No value | UK | SV-2 | Opc- |
| 40319 | M15 240910 | Neisseria meningitidis | C | ST-1157 complex | No value | UK | SV-1 | Opc+ |
| 40320 | M15 240921 | Neisseria meningitidis | NG | ST-41/44 complex | No value | Czech Republic | SV-6 | Opc+ |
| 40321 | M15 240851 | Neisseria meningitidis | B | ST-213 complex | No value | UK | SV-1 | Opc- |
| 40322 | M15 240852 | Neisseria meningitidis | W | ST-11 complex | No value | UK | SV-2 | Opc- |
| 40324 | M15 240854 | Neisseria meningitidis | B | ST-213 complex | No value | UK | SV-12 | Opc- |
| 40325 | 2601 | Neisseria meningitidis | B | ST-41/44 complex | Sporadic case | Italy | SV-6 | Opc+ |
| 40326 | 2606 | Neisseria meningitidis | Y | ST-23 complex | Sporadic case | Italy | SV-11 | Opc+ |
| 40327 | 2619 | Neisseria meningitidis | B | ST-269 complex | Sporadic case | Italy | SV-8 | Opc+ |
| 40328 | 2631 | Neisseria meningitidis | Y | ST-23 complex | Sporadic case | Italy | SV-11 | Opc+ |
| 40329 | 2634 | Neisseria meningitidis | B | ST-41/44 complex | Sporadic case | Italy | SV-6 | Opc+ |
| 40330 | 2683 | Neisseria meningitidis | X | ST-181 complex | Sporadic case | Italy | SV-5 | Opc+ |
| 40331 | 2704 | Neisseria meningitidis | B | ST-32 complex | Sporadic case | Italy | SV-1 | Opc+ |
| 40332 | 2724 | Neisseria meningitidis | B | No value | Sporadic case | Italy | SV-6 | Opc+ |
| 40333 | 2078 | Neisseria meningitidis | C | ST-35 complex | Sporadic case | Italy | SV-12 | Opc+ |
| 40334 | 2552 | Neisseria meningitidis | B | ST-1136 complex | Sporadic case | Italy | SV-2 | Opc+ |
| 40335 | 2558 | Neisseria meningitidis | Y | ST-23 complex | Sporadic case | Italy | SV-11 | Opc+ |
| 40336 | 2559 | Neisseria meningitidis | B | ST-1157 complex | Sporadic case | Italy | SV-1 | Opc+ |
| 40337 | 2579 | Neisseria meningitidis | B | ST-461 complex | Sporadic case | Italy | SV-1 | Opc- |
| 40338 | 2582 | Neisseria meningitidis | B | ST-41/44 complex | Sporadic case | Italy | SV-6 | Opc+ |
| 40339 | 2584 | Neisseria meningitidis | B | ST-41/44 complex | Sporadic case | Italy | SV-6 | Opc+ |
| 40340 | 2604 | Neisseria meningitidis | B | ST-41/44 complex | Sporadic case | Italy | SV-7 | Opc+ |
| 40341 | 2621 | Neisseria meningitidis | B | ST-41/44 complex | Sporadic case | Italy | SV-1 | Opc+ |
| 40342 | 2622 | Neisseria meningitidis | B | ST-41/44 complex | Sporadic case | Italy | SV-7 | Opc+ |
| 40343 | 2626 | Neisseria meningitidis | Y | ST-23 complex | Sporadic case | Italy | SV-11 | Opc+ |
| 40344 | 2628 | Neisseria meningitidis | B | ST-32 complex | Sporadic case | Italy | SV-1 | Opc+ |
| 40345 | 2638 | Neisseria meningitidis | B | ST-461 complex | Sporadic case | Italy | SV-5 | Opc- |
| 40346 | 2642 | Neisseria meningitidis | Y | ST-23 complex | Sporadic case | Italy | SV-11 | Opc+ |
| 40347 | 2649 | Neisseria meningitidis | Y | ST-23 complex | Sporadic case | Italy | SV-11 | Opc+ |
| 40348 | 2661 | Neisseria meningitidis | B | ST-41/44 complex | Sporadic case | Italy | SV-10 | Opc+ |
| 40349 | 2679 | Neisseria meningitidis | B | ST-461 complex | Sporadic case | Italy | SV-1 | Opc- |
| 40350 | 2680 | Neisseria meningitidis | B | ST-269 complex | Sporadic case | Italy | SV-1 | Opc+ |
| 40351 | 2681 | Neisseria meningitidis | Y | ST-23 complex | Sporadic case | Italy | SV-11 | Opc+ |
| 40352 | 2682 | Neisseria meningitidis | B | ST-461 complex | Sporadic case | Italy | SV-1 | Opc+ |
| 40353 | 2687 | Neisseria meningitidis | Y | ST-23 complex | Sporadic case | Italy | SV-11 | Opc+ |
| 40354 | 2688 | Neisseria meningitidis | B | ST-461 complex | Sporadic case | Italy | SV-1 | Opc- |
| 40355 | 2689 | Neisseria meningitidis | NG | ST-1157 complex | Sporadic case | Italy | SV-1 | Opc+ |
| 40356 | 2700 | Neisseria meningitidis | Y | ST-23 complex | Sporadic case | Italy | SV-11 | Opc+ |
| 40357 | 2709 | Neisseria meningitidis | B | ST-865 complex | Sporadic case | Italy | SV-2 | Opc+ |
| 40358 | 2712 | Neisseria meningitidis | B | ST-32 complex | Sporadic case | Italy | SV-1 | Opc+ |
| 40359 | 2713 | Neisseria meningitidis | Y | ST-23 complex | Sporadic case | Italy | SV-11 | Opc+ |
| 40360 | 2716 | Neisseria meningitidis | B | ST-32 complex | Sporadic case | Italy | SV-1 | Opc+ |
| 40361 | 2726 | Neisseria meningitidis | Y | ST-23 complex | Sporadic case | Italy | SV-11 | Opc+ |
| 40362 | 2732 | Neisseria meningitidis | Y | ST-23 complex | Sporadic case | Italy | SV-11 | Opc+ |
| 40382 | LNP28353 | Neisseria meningitidis | B | ST-865 complex | No value | France | SV-2 | Opc+ |
| 40383 | LNP28354 | Neisseria meningitidis | C | ST-11 complex | No value | France | SV-2 | Opc- |
| 40384 | LNP28355 | Neisseria meningitidis | B | ST-461 complex | No value | France | SV-1 | Opc- |
| 40385 | LNP28356 | Neisseria meningitidis | E | ST-60 complex | No value | France | SV-11 | Opc+ |
| 40386 | LNP28357 | Neisseria meningitidis | B | ST-41/44 complex | No value | France | SV-6 | Opc+ |
| 40387 | LNP28359 | Neisseria meningitidis | W | ST-11 complex | No value | France | SV-2 | Opc- |
| 40388 | LNP28360 | Neisseria meningitidis | C | ST-11 complex | No value | France | SV-2 | Opc- |
| 40389 | LNP28361 | Neisseria meningitidis | C | ST-11 complex | No value | Spain | SV-2 | Opc- |
| 40390 | LNP28362 | Neisseria meningitidis | B | ST-32 complex | No value | France | SV-1 | Opc+ |
| 40391 | LNP28363 | Neisseria meningitidis | W | ST-11 complex | No value | France | SV-2 | Opc- |
| 40392 | LNP28364 | Neisseria meningitidis | C | ST-11 complex | No value | France | SV-2 | Opc- |
| 40394 | LNP28366 | Neisseria meningitidis | B | ST-41/44 complex | No value | France | SV-6 | Opc+ |
| 40395 | LNP28367 | Neisseria meningitidis | C | ST-11 complex | No value | France | SV-2 | Opc- |
| 40396 | LNP28368 | Neisseria meningitidis | C | ST-11 complex | No value | France | SV-2 | Opc- |
| 40397 | LNP28369 | Neisseria meningitidis | B | ST-41/44 complex | No value | France | SV-6 | Opc+ |
| 40398 | LNP28370 | Neisseria meningitidis | B | ST-41/44 complex | No value | France | SV-6 | Opc+ |
| 40399 | LNP28371 | Neisseria meningitidis | W | ST-11 complex | No value | France | SV-2 | Opc- |
| 40400 | LNP28372 | Neisseria meningitidis | Y | ST-167 complex | No value | France | SV-5 | Opc+ |
| 40401 | LNP28374 | Neisseria meningitidis | W | ST-11 complex | No value | France | SV-2 | Opc- |
| 40402 | LNP28375 | Neisseria meningitidis | B | ST-41/44 complex | No value | France | SV-2 | Opc+ |
| 40403 | LNP28376 | Neisseria meningitidis | B | ST-269 complex | No value | France | SV-8 | Opc+ |
| 40404 | LNP28379 | Neisseria meningitidis | B | ST-461 complex | No value | France | SV-1 | Opc- |
| 40405 | LNP28380 | Neisseria meningitidis | Y | ST-23 complex | No value | France | SV-11 | Opc+ |
| 40406 | LNP28381 | Neisseria meningitidis | B | ST-213 complex | No value | France | SV-12 | Opc- |
| 40407 | LNP28382 | Neisseria meningitidis | X | ST-1157 complex | No value | France | SV-1 | Opc+ |
| 40408 | LNP28383 | Neisseria meningitidis | C | ST-11 complex | No value | France | SV-2 | Opc- |

| | | | | | | | | |
|---|---|---|---|---|---|---|---|---|
| 40409 | LNP28384 | Neisseria meningitidis | B | ST-60 complex | No value | France | SV-11 | Opc+ |
| 40410 | LNP28385 | Neisseria meningitidis | C | ST-11 complex | No value | France | SV-2 | Opc- |
| 40411 | LNP28386 | Neisseria meningitidis | B | ST-32 complex | No value | France | SV-1 | Opc+ |
| 40412 | DZ1 | Neisseria meningitidis | B | ST-213 complex | No value | Algeria | SV-12 | Opc- |
| 40413 | DZ3 | Neisseria meningitidis | B | ST-213 complex | No value | Algeria | SV-12 | Opc- |
| 40414 | DZ4 | Neisseria meningitidis | W | ST-11 complex | No value | Algeria | SV-2 | Opc- |
| 40415 | DZ5 | Neisseria meningitidis | B | ST-41/44 complex | No value | Algeria | SV-10 | Opc+ |
| 40416 | DZ7 | Neisseria meningitidis | W | ST-11 complex | No value | Algeria | SV-2 | Opc- |
| 40417 | DZ10 | Neisseria meningitidis | B | ST-41/44 complex | No value | Algeria | SV-10 | Opc+ |
| 40418 | DZ11 | Neisseria meningitidis | B | ST-41/44 complex | No value | Algeria | SV-6 | Opc+ |
| 40421 | DZ16 | Neisseria meningitidis | Y | ST-167 complex | No value | Algeria | SV-5 | Opc+ |
| 40422 | 2565 | Neisseria meningitidis | C | ST-11 complex | Endemic | Italy | SV-2 | Opc- |
| 40423 | 44133 | Neisseria meningitidis | W | ST-175 complex | No value | South Africa | SV-5 | Opc+ |
| 40424 | 44380 | Neisseria meningitidis | NG | No value | No value | South Africa | SV-1 | Opc+ |
| 40425 | 46627 | Neisseria meningitidis | NG | No value | No value | South Africa | SV-1 | Opc- |
| 40427 | LNP27296 | Neisseria meningitidis | B | ST-32 complex | No value | France | SV-1 | Opc+ |
| 40428 | LNP27334 | Neisseria meningitidis | B | ST-32 complex | No value | France | SV-1 | Opc+ |
| 40429 | LNP27363 | Neisseria meningitidis | B | ST-213 complex | No value | France | SV-12 | Opc- |
| 40430 | LNP27382 | Neisseria meningitidis | B | ST-32 complex | No value | France | SV-1 | Opc+ |
| 40431 | LNP27393 | Neisseria meningitidis | B | ST-32 complex | No value | France | SV-1 | Opc+ |
| 40432 | LNP27419 | Neisseria meningitidis | B | ST-41/44 complex | No value | France | SV-2 | Opc+ |
| 40433 | LNP27420 | Neisseria meningitidis | B | ST-41/44 complex | No value | France | SV-6 | Opc+ |
| 40434 | LNP27422 | Neisseria meningitidis | B | ST-32 complex | No value | France | SV-1 | Opc+ |
| 40435 | LNP27425 | Neisseria meningitidis | B | ST-32 complex | No value | France | SV-1 | Opc+ |
| 40436 | LNP27437 | Neisseria meningitidis | B | ST-35 complex | No value | France | SV-16 | Opc+ |
| 40437 | LNP27438 | Neisseria meningitidis | B | ST-32 complex | No value | France | SV-1 | Opc+ |
| 40438 | LNP27441 | Neisseria meningitidis | B | ST-269 complex | No value | France | SV-1 | Opc+ |
| 40439 | LNP27462 | Neisseria meningitidis | B | ST-32 complex | No value | France | SV-1 | Opc+ |
| 40440 | LNP27472 | Neisseria meningitidis | B | ST-11 complex | No value | France | SV-2 | Opc- |
| 40441 | LNP27477 | Neisseria meningitidis | B | ST-32 complex | No value | France | SV-1 | Opc+ |
| 40442 | LNP27525 | Neisseria meningitidis | B | ST-41/44 complex | No value | France | SV-6 | Opc+ |
| 40443 | LNP27532 | Neisseria meningitidis | B | ST-60 complex | No value | France | SV-11 | Opc+ |
| 40444 | LNP27540 | Neisseria meningitidis | B | ST-32 complex | No value | France | SV-1 | Opc+ |
| 40445 | LNP27573 | Neisseria meningitidis | B | ST-865 complex | No value | France | SV-5 | Opc+ |
| 40446 | LNP27584 | Neisseria meningitidis | B | ST-32 complex | No value | France | SV-1 | Opc+ |
| 40447 | LNP27589 | Neisseria meningitidis | B | ST-32 complex | No value | France | SV-1 | Opc+ |
| 40448 | LNP27598 | Neisseria meningitidis | B | ST-32 complex | No value | France | SV-1 | Opc+ |
| 40449 | LNP27652 | Neisseria meningitidis | B | ST-41/44 complex | No value | France | SV-10 | Opc+ |
| 40450 | LNP27654 | Neisseria meningitidis | B | ST-32 complex | No value | France | SV-1 | Opc+ |
| 40451 | LNP27656 | Neisseria meningitidis | B | ST-41/44 complex | No value | France | SV-6 | Opc+ |
| 40452 | LNP27661 | Neisseria meningitidis | B | No value | No value | France | SV-5 | Opc+ |
| 40453 | LNP27662 | Neisseria meningitidis | B | ST-32 complex | No value | France | SV-1 | Opc+ |
| 40454 | LNP27678 | Neisseria meningitidis | B | ST-41/44 complex | No value | France | SV-2 | Opc+ |
| 40455 | LNP27685 | Neisseria meningitidis | B | ST-32 complex | No value | France | SV-1 | Opc+ |
| 40456 | LNP27712 | Neisseria meningitidis | B | ST-32 complex | No value | France | SV-1 | Opc+ |
| 40457 | LNP27355 | Neisseria meningitidis | B | No value | No value | France | SV-1 | Opc+ |
| 40458 | LNP27694 | Neisseria meningitidis | B | No value | No value | France | SV-5 | Opc+ |
| 40459 | LNP27408 | Neisseria meningitidis | B | ST-461 complex | No value | France | SV-1 | Opc- |
| 40460 | LNP27367 | Neisseria meningitidis | B | No value | No value | France | SV-11 | Opc+ |
| 40461 | LNP27485 | Neisseria meningitidis | B | ST-41/44 complex | No value | France | SV-10 | Opc+ |
| 40462 | LNP27690 | Neisseria meningitidis | B | ST-41/44 complex | No value | France | SV-2 | Opc+ |
| 40463 | LNP27557 | Neisseria meningitidis | B | ST-11 complex | No value | France | SV-2 | Opc- |
| 40464 | LNP27415 | Neisseria meningitidis | B | No value | No value | France | SV-1 | Opc+ |
| 40465 | LNP27676 | Neisseria meningitidis | B | ST-32 complex | No value | France | SV-1 | Opc+ |
| 40466 | LNP27612 | Neisseria meningitidis | B | ST-213 complex | No value | France | SV-2 | Opc- |
| 40467 | LNP27629 | Neisseria meningitidis | B | ST-32 complex | No value | France | SV-1 | Opc+ |
| 40468 | LNP28262 | Neisseria meningitidis | B | ST-32 complex | No value | France | SV-1 | Opc+ |
| 40469 | LNP28264 | Neisseria meningitidis | B | ST-269 complex | No value | France | SV-8 | Opc+ |
| 40470 | LNP28266 | Neisseria meningitidis | NG | No value | No value | France | SV-7 | Opc+ |
| 40471 | LNP28267 | Neisseria meningitidis | W | ST-53 complex | No value | France | SV-2 | Opc- |
| 40472 | LNP28270 | Neisseria meningitidis | C | No value | No value | France | SV-2 | Opc- |
| 40473 | LNP28271 | Neisseria meningitidis | B | ST-269 complex | No value | France | SV-1 | Opc+ |
| 40474 | LNP28274 | Neisseria meningitidis | Y | ST-23 complex | No value | France | SV-7 | Opc+ |
| 40475 | LNP28276 | Neisseria meningitidis | C | ST-11 complex | No value | France | SV-2 | Opc- |
| 40476 | LNP28277 | Neisseria meningitidis | B | ST-41/44 complex | No value | France | SV-6 | Opc+ |
| 40477 | LNP28281 | Neisseria meningitidis | Y | ST-103 complex | No value | France | SV-5 | Opc+ |
| 40478 | LNP28282 | Neisseria meningitidis | B | ST-461 complex | No value | France | SV-1 | Opc- |
| 40479 | LNP28286 | Neisseria meningitidis | C | ST-11 complex | No value | France | SV-2 | Opc- |
| 40480 | LNP28287 | Neisseria meningitidis | B | ST-269 complex | No value | France | SV-1 | Opc+ |
| 40481 | LNP28288 | Neisseria meningitidis | B | ST-269 complex | No value | France | SV-1 | Opc+ |
| 40482 | LNP28289 | Neisseria meningitidis | C | ST-11 complex | No value | France | SV-2 | Opc- |
| 40483 | LNP28290 | Neisseria meningitidis | C | ST-213 complex | No value | France | SV-12 | Opc- |
| 40484 | LNP28291 | Neisseria meningitidis | B | ST-11 complex | No value | France | SV-2 | Opc- |
| 40485 | LNP28292 | Neisseria meningitidis | C | ST-11 complex | No value | France | SV-2 | Opc- |

| | | | | | | | | |
|---|---|---|---|---|---|---|---|---|
| 40486 | LNP28293 | Neisseria meningitidis | W | ST-22 complex | No value | France | SV-12 | Opc+ |
| 40487 | LNP28294 | Neisseria meningitidis | B | ST-41/44 complex | No value | France | SV-6 | Opc+ |
| 40488 | LNP28295 | Neisseria meningitidis | C | ST-11 complex | No value | France | SV-2 | Opc- |
| 40489 | LNP28296 | Neisseria meningitidis | B | ST-213 complex | No value | France | SV-12 | Opc- |
| 40490 | LNP28297 | Neisseria meningitidis | B | ST-269 complex | No value | France | SV-1 | Opc+ |
| 40491 | LNP28298 | Neisseria meningitidis | B | ST-11 complex | No value | France | SV-2 | Opc- |
| 40492 | LNP28284 | Neisseria meningitidis | C | ST-461 complex | No value | France | SV-1 | Opc- |
| 40493 | LNP28285 | Neisseria meningitidis | B | ST-32 complex | No value | France | SV-1 | Opc+ |
| 40494 | LNP27419 | Neisseria meningitidis | B | ST-41/44 complex | No value | France | SV-6 | Opc+ |
| 40495 | LNP27420 | Neisseria meningitidis | B | ST-41/44 complex | No value | France | SV-2 | Opc+ |
| 40500 | M17661 | Neisseria meningitidis | W | ST-11 complex | No value | USA | SV-2 | Opc- |
| 40501 | M18774 | Neisseria meningitidis | W | ST-11 complex | No value | USA | SV-2 | Opc- |
| 40502 | 34526 | Neisseria meningitidis | NG | No value | No value | South Africa | SV-1 | Opc- |
| 40503 | 116 | Neisseria meningitidis | C | ST-32 complex | Endemic | South Africa | SV-1 | Opc+ |
| 40504 | 475 | Neisseria meningitidis | C | ST-865 complex | Endemic | South Africa | SV-5 | Opc+ |
| 40505 | 493 | Neisseria meningitidis | C | ST-35 complex | Endemic | South Africa | SV-5 | Opc+ |
| 40506 | 586 | Neisseria meningitidis | C | ST-865 complex | Endemic | South Africa | SV-10 | Opc+ |
| 40507 | 625 | Neisseria meningitidis | C | ST-11 complex | Endemic | South Africa | SV-2 | Opc- |
| 40508 | 716 | Neisseria meningitidis | C | ST-865 complex | Endemic | South Africa | SV-5 | Opc+ |
| 40509 | 1394 | Neisseria meningitidis | C | ST-11 complex | Endemic | South Africa | SV-2 | Opc- |
| 40510 | 1397 | Neisseria meningitidis | C | ST-11 complex | Endemic | South Africa | SV-2 | Opc- |
| 40511 | 1428 | Neisseria meningitidis | C | ST-11 complex | Endemic | South Africa | SV-2 | Opc- |
| 40512 | 1429 | Neisseria meningitidis | C | ST-11 complex | Endemic | South Africa | SV-2 | Opc- |
| 40513 | 1942 | Neisseria meningitidis | C | ST-41/44 complex | Endemic | South Africa | SV-10 | Opc+ |
| 40514 | 2179 | Neisseria meningitidis | C | ST-865 complex | Endemic | South Africa | SV-5 | Opc+ |
| 40515 | 2251 | Neisseria meningitidis | C | ST-11 complex | Endemic | South Africa | SV-2 | Opc- |
| 40516 | 2281 | Neisseria meningitidis | C | ST-11 complex | Endemic | South Africa | SV-2 | Opc- |
| 40517 | 2458 | Neisseria meningitidis | C | ST-11 complex | Endemic | South Africa | SV-2 | Opc- |
| 40518 | 2459 | Neisseria meningitidis | C | ST-11 complex | Endemic | South Africa | SV-2 | Opc- |
| 40519 | 2781 | Neisseria meningitidis | C | ST-11 complex | Endemic | South Africa | SV-2 | Opc- |
| 40520 | 2885 | Neisseria meningitidis | C | No value | Endemic | South Africa | SV-2 | Opc- |
| 40521 | 3318 | Neisseria meningitidis | C | ST-11 complex | Endemic | South Africa | SV-2 | Opc- |
| 40522 | 3370 | Neisseria meningitidis | C | No value | Endemic | South Africa | SV-5 | Opc+ |
| 40523 | 3464 | Neisseria meningitidis | C | No value | Endemic | South Africa | SV-5 | Opc+ |
| 40524 | 4185 | Neisseria meningitidis | C | ST-865 complex | Endemic | South Africa | SV-5 | Opc+ |
| 40525 | 4641 | Neisseria meningitidis | C | ST-11 complex | Endemic | South Africa | SV-2 | Opc- |
| 40526 | 5212 | Neisseria meningitidis | C | ST-865 complex | Endemic | South Africa | SV-5 | Opc+ |
| 40527 | 5218 | Neisseria meningitidis | C | ST-11 complex | Endemic | South Africa | SV-2 | Opc- |
| 40528 | 5439 | Neisseria meningitidis | C | ST-32 complex | Endemic | South Africa | SV-1 | Opc+ |
| 40529 | 5548 | Neisseria meningitidis | C | ST-865 complex | Endemic | South Africa | SV-5 | Opc+ |
| 40530 | 5556 | Neisseria meningitidis | C | ST-41/44 complex | Endemic | South Africa | SV-10 | Opc+ |
| 40531 | 5803 | Neisseria meningitidis | C | ST-865 complex | Endemic | South Africa | SV-5 | Opc+ |
| 40532 | 6112 | Neisseria meningitidis | C | ST-11 complex | Endemic | South Africa | SV-2 | Opc- |
| 40533 | 6122 | Neisseria meningitidis | C | ST-865 complex | Endemic | South Africa | SV-5 | Opc+ |
| 40534 | 6183 | Neisseria meningitidis | C | No value | Endemic | South Africa | SV-7 | Opc- |
| 40535 | 6592 | Neisseria meningitidis | C | ST-11 complex | Endemic | South Africa | SV-2 | Opc- |
| 40536 | 6804 | Neisseria meningitidis | C | ST-11 complex | Endemic | South Africa | SV-2 | Opc- |
| 40537 | 6993 | Neisseria meningitidis | C | ST-35 complex | Endemic | South Africa | SV-12 | Opc+ |
| 40538 | 7794 | Neisseria meningitidis | C | ST-11 complex | Endemic | South Africa | SV-2 | Opc- |
| 40539 | 7826 | Neisseria meningitidis | C | No value | Endemic | South Africa | SV-7 | Opc- |
| 40540 | 7861 | Neisseria meningitidis | C | ST-11 complex | Endemic | South Africa | SV-2 | Opc- |
| 40541 | 8044 | Neisseria meningitidis | C | No value | Endemic | South Africa | SV-10 | Opc+ |
| 40542 | 8852 | Neisseria meningitidis | C | ST-41/44 complex | Endemic | South Africa | SV-6 | Opc+ |
| 40543 | 8956 | Neisseria meningitidis | C | ST-35 complex | Endemic | South Africa | SV-12 | Opc+ |
| 40544 | 9037 | Neisseria meningitidis | C | No value | Endemic | South Africa | SV-7 | Opc+ |
| 40545 | 9102 | Neisseria meningitidis | C | ST-11 complex | Endemic | South Africa | SV-2 | Opc- |
| 40546 | 9104 | Neisseria meningitidis | C | ST-865 complex | Endemic | South Africa | SV-5 | Opc+ |
| 40547 | 689 | Neisseria meningitidis | C | ST-11 complex | Endemic | South Africa | SV-2 | Opc- |
| 40548 | 1339 | Neisseria meningitidis | C | ST-865 complex | Endemic | South Africa | SV-5 | Opc+ |
| 40549 | 1628 | Neisseria meningitidis | C | No value | Endemic | South Africa | SV-5 | Opc+ |
| 40550 | 2105 | Neisseria meningitidis | C | ST-865 complex | Endemic | South Africa | SV-5 | Opc+ |
| 40551 | 2241 | Neisseria meningitidis | C | ST-11 complex | Endemic | South Africa | SV-2 | Opc- |
| 40552 | 2581 | Neisseria meningitidis | C | No value | Endemic | South Africa | SV-2 | Opc- |
| 40553 | 2666 | Neisseria meningitidis | C | ST-11 complex | Endemic | South Africa | SV-2 | Opc- |
| 40554 | 3027 | Neisseria meningitidis | C | No value | Endemic | South Africa | SV-5 | Opc+ |
| 40555 | 3198 | Neisseria meningitidis | C | ST-865 complex | Endemic | South Africa | SV-5 | Opc+ |
| 40556 | 4004 | Neisseria meningitidis | C | ST-865 complex | Endemic | South Africa | SV-5 | Opc+ |
| 40557 | 4005 | Neisseria meningitidis | C | ST-865 complex | Endemic | South Africa | SV-5 | Opc+ |
| 40558 | 5167 | Neisseria meningitidis | C | ST-865 complex | Endemic | South Africa | SV-5 | Opc+ |
| 40559 | 5180 | Neisseria meningitidis | C | ST-11 complex | Endemic | South Africa | SV-2 | Opc- |
| 40560 | 5593 | Neisseria meningitidis | C | ST-865 complex | Endemic | South Africa | SV-5 | Opc+ |
| 40561 | 5824 | Neisseria meningitidis | C | ST-865 complex | Endemic | South Africa | SV-5 | Opc+ |
| 40562 | 6367 | Neisseria meningitidis | C | No value | Endemic | South Africa | SV-5 | Opc+ |
| 40563 | 6368 | Neisseria meningitidis | C | No value | Endemic | South Africa | SV-5 | Opc+ |

| | | | | | | | | |
|---|---|---|---|---|---|---|---|---|
| 40564 | 6532 | Neisseria meningitidis | C | ST-865 complex | Endemic | South Africa | SV-5 | Opc+ |
| 40565 | 6966 | Neisseria meningitidis | C | ST-865 complex | Endemic | South Africa | SV-5 | Opc+ |
| 40566 | 7305 | Neisseria meningitidis | C | No value | Endemic | South Africa | SV-5 | Opc- |
| 40567 | 7328 | Neisseria meningitidis | C | ST-41/44 complex | Endemic | South Africa | SV-6 | Opc+ |
| 40568 | 7949 | Neisseria meningitidis | C | ST-865 complex | Endemic | South Africa | SV-5 | Opc+ |
| 40569 | 8046 | Neisseria meningitidis | C | ST-865 complex | Endemic | South Africa | SV-5 | Opc+ |
| 40570 | 8052 | Neisseria meningitidis | C | ST-865 complex | Endemic | South Africa | SV-5 | Opc+ |
| 40571 | 8126 | Neisseria meningitidis | C | ST-865 complex | Endemic | South Africa | SV-5 | Opc+ |
| 40572 | 8253 | Neisseria meningitidis | C | No value | Endemic | South Africa | SV-7 | Opc+ |
| 40573 | 8291 | Neisseria meningitidis | C | ST-212 complex | Endemic | South Africa | SV-11 | Opc+ |
| 40574 | 8422 | Neisseria meningitidis | C | ST-11 complex | Endemic | South Africa | SV-2 | Opc- |
| 40575 | 8602 | Neisseria meningitidis | C | ST-11 complex | Endemic | South Africa | SV-2 | Opc- |
| 40576 | 8903 | Neisseria meningitidis | C | ST-865 complex | Endemic | South Africa | SV-5 | Opc+ |
| 40577 | 9079 | Neisseria meningitidis | C | ST-865 complex | Endemic | South Africa | SV-5 | Opc+ |
| 40578 | 9129 | Neisseria meningitidis | C | ST-865 complex | Endemic | South Africa | SV-5 | Opc+ |
| 40579 | 9134 | Neisseria meningitidis | C | ST-11 complex | Endemic | South Africa | SV-2 | Opc- |
| 40580 | 9153 | Neisseria meningitidis | C | ST-11 complex | Endemic | South Africa | SV-2 | Opc- |
| 40581 | 9222 | Neisseria meningitidis | C | ST-865 complex | Endemic | South Africa | SV-5 | Opc+ |
| 40582 | 9224 | Neisseria meningitidis | C | ST-865 complex | Endemic | South Africa | SV-5 | Opc+ |
| 40583 | 9281 | Neisseria meningitidis | C | ST-175 complex | Endemic | South Africa | SV-2 | Opc+ |
| 40584 | 9282 | Neisseria meningitidis | C | ST-865 complex | Endemic | South Africa | SV-5 | Opc+ |
| 40585 | 9372 | Neisseria meningitidis | C | ST-231 complex | Endemic | South Africa | SV-7 | Opc- |
| 40586 | 9433 | Neisseria meningitidis | C | ST-11 complex | Endemic | South Africa | SV-2 | Opc- |
| 40587 | 9615 | Neisseria meningitidis | C | No value | Endemic | South Africa | SV-5 | Opc+ |
| 40588 | 9634 | Neisseria meningitidis | C | ST-175 complex | Endemic | South Africa | SV-5 | Opc+ |
| 40589 | 10073 | Neisseria meningitidis | C | ST-865 complex | Endemic | South Africa | SV-5 | Opc+ |
| 40590 | 10162 | Neisseria meningitidis | C | ST-865 complex | Endemic | South Africa | SV-5 | Opc+ |
| 40591 | 10179 | Neisseria meningitidis | C | ST-11 complex | Endemic | South Africa | SV-2 | Opc- |
| 40592 | 10267 | Neisseria meningitidis | C | ST-865 complex | Endemic | South Africa | SV-5 | Opc+ |
| 40593 | 10369 | Neisseria meningitidis | C | ST-175 complex | Endemic | South Africa | SV-5 | Opc+ |
| 40594 | 10379 | Neisseria meningitidis | C | ST-269 complex | Endemic | South Africa | SV-1 | Opc+ |
| 40595 | 10428 | Neisseria meningitidis | C | ST-11 complex | Endemic | South Africa | SV-2 | Opc- |
| 40596 | 10445 | Neisseria meningitidis | C | ST-865 complex | Endemic | South Africa | SV-5 | Opc+ |
| 40597 | 10541 | Neisseria meningitidis | C | ST-1 complex | Endemic | South Africa | SV-5 | Opc+ |
| 40598 | 10762 | Neisseria meningitidis | C | ST-32 complex | Endemic | South Africa | SV-1 | Opc+ |
| 40599 | 10773 | Neisseria meningitidis | C | ST-212 complex | Endemic | South Africa | SV-11 | Opc+ |
| 40600 | 10796 | Neisseria meningitidis | C | ST-11 complex | Endemic | South Africa | SV-2 | Opc- |
| 40601 | 10797 | Neisseria meningitidis | C | ST-11 complex | Endemic | South Africa | SV-2 | Opc- |
| 40602 | 10840 | Neisseria meningitidis | C | ST-11 complex | Endemic | South Africa | SV-2 | Opc- |
| 40603 | 11052 | Neisseria meningitidis | C | ST-865 complex | Endemic | South Africa | SV-5 | Opc+ |
| 40604 | 11069 | Neisseria meningitidis | C | ST-32 complex | Endemic | South Africa | SV-1 | Opc+ |
| 40605 | 11072 | Neisseria meningitidis | C | ST-11 complex | Endemic | South Africa | SV-2 | Opc- |
| 40606 | 11146 | Neisseria meningitidis | C | ST-11 complex | Endemic | South Africa | SV-2 | Opc- |
| 40607 | 11483 | Neisseria meningitidis | C | ST-1 complex | Endemic | South Africa | SV-5 | Opc+ |
| 40608 | 11683 | Neisseria meningitidis | C | ST-11 complex | Endemic | South Africa | SV-2 | Opc- |
| 40609 | 12111 | Neisseria meningitidis | C | ST-41/44 complex | Endemic | South Africa | SV-10 | Opc+ |
| 40610 | 12155 | Neisseria meningitidis | C | ST-11 complex | Endemic | South Africa | SV-2 | Opc- |
| 40611 | 12336 | Neisseria meningitidis | C | ST-865 complex | Endemic | South Africa | SV-5 | Opc+ |
| 40612 | 12655 | Neisseria meningitidis | C | ST-41/44 complex | Endemic | South Africa | SV-10 | Opc+ |
| 40613 | 12781 | Neisseria meningitidis | C | ST-865 complex | Endemic | South Africa | SV-5 | Opc+ |
| 40614 | 12782 | Neisseria meningitidis | C | ST-865 complex | Endemic | South Africa | SV-5 | Opc+ |
| 40615 | 12783 | Neisseria meningitidis | C | ST-11 complex | Endemic | South Africa | SV-2 | Opc- |
| 40616 | 12805 | Neisseria meningitidis | C | ST-865 complex | Endemic | South Africa | SV-5 | Opc+ |
| 40617 | 12891 | Neisseria meningitidis | C | ST-865 complex | Endemic | South Africa | SV-5 | Opc+ |
| 40618 | 12893 | Neisseria meningitidis | C | ST-212 complex | Endemic | South Africa | SV-11 | Opc+ |
| 40619 | 13216 | Neisseria meningitidis | C | ST-865 complex | Endemic | South Africa | SV-5 | Opc+ |
| 40620 | 13453 | Neisseria meningitidis | C | ST-41/44 complex | Endemic | South Africa | SV-10 | Opc+ |
| 40621 | 13503 | Neisseria meningitidis | C | ST-865 complex | Endemic | South Africa | SV-5 | Opc+ |
| 40622 | 13619 | Neisseria meningitidis | C | ST-865 complex | Endemic | South Africa | SV-5 | Opc+ |
| 40623 | 13634 | Neisseria meningitidis | C | ST-865 complex | Endemic | South Africa | SV-5 | Opc+ |
| 40624 | 13774 | Neisseria meningitidis | C | ST-11 complex | Endemic | South Africa | SV-2 | Opc- |
| 40625 | 13845 | Neisseria meningitidis | C | ST-865 complex | Endemic | South Africa | SV-5 | Opc+ |
| 40626 | 13861 | Neisseria meningitidis | C | ST-865 complex | Endemic | South Africa | SV-5 | Opc+ |
| 40627 | 13945 | Neisseria meningitidis | C | No value | Endemic | South Africa | SV-1 | Opc+ |
| 40628 | 14221 | Neisseria meningitidis | C | ST-865 complex | Endemic | South Africa | SV-5 | Opc+ |
| 40629 | 14229 | Neisseria meningitidis | C | ST-865 complex | Endemic | South Africa | SV-5 | Opc+ |
| 40630 | 14541 | Neisseria meningitidis | C | ST-865 complex | Endemic | South Africa | SV-5 | Opc+ |
| 40631 | 14683 | Neisseria meningitidis | C | ST-231 complex | Endemic | South Africa | SV-7 | Opc- |
| 40632 | 14684 | Neisseria meningitidis | C | ST-865 complex | Endemic | South Africa | SV-5 | Opc+ |
| 40633 | 14811 | Neisseria meningitidis | C | ST-865 complex | Endemic | South Africa | SV-5 | Opc+ |
| 40634 | 14821 | Neisseria meningitidis | C | ST-865 complex | Endemic | South Africa | SV-5 | Opc+ |
| 40635 | 15011 | Neisseria meningitidis | C | ST-865 complex | Endemic | South Africa | SV-5 | Opc+ |
| 40636 | 15099 | Neisseria meningitidis | C | ST-865 complex | Endemic | South Africa | SV-5 | Opc+ |
| 40637 | 15121 | Neisseria meningitidis | C | ST-865 complex | Endemic | South Africa | SV-5 | Opc+ |

| | | | | | | | | |
|---|---|---|---|---|---|---|---|---|
| 40638 | 15172 | Neisseria meningitidis | C | ST-865 complex | Endemic | South Africa | SV-5 | Opc+ |
| 40639 | 15420 | Neisseria meningitidis | C | ST-865 complex | Endemic | South Africa | SV-5 | Opc+ |
| 40640 | 15477 | Neisseria meningitidis | C | ST-41/44 complex | Endemic | South Africa | SV-10 | Opc+ |
| 40641 | 15600 | Neisseria meningitidis | C | ST-11 complex | Endemic | South Africa | SV-2 | Opc- |
| 40642 | 15643 | Neisseria meningitidis | C | ST-865 complex | Endemic | South Africa | SV-5 | Opc+ |
| 40643 | 15657 | Neisseria meningitidis | C | ST-11 complex | Endemic | South Africa | SV-2 | Opc- |
| 40644 | 15734 | Neisseria meningitidis | C | ST-175 complex | Endemic | South Africa | SV-5 | Opc+ |
| 40645 | 15847 | Neisseria meningitidis | C | ST-865 complex | Endemic | South Africa | SV-5 | Opc+ |
| 40646 | 15864 | Neisseria meningitidis | C | ST-35 complex | Endemic | South Africa | SV-17 | Opc+ |
| 40647 | 15921 | Neisseria meningitidis | C | ST-11 complex | Endemic | South Africa | SV-2 | Opc- |
| 40648 | 15956 | Neisseria meningitidis | C | ST-865 complex | Endemic | South Africa | SV-5 | Opc+ |
| 40649 | 16045 | Neisseria meningitidis | C | ST-865 complex | Endemic | South Africa | SV-5 | Opc+ |
| 40650 | 16132 | Neisseria meningitidis | C | ST-865 complex | Endemic | South Africa | SV-5 | Opc+ |
| 40651 | 16439 | Neisseria meningitidis | C | ST-865 complex | Endemic | South Africa | SV-5 | Opc+ |
| 40652 | 16612 | Neisseria meningitidis | C | ST-865 complex | Endemic | South Africa | SV-5 | Opc+ |
| 40653 | 16649 | Neisseria meningitidis | C | ST-865 complex | Endemic | South Africa | SV-5 | Opc+ |
| 40654 | 16675 | Neisseria meningitidis | C | ST-11 complex | Endemic | South Africa | SV-2 | Opc- |
| 40655 | 16701 | Neisseria meningitidis | C | ST-865 complex | Endemic | South Africa | SV-5 | Opc+ |
| 40656 | 16733 | Neisseria meningitidis | C | ST-11 complex | Endemic | South Africa | SV-2 | Opc- |
| 40657 | 16775 | Neisseria meningitidis | C | ST-11 complex | Endemic | South Africa | SV-2 | Opc- |
| 40658 | 16934 | Neisseria meningitidis | C | ST-11 complex | Endemic | South Africa | SV-2 | Opc- |
| 40659 | 17017 | Neisseria meningitidis | C | ST-865 complex | Endemic | South Africa | SV-5 | Opc+ |
| 40660 | 17100 | Neisseria meningitidis | C | No value | Endemic | South Africa | SV-11 | Opc+ |
| 40661 | 17136 | Neisseria meningitidis | C | No value | Endemic | South Africa | SV-1 | Opc+ |
| 40662 | 17585 | Neisseria meningitidis | C | ST-212 complex | Endemic | South Africa | SV-11 | Opc+ |
| 40663 | 17612 | Neisseria meningitidis | C | ST-11 complex | Endemic | South Africa | SV-2 | Opc- |
| 40664 | 17614 | Neisseria meningitidis | C | ST-865 complex | Endemic | South Africa | SV-5 | Opc+ |
| 40665 | 17642 | Neisseria meningitidis | C | ST-11 complex | Endemic | South Africa | SV-2 | Opc- |
| 40666 | 18159 | Neisseria meningitidis | C | ST-32 complex | Endemic | South Africa | SV-1 | Opc+ |
| 40667 | 18162 | Neisseria meningitidis | C | ST-11 complex | Endemic | South Africa | SV-2 | Opc- |
| 40668 | 18180 | Neisseria meningitidis | C | ST-11 complex | Endemic | South Africa | SV-2 | Opc- |
| 40669 | 18207 | Neisseria meningitidis | C | ST-32 complex | Endemic | South Africa | SV-1 | Opc+ |
| 40670 | 18273 | Neisseria meningitidis | C | ST-865 complex | Endemic | South Africa | SV-5 | Opc+ |
| 40671 | 18561 | Neisseria meningitidis | C | ST-865 complex | Endemic | South Africa | SV-5 | Opc+ |
| 40672 | 18692 | Neisseria meningitidis | C | ST-865 complex | Endemic | South Africa | SV-5 | Opc+ |
| 40673 | 18872 | Neisseria meningitidis | C | ST-865 complex | Endemic | South Africa | SV-5 | Opc+ |
| 40674 | 18962 | Neisseria meningitidis | C | ST-60 complex | Endemic | South Africa | SV-11 | Opc+ |
| 40675 | 19127 | Neisseria meningitidis | C | ST-865 complex | Endemic | South Africa | SV-5 | Opc+ |
| 40676 | 20560 | Neisseria meningitidis | C | ST-865 complex | Endemic | South Africa | SV-5 | Opc+ |
| 40677 | 20562 | Neisseria meningitidis | C | ST-865 complex | Endemic | South Africa | SV-5 | Opc+ |
| 40678 | 20764 | Neisseria meningitidis | C | ST-32 complex | Endemic | South Africa | SV-1 | Opc+ |
| 40679 | 20784 | Neisseria meningitidis | C | ST-865 complex | Endemic | South Africa | SV-5 | Opc+ |
| 40680 | 20807 | Neisseria meningitidis | C | ST-60 complex | Endemic | South Africa | SV-11 | Opc+ |
| 40681 | 20967 | Neisseria meningitidis | C | ST-212 complex | Endemic | South Africa | SV-11 | Opc+ |
| 40682 | 21024 | Neisseria meningitidis | C | ST-865 complex | Endemic | South Africa | SV-5 | Opc+ |
| 40683 | 21030 | Neisseria meningitidis | C | ST-865 complex | Endemic | South Africa | SV-5 | Opc+ |
| 40684 | 21048 | Neisseria meningitidis | C | ST-865 complex | Endemic | South Africa | SV-5 | Opc+ |
| 40685 | 21116 | Neisseria meningitidis | C | ST-11 complex | Endemic | South Africa | SV-2 | Opc- |
| 40686 | 21141 | Neisseria meningitidis | C | ST-865 complex | Endemic | South Africa | SV-5 | Opc+ |
| 40687 | 21763 | Neisseria meningitidis | C | ST-32 complex | Endemic | South Africa | SV-1 | Opc+ |
| 40688 | 21793 | Neisseria meningitidis | C | ST-35 complex | Endemic | South Africa | SV-14 | Opc+ |
| 40689 | 22189 | Neisseria meningitidis | C | ST-11 complex | Endemic | South Africa | SV-2 | Opc- |
| 40690 | 22335 | Neisseria meningitidis | C | ST-865 complex | Endemic | South Africa | SV-5 | Opc+ |
| 40691 | 22952 | Neisseria meningitidis | C | ST-865 complex | Endemic | South Africa | SV-5 | Opc+ |
| 40692 | 22971 | Neisseria meningitidis | C | ST-865 complex | Endemic | South Africa | SV-5 | Opc+ |
| 40693 | 23069 | Neisseria meningitidis | C | ST-865 complex | Endemic | South Africa | SV-5 | Opc+ |
| 40694 | 23429 | Neisseria meningitidis | C | ST-865 complex | Endemic | South Africa | SV-5 | Opc+ |
| 40695 | 23443 | Neisseria meningitidis | C | ST-103 complex | Endemic | South Africa | SV-5 | Opc+ |
| 40696 | 23612 | Neisseria meningitidis | C | ST-231 complex | Endemic | South Africa | SV-7 | Opc- |
| 40697 | 24049 | Neisseria meningitidis | C | ST-11 complex | Endemic | South Africa | SV-2 | Opc- |
| 40698 | 24236 | Neisseria meningitidis | C | ST-11 complex | Endemic | South Africa | SV-2 | Opc- |
| 40699 | 24482 | Neisseria meningitidis | C | No value | Endemic | South Africa | SV-1 | Opc+ |
| 40700 | 24662 | Neisseria meningitidis | C | ST-865 complex | Endemic | South Africa | SV-5 | Opc+ |
| 40701 | 24834 | Neisseria meningitidis | C | ST-865 complex | Endemic | South Africa | SV-5 | Opc+ |
| 40702 | 24898 | Neisseria meningitidis | C | ST-865 complex | Endemic | South Africa | SV-5 | Opc+ |
| 40703 | 26244 | Neisseria meningitidis | C | ST-11 complex | Endemic | South Africa | SV-2 | Opc- |
| 40704 | 26838 | Neisseria meningitidis | C | ST-865 complex | Endemic | South Africa | SV-5 | Opc+ |
| 40705 | 27437 | Neisseria meningitidis | C | ST-865 complex | Endemic | South Africa | SV-5 | Opc+ |
| 40706 | 27559 | Neisseria meningitidis | C | ST-11 complex | Endemic | South Africa | SV-2 | Opc- |
| 40707 | 27586 | Neisseria meningitidis | C | ST-865 complex | Endemic | South Africa | SV-5 | Opc+ |
| 40708 | 28613 | Neisseria meningitidis | C | No value | Endemic | South Africa | SV-5 | Opc+ |
| 40709 | 29100 | Neisseria meningitidis | C | ST-11 complex | Endemic | South Africa | SV-2 | Opc- |
| 40710 | 29989 | Neisseria meningitidis | C | ST-865 complex | Endemic | South Africa | SV-5 | Opc+ |
| 40711 | 30079 | Neisseria meningitidis | C | ST-865 complex | Endemic | South Africa | SV-5 | Opc+ |

| | | | | | | | | |
|---|---|---|---|---|---|---|---|---|
| 40712 | 30346 | Neisseria meningitidis | C | ST-32 complex | Endemic | South Africa | SV-1 | Opc+ |
| 40713 | 30407 | Neisseria meningitidis | C | ST-865 complex | Endemic | South Africa | SV-5 | Opc+ |
| 40714 | 30408 | Neisseria meningitidis | C | ST-865 complex | Endemic | South Africa | SV-5 | Opc+ |
| 40715 | 30517 | Neisseria meningitidis | C | No value | Endemic | South Africa | SV-5 | Opc+ |
| 40716 | 31345 | Neisseria meningitidis | C | ST-11 complex | Endemic | South Africa | SV-2 | Opc- |
| 40717 | 31442 | Neisseria meningitidis | C | ST-103 complex | Endemic | South Africa | SV-5 | Opc+ |
| 40718 | 32200 | Neisseria meningitidis | C | ST-865 complex | Endemic | South Africa | SV-5 | Opc+ |
| 40719 | 32498 | Neisseria meningitidis | C | ST-865 complex | Endemic | South Africa | SV-5 | Opc+ |
| 40720 | 32551 | Neisseria meningitidis | C | ST-865 complex | Endemic | South Africa | SV-5 | Opc+ |
| 40721 | 32661 | Neisseria meningitidis | C | No value | Endemic | South Africa | SV-5 | Opc+ |
| 40722 | 32733 | Neisseria meningitidis | C | ST-865 complex | Endemic | South Africa | SV-5 | Opc+ |
| 40723 | 32998 | Neisseria meningitidis | C | ST-865 complex | Endemic | South Africa | SV-5 | Opc+ |
| 40724 | 33690 | Neisseria meningitidis | C | ST-103 complex | Endemic | South Africa | SV-5 | Opc+ |
| 40725 | 33792 | Neisseria meningitidis | C | ST-865 complex | Endemic | South Africa | SV-5 | Opc+ |
| 40726 | 34146 | Neisseria meningitidis | C | ST-32 complex | Endemic | South Africa | SV-1 | Opc+ |
| 40727 | 34205 | Neisseria meningitidis | C | ST-865 complex | Endemic | South Africa | SV-5 | Opc+ |
| 40728 | 34249 | Neisseria meningitidis | C | ST-865 complex | Endemic | South Africa | SV-5 | Opc+ |
| 40729 | 34392 | Neisseria meningitidis | C | ST-865 complex | Endemic | South Africa | SV-5 | Opc+ |
| 40730 | 34393 | Neisseria meningitidis | C | ST-32 complex | Endemic | South Africa | SV-1 | Opc+ |
| 40731 | 34528 | Neisseria meningitidis | C | No value | Endemic | South Africa | SV-7 | Opc+ |
| 40732 | 34541 | Neisseria meningitidis | C | ST-865 complex | Endemic | South Africa | SV-5 | Opc+ |
| 40733 | 34805 | Neisseria meningitidis | C | ST-865 complex | Endemic | South Africa | SV-5 | Opc+ |
| 40734 | 34811 | Neisseria meningitidis | C | ST-865 complex | Endemic | South Africa | SV-5 | Opc+ |
| 40735 | 34826 | Neisseria meningitidis | C | No value | Endemic | South Africa | SV-5 | Opc+ |
| 40736 | 34911 | Neisseria meningitidis | C | ST-212 complex | Endemic | South Africa | SV-11 | Opc+ |
| 40737 | 35572 | Neisseria meningitidis | C | ST-865 complex | Endemic | South Africa | SV-5 | Opc+ |
| 40738 | 36201 | Neisseria meningitidis | C | ST-11 complex | Endemic | South Africa | SV-2 | Opc- |
| 40739 | 36509 | Neisseria meningitidis | C | ST-865 complex | Endemic | South Africa | SV-5 | Opc+ |
| 40740 | 36917 | Neisseria meningitidis | C | ST-32 complex | Endemic | South Africa | SV-1 | Opc+ |
| 40741 | 36960 | Neisseria meningitidis | C | ST-865 complex | Endemic | South Africa | SV-5 | Opc+ |
| 40742 | 37236 | Neisseria meningitidis | C | ST-212 complex | Endemic | South Africa | SV-11 | Opc+ |
| 40743 | 37244 | Neisseria meningitidis | C | ST-865 complex | Endemic | South Africa | SV-5 | Opc+ |
| 40744 | 37724 | Neisseria meningitidis | C | ST-11 complex | Endemic | South Africa | SV-2 | Opc- |
| 40745 | 37782 | Neisseria meningitidis | C | ST-865 complex | Endemic | South Africa | SV-5 | Opc+ |
| 40746 | 38277 | Neisseria meningitidis | C | ST-865 complex | Endemic | South Africa | SV-5 | Opc+ |
| 40747 | 38412 | Neisseria meningitidis | C | No value | Endemic | South Africa | SV-5 | Opc+ |
| 40748 | 38801 | Neisseria meningitidis | C | ST-865 complex | Endemic | South Africa | SV-5 | Opc+ |
| 40749 | 39857 | Neisseria meningitidis | C | ST-865 complex | Endemic | South Africa | SV-5 | Opc+ |
| 40750 | 40321 | Neisseria meningitidis | C | ST-865 complex | Endemic | South Africa | SV-5 | Opc+ |
| 40751 | 40356 | Neisseria meningitidis | C | No value | Endemic | South Africa | SV-5 | Opc+ |
| 40752 | 40495 | Neisseria meningitidis | C | ST-213 complex | Endemic | South Africa | SV-12 | Opc- |
| 40753 | 41367 | Neisseria meningitidis | C | No value | Endemic | South Africa | SV-5 | Opc+ |
| 40754 | 41423 | Neisseria meningitidis | C | ST-865 complex | Endemic | South Africa | SV-5 | Opc+ |
| 40755 | 283 | Neisseria meningitidis | C | ST-865 complex | Endemic | South Africa | SV-5 | Opc+ |
| 40756 | 1173 | Neisseria meningitidis | C | ST-865 complex | Endemic | South Africa | SV-5 | Opc+ |
| 40757 | 3235 | Neisseria meningitidis | C | ST-865 complex | Endemic | South Africa | SV-5 | Opc+ |
| 40758 | 3769 | Neisseria meningitidis | C | ST-11 complex | Endemic | South Africa | SV-2 | Opc- |
| 40759 | 3910 | Neisseria meningitidis | C | ST-865 complex | Endemic | South Africa | SV-5 | Opc+ |
| 40760 | 6427 | Neisseria meningitidis | C | ST-865 complex | Endemic | South Africa | SV-5 | Opc+ |
| 40761 | 7583 | Neisseria meningitidis | C | No value | Endemic | South Africa | SV-1 | Opc- |
| 40762 | 8865 | Neisseria meningitidis | C | ST-11 complex | Endemic | South Africa | SV-2 | Opc- |
| 40763 | 15974 | Neisseria meningitidis | C | ST-865 complex | Endemic | South Africa | SV-5 | Opc+ |
| 40764 | 16256 | Neisseria meningitidis | C | ST-865 complex | Endemic | South Africa | SV-5 | Opc+ |
| 40765 | 16981 | Neisseria meningitidis | C | No value | Endemic | South Africa | SV-5 | Opc+ |
| 40766 | 17417 | Neisseria meningitidis | C | ST-11 complex | Endemic | South Africa | SV-2 | Opc- |
| 40767 | 17740 | Neisseria meningitidis | C | No value | Endemic | South Africa | SV-11 | Opc+ |
| 40768 | 19934 | Neisseria meningitidis | C | ST-865 complex | Endemic | South Africa | SV-5 | Opc+ |
| 40769 | 20308 | Neisseria meningitidis | C | ST-865 complex | Endemic | South Africa | SV-5 | Opc+ |
| 40770 | 21563 | Neisseria meningitidis | C | ST-865 complex | Endemic | South Africa | SV-5 | Opc+ |
| 40771 | 21712 | Neisseria meningitidis | C | ST-212 complex | Endemic | South Africa | SV-11 | Opc+ |
| 40772 | 26246 | Neisseria meningitidis | C | ST-865 complex | Endemic | South Africa | SV-5 | Opc+ |
| 40773 | 26843 | Neisseria meningitidis | C | ST-865 complex | Endemic | South Africa | SV-5 | Opc+ |
| 40774 | 27785 | Neisseria meningitidis | C | No value | Endemic | South Africa | SV-5 | Opc+ |
| 40775 | 27913 | Neisseria meningitidis | C | ST-32 complex | Endemic | South Africa | SV-1 | Opc+ |
| 40776 | 30924 | Neisseria meningitidis | C | No value | Endemic | South Africa | SV-1 | Opc- |
| 40777 | 32632 | Neisseria meningitidis | C | ST-865 complex | Endemic | South Africa | SV-5 | Opc+ |
| 40778 | 33177 | Neisseria meningitidis | C | No value | Endemic | South Africa | SV-14 | Opc+ |
| 40779 | 34027 | Neisseria meningitidis | C | No value | Endemic | South Africa | SV-1 | Opc+ |
| 40780 | 36148 | Neisseria meningitidis | C | ST-865 complex | Endemic | South Africa | SV-5 | Opc+ |
| 40781 | 36232 | Neisseria meningitidis | C | ST-865 complex | Endemic | South Africa | SV-5 | Opc+ |
| 40782 | 36525 | Neisseria meningitidis | C | ST-865 complex | Endemic | South Africa | SV-5 | Opc+ |
| 40783 | 41483 | Neisseria meningitidis | C | ST-865 complex | Endemic | South Africa | SV-5 | Opc+ |
| 40784 | 41661 | Neisseria meningitidis | C | ST-865 complex | Endemic | South Africa | SV-1 | Opc+ |
| 40785 | 42019 | Neisseria meningitidis | C | ST-865 complex | Endemic | South Africa | SV-5 | Opc+ |

| | | | | | | | | |
|---|---|---|---|---|---|---|---|---|
| 40786 | 42258 | Neisseria meningitidis | C | ST-865 complex | Endemic | South Africa | SV-5 | Opc+ |
| 40787 | 42366 | Neisseria meningitidis | C | ST-865 complex | Endemic | South Africa | SV-5 | Opc+ |
| 40788 | 43124 | Neisseria meningitidis | C | ST-865 complex | Endemic | South Africa | SV-5 | Opc+ |
| 40789 | 43574 | Neisseria meningitidis | C | No value | Endemic | South Africa | SV-5 | Opc+ |
| 40790 | 43783 | Neisseria meningitidis | C | ST-103 complex | Endemic | South Africa | SV-5 | Opc+ |
| 40791 | 44026 | Neisseria meningitidis | C | ST-32 complex | Endemic | South Africa | SV-1 | Opc+ |
| 41187 | Z0605086 | Neisseria meningitidis | W | ST-11 complex | No value | Central African Republic | SV-2 | Opc- |
| 41188 | Z0512115 | Neisseria meningitidis | W | ST-11 complex | No value | Central African Republic | SV-2 | Opc- |
| 41189 | Z0612118 | Neisseria meningitidis | W | ST-11 complex | No value | Central African Republic | SV-2 | Opc- |
| 41190 | Z0710082 | Neisseria meningitidis | W | ST-11 complex | No value | Central African Republic | SV-2 | Opc- |
| 41211 | LNP28387 | Neisseria meningitidis | B | ST-32 complex | No value | France | SV-1 | Opc+ |
| 41212 | LNP28388 | Neisseria meningitidis | W | ST-11 complex | No value | France | SV-2 | Opc- |
| 41213 | LNP28389 | Neisseria meningitidis | Y | ST-23 complex | No value | France | SV-11 | Opc+ |
| 41214 | LNP28390 | Neisseria meningitidis | W | No value | No value | France | SV-2 | Opc- |
| 41215 | LNP28391 | Neisseria meningitidis | Y | ST-23 complex | No value | France | SV-11 | Opc+ |
| 41216 | LNP28392 | Neisseria meningitidis | Y | ST-23 complex | No value | France | SV-11 | Opc+ |
| 41217 | LNP28393 | Neisseria meningitidis | E | ST-60 complex | No value | France | SV-11 | Opc+ |
| 41218 | LNP28394 | Neisseria meningitidis | B | ST-35 complex | No value | France | SV-12 | Opc+ |
| 41219 | LNP28395 | Neisseria meningitidis | B | ST-35 complex | No value | France | SV-12 | Opc+ |
| 41221 | LNP28397 | Neisseria meningitidis | B | ST-461 complex | No value | France | SV-1 | Opc- |
| 41222 | LNP28398 | Neisseria meningitidis | C | ST-461 complex | No value | France | SV-1 | Opc- |
| 41223 | LNP28399 | Neisseria meningitidis | B | ST-162 complex | No value | France | SV-1 | Opc+ |
| 41225 | LNP28401 | Neisseria meningitidis | Y | No value | No value | France | SV-5 | Opc+ |
| 41226 | LNP28402 | Neisseria meningitidis | B | ST-269 complex | No value | France | SV-17 | Opc+ |
| 41227 | LNP28403 | Neisseria meningitidis | B | No value | No value | France | SV-12 | Opc- |
| 41228 | LNP28404 | Neisseria meningitidis | B | ST-865 complex | No value | France | SV-5 | Opc+ |
| 41229 | LNP28405 | Neisseria meningitidis | B | ST-32 complex | No value | France | SV-1 | Opc+ |
| 41230 | LNP28267 | Neisseria meningitidis | W | No value | No value | France | SV-7 | Opc+ |
| 41231 | LNP28279 | Neisseria meningitidis | C | ST-11 complex | No value | France | SV-2 | Opc- |
| 41232 | LNP28290 | Neisseria meningitidis | C | ST-11 complex | No value | France | SV-2 | Opc- |
| 41233 | LNP28291 | Neisseria meningitidis | B | ST-213 complex | No value | France | SV-12 | Opc- |
| 41234 | LNP28298 | Neisseria meningitidis | B | ST-461 complex | No value | France | SV-1 | Opc- |
| 41235 | LNP27472 | Neisseria meningitidis | B | ST-32 complex | No value | France | SV-1 | Opc+ |
| 41236 | LNP27547 | Neisseria meningitidis | B | No value | No value | France | SV-6 | Opc+ |
| 41237 | LNP27631 | Neisseria meningitidis | B | No value | No value | France | SV-1 | Opc+ |
| 41238 | LNP27662 | Neisseria meningitidis | B | ST-41/44 complex | No value | France | SV-2 | Opc+ |
| 41239 | LNP27676 | Neisseria meningitidis | B | ST-60 complex | No value | France | SV-11 | Opc+ |
| 41240 | LNP27678 | Neisseria meningitidis | B | ST-32 complex | No value | France | SV-1 | Opc+ |
| 41241 | LNP27700 | Neisseria meningitidis | B | ST-32 complex | No value | France | SV-1 | Opc+ |
| 41242 | LNP27712 | Neisseria meningitidis | B | ST-32 complex | No value | France | SV-1 | Opc+ |
| 41307 | 1359000009 | Neisseria meningitidis | Y | No value | No value | Sweden | SV-11 | Opc+ |
| 41308 | 1359000014 | Neisseria meningitidis | Y | No value | No value | Sweden | SV-5 | Opc+ |
| 41309 | 1359000043 | Neisseria meningitidis | Y | No value | No value | Sweden | SV-11 | Opc+ |
| 41310 | 1359000054 | Neisseria meningitidis | Y | No value | No value | Sweden | SV-11 | Opc+ |
| 41311 | 1359000111 | Neisseria meningitidis | Y | ST-23 complex | No value | Sweden | SV-11 | Opc+ |
| 41312 | 1359000130 | Neisseria meningitidis | Y | ST-23 complex | No value | Sweden | SV-11 | Opc+ |
| 41313 | 1359000142 | Neisseria meningitidis | Y | No value | No value | Sweden | SV-11 | Opc+ |
| 41314 | 1359000144 | Neisseria meningitidis | Y | No value | No value | Sweden | SV-11 | Opc+ |
| 41315 | 1359000194 | Neisseria meningitidis | Y | No value | No value | Sweden | SV-11 | Opc+ |
| 41317 | 1359000319 | Neisseria meningitidis | Y | ST-23 complex | No value | Sweden | SV-11 | Opc+ |
| 41319 | 1359000403 | Neisseria meningitidis | Y | ST-23 complex | No value | Sweden | SV-11 | Opc+ |
| 41320 | 1359000600 | Neisseria meningitidis | Y | ST-23 complex | No value | Sweden | SV-11 | Opc+ |
| 41321 | 1359000669 | Neisseria meningitidis | Y | ST-23 complex | No value | Sweden | SV-11 | Opc+ |
| 41322 | 1459000009 | Neisseria meningitidis | Y | ST-23 complex | No value | Sweden | SV-11 | Opc+ |
| 41323 | 1459000018 | Neisseria meningitidis | Y | ST-23 complex | No value | Sweden | SV-11 | Opc+ |
| 41324 | 1459000020 | Neisseria meningitidis | Y | ST-23 complex | No value | Sweden | SV-11 | Opc+ |
| 41325 | 1459000024 | Neisseria meningitidis | Y | ST-23 complex | No value | Sweden | SV-11 | Opc+ |
| 41326 | 1459000026 | Neisseria meningitidis | Y | ST-23 complex | No value | Sweden | SV-11 | Opc+ |
| 41327 | 1459000134 | Neisseria meningitidis | Y | ST-23 complex | No value | Sweden | SV-11 | Opc+ |
| 41328 | 1459000177 | Neisseria meningitidis | Y | ST-23 complex | No value | Sweden | SV-11 | Opc+ |
| 41329 | 1459000324 | Neisseria meningitidis | Y | ST-23 complex | No value | Sweden | SV-11 | Opc+ |
| 41331 | 1459000563 | Neisseria meningitidis | Y | ST-23 complex | No value | Sweden | SV-11 | Opc+ |
| 41332 | NEIS15-104 | Neisseria meningitidis | Y | ST-23 complex | No value | Sweden | SV-11 | Opc+ |
| 41337 | 95-134 | Neisseria meningitidis | NG | ST-23 complex | No value | Sweden | SV-11 | Opc+ |
| 41338 | 98-182 | Neisseria meningitidis | NG | ST-23 complex | No value | Sweden | SV-11 | Opc+ |
| 41339 | 06-178 | Neisseria meningitidis | NG | ST-23 complex | No value | Sweden | SV-11 | Opc+ |
| 41340 | 11-Jul | Neisseria meningitidis | NG | ST-23 complex | No value | Sweden | SV-11 | Opc+ |
| 41341 | Nov-14 | Neisseria meningitidis | NG | ST-23 complex | No value | Sweden | SV-11 | Opc+ |
| 41342 | 12-176 | Neisseria meningitidis | NG | ST-23 complex | No value | Sweden | SV-11 | Opc+ |
| 41343 | 12-221 | Neisseria meningitidis | NG | ST-23 complex | No value | Sweden | SV-11 | Opc+ |
| 41344 | 12-330 | Neisseria meningitidis | NG | ST-23 complex | No value | Sweden | SV-11 | Opc+ |
| 41413 | 12-584 | Neisseria meningitidis | Y | ST-23 complex | No value | Sweden | SV-11 | Opc+ |
| 41451 | M15 240753 | Neisseria meningitidis | W | ST-11 complex | No value | UK | SV-2 | Opc- |
| 41452 | M15 240846 | Neisseria meningitidis | B | ST-32 complex | No value | UK | SV-1 | Opc+ |

| | | | | | | | | |
|---|---|---|---|---|---|---|---|---|
| 41453 | M15 240853 | Neisseria meningitidis | B | ST-213 complex | No value | UK | SV-12 | Opc- |
| 41454 | M15 240855 | Neisseria meningitidis | B | ST-32 complex | No value | UK | SV-1 | Opc+ |
| 41455 | M15 240856 | Neisseria meningitidis | W | ST-11 complex | No value | UK | SV-2 | Opc- |
| 41456 | M15 240857 | Neisseria meningitidis | B | ST-461 complex | No value | UK | SV-1 | Opc- |
| 41457 | M15 240859 | Neisseria meningitidis | B | ST-269 complex | No value | UK | SV-8 | Opc+ |
| 41458 | M15 240860 | Neisseria meningitidis | W | ST-11 complex | No value | UK | SV-2 | Opc- |
| 41459 | M15 240861 | Neisseria meningitidis | B | ST-11 complex | No value | UK | SV-2 | Opc- |
| 41460 | M15 240862 | Neisseria meningitidis | W | No value | No value | UK | SV-12 | Opc+ |
| 41461 | M15 240863 | Neisseria meningitidis | B | ST-35 complex | No value | UK | SV-16 | Opc+ |
| 41462 | M15 240864 | Neisseria meningitidis | W | ST-11 complex | No value | UK | SV-2 | Opc- |
| 41463 | M15 240866 | Neisseria meningitidis | W | ST-11 complex | No value | UK | SV-2 | Opc- |
| 41464 | M15 240868 | Neisseria meningitidis | B | ST-41/44 complex | No value | UK | SV-2 | Opc+ |
| 41465 | M15 240869 | Neisseria meningitidis | B | ST-269 complex | No value | UK | SV-1 | Opc+ |
| 41466 | M15 240870 | Neisseria meningitidis | Y | ST-23 complex | No value | UK | SV-11 | Opc+ |
| 41467 | M15 240871 | Neisseria meningitidis | W | ST-11 complex | No value | UK | SV-2 | Opc- |
| 41468 | M15 240872 | Neisseria meningitidis | C | ST-11 complex | No value | UK | SV-2 | Opc- |
| 41469 | M15 240873 | Neisseria meningitidis | W | ST-11 complex | No value | UK | SV-2 | Opc- |
| 41470 | M15 240876 | Neisseria meningitidis | W | ST-11 complex | No value | UK | SV-2 | Opc- |
| 41471 | M15 240877 | Neisseria meningitidis | B | ST-41/44 complex | No value | UK | SV-2 | Opc+ |
| 41472 | M15 240878 | Neisseria meningitidis | C | No value | No value | UK | SV-2 | Opc- |
| 41473 | M15 240879 | Neisseria meningitidis | W | ST-11 complex | No value | UK | SV-2 | Opc- |
| 41475 | M15 240881 | Neisseria meningitidis | B | ST-41/44 complex | No value | UK | SV-10 | Opc+ |
| 41476 | M15 240882 | Neisseria meningitidis | Y | ST-23 complex | No value | UK | SV-11 | Opc+ |
| 41477 | M15 240883 | Neisseria meningitidis | B | ST-41/44 complex | No value | UK | SV-2 | Opc+ |
| 41478 | M15 240885 | Neisseria meningitidis | W | ST-11 complex | No value | UK | SV-2 | Opc- |
| 41479 | M15 240886 | Neisseria meningitidis | B | No value | No value | UK | SV-8 | Opc+ |
| 41480 | M15 240887 | Neisseria meningitidis | Y | ST-23 complex | No value | UK | SV-11 | Opc+ |
| 41481 | M15 240888 | Neisseria meningitidis | W | ST-11 complex | No value | UK | SV-2 | Opc- |
| 41482 | M15 240892 | Neisseria meningitidis | B | ST-32 complex | No value | UK | SV-1 | Opc+ |
| 41483 | M15 240893 | Neisseria meningitidis | NG | ST-11 complex | No value | UK | SV-2 | Opc- |
| 41484 | M15 240894 | Neisseria meningitidis | C | No value | No value | UK | SV-2 | Opc- |
| 41485 | M15 240895 | Neisseria meningitidis | B | ST-269 complex | No value | UK | SV-8 | Opc+ |
| 41486 | M15 240896 | Neisseria meningitidis | W | ST-11 complex | No value | UK | SV-2 | Opc- |
| 41487 | M15 240897 | Neisseria meningitidis | B | ST-269 complex | No value | UK | SV-8 | Opc+ |
| 41488 | M15 240899 | Neisseria meningitidis | B | ST-213 complex | No value | UK | SV-12 | Opc- |
| 41489 | M15 240900 | Neisseria meningitidis | W | No value | No value | UK | SV-2 | Opc- |
| 41490 | M15 240901 | Neisseria meningitidis | B | No value | No value | UK | SV-8 | Opc+ |
| 41491 | M15 240902 | Neisseria meningitidis | B | ST-41/44 complex | No value | UK | SV-2 | Opc+ |
| 41492 | M15 240903 | Neisseria meningitidis | B | ST-41/44 complex | No value | UK | SV-10 | Opc+ |
| 41493 | M15 240904 | Neisseria meningitidis | C | ST-11 complex | No value | UK | SV-2 | Opc- |
| 41494 | M15 240905 | Neisseria meningitidis | W | ST-11 complex | No value | UK | SV-2 | Opc- |
| 41495 | M15 240911 | Neisseria meningitidis | Y | ST-23 complex | No value | UK | SV-11 | Opc+ |
| 41496 | M15 240912 | Neisseria meningitidis | B | ST-269 complex | No value | UK | SV-8 | Opc+ |
| 41497 | M15 240913 | Neisseria meningitidis | Y | ST-23 complex | No value | UK | SV-11 | Opc+ |
| 41498 | M15 240914 | Neisseria meningitidis | Y | ST-23 complex | No value | UK | SV-11 | Opc+ |
| 41499 | M15 240915 | Neisseria meningitidis | Y | ST-23 complex | No value | UK | SV-11 | Opc+ |
| 41500 | M15 240917 | Neisseria meningitidis | B | ST-213 complex | No value | UK | SV-12 | Opc- |
| 41501 | M15 240918 | Neisseria meningitidis | W | ST-11 complex | No value | UK | SV-2 | Opc- |
| 41502 | M15 240919 | Neisseria meningitidis | W | ST-11 complex | No value | UK | SV-2 | Opc- |
| 41503 | M15 240920 | Neisseria meningitidis | B | ST-41/44 complex | No value | UK | SV-6 | Opc+ |
| 41504 | M15 240922 | Neisseria meningitidis | Y | No value | No value | UK | SV-11 | Opc+ |
| 41505 | M15 240923 | Neisseria meningitidis | B | ST-41/44 complex | No value | UK | SV-2 | Opc+ |
| 41506 | M15 240924 | Neisseria meningitidis | B | ST-269 complex | No value | UK | SV-8 | Opc+ |
| 41507 | M15 240925 | Neisseria meningitidis | B | ST-41/44 complex | No value | UK | SV-6 | Opc+ |
| 41508 | M15 240926 | Neisseria meningitidis | Y | ST-23 complex | No value | UK | SV-11 | Opc+ |
| 41509 | M15 240929 | Neisseria meningitidis | B | ST-32 complex | No value | UK | SV-1 | Opc+ |
| 41510 | M15 240930 | Neisseria meningitidis | B | ST-32 complex | No value | UK | SV-1 | Opc+ |
| 41511 | M15 240931 | Neisseria meningitidis | B | ST-41/44 complex | No value | UK | SV-10 | Opc+ |
| 41512 | M15 240933 | Neisseria meningitidis | B | ST-41/44 complex | No value | UK | SV-10 | Opc+ |
| 41513 | M15 240934 | Neisseria meningitidis | B | ST-269 complex | No value | UK | SV-1 | Opc+ |
| 41514 | M15 240935 | Neisseria meningitidis | Y | ST-23 complex | No value | UK | SV-11 | Opc+ |
| 41515 | M15 240936 | Neisseria meningitidis | W | ST-11 complex | No value | UK | SV-2 | Opc- |
| 41516 | M15 240937 | Neisseria meningitidis | W | ST-11 complex | No value | UK | SV-2 | Opc- |
| 41517 | M15 240938 | Neisseria meningitidis | Y | ST-23 complex | No value | UK | SV-11 | Opc+ |
| 41518 | M15 240939 | Neisseria meningitidis | B | ST-41/44 complex | No value | UK | SV-2 | Opc+ |
| 41519 | M15 240940 | Neisseria meningitidis | W | ST-11 complex | No value | UK | SV-2 | Opc- |
| 41520 | M15 240941 | Neisseria meningitidis | Y | No value | No value | UK | SV-11 | Opc+ |
| 41521 | M15 240945 | Neisseria meningitidis | Y | ST-23 complex | No value | UK | SV-11 | Opc+ |
| 41522 | M15 240946 | Neisseria meningitidis | W | ST-11 complex | No value | UK | SV-2 | Opc- |
| 41523 | M15 240947 | Neisseria meningitidis | B | No value | No value | UK | SV-5 | Opc- |
| 41524 | M15 240949 | Neisseria meningitidis | W | No value | No value | UK | SV-11 | Opc+ |
| 41525 | M15 240952 | Neisseria meningitidis | W | ST-11 complex | No value | UK | SV-2 | Opc- |
| 41526 | M15 240953 | Neisseria meningitidis | NG | ST-175 complex | No value | UK | SV-5 | Opc+ |
| 41527 | M15 240954 | Neisseria meningitidis | B | ST-213 complex | No value | UK | SV-1 | Opc- |

| | | | | | | | | |
|---|---|---|---|---|---|---|---|---|
| 41529 | M15 240957 | Neisseria meningitidis | Y | ST-23 complex | No value | UK | SV-11 | Opc+ |
| 41530 | M15 240958 | Neisseria meningitidis | W | ST-11 complex | No value | UK | SV-2 | Opc- |
| 41531 | M15 240960 | Neisseria meningitidis | W | ST-11 complex | No value | UK | SV-2 | Opc- |
| 41532 | M15 240961 | Neisseria meningitidis | B | No value | No value | UK | SV-8 | Opc+ |
| 41533 | M15 240963 | Neisseria meningitidis | W | ST-11 complex | No value | UK | SV-2 | Opc- |
| 41534 | M15 240965 | Neisseria meningitidis | Y | ST-23 complex | No value | UK | SV-11 | Opc+ |
| 41535 | M15 240967 | Neisseria meningitidis | Y | ST-23 complex | No value | UK | SV-11 | Opc+ |
| 41536 | M15 240968 | Neisseria meningitidis | W | No value | No value | UK | SV-2 | Opc- |
| 41537 | M15 240969 | Neisseria meningitidis | B | ST-41/44 complex | No value | UK | SV-2 | Opc+ |
| 41538 | M15 240973 | Neisseria meningitidis | B | ST-41/44 complex | No value | UK | SV-2 | Opc+ |
| 41539 | M15 240974 | Neisseria meningitidis | W | ST-22 complex | No value | UK | SV-12 | Opc+ |
| 41540 | M15 240975 | Neisseria meningitidis | B | ST-269 complex | No value | UK | SV-8 | Opc+ |
| 41542 | M15 240977 | Neisseria meningitidis | B | No value | No value | UK | SV-12 | Opc- |
| 41543 | M15 240978 | Neisseria meningitidis | W | ST-11 complex | No value | UK | SV-2 | Opc- |
| 41544 | M15 240979 | Neisseria meningitidis | B | No value | No value | UK | SV-2 | Opc+ |
| 41545 | M15 240980 | Neisseria meningitidis | B | No value | No value | UK | SV-1 | Opc+ |
| 41546 | M15 240981 | Neisseria meningitidis | W | ST-11 complex | No value | UK | SV-2 | Opc- |
| 41547 | M15 240983 | Neisseria meningitidis | W | ST-11 complex | No value | UK | SV-2 | Opc- |
| 41548 | M15 240984 | Neisseria meningitidis | B | ST-60 complex | No value | UK | SV-10 | Opc+ |
| 41549 | M15 240985 | Neisseria meningitidis | W | ST-11 complex | No value | UK | SV-2 | Opc- |
| 41550 | M15 240986 | Neisseria meningitidis | B | ST-41/44 complex | No value | UK | SV-2 | Opc+ |
| 41551 | M15 240987 | Neisseria meningitidis | Y | ST-174 complex | No value | UK | SV-1 | Opc+ |
| 41552 | M15 240988 | Neisseria meningitidis | W | ST-11 complex | No value | UK | SV-2 | Opc- |
| 41553 | M15 240989 | Neisseria meningitidis | Y | ST-23 complex | No value | UK | SV-11 | Opc+ |
| 41554 | M15 240990 | Neisseria meningitidis | B | ST-41/44 complex | No value | UK | SV-2 | Opc+ |
| 41555 | M15 240991 | Neisseria meningitidis | B | ST-60 complex | No value | UK | SV-11 | Opc+ |
| 41556 | M15 240992 | Neisseria meningitidis | Y | ST-23 complex | No value | UK | SV-11 | Opc+ |
| 41557 | M15 240994 | Neisseria meningitidis | Y | ST-23 complex | No value | UK | SV-11 | Opc+ |
| 41558 | M15 240995 | Neisseria meningitidis | W | ST-11 complex | No value | UK | SV-2 | Opc- |
| 41559 | M15 240996 | Neisseria meningitidis | B | ST-41/44 complex | No value | UK | SV-2 | Opc+ |
| 41560 | M15 240982 | Neisseria meningitidis | W | ST-11 complex | No value | UK | SV-2 | Opc- |
| 41564 | 107519 | Neisseria meningitidis | C | ST-11 complex | No value | Finland | SV-2 | Opc- |
| 41565 | 107520 | Neisseria meningitidis | C | ST-11 complex | No value | Finland | SV-2 | Opc- |
| 41566 | 107518 | Neisseria meningitidis | Y | ST-23 complex | No value | Finland | SV-11 | Opc+ |
| 41567 | 107515 | Neisseria meningitidis | B | No value | No value | Finland | SV-12 | Opc+ |
| 41568 | 107516 | Neisseria meningitidis | B | No value | No value | Finland | SV-12 | Opc+ |
| 41569 | 107514 | Neisseria meningitidis | B | ST-41/44 complex | No value | Finland | SV-6 | Opc+ |
| 41570 | 107517 | Neisseria meningitidis | C | ST-11 complex | No value | Finland | SV-2 | Opc- |
| 41571 | 107496 | Neisseria meningitidis | B | ST-41/44 complex | No value | Finland | SV-6 | Opc+ |
| 41576 | LNP28407 | Neisseria meningitidis | B | ST-213 complex | No value | France | SV-12 | Opc- |
| 41577 | LNP28409 | Neisseria meningitidis | Y | ST-23 complex | No value | France | SV-11 | Opc+ |
| 41578 | LNP28411 | Neisseria meningitidis | C | ST-11 complex | No value | France | SV-2 | Opc- |
| 41579 | LNP28412 | Neisseria meningitidis | B | ST-32 complex | No value | France | SV-1 | Opc+ |
| 41580 | LNP28413 | Neisseria meningitidis | C | ST-11 complex | No value | France | SV-2 | Opc- |
| 41581 | LNP28414 | Neisseria meningitidis | Y | ST-23 complex | No value | France | SV-11 | Opc+ |
| 41582 | LNP28415 | Neisseria meningitidis | W | ST-11 complex | No value | France | SV-2 | Opc- |
| 41583 | LNP28416 | Neisseria meningitidis | C | ST-11 complex | No value | France | SV-2 | Opc- |
| 41585 | LNP28418 | Neisseria meningitidis | B | ST-32 complex | No value | France | SV-1 | Opc+ |
| 41586 | LNP28420 | Neisseria meningitidis | C | ST-11 complex | No value | France | SV-2 | Opc- |
| 41587 | LNP28422 | Neisseria meningitidis | C | ST-11 complex | No value | France | SV-2 | Opc- |
| 41588 | LNP27469 | Neisseria meningitidis | B | ST-32 complex | No value | France | SV-1 | Opc+ |
| 41589 | LNP27547 | Neisseria meningitidis | B | No value | No value | France | SV-6 | Opc+ |
| 41590 | LNP27612 | Neisseria meningitidis | B | ST-213 complex | No value | France | SV-2 | Opc- |
| 41591 | LNP27725 | Neisseria meningitidis | B | ST-32 complex | No value | France | SV-1 | Opc+ |
| 41592 | LNP27700 | Neisseria meningitidis | B | ST-32 complex | No value | France | SV-1 | Opc+ |
| 41593 | LNP28279 | Neisseria meningitidis | C | ST-11 complex | No value | France | SV-2 | Opc- |
| 41598 | 107523 | Neisseria meningitidis | B | No value | No value | Finland | SV-5 | Opc- |
| 41599 | 145M | Neisseria meningitidis | B | ST-41/44 complex | Sporadic case | Slovenia | SV-6 | Opc+ |
| 41600 | 147M | Neisseria meningitidis | B | ST-269 complex | Sporadic case | Slovenia | SV-8 | Opc+ |
| 41601 | 148M | Neisseria meningitidis | B | ST-41/44 complex | Sporadic case | Slovenia | SV-5 | Opc- |
| 41602 | 150M | Neisseria meningitidis | B | ST-269 complex | Sporadic case | Slovenia | SV-13 | Opc+ |
| 41611 | 09-292 | Neisseria meningitidis | NG | ST-23 complex | No value | Sweden | SV-11 | Opc+ |
| 41614 | 2686 | Neisseria meningitidis | NG | ST-11 complex | Endemic | Italy | SV-2 | Opc- |
| 41615 | W-5295 | Neisseria meningitidis | B | ST-32 complex | Sporadic case | Greece | SV-1 | Opc+ |
| 41616 | W-5302 | Neisseria meningitidis | B | ST-35 complex | Sporadic case | Greece | SV-12 | Opc+ |
| 41617 | W-5316 | Neisseria meningitidis | B | ST-32 complex | Sporadic case | Greece | SV-1 | Opc+ |
| 41618 | W-5321 | Neisseria meningitidis | B | ST-60 complex | Sporadic case | Greece | SV-11 | Opc+ |
| 41619 | W-5347 | Neisseria meningitidis | B | ST-213 complex | Sporadic case | Greece | SV-12 | Opc- |
| 41620 | W-5358 | Neisseria meningitidis | B | ST-32 complex | Sporadic case | Greece | SV-1 | Opc+ |
| 41621 | W-5371 | Neisseria meningitidis | B | ST-35 complex | Sporadic case | Greece | SV-12 | Opc+ |
| 41622 | W-5376 | Neisseria meningitidis | B | ST-865 complex | Sporadic case | Greece | SV-2 | Opc+ |
| 41623 | W-5379 | Neisseria meningitidis | B | ST-213 complex | Sporadic case | Greece | SV-12 | Opc- |
| 41624 | W-5551 | Neisseria meningitidis | B | No value | Sporadic case | Greece | SV-7 | Opc+ |
| 41625 | W-5573 | Neisseria meningitidis | B | ST-269 complex | Sporadic case | Greece | SV-1 | Opc+ |

| | | | | | | | | |
|---|---|---|---|---|---|---|---|---|
| 41626 | W-5731 | Neisseria meningitidis | B | ST-35 complex | Sporadic case | Greece | SV-12 | Opc+ |
| 41627 | W-5779 | Neisseria meningitidis | B | ST-35 complex | Sporadic case | Greece | SV-12 | Opc+ |
| 41628 | W-5792 | Neisseria meningitidis | Y | ST-23 complex | Sporadic case | Greece | SV-11 | Opc+ |
| 41629 | W-5818 | Neisseria meningitidis | B | ST-32 complex | Sporadic case | Greece | SV-1 | Opc+ |
| 41630 | W-5827 | Neisseria meningitidis | B | ST-865 complex | Sporadic case | Greece | SV-2 | Opc+ |
| 41631 | AK-1830 | Neisseria meningitidis | B | ST-269 complex | Sporadic case | Greece | SV-8 | Opc+ |
| 41632 | AK-1855 | Neisseria meningitidis | B | ST-213 complex | Sporadic case | Greece | SV-12 | Opc- |
| 41633 | AK-1894 | Neisseria meningitidis | B | ST-269 complex | Sporadic case | Greece | SV-1 | Opc+ |
| 41634 | AK-1896 | Neisseria meningitidis | B | ST-269 complex | Sporadic case | Greece | SV-8 | Opc+ |
| 41635 | AK-1904 | Neisseria meningitidis | B | ST-35 complex | Sporadic case | Greece | SV-12 | Opc+ |
| 41636 | BM-1218 | Neisseria meningitidis | B | ST-162 complex | Sporadic case | Greece | SV-1 | Opc+ |
| 41637 | BM-1238 | Neisseria meningitidis | B | ST-162 complex | Sporadic case | Greece | SV-1 | Opc+ |
| 41638 | K-30 | Neisseria meningitidis | B | ST-41/44 complex | Sporadic case | Cyprus | SV-2 | Opc+ |
| 41640 | 2733 | Neisseria meningitidis | W | ST-22 complex | Endemic | Italy | SV-12 | Opc+ |
| 41641 | 2737 | Neisseria meningitidis | W | ST-22 complex | Endemic | Italy | SV-12 | Opc+ |
| 41642 | 2738 | Neisseria meningitidis | C | ST-11 complex | Endemic | Italy | SV-2 | Opc- |
| 41643 | 2739 | Neisseria meningitidis | B | ST-60 complex | Endemic | Italy | SV-11 | Opc+ |
| 41644 | 2740 | Neisseria meningitidis | Y | ST-92 complex | Endemic | Italy | SV-1 | Opc+ |
| 41645 | 2741 | Neisseria meningitidis | B | ST-162 complex | Endemic | Italy | SV-1 | Opc+ |
| 41646 | 2742 | Neisseria meningitidis | C | ST-11 complex | Endemic | Italy | SV-2 | Opc- |
| 41647 | 2743 | Neisseria meningitidis | C | ST-11 complex | Endemic | Italy | SV-2 | Opc- |
| 41648 | 2744 | Neisseria meningitidis | C | ST-11 complex | Endemic | Italy | SV-2 | Opc- |
| 41649 | 2745 | Neisseria meningitidis | Y | ST-23 complex | Endemic | Italy | SV-11 | Opc+ |
| 41650 | 2746 | Neisseria meningitidis | Y | ST-167 complex | Endemic | Italy | SV-5 | Opc+ |
| 41651 | 2747 | Neisseria meningitidis | C | ST-11 complex | Endemic | Italy | SV-2 | Opc- |
| 41653 | 2749 | Neisseria meningitidis | Y | ST-23 complex | Endemic | Italy | SV-11 | Opc+ |
| 41654 | 2750 | Neisseria meningitidis | C | ST-334 complex | Endemic | Italy | SV-15 | Opc- |
| 41655 | 2751 | Neisseria meningitidis | B | ST-41/44 complex | Endemic | Italy | SV-6 | Opc+ |
| 41656 | 2752 | Neisseria meningitidis | Y | ST-23 complex | Endemic | Italy | SV-11 | Opc+ |
| 41657 | 2753 | Neisseria meningitidis | B | No value | Endemic | Italy | SV-1 | Opc+ |
| 41658 | 2754 | Neisseria meningitidis | B | ST-32 complex | Endemic | Italy | SV-1 | Opc+ |
| 41659 | 2755 | Neisseria meningitidis | C | ST-11 complex | Endemic | Italy | SV-2 | Opc- |
| 41660 | 2756 | Neisseria meningitidis | W | ST-22 complex | Endemic | Italy | SV-12 | Opc+ |
| 41661 | 2757 | Neisseria meningitidis | C | ST-11 complex | Endemic | Italy | SV-2 | Opc- |
| 41662 | 2759 | Neisseria meningitidis | B | ST-41/44 complex | Endemic | Italy | SV-2 | Opc+ |
| 41663 | 2760 | Neisseria meningitidis | B | ST-1157 complex | Endemic | Italy | SV-1 | Opc+ |
| 41664 | 2764 | Neisseria meningitidis | B | ST-269 complex | Endemic | Italy | SV-8 | Opc+ |
| 41665 | 2774 | Neisseria meningitidis | C | ST-11 complex | Endemic | Italy | SV-2 | Opc- |
| 41666 | 2776 | Neisseria meningitidis | C | ST-11 complex | Endemic | Italy | SV-2 | Opc- |
| 41667 | 2777 | Neisseria meningitidis | C | ST-11 complex | Endemic | Italy | SV-2 | Opc- |
| 41668 | 2778 | Neisseria meningitidis | C | ST-11 complex | Endemic | Italy | SV-2 | Opc- |
| 41669 | LNP28423 | Neisseria meningitidis | B | ST-41/44 complex | No value | France | SV-2 | Opc+ |
| 41670 | LNP28424 | Neisseria meningitidis | B | No value | No value | France | SV-8 | Opc+ |
| 41671 | LNP28425 | Neisseria meningitidis | C | ST-11 complex | No value | France | SV-2 | Opc- |
| 41672 | LNP28428 | Neisseria meningitidis | NG | ST-53 complex | No value | France | SV-2 | Opc- |
| 41673 | LNP28429 | Neisseria meningitidis | C | ST-11 complex | No value | France | SV-2 | Opc- |
| 41674 | LNP28430 | Neisseria meningitidis | Y | ST-23 complex | No value | France | SV-11 | Opc+ |
| 41675 | LNP28431 | Neisseria meningitidis | C | ST-11 complex | No value | France | SV-2 | Opc- |
| 41676 | LNP28432 | Neisseria meningitidis | B | ST-32 complex | No value | France | SV-1 | Opc+ |
| 41677 | LNP28433 | Neisseria meningitidis | B | ST-269 complex | No value | France | SV-8 | Opc+ |
| 41678 | 2766 | Neisseria meningitidis | C | ST-11 complex | Endemic | Italy | SV-2 | Opc- |
| 41679 | 2763 | Neisseria meningitidis | B | ST-162 complex | Endemic | Italy | SV-1 | Opc+ |
| 41683 | 107524 | Neisseria meningitidis | B | ST-269 complex | No value | Finland | SV-1 | Opc+ |
| 41684 | 76Mo | Neisseria meningitidis | Y | ST-167 complex | Sporadic case | Slovenia | SV-5 | Opc+ |
| 41685 | 77Mo | Neisseria meningitidis | Z | ST-60 complex | Sporadic case | Slovenia | SV-11 | Opc+ |
| 41686 | 78Mo | Neisseria meningitidis | B | No value | Sporadic case | Slovenia | SV-1 | Opc+ |
| 41687 | 79Mo | Neisseria meningitidis | Y | ST-23 complex | Sporadic case | Slovenia | SV-15 | Opc+ |
| 41688 | 80Mo | Neisseria meningitidis | Y | ST-167 complex | Sporadic case | Slovenia | SV-5 | Opc+ |
| 41689 | 81Mo | Neisseria meningitidis | B | No value | Sporadic case | Slovenia | SV-6 | Opc+ |
| 41690 | 82Mo | Neisseria meningitidis | B | ST-41/44 complex | Sporadic case | Slovenia | SV-5 | Opc- |
| 41691 | 83Mo | Neisseria meningitidis | Y | No value | Sporadic case | Slovenia | SV-15 | Opc+ |
| 41692 | 84Mo | Neisseria meningitidis | B | ST-41/44 complex | Sporadic case | Slovenia | SV-6 | Opc+ |
| 41693 | 86Mo | Neisseria meningitidis | B | ST-32 complex | Sporadic case | Slovenia | SV-1 | Opc+ |
| 41694 | 88Mo | Neisseria meningitidis | B | ST-32 complex | Sporadic case | Slovenia | SV-1 | Opc+ |
| 41695 | 98Mo | Neisseria meningitidis | C | ST-11 complex | Sporadic case | Slovenia | SV-2 | Opc- |
| 41696 | 127M | Neisseria meningitidis | C | ST-8 complex | Sporadic case | Slovenia | SV-10 | Opc- |
| 41697 | 128M | Neisseria meningitidis | B | ST-231 complex | Sporadic case | Slovenia | SV-7 | Opc- |
| 41698 | 129M | Neisseria meningitidis | B | ST-461 complex | Sporadic case | Slovenia | SV-1 | Opc- |
| 41699 | 130M | Neisseria meningitidis | B | ST-41/44 complex | Sporadic case | Slovenia | SV-6 | Opc+ |
| 41700 | 131M | Neisseria meningitidis | B | ST-269 complex | Sporadic case | Slovenia | SV-8 | Opc+ |
| 41701 | 132M | Neisseria meningitidis | B | ST-32 complex | Sporadic case | Slovenia | SV-1 | Opc+ |
| 41702 | 133M | Neisseria meningitidis | B | ST-32 complex | Sporadic case | Slovenia | SV-1 | Opc+ |
| 41703 | 134M | Neisseria meningitidis | B | ST-32 complex | Sporadic case | Slovenia | SV-1 | Opc+ |
| 41704 | 135M | Neisseria meningitidis | B | ST-41/44 complex | Sporadic case | Slovenia | SV-6 | Opc+ |

| | | | | | | | | |
|---|---|---|---|---|---|---|---|---|
| 41705 | 136M | Neisseria meningitidis | B | ST-162 complex | Sporadic case | Slovenia | SV-1 | Opc+ |
| 41706 | 137M | Neisseria meningitidis | B | ST-41/44 complex | Sporadic case | Slovenia | SV-2 | Opc+ |
| 41707 | 138M | Neisseria meningitidis | C | ST-41/44 complex | Sporadic case | Slovenia | SV-10 | Opc+ |
| 41708 | 139M | Neisseria meningitidis | B | No value | Sporadic case | Slovenia | SV-1 | Opc+ |
| 41709 | 140M | Neisseria meningitidis | B | No value | Sporadic case | Slovenia | SV-8 | Opc+ |
| 41710 | 141M | Neisseria meningitidis | B | ST-41/44 complex | Sporadic case | Slovenia | SV-6 | Opc+ |
| 41711 | 142M | Neisseria meningitidis | B | No value | Sporadic case | Slovenia | SV-2 | Opc+ |
| 41712 | 143M | Neisseria meningitidis | B | ST-213 complex | Sporadic case | Slovenia | SV-6 | Opc- |
| 41713 | 144M | Neisseria meningitidis | B | ST-269 complex | Sporadic case | Slovenia | SV-8 | Opc+ |
| 41714 | 153M | Neisseria meningitidis | B | ST-213 complex | Sporadic case | Slovenia | SV-12 | Opc- |
| 41715 | 154M | Neisseria meningitidis | B | ST-269 complex | Sporadic case | Slovenia | SV-8 | Opc+ |
| 41716 | 91Mo | Neisseria meningitidis | B | ST-41/44 complex | Sporadic case | Slovenia | SV-6 | Opc+ |
| 41717 | 149M | Neisseria meningitidis | C | ST-11 complex | Sporadic case | Slovenia | SV-2 | Opc- |
| 41718 | 151M | Neisseria meningitidis | B | ST-41/44 complex | Sporadic case | Slovenia | SV-6 | Opc+ |
| 41719 | 152M | Neisseria meningitidis | B | ST-213 complex | Sporadic case | Slovenia | SV-12 | Opc- |
| 41726 | LNP28434 | Neisseria meningitidis | B | ST-162 complex | No value | France | SV-1 | Opc+ |
| 41727 | LNP28435 | Neisseria meningitidis | NG | ST-175 complex | No value | France | SV-5 | Opc+ |
| 41728 | LNP28436 | Neisseria meningitidis | W | ST-11 complex | No value | France | SV-2 | Opc- |
| 41729 | LNP28437 | Neisseria meningitidis | Y | ST-23 complex | No value | France | SV-11 | Opc+ |
| 41730 | LNP28438 | Neisseria meningitidis | B | ST-41/44 complex | No value | France | SV-10 | Opc+ |
| 41731 | LNP28439 | Neisseria meningitidis | B | ST-461 complex | No value | France | SV-1 | Opc- |
| 41732 | LNP28440 | Neisseria meningitidis | C | ST-11 complex | No value | France | SV-2 | Opc- |
| 41733 | LNP28441 | Neisseria meningitidis | B | ST-32 complex | No value | France | SV-1 | Opc+ |
| 41734 | LNP27734 | Neisseria meningitidis | Y | No value | No value | France | SV-11 | Opc+ |
| 41735 | LNP27747 | Neisseria meningitidis | Y | No value | No value | France | SV-5 | Opc+ |
| 41736 | LNP27751 | Neisseria meningitidis | Y | ST-23 complex | No value | France | SV-11 | Opc+ |
| 41737 | LNP27772 | Neisseria meningitidis | Y | No value | No value | France | SV-11 | Opc+ |
| 41738 | LNP27804 | Neisseria meningitidis | Y | No value | No value | France | SV-11 | Opc+ |
| 41739 | LNP27830 | Neisseria meningitidis | Y | No value | No value | France | SV-11 | Opc+ |
| 41740 | LNP27832 | Neisseria meningitidis | Y | ST-23 complex | No value | France | SV-11 | Opc+ |
| 41741 | LNP27836 | Neisseria meningitidis | Y | ST-167 complex | No value | France | SV-5 | Opc+ |
| 41742 | LNP27841 | Neisseria meningitidis | Y | ST-23 complex | No value | France | SV-11 | Opc+ |
| 41743 | LNP27852 | Neisseria meningitidis | Y | ST-167 complex | No value | France | SV-5 | Opc+ |
| 41744 | LNP27859 | Neisseria meningitidis | Y | No value | No value | France | SV-11 | Opc+ |
| 41745 | LNP27890 | Neisseria meningitidis | Y | ST-174 complex | No value | France | SV-1 | Opc+ |
| 41746 | LNP27893 | Neisseria meningitidis | Y | ST-23 complex | No value | France | SV-11 | Opc+ |
| 41747 | LNP27904 | Neisseria meningitidis | Y | ST-167 complex | No value | France | SV-5 | Opc+ |
| 41748 | LNP27918 | Neisseria meningitidis | Y | ST-103 complex | No value | France | SV-5 | Opc+ |
| 41749 | LNP27919 | Neisseria meningitidis | Y | ST-23 complex | No value | France | SV-11 | Opc+ |
| 41750 | LNP27945 | Neisseria meningitidis | Y | ST-174 complex | No value | France | SV-1 | Opc+ |
| 41751 | LNP27955 | Neisseria meningitidis | Y | ST-23 complex | No value | France | SV-11 | Opc+ |
| 41752 | LNP27970 | Neisseria meningitidis | Y | ST-23 complex | No value | France | SV-11 | Opc+ |
| 41753 | LNP27972 | Neisseria meningitidis | Y | No value | No value | France | SV-1 | Opc+ |
| 41754 | LNP27987 | Neisseria meningitidis | Y | ST-23 complex | No value | France | SV-11 | Opc+ |
| 41755 | LNP27995 | Neisseria meningitidis | Y | ST-167 complex | No value | France | SV-5 | Opc+ |
| 41756 | LNP27997 | Neisseria meningitidis | Y | ST-23 complex | No value | France | SV-11 | Opc+ |
| 41757 | LNP27998 | Neisseria meningitidis | Y | ST-167 complex | No value | France | SV-5 | Opc+ |
| 41758 | LNP27999 | Neisseria meningitidis | Y | No value | No value | France | SV-5 | Opc+ |
| 41759 | LNP28007 | Neisseria meningitidis | Y | No value | No value | France | SV-11 | Opc+ |
| 41760 | LNP28012 | Neisseria meningitidis | Y | ST-103 complex | No value | France | SV-5 | Opc+ |
| 41761 | LNP28020 | Neisseria meningitidis | Y | ST-23 complex | No value | France | SV-11 | Opc+ |
| 41762 | LNP28024 | Neisseria meningitidis | Y | No value | No value | France | SV-11 | Opc+ |
| 41763 | LNP28028 | Neisseria meningitidis | Y | ST-23 complex | No value | France | SV-5 | Opc+ |
| 41764 | LNP28034 | Neisseria meningitidis | Y | No value | No value | France | SV-11 | Opc+ |
| 41765 | LNP28035 | Neisseria meningitidis | Y | No value | No value | France | SV-11 | Opc+ |
| 41766 | LNP28056 | Neisseria meningitidis | Y | ST-23 complex | No value | France | SV-11 | Opc+ |
| 41767 | LNP28057 | Neisseria meningitidis | Y | ST-167 complex | No value | France | SV-5 | Opc+ |
| 41768 | LNP28077 | Neisseria meningitidis | Y | No value | No value | France | SV-11 | Opc+ |
| 41769 | LNP28081 | Neisseria meningitidis | Y | ST-23 complex | No value | France | SV-11 | Opc+ |
| 41770 | LNP28093 | Neisseria meningitidis | Y | ST-103 complex | No value | France | SV-5 | Opc+ |
| 41771 | LNP28094 | Neisseria meningitidis | Y | ST-103 complex | No value | France | SV-5 | Opc+ |
| 41772 | LNP28095 | Neisseria meningitidis | Y | ST-23 complex | No value | France | SV-11 | Opc+ |
| 41773 | LNP28096 | Neisseria meningitidis | Y | ST-23 complex | No value | France | SV-11 | Opc+ |
