# Supplementary material for: Bioinformatic analysis of meningococcal Msf and Opc to inform vaccine antigen design
Source: PLoS One. 2018 Mar 16;13(3):e0193940. doi: 10.1371/journal.pone.0193940 (PMC5856348; doi:10.1371/journal.pone.0193940)
Supplement: S1 Fig — (PDF) [file pone.0193940.s005.pdf]

Materials and Methods:

Serum bactericidal assay (SBA)

Serum bactericidal activity of purified anti-MsfSV1/2/5, and anti-Opc loop2 (raised against a synthetic peptide of Opc loop 2 conjugated to KLH) was determined using 10% normal human serum against *N. meningitidis* (strain G7/4) and a control strain lacking both *opc* and *msf* (H44/76 Δ*opc*Δ*msf*). The bacteria were resuspended in DBPS prior to dilution to give a final concentration of 104 cfu in total volume of 100 µL, containing the purified antibodies (0-10 µg/mL) and 10% normal human serum, or 10% decomplemented serum (heat inactivated at 56oC for 30 minutes). Cell counts were performed by plating bacterial cells onto HBHI agar plates at 0 minutes and after 60 minutes incubation at 37oC with 5% CO_2. Percentage survival was measured by dividing the CFU counts after 60 minutes incubation with the number of bacteria at the start of the assay. The experiment was performed using three technical replicates of three biological replicates.

Opc loop-2 synthetic peptide

A synthetic peptide of loop-2 of Opc (KKNIINLETDENKLGKTKNVKLPTGVP) N-terminally conjugated to KLH was synthesised by Cambridge Research Biochemicals prior to immunisation. Rabbit immunisations were performed by Cambridge Research Biochemicals following a 77 day immunisation protocol with 2mg of Opc synthetic peptide with Freunds complete adjuvant. This antisera is referred to as anti-Opc loop2-KLH.

Competitive ELISA

Immulon 2HB ELISA plates were coated overnight with 100 µl Msf recombinant rMsf-SV149-189 in 50 mM carbonate buffer (pH 9.6). Control wells were coated with 3% BSA. Unbound protein was removed by washing with ELISA wash (154 mM NaCl containing 0.05% Tween-20). To determine whether anti-Msf SV1/2/5 could inhibit Vn binding to Msf, wells were blocked with 3% BSA, 0-40 µg/mL. Subsequently, anti-Msf SV1/2/5 was added to the appropriate wells for 30 minutes prior to the addition of 0.2 µg vitronectin for 1h at RT. The plates were then washed four times with ELISA wash and level of Vn binding determined using mouse anti-vitronectin MsX (8E6 clone; Millipore) and a secondary anti-mouse antibody conjugated to alkaline phosphatase. ELISA plates were developed using SigmaFast p-Nitrophenyl phosphate substrate and the absorbance was measured at 405nm. All ELISA experiments were repeated in triplicate, with the result showing averages of three independent biological replicates.

Results:

Serum Bactericidal and function blocking activity of purified polyclonal rabbit antibodies produced against the Vn binding domains of Msf and Opc

Previously we have shown Opc and Msf to play roles in vitronectin binding and serum resistance, highlighting their importance as meningococcal vaccine candidates (Griffiths, et al. 2011; Hill et al. 2015; Cunha Sa E et al 2010). Our current studies have shown that anti-MsfSV1/2/5 elicits antibodies that display both serum bactericidal and function blocking activity (Figure S1A and B), with a 28% increase in killing seen against *N. meningitidis* in 10%

normal human serum (Figure S1A), and a 41% inhibition of vitronectin binding to rMsfSV149-189 (Figure S1B). We have also demonstrated antibodies against a synthetic peptide Opc (loop 2) to elicit bactericidal activity (Figure S1A), with 10 µg/mL purified anti-Opc-loop2-KLH increasing bactericidal killing by 45% (Figure S1A).

Fig S1

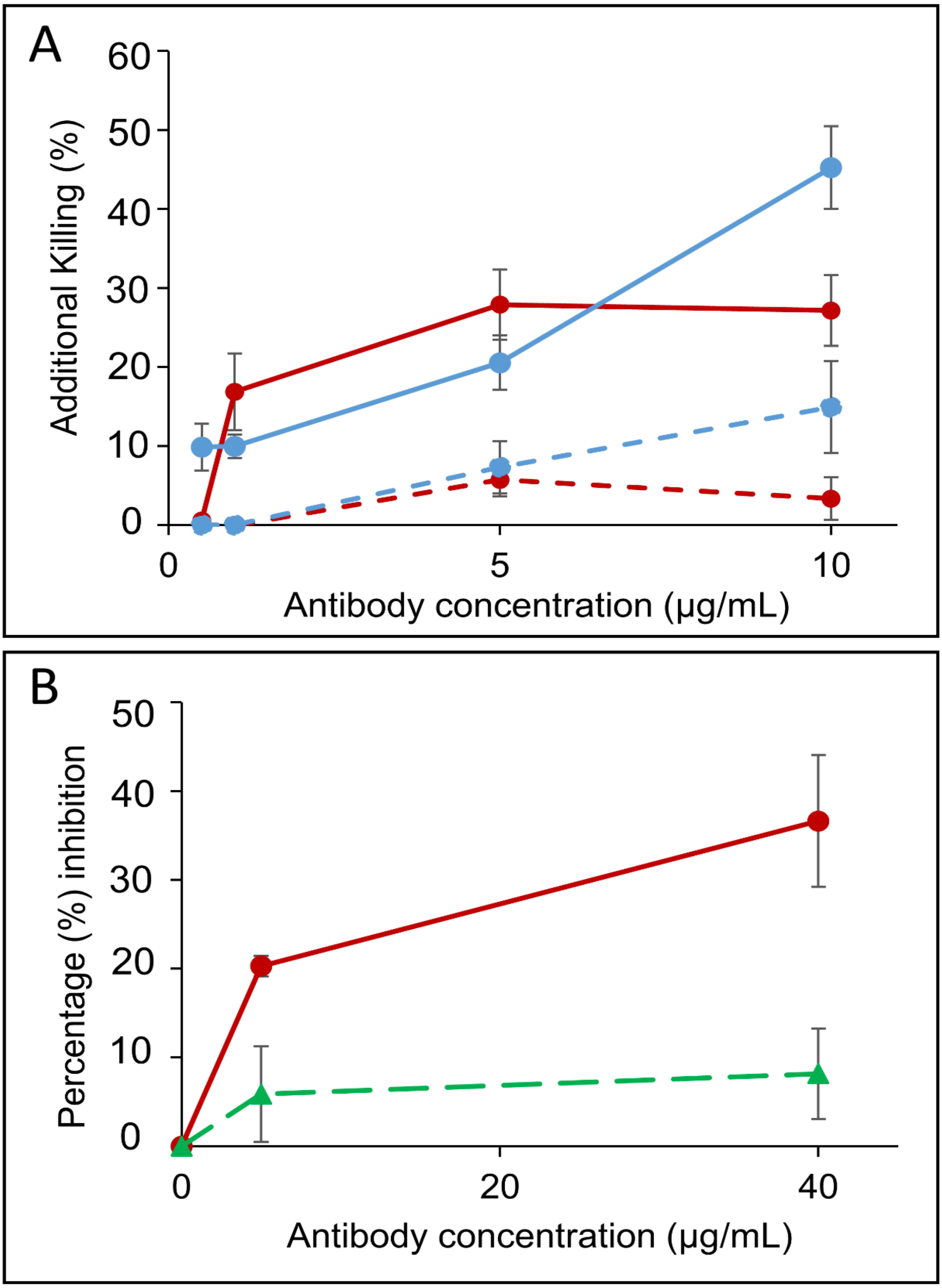
A
60
50
40
30
20
10
0
Additional Killing (%)
0
5
10
Antibody concentration (µg/mL)
B
50
40
30
20
10
0
Percentage (%) inhibition
0
20
40
Antibody concentration (µg/mL)

Figure S1 – Antibodies against the functional domains of Msf or Opc are serum bactericidal and function blocking. A) Serum bactericidal activity of polyclonal antisera raised against recombinant proteins of three Msf SV's (anti-Msf SV1/2/5; red lines) or an Opc loop 2 synthetic peptide conjugated to KLH (anti-Opc loop2; blue lines). Killing was assessed using 10% normal human serum (NHS) against *N. meningitidis* expressing Msf and Opc (solid lines) or a double knockout mutant lacking both antigens (dashed lines). Both anti-Msf SV1/2/5 and anti-Opc loop2 displayed serum bactericidal activity, with a 28% or 45% respective increase in killing observed compared to the control strain. B) Competitive ELISA was performed by coating an ELISA plate with 3 µM rMsfSV-1_{49-189}, prior to overlay with either anti-Msf SV1/2/5 (red circles solid line) or anti-CEACAM negative control (green triangles dashed line) and subsequent addition of 0.2 µg Vn. Vitronectin binding was detected using murine anti-vitronectin (8E6) and mouse anti-alkaline phosphatase as the secondary antibody. Results show anti-Msf SV1/2/5 to cause a 41% inhibition of vitronectin binding to purified Msf recombinant protein. Results are the mean and standard error of three to four independent biological replicates each performed in triplicate.

References:

Griffiths NJ, et al. 2011. Meningococcal surface fibril (Msf) binds to activated vitronectin and inhibits the terminal complement pathway to increase serum resistance. Mol Microbiol 82: 1129-1149. doi: 10.1111/j.1365-2958.2011.07876.x

Hill DJ, et al. 2015. Identification and therapeutic potential of a vitronectin binding region of meningococcal msf. PLoS One 10: e0124133. doi: 10.1371/journal.pone.0124133

Sa ECC, Griffiths NJ, Virji M 2010. Neisseria meningitidis Opc invasin binds to the sulphated tyrosines of activated vitronectin to attach to and invade human brain endothelial cells. PLoS Pathog 6: e1000911. doi: 10.1371/journal.ppat.1000911
