# Supplementary material for: Bioinformatic analysis of meningococcal Msf and Opc to inform vaccine antigen design
Source: PLoS One. 2018 Mar 16;13(3):e0193940. doi: 10.1371/journal.pone.0193940 (PMC5856348; doi:10.1371/journal.pone.0193940)
Supplement: S2 Fig — (PDF) [file pone.0193940.s006.pdf]

Fig S2

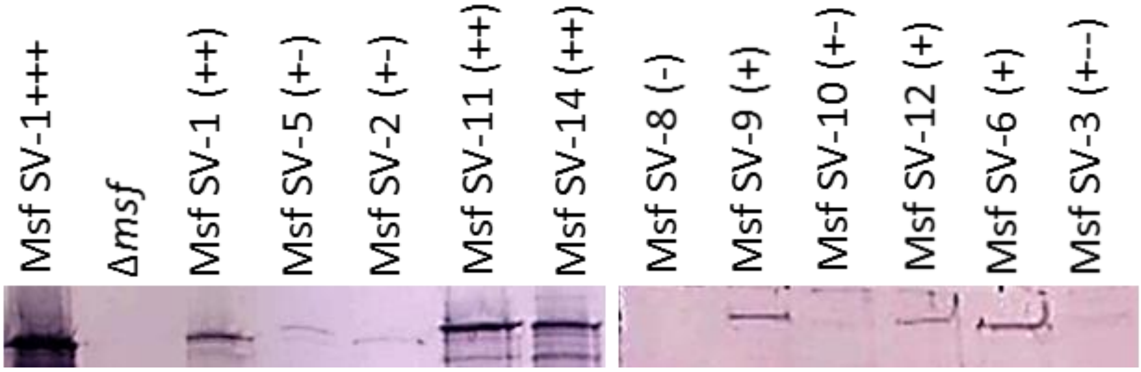

Figure S2. **Msf exhibits varying expression levels in different meningococcal clinical isolates.** Anti-MsfSV1/2/5 was able detect Msf from a range of different clinical isolates expressing different Msf sequence variants; G7/4 Msf ++ (Msf SV-1), H18.18 Δ*msf*, MC58 (SV-1), PMC3 (SV-5), PMC8 (SV-2), PMC9 (SV-11), PMC14 (SV-14), M07 240646 (SV-8), M07 240789 (SV-9), M07 240680 (SV-10), M07 240909 (SV-12), M07 240669 (SV-6), and M07 240949 (SV-3). Western blot results are representative of two independent experiments.
